# Supplementary material for: Cingulin regulates hair cell cuticular plate morphology and is required for hearing in human and mouse
Source: EMBO Mol Med. 2023 Sep 11;15(11):e17611. doi: 10.15252/emmm.202317611 (PMC10630877; doi:10.15252/emmm.202317611)
Supplement: Supplementary file 1 — Appendix [file EMMM-15-e17611-s004.pdf]

Appendix

Cingulin regulates hair cell cuticular plate morphology and is required for hearing in human and mouse

Table of Contents……………………………………………………………………………….….1

Appendix Fig S1. Multipoint linkage analysis and the candidate genetic variants……………………………………………………………………………………......2

Appendix Fig S2. Validation of CGN antibodies……………………………………………...3

Appendix Fig S3. Abnormal expression pattern of the mutant human CGN in various cell lines..…………………………………………………………………………………………5

Appendix Fig S4. Normal cochlear synaptic ribbon densities in the *Cgn*^{delG} mice…………...7

Appendix Fig S5. No effects of abnormal CGN expression on tight junction related markers.8

Appendix Fig S6. Normal microtubule structures in the *Cgn* mutant mice..........................10

Appendix Fig S7. Abnormal hair cell cuticular plate morphology in the *Cgn*^{delG} mice at middle frequency...11

Appendix Table S1. Classification of the different genetic variants identified in *CGN* in Spanish families...12

Appendix Table S2. Primers for RT-qPCR analyses..14

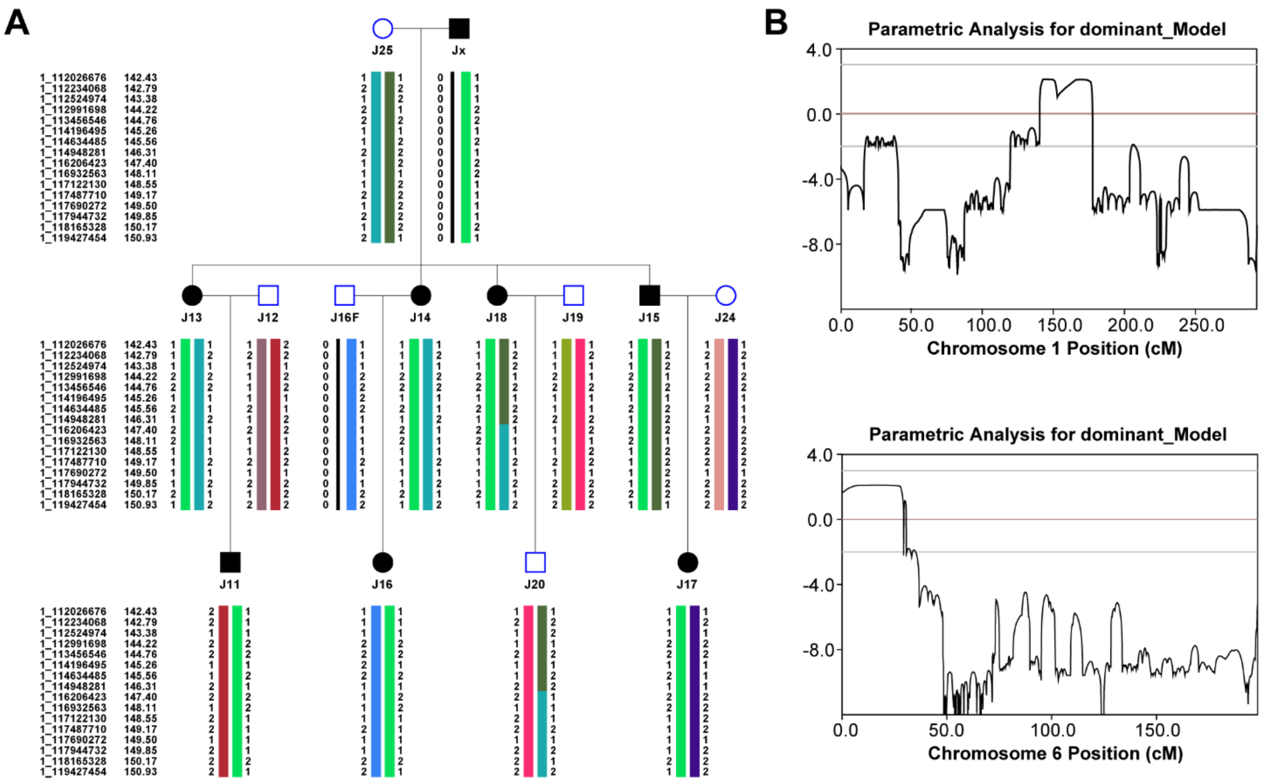

C

CHROM	POS	REF	ALT	Func.refGene	Gene.refGene	ExonicFunc.refGene	cytoBand	avsnp147
chr1	145538716	TCA	T	splicing	*ITGA10*	.	1q21.1	rs587614056
chr1	151509228	TG	T	exonic	*CGN*	frameshift_deletion	1q21.3	.
chr1	154407658	AG	A	intronic	*IL6R*	.	1q21.3	rs761937636
chr1	17084536	TGGAACA	T	exonic	*MST1L*	nonframeshift_deletion	1p36.13	rs142741624
chr6	2971195	A	AG	UTR5	*SERPINB6*	.	6p25.2	rs149382735
chr6	7295300	TA	T	intronic	*SSR1*	.	6p24.3	rs35998405
chr6	3232460	TGGGC	T	intergenic	*TUBB2B;PSMG4*	.	6p25.2	rs10597566

Appendix Fig S1. Multipoint linkage analysis and the candidate genetic variants.

(A) Pedigree and haplotype diagram for linkage region on chromosome 1 of the ADNSHL family. (B) Multipoint linkage analysis results for chromosomes 1 and 6. (C) Candidate genetic variants co-segregated with the ADNSHL phenotype.

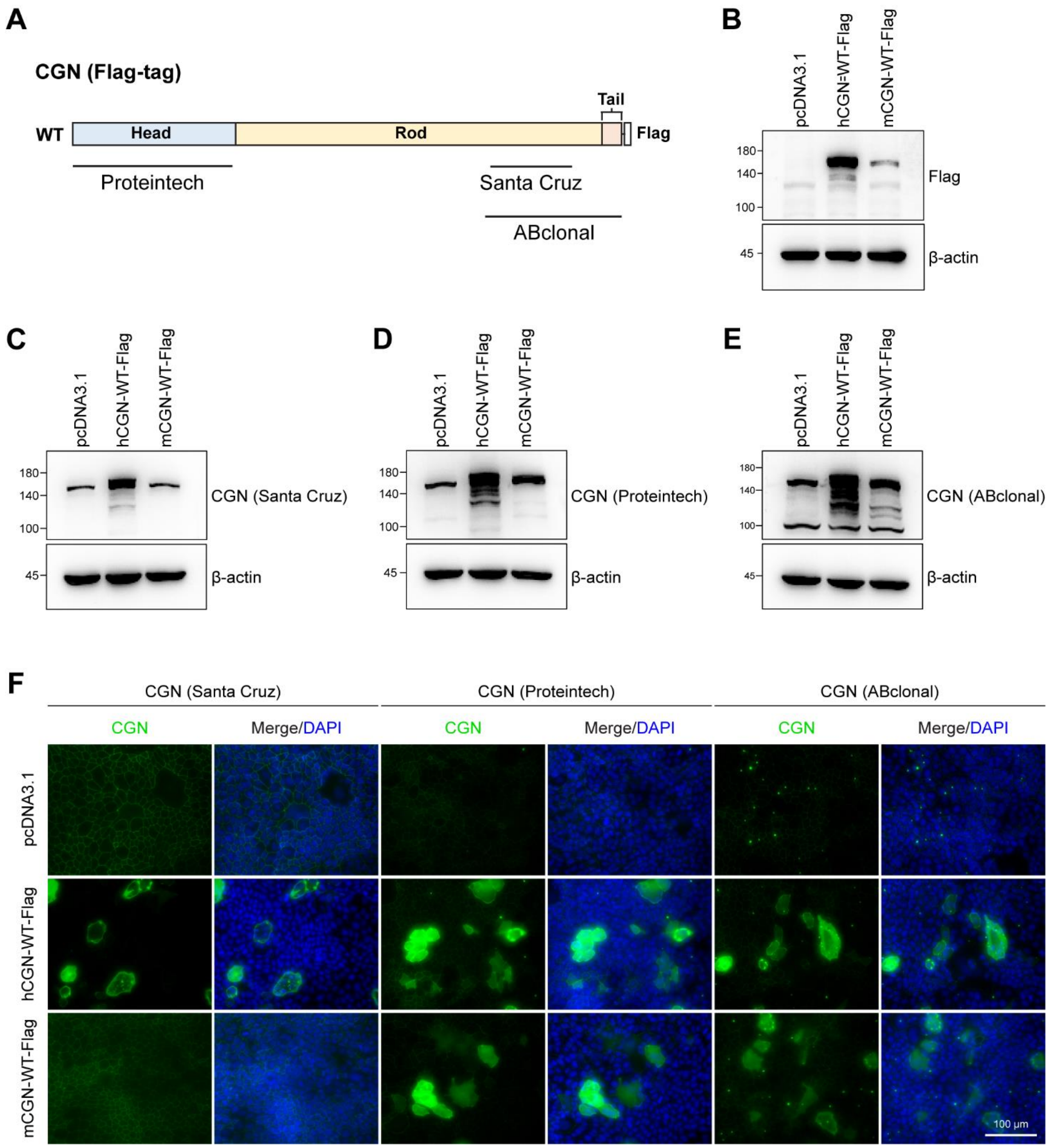

Appendix Fig S2. Validation of CGN antibodies.

(A) A schematic diagram of C-terminal Flag-tagged human or mouse CGN WT constructs (human: hCGN-WT-Flag; mouse: mCGN-WT-Flag) used in this study and antigen recognition sequence of three CGN antibodies. (B) Western blot analysis of whole cell lysates from MDCK cells transfected with WT CGN. Exogenous CGN was immunoblotted with Flag antibody. Both human and mouse exogenous WT CGN were expressed. (C-E) Western blot analysis of whole cell lysates from MDCK cells transfected with WT CGN. CGN was immunoblotted with three different CGN antibodies. (F) MDCK cells expressing WT CGN were immunolabeled with three different CGN antibodies. High magnification immunofluorescent images showing subcellular localizations of WT CGN. According above

results, three different CGN antibodies can recognize the human WT CGN protein sequence, while the monoclonal antibody from Santa Cruz has a lower efficiency for mouse WT CGN protein. Therefore, rabbit anti-Cingulin (Proteintech) and mouse anti-Cingulin (Santa Cruz) were selected for probing CGN protein in cell line samples (Western blot and immunofluorescent). Rabbit anti-Cingulin (Proteintech) has fewer nonspecific bands and was used for Western blot of mouse tissue samples. For mouse cochlear whole mount samples, rabbit anti-Cingulin (ABclonal) showed less background and was used to for immunofluorescence of whole mount samples.

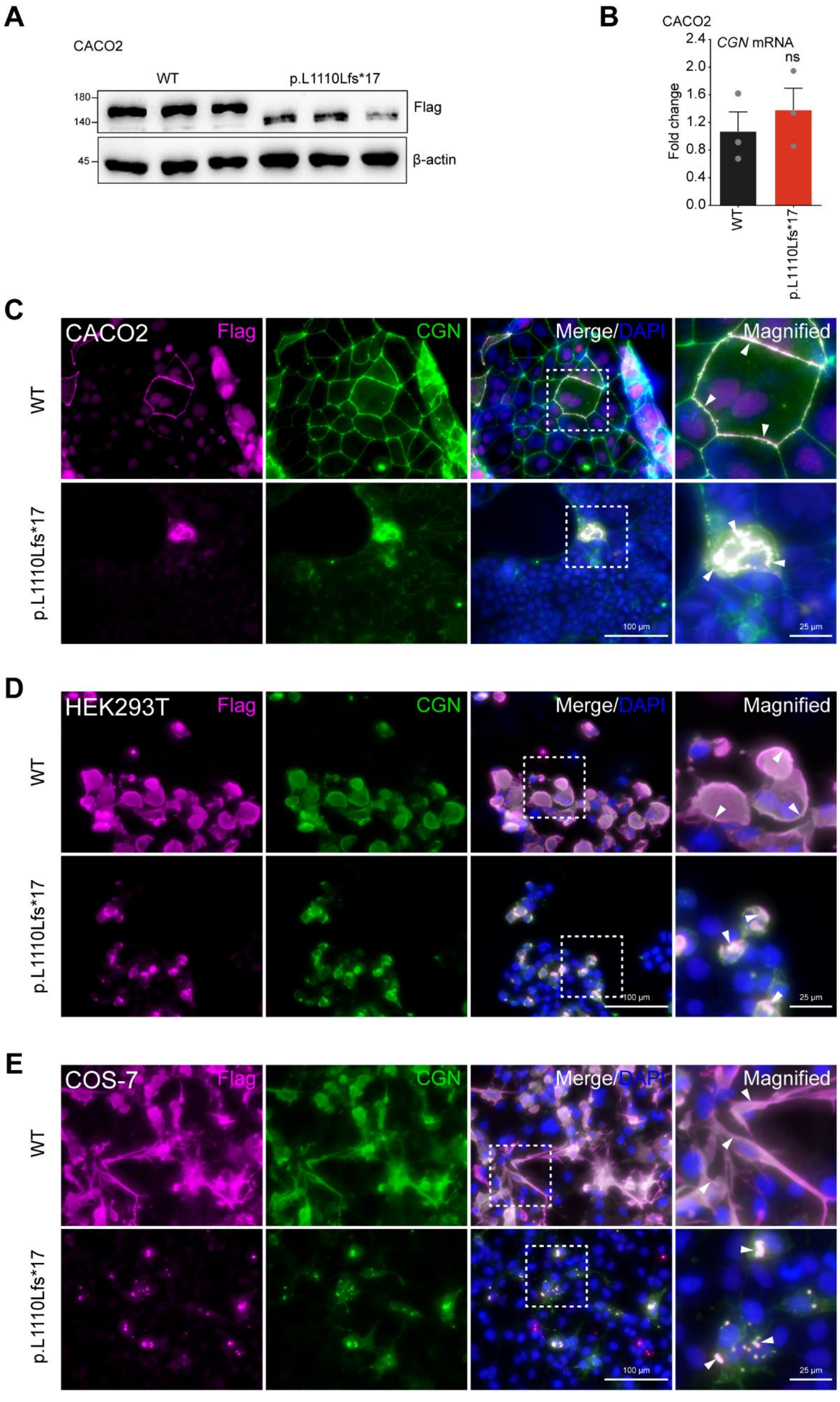

A
CACO2
WT
p.L1110Lfs*17
180
140
Flag
45
β-actin
B
CACO2
CGN mRNA
ns
Fold change
2.4
2.0
1.6
1.2
0.8
0.4
0.0
WT
p.L1110Lfs*17
C
CACO2
Flag
CGN
Merge/DAPI
Magnified
WT
p.L1110Lfs*17
100 µm
25 µm
D
HEK293T
Flag
CGN
Merge/DAPI
Magnified
WT
p.L1110Lfs*17
100 µm
25 µm
E
COS-7
Flag
CGN
Merge/DAPI
Magnified
WT
p.L1110Lfs*17
100 µm
25 µm

Appendix Fig S3. Abnormal expression pattern of the mutant human CGN in various cell lines.

(A) Western blot analysis of whole cell lysates from CACO2 cells transfected with WT or mutant CGN. Exogenous CGN was immunoblotted with Flag antibody. (B) RT-qPCR of *CGN* expression in transfected CACO2 cells. N = 3. Error bars represent ± SEM. ns, $P > 0.05$ by unpaired student's t-test. (C-E) Immunofluorescence of CGN expression in CACO2 (C), HEK293T (D) or COS-7 (E) cells transfected with WT or mutant CGN. Localizations of WT or mutant CGN (white arrows) were visualized with CGN or Flag antibodies.

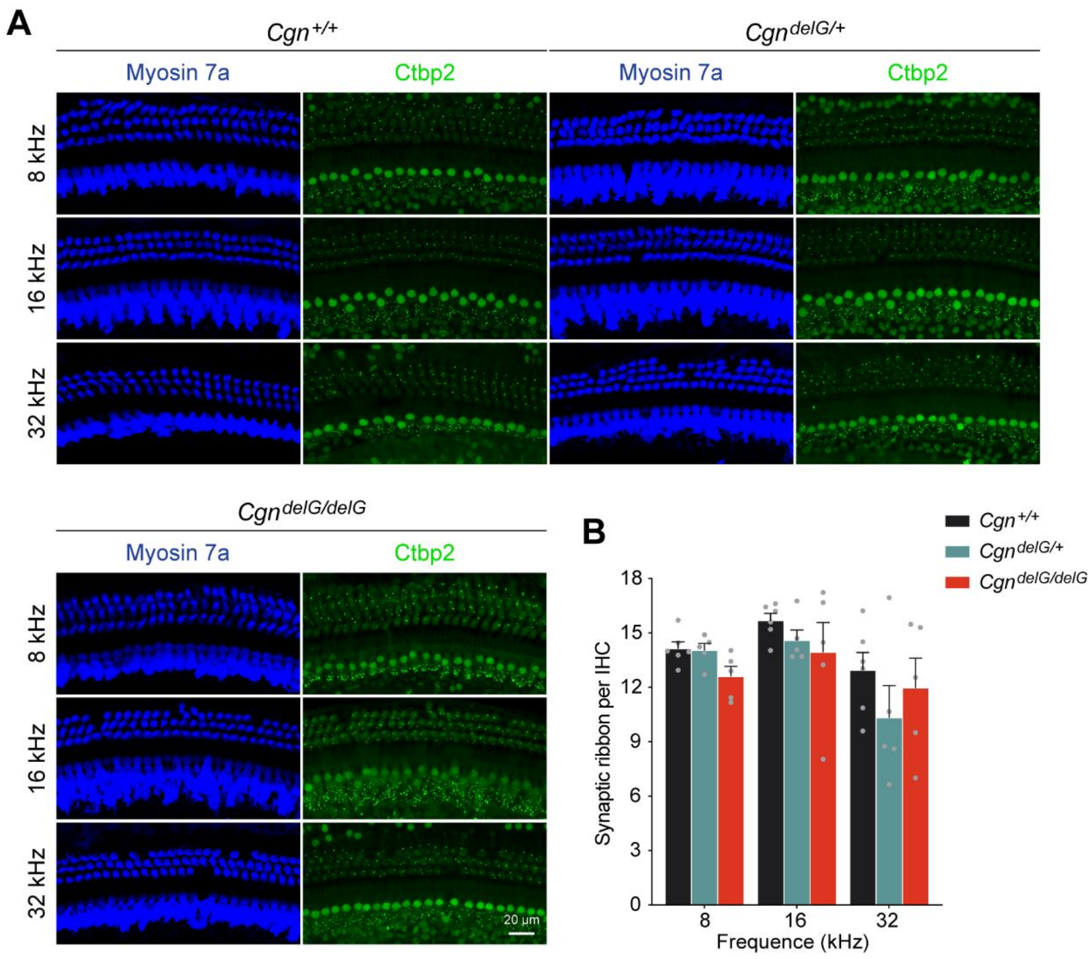

Appendix Fig S4. Normal cochlear synaptic ribbon densities in the *Cgn^delG^* mice.

(A) Whole mount immunofluorescence of cochlear synaptic ribbons by labeling Ctbp2 (presynaptic ribbons marker, green) and Myosin 7a (blue) of the 2-months old *Cgn*^{delG} mice. (B) Densities of synaptic ribbons in 2-months old *Cgn*^{delG} mice. N = 5-6. Error bars represent ± SEM. $P > 0.05$ by two-way ANOVA.

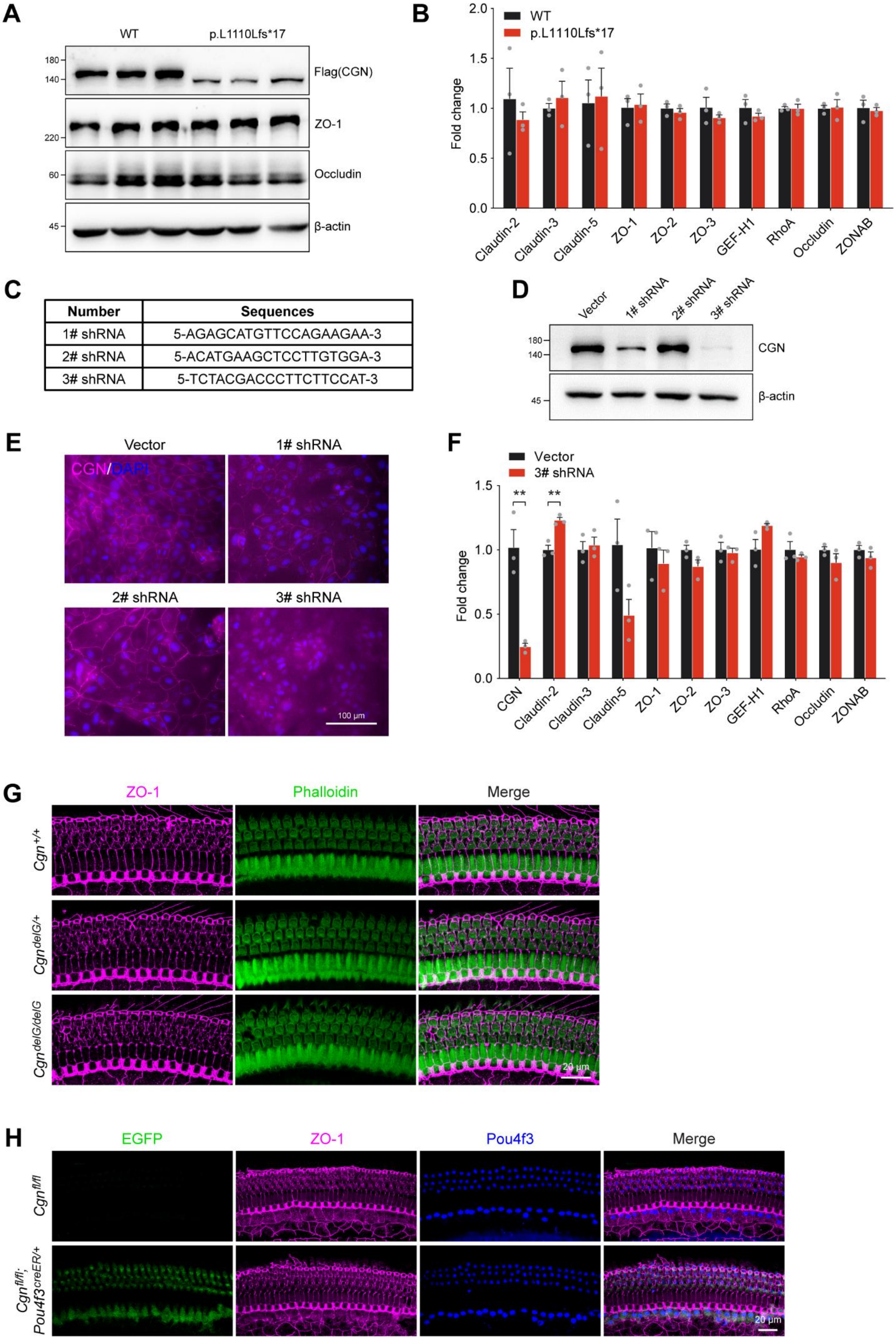
A
WT
p.L1110Lfs*17
Flag(CGN)
ZO-1
Occludin
β-actin
B
WT
p.L1110Lfs*17
Fold change
Claudin-2
Claudin-3
Claudin-5
ZO-1
ZO-2
ZO-3
GEF-H1
RhoA
Occludin
ZONAB
C
Number | Sequences
1# shRNA | 5-AGAGCATGTTCCAGAAGAA-3
2# shRNA | 5-ACATGAAGCTCCTTGTGGA-3
3# shRNA | 5-TCTACGACCCTTCTTCCAT-3
D
Vector
1# shRNA
2# shRNA
3# shRNA
CGN
β-actin
E
Vector
1# shRNA
CGN/DAPI
2# shRNA
3# shRNA
100 μm
F
Vector
3# shRNA
Fold change
CGN
Claudin-2
Claudin-3
Claudin-5
ZO-1
ZO-2
ZO-3
GEF-H1
RhoA
Occludin
ZONAB
G
ZO-1
Phalloidin
Merge
Cgn+/+
CgndelG/+
CgndelG/delG
20 μm
H
EGFP
ZO-1
Pou4f3
Merge
Cgnfl/fl
Cgnfl/fl; Pou4f3creER/+
20 μm

Appendix Fig S5. No effects of abnormal CGN expression on tight junction related markers.

(A) Western blot analyses of the endogenous ZO-1 and Occludin protein expression in MDCK cells transfected with WT or mutant CGN. (B) RT-qPCR of tight junction related markers in MDCK cells transfected with WT or mutant CGN. Error bars represent ± SEM. P > 0.05 by unpaired student's t-test. (C) shRNA sequences for knocking down *Cgn* in MDCK cells. (D, E) Western blot (D) and immunofluorescence (E) validation of Cgn knockdown from *Cgn-KD* MDCK cells. (F) RT-qPCR of tight junction related markers in *Cgn-KD* MDCK cells by *Cgn* shRNA (3#). N = 3. Error bars represent ± SEM. ** P < 0.01 by unpaired student's *t*-test. (G) Cochlear whole mount immunofluorescence of tight junctions labeled with ZO-1 (magenta) from 2-months old *Cgn*^{delG} mice. (H) Cochlear whole mount immunofluorescence of the tight junctions labeled with ZO-1 (magenta) from 2-months old *Cgn-cKO* mice. Hair cells were visualized with Pou4f3 (blue) or Pou4f3-driven EGFP (green).

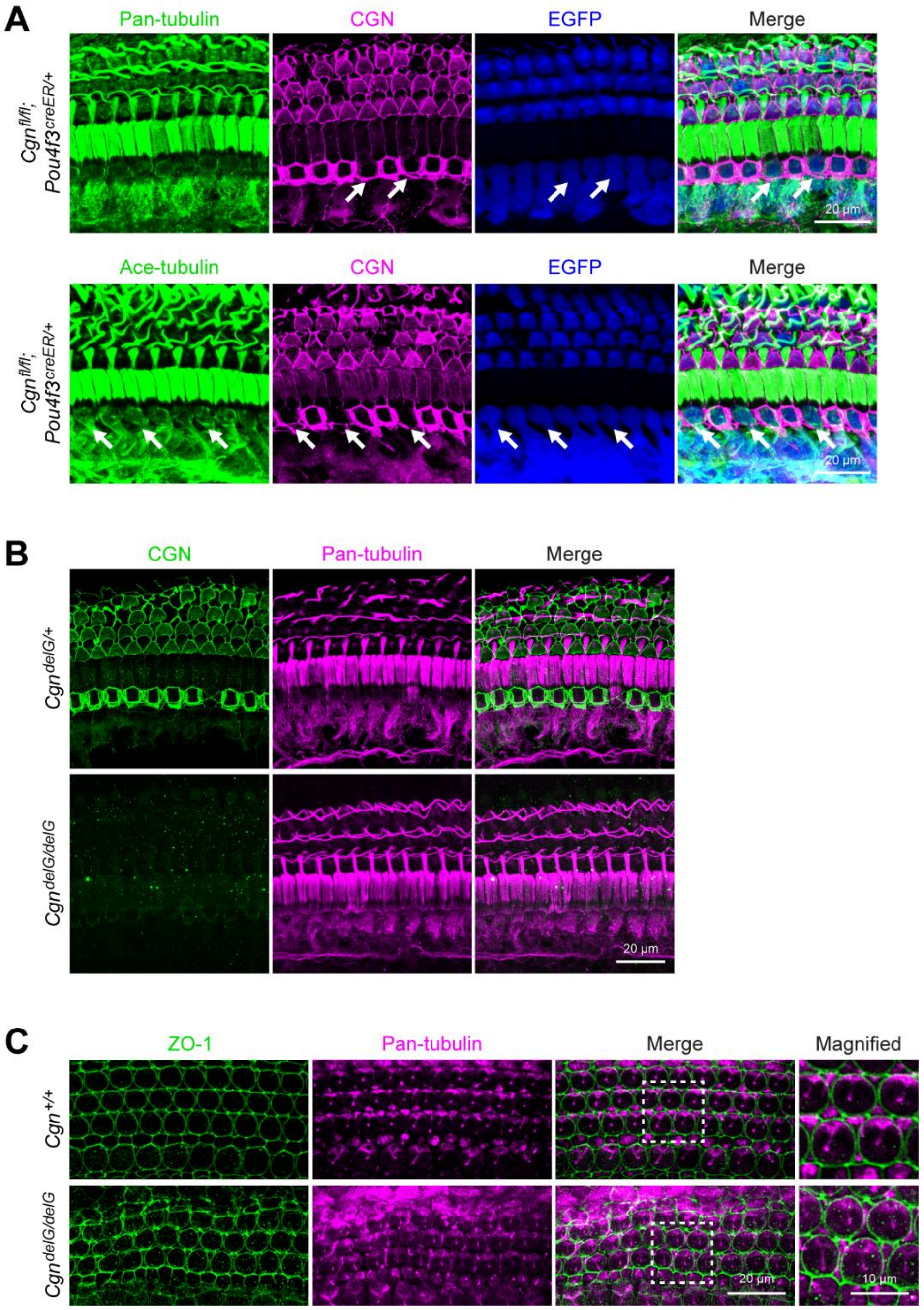

Appendix Fig S6. Normal microtubule structures in the *Cgn* mutant mice.

(A) Cochlear whole mount immunofluorescence of microtubules by labeling pan-tubulin or acetylated-tubulin (green) from 2-months old *Cgn-cKO* mice. White arrows label the recombined IHCs. (B) Cochlear whole mount immunofluorescence of microtubules by labeling pan-tubulin (magenta) from 7-months old *Cgn*^{delG} mice. (C) Cochlear whole mount immunofluorescence of kinocilium by labeling pan-tubulin (magenta) from P0 *Cgn*^{delG} mice.

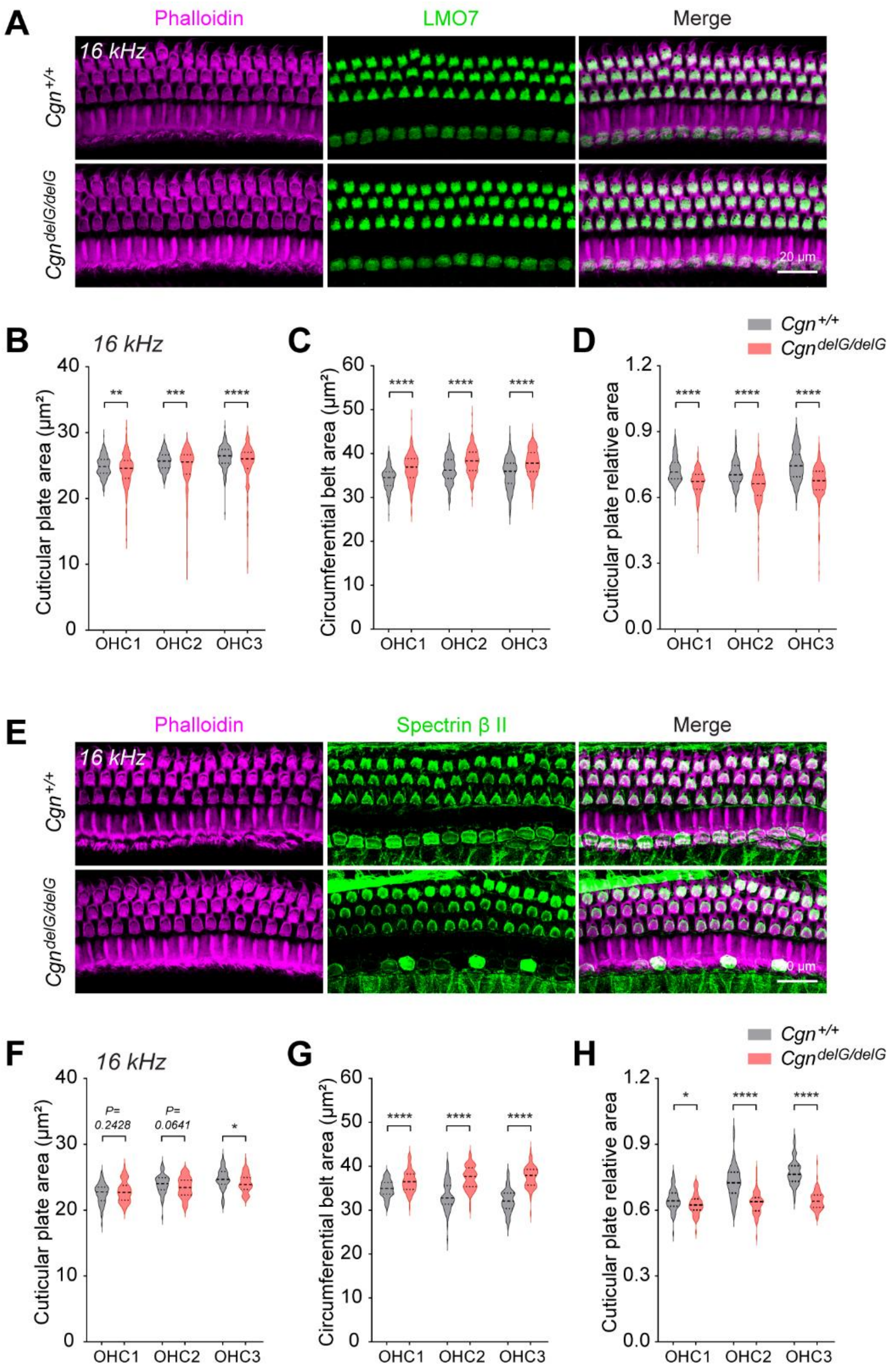

Appendix Fig S7. Abnormal hair cell cuticular plate morphology in the *Cgn*^{delG} mice at middle frequency.

(A, E) Whole mount immunofluorescence of hair cell cuticular plates by labeling (A) LMO7 or (E) Spectrin β II from 2-months old *Cgn*^{delG} mice at 16 kHz cochlear region. (B, F) Areas of the cuticular plates, (C, G) areas of the circumferential belts and (D, H) relative areas of the cuticular plates quantified from immunofluorescent images obtained by (B-D) LMO7 (N = 186-195 hair cells) or (F-H) Spectrin β II (N = 48-64 hair cells). * P < 0.05, ** P < 0.01, *** P < 0.001 and **** P < 0.0001 by unpaired student's *t*-test.

Appendix Table S1. Classification of the different genetic variants identified in *CGN* in Spanish families.

DNA change / Protein change	Coding impact	Transcript variant	Exon	Origin	ACMG classification	CSVS Allele Freq	GnomAD Allele Freq	Number of occurrences
c.31C>T p.Arg11Trp	Missense	NM_020770.3	2/21	Spanish	Benign (BS1, BS2, BP4, BP6, BP1)	0.0065	0.00275	1/96
c.586C>T p.Arg196Trp	Missense	NM_020770.3	2/21	Spanish	Likely Benign (PM2, BP4,BP1)	0.001	0.000331	1/96
c.1640G>A p.Arg547Lys	Missense	NM_020770.3	9/21	Spanish	Benign (BS1, BS2, BP4, BP6, BP1)	0.01	0.00654	2/96
c.2311G>A p.Glu771Lys	Missense	NM_020770.3	12/21	Spanish	Benign (BS1, BS2, BP4, BP6, BP1)	0.017	0.007	2/96
c.2420G>A p.Arg807Gln	Missense	NM_020770.3	13/21	Spanish	Benign (BS1, BS2, BP1, BP4)	0.341	0.00389	2/96
c.2500C>T Arg834Trp	Missense	NM_020770.3	13/21	Spanish	Likely Benign (PM2, BP4,BP1)	0.00050	0.000139	1/96
c.2650C>T p.Arg884Trp	Missense	NM_020770.3	14/21	Spanish	Likely Benign (PM2, BP4,BP1)	0.001	0.000162	1/96
c.3113A>G p.Glu1038Gly	Missense	NM_020770.3	18/21	Spanish	Likely Benign (PM2, BP4,BP1	0	0.000573	1/96

All the variants are named according to NM_020770.3 transcript. The nomenclature was checked using Mutalyzer 2.0.34 (Wildeman, van Ophuizen et al., 2008). ACMG criteria (Kopanos, Tsiolkas et al., 2019, Richards, Aziz et al., 2015): **PM2** (Pathogenic, Moderate): absent from controls (or at extremely low frequency if recessive) in Exome Sequencing Project,

1000 Genomes Project, or Exome Aggregation Consortium. **BS1** (Benign, Strong): allele frequency is greater than expected for disorder. **BS2** (Benign, Strong): observed in a healthy adult individual for a recessive (homozygous), dominant (heterozygous), or X-linked (hemizygous) disorder, with full penetrance expected at an early age. **BP1** (Benign, Supporting): missense variant in a gene for which primarily truncating variants are known to cause disease. **BP4** (Benign, Supporting): multiple lines of computational evidence suggest no impact on gene or gene product (conservation, evolutionary, splicing impact, etc.). **BP6** (Benign, Supporting): Reputable source recently reports variant as benign, but the evidence is not available to the laboratory to perform an independent evaluation. The databases GnomAD (Karczewski, Francioli et al., 2020) and CSVS (Peña-Chilet, Roldán et al., 2020) were searched on the 17th of February 2023. N.A: not available.

References

Karczewski KJ, Francioli LC, Tiao G, Cummings BB, Alföldi J, Wang Q, Collins RL, Laricchia KM, Ganna A, Birnbaum DP, Gauthier LD, Brand H, Solomonson M, Watts NA, Rhodes D, Singer-Berk M, England EM, Seaby EG, Kosmicki JA, Walters RK et al. (2020) The mutational constraint spectrum quantified from variation in 141,456 humans. *Nature* 581: 434-443

Kopanos C, Tsiolkas V, Kouris A, Chapple CE, Albarca Aguilera M, Meyer R, Massouras A (2019) VarSome: the human genomic variant search engine. *Bioinformatics* 35: 1978-1980

Peña-Chilet M, Roldán G, Perez-Florido J, Ortuño FM, Carmona R, Aquino V, Lopez-Lopez D, Loucera C, Fernandez-Rueda JL, Gallego A, García-Garcia F, González-Neira A, Pita G, Núñez-Torres R, Santoyo-López J, Ayuso C, Minguez P, Avila-Fernandez A, Corton M, Moreno-Pelayo M et al. (2020) CSVS, a crowdsourcing database of the Spanish population genetic variability. *Nucleic Acids Res*

Richards S, Aziz N, Bale S, Bick D, Das S, Gastier-Foster J, Grody WW, Hegde M, Lyon E, Spector E, Voelkerding K, Rehm HL, Committee ALQA (2015) Standards and guidelines for the interpretation of sequence variants: a joint consensus recommendation of the American College of Medical Genetics and Genomics and the Association for Molecular Pathology. *Genet Med* 17: 405-24

Wildeman M, van Ophuizen E, den Dunnen JT, Taschner PE (2008) Improving sequence variant descriptions in mutation databases and literature using the Mutalyzer sequence variation nomenclature checker. *Hum Mutat* 29: 6-13

Appendix Table S2. Primers for RT-qPCR analyses.

Genes	Primers	Sequences
Mouse *Gapdh*	primer F	5’-ACCACGAGAAATATGACAACTCAC-3’
	primer R	5’-CCAAAGTTGTCATGGATGACC-3’
Human *Gapdh*	primer F	5’-ATGACAACAGCCTCAAGAT-3’
	primer R	5’-GAGTCCTTCCACGATACC-3’
Canis *β-actin*	primer F	5’-GGACCTCTATGCCAACAC-3’
	primer R	5’-TGCGATGATCTTGATCTTCA-3’
EGFP	primer F	5’-TAAACGGCCACAAGTTCAGC-3’
	primer R	5’-GAACTTCAGGGTCAGCTTGC-3’
Human *Cingulin*	primer F	5’-CCAACCACTGGACCTCTA-3’
	primer R	5’-TCTGACGAGAACGGCTAA-3’
Canis *Cingulin*	primer F	5’-GTCCTTCAGTCCACCAAC-3’
	primer R	5’-TCGCTCAATCTCCTCTTCT-3’
Mouse *Cingulin*	primer F	5’-CAACTGCGGATGGAGAAG-3’
	primer R	5’-AACCTGGTGAGTATCTCTTGTA-3’
Canis *Claudin-2*	primer F	5’-CCGACTACTATGACTCCTACC-3’
	primer R	5’-TAAACTCGCTCTTGGCTTTG-3’
Canis *Claudin-3*	primer F	5’-GTGCAAGGTGTACGACTC-3’
	primer R	5’-CAGGATGGACACGACGAT-3’
Canis *Claudin-5*	primer F	5’-CCTTCCTGGACCACAACAT-3’
	primer R	5’-CCGAGTCGTACACCTTGC-3’
Canis *ZO-1*	primer F	5’-CGCAGTCCTATTCTTCAG-3’
	primer R	5’-ACTTCTGGCTTATGTTGAG-3’
Canis *ZO-2*	primer F	5’-GCACAGAGAACAGCAAGGA-3’
	primer R	5’-CACCAGCCAATCGGAGTC-3’
Canis *ZO-3*	primer F	5’-CTCATCCTACAGATCAATGGT-3’
	primer R	5’-CCCTCGGACTTCTCAATC-3’
Canis *GEF-H1*	primer F	5’-TAACAAGAGCATCACAGCCAAG-3’
	primer R	5’-TTCAGCAGAGCAGCCTTC-3’
Canis *RhoA*	primer F	5’-TGGTGATTGTTGGTGATGGA-3’
	primer R	5’-TCAATATCTGCCACATAGTTCTCA-3’
Canis *Occludin*	primer F	5’-CCAGGAGTAATTCGGATTCT-3’
	primer R	5’-CATTAAGCCAGTTCCATAGC-3’
Canis *ZONAB*	primer F	5’-ACTGCCATCAAGAAGAATAAC-3’
	primer R	5’-CGTCTGCCATAGTAACCA-3’
