# Supplementary material for: Cingulin regulates hair cell cuticular plate morphology and is required for hearing in human and mouse
Source: EMBO Mol Med. 2023 Sep 11;15(11):e17611. doi: 10.15252/emmm.202317611 (PMC10630877; doi:10.15252/emmm.202317611)
Supplement: Supplementary file 2 — Expanded View Figures PDF [file EMMM-15-e17611-s011.pdf]

Expanded View Figures

Figure EV1. Audiograms of the individuals within the family.

A Audiograms of the individuals affected by hearing loss.
B Audiograms of the individuals without hearing loss. Individual II:2 developed unilateral hearing loss at age 30 due to chronic suppurative otitis media of the right ear.

Source data are available online for this figure.

© 2023 The Authors

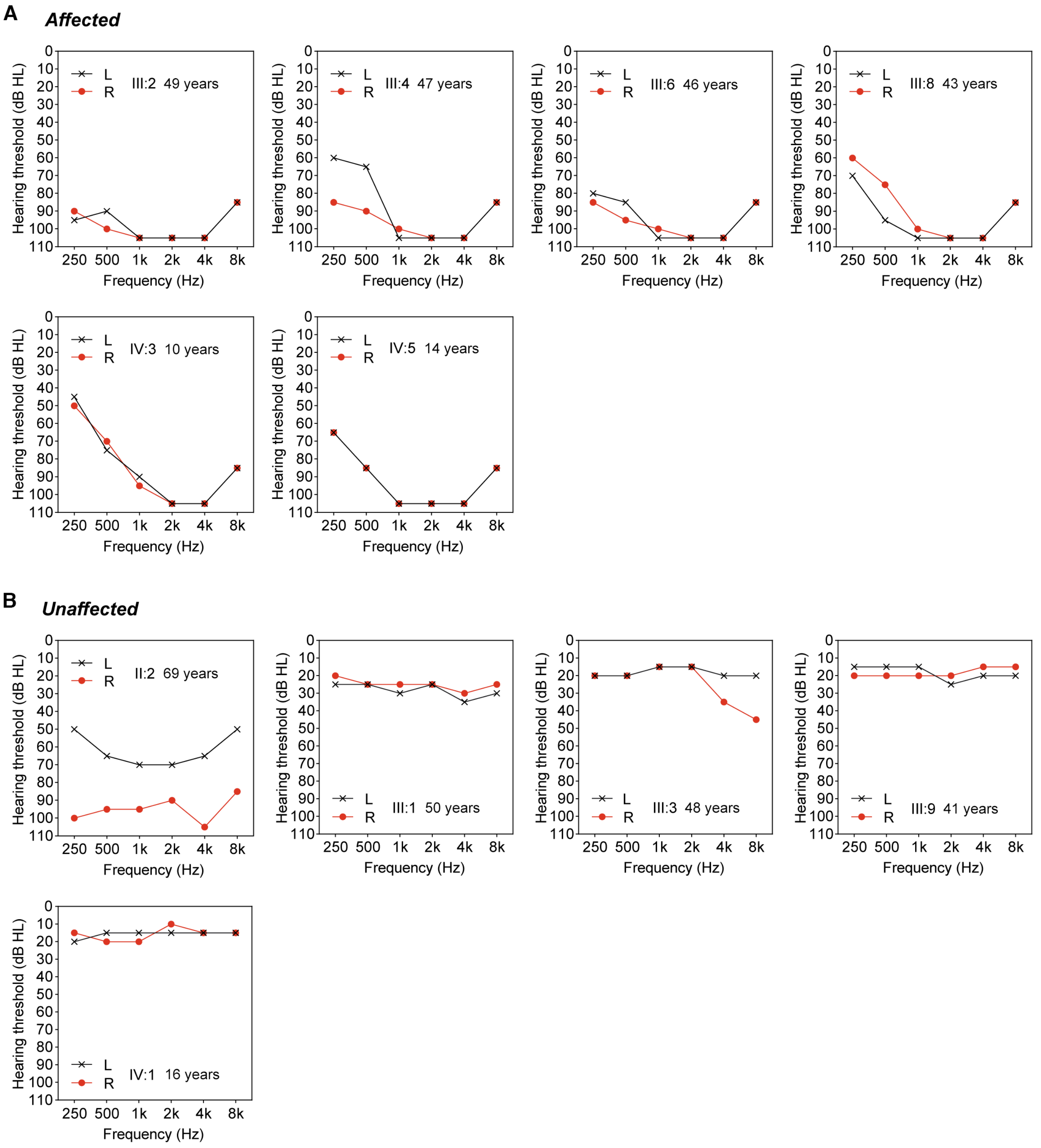

Figure EV1.

© 2023 The Authors

Figure EV2. Abnormal expression pattern of N-terminal EGFP-tagged mutant human CGN.

A A schematic diagram of N-terminal EGFP-tagged WT and mutant (p.L1110Lfs*17) CGN constructs used in this study.
B MDCK cells expressing EGFP-tagged WT or mutant CGN were immunolabeled with CGN or EGFP antibodies.
C Western blot analysis of whole cell lysates from MDCK cells transfected with EGFP-tagged WT or mutant CGN. Exogenous EGFP-CGN fusion protein was immunoblotted with EGFP antibody.
D RT-qPCR of *CGN* expression in transfected MDCK cells ($n = 3$ biological replicates).
E High-magnification immunofluorescent images showing subcellular localizations of EGFP-tagged WT and mutant CGN (white arrows).
F HEK293T cells expressing EGFP-tagged WT or mutant CGN were immunolabeled with EGFP antibodies. Arrow heads indicate subcellular localization of the CGN proteins.
G Western blot analysis of whole cell lysates from HEK293T cells transfected with EGFP-tagged WT or mutant CGN. Exogenous EGFP-CGN fusion protein was immunoblotted with EGFP antibody.

Data information: Data are presented as mean ± SEM; unpaired Student's *t*-test was used in (D). ns, not significant, $P > 0.05$.
Source data are available online for this figure.

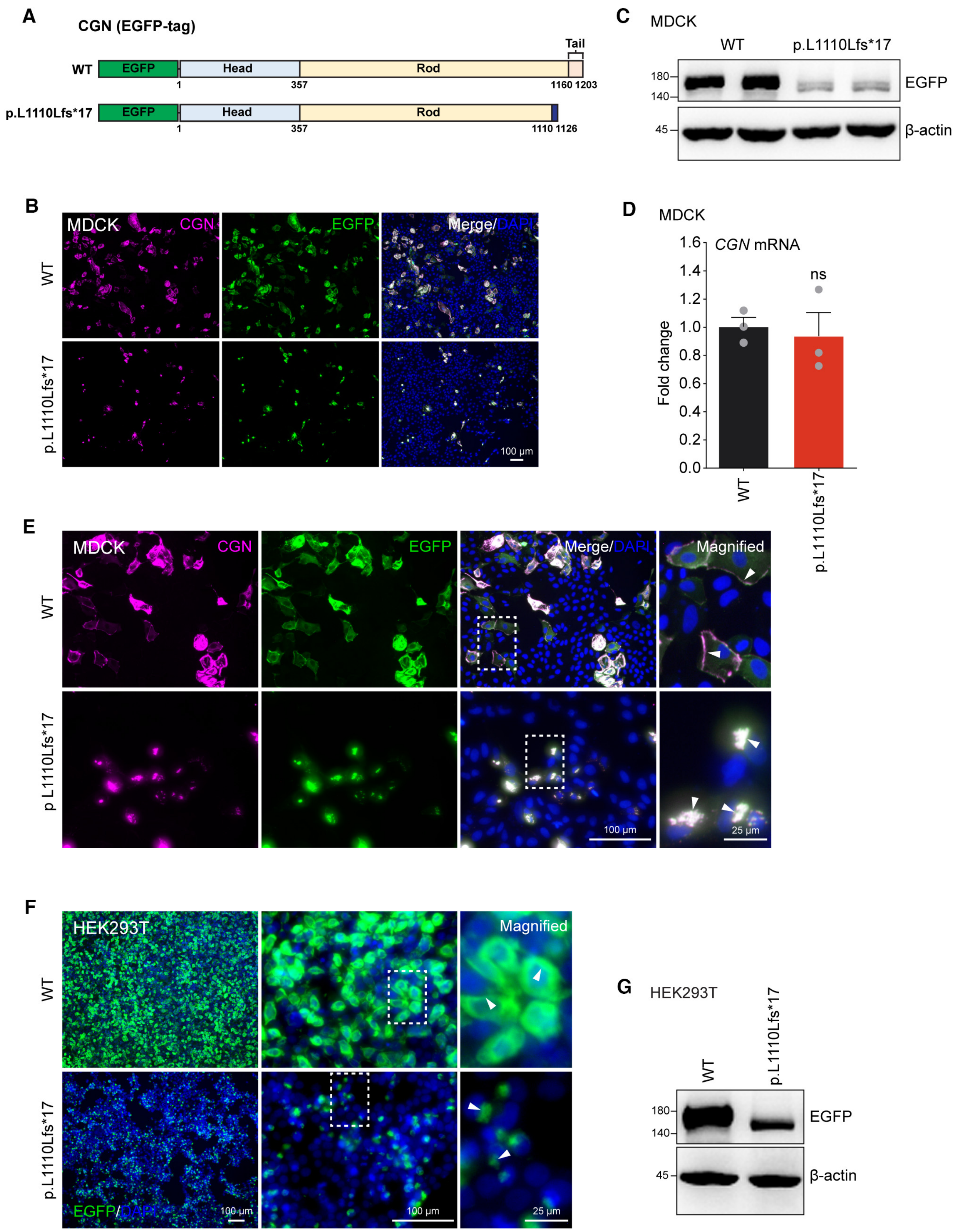

Figure EV2.

 © 2023 The Authors

Figure EV3. Generation and validation of the *Cgn-cKO and Cgn^{delG} knockin mice.*

A Strategy to generate hair cell-specific *Cgn-cKO* mice by crossing *Cgn$^{fl/fl}$* with *Pou4f3$^{EGFP\text{-}creER}$* (*Pou4f3^{creER}*) mice.

B Genotyping of the LoxP knockin allele in *Cgn$^{fl/fl}$* and the creER knockin allele in *Pou4f3^{creER}* mice. A 398-bp size band can be detected in the genomic DNA from *Cgn$^{fl/fl}$* mice. A 504-bp size band can be detected in the genomic DNA from *Pou4f3^{creER}* mice.

C Cochlear whole mount immunofluorescence to validate efficiency of Cre-mediated recombination and *Cgn* knockout by labeling Pou4f3-driven EGFP (hair cells, green) and CGN (magenta) of 2-month-old *Cgn-cKO* mice. Examples of un-recombined hair cells (asterisks) and the neighboring recombined hair cells (arrowheads) were shown.

D Efficiency of Cre-mediated recombination and CGN knockout in IHCs and OHCs from 2-month-old *Cgn-cKO* cochlea (n = 4–22 biological replicates from 2 to 5 cochleae).

E Strategy to generate *Cgn^{delG}* mice by CRISPR-Cas9 technology.

F Sanger sequencing of the genomic DNA from *Cgn$^{+/+}$* and *Cgn$^{delG/delG}$* mice. Arrow indicates the G-base deleted from the *Cgn$^{delG/delG}$* mouse genome.

G Genotyping of *Cgn^{delG}* mice using wild-type and knockin-specific primers.

Data information: Data are presented as mean ± SEM; unpaired Student's t-test was used in (D). ****$P < 0.0001$.
Source data are available online for this figure.

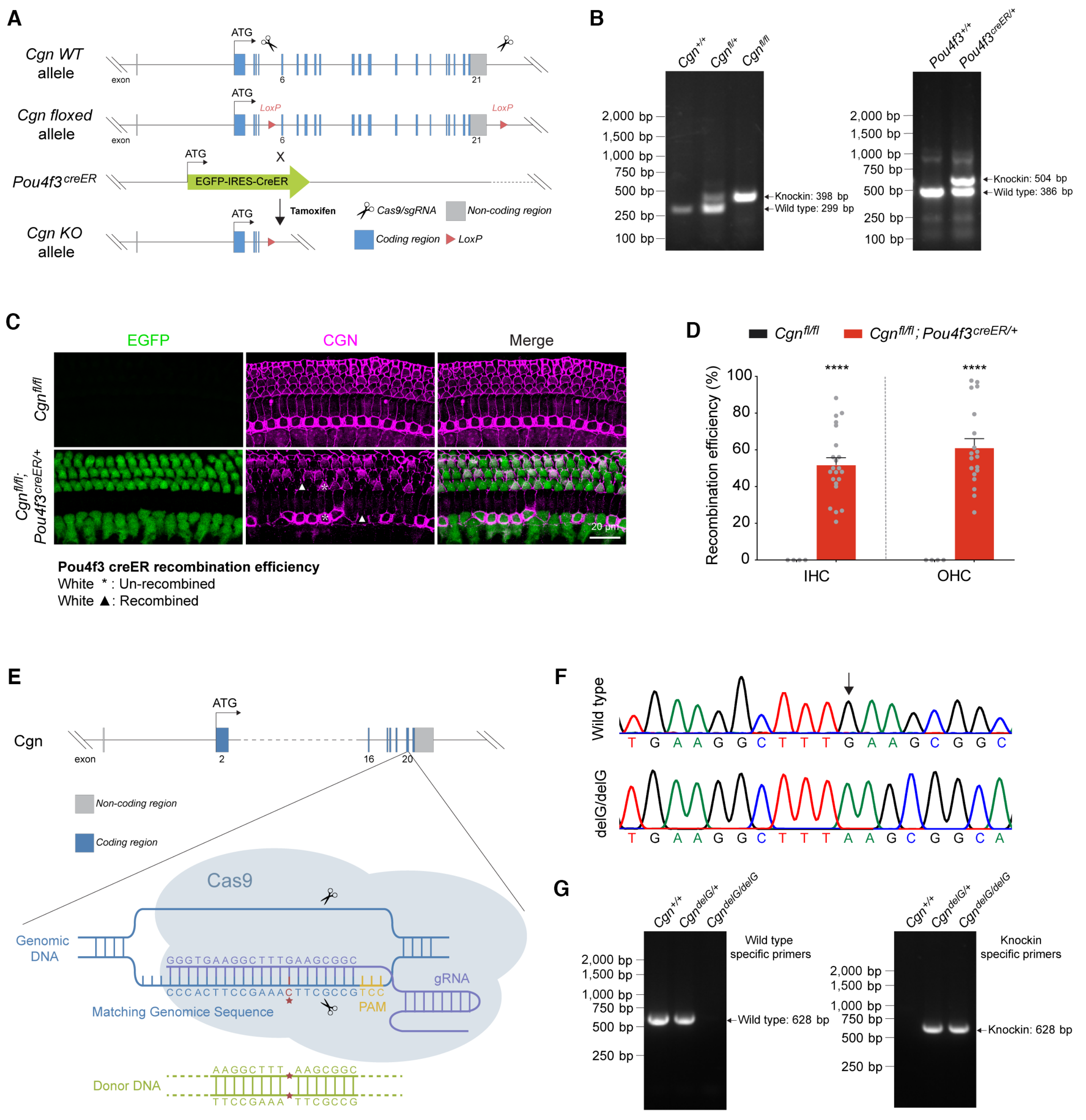

Figure EV3.

© 2023 The Authors

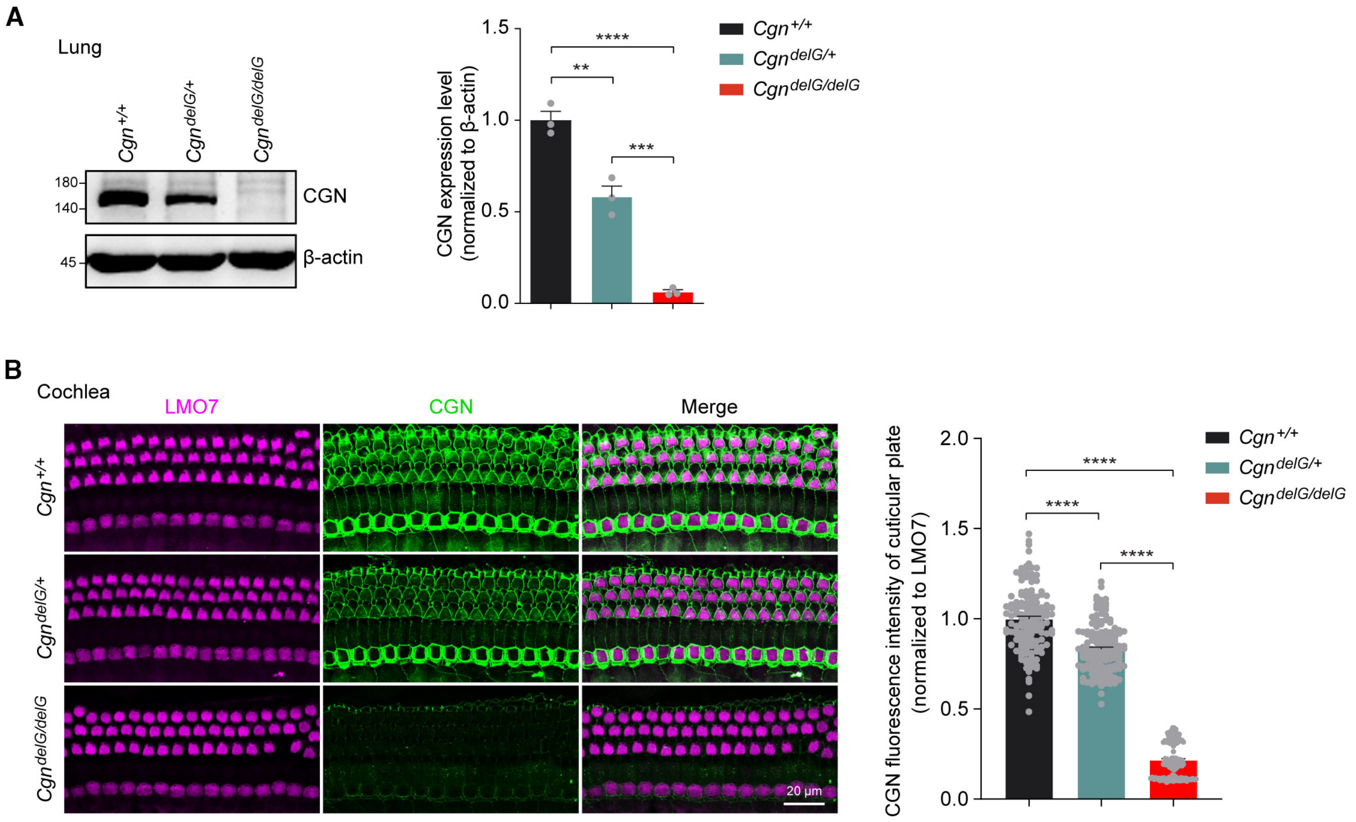

Figure EV4. Abnormal CGN expression in lung and cochlear sensory epithelia of *Cgn*^{delG} mice.

A Western blot and quantification of CGN protein expression in P21 *Cgn*^{delG} lung lysates ($n = 3$ biological replicates).

B Whole mount immunofluorescence of CGN expression in P21 *Cgn*^{delG} mouse cochlea coimmunostained with LMO7. CGN expression levels at the cuticular plates were normalized to LMO7 immunofluorescent signals ($n = 143$–148 hair cells from three cochleae).

Data information: Data are presented as mean ± SEM; one-way ANOVA was used in (A and B). $^{**}P < 0.01$, $^{***}P < 0.001$, and $^{****}P < 0.0001$.
Source data are available online for this figure.

Figure EV5. Expression and function of CGN in mouse utricles.

A, B Whole mount immunofluorescence of CGN expression in P3, P14, and P21 mouse utricle coimmunostained with Parvalbumin (A) or LMO7 (B).

C Whole mount immunofluorescence of CGN expression in utricles from P21 *Cgn*^{delG} mouse.

D The time to fall from the rotarod of the 2-month-old wildtype and *Cgn*^{delG} mice. No significant difference was observed with all three testing protocols ($n = 3$–10 mice).

Data information: Data are presented as mean ± SEM; one-way ANOVA was used in (D). not significant, $P > 0.05$.
Source data are available online for this figure.

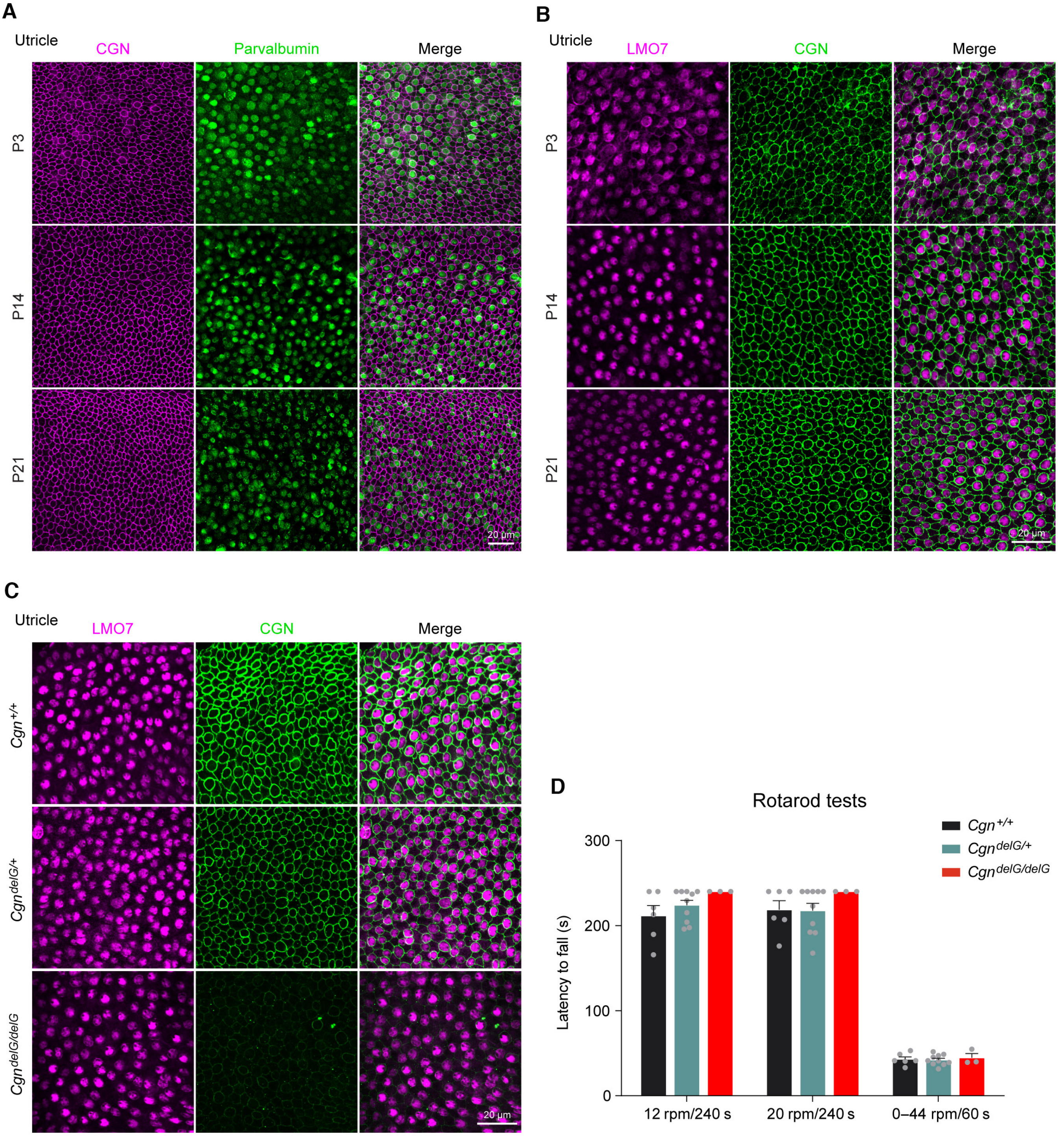

Figure EV5.

 © 2023 The Authors
