# Supplementary figures and images for: Cingulin regulates hair cell cuticular plate morphology and is required for hearing in human and mouse (part 1 of 2)
Source: EMBO Mol Med. 2023 Sep 11;15(11):e17611. doi: 10.15252/emmm.202317611 (PMC10630877; doi:10.15252/emmm.202317611)

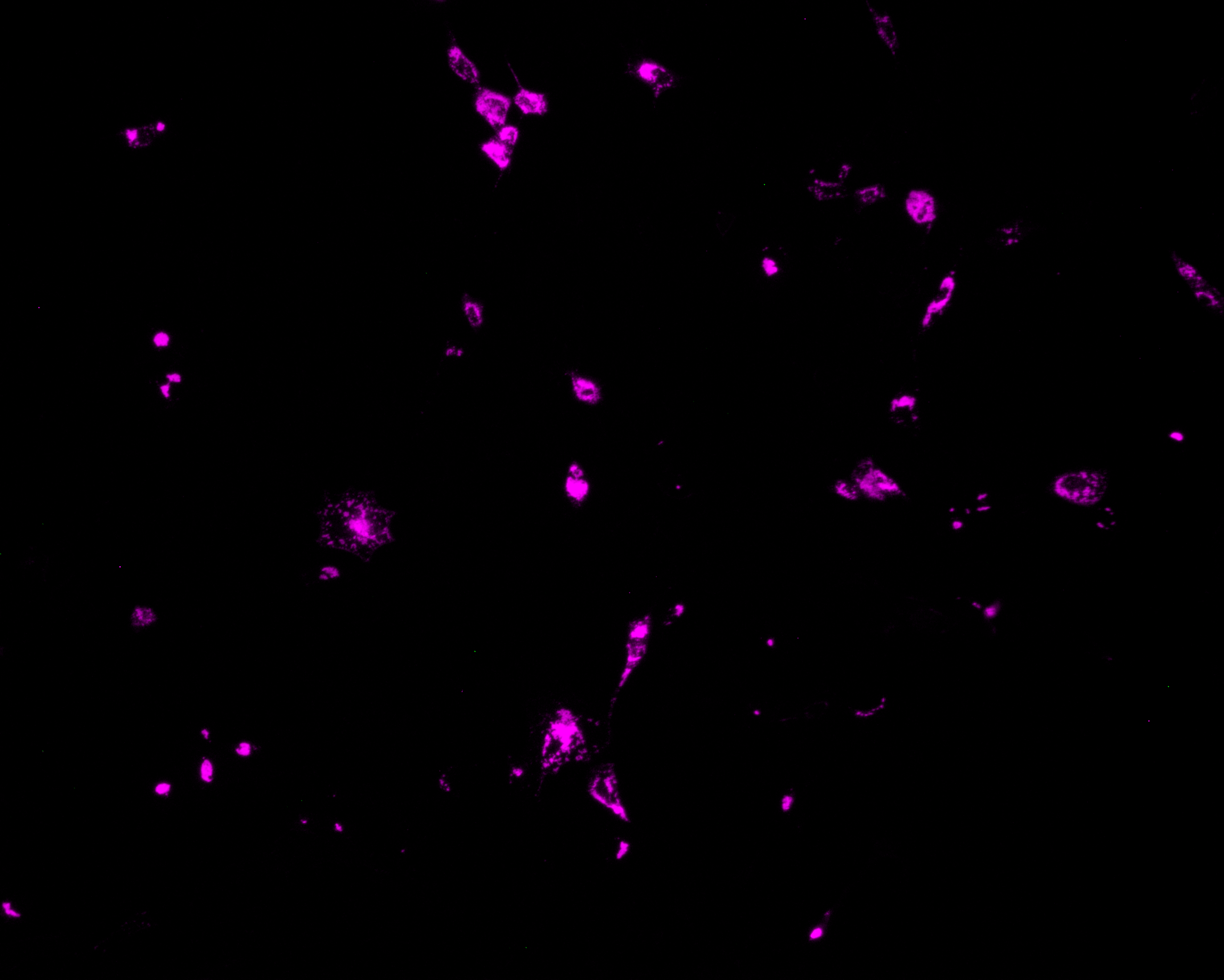

Supplement: Supplementary file 3 — Source Data for Expanded View [file EMMM-15-e17611-s013.zip › Figure EV2/EV2B/Mut CGN.tif]

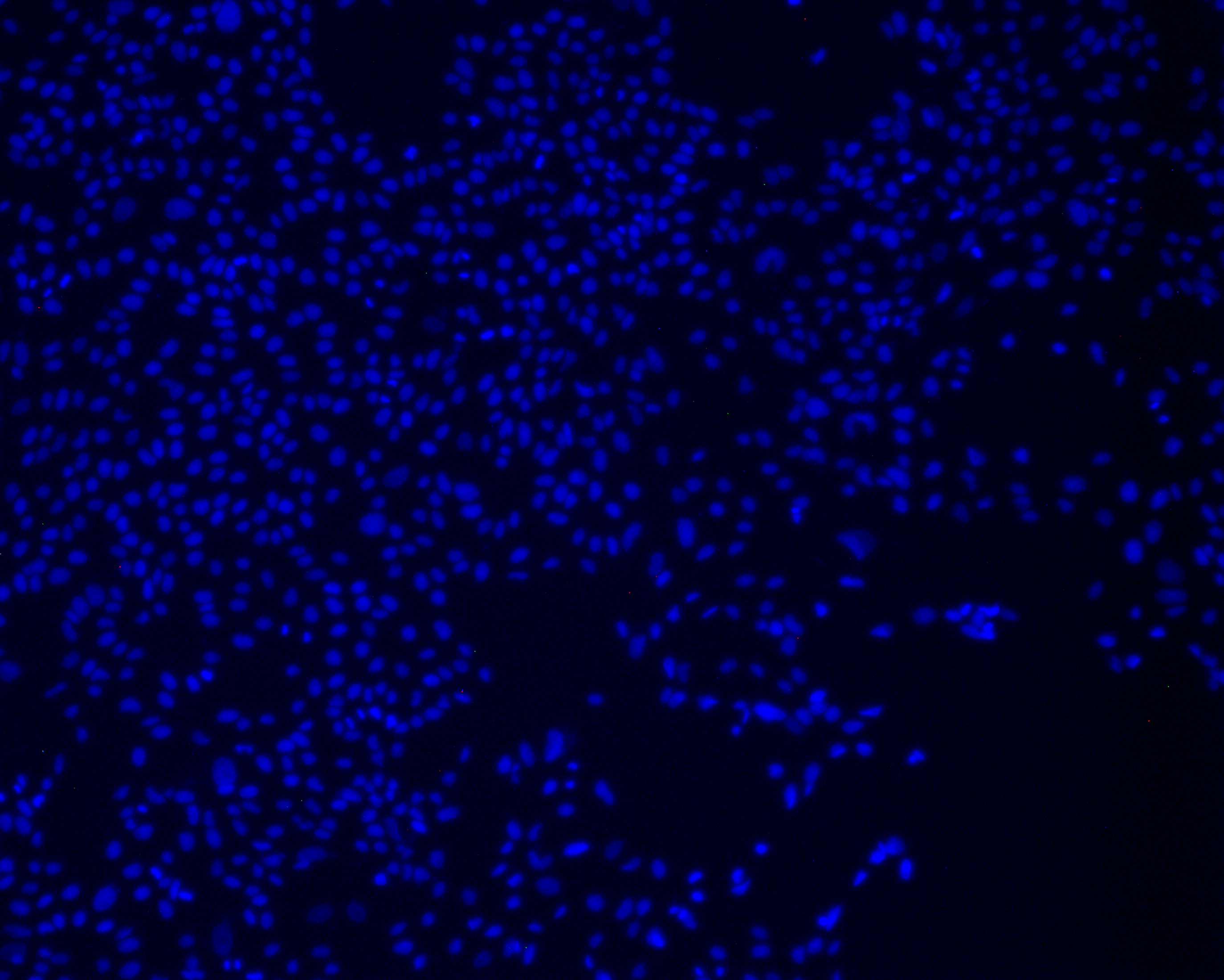

Supplement: Supplementary file 3 — Source Data for Expanded View [file EMMM-15-e17611-s013.zip › Figure EV2/EV2B/Mut DAPI.tif]

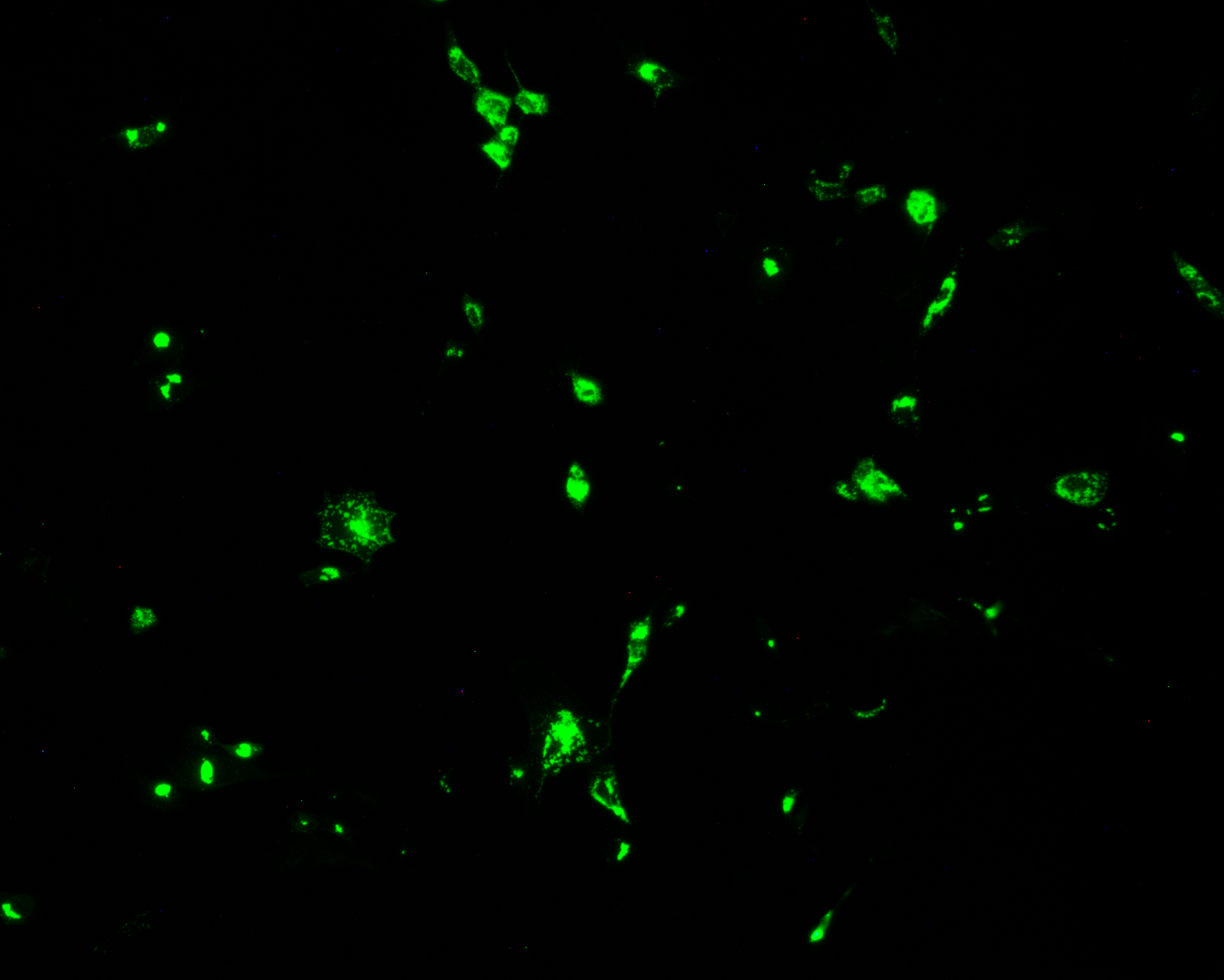

Supplement: Supplementary file 3 — Source Data for Expanded View [file EMMM-15-e17611-s013.zip › Figure EV2/EV2B/Mut EGFP.tif]

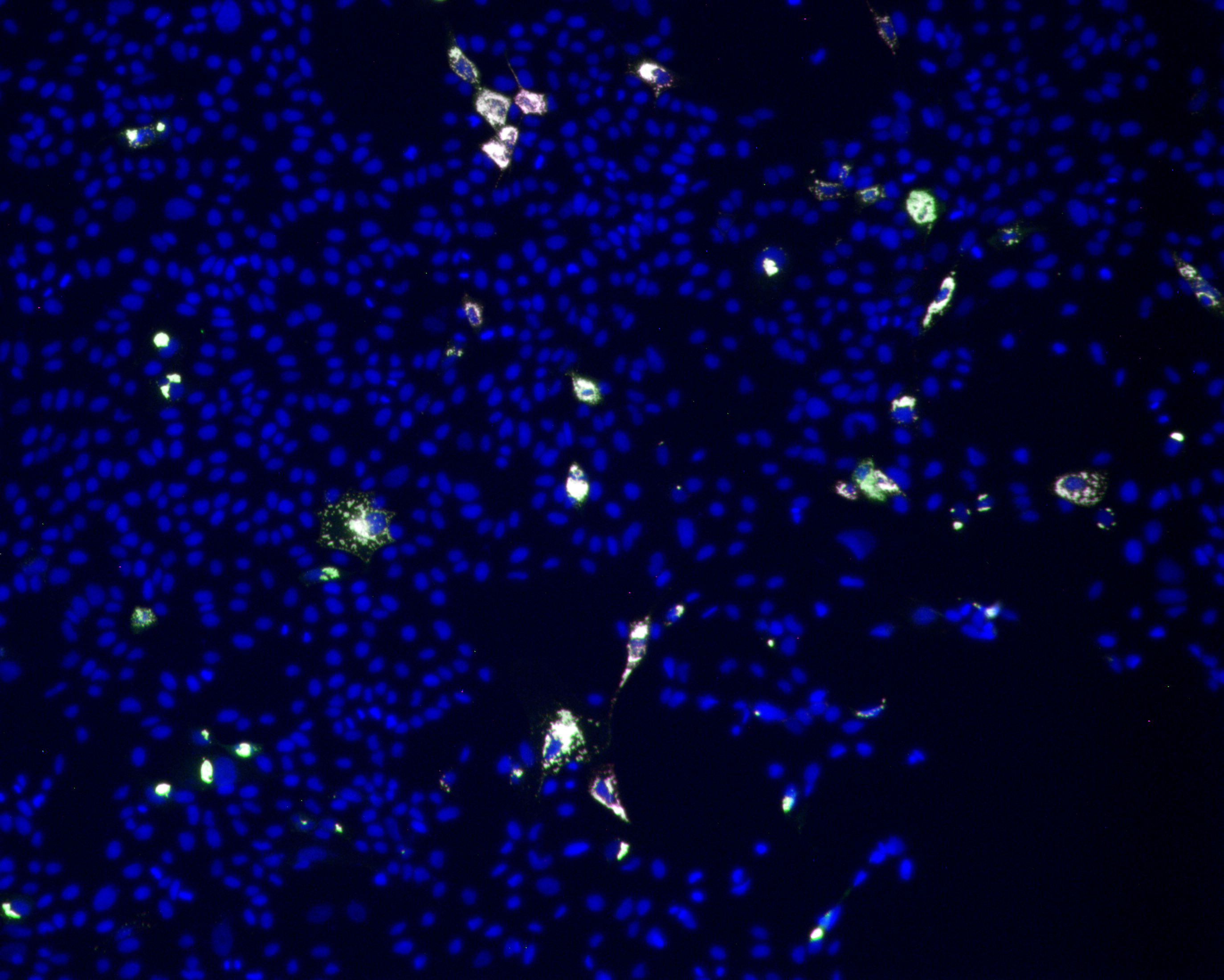

Supplement: Supplementary file 3 — Source Data for Expanded View [file EMMM-15-e17611-s013.zip › Figure EV2/EV2B/Mut Merge.tif]

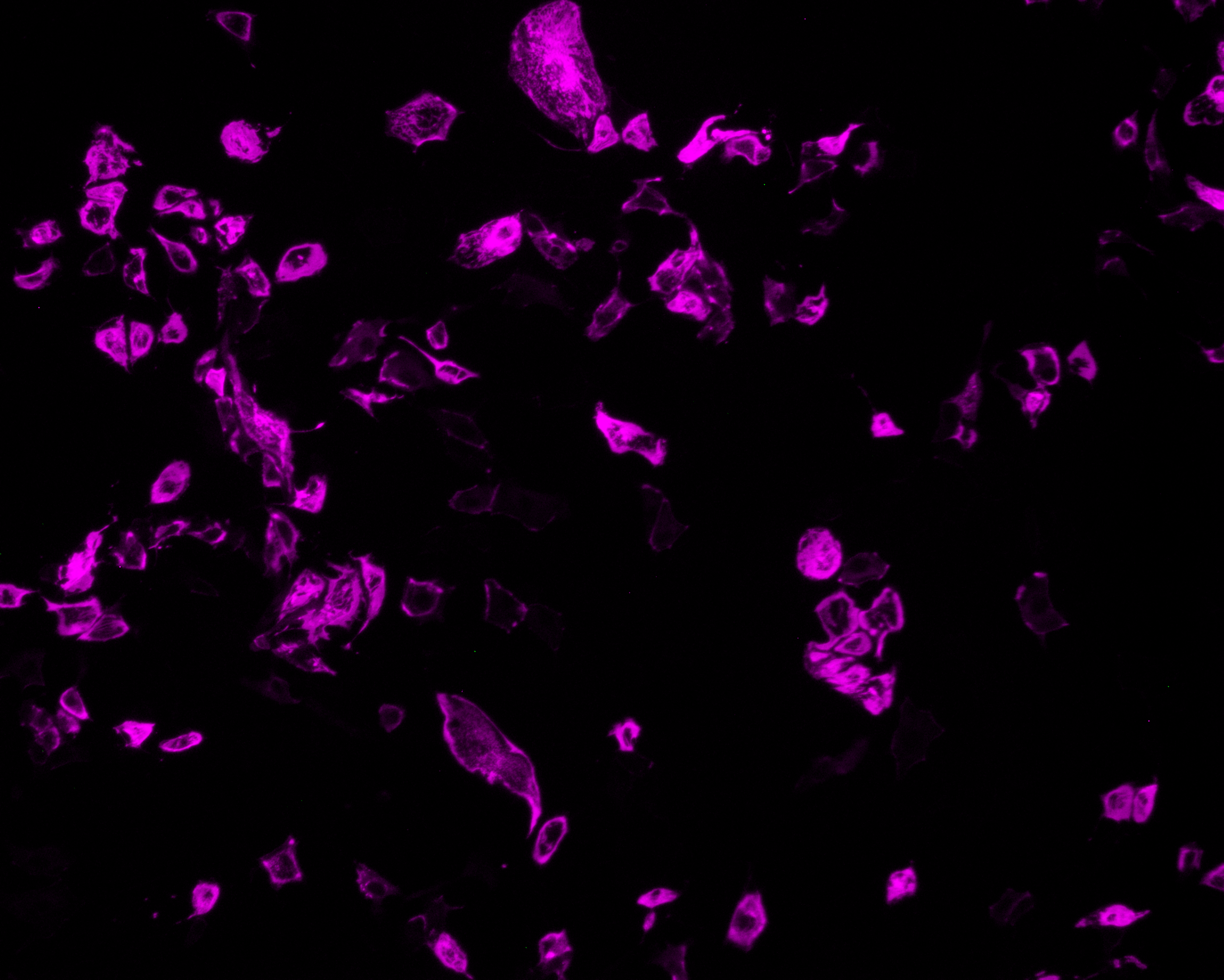

Supplement: Supplementary file 3 — Source Data for Expanded View [file EMMM-15-e17611-s013.zip › Figure EV2/EV2B/WT CGN.tif]

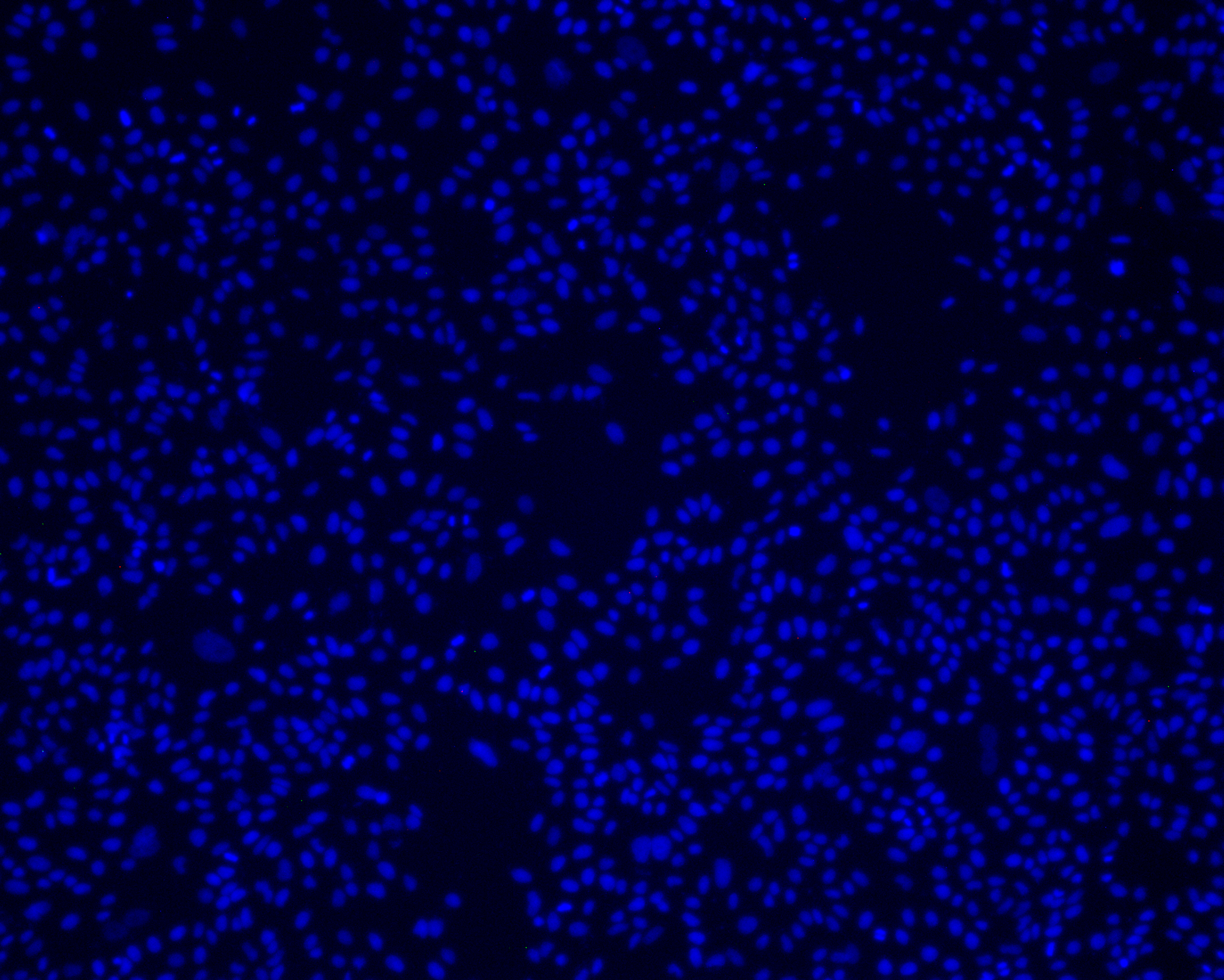

Supplement: Supplementary file 3 — Source Data for Expanded View [file EMMM-15-e17611-s013.zip › Figure EV2/EV2B/WT DAPI.tif]

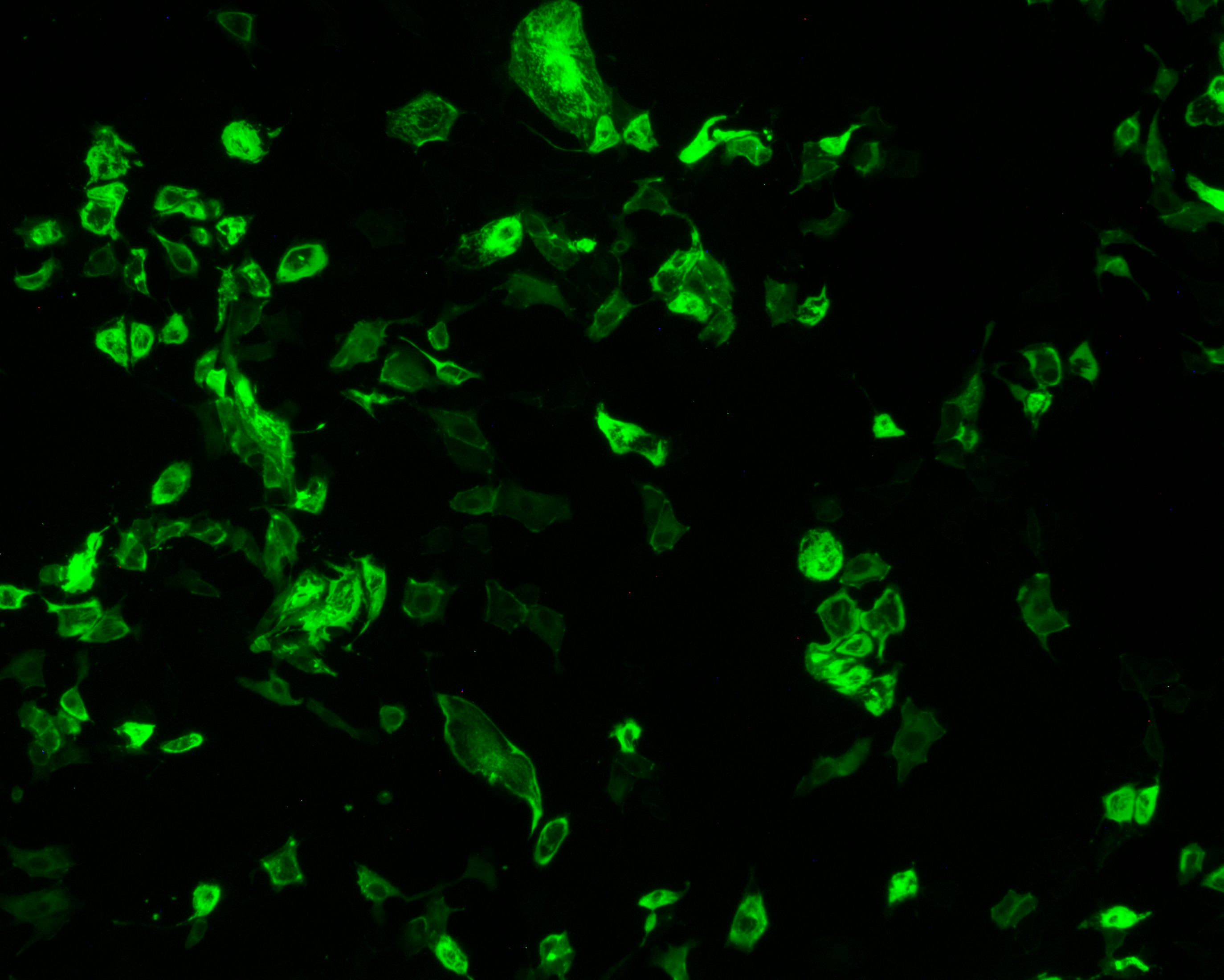

Supplement: Supplementary file 3 — Source Data for Expanded View [file EMMM-15-e17611-s013.zip › Figure EV2/EV2B/WT EGFP.tif]

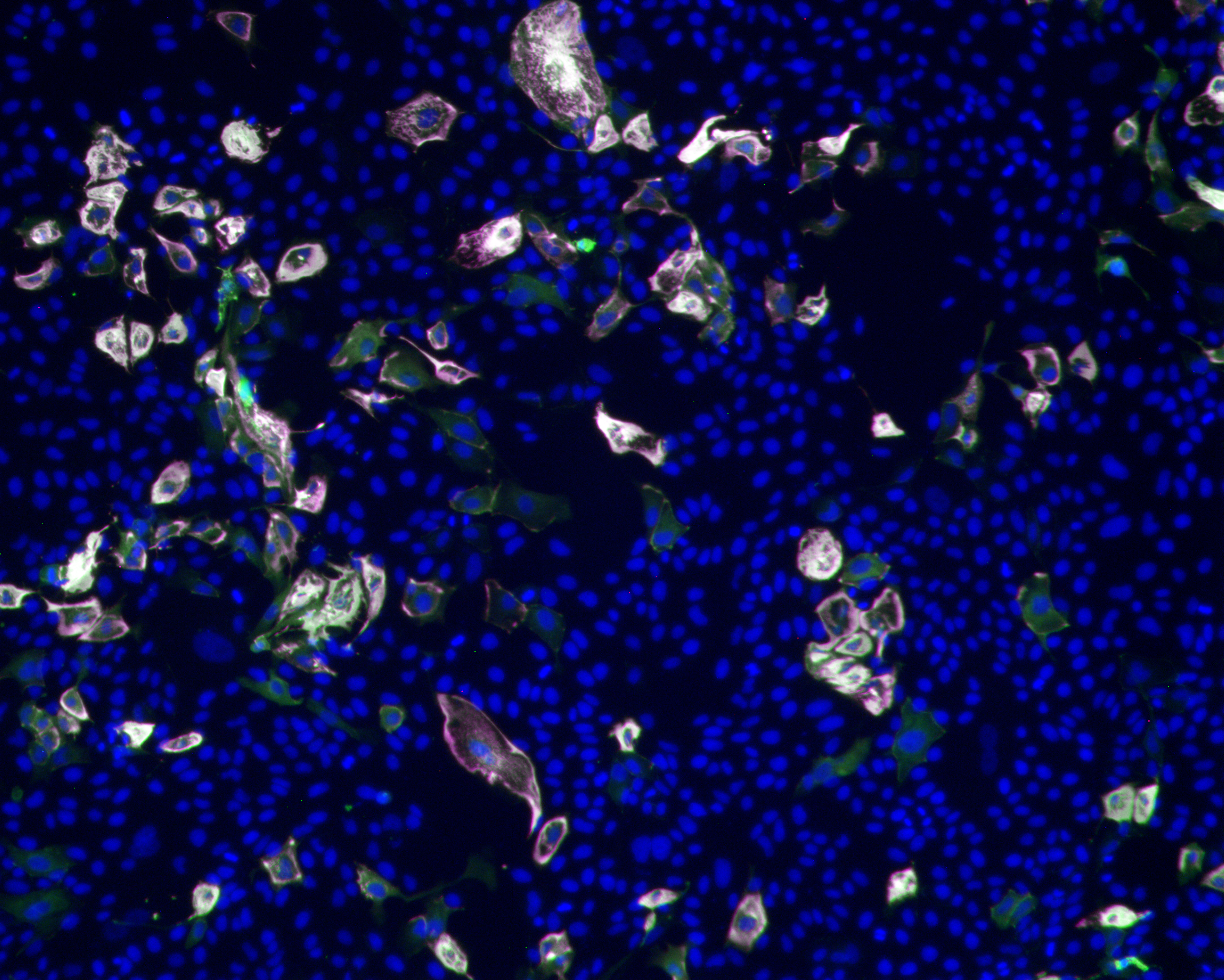

Supplement: Supplementary file 3 — Source Data for Expanded View [file EMMM-15-e17611-s013.zip › Figure EV2/EV2B/WT Merge.tif]

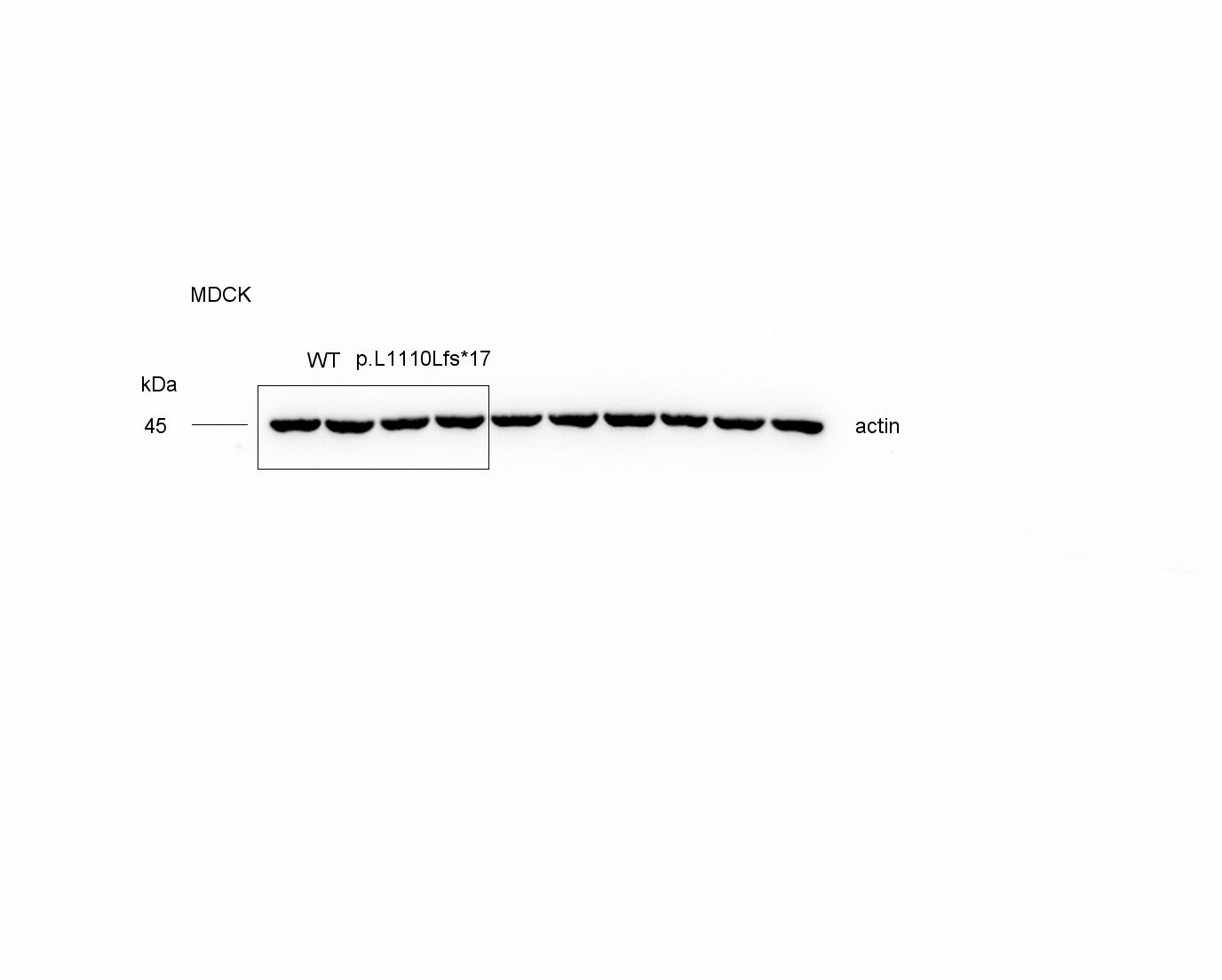

Supplement: Supplementary file 3 — Source Data for Expanded View [file EMMM-15-e17611-s013.zip › Figure EV2/EV2C/western actin.tif]

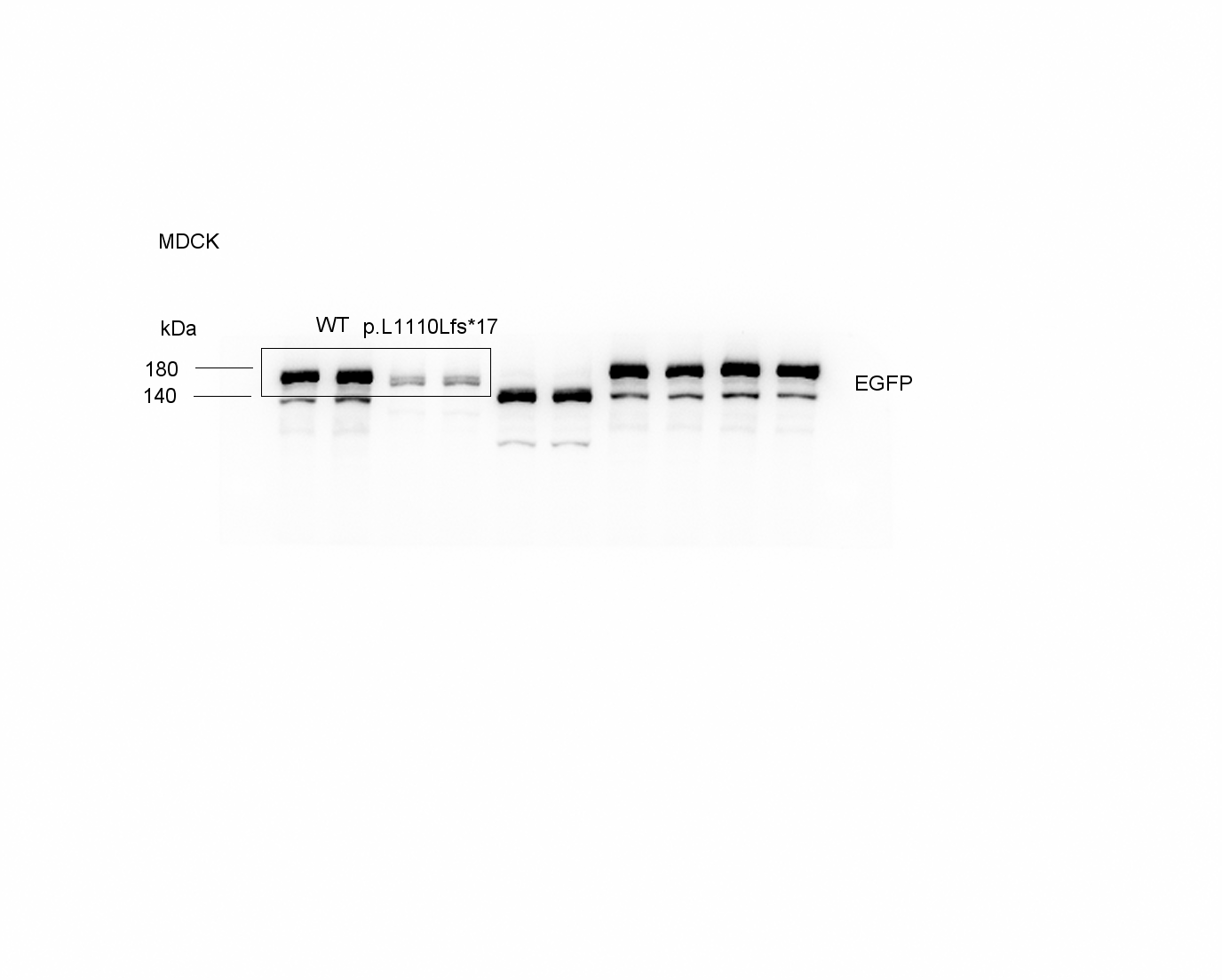

Supplement: Supplementary file 3 — Source Data for Expanded View [file EMMM-15-e17611-s013.zip › Figure EV2/EV2C/western EGFP.tif]

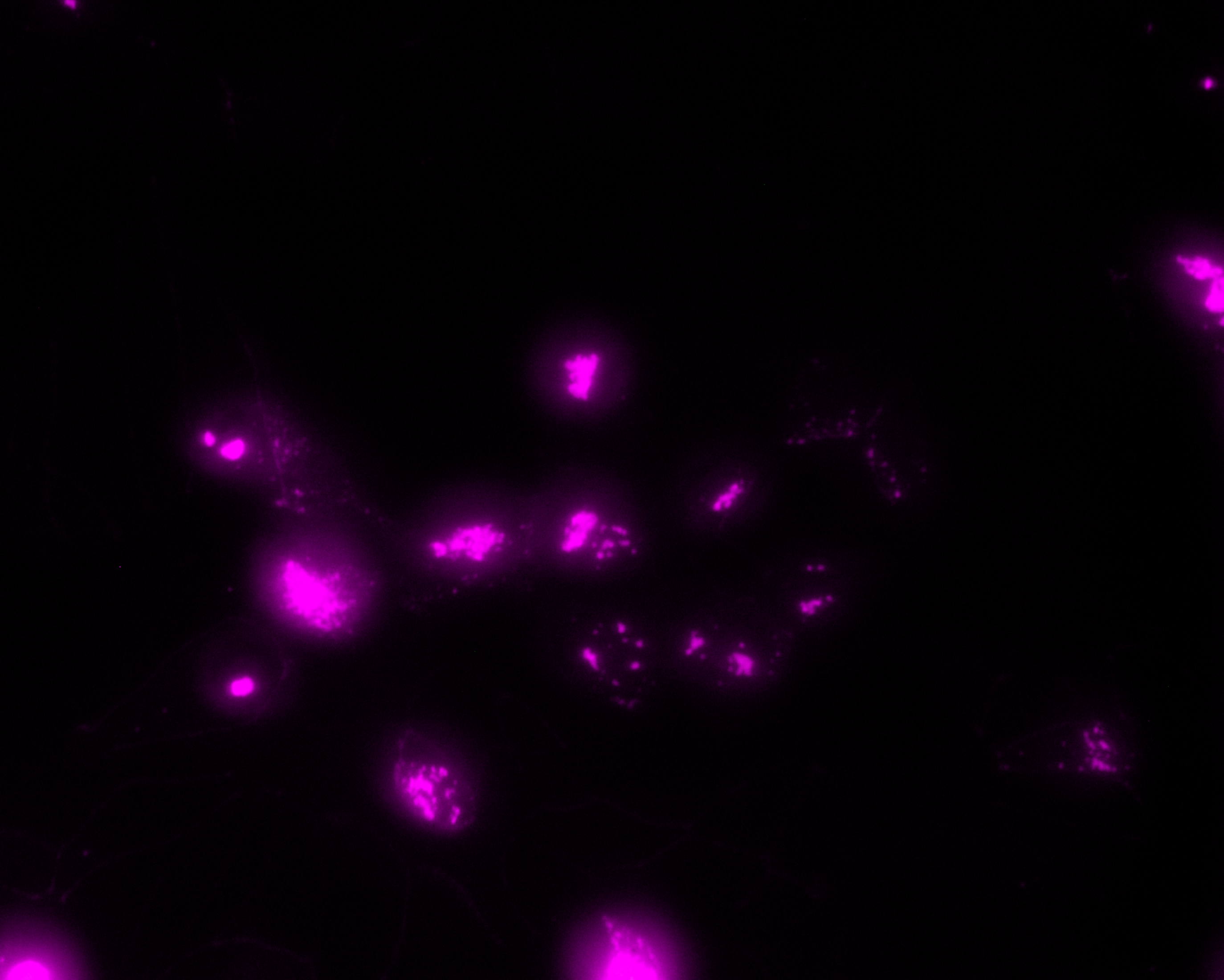

Supplement: Supplementary file 3 — Source Data for Expanded View [file EMMM-15-e17611-s013.zip › Figure EV2/EV2E/Mut CGN.tif]

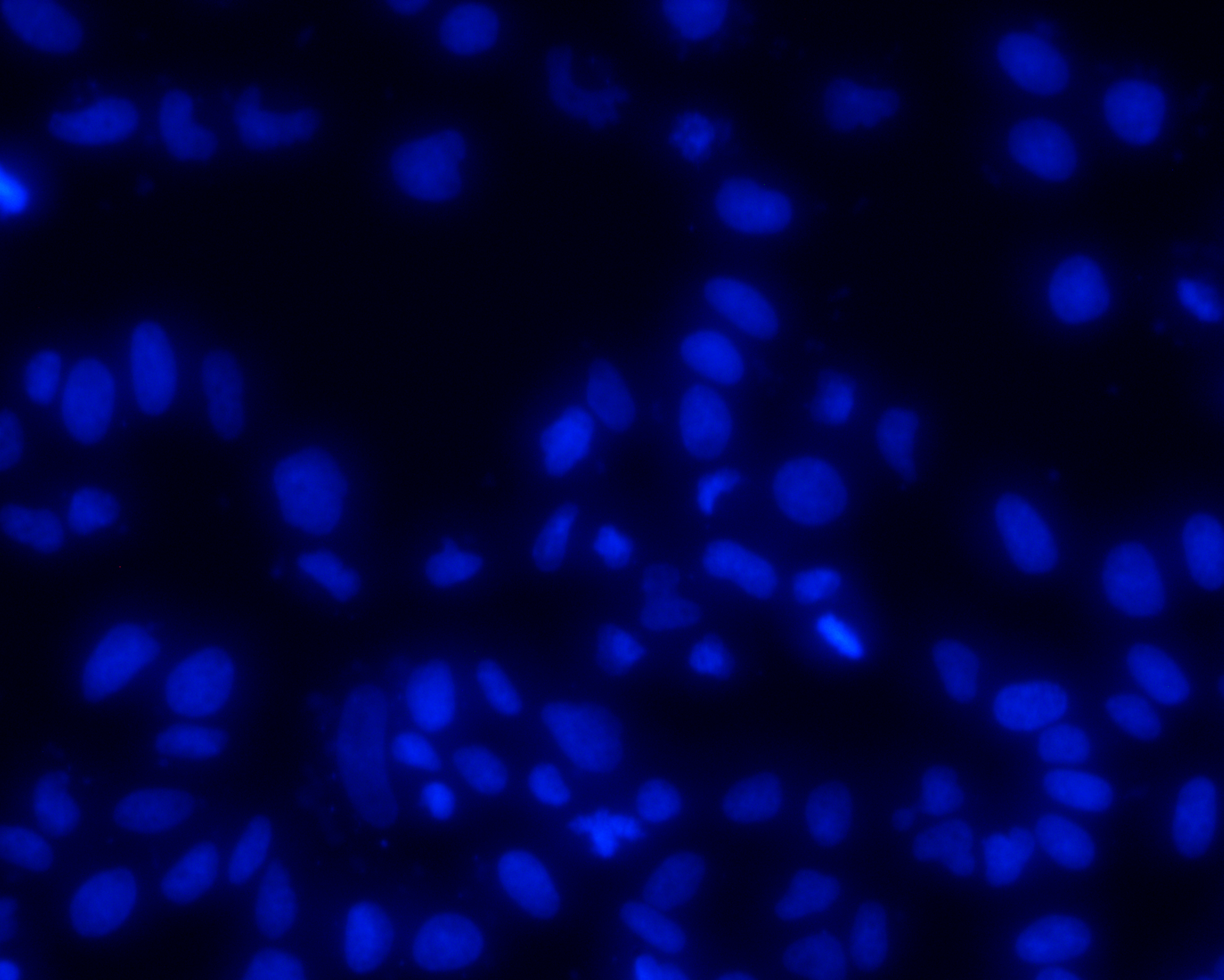

Supplement: Supplementary file 3 — Source Data for Expanded View [file EMMM-15-e17611-s013.zip › Figure EV2/EV2E/Mut DAPI.tif]

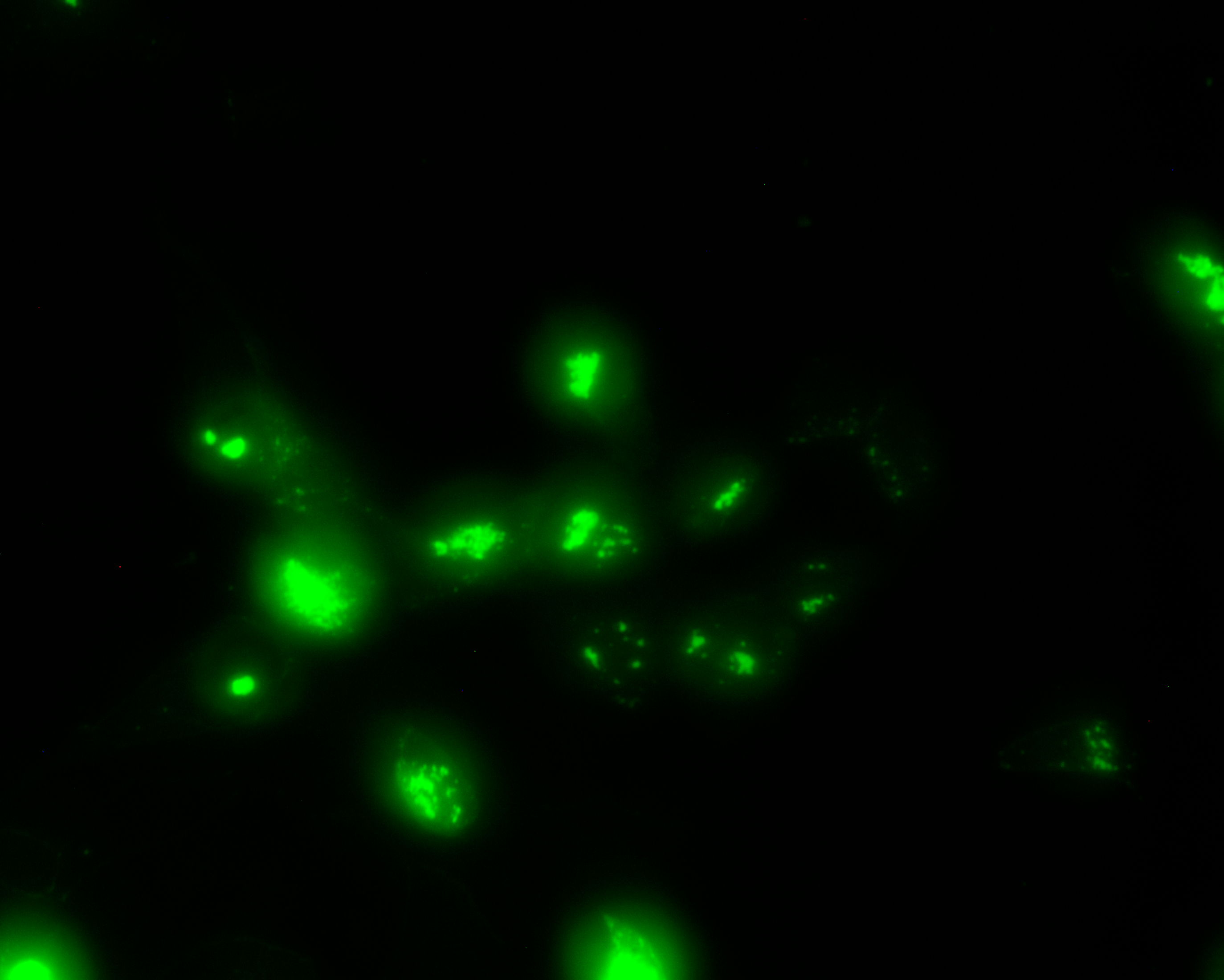

Supplement: Supplementary file 3 — Source Data for Expanded View [file EMMM-15-e17611-s013.zip › Figure EV2/EV2E/Mut EGFP.tif]

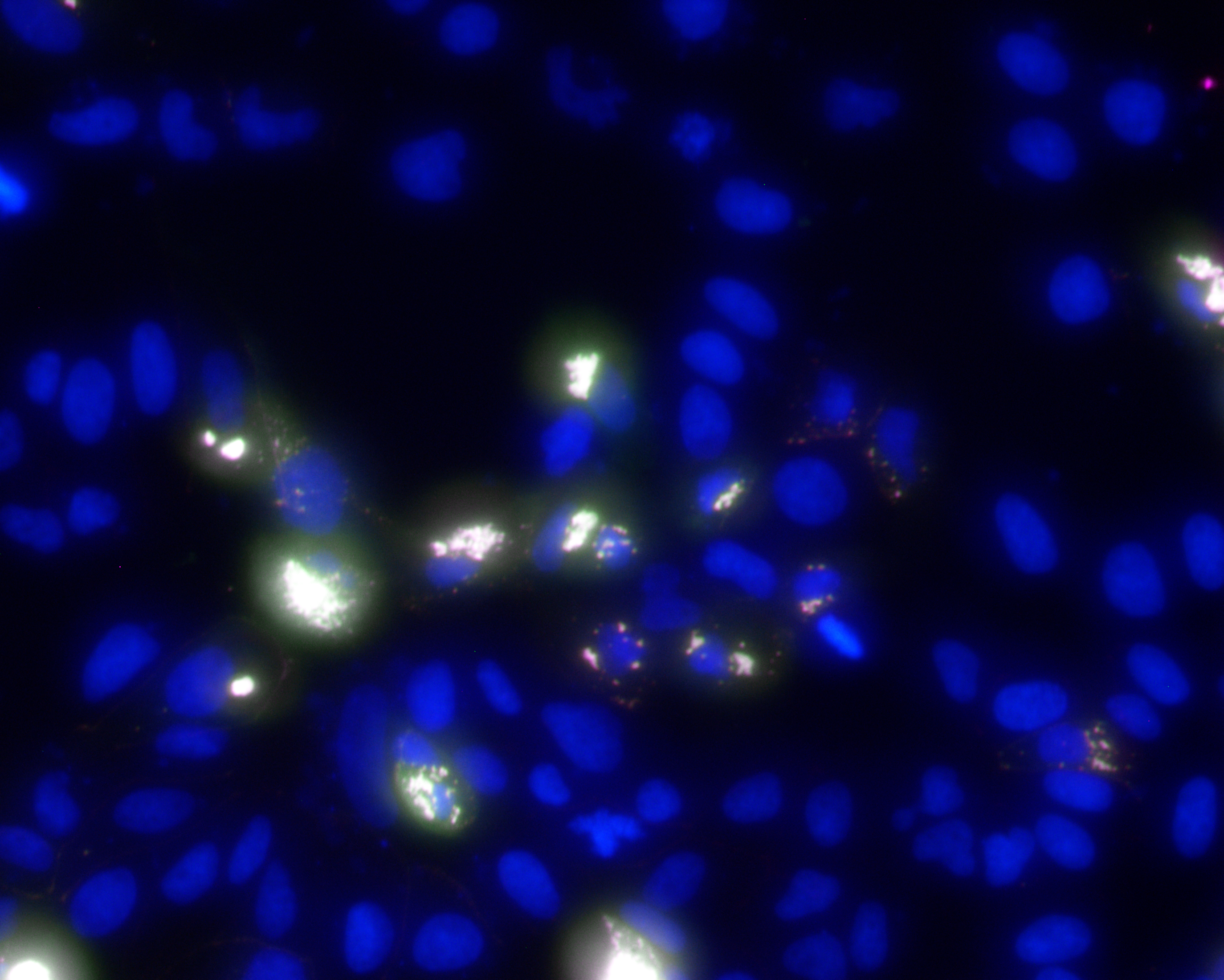

Supplement: Supplementary file 3 — Source Data for Expanded View [file EMMM-15-e17611-s013.zip › Figure EV2/EV2E/Mut Merge.tif]

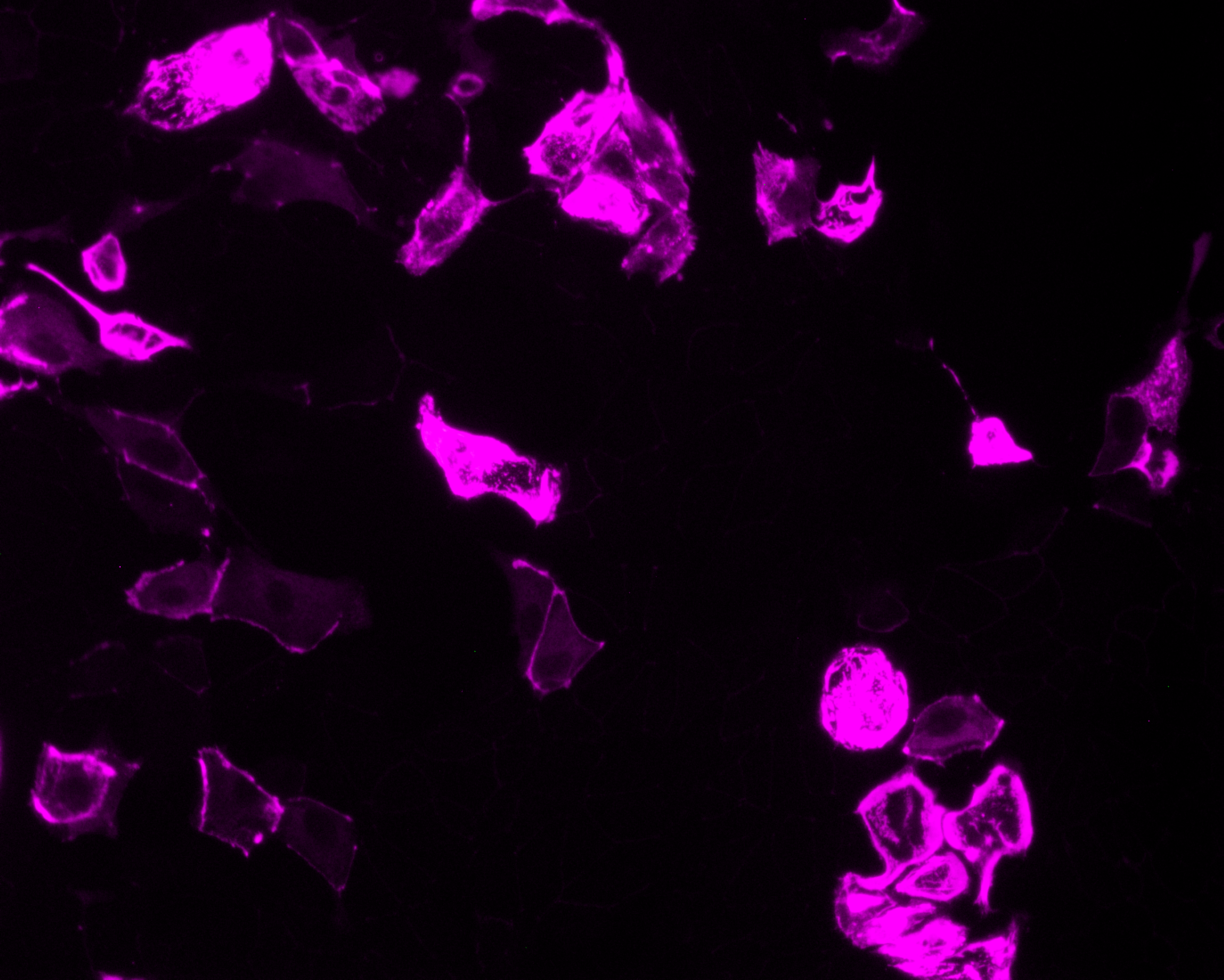

Supplement: Supplementary file 3 — Source Data for Expanded View [file EMMM-15-e17611-s013.zip › Figure EV2/EV2E/WT CGN.tif]

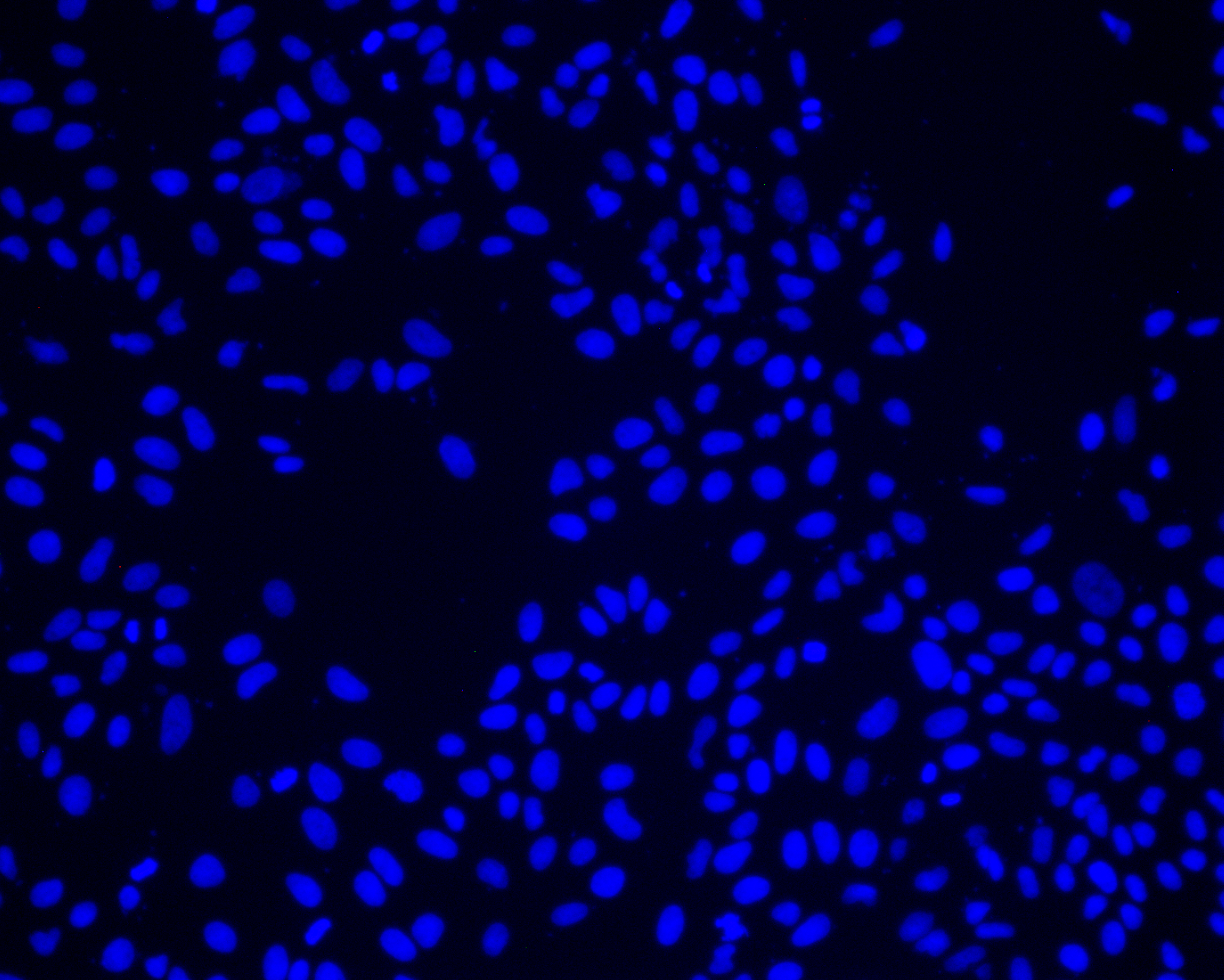

Supplement: Supplementary file 3 — Source Data for Expanded View [file EMMM-15-e17611-s013.zip › Figure EV2/EV2E/WT DAPI.tif]

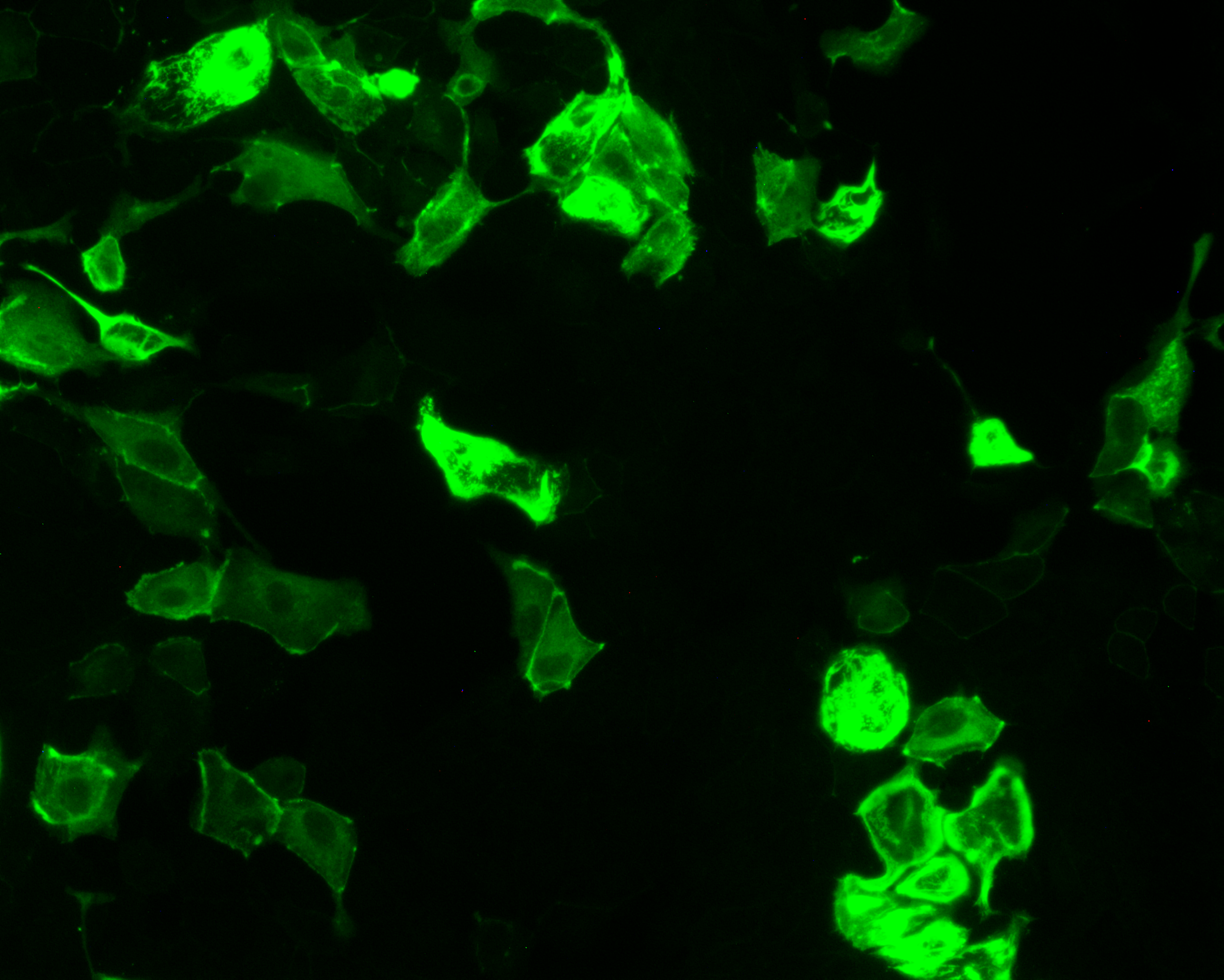

Supplement: Supplementary file 3 — Source Data for Expanded View [file EMMM-15-e17611-s013.zip › Figure EV2/EV2E/WT EGFP.tif]

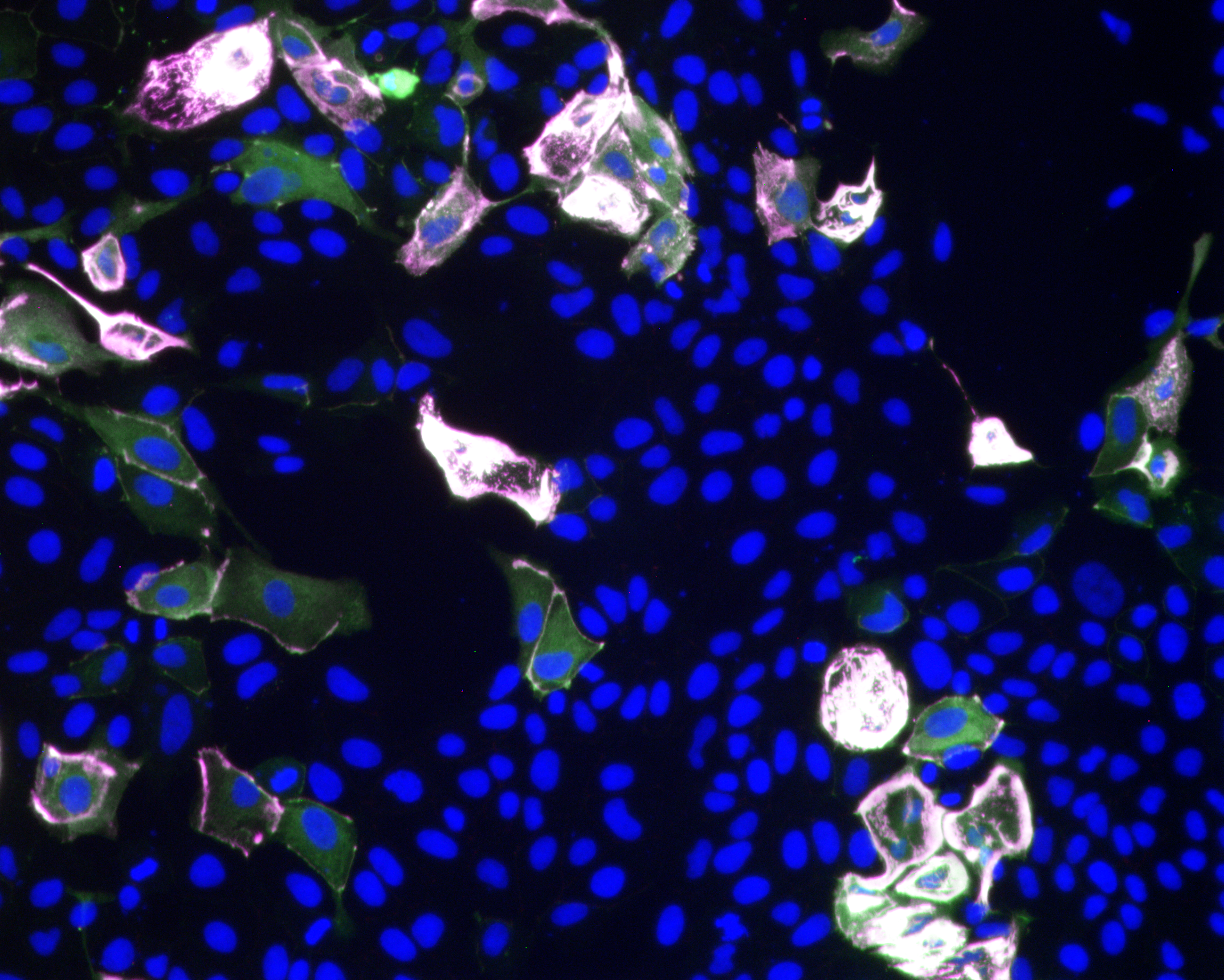

Supplement: Supplementary file 3 — Source Data for Expanded View [file EMMM-15-e17611-s013.zip › Figure EV2/EV2E/WT Merge.tif]

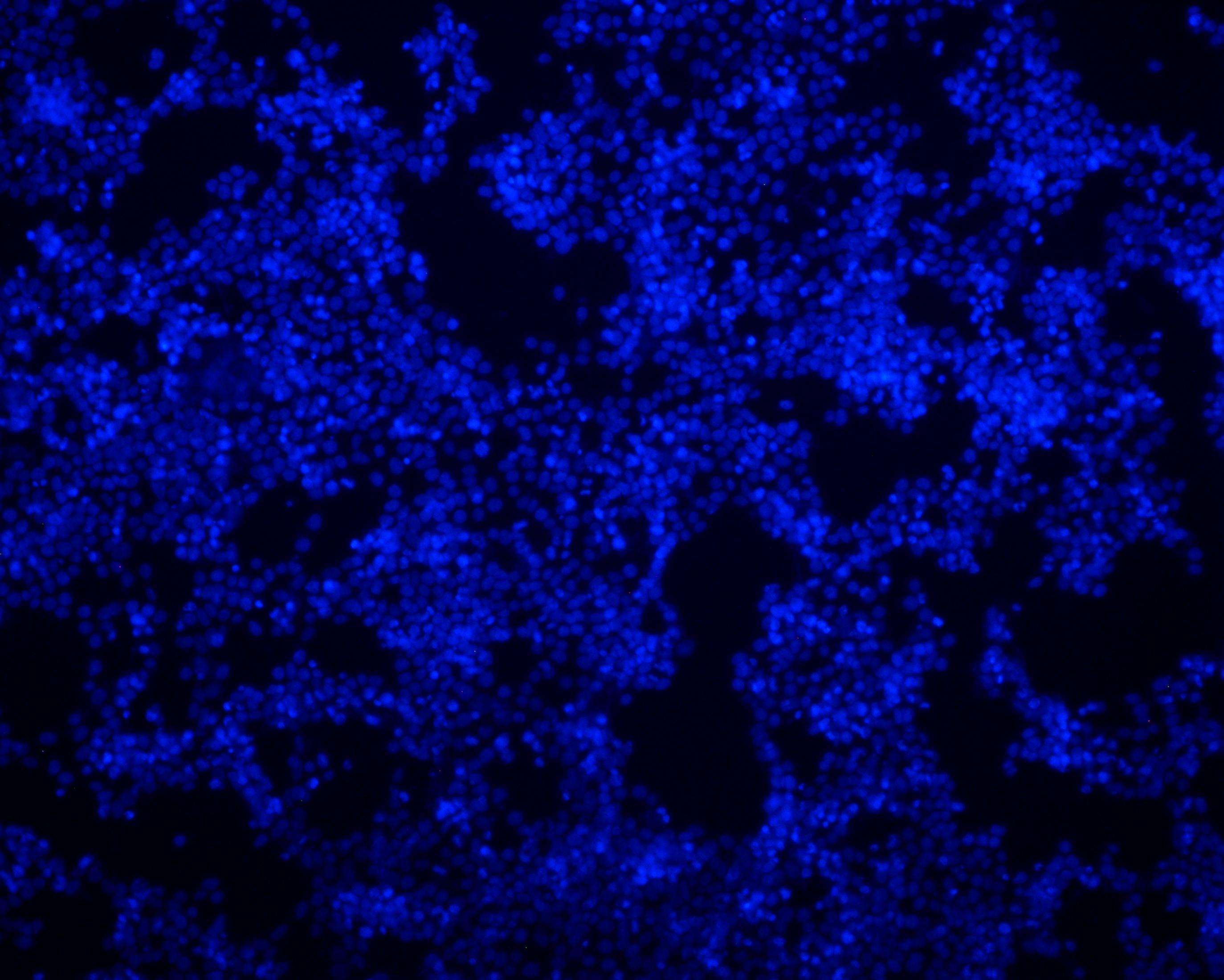

Supplement: Supplementary file 3 — Source Data for Expanded View [file EMMM-15-e17611-s013.zip › Figure EV2/EV2F/10x Mut DAPI.tif]

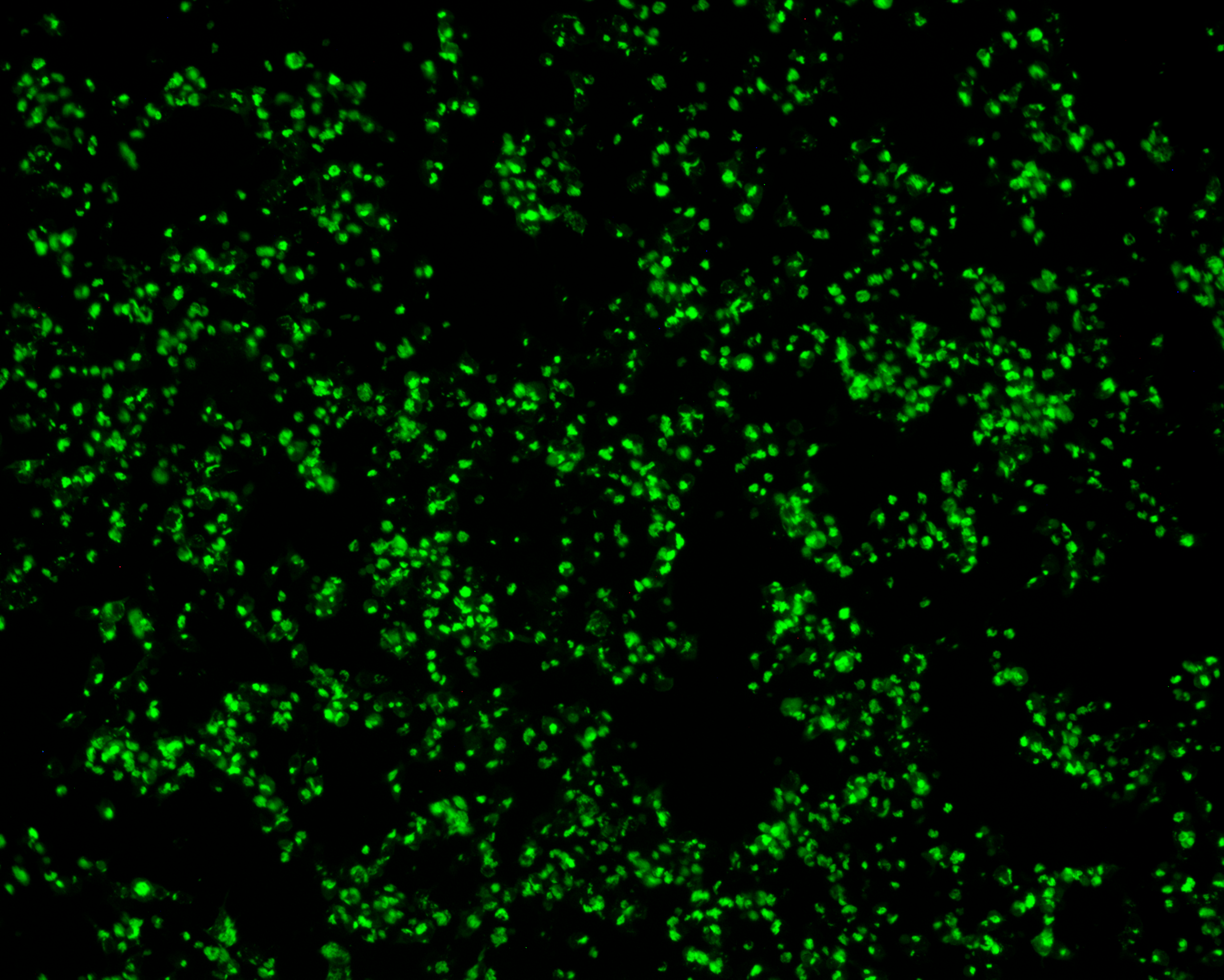

Supplement: Supplementary file 3 — Source Data for Expanded View [file EMMM-15-e17611-s013.zip › Figure EV2/EV2F/10x Mut EGFP.tif]

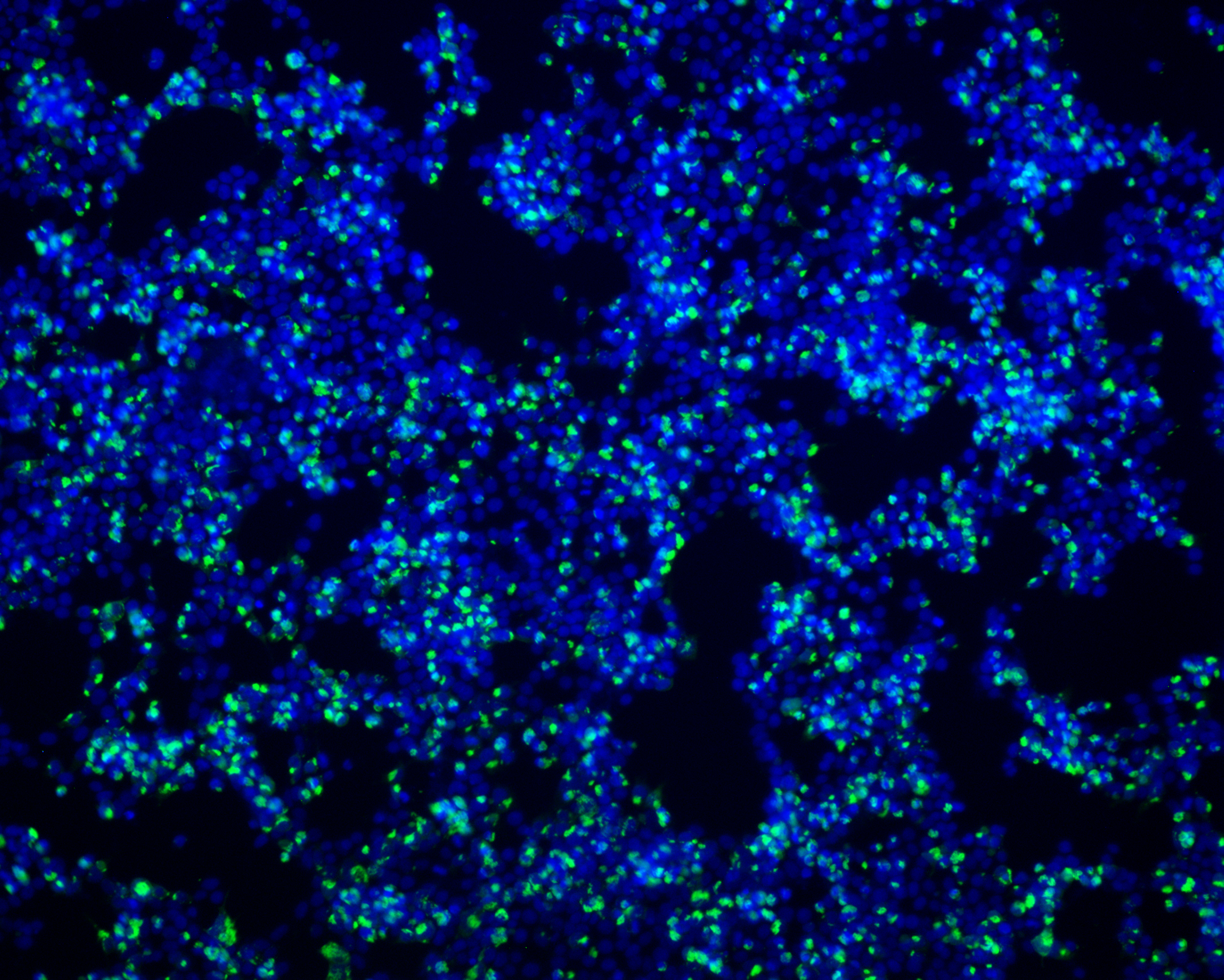

Supplement: Supplementary file 3 — Source Data for Expanded View [file EMMM-15-e17611-s013.zip › Figure EV2/EV2F/10x Mut Merge.tif]

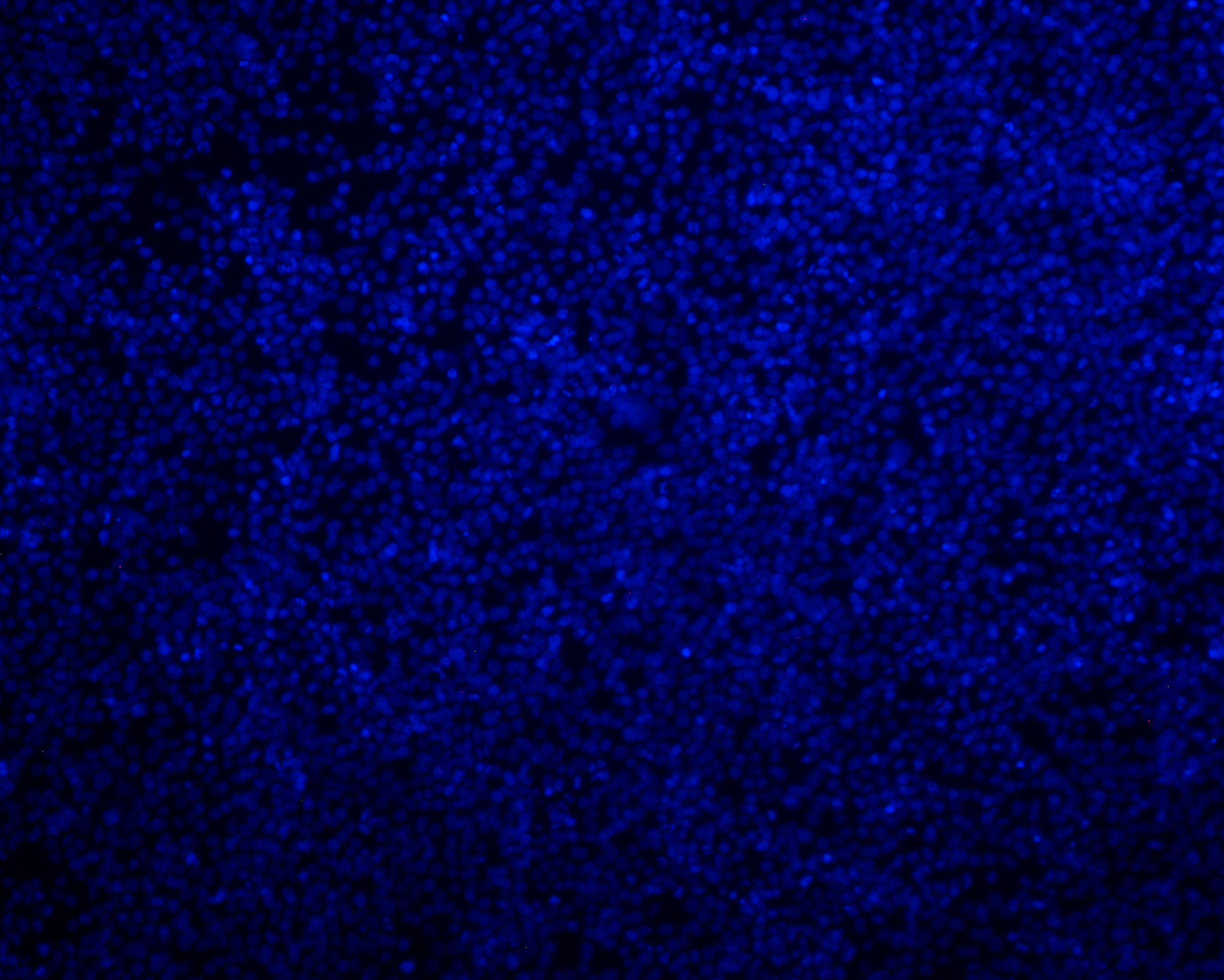

Supplement: Supplementary file 3 — Source Data for Expanded View [file EMMM-15-e17611-s013.zip › Figure EV2/EV2F/10x WT DAPI.tif]

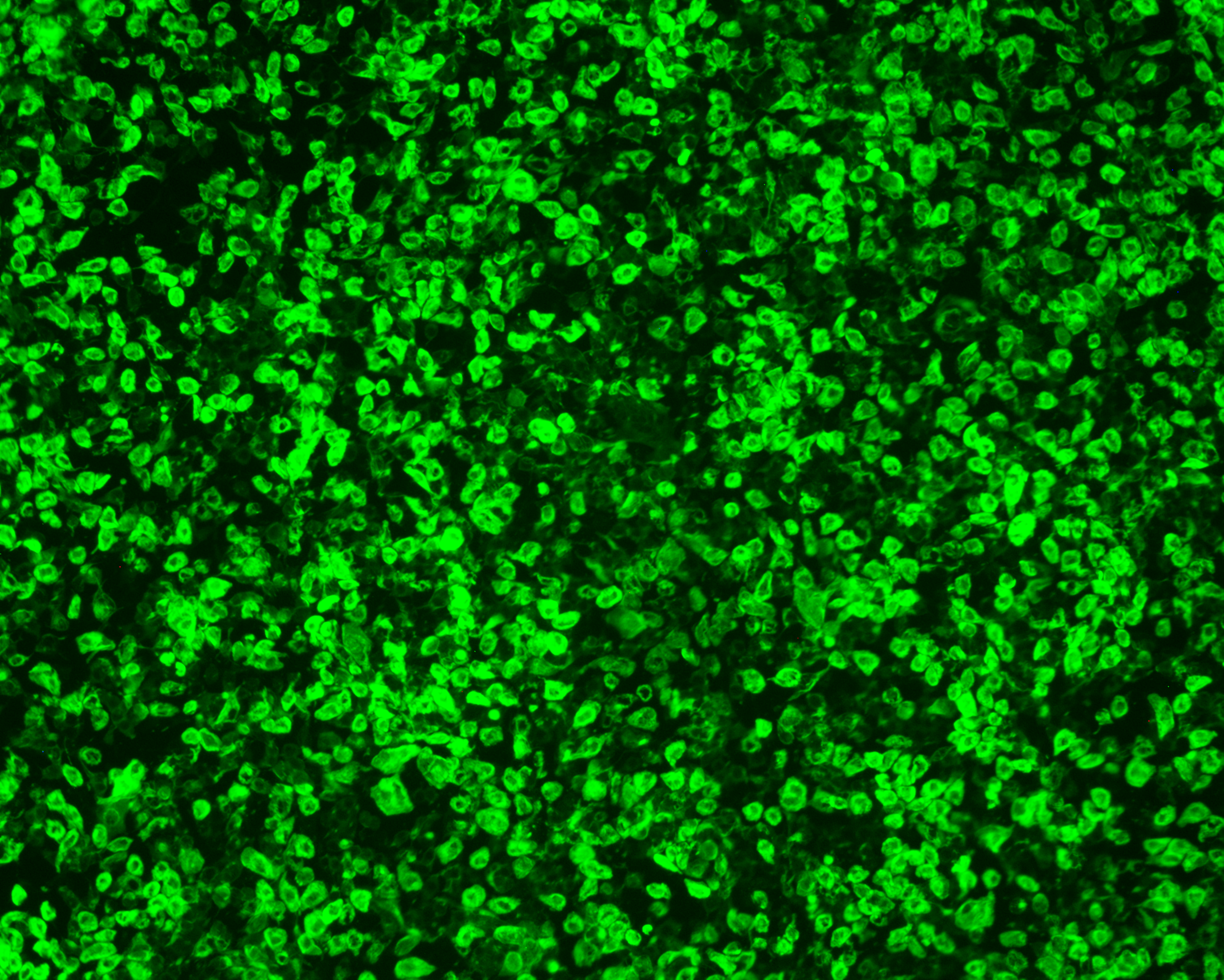

Supplement: Supplementary file 3 — Source Data for Expanded View [file EMMM-15-e17611-s013.zip › Figure EV2/EV2F/10x WT EGFP.tif]

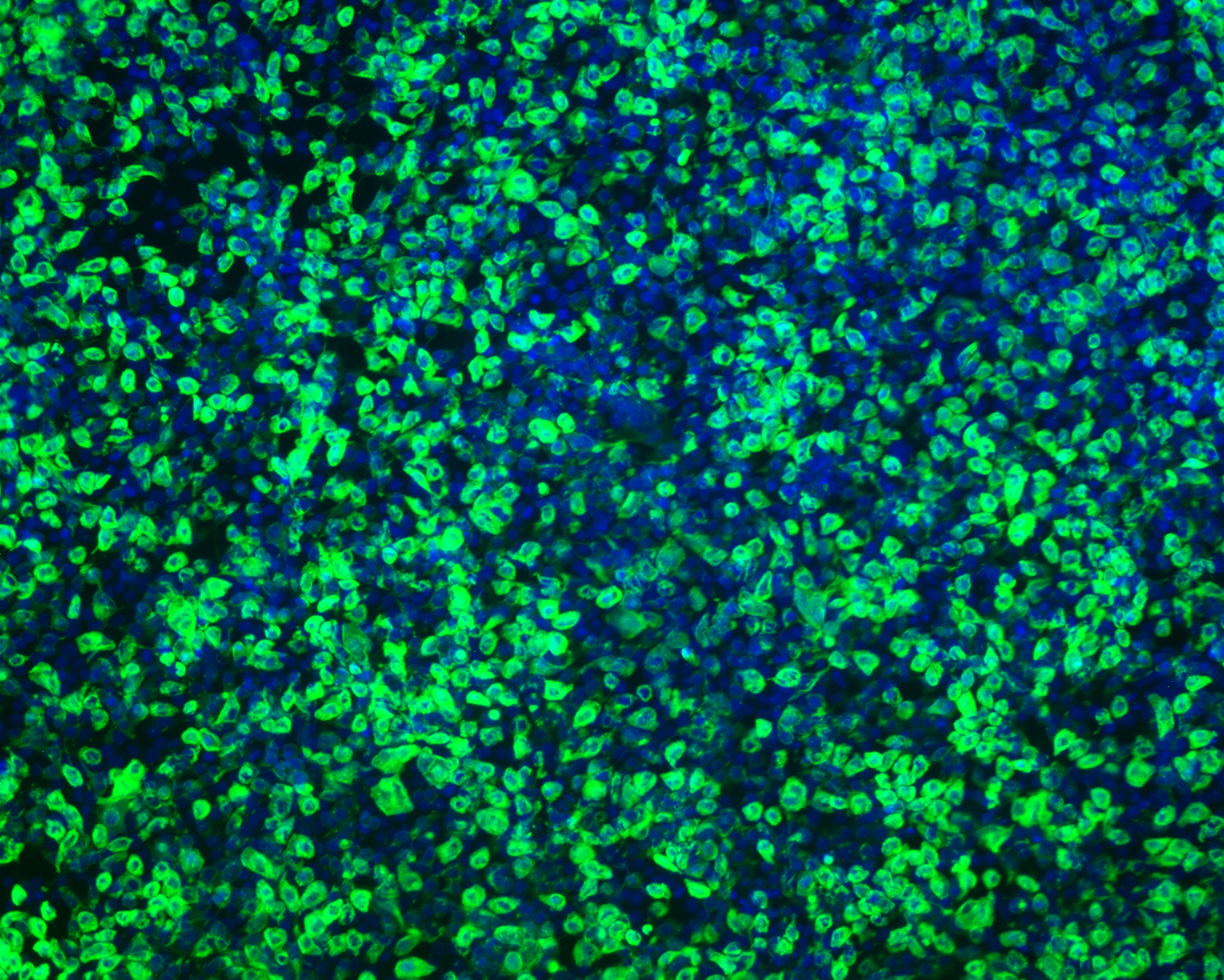

Supplement: Supplementary file 3 — Source Data for Expanded View [file EMMM-15-e17611-s013.zip › Figure EV2/EV2F/10x WT Merge.tif]

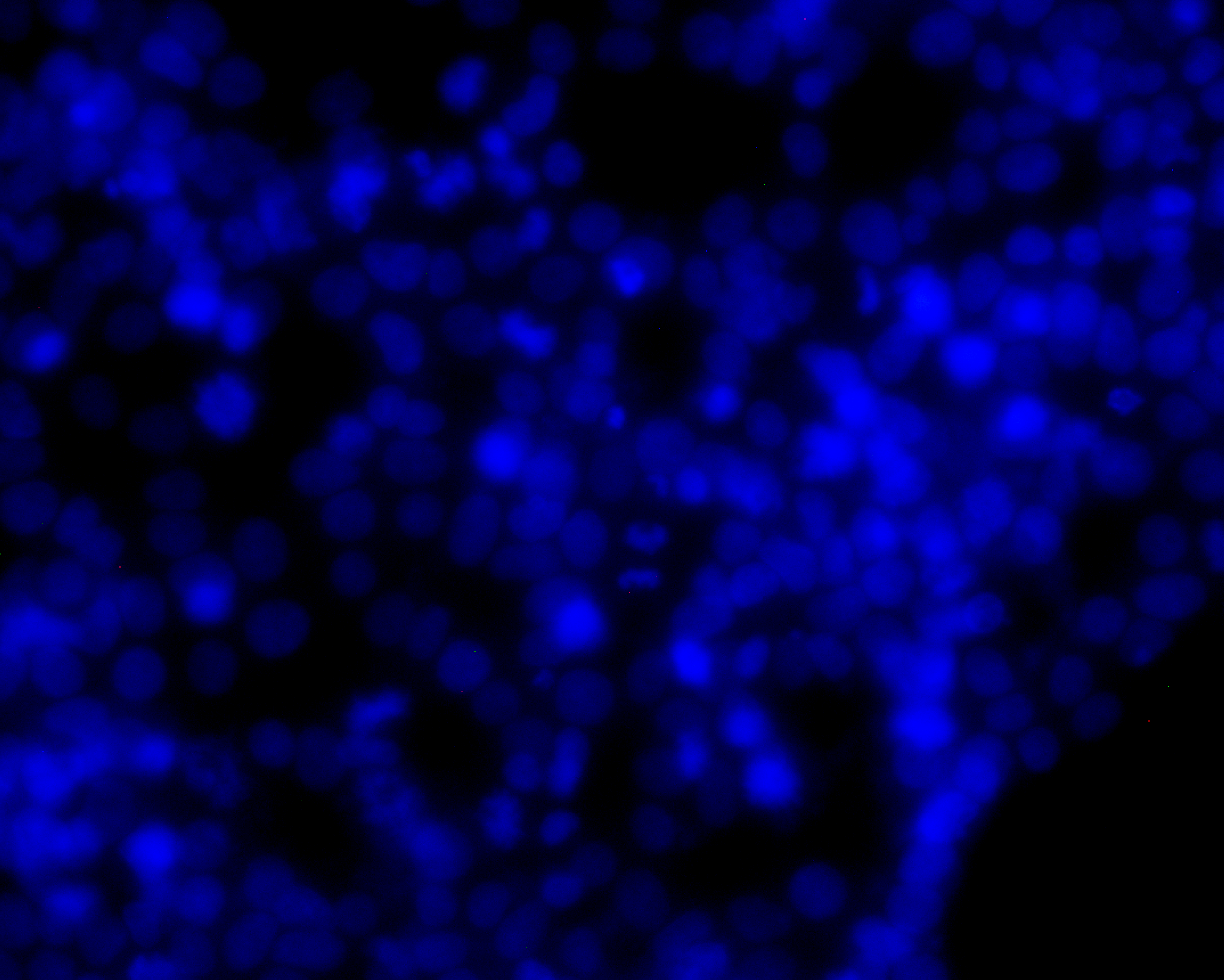

Supplement: Supplementary file 3 — Source Data for Expanded View [file EMMM-15-e17611-s013.zip › Figure EV2/EV2F/40x Mut DAPI.tif]

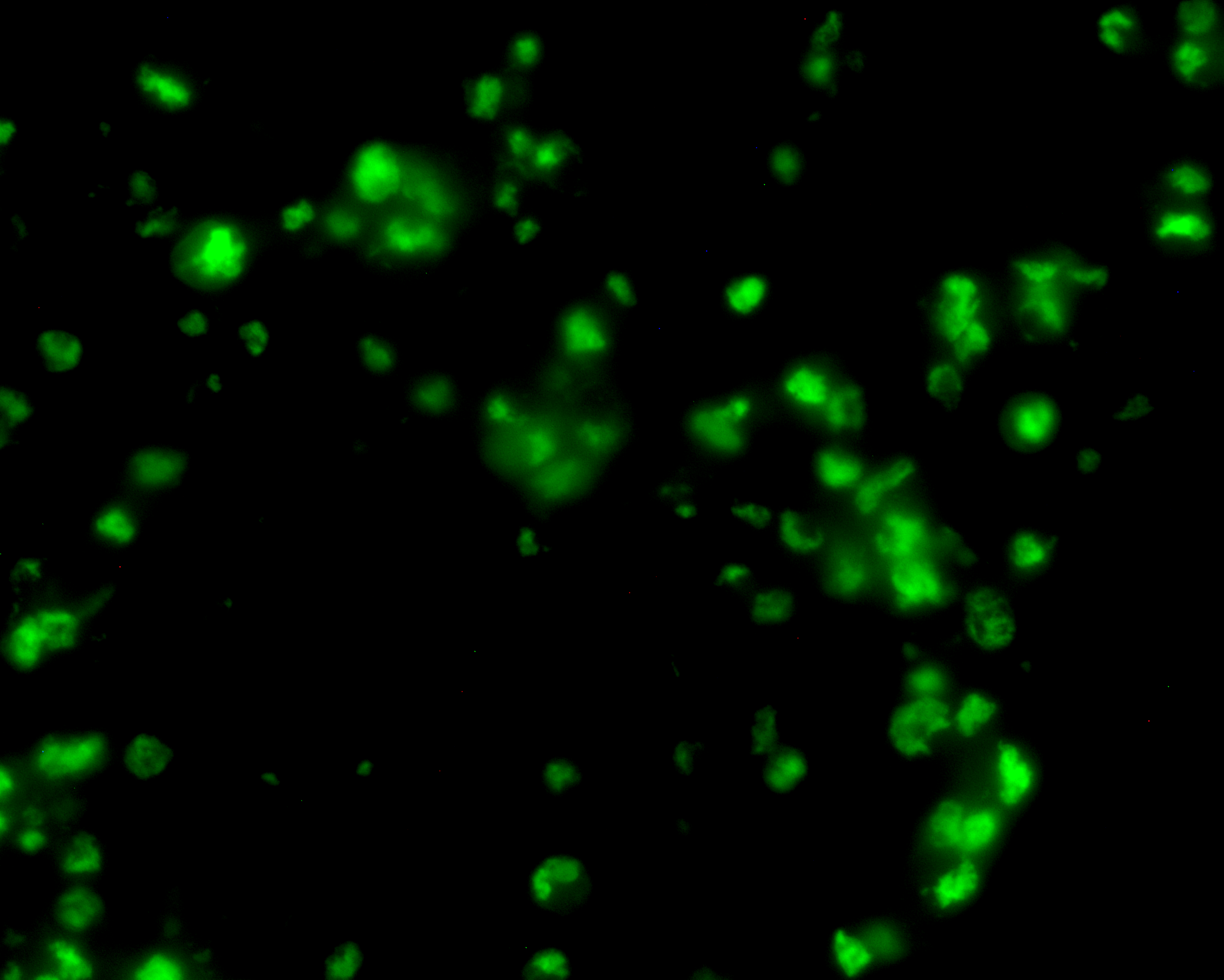

Supplement: Supplementary file 3 — Source Data for Expanded View [file EMMM-15-e17611-s013.zip › Figure EV2/EV2F/40x Mut EGFP.tif]

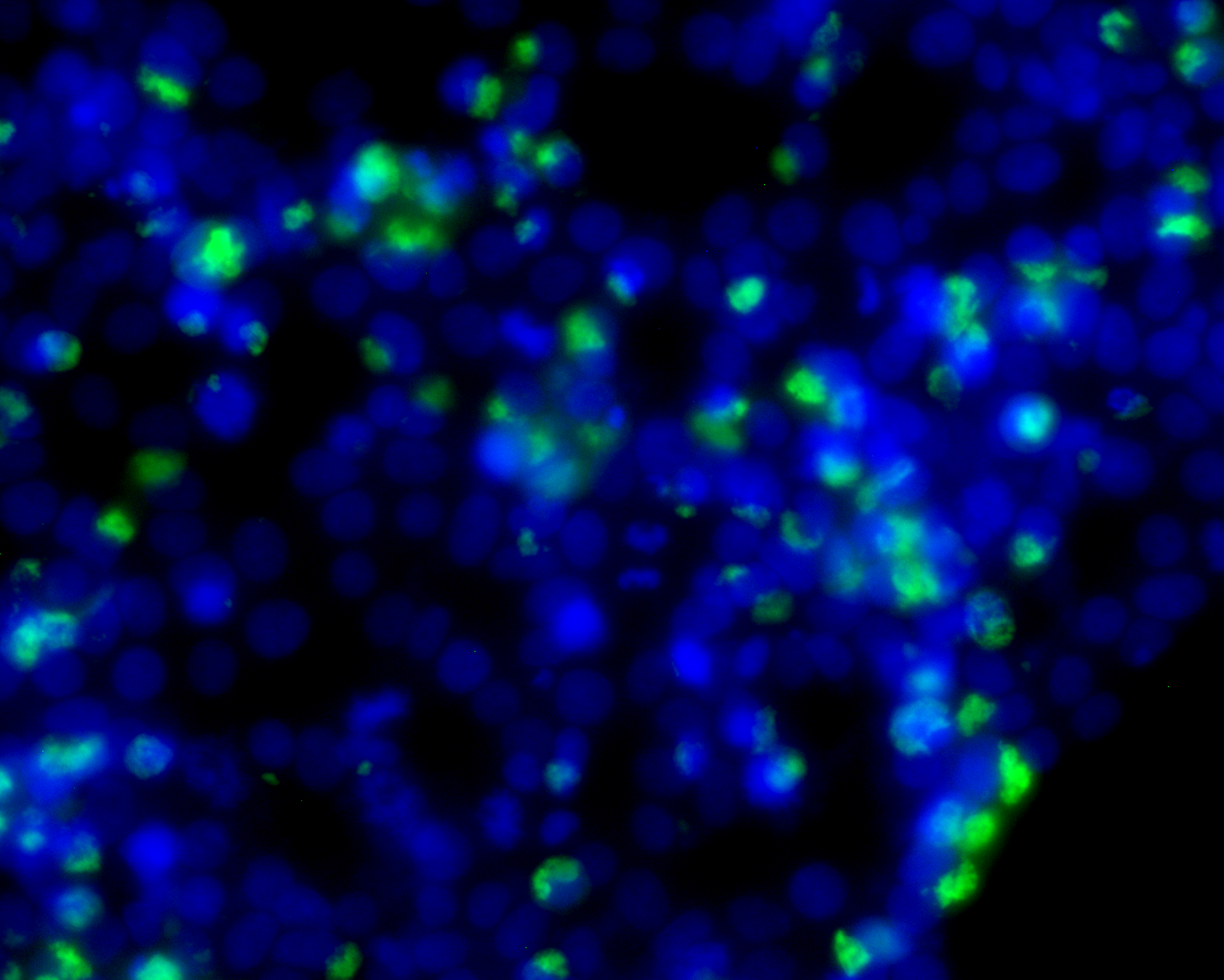

Supplement: Supplementary file 3 — Source Data for Expanded View [file EMMM-15-e17611-s013.zip › Figure EV2/EV2F/40x Mut Merge.tif]

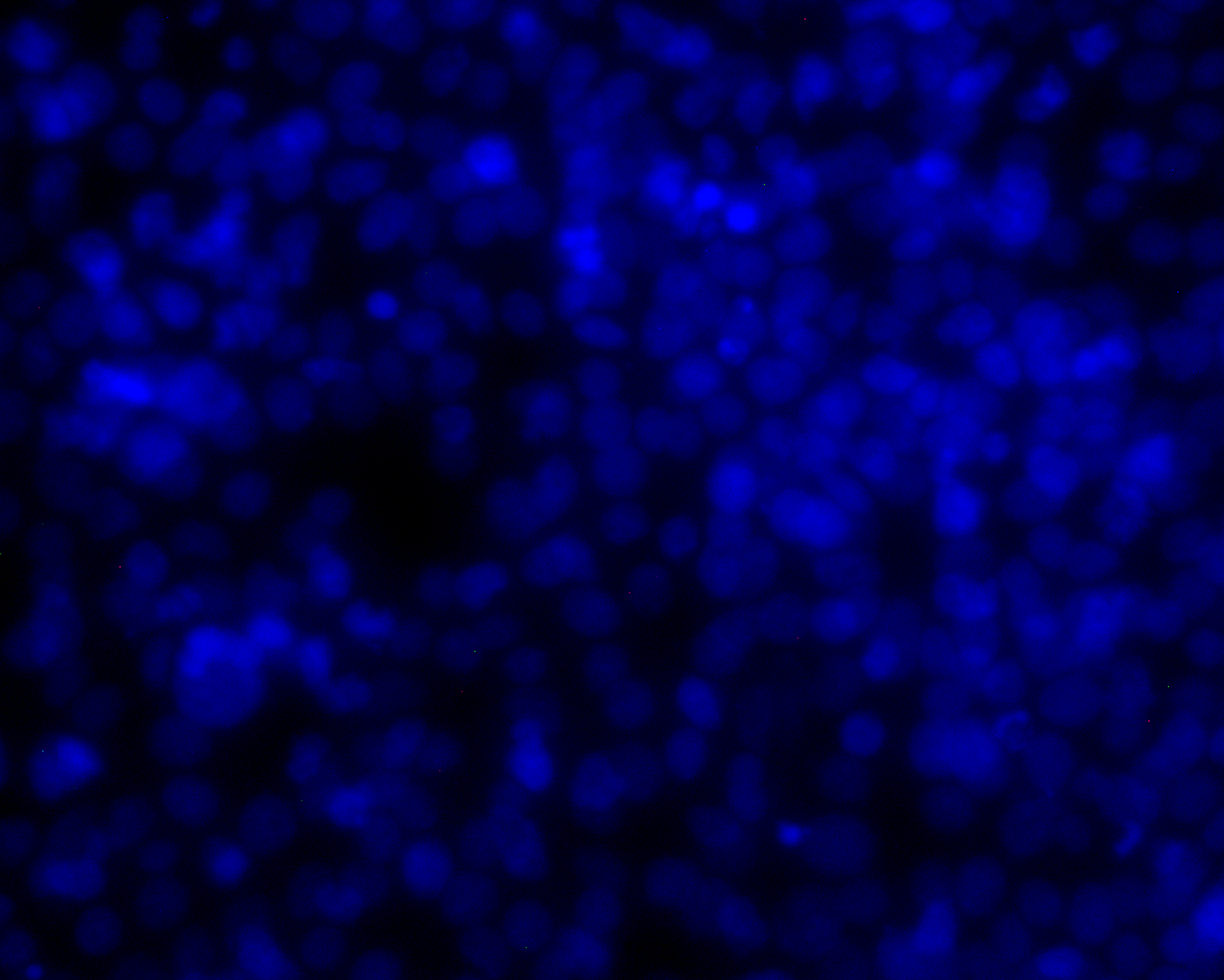

Supplement: Supplementary file 3 — Source Data for Expanded View [file EMMM-15-e17611-s013.zip › Figure EV2/EV2F/40x WT DAPI.tif]

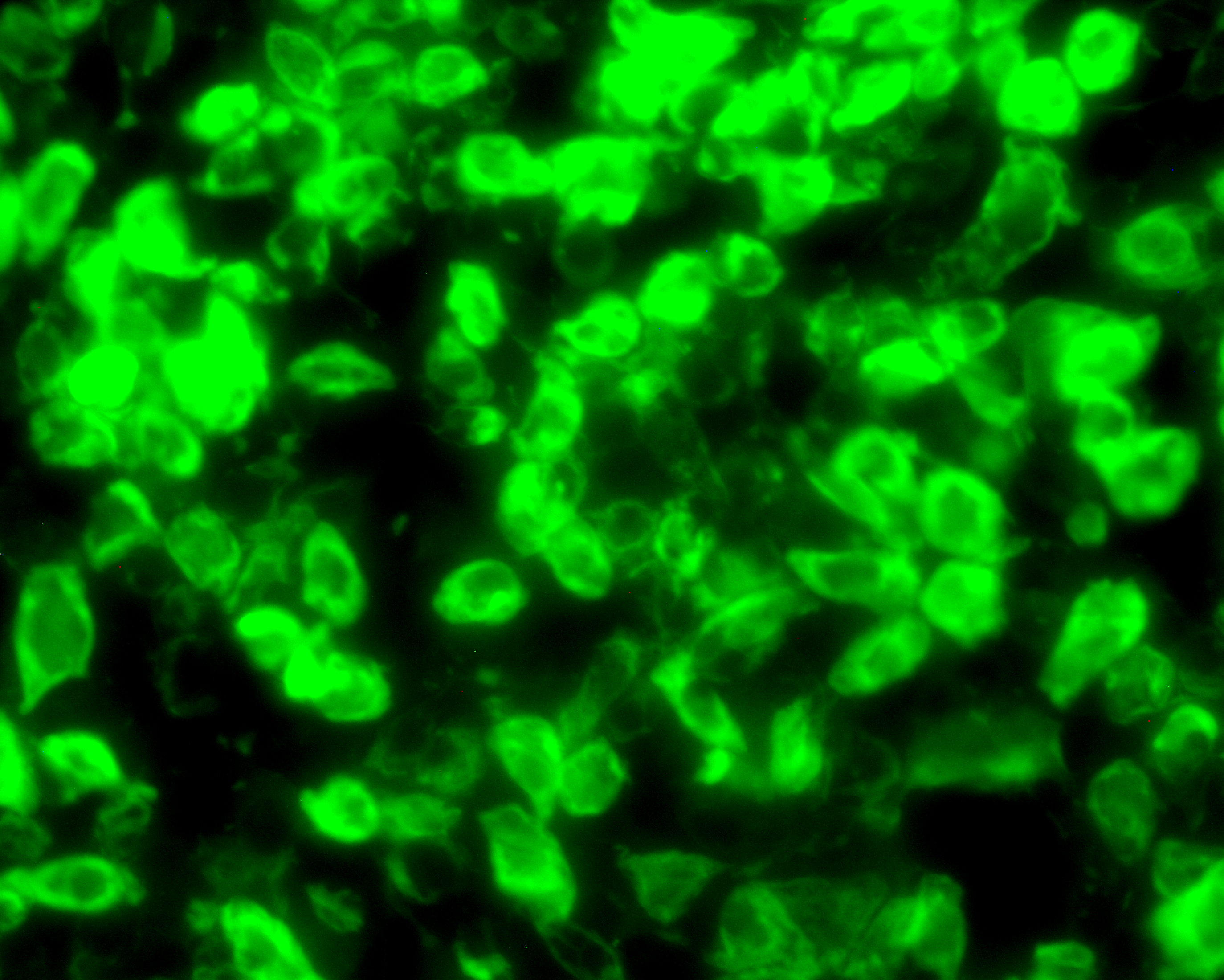

Supplement: Supplementary file 3 — Source Data for Expanded View [file EMMM-15-e17611-s013.zip › Figure EV2/EV2F/40x WT EGFP.tif]

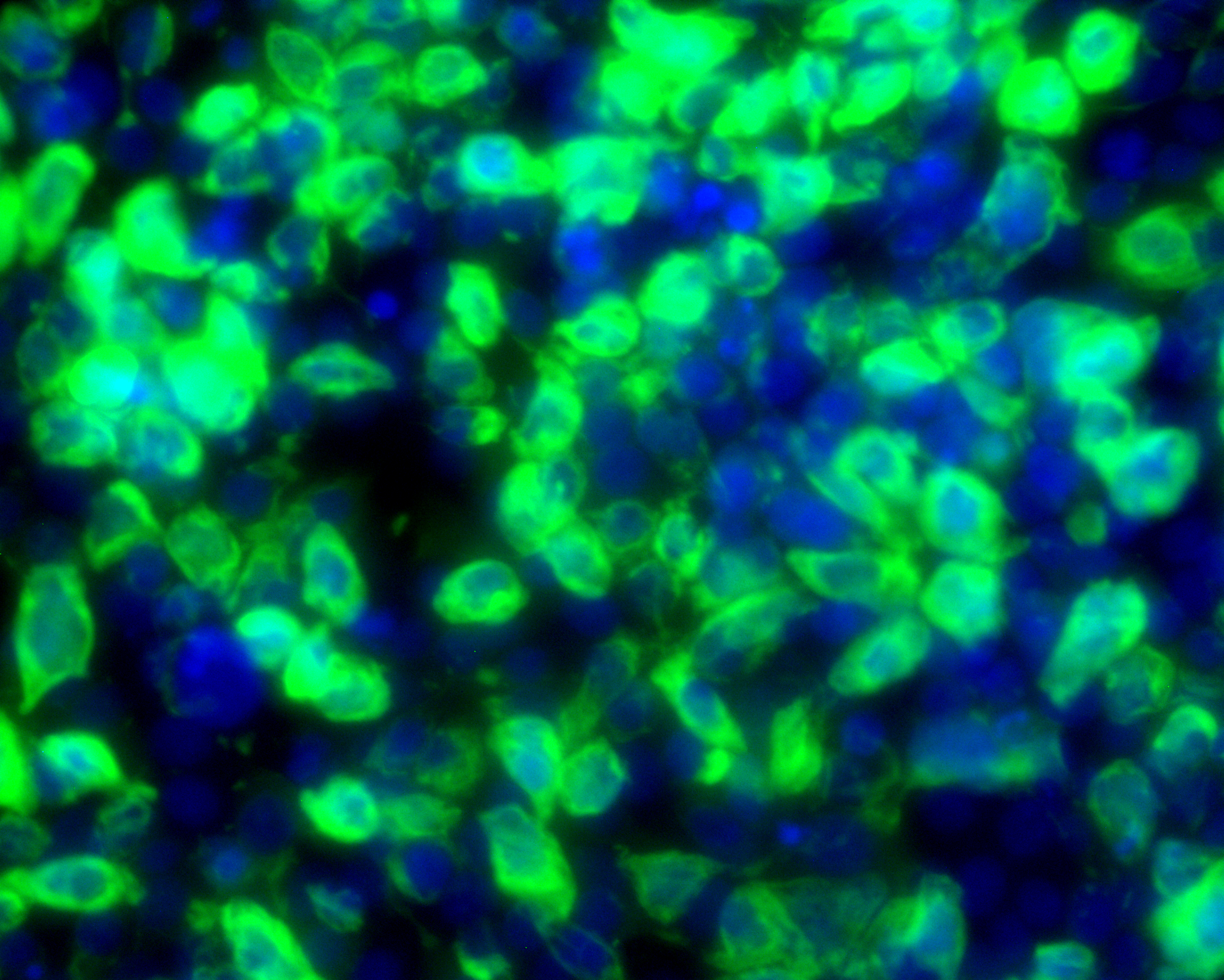

Supplement: Supplementary file 3 — Source Data for Expanded View [file EMMM-15-e17611-s013.zip › Figure EV2/EV2F/40x WT Merge.tif]

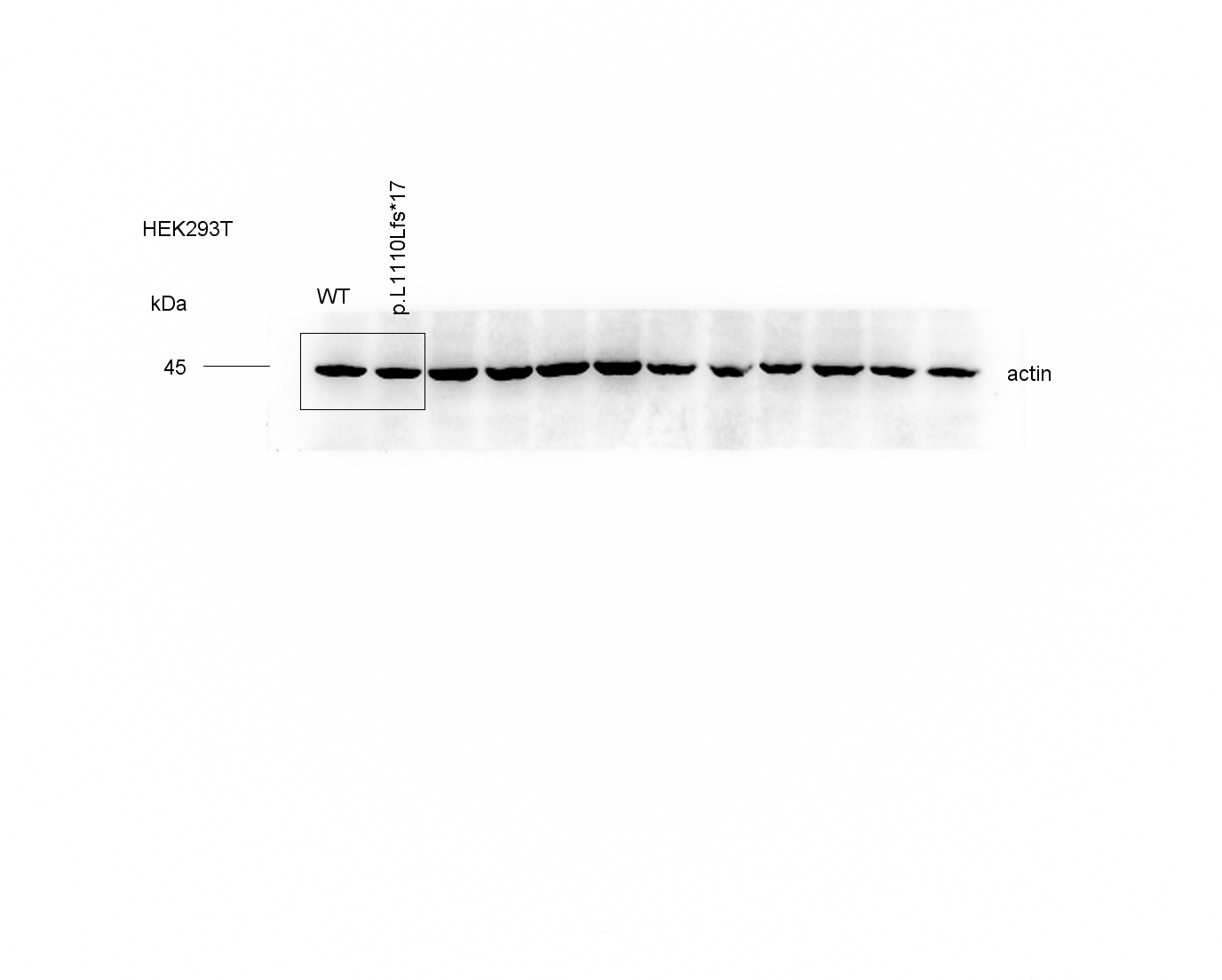

Supplement: Supplementary file 3 — Source Data for Expanded View [file EMMM-15-e17611-s013.zip › Figure EV2/EV2G/western actin.tif]

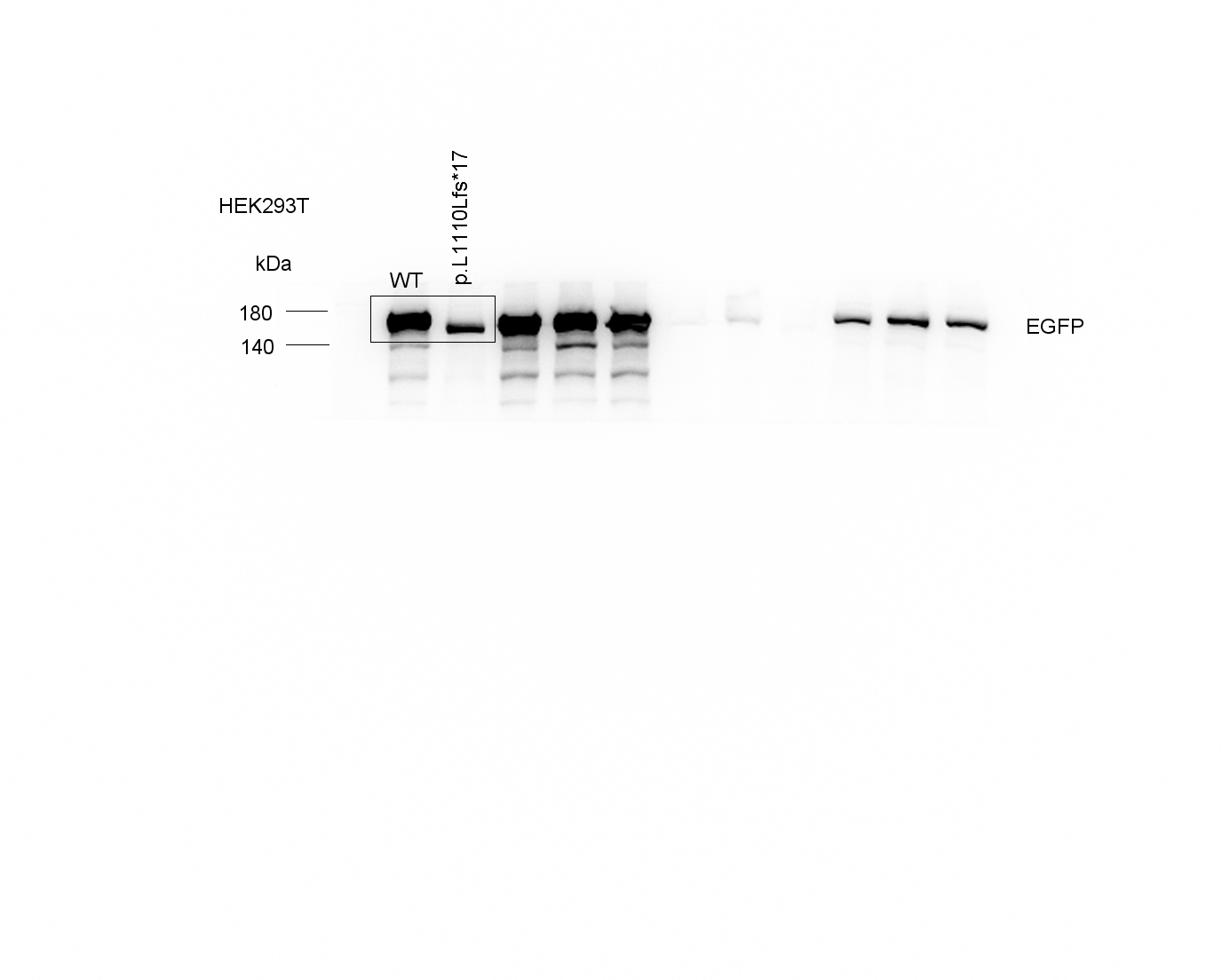

Supplement: Supplementary file 3 — Source Data for Expanded View [file EMMM-15-e17611-s013.zip › Figure EV2/EV2G/western EGFP.tif]

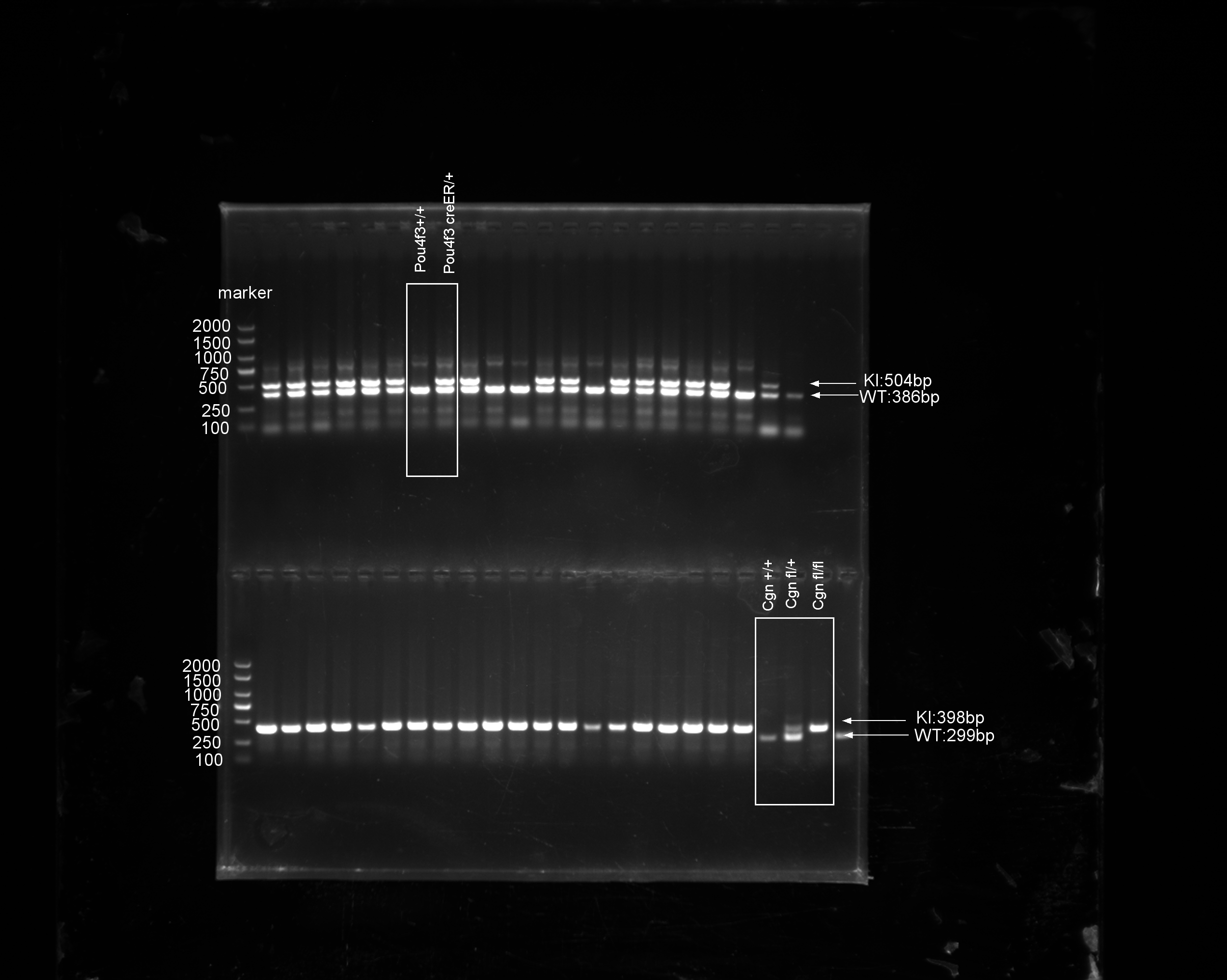

Supplement: Supplementary file 3 — Source Data for Expanded View [file EMMM-15-e17611-s013.zip › Figure EV3/EV3B/Genotyping Cgn-flox pou4f3-creER.Tif]

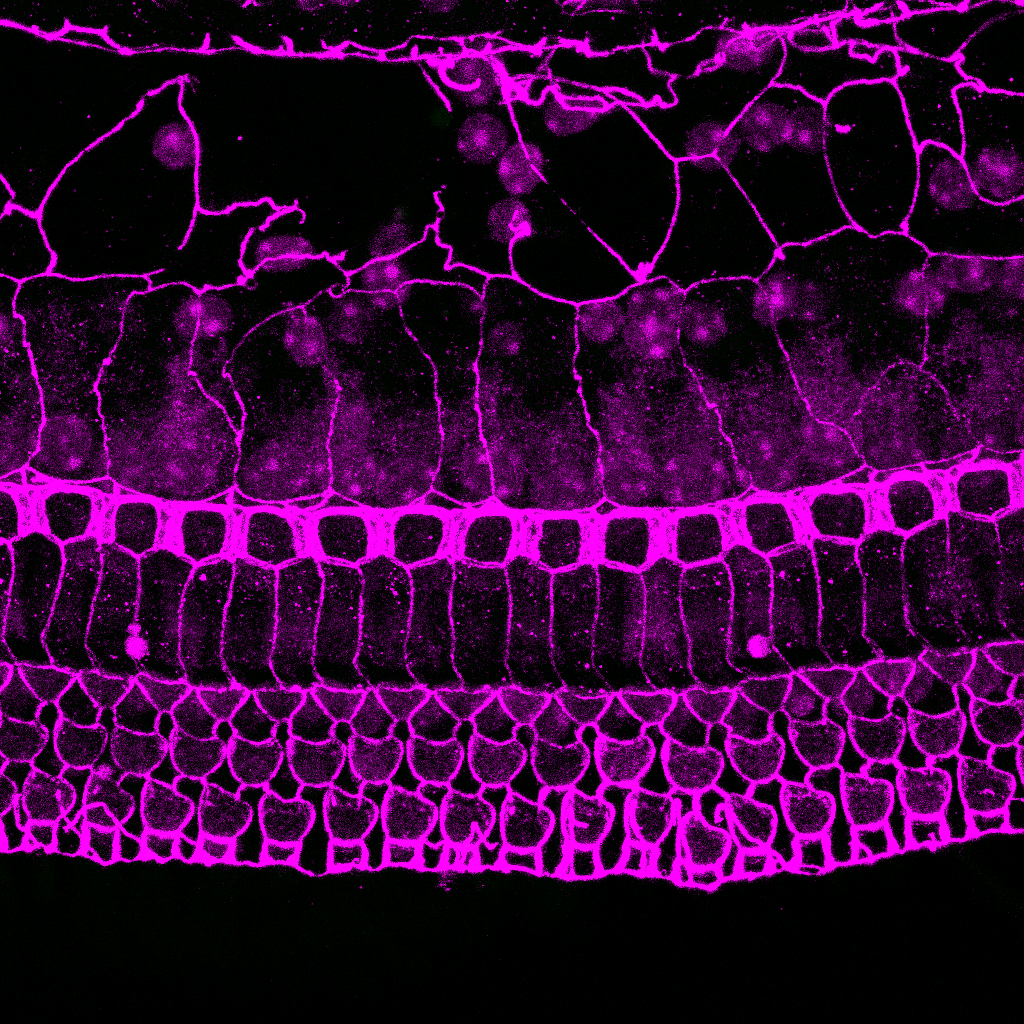

Supplement: Supplementary file 3 — Source Data for Expanded View [file EMMM-15-e17611-s013.zip › Figure EV3/EV3C/Cgn-fl CGN.tif]

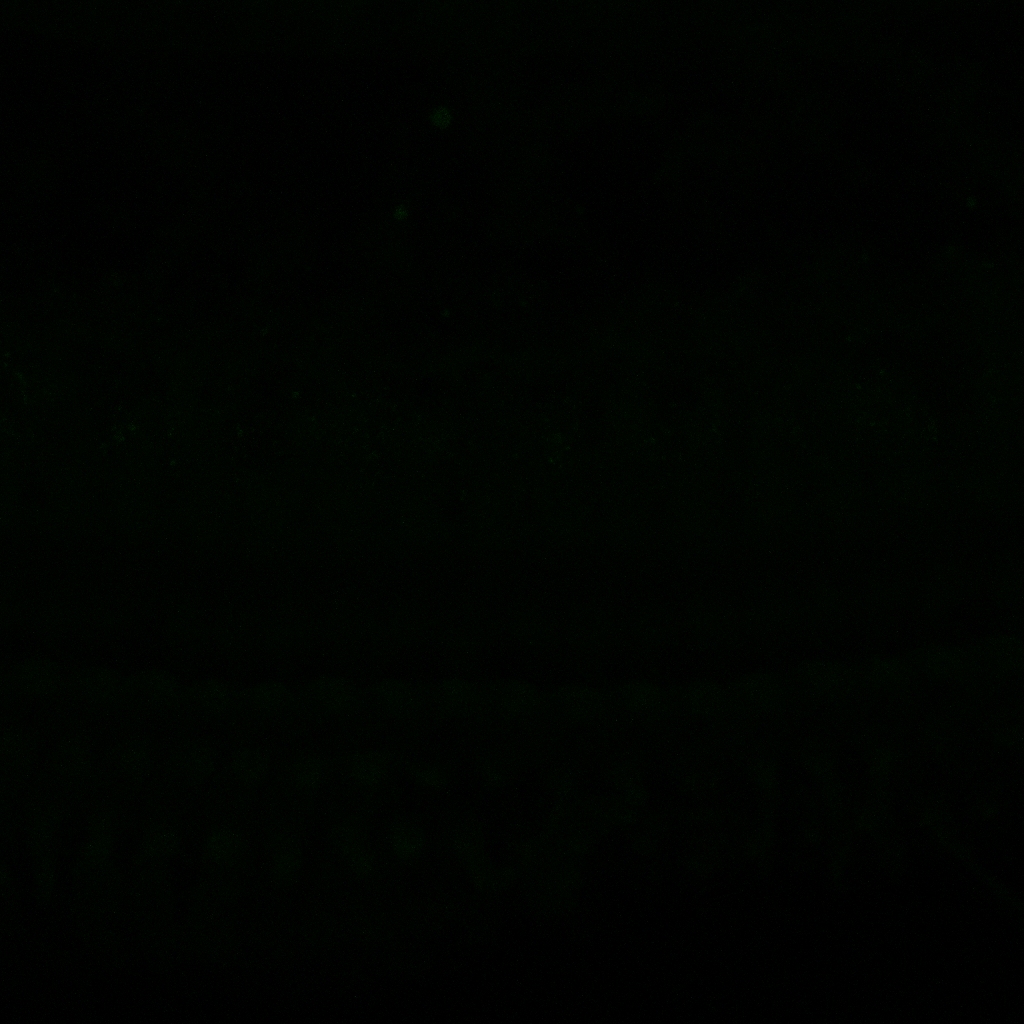

Supplement: Supplementary file 3 — Source Data for Expanded View [file EMMM-15-e17611-s013.zip › Figure EV3/EV3C/Cgn-fl EGFP.tif]

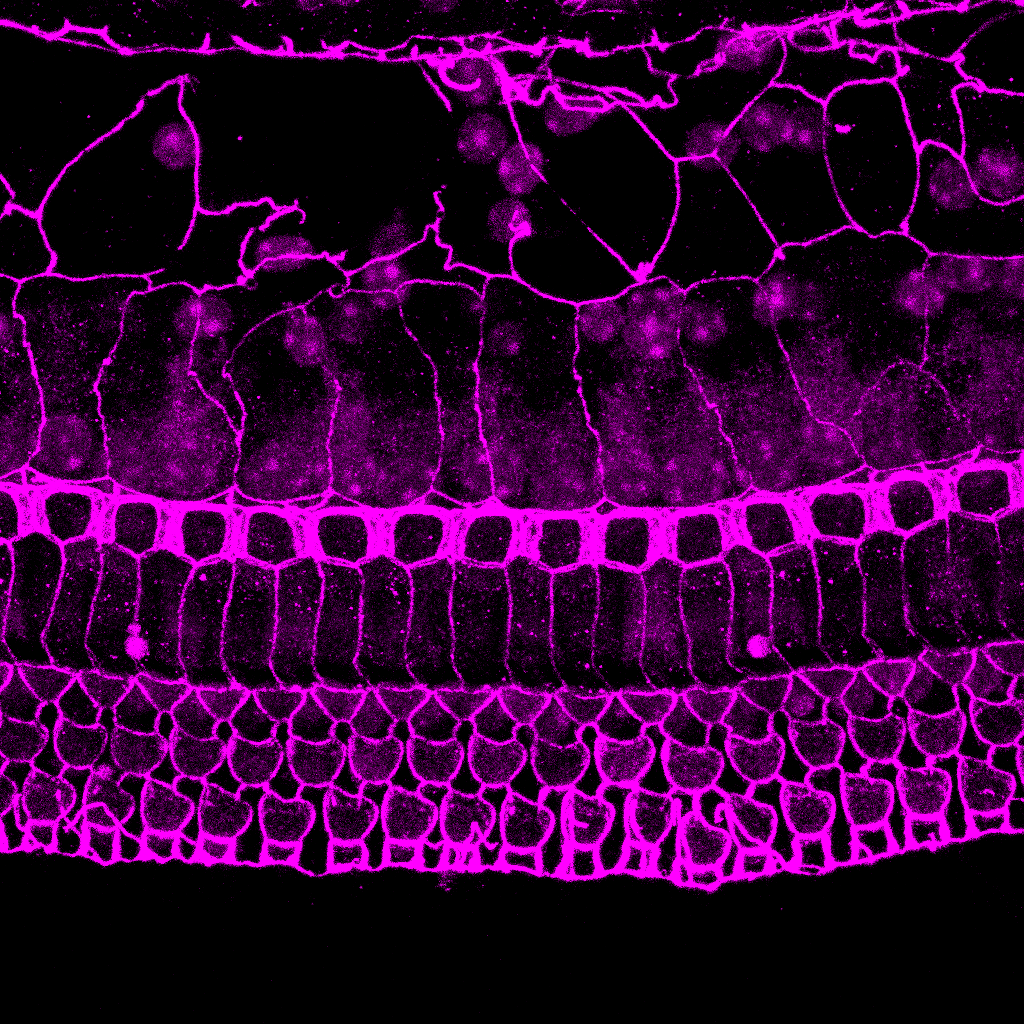

Supplement: Supplementary file 3 — Source Data for Expanded View [file EMMM-15-e17611-s013.zip › Figure EV3/EV3C/Cgn-fl Merge.tif]

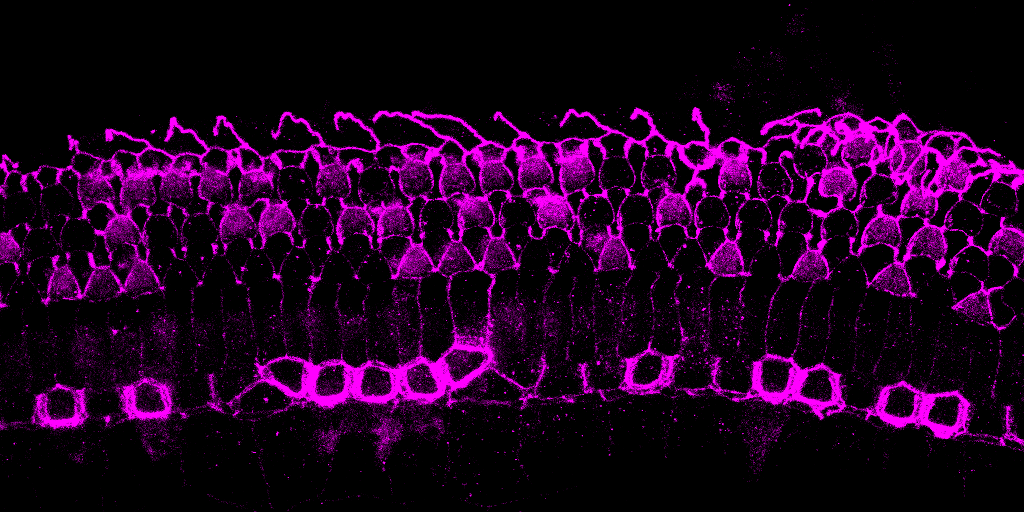

Supplement: Supplementary file 3 — Source Data for Expanded View [file EMMM-15-e17611-s013.zip › Figure EV3/EV3C/Cgn-fl-Pou4f3-creER CGN.tif]

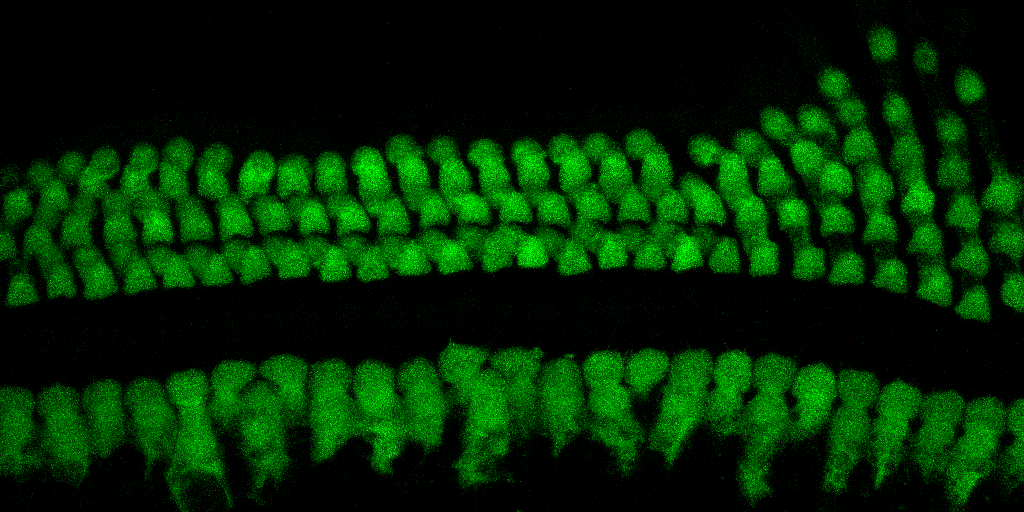

Supplement: Supplementary file 3 — Source Data for Expanded View [file EMMM-15-e17611-s013.zip › Figure EV3/EV3C/Cgn-fl-Pou4f3-creER EGFP.tif]

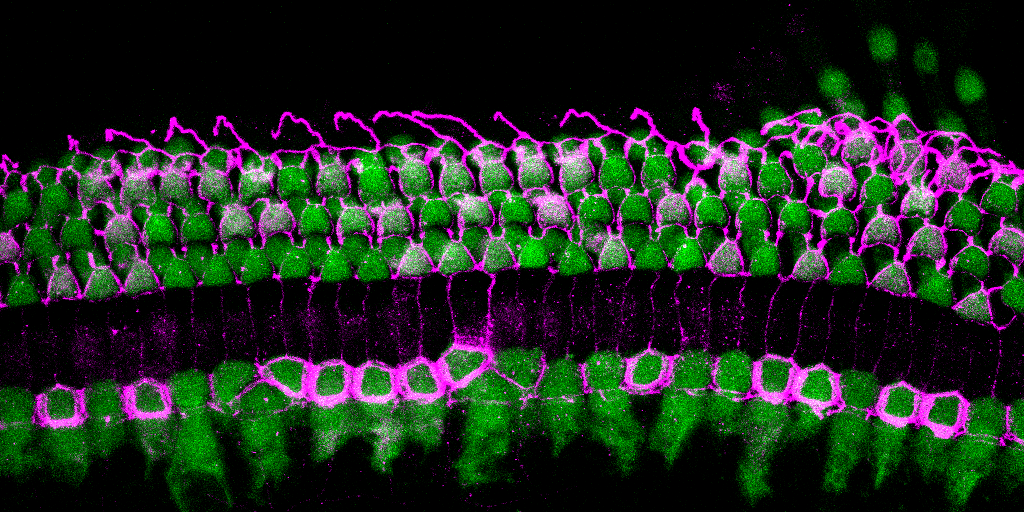

Supplement: Supplementary file 3 — Source Data for Expanded View [file EMMM-15-e17611-s013.zip › Figure EV3/EV3C/Cgn-fl-Pou4f3-creER Merge.tif]

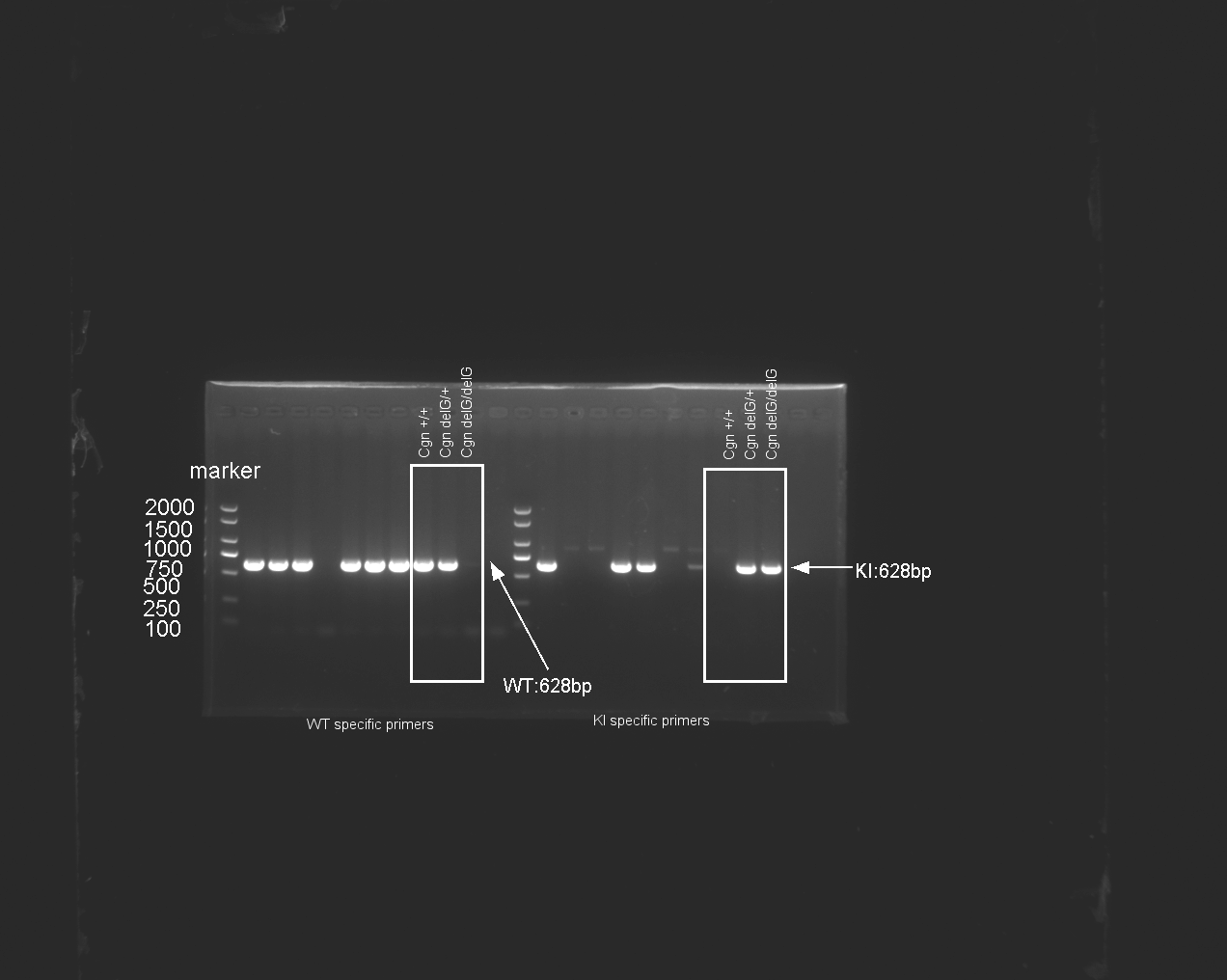

Supplement: Supplementary file 3 — Source Data for Expanded View [file EMMM-15-e17611-s013.zip › Figure EV3/EV3G/Genotyping Cgn-delG.tif]

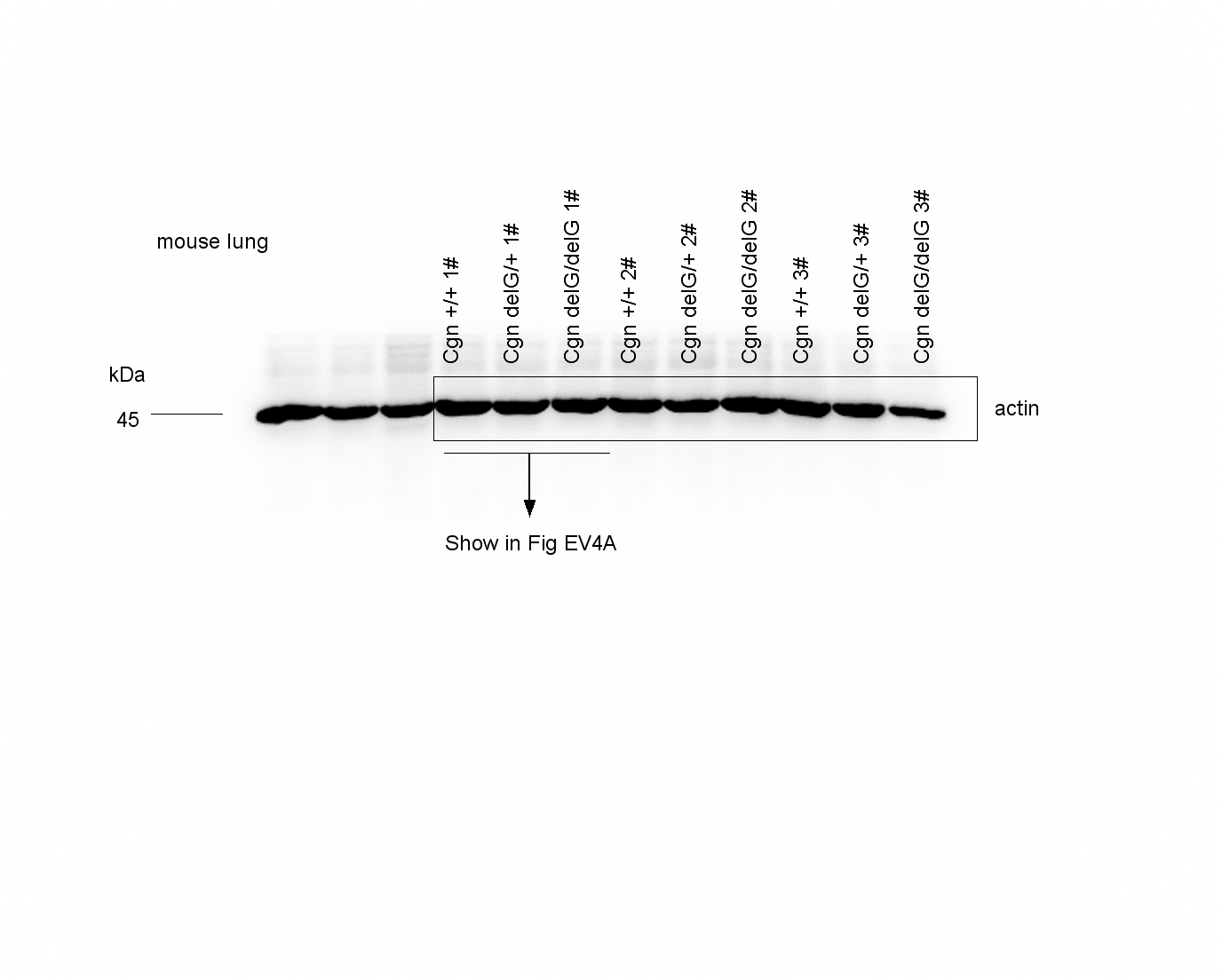

Supplement: Supplementary file 3 — Source Data for Expanded View [file EMMM-15-e17611-s013.zip › Figure EV4/EV4A/Quantification of Cgn protein expression Western blot/western actin.tif]

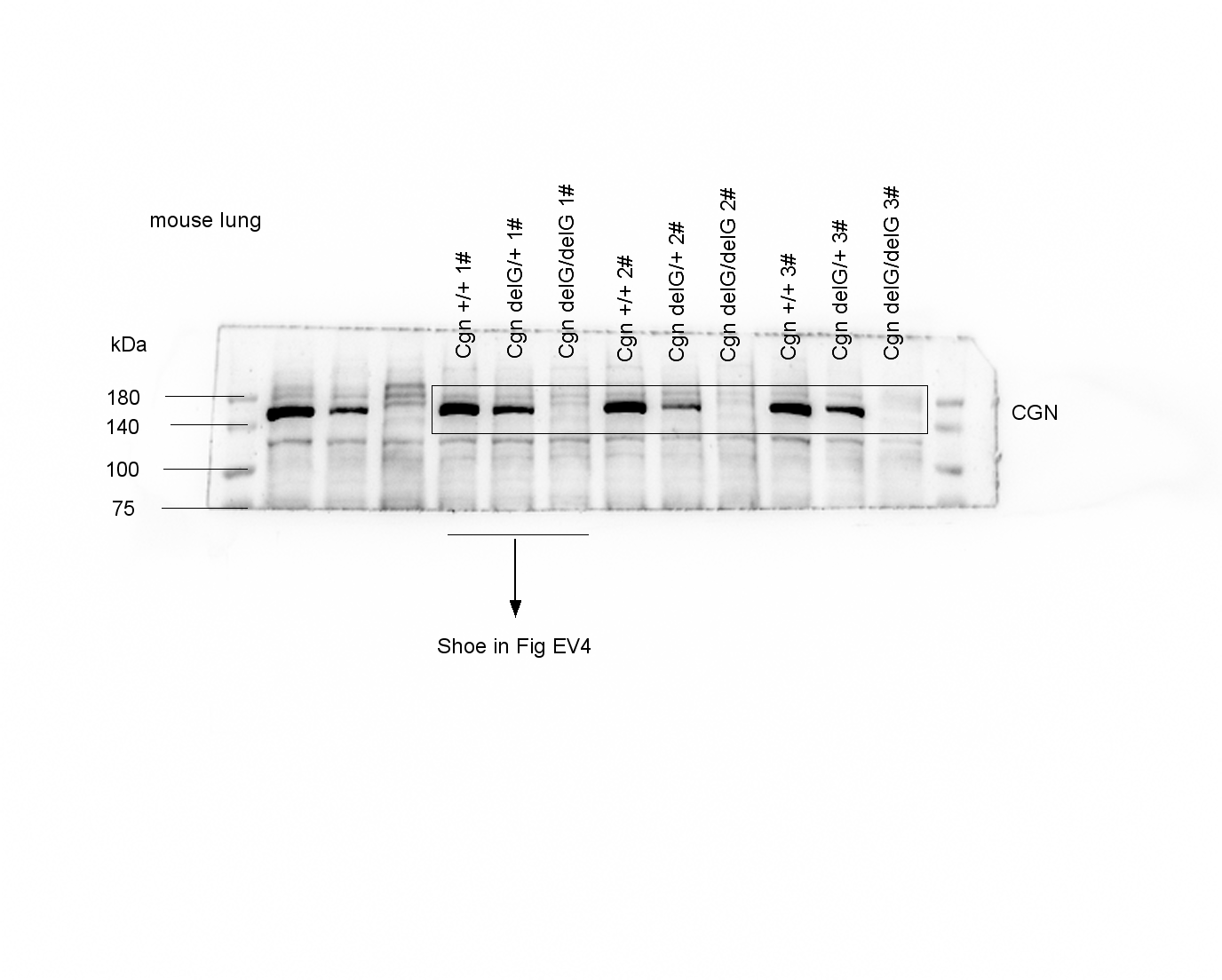

Supplement: Supplementary file 3 — Source Data for Expanded View [file EMMM-15-e17611-s013.zip › Figure EV4/EV4A/Quantification of Cgn protein expression Western blot/western CGN.tif]

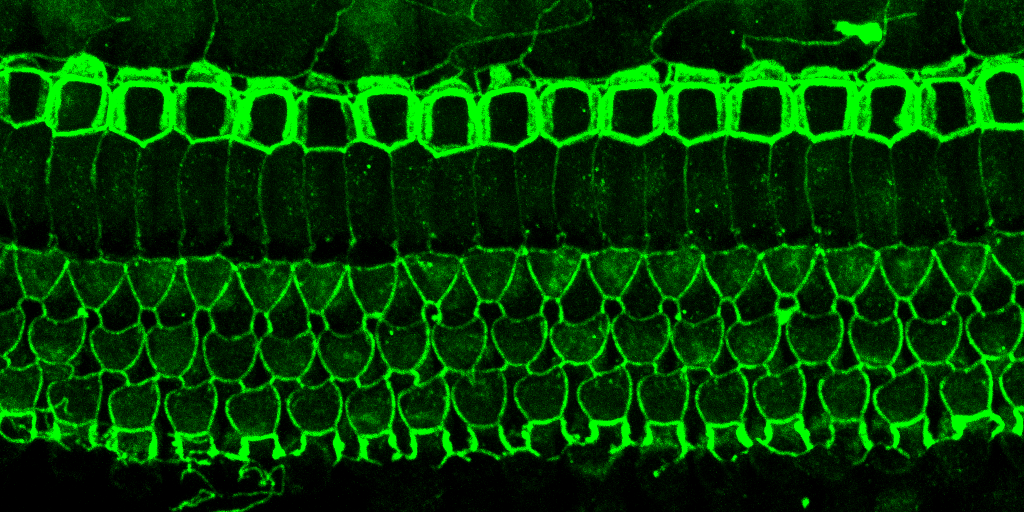

Supplement: Supplementary file 3 — Source Data for Expanded View [file EMMM-15-e17611-s013.zip › Figure EV4/EV4B/Whole mount immunofluorescence of Cgn expression/HE CGN.tif]

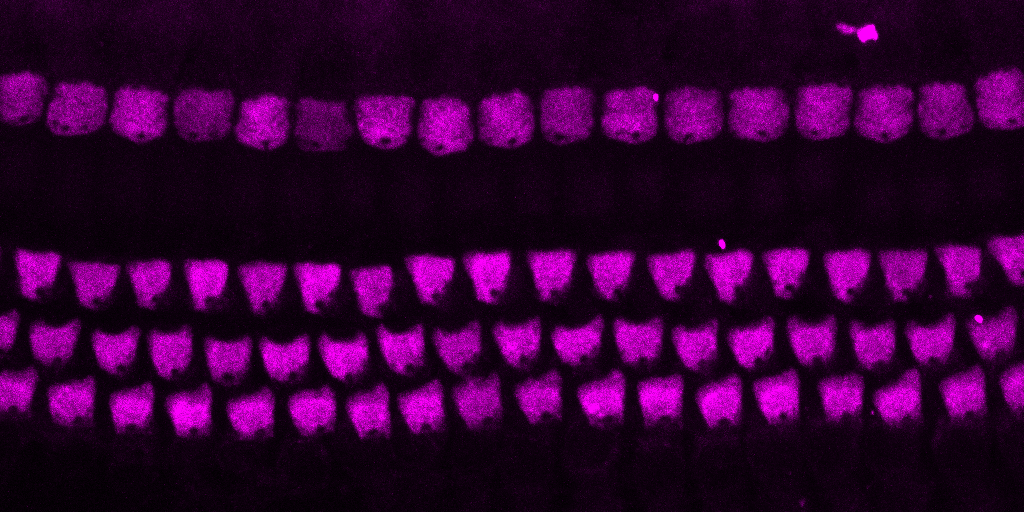

Supplement: Supplementary file 3 — Source Data for Expanded View [file EMMM-15-e17611-s013.zip › Figure EV4/EV4B/Whole mount immunofluorescence of Cgn expression/HE LMO7.tif]

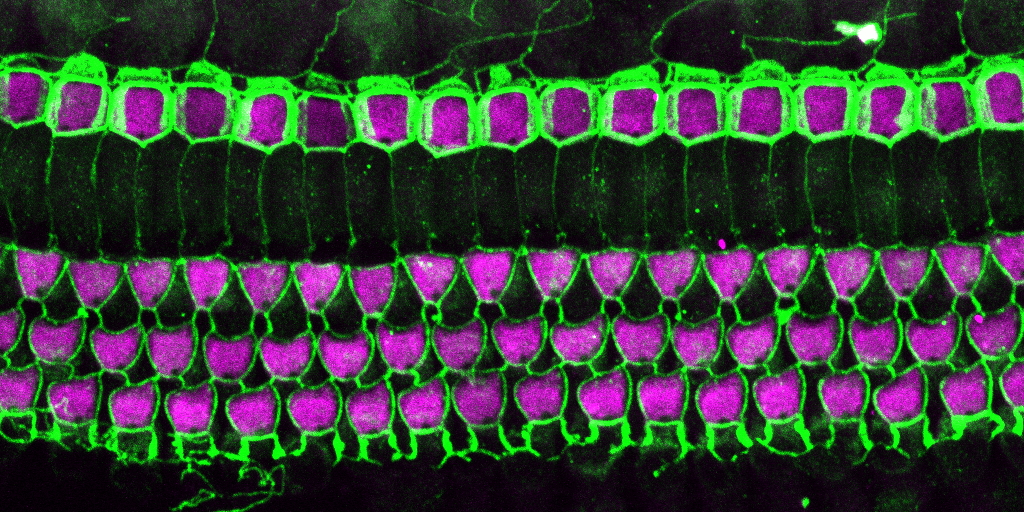

Supplement: Supplementary file 3 — Source Data for Expanded View [file EMMM-15-e17611-s013.zip › Figure EV4/EV4B/Whole mount immunofluorescence of Cgn expression/HE Merge.tif]

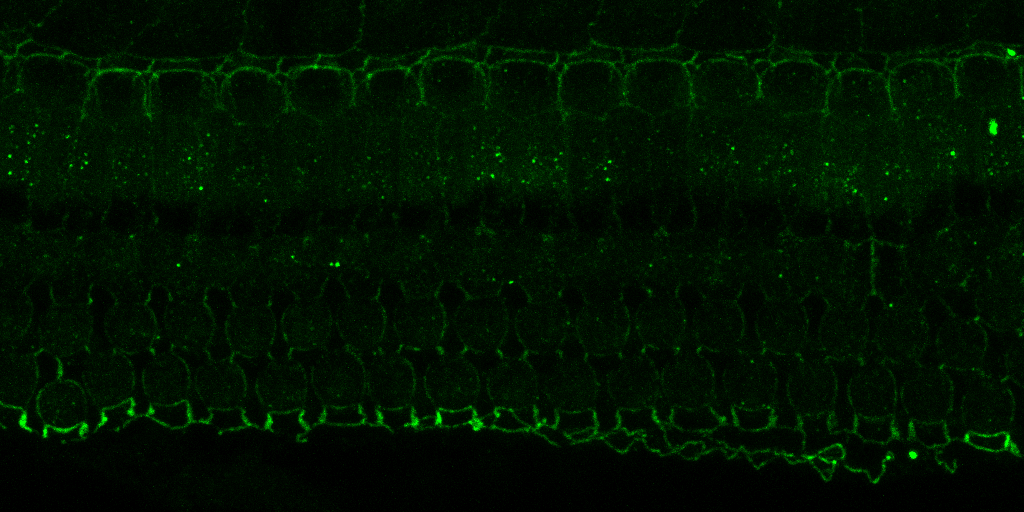

Supplement: Supplementary file 3 — Source Data for Expanded View [file EMMM-15-e17611-s013.zip › Figure EV4/EV4B/Whole mount immunofluorescence of Cgn expression/HO CGN.tif]

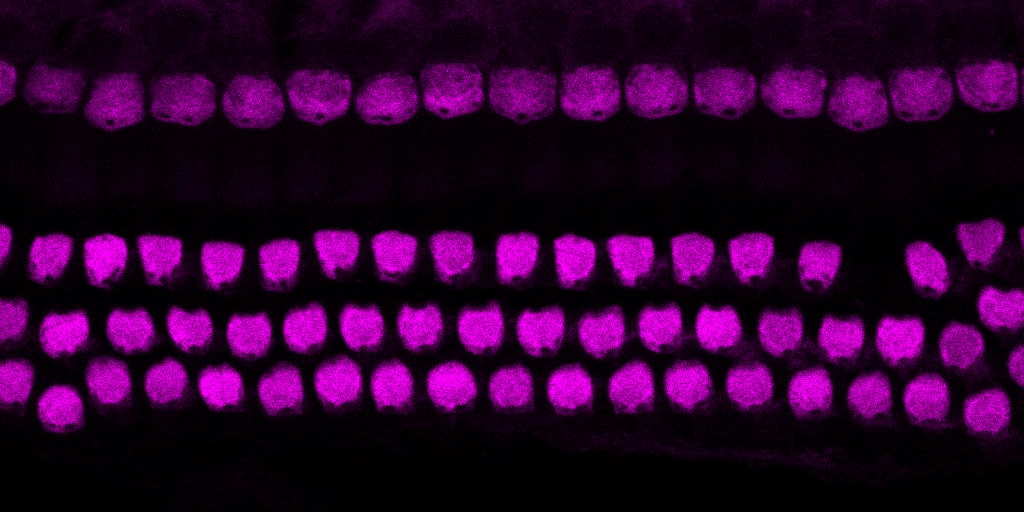

Supplement: Supplementary file 3 — Source Data for Expanded View [file EMMM-15-e17611-s013.zip › Figure EV4/EV4B/Whole mount immunofluorescence of Cgn expression/HO LMO7.tif]

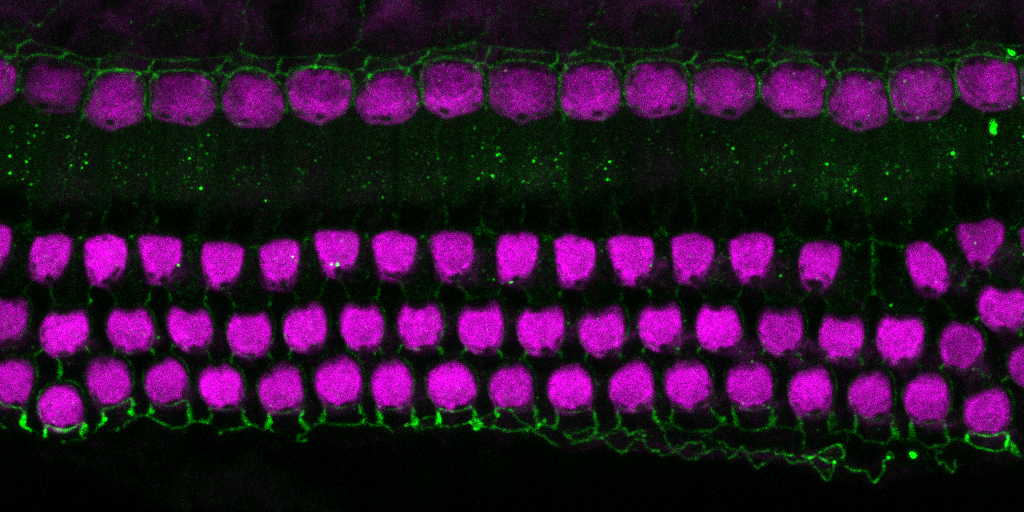

Supplement: Supplementary file 3 — Source Data for Expanded View [file EMMM-15-e17611-s013.zip › Figure EV4/EV4B/Whole mount immunofluorescence of Cgn expression/HO Merge.tif]

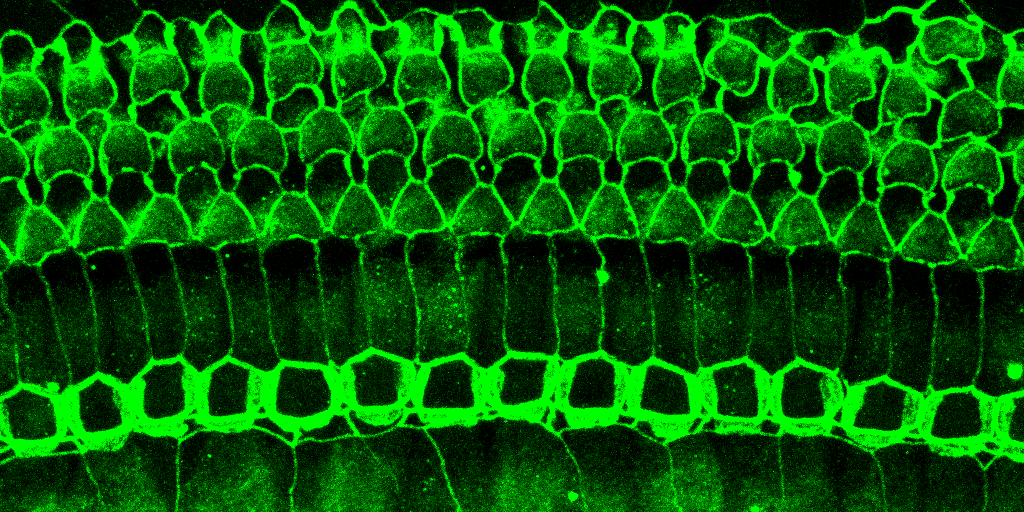

Supplement: Supplementary file 3 — Source Data for Expanded View [file EMMM-15-e17611-s013.zip › Figure EV4/EV4B/Whole mount immunofluorescence of Cgn expression/WT CGN.tif]

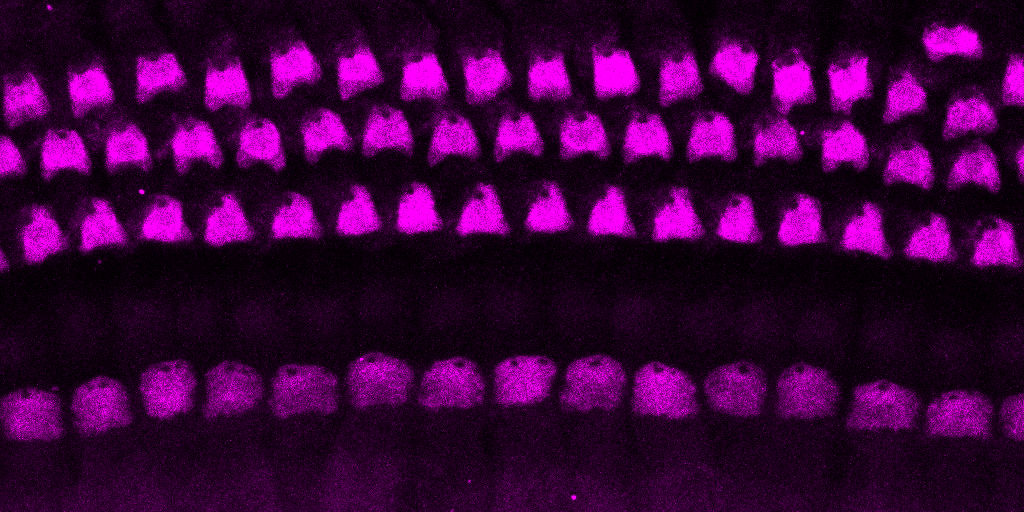

Supplement: Supplementary file 3 — Source Data for Expanded View [file EMMM-15-e17611-s013.zip › Figure EV4/EV4B/Whole mount immunofluorescence of Cgn expression/WT LMO7.tif]

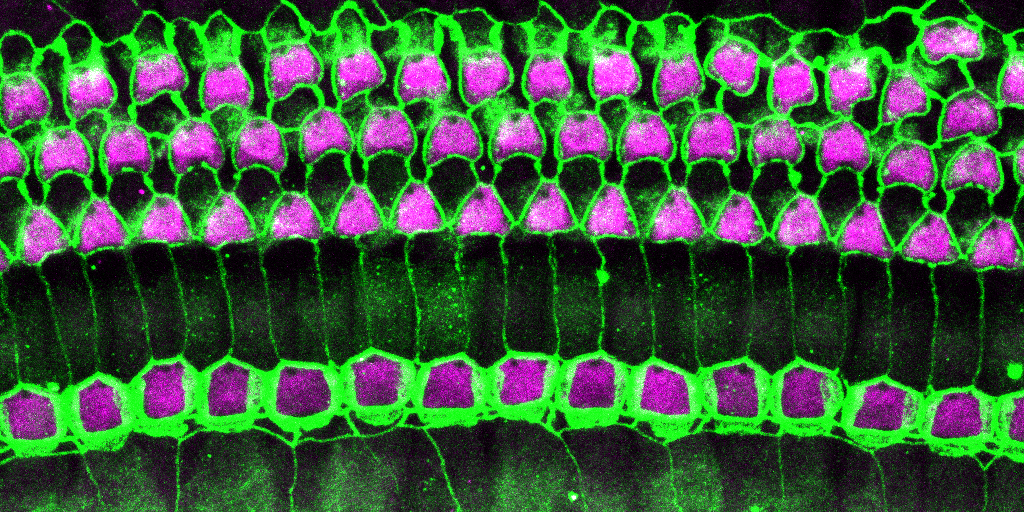

Supplement: Supplementary file 3 — Source Data for Expanded View [file EMMM-15-e17611-s013.zip › Figure EV4/EV4B/Whole mount immunofluorescence of Cgn expression/WT Merge.tif]

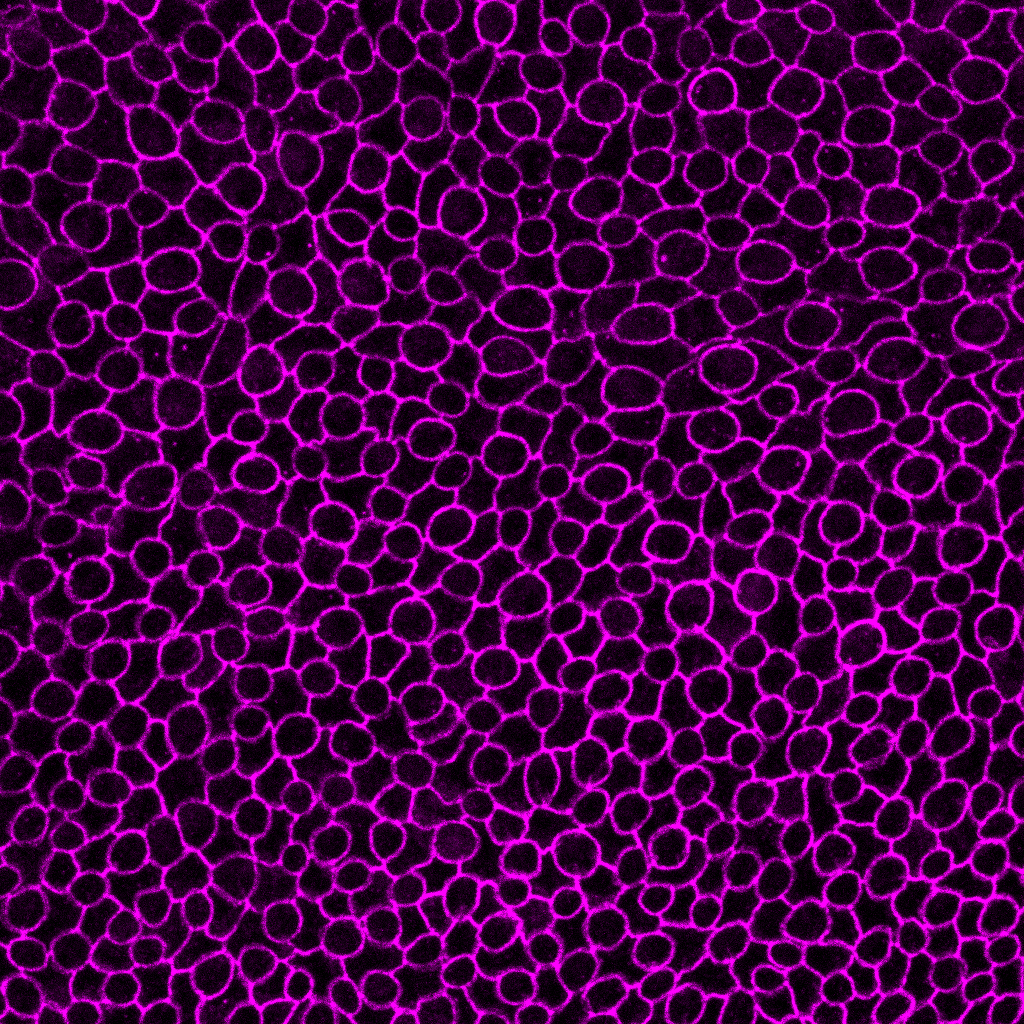

Supplement: Supplementary file 3 — Source Data for Expanded View [file EMMM-15-e17611-s013.zip › Figure EV5/EV5A/P14 utricle CGN.tif]

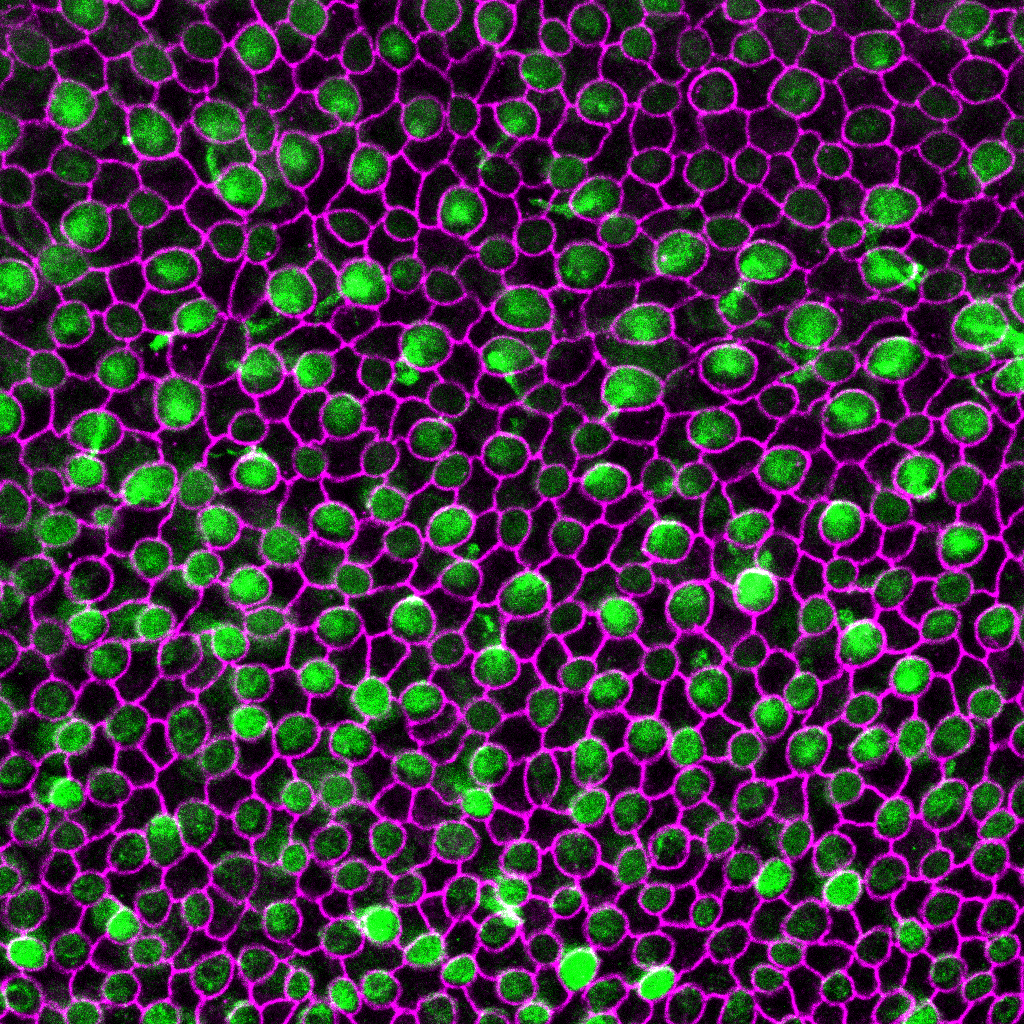

Supplement: Supplementary file 3 — Source Data for Expanded View [file EMMM-15-e17611-s013.zip › Figure EV5/EV5A/P14 utricle Merge.tif]

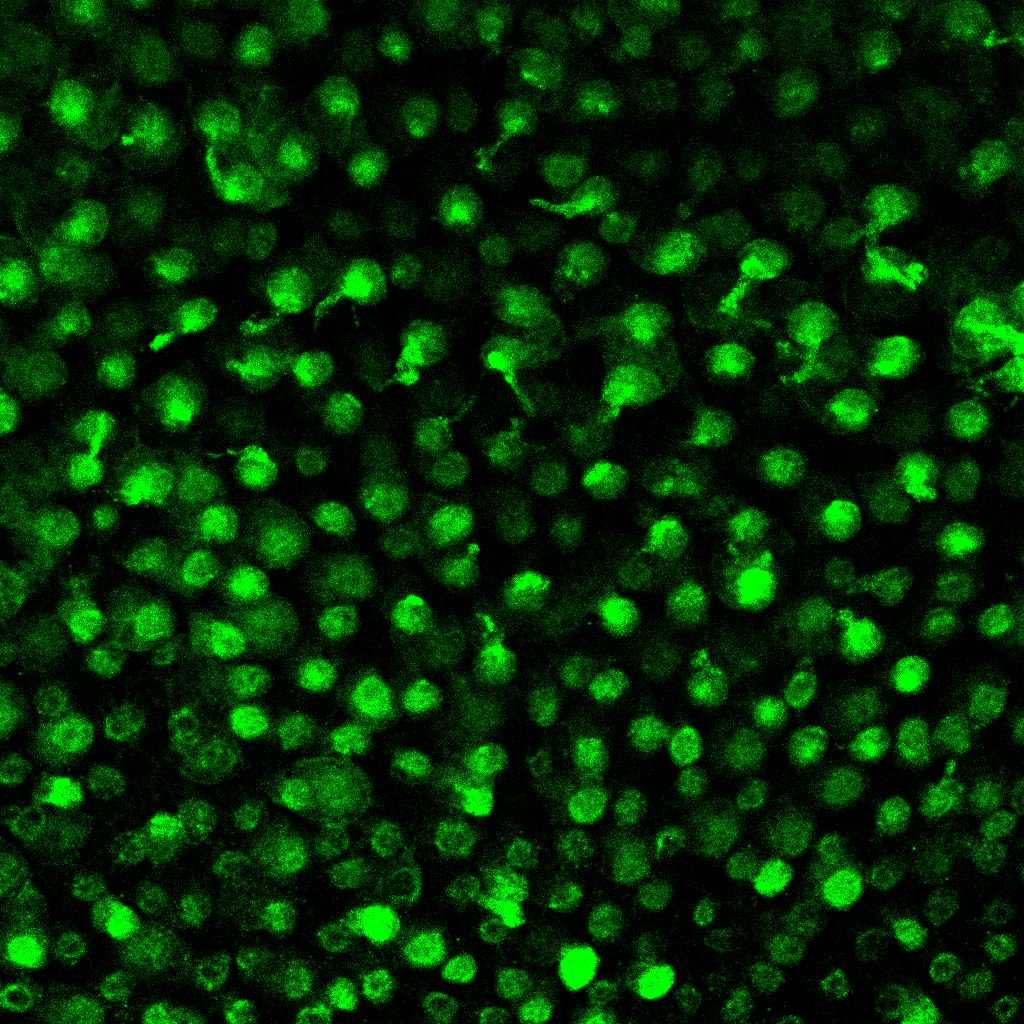

Supplement: Supplementary file 3 — Source Data for Expanded View [file EMMM-15-e17611-s013.zip › Figure EV5/EV5A/P14 utricle Parvalbumin.tif]

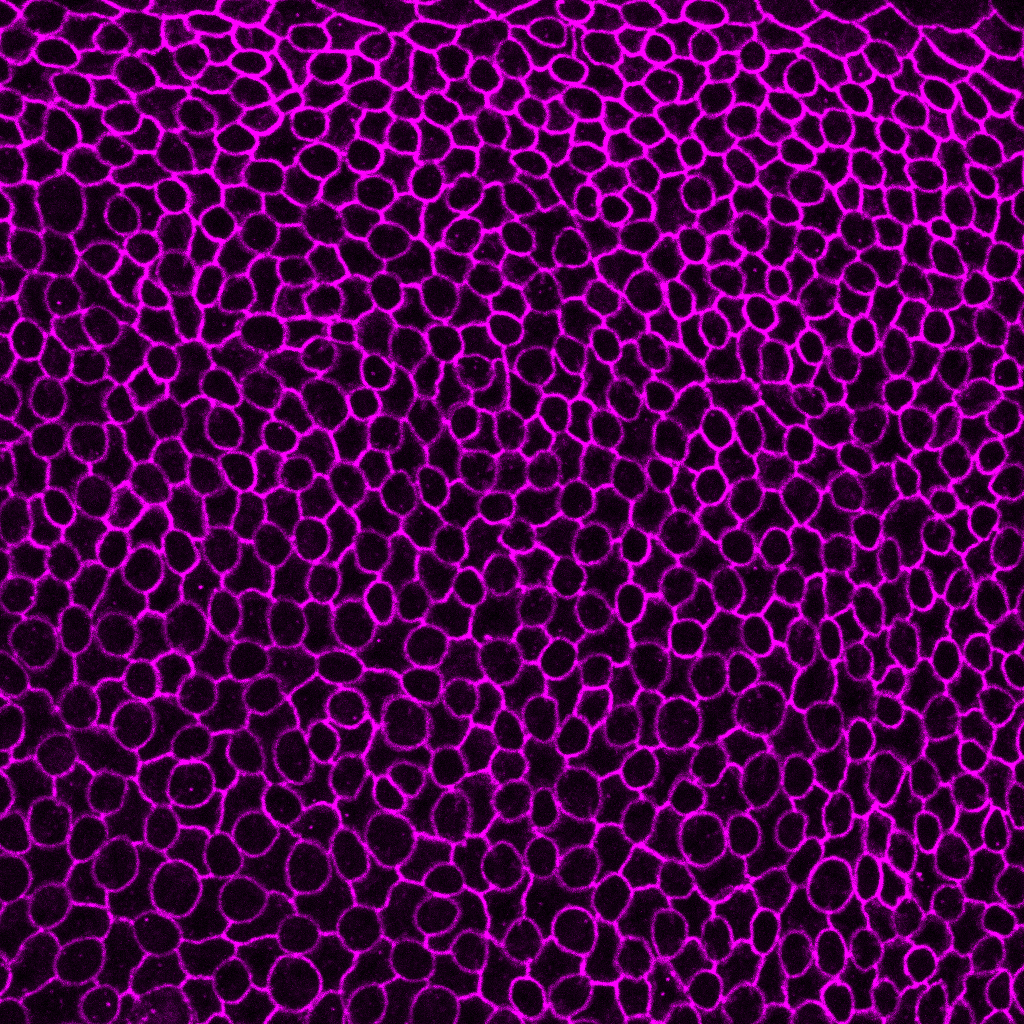

Supplement: Supplementary file 3 — Source Data for Expanded View [file EMMM-15-e17611-s013.zip › Figure EV5/EV5A/P21 utricle CGN.tif]

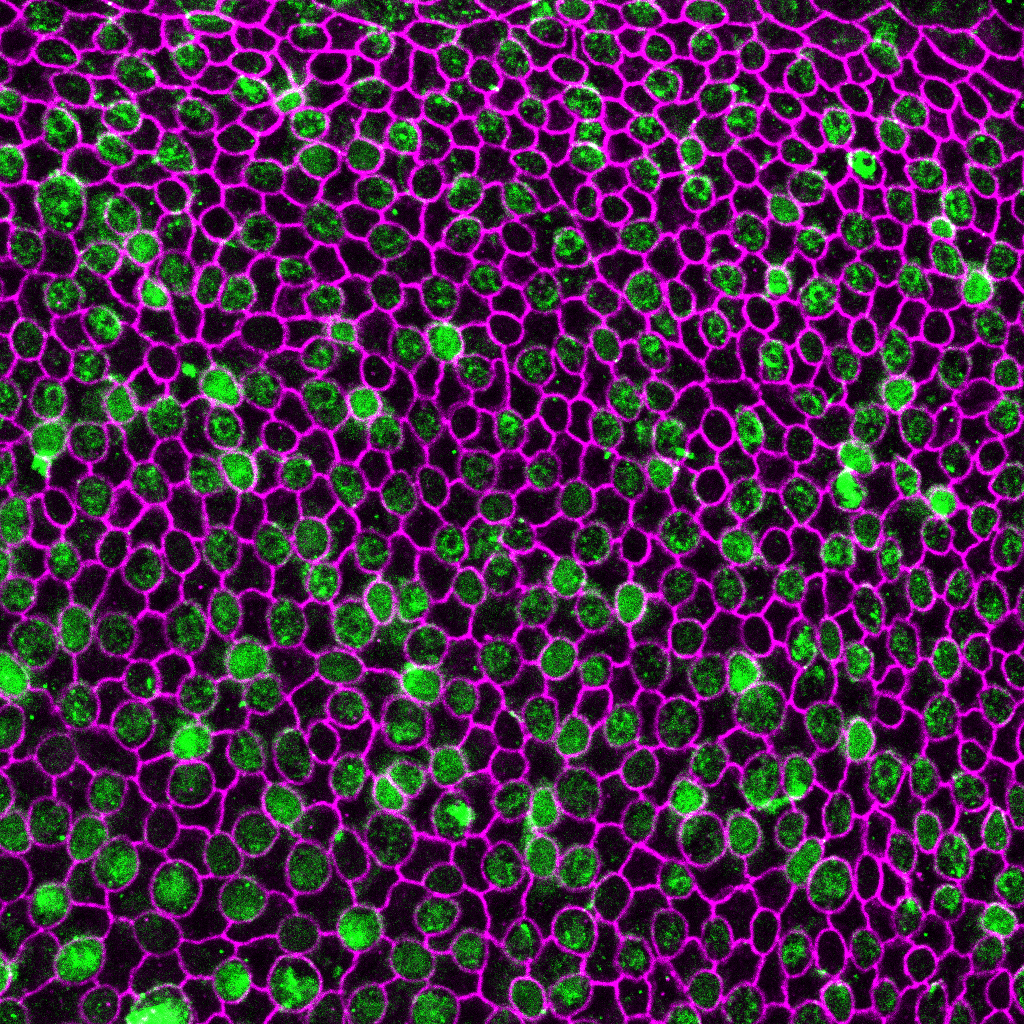

Supplement: Supplementary file 3 — Source Data for Expanded View [file EMMM-15-e17611-s013.zip › Figure EV5/EV5A/P21 utricle Merge.tif]

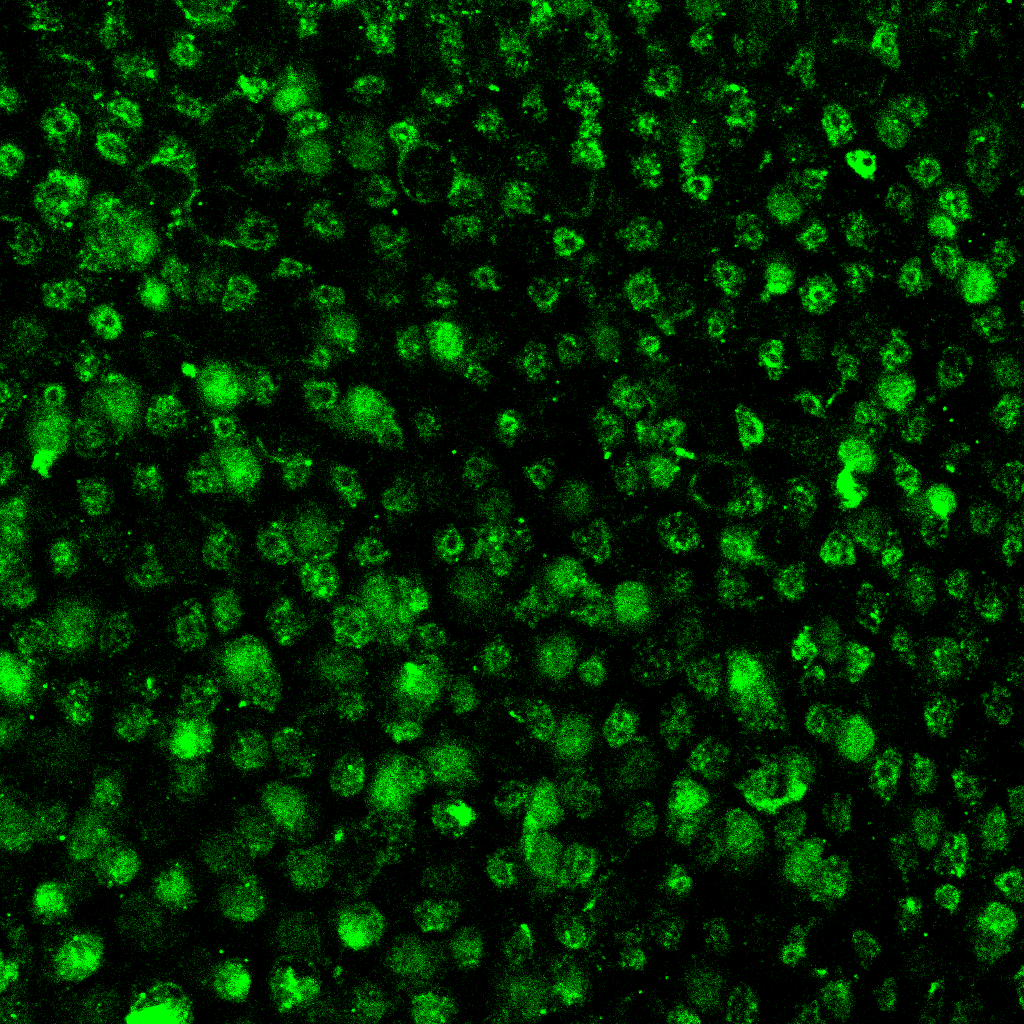

Supplement: Supplementary file 3 — Source Data for Expanded View [file EMMM-15-e17611-s013.zip › Figure EV5/EV5A/P21 utricle Parvalbumin.tif]

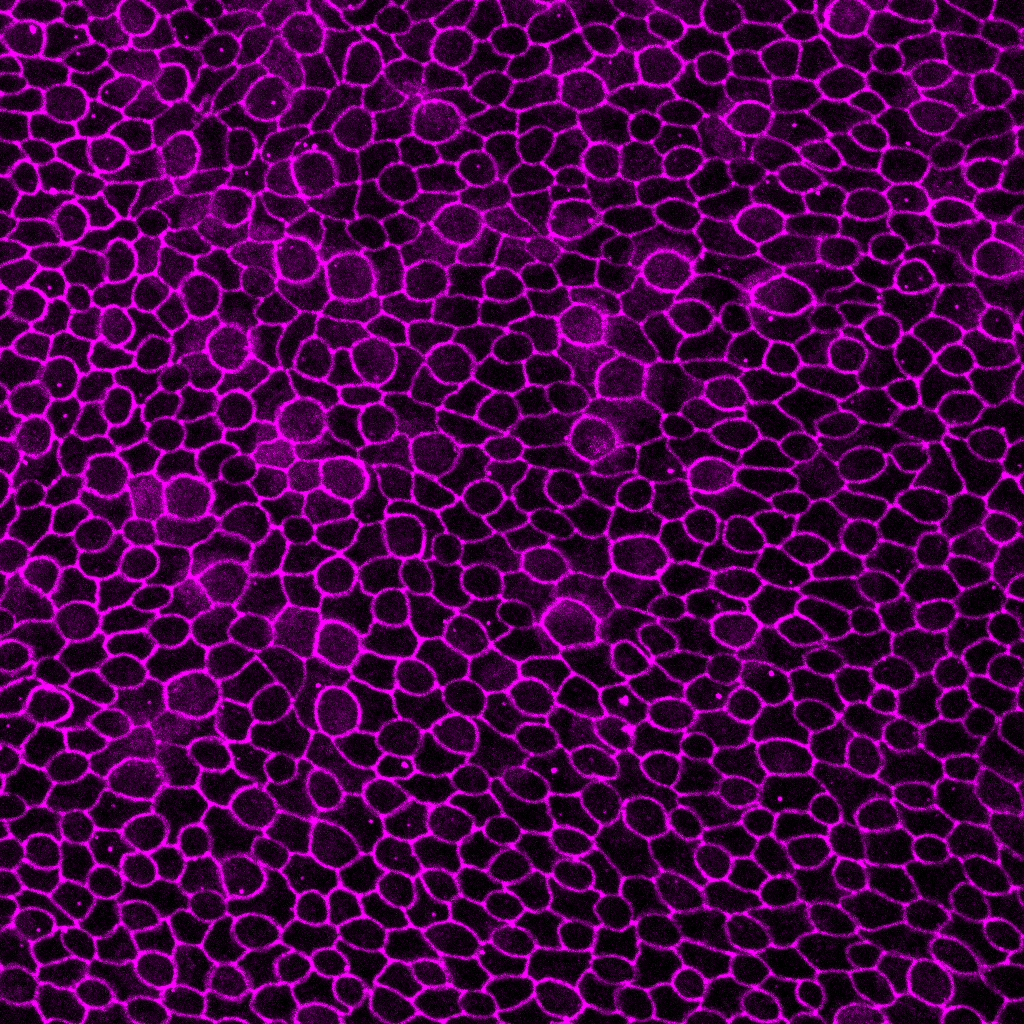

Supplement: Supplementary file 3 — Source Data for Expanded View [file EMMM-15-e17611-s013.zip › Figure EV5/EV5A/P3 utricle CGN.tif]

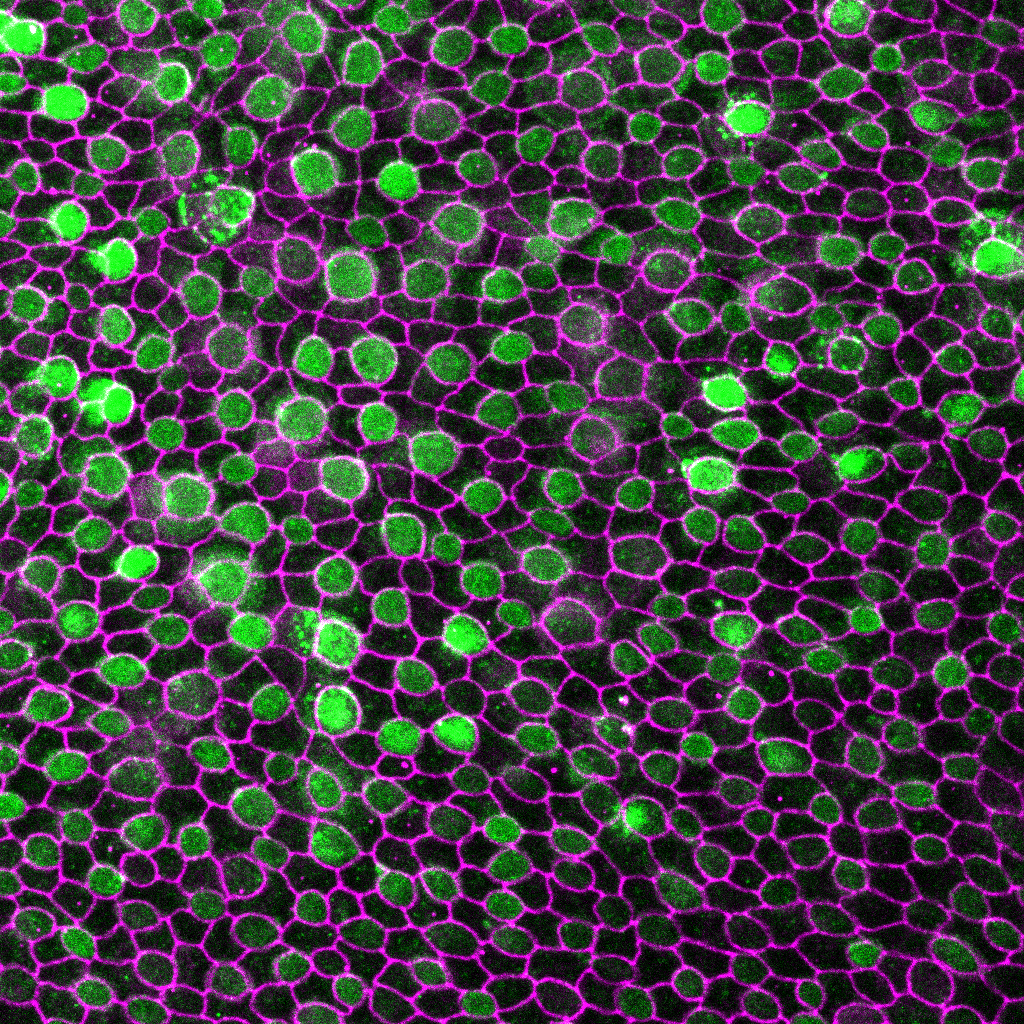

Supplement: Supplementary file 3 — Source Data for Expanded View [file EMMM-15-e17611-s013.zip › Figure EV5/EV5A/P3 utricle Merge.tif]

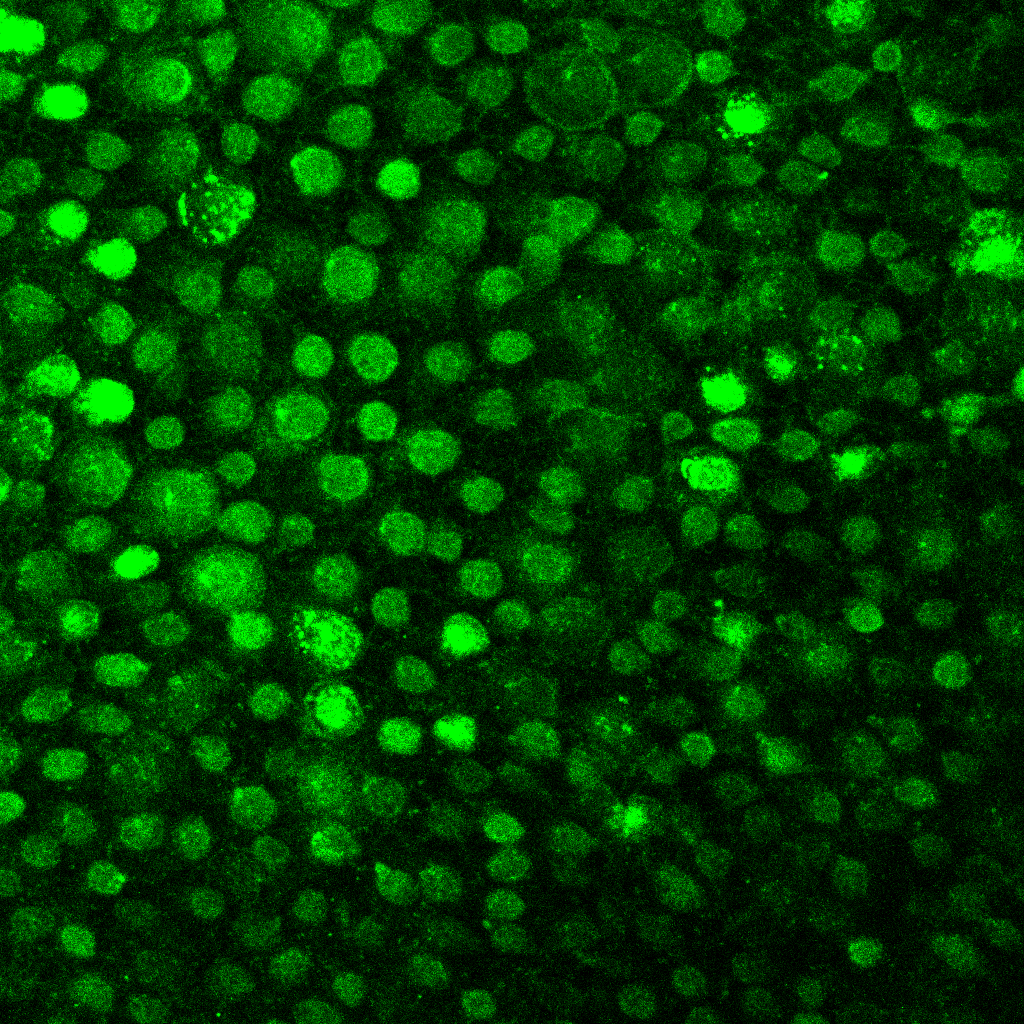

Supplement: Supplementary file 3 — Source Data for Expanded View [file EMMM-15-e17611-s013.zip › Figure EV5/EV5A/P3 utricle Parvalbumin.tif]

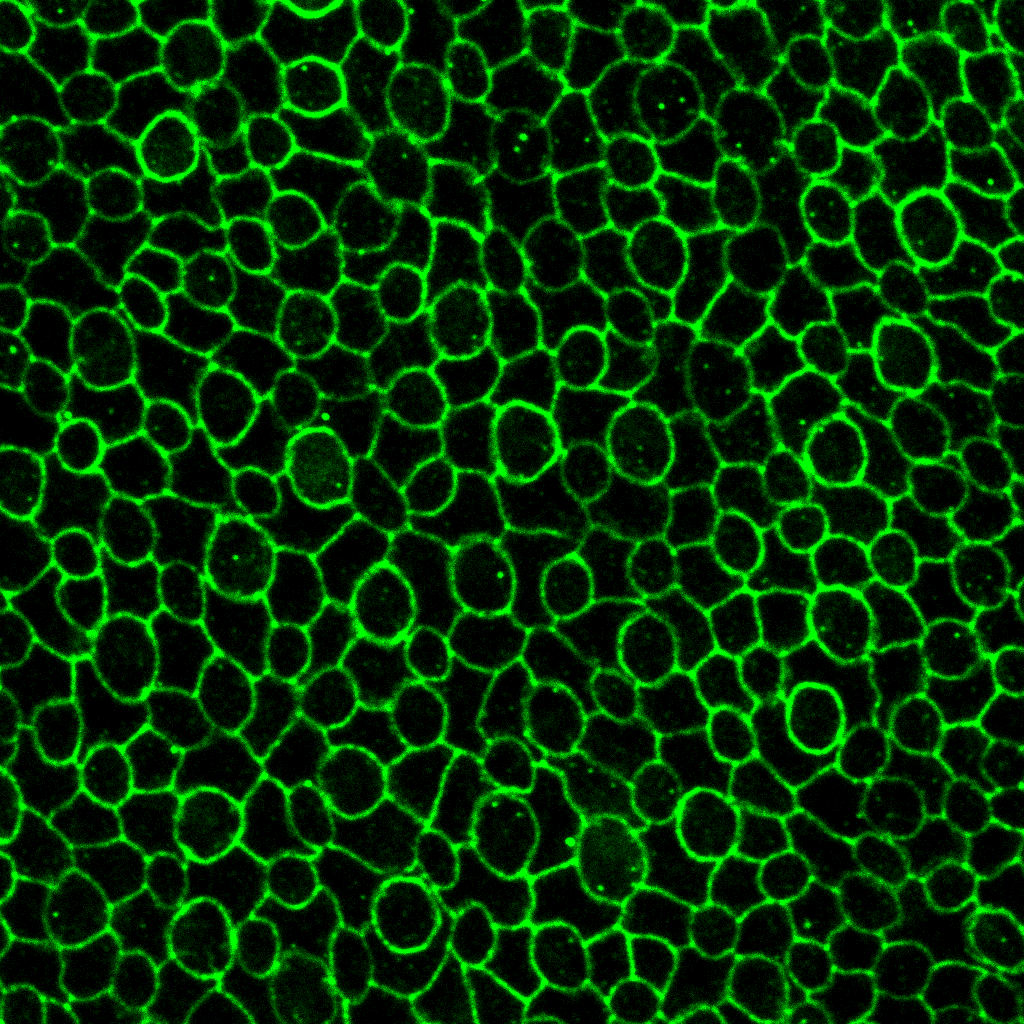

Supplement: Supplementary file 3 — Source Data for Expanded View [file EMMM-15-e17611-s013.zip › Figure EV5/EV5B/P14 utricle CGN.tif]

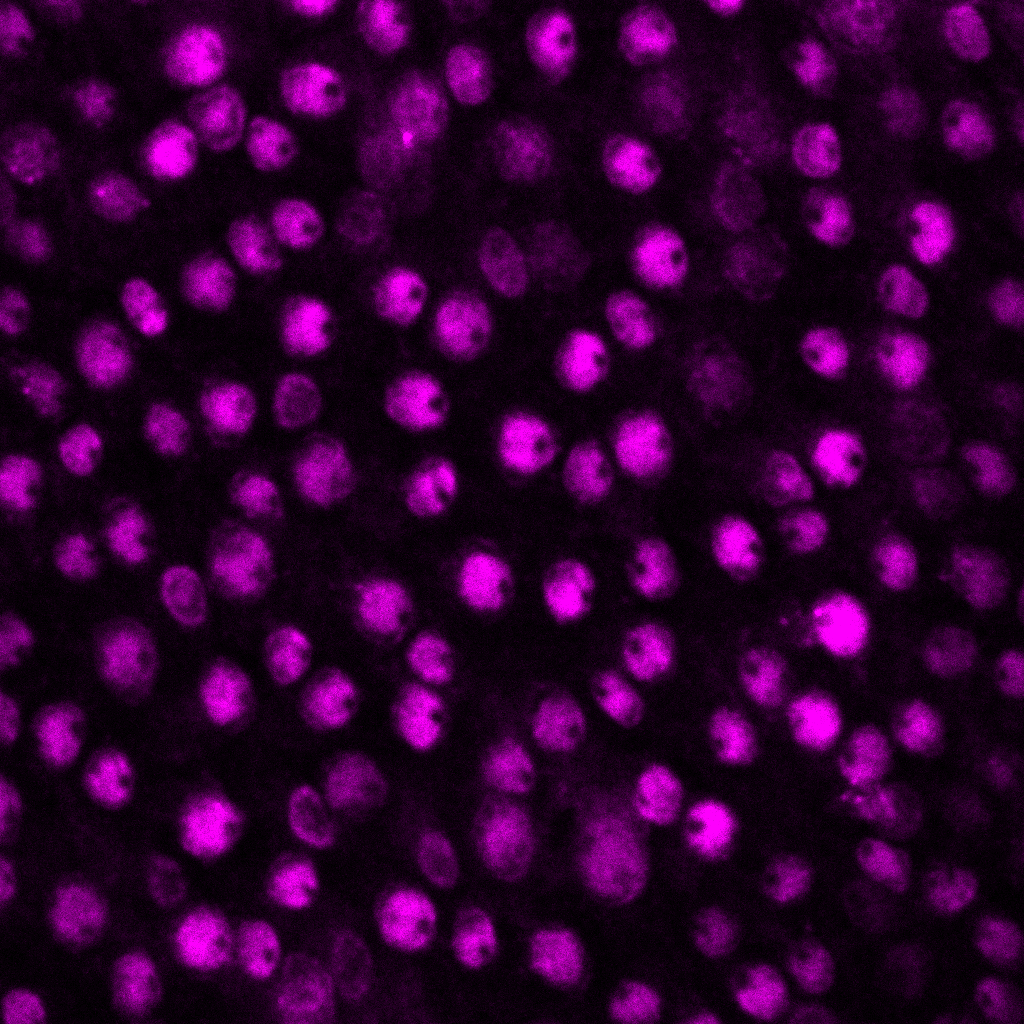

Supplement: Supplementary file 3 — Source Data for Expanded View [file EMMM-15-e17611-s013.zip › Figure EV5/EV5B/P14 utricle LMO7.tif]

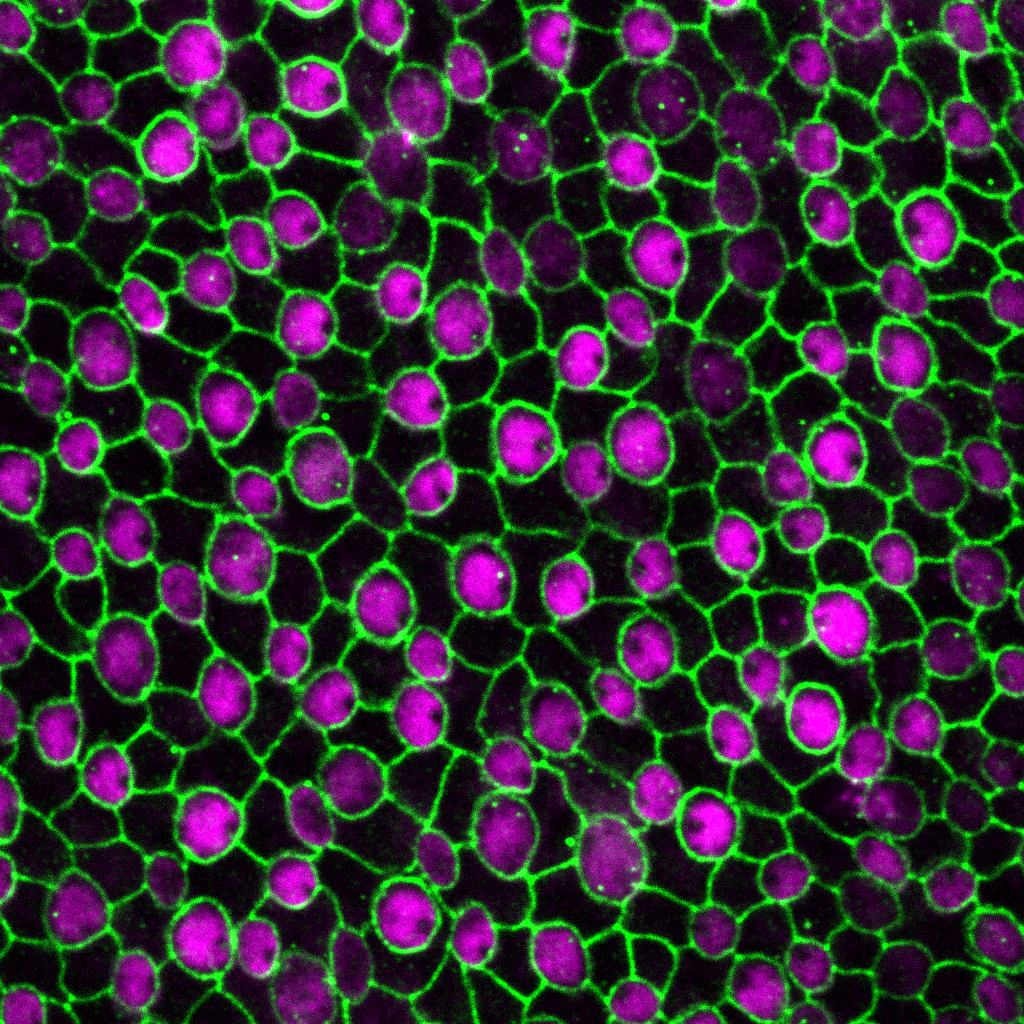

Supplement: Supplementary file 3 — Source Data for Expanded View [file EMMM-15-e17611-s013.zip › Figure EV5/EV5B/P14 utricle Merge.tif]

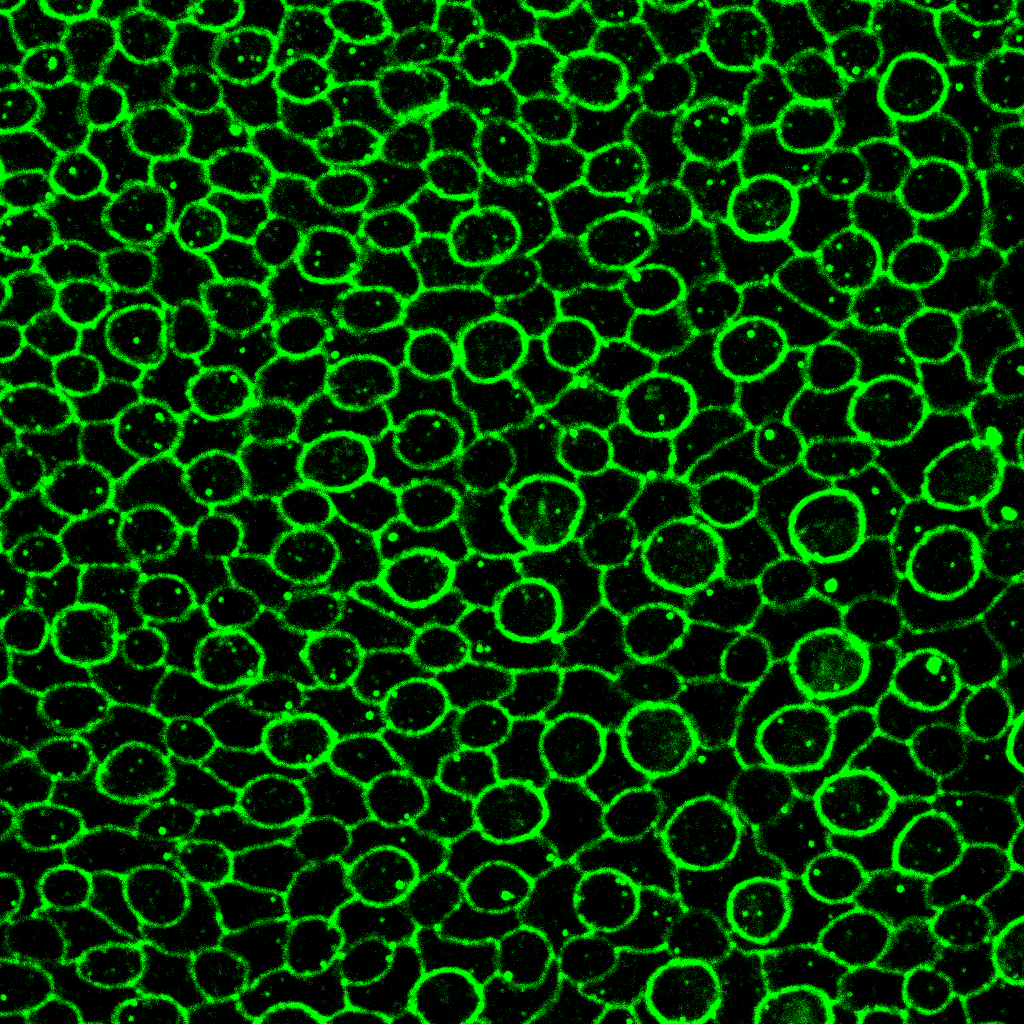

Supplement: Supplementary file 3 — Source Data for Expanded View [file EMMM-15-e17611-s013.zip › Figure EV5/EV5B/P21 utricle CGN.tif]

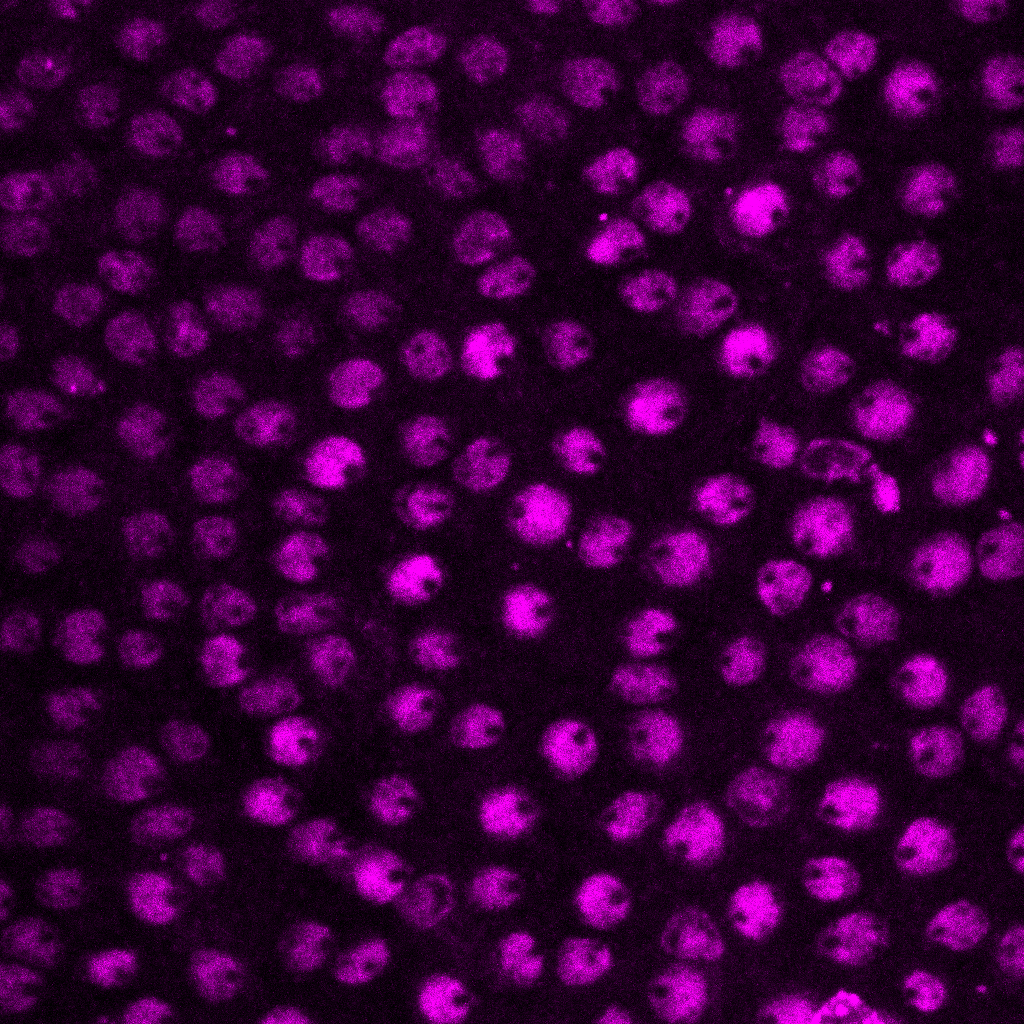

Supplement: Supplementary file 3 — Source Data for Expanded View [file EMMM-15-e17611-s013.zip › Figure EV5/EV5B/P21 utricle LMO7.tif]

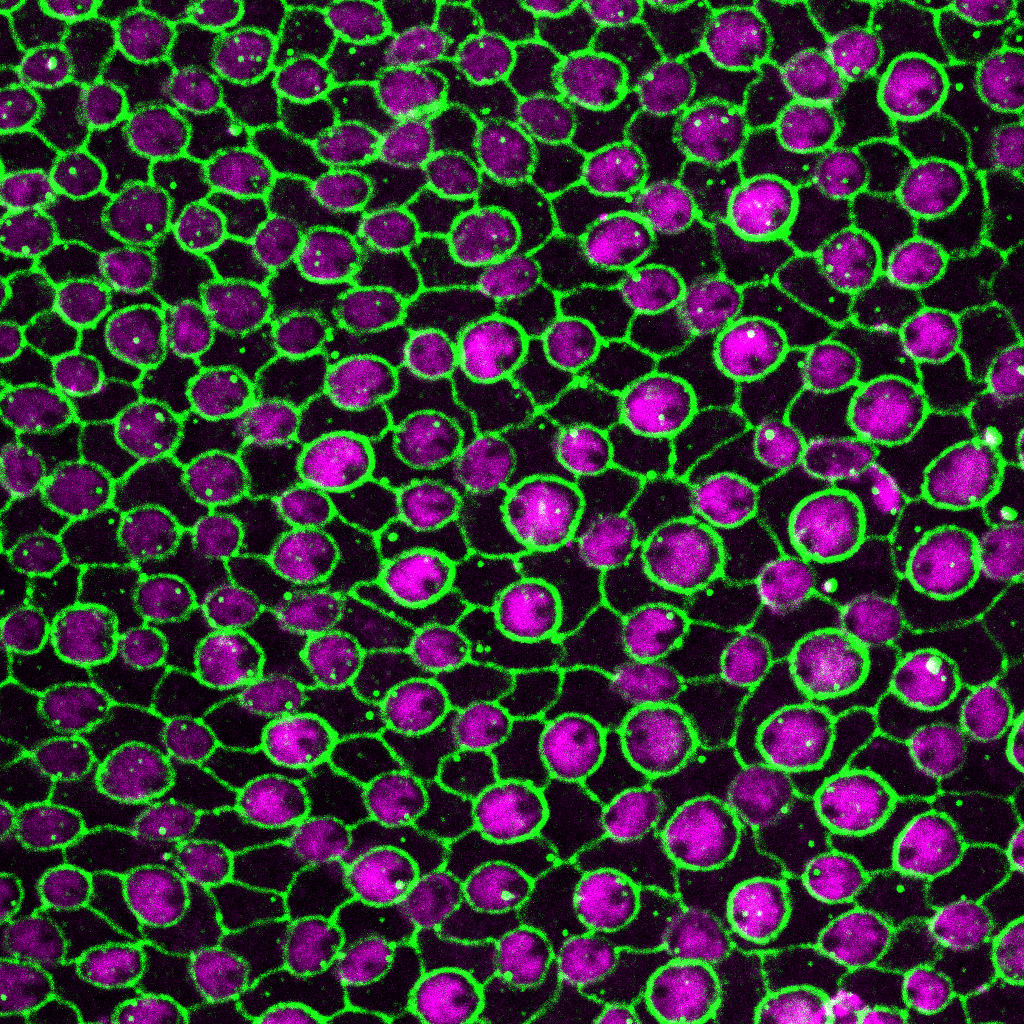

Supplement: Supplementary file 3 — Source Data for Expanded View [file EMMM-15-e17611-s013.zip › Figure EV5/EV5B/P21 utricle Merge.tif]

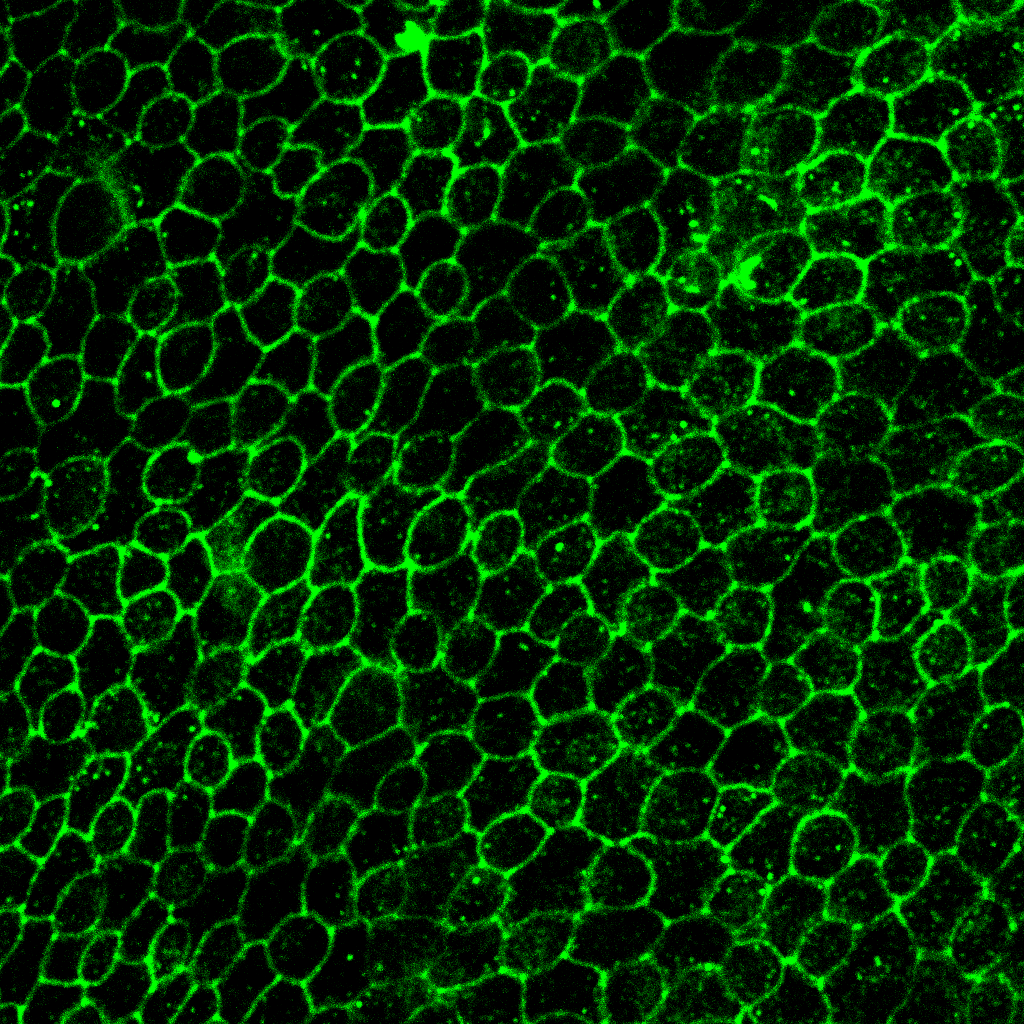

Supplement: Supplementary file 3 — Source Data for Expanded View [file EMMM-15-e17611-s013.zip › Figure EV5/EV5B/P3 utricle CGN.tif]

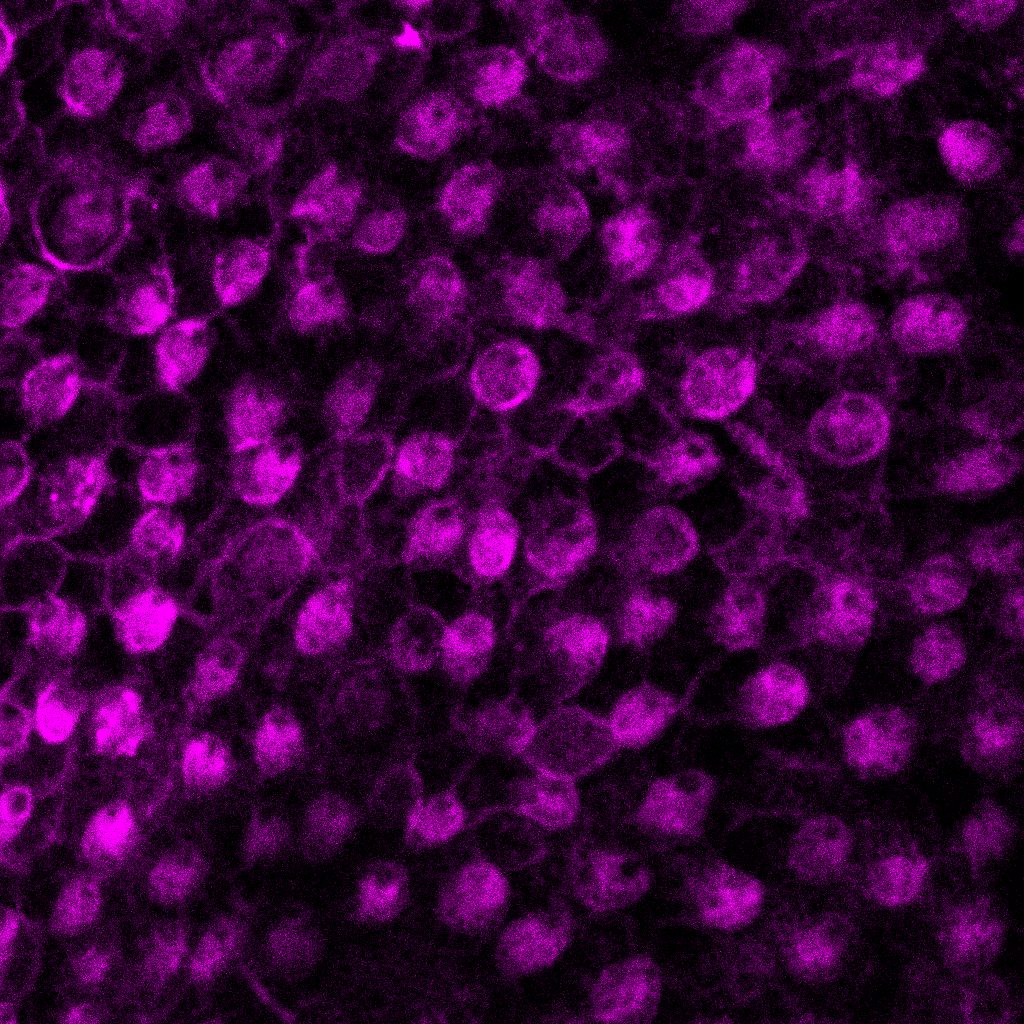

Supplement: Supplementary file 3 — Source Data for Expanded View [file EMMM-15-e17611-s013.zip › Figure EV5/EV5B/P3 utricle LMO7.tif]

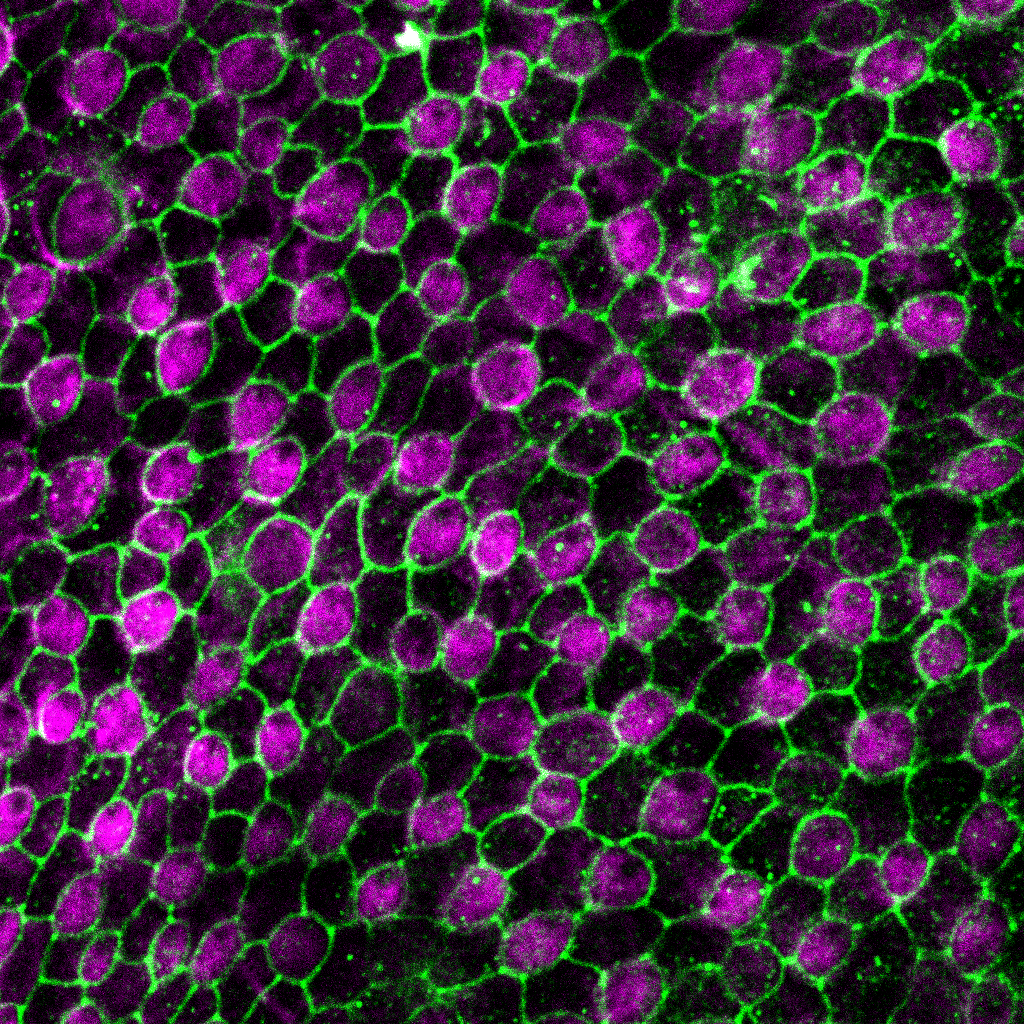

Supplement: Supplementary file 3 — Source Data for Expanded View [file EMMM-15-e17611-s013.zip › Figure EV5/EV5B/P3 utricle Merge.tif]

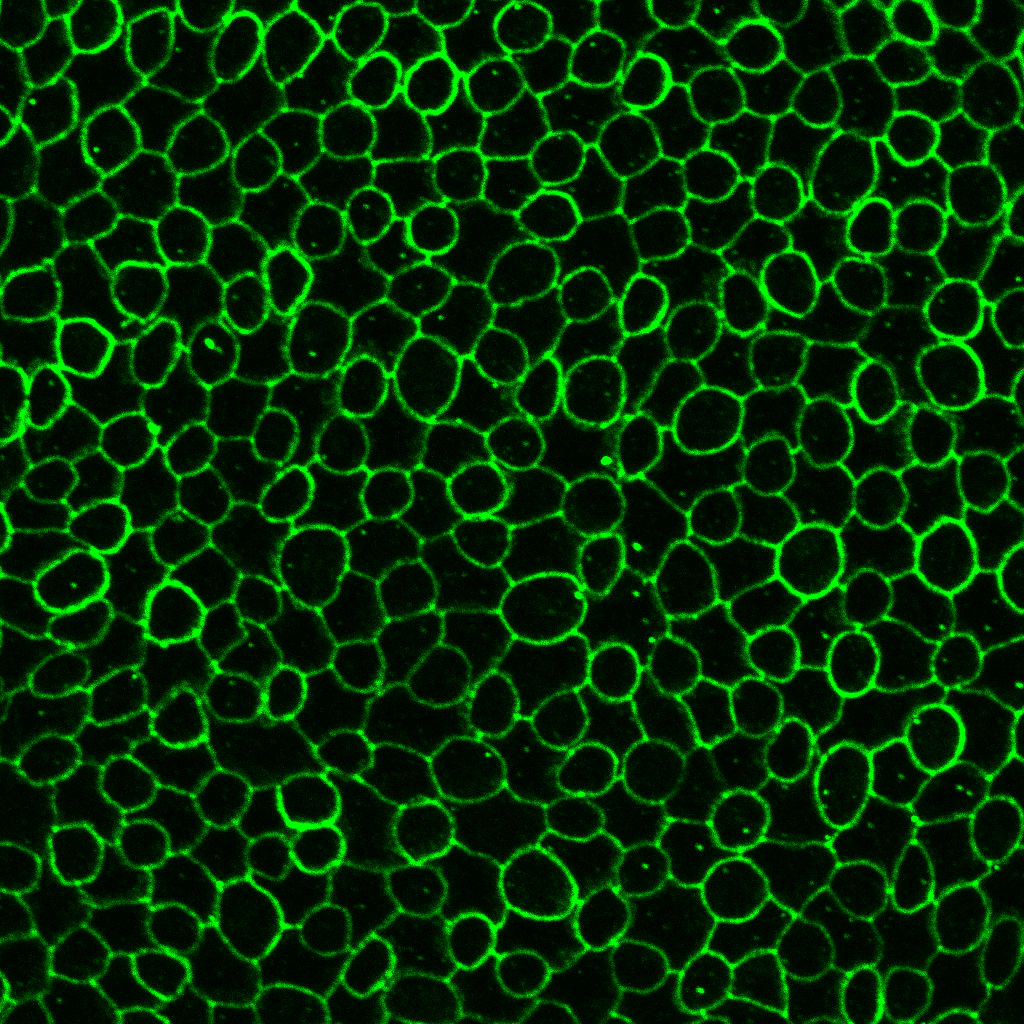

Supplement: Supplementary file 3 — Source Data for Expanded View [file EMMM-15-e17611-s013.zip › Figure EV5/EV5C/HE utricle CGN.tif]

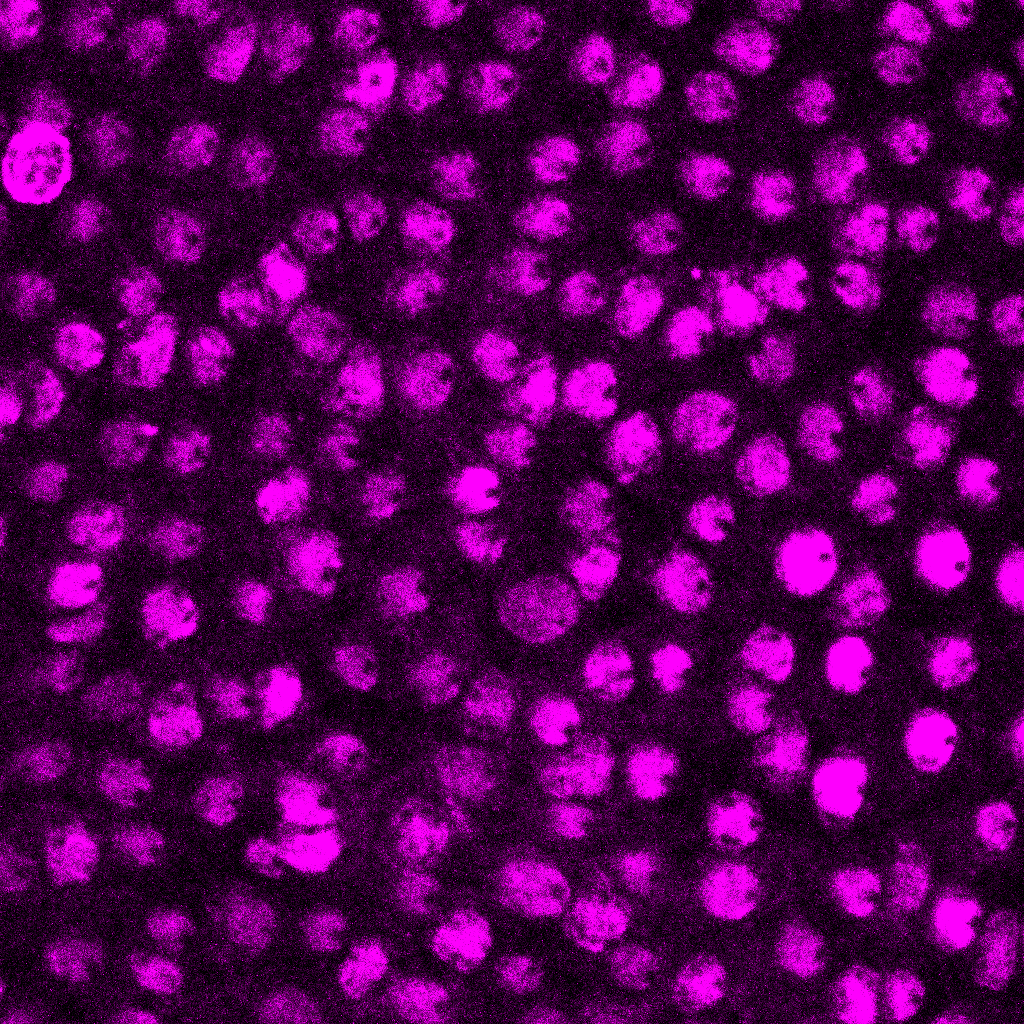

Supplement: Supplementary file 3 — Source Data for Expanded View [file EMMM-15-e17611-s013.zip › Figure EV5/EV5C/HE utricle LMO7.tif]

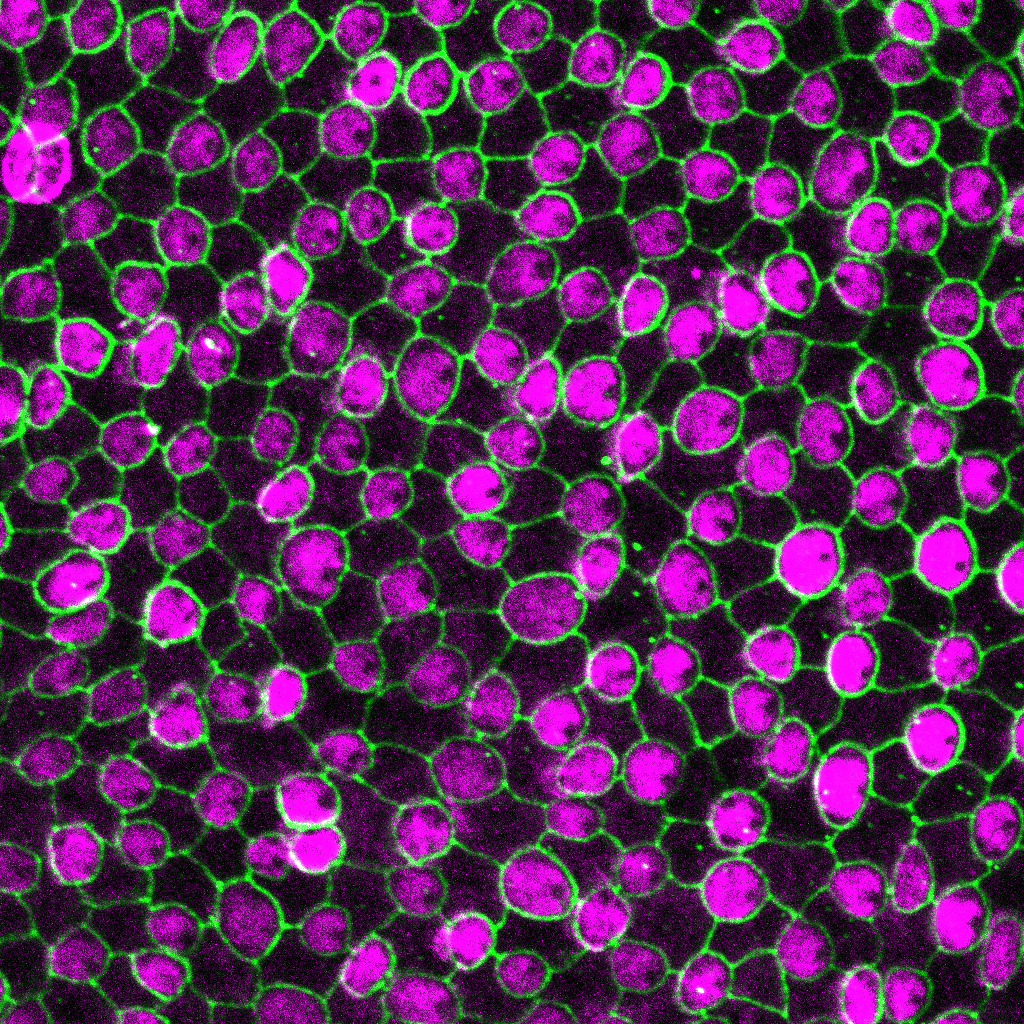

Supplement: Supplementary file 3 — Source Data for Expanded View [file EMMM-15-e17611-s013.zip › Figure EV5/EV5C/HE utricle Merge.tif]

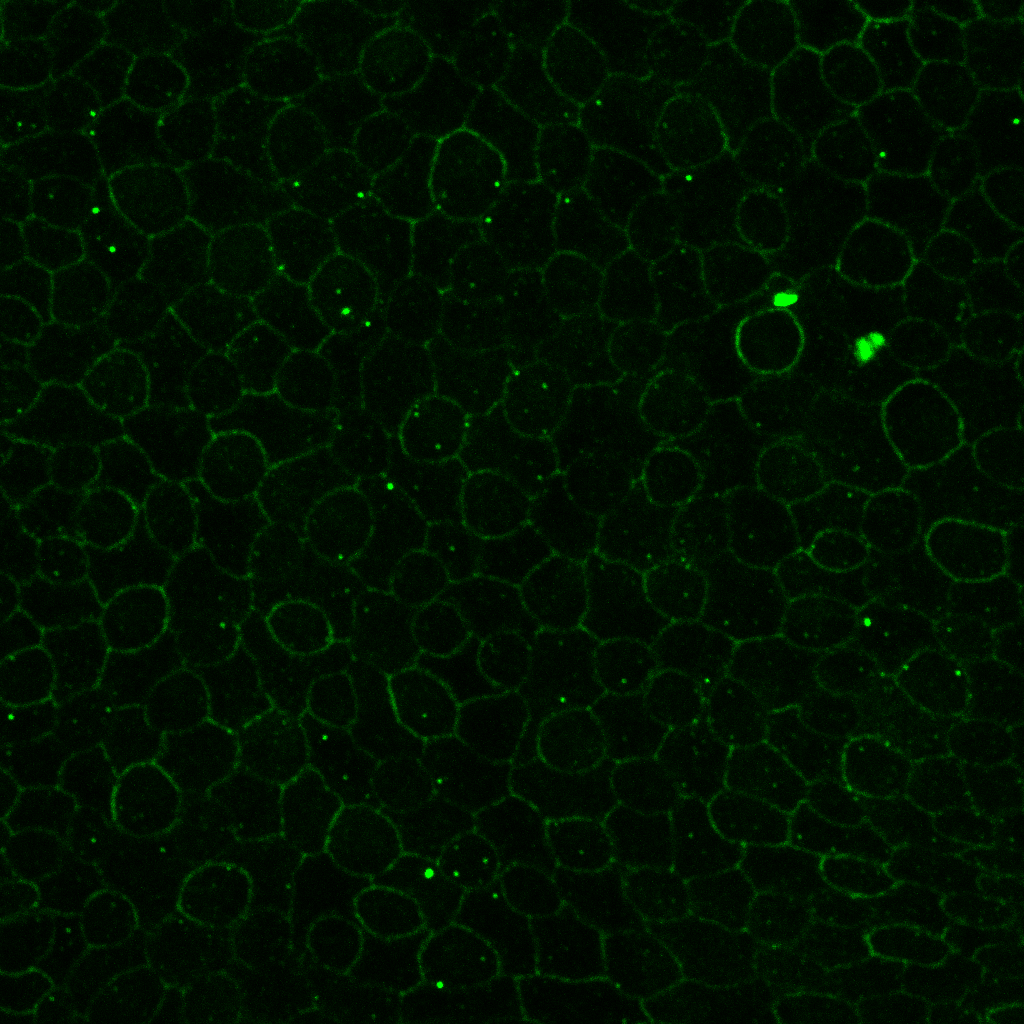

Supplement: Supplementary file 3 — Source Data for Expanded View [file EMMM-15-e17611-s013.zip › Figure EV5/EV5C/HO utricle CGN.tif]

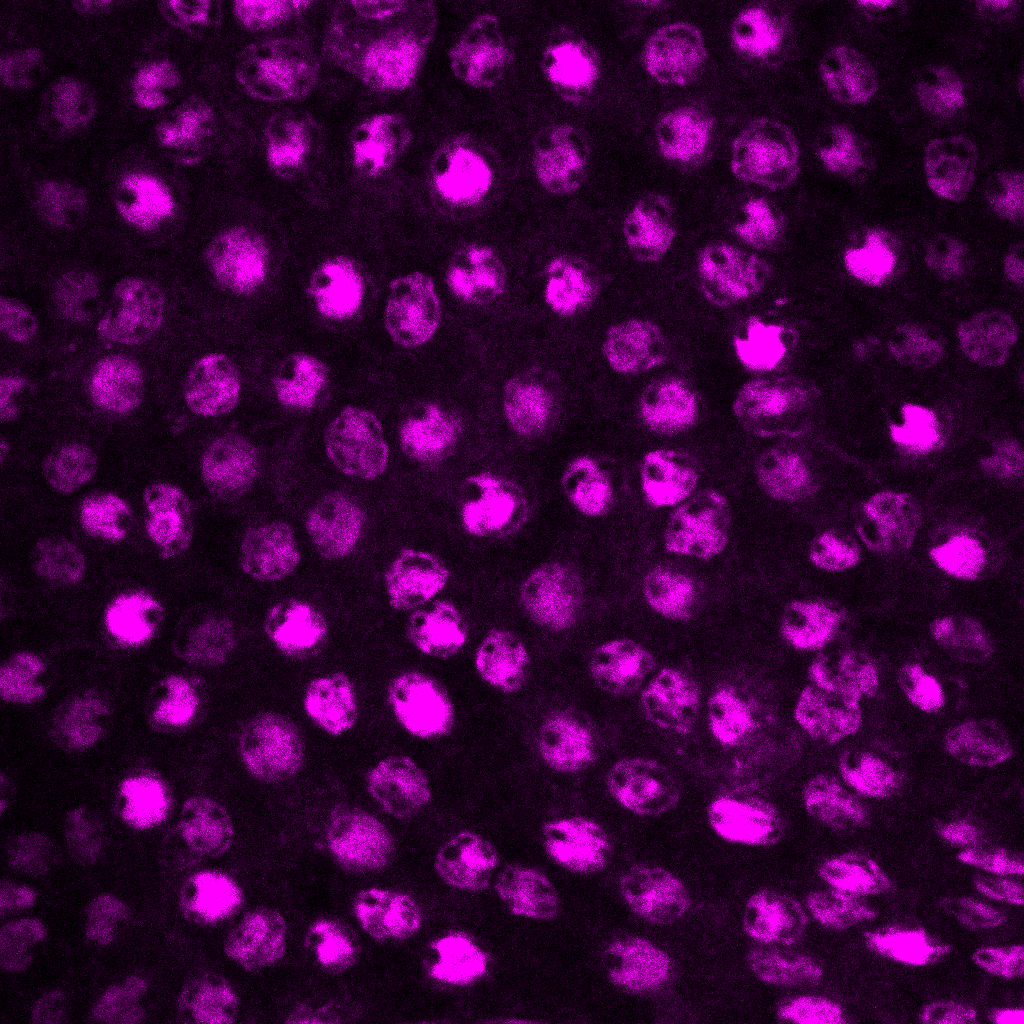

Supplement: Supplementary file 3 — Source Data for Expanded View [file EMMM-15-e17611-s013.zip › Figure EV5/EV5C/HO utricle LMO7.tif]

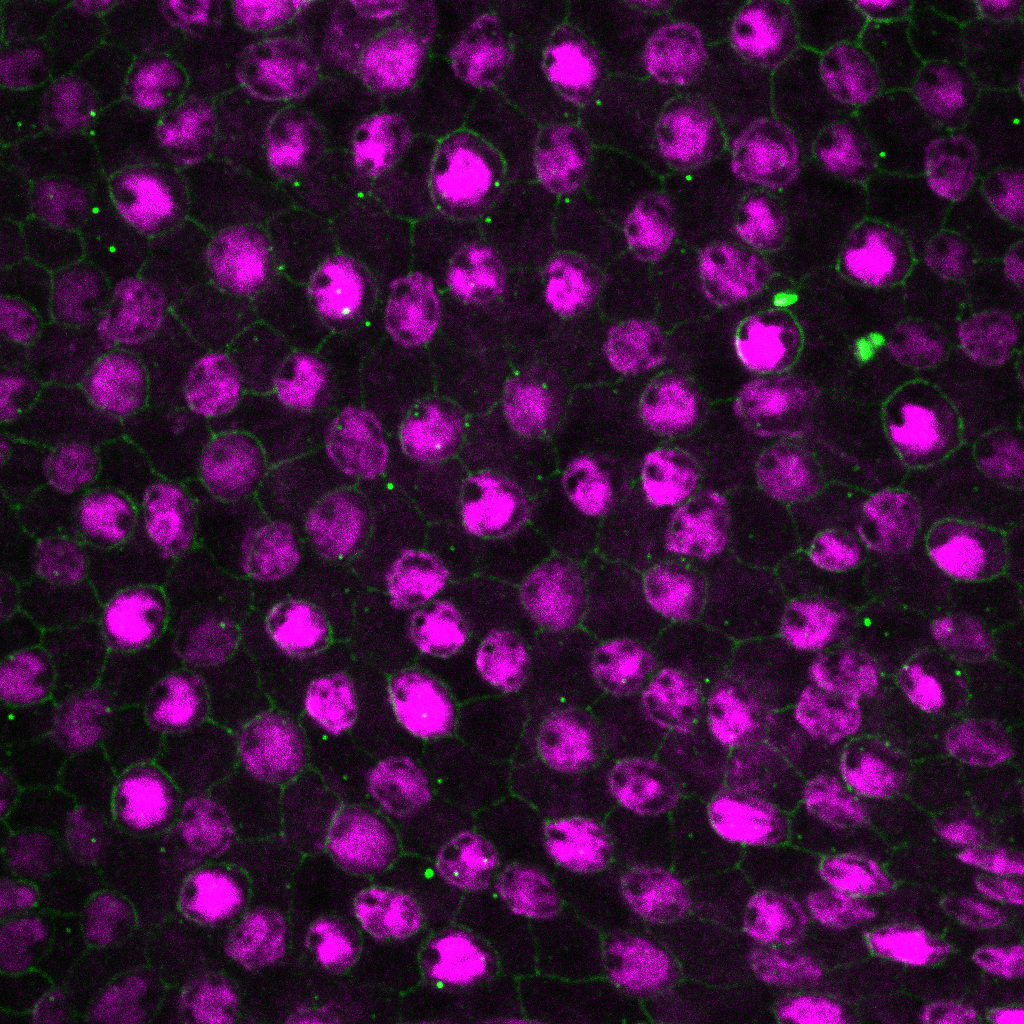

Supplement: Supplementary file 3 — Source Data for Expanded View [file EMMM-15-e17611-s013.zip › Figure EV5/EV5C/HO utricle Merge.tif]

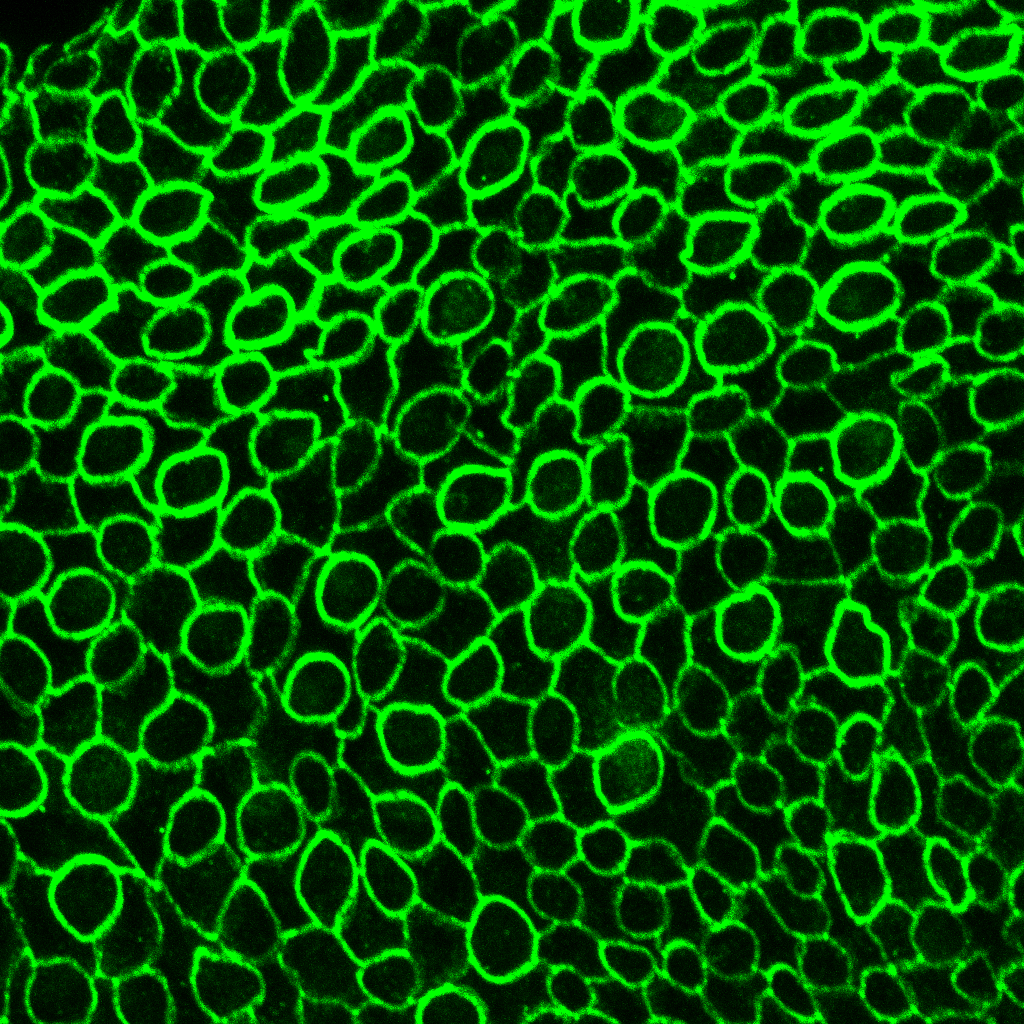

Supplement: Supplementary file 3 — Source Data for Expanded View [file EMMM-15-e17611-s013.zip › Figure EV5/EV5C/WT utricle CGN.tif]

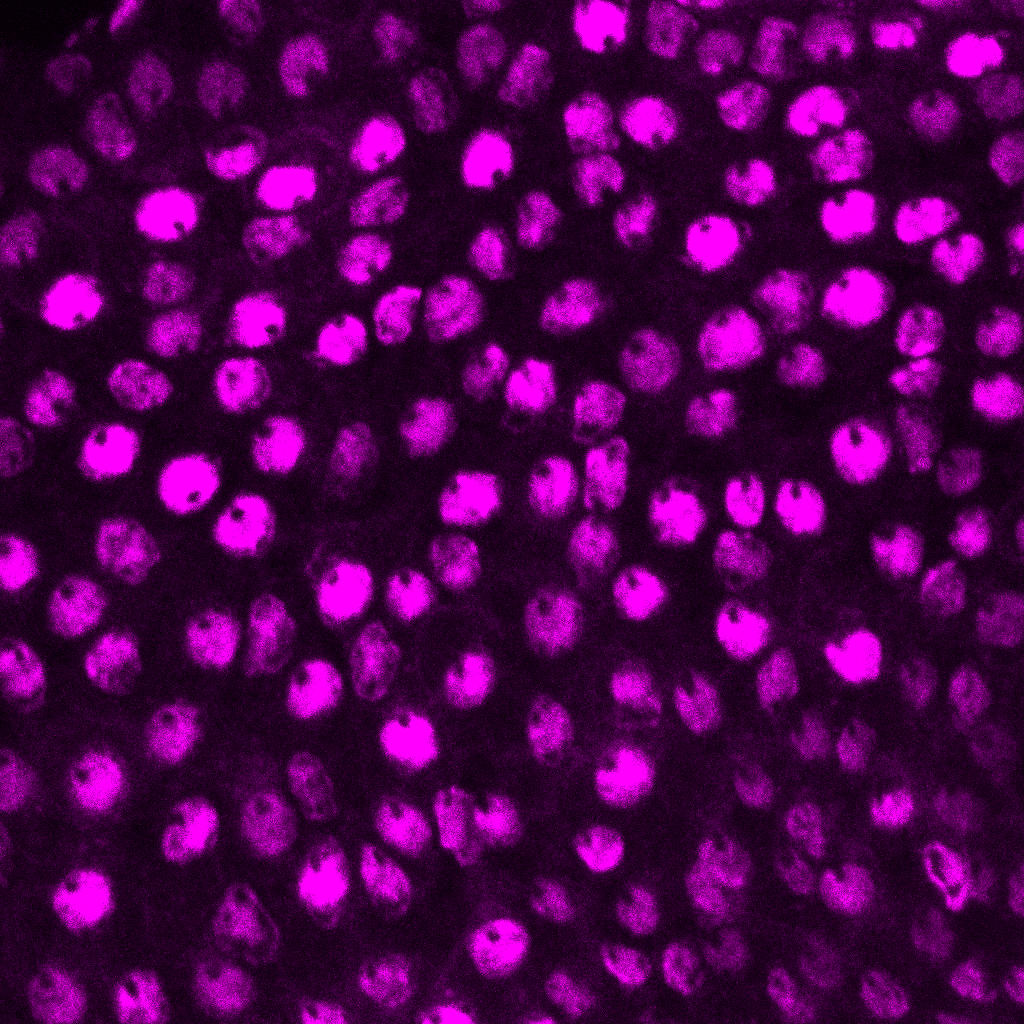

Supplement: Supplementary file 3 — Source Data for Expanded View [file EMMM-15-e17611-s013.zip › Figure EV5/EV5C/WT utricle LMO7.tif]

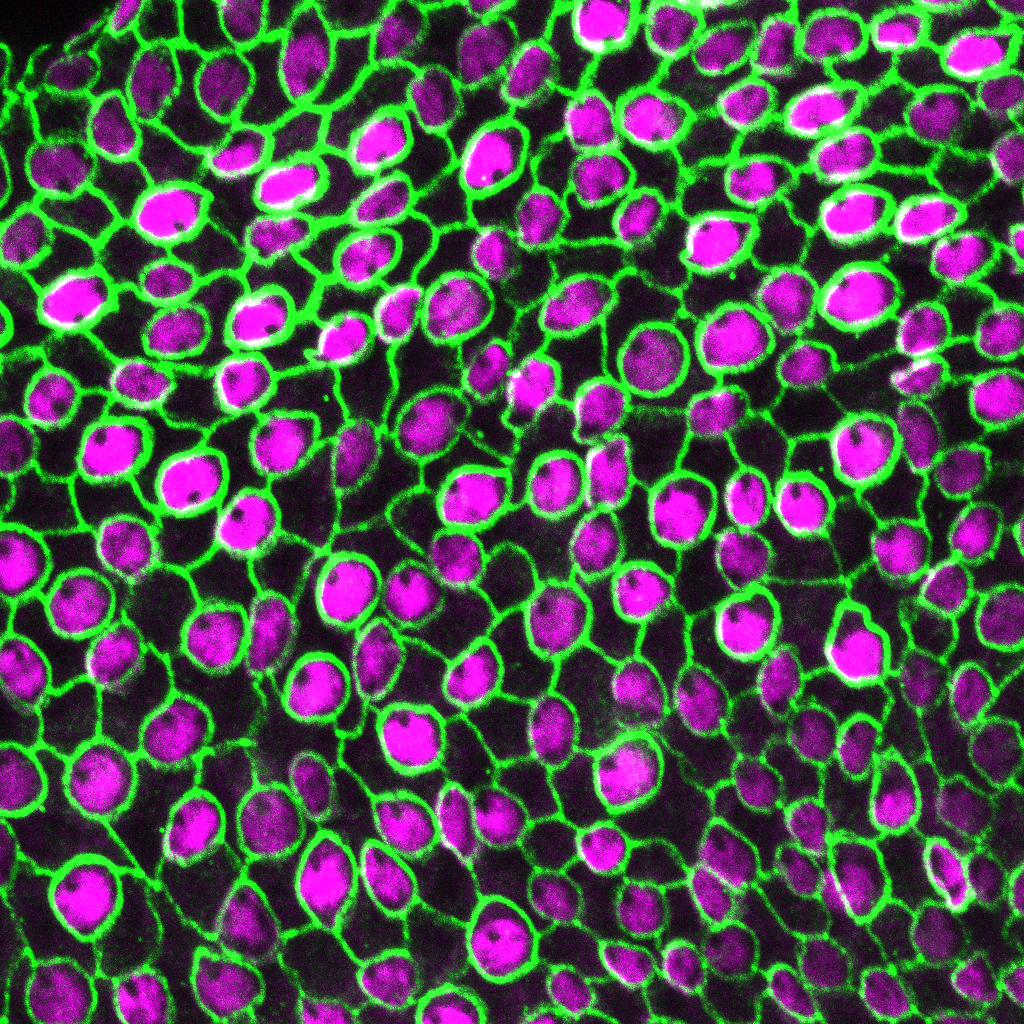

Supplement: Supplementary file 3 — Source Data for Expanded View [file EMMM-15-e17611-s013.zip › Figure EV5/EV5C/WT utricle Merge.tif]

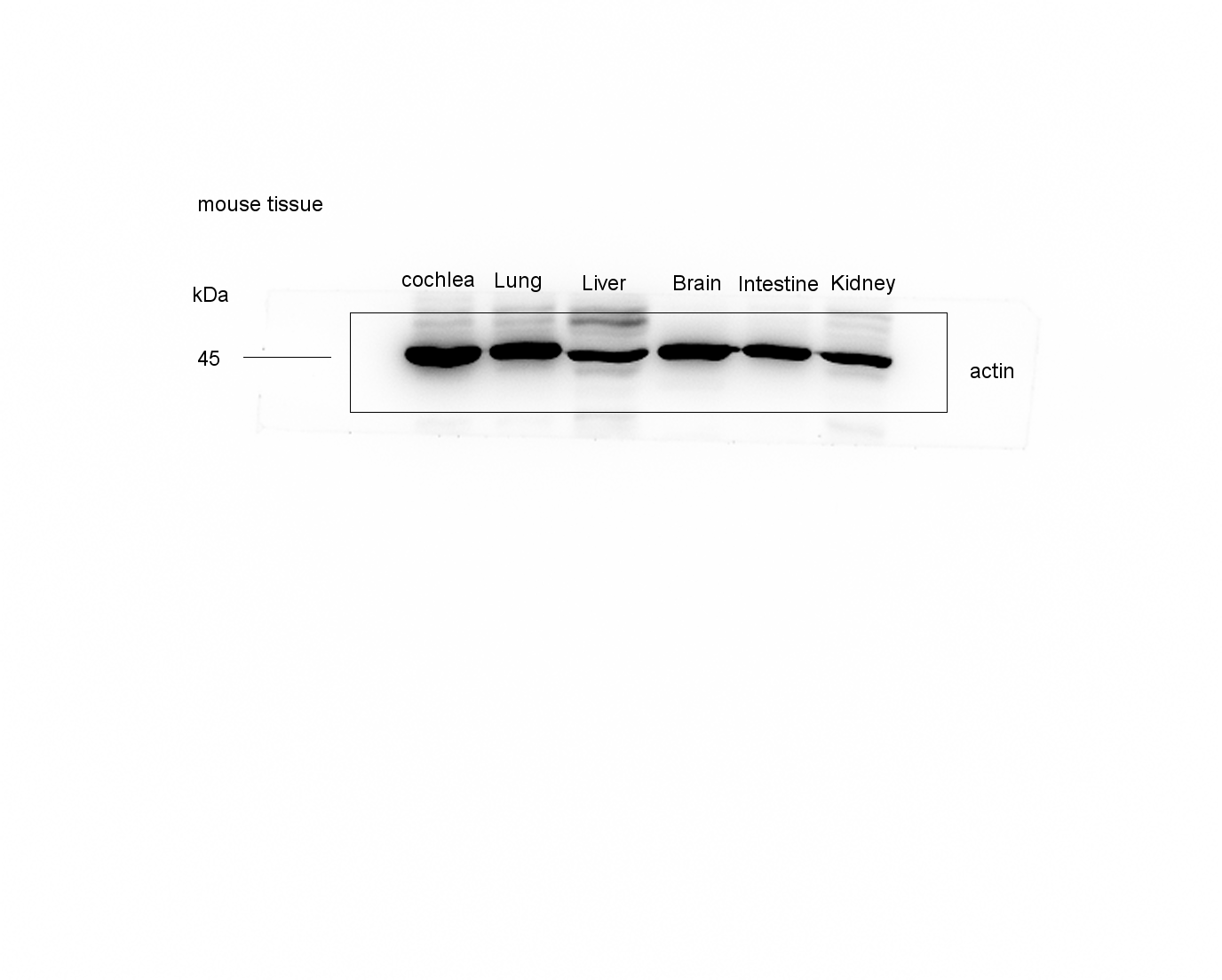

Supplement: Supplementary file 6 — Source Data for Figure 2 [file EMMM-15-e17611-s001.zip › Figure 2/2B/western actin.tif]

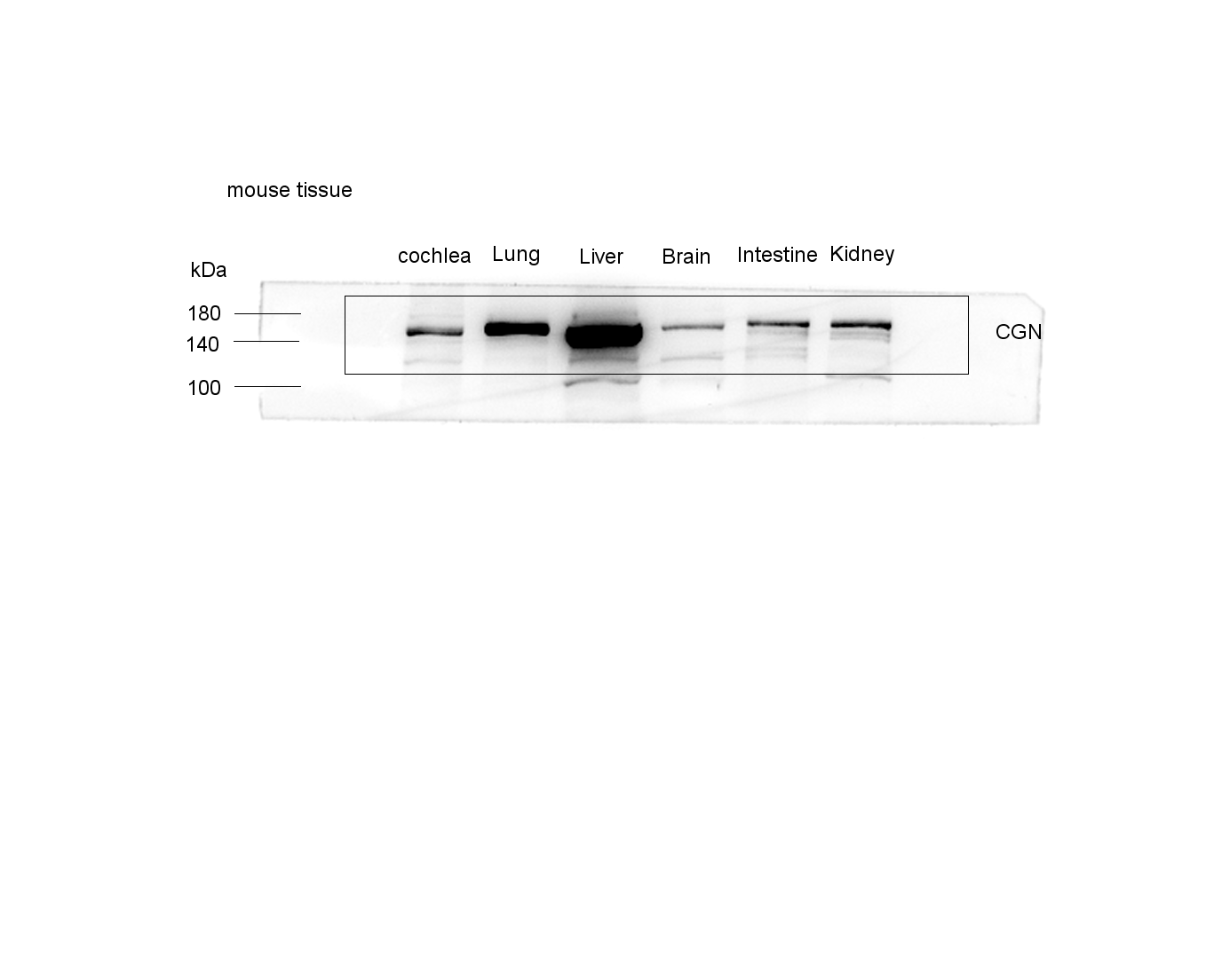

Supplement: Supplementary file 6 — Source Data for Figure 2 [file EMMM-15-e17611-s001.zip › Figure 2/2B/western Cgn.tif]

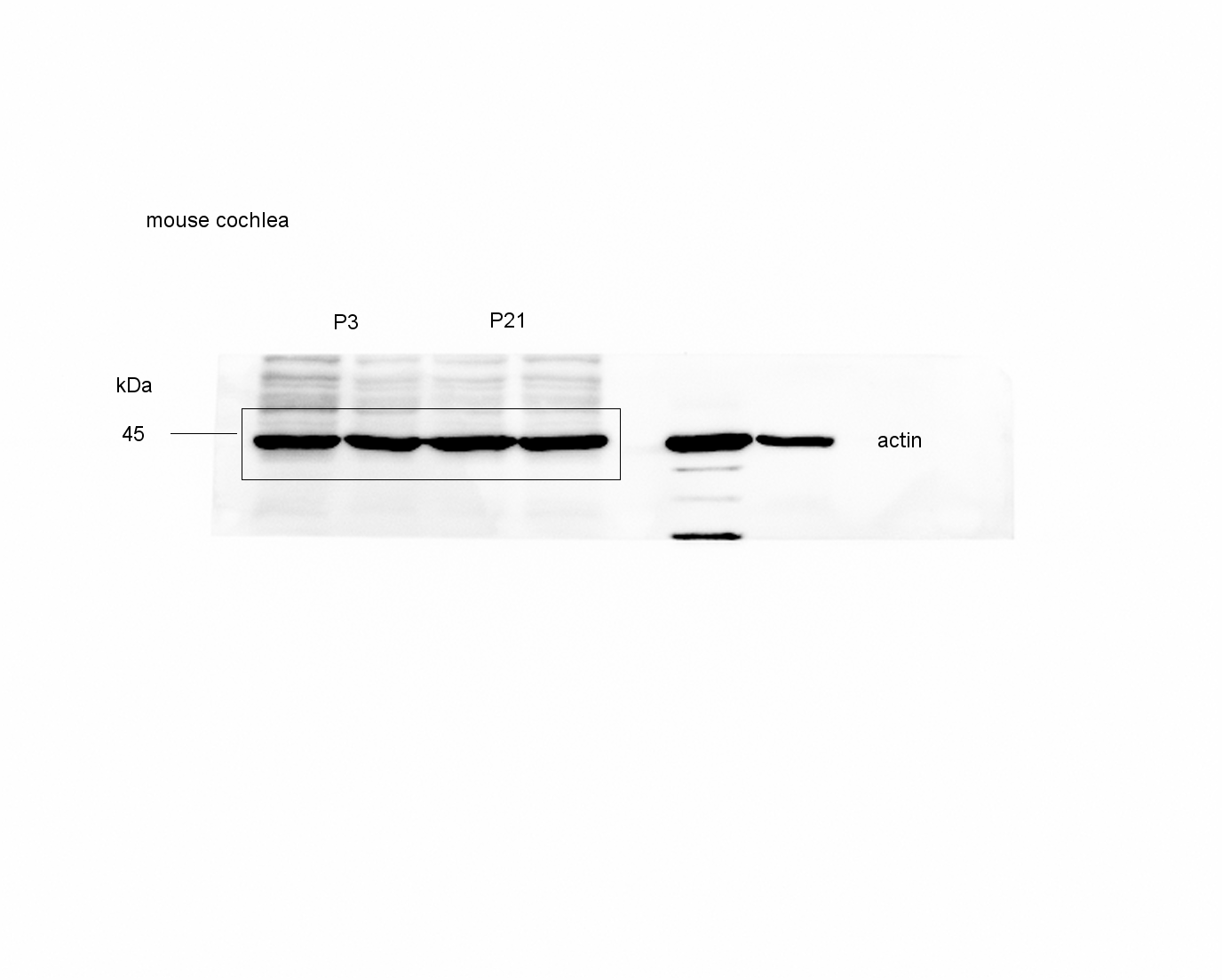

Supplement: Supplementary file 6 — Source Data for Figure 2 [file EMMM-15-e17611-s001.zip › Figure 2/2D/western actin.tif]

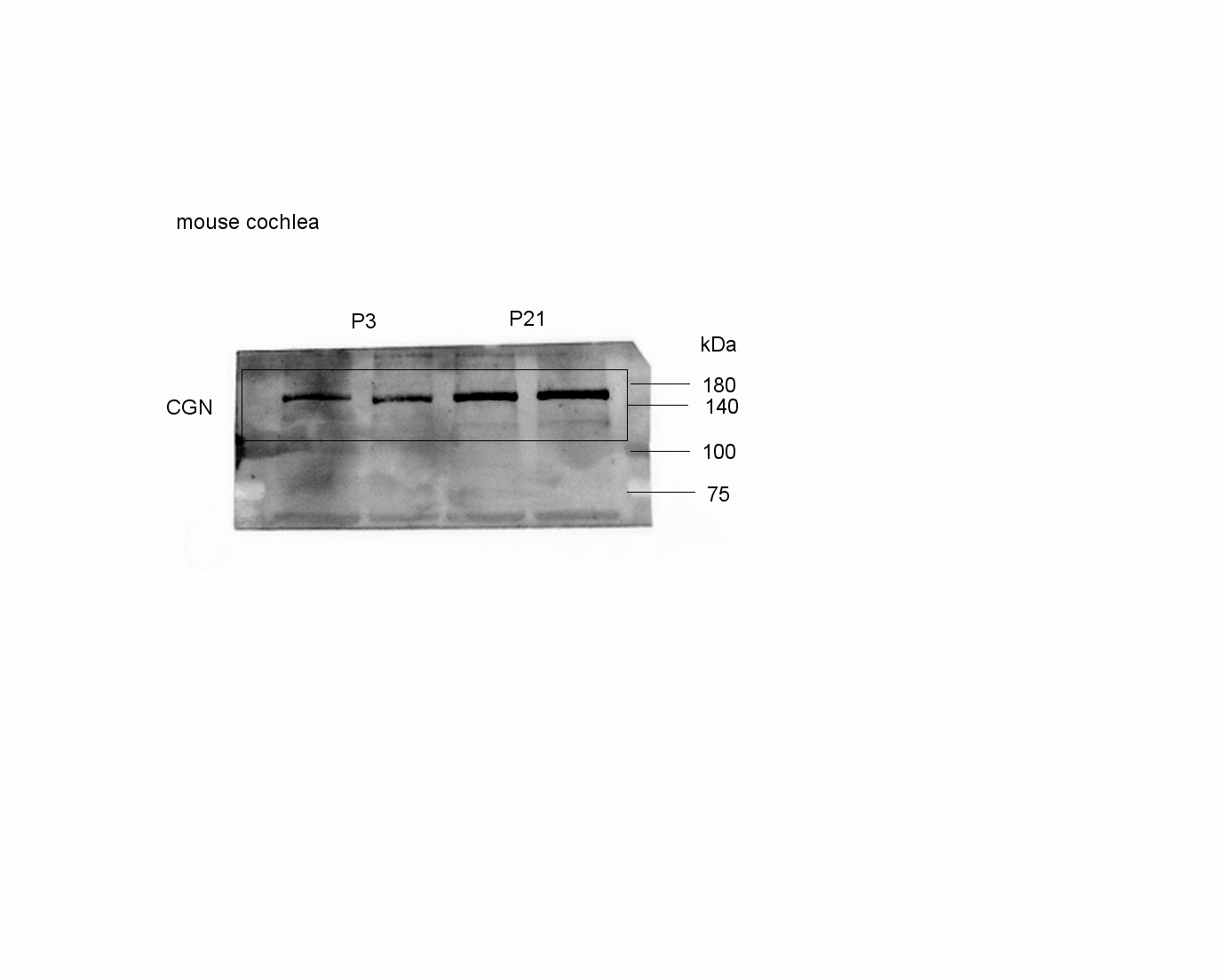

Supplement: Supplementary file 6 — Source Data for Figure 2 [file EMMM-15-e17611-s001.zip › Figure 2/2D/western CGN.tif]

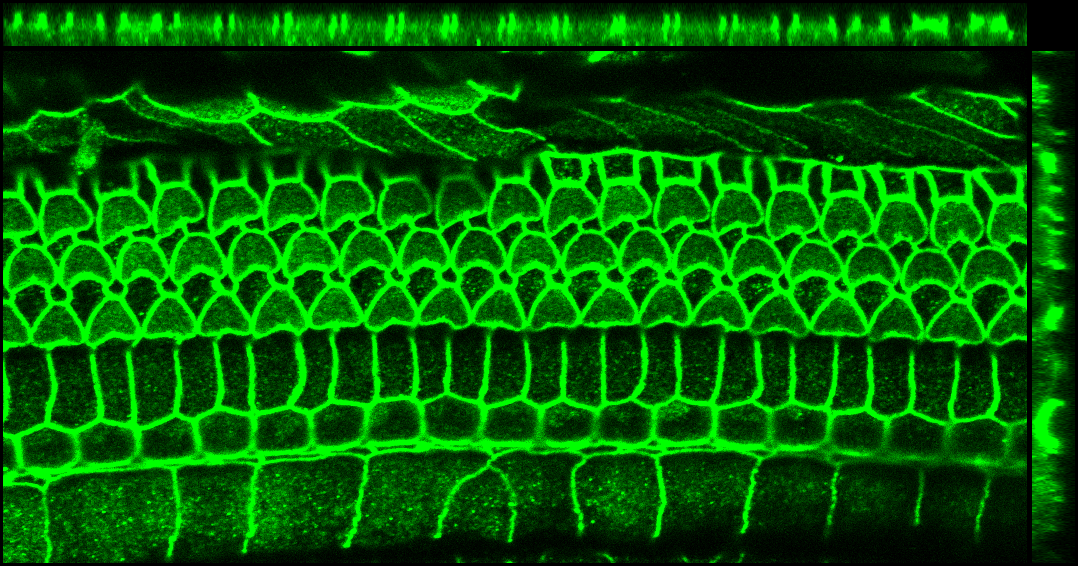

Supplement: Supplementary file 6 — Source Data for Figure 2 [file EMMM-15-e17611-s001.zip › Figure 2/2F-G/Whole mount immunofluorescence of Cgn expression LMO7/P14 cochlea CGN.tif]

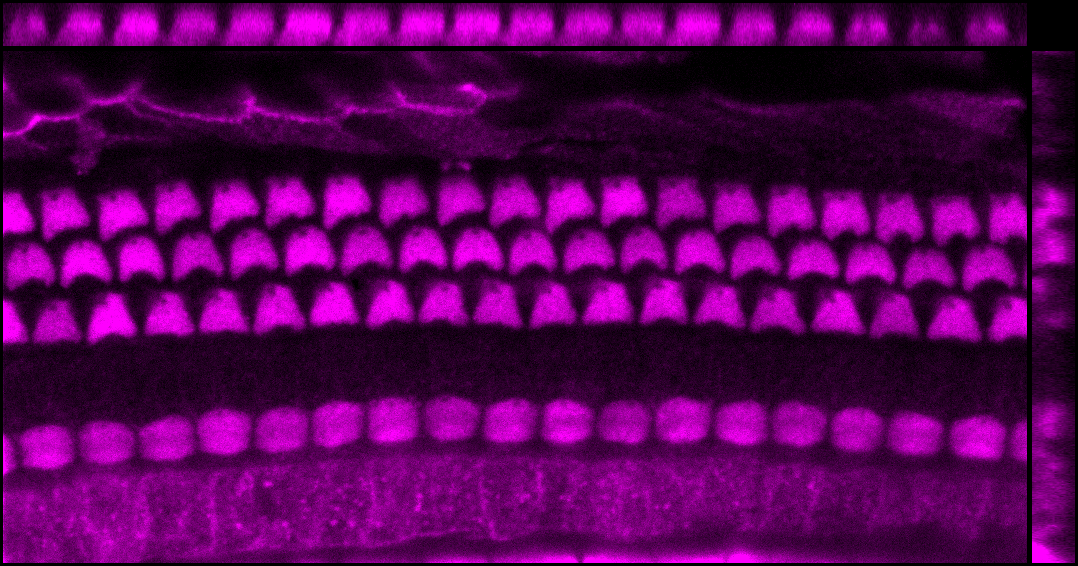

Supplement: Supplementary file 6 — Source Data for Figure 2 [file EMMM-15-e17611-s001.zip › Figure 2/2F-G/Whole mount immunofluorescence of Cgn expression LMO7/P14 cochlea LMO7.tif]

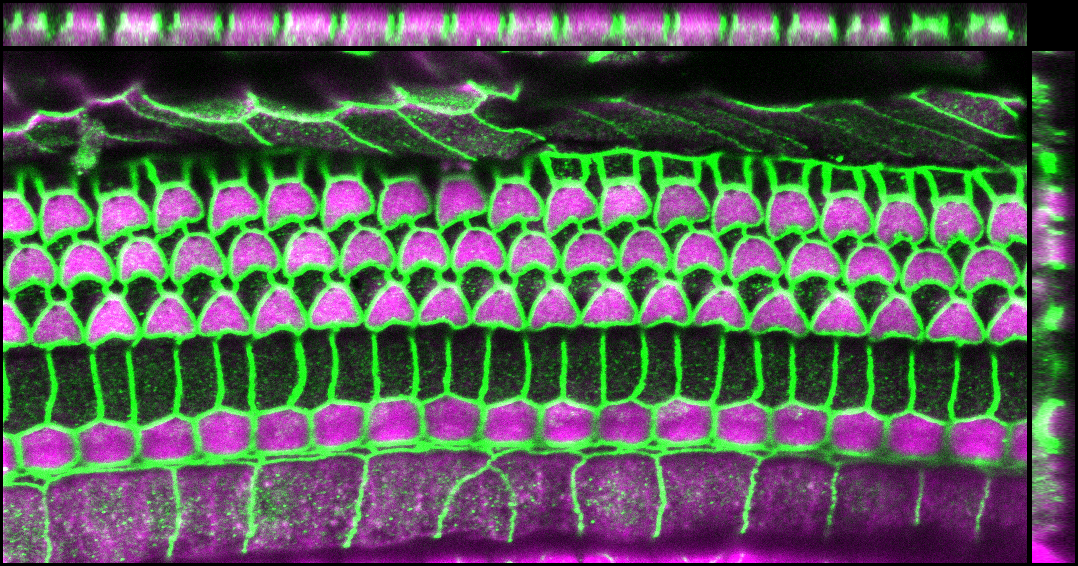

Supplement: Supplementary file 6 — Source Data for Figure 2 [file EMMM-15-e17611-s001.zip › Figure 2/2F-G/Whole mount immunofluorescence of Cgn expression LMO7/P14 cochlea Merge.tif]

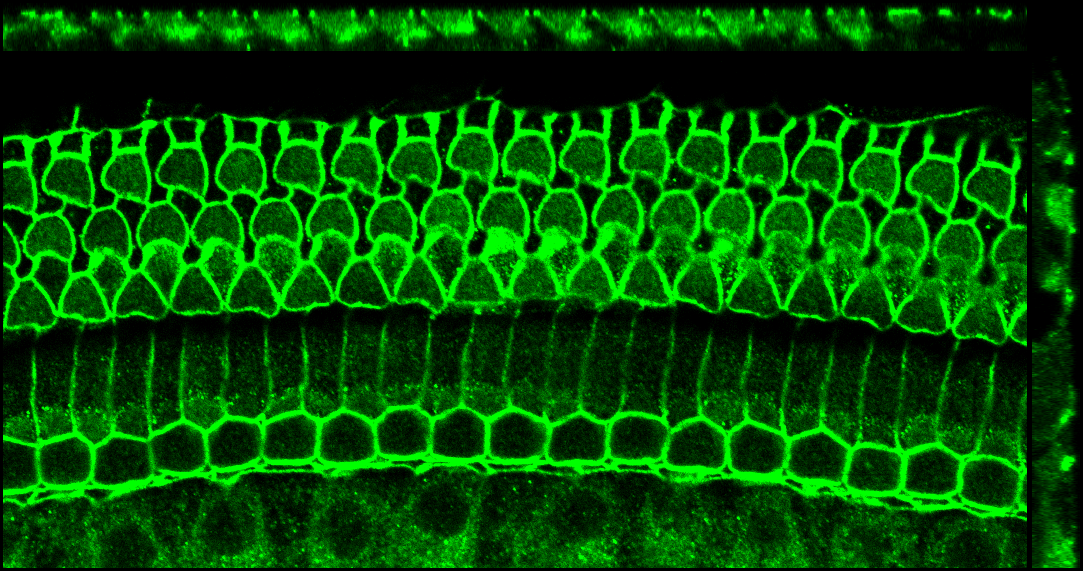

Supplement: Supplementary file 6 — Source Data for Figure 2 [file EMMM-15-e17611-s001.zip › Figure 2/2F-G/Whole mount immunofluorescence of Cgn expression LMO7/P21 cochlea CGN.tif]

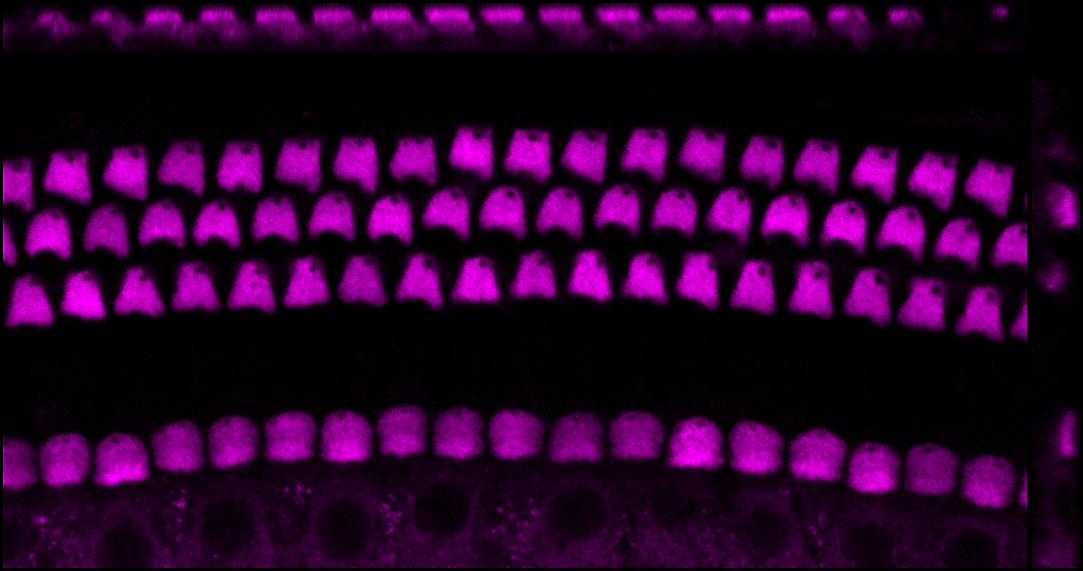

Supplement: Supplementary file 6 — Source Data for Figure 2 [file EMMM-15-e17611-s001.zip › Figure 2/2F-G/Whole mount immunofluorescence of Cgn expression LMO7/P21 cochlea LMO7.tif]

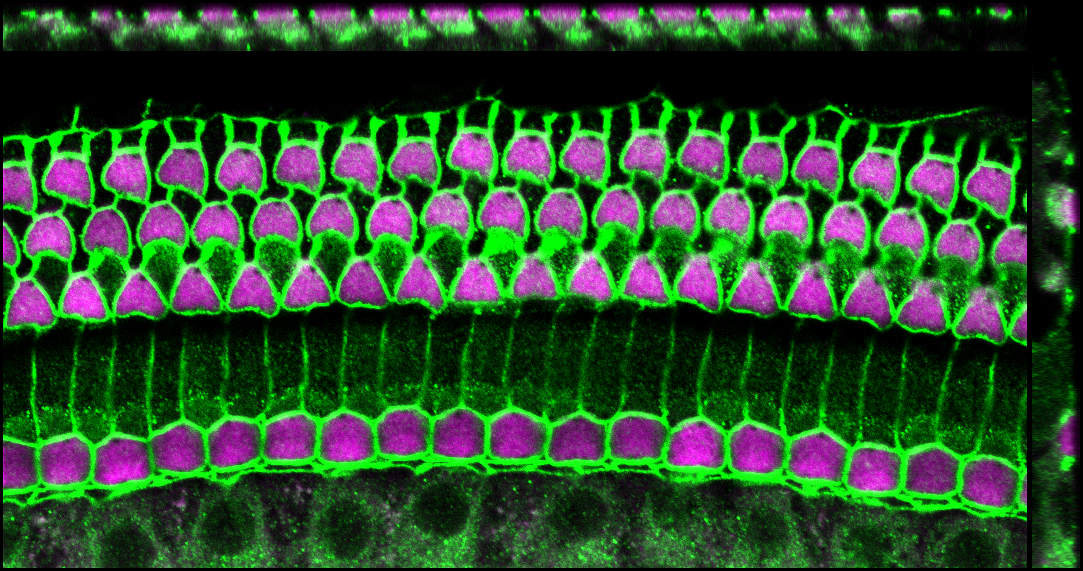

Supplement: Supplementary file 6 — Source Data for Figure 2 [file EMMM-15-e17611-s001.zip › Figure 2/2F-G/Whole mount immunofluorescence of Cgn expression LMO7/P21 cochlea Merge.tif]

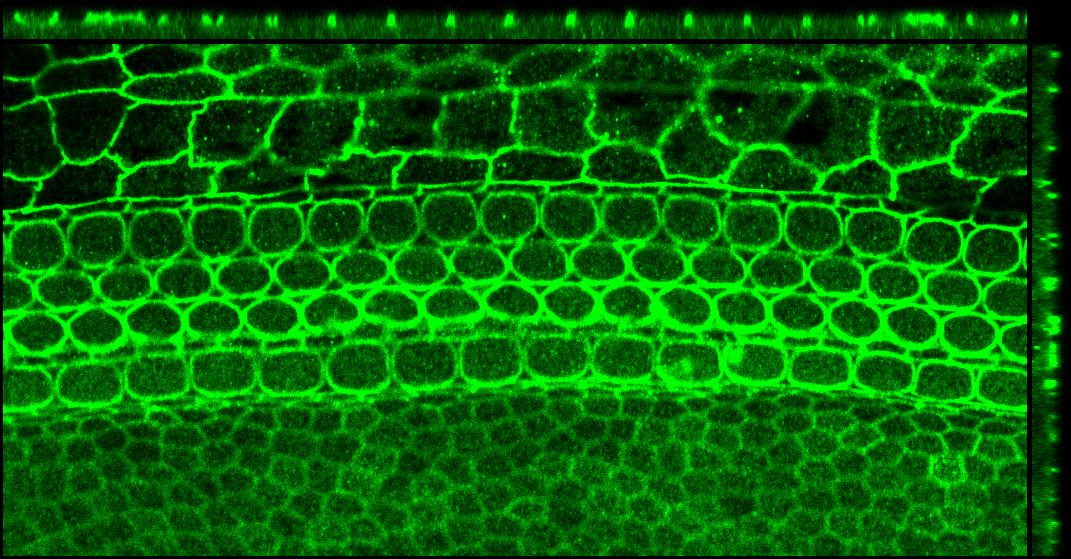

Supplement: Supplementary file 6 — Source Data for Figure 2 [file EMMM-15-e17611-s001.zip › Figure 2/2F-G/Whole mount immunofluorescence of Cgn expression LMO7/P3 cochlea CGN.tif]

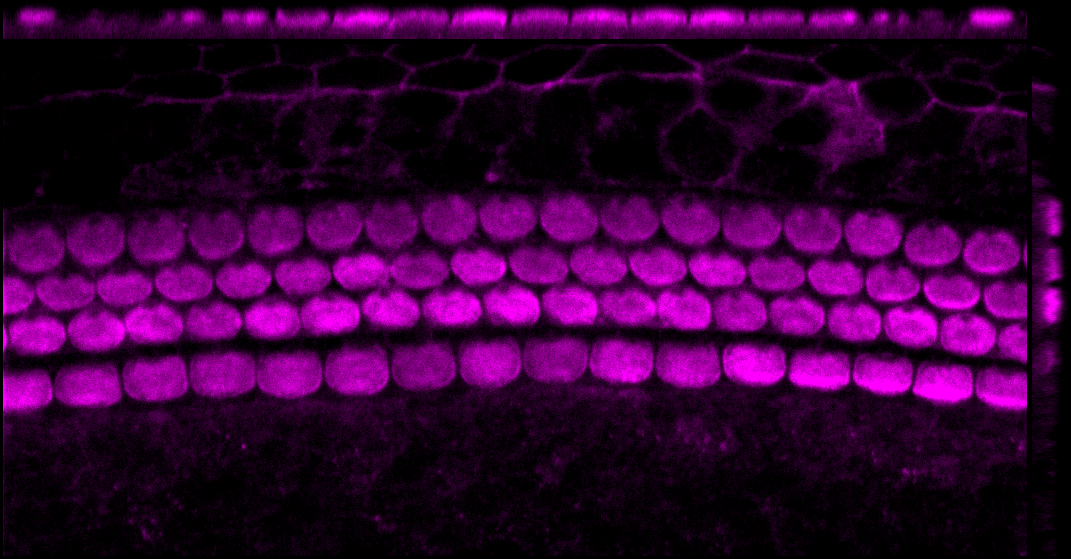

Supplement: Supplementary file 6 — Source Data for Figure 2 [file EMMM-15-e17611-s001.zip › Figure 2/2F-G/Whole mount immunofluorescence of Cgn expression LMO7/P3 cochlea LMO7.tif]

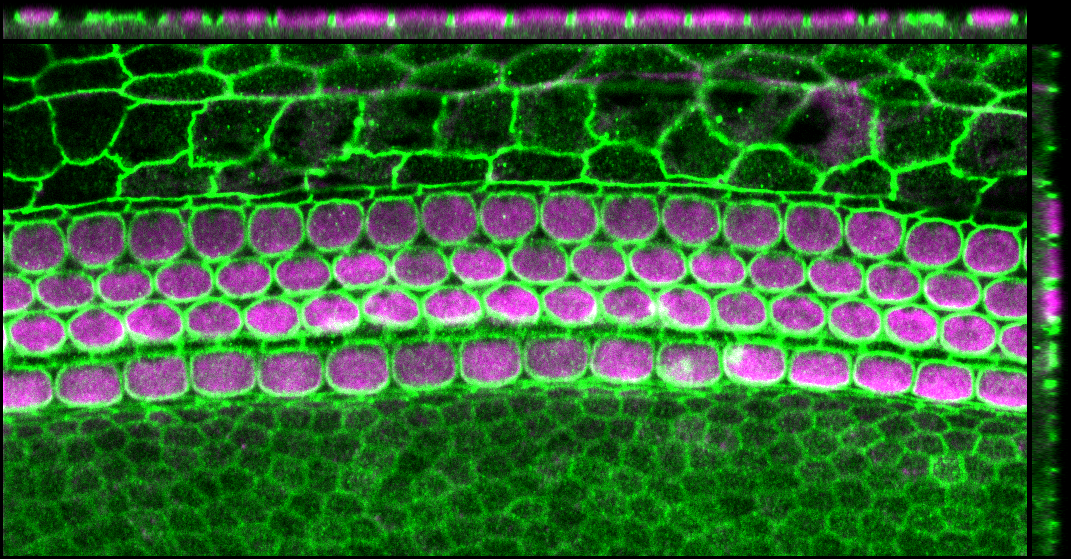

Supplement: Supplementary file 6 — Source Data for Figure 2 [file EMMM-15-e17611-s001.zip › Figure 2/2F-G/Whole mount immunofluorescence of Cgn expression LMO7/P3 cochlea Merge.tif]

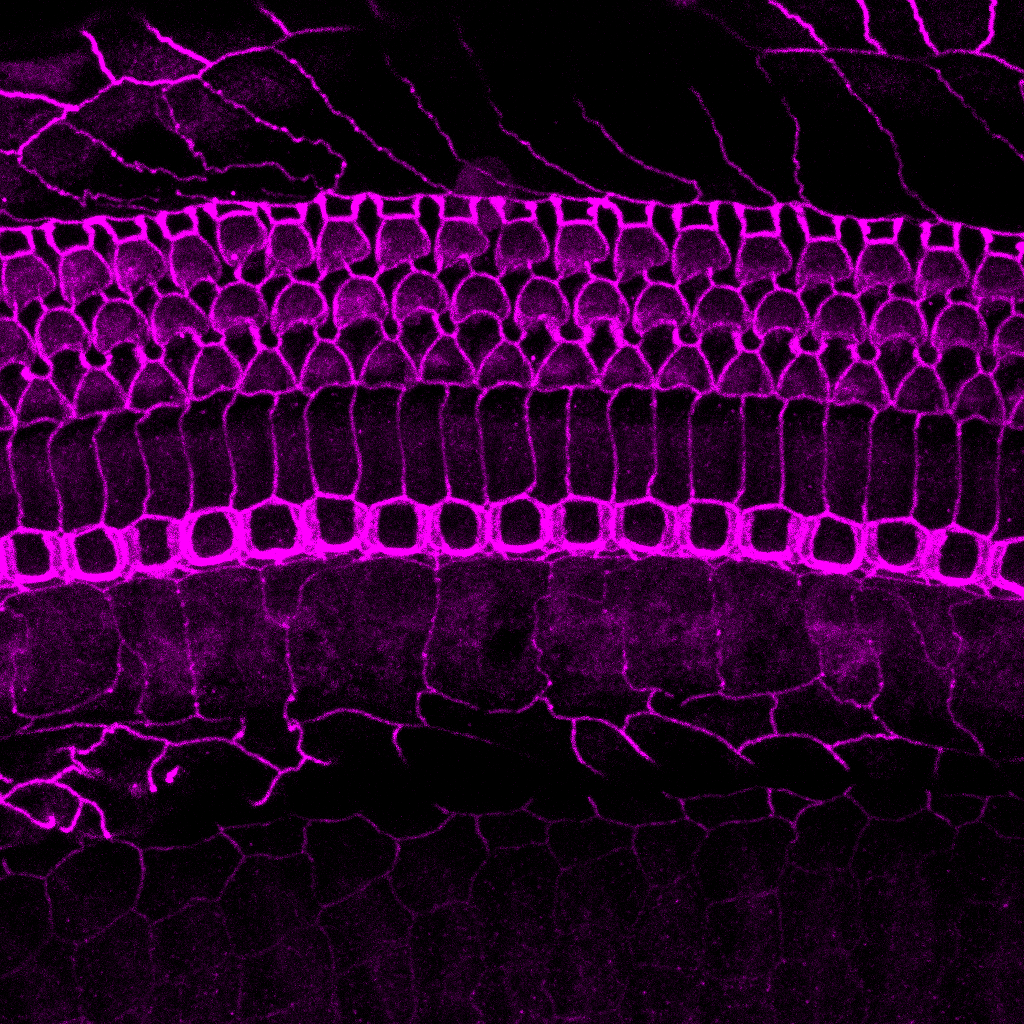

Supplement: Supplementary file 6 — Source Data for Figure 2 [file EMMM-15-e17611-s001.zip › Figure 2/2F-G/Whole mount immunofluorescence of Cgn expression Parvalbumin/P14 cochlea CGN.tif]

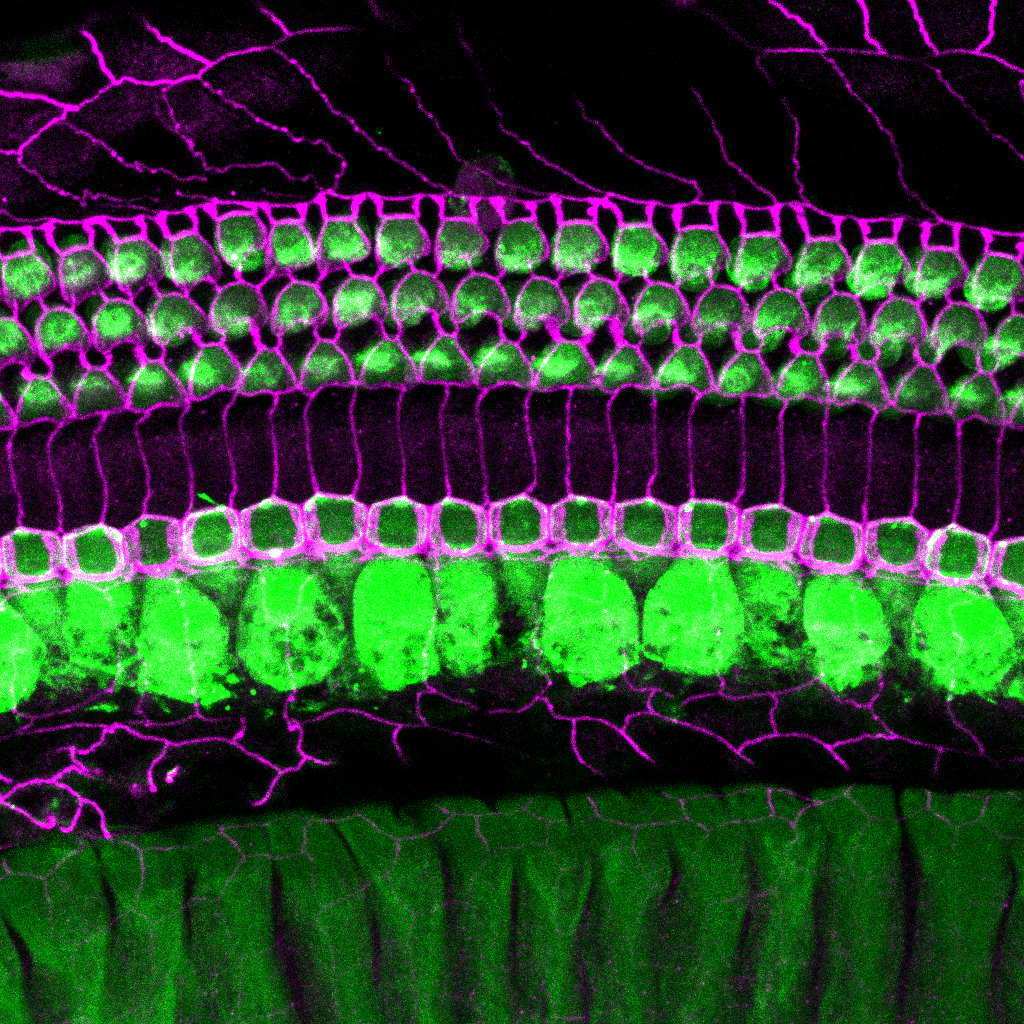

Supplement: Supplementary file 6 — Source Data for Figure 2 [file EMMM-15-e17611-s001.zip › Figure 2/2F-G/Whole mount immunofluorescence of Cgn expression Parvalbumin/P14 cochlea Merge.tif]

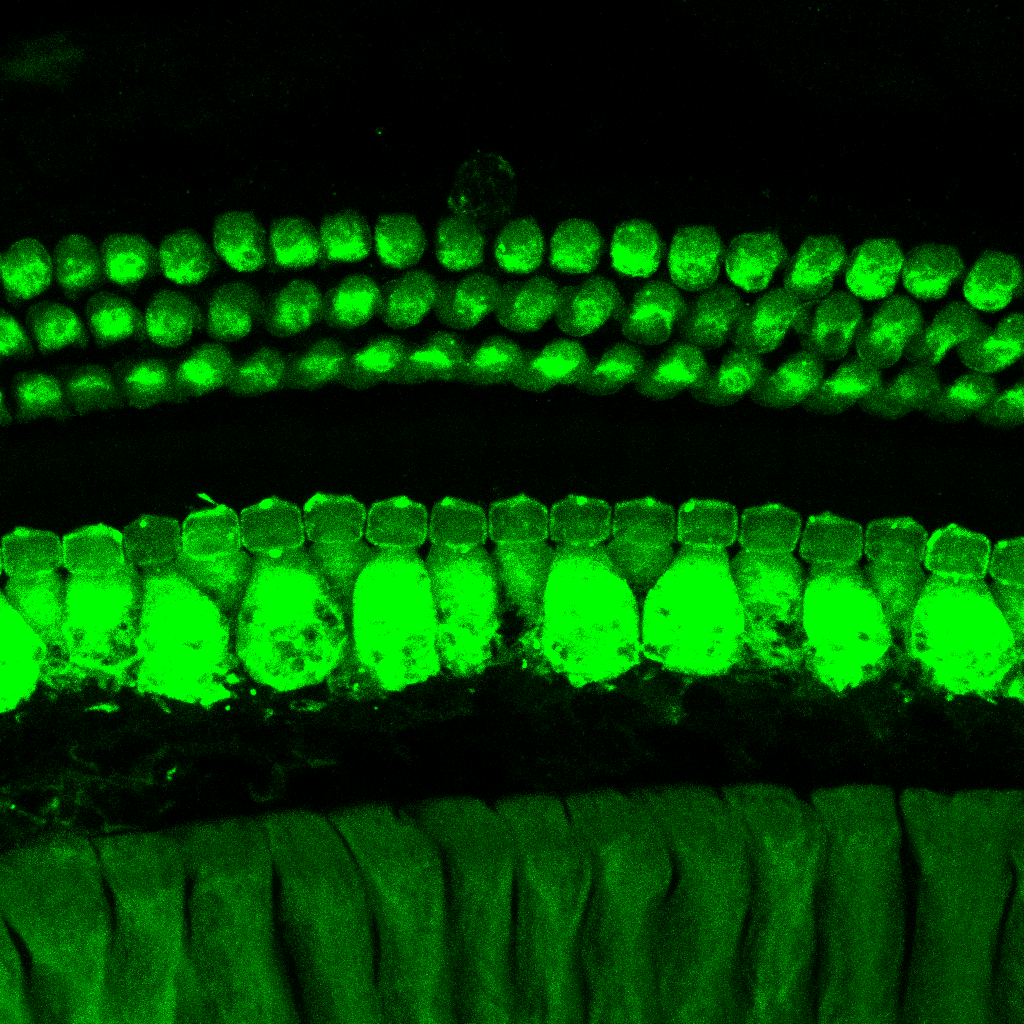

Supplement: Supplementary file 6 — Source Data for Figure 2 [file EMMM-15-e17611-s001.zip › Figure 2/2F-G/Whole mount immunofluorescence of Cgn expression Parvalbumin/P14 Cochlea Parvalbumin.tif]

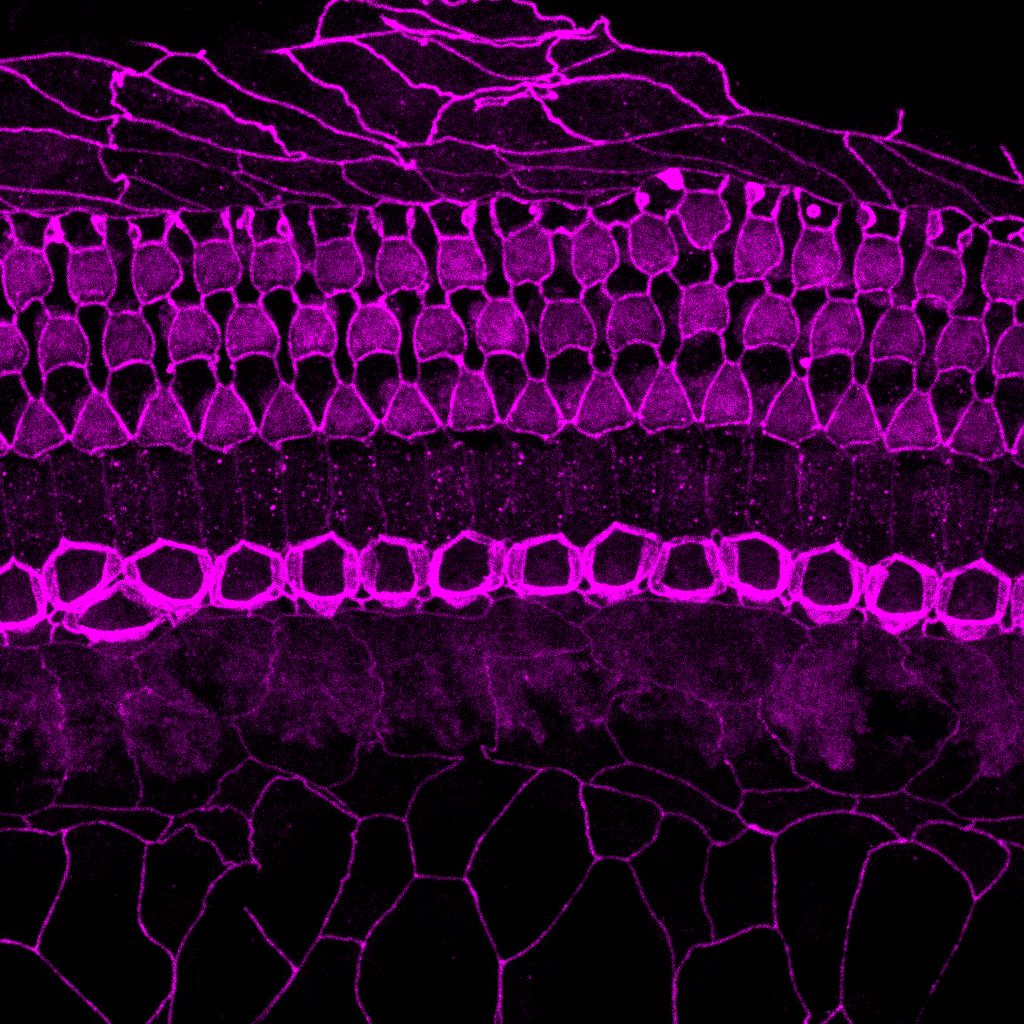

Supplement: Supplementary file 6 — Source Data for Figure 2 [file EMMM-15-e17611-s001.zip › Figure 2/2F-G/Whole mount immunofluorescence of Cgn expression Parvalbumin/P21 Cochlea CGN.tif]

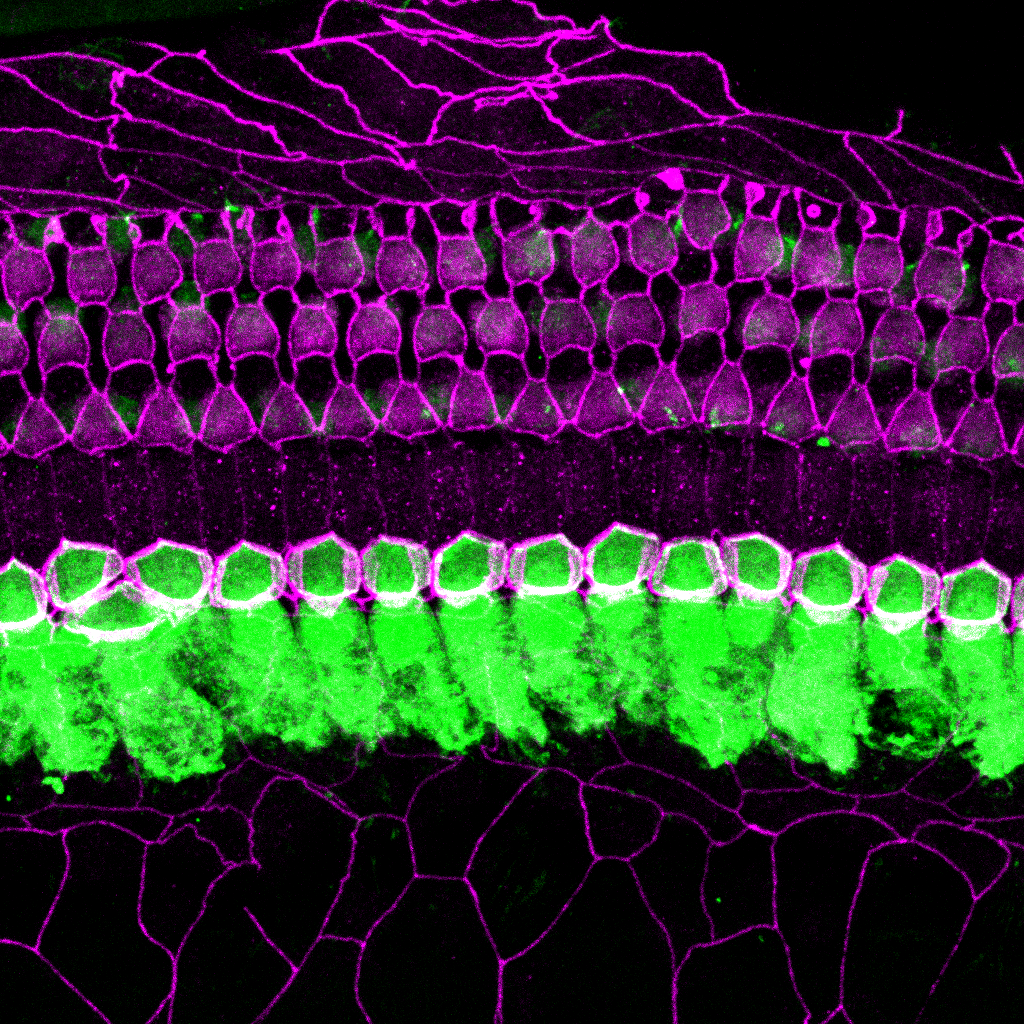

Supplement: Supplementary file 6 — Source Data for Figure 2 [file EMMM-15-e17611-s001.zip › Figure 2/2F-G/Whole mount immunofluorescence of Cgn expression Parvalbumin/P21 Cochlea Merge.tif]

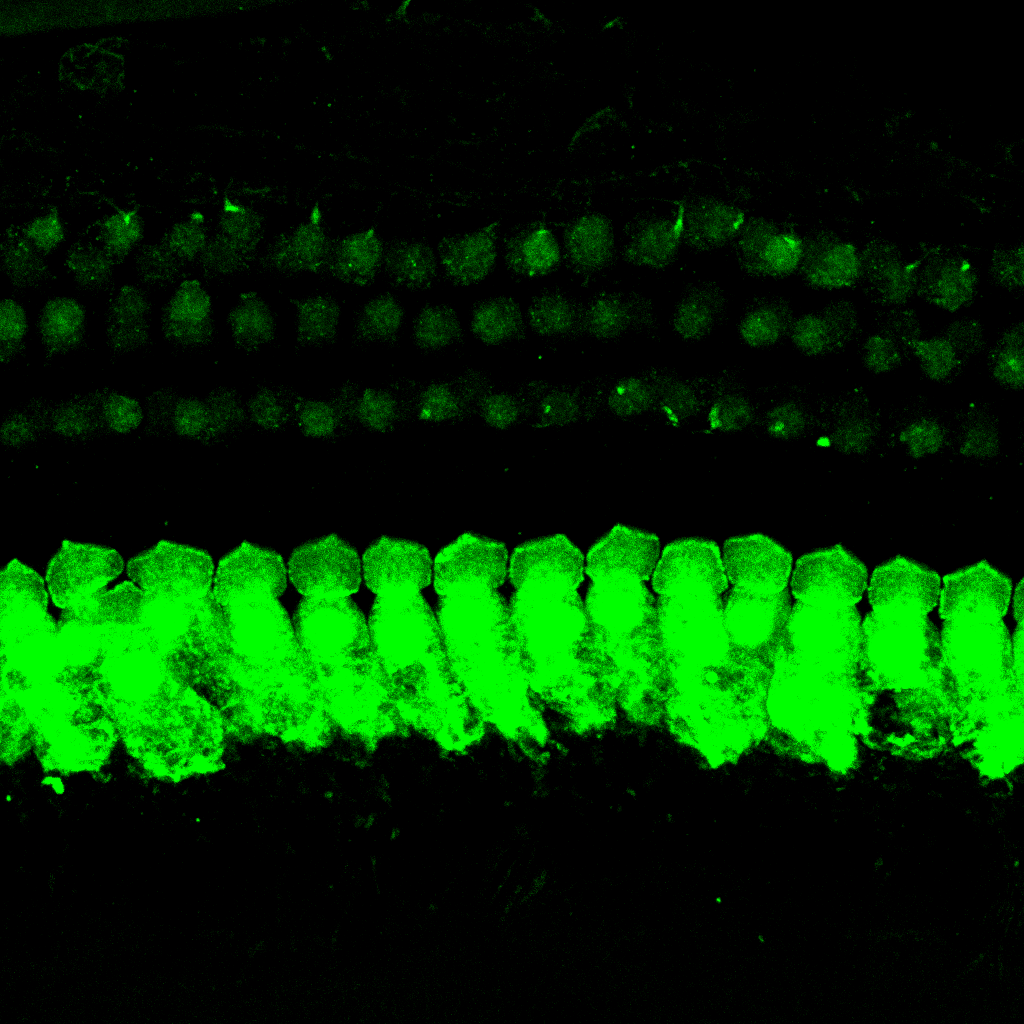

Supplement: Supplementary file 6 — Source Data for Figure 2 [file EMMM-15-e17611-s001.zip › Figure 2/2F-G/Whole mount immunofluorescence of Cgn expression Parvalbumin/P21 Cochlea Parvalbumin.tif]

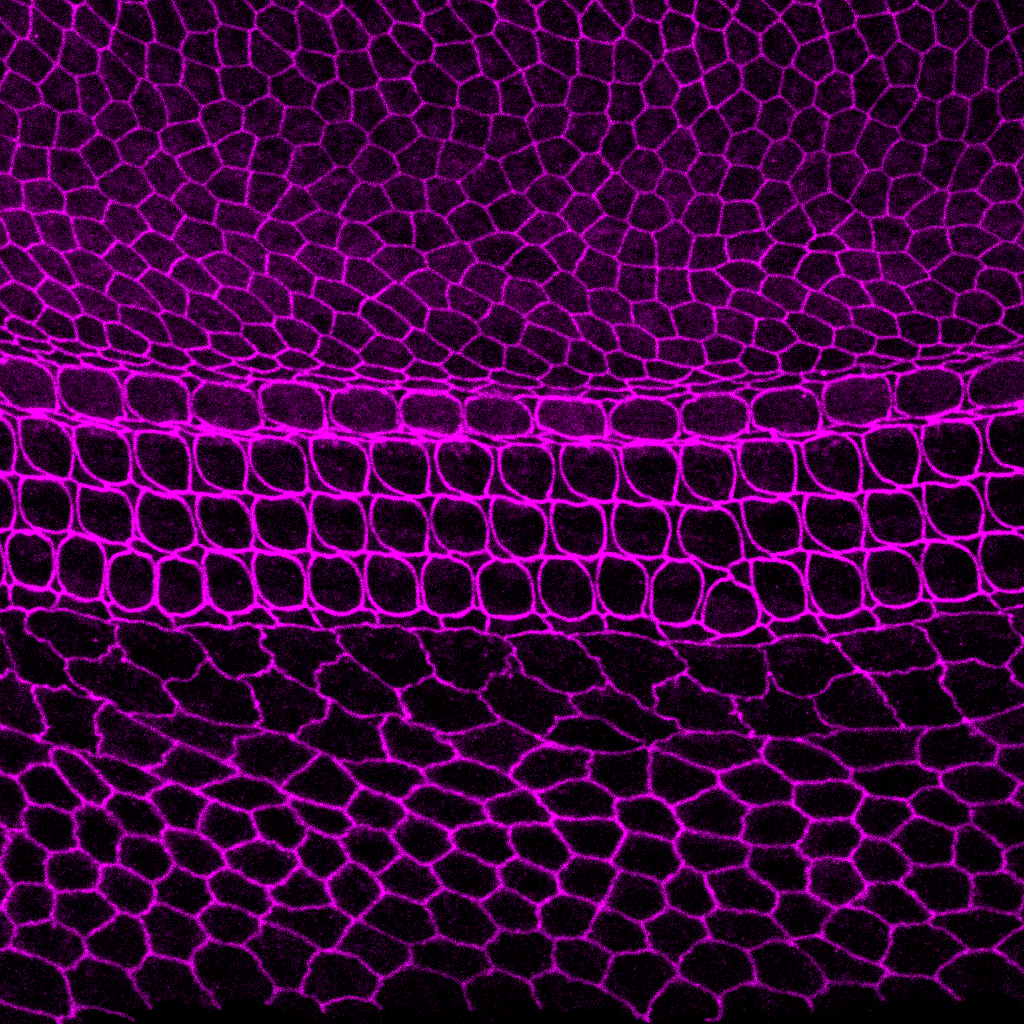

Supplement: Supplementary file 6 — Source Data for Figure 2 [file EMMM-15-e17611-s001.zip › Figure 2/2F-G/Whole mount immunofluorescence of Cgn expression Parvalbumin/P3 cochlea CGN.tif]

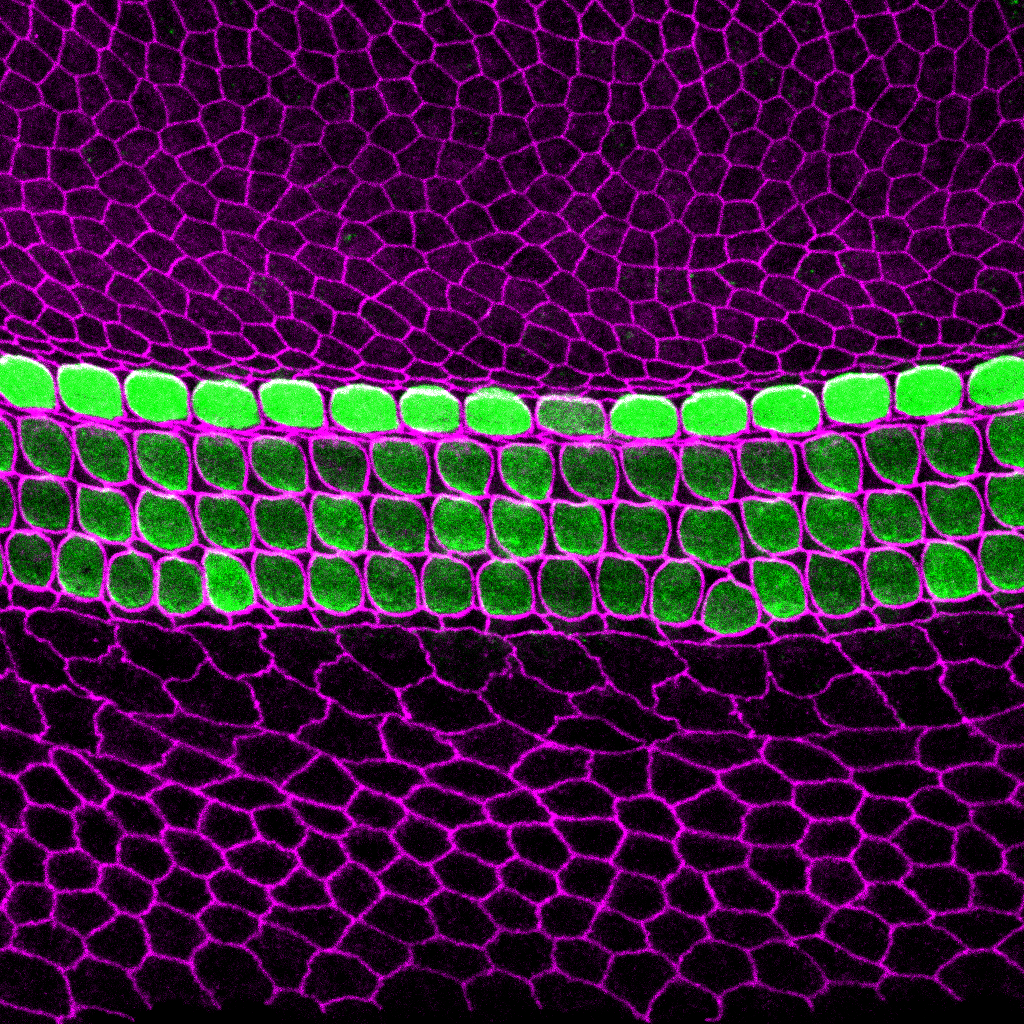

Supplement: Supplementary file 6 — Source Data for Figure 2 [file EMMM-15-e17611-s001.zip › Figure 2/2F-G/Whole mount immunofluorescence of Cgn expression Parvalbumin/P3 cochlea Merge.tif]

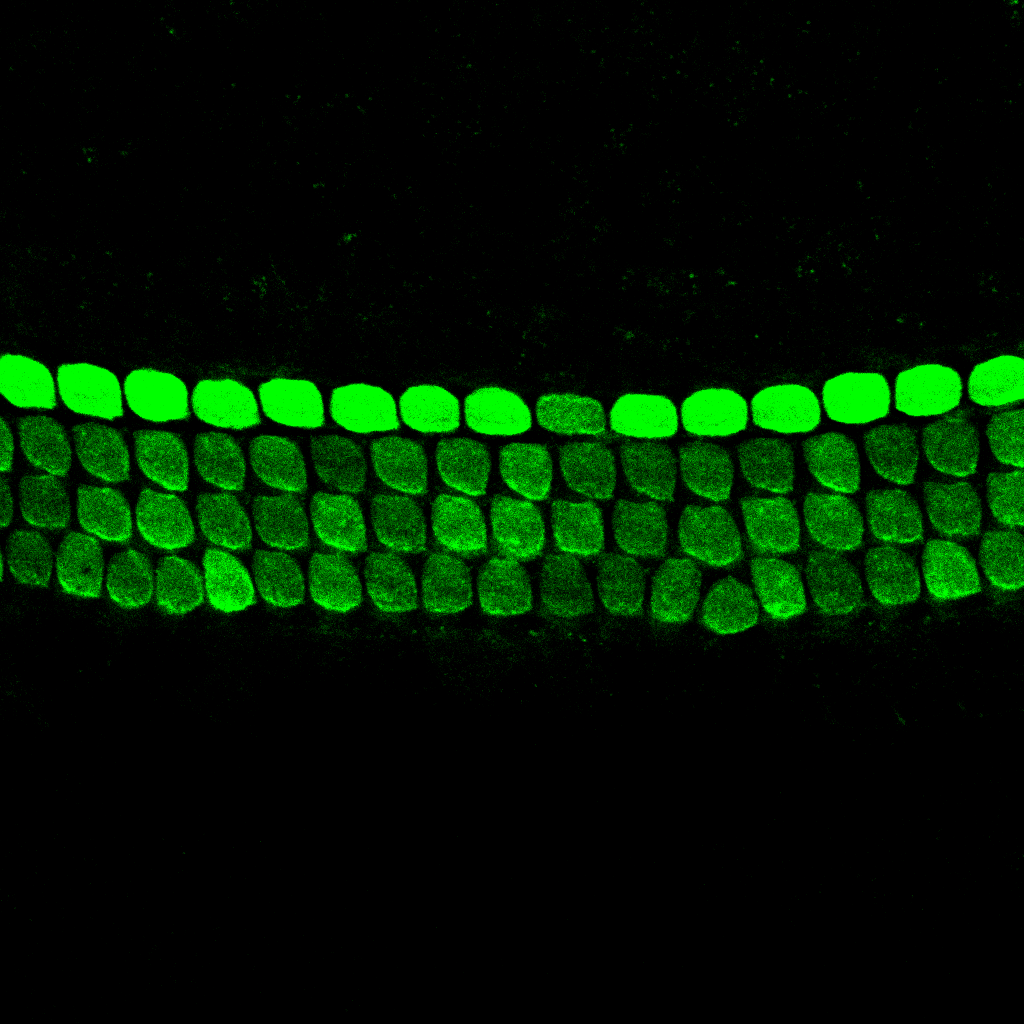

Supplement: Supplementary file 6 — Source Data for Figure 2 [file EMMM-15-e17611-s001.zip › Figure 2/2F-G/Whole mount immunofluorescence of Cgn expression Parvalbumin/P3 cochlea Parvalbumin.tif]
