# Supplementary material for: Cingulin regulates hair cell cuticular plate morphology and is required for hearing in human and mouse
Source: EMBO Mol Med. 2023 Sep 11;15(11):e17611. doi: 10.15252/emmm.202317611 (PMC10630877; doi:10.15252/emmm.202317611)
Supplement: Supplementary file 4 — PDF+ [file EMMM-15-e17611-s003.pdf]

Article

SOURCE DATA | TRANSPARENT PROCESS | OPEN ACCESS

EMBO Molecular Medicine

Cingulin regulates hair cell cuticular plate morphology and is required for hearing in human and mouse

Guang-Jie Zhu[1,2,3,†], Yuhang Huang[1,2,†], Linqing Zhang[1,2,†], Keji Yan[4,†], Cui Qiu[1,2], Yihan He[1,2], Qing Liu[1,2,3], Chengwen Zhu[1,2,3], Matías Morín[5,6], Miguel Ángel Moreno-Pelayo[5,6], Min-Sheng Zhu[1,2,3], Xin Cao[7], Han Zhou[1,3], Xiaoyun Qian[1,3], Zhigang Xu[4,*], Jie Chen[1,3,**], Xia Gao[1,3,***] & Guoqiang Wan[1,2,3,****]

Abstract

Cingulin (CGN) is a cytoskeleton-associated protein localized at the apical junctions of epithelial cells. CGN interacts with major cytoskeletal filaments and regulates RhoA activity. However, physiological roles of CGN in development and human diseases are currently unknown. Here, we report a multi-generation family presenting with autosomal dominant non-syndromic hearing loss (ADNSHL) that co-segregates with a *CGN* heterozygous truncating variant, c.3330delG (p.Leu1110Leufs*17). CGN is normally expressed at the apical cell junctions of the organ of Corti, with enriched localization at hair cell cuticular plates and circumferential belts. In mice, the putative disease-causing mutation results in reduced expression and abnormal subcellular localization of the CGN protein, abolishes its actin polymerization activity, and impairs the normal morphology of hair cell cuticular plates and hair bundles. Hair cell-specific *Cgn* knockout leads to high-frequency hearing loss. Importantly, *Cgn* mutation knockin mice display noise-sensitive, progressive hearing loss and outer hair cell degeneration. In summary, we identify *CGN* c.3330delG as a pathogenic variant for ADNSHL and reveal essential roles of CGN in the maintenance of cochlear hair cell structures and auditory function.

Keywords ADNSHL; cingulin; cuticular plate; hair cells; hearing loss
Subject Categories Genetics, Gene Therapy & Genetic Disease

DOI 10.15252/emmm.202317611 | Received 21 February 2023 | Revised 17 August 2023 | Accepted 22 August 2023 | Published online 11 September 2023
EMBO Mol Med (2023) 15: e17611

Introduction

Based on a recent report from the World Health Organization, more than 430 million people worldwide suffer from disabling hearing loss (Chadha *et al*, 2021). Genetic defects, excessive noise exposure, aging, and ototoxic drugs are all associated with hearing loss (Wang & Puel, 2018). Roughly one out of every 1,000 newborns have hearing impairment, and approximately 50% of these cases are associated with genetic factors (Wrobel *et al*, 2021). Non-syndromic hearing loss (NSHL) is the most common hereditary sensory defect (Kochhar *et al*, 2007). Eighty percent of genetic NSHL cases are autosomal recessive (ARNSHL), whereas 18% are autosomal dominant (ADNSHL) and the remaining 2% exhibit either X-linked or mitochondrial inheritance. ARNSHL is mainly characterized by congenital prelingual deafness that is generally not progressive, while ADNSHL is usually postlingual and progressive.

Currently, over 170 deafness loci have been mapped and mutations in more than 130 genes have been identified in individuals with hereditary hearing loss (Hereditary hearing loss homepage

1 State Key Laboratory of Pharmaceutical Biotechnology, Department of Otolaryngology Head and Neck Surgery, Jiangsu Provincial Key Medical Discipline (Laboratory), The Affiliated Drum Tower Hospital of Medical School, Model Animal Research Center of Medical School, Nanjing University, Nanjing, China
2 MOE Key Laboratory of Model Animal for Disease Study, Jiangsu Key Laboratory of Molecular Medicine, National Resource Center for Mutant Mice of China, Nanjing University, Nanjing, China
3 Research Institute of Otolaryngology, Nanjing, China
4 Shandong Provincial Key Laboratory of Animal Cells and Developmental Biology and Key Laboratory for Experimental Teratology of the Ministry of Education, School of Life Sciences, Shandong University, Qingdao, China
5 Servicio de Genética, Hospital Universitario Ramón y Cajal, IRYCIS, Madrid, Spain
6 Centro de Investigación Biomédica en Red de Enfermedades Raras, Instituto de Salud Carlos III (CB06/07/0048; CIBERER-ISCIII), Madrid, Spain
7 Department of Medical Genetics, School of Basic Medical Science, Nanjing Medical University, Nanjing, China
*Corresponding author. Tel: +86 532 58630869; E-mail: xuzg@sdu.edu.cn
**Corresponding author. Tel: +86 025 58641594; E-mail: njjiechen@163.com
***Corresponding author. Tel: +86 025 58641594; E-mail: gaoxia@nju.edu.cn
****Corresponding author. Tel: +86 025 58641594; E-mail: guoqiangwan@nju.edu.cn
†These authors contributed equally to this work

© 2023 The Authors. Published under the terms of the CC BY 4.0 license.

http://hereditaryhearingloss.org) (Petit *et al*, 2023). However, identification of ADNSHL deafness genes remains a difficult task due to substantial genetic and phenotypic heterogeneity. Indeed, some of the identified mutations have not been rigorously verified *in vitro* or *in vivo*. Therefore, identifying and validating novel mutations in genes associated with ADNSHL, and clarifying their pathogenic mechanisms, would not only help evaluate the efficacy of cochlear implant surgery but also provide tools for personalized treatment of hereditary deafness cases.

Tight junctions (TJs) are specialized intercellular junctions that form a continuous, belt-like seal between adjacent cells in epithelial and endothelial tissues (Sawada *et al*, 2003). TJs in the inner ear are critical for the maintenance of the unique fluid environment in the cochlea and of the electrochemical gradient that drives mechanotransduction of sound (Gao *et al*, 2023). Defects in multiple tight junction-related proteins, such as ZO-2 (*TJP2*), claudin-9 (*CLDN9*), claudin-14 (*CLDN14*), and claudin-11 (*CLDN11*) are associated with hearing loss in humans and mice (Wilcox *et al*, 2001; Gow *et al*, 2004; Walsh *et al*, 2010; Sineni *et al*, 2019). In addition, mutations in genes encoding the tricellular TJ components, including tricellulin (*TRIC*) and angulin-2 (*ILDR1*), also result in human deafness (Riazuddin *et al*, 2006; Borck *et al*, 2011), which can be recapitulated in knockout mouse models (Nayak *et al*, 2013; Morozko *et al*, 2015). These reports indicate that the integrity of cochlear TJs is pivotal to auditory function.

Cingulin (CGN, OMIM: 609437) is a 140-kDa TJ-related protein localized in the apical circumferential belts of epithelial cells (Citi *et al*, 2012). CGN was discovered by its copurification with myosin II from intestinal epithelial cells. Studies have shown that CGN can regulate myosin phosphorylation through RhoA and Rac1 (Aijaz *et al*, 2005), tether the nonmuscle myosin-2 to the junction (Rouaud *et al*, 2023), regulate the activity of Rho family GTPases, and thereby regulate the formation and membrane tortuosity of epithelial tight junctions (Gonzalez-Mariscal *et al*, 2014). CGN also interacts with TJ proteins ZO-1 and ZO-2, thereby linking TJs with the actin cytoskeleton (Citi *et al*, 2000, 2012). Interestingly, a recent report demonstrated that inhibition of *Cingulin b* expression leads to abnormal development of the zebrafish lateral line (Lu *et al*, 2022). However, the physiological function of CGN in the auditory system is still unclear, and no *CGN* mutations have been associated with human diseases, including deafness.

Using linkage analysis and exome sequencing, here we report an ADNSHL family in which all affected individuals carry a deletion mutation in exon 20 of the *CGN* gene (c.3330delG, p.Leu1110Leufs*17). We demonstrate that CGN is preferentially localized at the hair cell cuticular plate and circumferential belt in normal control mice. Studies *in vitro* and in a knockin mouse line indicate that the p.Leu1110Leufs*17 mutation results in reduced expression and abnormal subcellular localization of CGN. Mice with either CGN conditional knockout or p.Leu1110Leufs*17 knockin display increased sensitivity to noise damage, progressive hearing loss, and hair cell degeneration. Our findings support *CGN* as a novel deafness gene and indicate that CGN is required for maintenance of the normal apical structure of sensory hair cells in the cochlea and plays a critical role in auditory function.

Results

ADNSHL is diagnosed in 10 individuals from a Chinese pedigree

We ascertained a pedigree of Chinese Han origin, with bilateral, postlingual ADNSHL, without tinnitus or vestibular symptoms. In affected individuals, hearing loss started at middle to high frequencies by 6–7 years of age, and subsequently progressed to all frequencies by the first or second decade (Figs 1A and EV1). For example, during our 6 years of clinical follow-up, the pure tone threshold of the proband (IV:2) was elevated by about 30 dB on average (Fig 1B). Otoscopic examinations revealed a normal external auditory canal and tympanic membranes with normal tympanometry. Auditory steady-state response (ASSR), temporal bone high-resolution computed tomography (HRCT), and magnetic resonance imaging (MRI) for the proband (at 13 years of age) were normal, excluding auditory neuropathy spectrum disorders and inner ear malformations as potential causes of hearing loss. Excessive noise exposure, ototoxic medication history, or complaints of other systemic abnormalities were absent from the clinical reports of the affected individuals. A summary of the auditory phenotype is shown in Table 1.

A *CGN* variant co-segregates with the ADNSHL

To identify the pathogenic variant, exome sequencing (ES) was carried out on seven affected and five unaffected individuals of the family. Genome-wide linkage analysis of the ES results identified two candidate regions on chromosome 1 and one candidate region on chromosome 6, all with a maximum logarithm of the odds (LOD) score of 2.1 (Appendix Fig S1A and B). Further analysis revealed variants in a total of seven candidate genes (*ITGA10*, *CGN*, *IL6R*, *MST1L*, *SERPINB6*, *SSR1*, and *TUBB2B/PSMG4*) that co-segregated with the disease phenotype (Appendix Fig S1C). However, except for the *CGN* variant, all other variants were common single-nucleotide polymorphisms (SNPs) and, therefore, excluded from further analyses.

Sanger sequencing on the seven affected and five unaffected individuals, confirmed that the *CGN* variant co-segregating with hearing loss was c.3330delG located in exon 20 of the human gene (NM_020770) (Fig 1C and D). The variant resulted in a frameshift and premature truncation of the normal C-terminal tail of the CGN protein at amino acid Leu1110 (p.Leu1110Leufs*17), which is highly conserved among the mammalian species (Fig 1E and F). The variant was predicted to be deleterious by the Mutation Taster, PolyPhen-2, and SIFT computational tools and was not present in the dbSNP, 1000 Genomes, ESP6500, nci60, GnomAD databases or in 10,588 ethically matched control individuals (Cao *et al*, 2020). This variant also was not detected in 150 additional sporadic cases of postlingual NSHL patients.

We then enrolled in the study a selection of 96 probands of independent familiar cases with hereditary hearing loss compatible with an autosomal dominant inheritance pattern. These samples have been previously subjected to genetic screening using the custom gene panel, OTO-NGS, version 1 and 2, designed in our laboratory (Morin *et al*, 2020), with no causative deafness mutations being identified. *CGN* exon screening in this Spanish cohort only revealed benign or likely benign genetic variations, all missense in nature

© 2023 The Authors

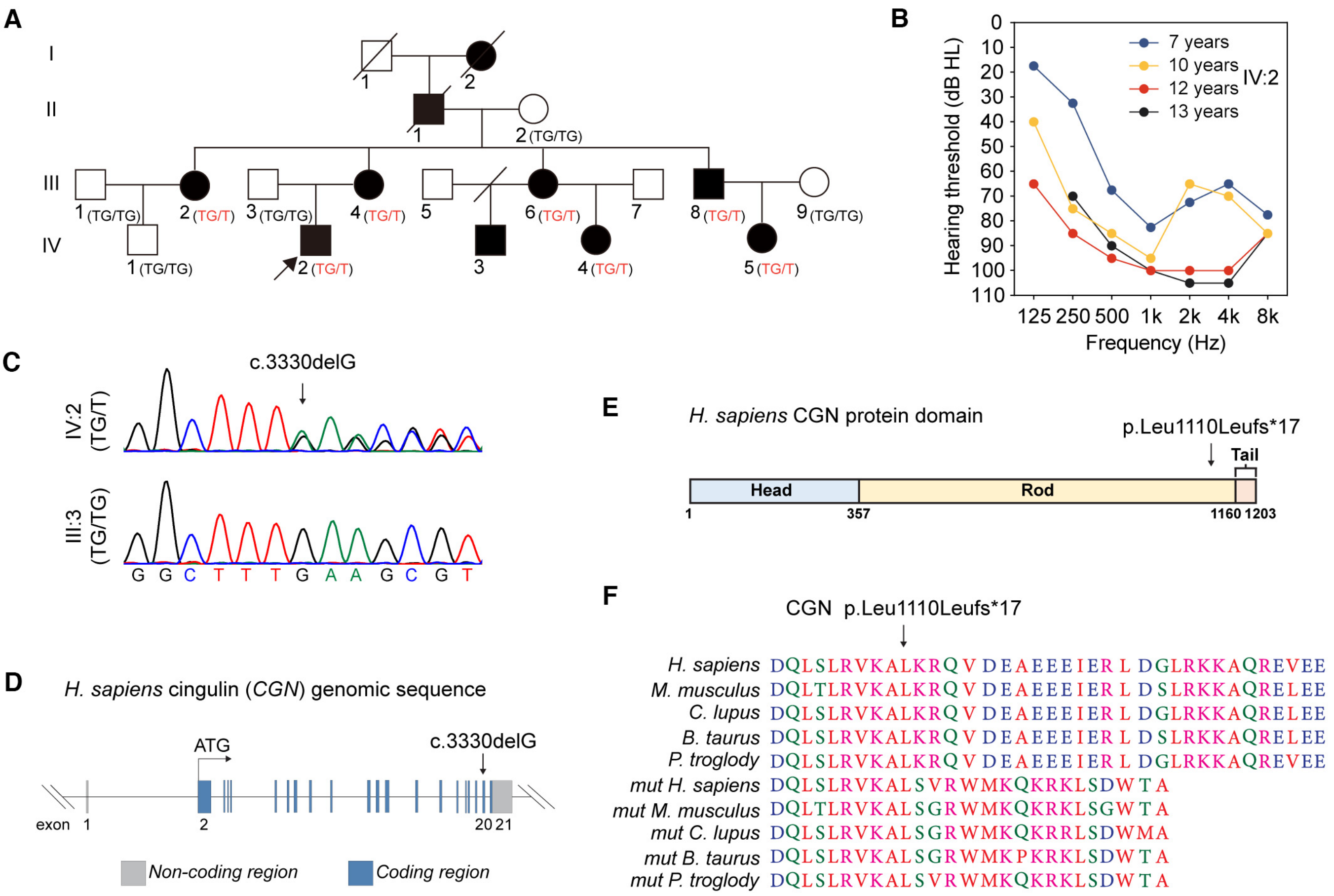

Figure 1. A heterozygous *CGN* variant identified in affected individuals of an autosomal dominant non-syndromic hearing loss family.

A Pedigree of the *CGN* c.3330delG variant within the family. Circles and squares represent females and males, respectively. Filled symbols denote individuals with hearing loss and non-filled symbols show individuals with normal hearing.

B Audiograms of the proband (IV:2) over 6 years of follow-up.

C Sanger sequencing of the *CGN* c.3330delG variant present in affected proband (IV:2) but not in his unaffected father (III:3).

D Exon organization of the human *CGN* gene with position of the variant (c.3330delG).

E Domain structure of the CGN protein and position of the variant (p.Leu1110Leufs*17).

F Conservation of the Leu1110 residues of CGN in different mammalian species. Protein products with the CGN mutation (mut) in various species were also displayed.

Source data are available online for this figure.

Table 1. Audiological phenotypes of the affected individuals.

Patient ID	Age[a] (years)	Gender	Age at HL onset	Severity	Progressive HL	Laterality
III:2	49	Female	6	Complete HL	Yes	Bilateral
III:4	47	Female	6	Complete HL	Yes	Bilateral
III:6	46	Female	6	Complete HL	Yes	Bilateral
III:8	43	Male	6	Complete HL	Yes	Bilateral
IV:2	13	Male	7	Complete HL	Yes	Bilateral
IV:3	10	Male	6	Profound HL	Yes	Bilateral
IV:4	9	Female	6	Complete HL	Yes	Bilateral
IV:5	14	Female	7	Complete HL	Yes	Bilateral

[a]Age at last examination.

with some detected in two probands of independent familial cases (Appendix Table S1). These results indicate that the *CGN*-associated hearing loss is likely one of the rarer forms of genetic deafness, similar to those linked to *HOMER2* or *CCDC50* genes (Modamio-Hoybjor *et al*, 2007; Lachgar *et al*, 2021).

CGN is expressed in the mouse cochlea

To explore potential roles of *CGN*, we first examined its expression pattern in different mouse tissues, including the cochlea by RT-qPCR analysis. *Cgn* mRNA is widely expressed in mouse tissues, including cochlea, kidney, and liver, with higher expression levels in lung, gonad, and intestine (Fig 2A). Western blot analysis demonstrated that the 140-kDa CGN protein is expressed in the cochlea and the other tissues examined (Fig 2B). The specificity of the three CGN antibodies used in this study was validated by western blot and immunofluorescence analyses of cell lines transfected with *CGN* plasmids (Appendix Fig S2). In the postnatal mouse cochlea, *Cgn* expression is detected at P0, and increases from P7 to P21 (Fig 2C and D). Based on previously published cell-type-specific RNA-seq results (Liu *et al*, 2018a; Data ref: Liu *et al*, 2018b), *Cgn* is widely expressed in P28-35 cochlear sensory epithelial cells, with enriched expression in both inner (IHCs) and outer (OHCs) hair cells (Fig 2E). Consistently, immunofluorescence analysis of whole mount cochleae showed that CGN protein is mainly expressed at the cellular junctions in the organ of Corti, with enriched localization at the cuticular plates and circumferential belts of both cochlear IHCs and OHCs (Fig 2F). The presence of CGN at the cuticular plates of OHCs was further confirmed by its co-immunostaining with LMO7 (Fig 2G), a cuticular plate marker protein (Du *et al*, 2019). Similar to the RT-qPCR and Western blot analyses (Fig 2C and D), expression levels of CGN at the cuticular plates of OHCs increase gradually from P3 to P21 (Fig 2G).

The *CGN* variant results in abnormal expression of CGN protein

In order to evaluate the effect of the mutation on CGN expression, we constructed both wild-type (WT) and mutant human *CGN* (p.L1110Lfs*17) plasmids with a C-terminal FLAG-tag (Fig 3A), and transfected them into Madin–Darby canine kidney (MDCK) cells. Compared to the WT CGN, mutant CGN was expressed at significantly lower levels by either immunofluorescence (Fig 3B) or Western blot (Fig 3C). Reduced protein expression of the mutant CGN was not due to alterations in steady-state RNA as shown by comparable levels of the WT and mutant *CGN* mRNA (Fig 3D). Furthermore, transfected WT CGN protein showed preferential localizations at the cell periphery with sheet-like or filamentous accumulations in the cytoplasm (Fig 3E), similar to previous observations (Citi *et al*, 2009). In contrast, expression of the residual mutant CGN protein was mainly distributed in the cytoplasm as puncta and failed to localize to the cell periphery (Fig 3E). Similar results were also observed in human colon epithelial cancer (CACO2) cells, human embryonic kidney (HEK) 293T, and CV-1 in Origin Simian-7 (COS-7) cells (Appendix Fig S3). To exclude possible interference of the C-terminal Flag-tag on CGN expression, N-terminal EGFP-tagged WT and mutant human *CGN* plasmids were also constructed and transfected into MDCK or HEK293T cells (Fig EV2). The N-terminal EGFP-tagged mutant CGN exhibited the same reduced protein expression and abnormal subcellular localization as the C-terminal Flag-tag protein (Fig EV2). These results suggest that the expression and subcellular localization of human CGN are significantly altered by the candidate deafness mutation.

Deletion of *Cgn* in hair cells induces hearing impairment

As wildtype CGN is highly expressed at the apical surfaces of hair cells, we generated a hair cell-specific *Cgn* conditional knockout mice (*Cgn-cKO*) by crossing *Cgn*$^{fl/fl}$ mice with the inner ear hair cell-specific CreER line, *Pou4f3*$^{EGFP\text{-}creER}$ mice (Du *et al*, 2020) (Fig EV3A and B). Following tamoxifen injection at P3-P6, *Cgn-cKO* mice lost substantial CGN immunoreactivity in both IHCs and OHCs at 2 months of age (Fig EV3C), and the average Cre recombination efficiency was about 60% (Fig EV3D).

We then assessed the auditory function in 2-month-old *Cgn-cKO* mice by measuring distortion product otoacoustic emissions (DPOAE) which reflect the activity of OHCs, and auditory brainstem responses (ABR), which represent the sound-evoked neural activity in the auditory nerve and brainstem. Compared to wild-type littermates, *Cgn-cKO* mice had significant elevations in DPOAE and ABR thresholds at 32 kHz (Fig 4A and B). In addition, ABR peak 1 (P1) amplitudes, the summed activity of the cochlear nerve, were also reduced at 8–32 kHz (Fig 4C–I). Histological analyses demonstrated significant loss of OHCs in *Cgn-cKO* mice at the 32 kHz cochlear region (Fig 4J and K), consistent with observed ABR and DPOAE threshold elevations. These data indicate that *Cgn* deletion in cochlear hair cells leads to high-frequency OHC loss and hearing impairment.

Cgn^{delG} knockin mice display dose-dependent progressive hearing loss

To determine the pathophysiological roles of the *CGN* variant (c.3330delG) identified in the affected individuals, we generated

Figure 2. Expression pattern of CGN in mouse cochlea and other organs.

A, B RT-qPCR (n = 3–12 biological replicates) (A) and Western blot (B) analyses of CGN expression in different mouse organs at P21.
C, D RT-qPCR (n = 6 cochleae) (C) and Western blot (D) analyses of CGN expression in postnatal mouse cochlea.
E Expression levels of *Cgn* in different cochlear cell types of P28-35 mouse (n = 4–6 biological replicates). Data were extracted from RNA-seq results published by Liu *et al* (2018a) and Liu *et al* (Data ref: Liu *et al*, 2018b). Unit: RPKM, Reads Per Kilo base per Million mapped reads; IHC, inner hair cell; OHC, outer hair cell; DC, Deiters cell; PC, Pillar cell.
F, G Whole mount immunofluorescence of CGN expression in P3, P14, and P21 mouse cochlea coimmunostained with hair cell marker Parvalbumin (F) or cuticular plate marker LMO7 (G). CGN expression levels at the cuticular plates were normalized to LMO7 immunofluorescent signals (n = 130–193 hair cells from 3 to 4 cochleae).

Data information: Data are presented as mean ± SEM; unpaired Student's t-test was used in (C), one-way ANOVA was used in (E and G). *$P < 0.05$, **$P < 0.01$, ***$P < 0.001$, and ****$P < 0.0001$.
Source data are available online for this figure.

 © 2023 The Authors

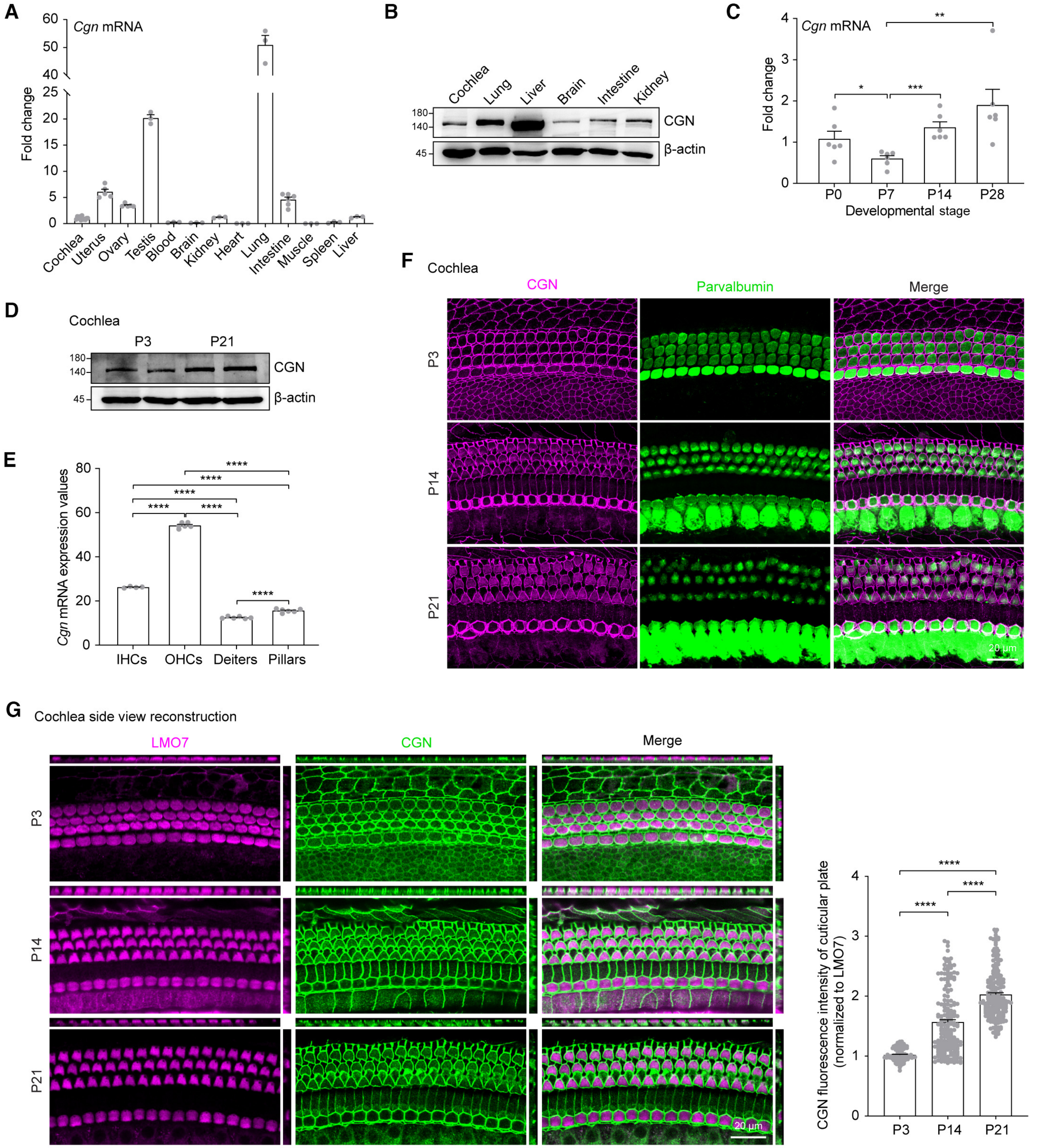

Figure 2.

Cgn knockin mice (*Cgn*^{delG} mice) harboring the exact G-base deletion using CRISPR-Cas9 technology (Fig EV3E–G). *Cgn* mRNA expression in cochlea of homozygous *Cgn* knockin (*CGN*$^{delG/delG}$) mice was significantly decreased compared with wild-type (*Cgn*$^{+/+}$) and heterozygous *Cgn* knock in (*Cgn*$^{delG/+}$) mice (Fig 5A), likely due to nonsense mediated mRNA decay (NMD) of the frameshifted mutant *Cgn* mRNA. Western blot results confirmed that CGN protein was expressed as a truncated form and that expression was significantly reduced in cochlea and lung tissues from both heterozygous and homozygous *Cgn*^{delG} mice (Figs 5B and EV4A).

© 2023 The Authors

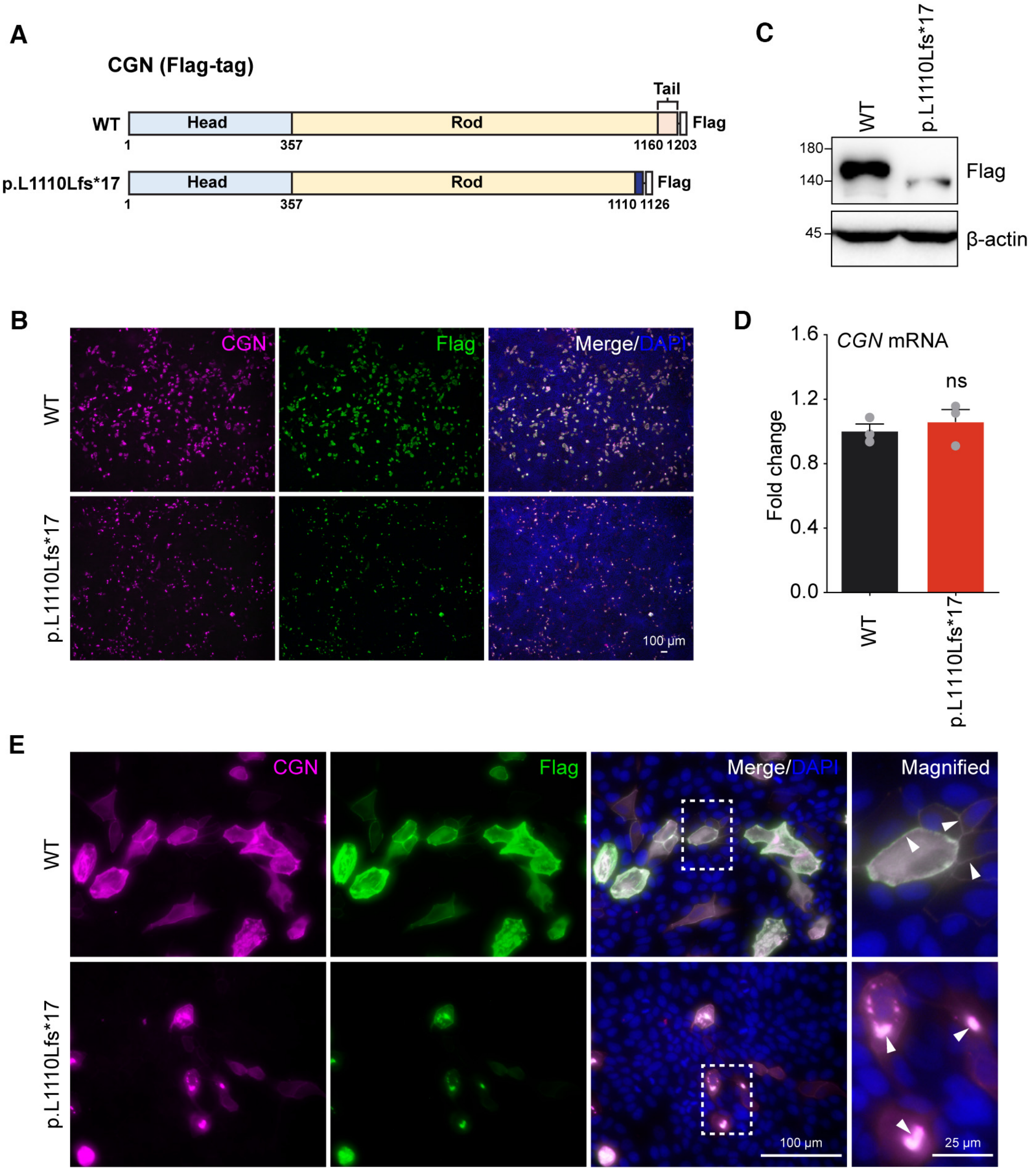

Figure 3. Abnormal expression pattern of the mutant human CGN in MDCK cells.

A A schematic diagram of C-terminal Flag-tagged CGN WT and p.Leu1110Leufs*17 (p.L1110Lfs*17) constructs used in this study.
B MDCK cells expressing WT or mutant CGN were immunolabeled with CGN or Flag antibodies.
C Western blot analysis of whole cell lysates from MDCK cells transfected with WT or mutant CGN. Exogenous CGN was immunoblotted with Flag antibody.
D RT-qPCR of *CGN* expression in transfected MDCK cells (n = 3 biological replicates).
E High magnification immunofluorescent images showing subcellular localizations of WT and mutant CGN (white arrows).

Data information: Data are presented as mean ± SEM; unpaired Student's t-test was used in (D). ns, not significant, $P > 0.05$.
Source data are available online for this figure.

Whole mount immunofluorescence of *Cgn*^{delG} mice cochlea showed similar results (Figs 5C and EV4B).

Similar to the *Cgn-cKO* mouse, DPOAE and ABR thresholds were elevated in *Cgn*$^{delG/delG}$ mice at 2 and 6 months of age (Fig 5D–G). At 2 months of age, *Cgn*$^{delG/delG}$ mice exhibited significant decreases in ABR P1 amplitudes (32 kHz) (Fig 5H). Although hearing thresholds in the heterozygous *Cgn*$^{delG/+}$ mice were normal, the ABR P1 amplitudes (32 kHz) of the *Cgn*$^{delG/+}$ mice were significantly decreased at 6 months (Fig 5I), but not at 2 months (Fig 5H). These results indicated that the *Cgn delG* mutation results in a dose-dependent and progressive hearing impairment in the knockin mice. Similar to the *Cgn-cKO* mice, OHCs were also lost at high frequency

 © 2023 The Authors

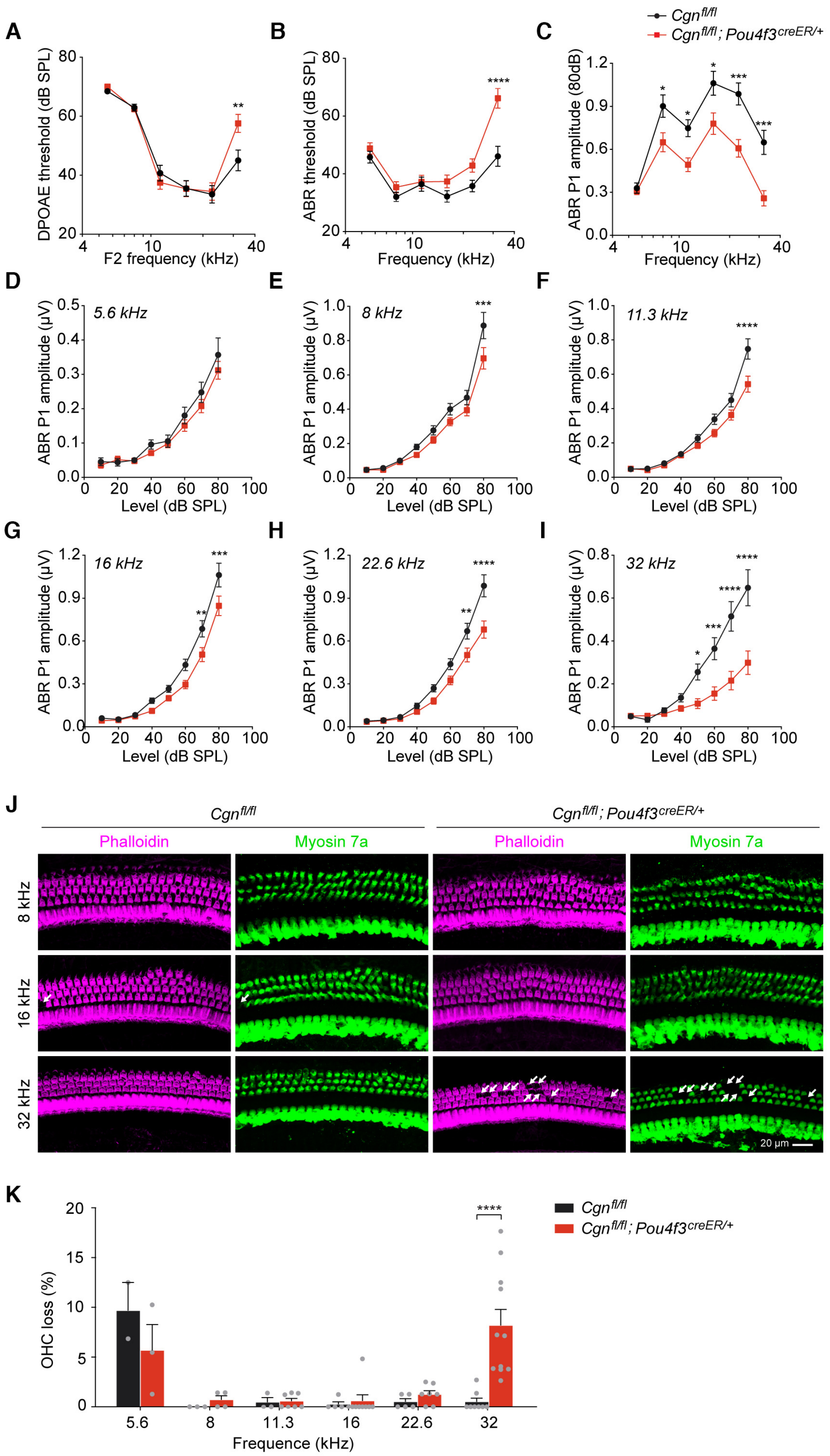

A
B
C
Cgn^fl/fl
Cgn^fl/fl; Pou4f3^creER/+
DPOAE threshold (dB SPL)
F2 frequency (kHz)
ABR threshold (dB SPL)
Frequency (kHz)
ABR P1 amplitude (80dB)
D
E
F
G
H
I
5.6 kHz
8 kHz
11.3 kHz
16 kHz
22.6 kHz
32 kHz
ABR P1 amplitude (μV)
Level (dB SPL)
J
Phalloidin
Myosin 7a
8 kHz
16 kHz
32 kHz
20 μm
K
OHC loss (%)
Frequence (kHz)

Figure 4.

Figure 4. Hearing loss and OHC degeneration of the *Cgn-cKO* mice.

A–C DPOAE thresholds (A), ABR thresholds (B), and ABR P1 amplitudes (C) of the 2-month-old control ($Cgn^{fl/fl}$) and *Cgn-cKO* ($Cgn^{fl/fl};Pou4f3^{creER/+}$) mice. Control mice (circle), $n = 16$ mice (female = 8, male = 8); *Cgn-cKO* mice (square), $n = 19$ mice (female = 11, male = 8).

D–I Growth curves of ABR P1 amplitudes at different cochlear frequencies (5.6–32 kHz) of the 2-month-old control and *Cgn-cKO* mice. Control mice (circle), $n = 16$ mice (female = 8, male = 8); *Cgn-cKO* mice (square), $n = 19$ mice (female = 11, male = 8).

J Whole mount immunofluorescence of the organ of Corti from 2-month-old control and *Cgn-cKO* mice. Phalloidin (F-actin, magenta); Myosin 7a (hair cells, green). White arrows label the lost OHCs.

K Percentage of OHC loss in 2-month-old control and *Cgn-cKO* mice ($n = 2$–11 cochleae).

Data information: Data are presented as mean ± SEM; two-way ANOVA was used in (A–I and K). *$P < 0.05$, **$P < 0.01$, ***$P < 0.001$ and ****$P < 0.0001$.
Source data are available online for this figure.

in the $Cgn^{delG/delG}$ mice (Fig 5J and K). Together, these data show that the reduced expression of CGN in the $Cgn^{delG/delG}$ mice results in hearing impairment and loss of OHCs. However, OHCs of the heterozygous knockin mice were not lost, suggesting that other hair cell pathologies may also contribute to the high-frequency hearing loss in Cgn^{delG} mice.

Interestingly, CGN is also highly expressed in the apical junctions of the utricular sensory epithelia of wild-type mice (Fig EV5A and B) but reduced in Cgn^{delG} mice (Fig EV5C). However, balance function behavior assessed by rotarod tests is normal in the mutant mice (Fig EV5D). These results suggest that although CGN is widely expressed in sensory epithelia of cochlea and utricle, it is essential for normal auditory function but dispensable for vestibular function, consistent with a lack of overt vestibular symptoms in human patients.

Cgn^{delG} knockin mice have increased sensitivity to noise exposure

To evaluate whether carrying the Cgn^{delG} mutation alters the sensitivity to environmental insults, 2-month-old mice were exposed to broadband noise at 100 dB SPL for 2 h (Fig 6A). This noise exposure results in temporary shifts of auditory thresholds, which typically recover to baseline after 1 week in wild-type mice ($Cgn^{+/+}$). In contrast, DPOAE thresholds, ABR thresholds, and ABR P1 amplitudes (32 kHz) of the $Cgn^{delG/delG}$ mice exhibited larger impairment after noise exposure (Fig 6B–D). Remarkably, the heterozygous $Cgn^{delG/+}$ mice also showed significant hearing impairment after noise exposure in all three auditory measures (Fig 6B–D). Consistently, OHCs were lost at high frequency (32 kHz) in $Cgn^{delG/+}$ and $Cgn^{delG/delG}$ mice after noise exposure (Fig 6E and F). These data indicate that both $Cgn^{delG/+}$ and $Cgn^{delG/delG}$ mice are more sensitive to moderate noise insults than wild types, and that noise exposure exacerbates overall hearing impairment in the heterozygous Cgn^{delG} mice.

Cgn^{delG} mice show normal synaptic density, tight junction, and microtubule structures

Since the reduced ABR P1 amplitudes that we observed in Cgn^{delG} mice are often associated with loss of synapses formed between IHCs and SGN fibers, a.k.a. cochlear synaptopathy (Kohrman *et al*, 2020), we compared the number of ribbon synapses in wild-type and *Cgn* mutants (Appendix Fig S4A). The synaptic ribbon density in Cgn^{delG} mice was comparable to that in wildtype mice (Appendix Fig S4B), indicating that the hearing impairment of the Cgn^{delG} mice may not be due to synaptic loss.

Cingulin is specifically localized at the apical junctions of cells (Citi *et al*, 2012) (Fig 3E) and the circumferential belts in the organ of Corti (Fig 2F and G). It has been suggested that CGN might stabilize tight junction structures by regulating the activity of Rho family GTPases (Gonzalez-Mariscal *et al*, 2014). Therefore, we speculated that hearing impairment in *Cgn* mutant mice might be related to the disruption of its regulatory function in tight junctions. Therefore, we evaluated the expression of several markers associated with tight junction formation in MDCK cells overexpressing wildtype and mutant human *CGN* plasmids. Overexpression of the mutant human *CGN* had no significant effect on mRNA and protein expression of tight junction markers, Occludin and ZO-1 (Appendix Fig S5A and B). mRNA expression levels of other tight junction-related markers (Claudin-1, Claudin-3, Claudin-5, ZO-2, ZO-3, GEF-H1, RhoA, and ZONAB) were also unaffected (Guillemot & Citi, 2006) (Appendix Fig S5B). We next used shRNAs reported in a previous study to knock down (KD) endogenous *Cgn* in MDCK cells (Guillemot & Citi, 2006) (Appendix Fig S5C), and examined whether CGN

Figure 5. Abnormal CGN expression, hearing loss, and OHC degeneration of the Cgn^{delG} mice.

A RT-qPCR of *Cgn* mRNA expression in P21 Cgn^{delG} cochleae ($n = 4$–6 cochleae).

B Western blot and quantification of CGN protein expression in P21 Cgn^{delG} cochlear lysates ($n = 6$ cochleae).

C Whole mount immunofluorescence of CGN expression in organ of Corti from P21 Cgn^{delG} mouse.

D–I DPOAE thresholds (D, E), ABR thresholds (F, G), and ABR P1 amplitudes at 32 kHz (H, I) of 2-month-old (D, F, H) or 6-month-old (E, G, I) Cgn^{delG} mice. Control $Cgn^{+/+}$ mice (circle), $n = 3$ mice (2-month, female = 1, male = 2) or 15 mice (6-month, female = 4, male = 11); $Cgn^{delG/+}$ mice (triangle), $n = 8$ mice (2-month, female = 4, male = 4) or 20 mice (6-month, female = 9, male = 11); $Cgn^{delG/delG}$ mice (square), $n = 7$ mice (2-month, female = 4, male = 3) or 16 mice (6-month, female = 6, male = 10). $Cgn^{+/+}$ versus $Cgn^{delG/delG}$, * (red); $Cgn^{+/+}$ versus $Cgn^{delG/+}$, * (green).

J Whole mount immunofluorescence of the organ of Corti from 7-month-old Cgn^{delG} mice. White arrows indicate lost OHCs by F-actin labeling (Phalloidin).

K Percentage of OHC loss in 7-month-old Cgn^{delG} mice ($n = 3$–8 biological replicates from three cochleae).

Data information: Data are presented as mean ± SEM; one-way ANOVA was used in (A and B), two-way ANOVA was used in (D–I and K). *$P < 0.05$, **$P < 0.01$, ***$P < 0.001$, and ****$P < 0.0001$.
Source data are available online for this figure.

 © 2023 The Authors

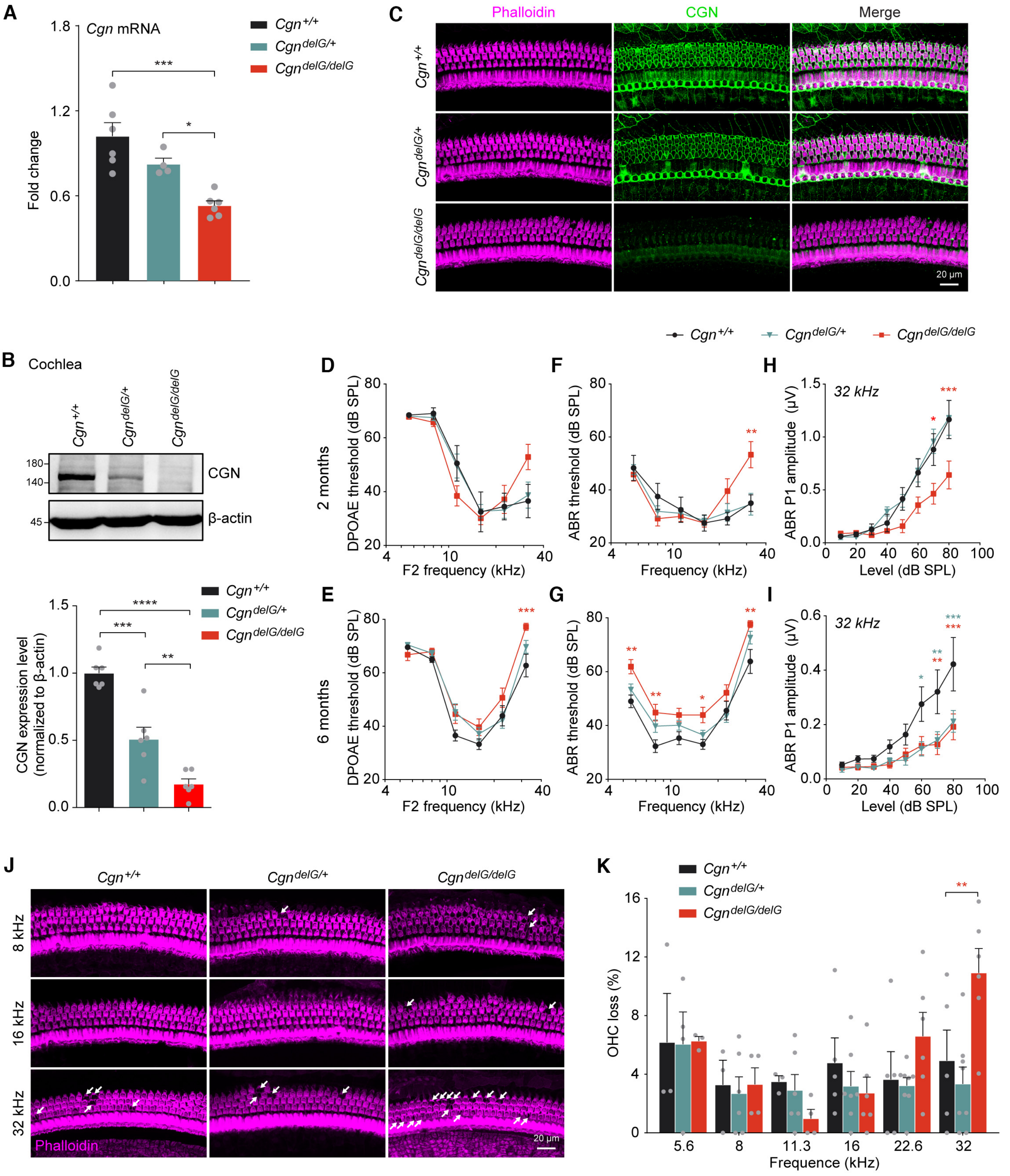

Figure 5.

deficiency would affect the formation of tight junctions (Appendix Fig S5D and E). Again, *Cgn* KD in MDCK cells did not affect expression of the tight junction markers (Appendix Fig S5F). Lastly, we examined the expression of ZO-1, a marker of tight junction formation and maintenance, in cochleae of the *Cgn*^{delG} and *Cgn-cKO* mice. The expression patterns of ZO-1 in cochleae of the two mutant mice

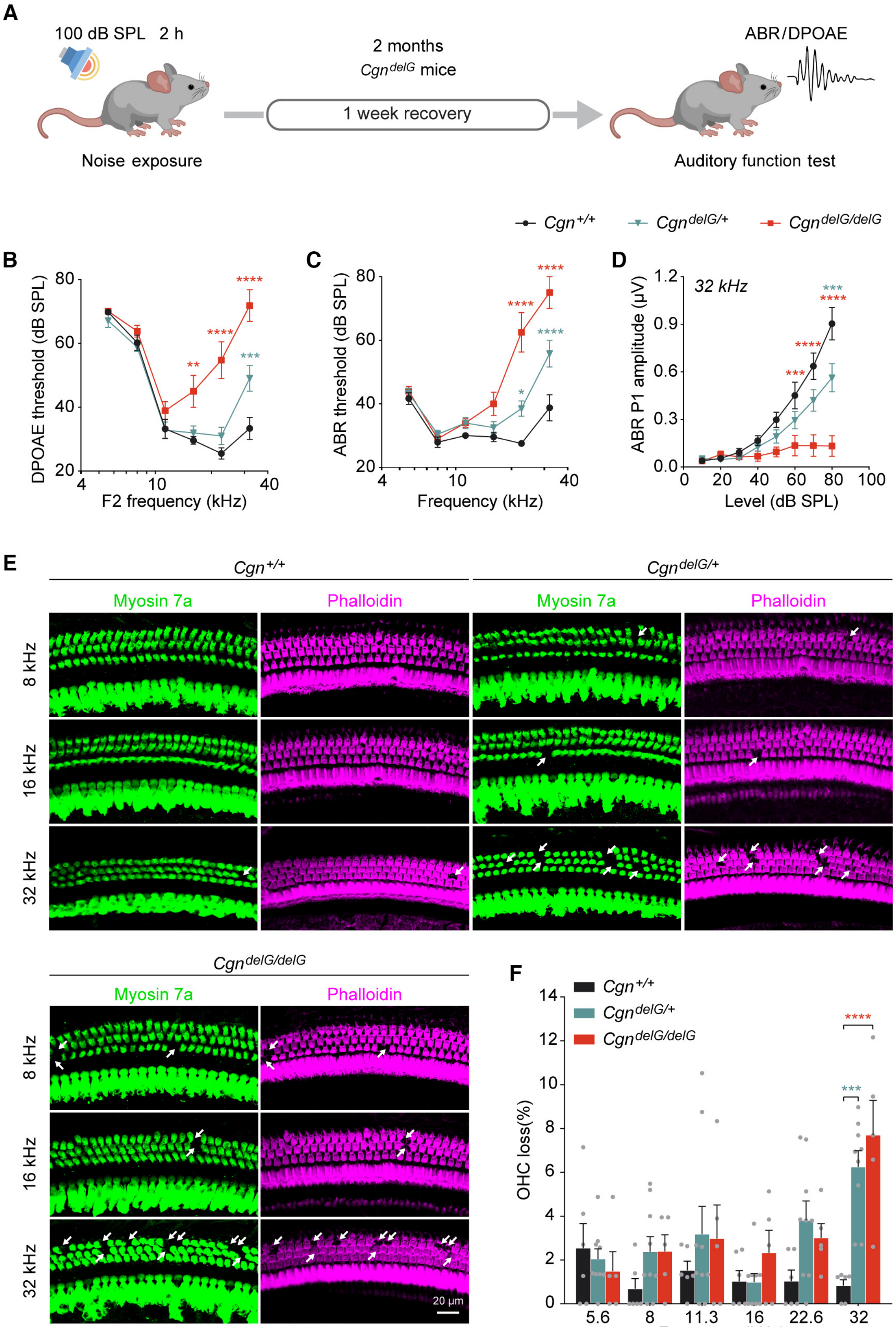
A
100 dB SPL 2 h
2 months
Cgn delG mice
ABR/DPOAE
1 week recovery
Noise exposure
Auditory function test
Cgn +/+
Cgn delG/+
Cgn delG/delG
B
DPOAE threshold (dB SPL)
F2 frequency (kHz)
C
ABR threshold (dB SPL)
Frequency (kHz)
D
32 kHz
ABR P1 amplitude (μV)
Level (dB SPL)
E
Myosin 7a
Phalloidin
8 kHz
16 kHz
32 kHz
20 μm
F
OHC loss(%)
Frequence (kHz)

Figure 6.

Figure 6. Exacerbated hearing loss and OHC degeneration in the Cgn^{delG} mice after noise exposure.

A A schematic diagram of noise exposure in control and Cgn^{delG} mice.

B–D DPOAE thresholds (B), ABR thresholds (C), and ABR P1 amplitudes at 32 kHz (D) of the 2-month-old Cgn^{delG} mice 1 week after noise exposure. Control $Cgn^{+/+}$ mice (circle), n = 6 mice (female = 3, male = 3); $Cgn^{delG/+}$ mice (triangle), n = 12 mice (female = 7, male = 5); $Cgn^{delG/delG}$ mice (square), n = 5 mice (female = 1, male = 4). $Cgn^{+/+}$ versus $Cgn^{delG/delG}$, * (red); $Cgn^{+/+}$ versus $Cgn^{delG/+}$, * (green).

E Whole mount immunofluorescence of the organ of Corti from the 2-month-old Cgn^{delG} mice 1 week after noise exposure. White arrows indicate lost OHCs labeled with F-actin (Phalloidin, magenta) or Myosin 7a (green).

F Percentage of OHC loss in noise exposed Cgn^{delG} mice (n = 5–9 cochleae).

Data information: Data are presented as mean ± SEM; two-way ANOVA was used in (B–D), one-way ANOVA was used in (F). **$P < 0.01$, ***$P < 0.001$, and ****$P < 0.0001$.

Source data are available online for this figure.

were also similar to the wild type (Appendix Fig S5G and H), suggesting that the auditory phenotypes of the *Cgn* mutant mice may not be caused by tight junction abnormalities.

Cingulin has been reported to regulate the localization of microtubules at tight junctions (Yano *et al*, 2018) and microtubules are widely expressed in the organ of Corti (Steyger *et al*, 1989). During inner ear development, each hair cell has a single kinocilium, which is a microtubule-based organelle. Kinocilia spontaneously degenerate postnatally and are thought to play an important role in the proper orientation of the actin filament-based stereocilia bundles in mature hair cells (Bieniussa *et al*, 2023). In mature hair cells, microtubules are mainly found in the apical and lateral membrane regions of the cell (Steyger *et al*, 1989). We examined microtubule expression in mature cochlea from 2-month-old *Cgn-cKO* and 7-month-old Cgn^{delG} mice. However, both types of *Cgn* mutant mice exhibited normal microtubule structures (Appendix Fig S6A and B). We also examined kinocilium position in immature cochlea of P0 Cgn^{delG} mice and found no significant difference between the wild-type and Cgn^{delG} mice (Appendix Fig S6C). These data indicate that abnormal expression of *CGN* had no obvious effects on microtubule expression patterns, and that the hearing impairment in *Cgn* mutant mice thus may not be related to microtubule alterations in the cochlea.

Cgn^{delG} mice display abnormal morphology of the hair cell cuticular plates

Cingulin is considered a classical actin-binding protein and its N-terminal globular head domain has high binding affinity for actin filaments, thus serving as an important contributor to epithelial structural formation (Cordenonsi *et al*, 1999; Yano *et al*, 2018). Therefore, we examined the effects of wild-type and mutant CGN on actin dynamics using the SRF-RE luciferase reporter system (Miralles *et al*, 2003) (Fig 7A). Expression of wild-type CGN significantly enhanced actin polymerization. Moreover, co-expression of wild-type CGN and RhoA, a member of Rho GTPase family, synergistically promote actin polymerization (Fig 7B). In contrast, the mutant CGN (p.L1110Lfs*17) showed significantly reduced actin polymerization activity (Fig 7C), likely due to its reduced expression levels and abnormal subcellular localization.

We also used fluorescent labeled-Phalloidin to visualize F-actin structures present in the cuticular plates and hair cell stereocilia in wild-type and mutant mice. Interestingly, in addition to the loss of the OHCs (visualized as loss of F-actin signals), the cuticular plates of the surviving OHCs appeared abnormal in the Cgn^{delG} cochleae (Figs 5J and EV4B). To further interrogate this morphological feature, we immunostained the organ of Corti for two cuticular plate-specific markers, LMO7 and Spectrin β II (Du *et al*, 2019; Liu *et al*, 2019). The areas of both cuticular plates and circumferential belts from three rows of OHCs were also quantified (Fig 7D). Bases on the LMO7 immunostaining, the morphology of the cuticular plates in $Cgn^{delG/delG}$ mice was significantly altered compared to the wild-type mice at both basal (32 kHz, Fig 7E and F) and middle (16 kHz, Appendix Fig S7A) cochlear regions. Quantitative results showed that the cuticular plate areas of $Cgn^{delG/delG}$ mice decreased (Fig 7G and Appendix Fig S7B), while the circumferential belt areas increased (Fig 7H and Appendix Fig S7C) relative to those of wild-type mice. Importantly, the relative areas of the cuticular plates were significantly reduced (Fig 7I and Appendix Fig S7D). Similar results were obtained with the Spectrin β II antibody (Fig 7J–N and Appendix Fig S7E–H). Together, these data suggest that the *Cgn* mutant reduces actin polymerization and alters the morphology of the actin-rich cuticular plates of the OHCs.

Figure 7. Effects of the CGN mutation on actin polymerization and hair cell cuticular plate morphology.

A Schematic diagram of actin polymerization activity detected by the SRF-RE luciferase reporter system.

B Effects of CGN and RhoA expression on actin polymerization (n = 3 biological replicates).

C Effect of WT and mutant CGN on actin polymerization (n = 3 biological replicates).

D Experimental approach to quantify areas of cuticular plates, circumferential belts, and relative cuticular plate areas. The image shows apical surface of an OHC from a wild-type mouse. Phalloidin (magenta), LMO7 (green).

E–I Whole mount immunofluorescence with LMO7 antibody on 2-month-old Cgn^{delG} mice at 32 kHz cochlear region (E, F), areas of the OHC cuticular plates (G), areas of the OHC circumferential belts (H) and relative areas of the OHC cuticular plates (I) (n = 159–187).

J–N Whole mount immunofluorescence with Spectrin β II antibody on 2-month-old Cgn^{delG} mice at 32 kHz cochlear region (J, K), areas of the OHC cuticular plates (L), areas of the OHC circumferential belts (M), and relative areas of the OHC cuticular plates (N) (n = 56–62).

Data information: Data are presented as mean ± SEM; unpaired Student's t-test was used in (B, C, G–I and L–N). **$P < 0.01$, ***$P < 0.001$, and ****$P < 0.0001$.

Source data are available online for this figure.

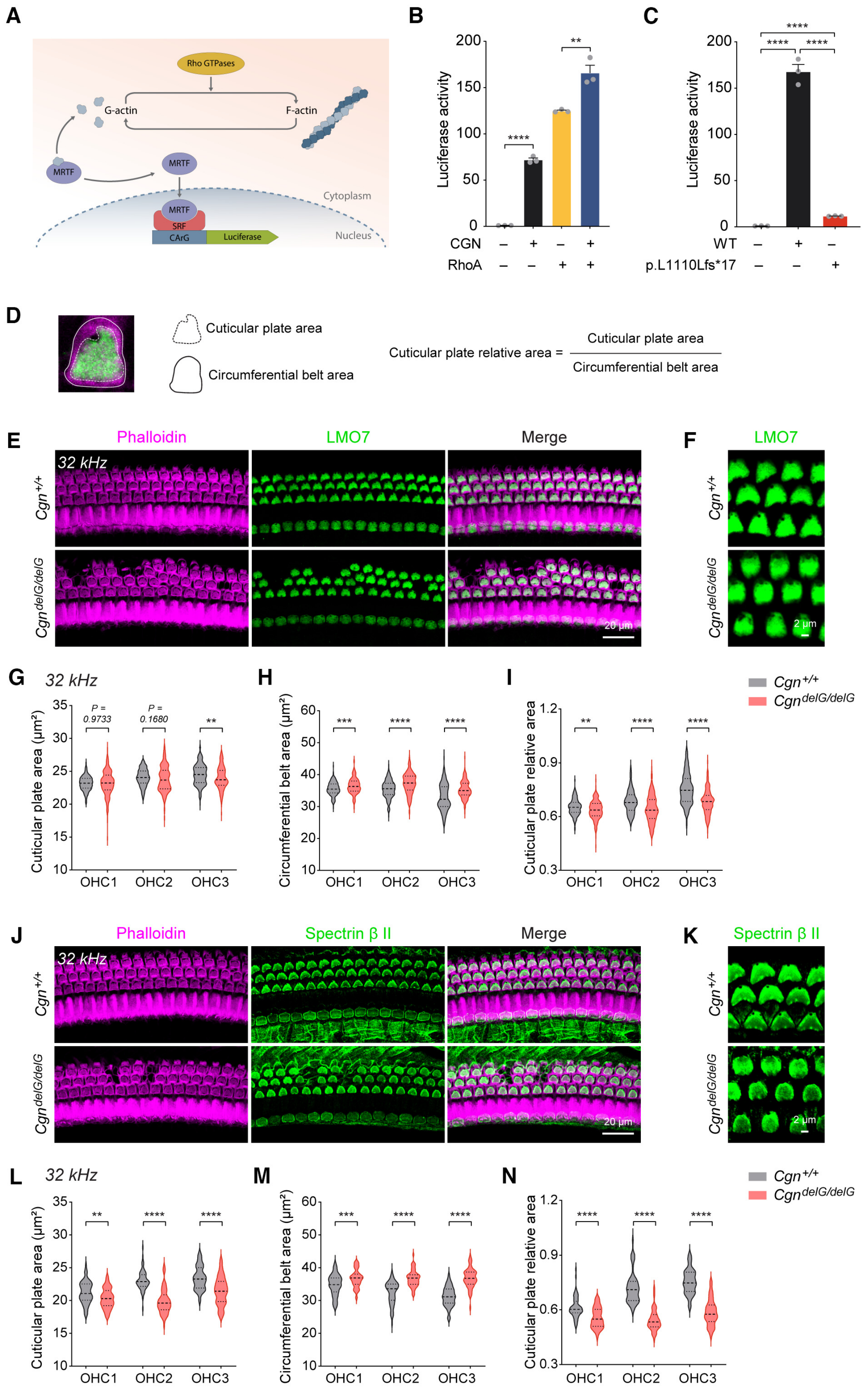

Figure 7.

© 2023 The Authors

Cgn[delG] mice display abnormal hair cell bundle morphology

As the actin filaments of stereocilia bundles are rooted in the cuticular plate, we predicted that the altered cuticular plates in *Cgn* mutant mice might affect the morphology of their associated bundles. Scanning electron microscopy (SEM) revealed a sporadic OHC bundle loss at 32 kHz cochlear region in 2-month-old *Cgn*$^{delG/delG}$ but not *Cgn*$^{+/+}$ mice (Fig 8A), consistent with loss of OHCs in these mice observed previously (Fig 5). The morphology of the remaining hair bundles in *Cgn*$^{delG/delG}$ cochlea is largely indistinguishable from that in *Cgn*$^{+/+}$ cochlea, with neither orientation deficits nor stereocilia degeneration detected in either IHCs or OHCs (Fig 8A–C). Interestingly, the demarcation of outer hair cell apical surfaces appeared to be more rounded in the *Cgn*$^{delG/delG}$ mice (Fig 8C), supporting the notion that cuticular plate morphology was altered based on LMO7 and Spectrin β II immunostainings (Fig 7).

In *Cgn*$^{+/+}$ mice, OHC hair bundles were organized into the normal V-shaped morphology, with an increase in bundle opening angles from apical (8 kHz) to basal (32 kHz) cochlear regions (Fig 8C, left). In contrast, the opening angles of the V-shaped OHC bundles were significantly reduced in *Cgn*$^{delG/delG}$ mice (Fig 8C, right). To quantify the opening angles of the OHC bundles, the bundle width and the height of the bundle arc were measured for individual OHCs, and the width index calculated (bundle width/height of bundle arc) (Fig 8D). The width indices of bundles from all three rows of OHCs were significantly smaller in the *Cgn*$^{delG/delG}$ mice (Fig 8C, right) than those of *Cgn*$^{+/+}$ mice at 8 kHz (Fig 8E), 16 kHz (Fig 8F), or 32 kHz (Fig 8G). Together, the data suggest that the altered cuticular plates affect the morphology of hair bundles in *Cgn* mutant OHCs, which may contribute to the hearing loss observed in *Cgn* mutant mice and the affected human patients.

Discussion

Cingulin (CGN) is a cytoskeleton-associated protein and an important component of the tight junction in vertebrate epithelial cells; however, association of *CGN* mutations with human diseases has

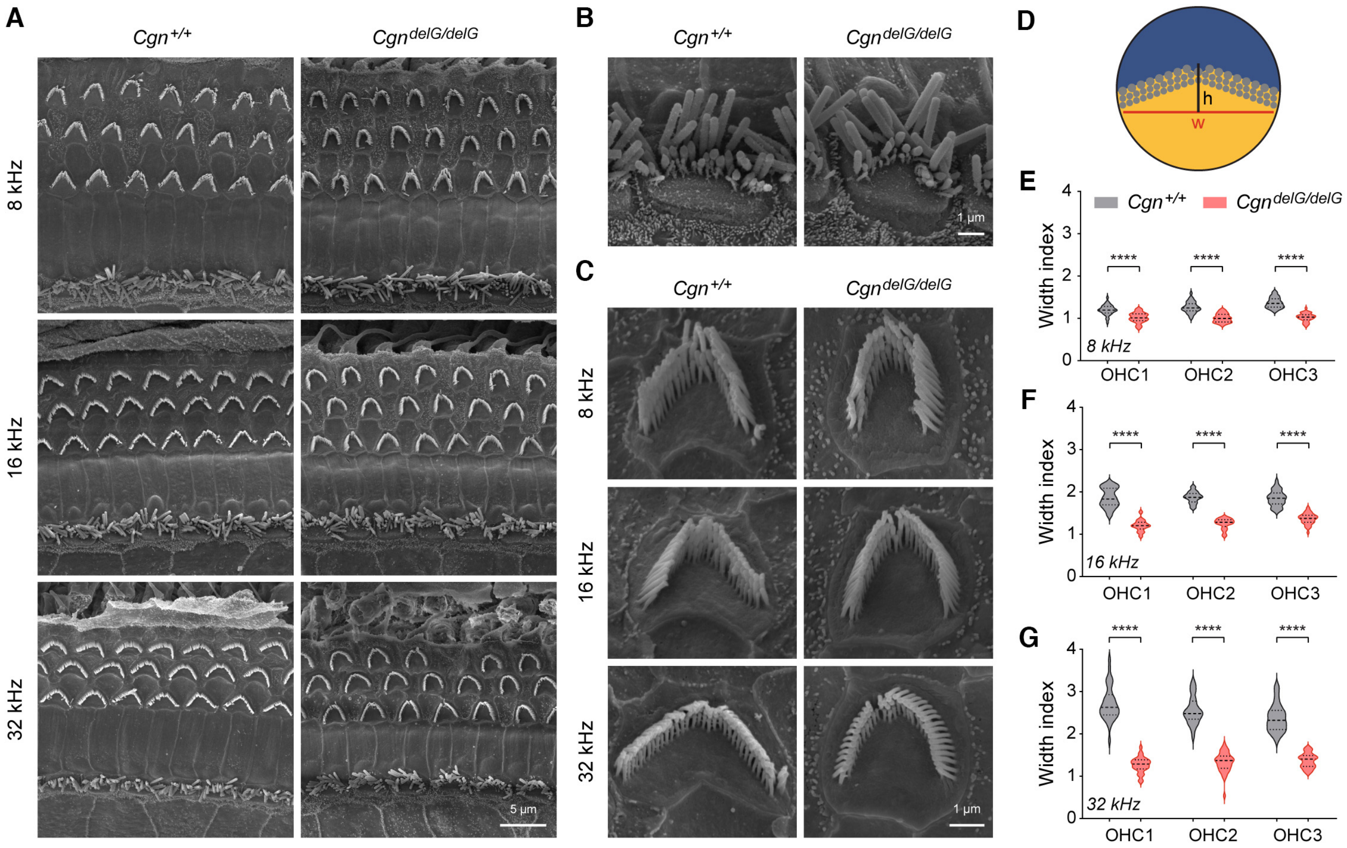

Figure 8. Effects of the CGN mutation on hair bundle morphology.

A Low-magnification SEM images of hair bundles from 2-month-old *Cgn*$^{+/+}$ and *Cgn*$^{delG/delG}$ mice.

B High-magnification SEM images of IHC hair bundles from 2-month-old *Cgn*$^{+/+}$ and *Cgn*$^{delG/delG}$ mice.

C High-magnification SEM images of OHC hair bundles from 2-month-old *Cgn*$^{+/+}$ and *Cgn*$^{delG/delG}$ mice. Shown are hair bundles of the second-row OHCs (OHC2).

D Width index of each hair bundle is calculated as the bundle width (w) divided by the height of the bundle arc (h).

E–G Width indices of OHC hair bundles at (E) 8 kHz, (F) 16 kHz, and (G) 32 kHz cochlear regions were calculated from SEM images. n = 28–37 from 6 mice (*Cgn*$^{+/+}$) or 3 mice (*Cgn*$^{delG/delG}$).

Data information: Data are presented as mean ± SEM; unpaired Student's t-test was used in (E–G). ****$P < 0.0001$.
Source data are available online for this figure.

© 2023 The Authors

not been reported. Here, we identify a novel *CGN* variant (*CGN delG*) co-segregating with affected individuals in an ADNSHL family. CGN is normally enriched at the apical cuticular plates and circumferential belts of cochlear hair cells and mediates actin polymerization. The *CGN delG* disease variant abolishes the expression and localization of CGN protein, altering the morphology of cuticular plates and stereocilia bundles in hair cells, and resulting in progressive and noise-sensitive OHC loss in mutant mouse models. These data, together with our human genetic and sequencing data, strongly supports the *CGN delG* variant as the causative mutation underlying hearing loss in this Chinese pedigree.

In the sensory epithelium of the mammalian inner ear, the apical surface of hair cells has an organelle rich in a dense actin filament network (named "cuticular plate"), where the roots of the stereocilia bundles are inserted (Liberman, 1987; DeRosier & Tilney, 1989; Pollock & McDermott, 2015). A structurally separate group of actin filaments form the circumferential belts and encircle the lateral edges of the cuticular plates (Hirokawa *et al*, 1982; Jaeger *et al*, 1994). Between the cuticular plate and the circumferential belt, there is a region without F-actin, known as the pericuticular necklace (Raphael *et al*, 1994). The morphology of these apical structures is dynamically remodeled during postnatal development in mouse, in which an immature non-convex apical circumference is molded to the mature V-shape of the overlying hair bundle in cochlear OHCs (Etournay *et al*, 2010). Interestingly and consistent with our findings, this remodeling is significantly impaired in mouse mutants with defective hair bundles (such as *Cdh23*$^{-/-}$, *Ush1c*$^{-/-}$, *Myo7a*$^{SB/SB}$, *Whrn*$^{wi/wi}$, and *Myo15*$^{ash2/sh2}$), suggesting potential interplay between morphogenesis of hair bundles and the underlying cuticular plates (Etournay *et al*, 2010). Actomyosin cytoskeleton tension is thought to play a pivotal role in these processes, although the regulatory mechanisms are largely unknown (Etournay *et al*, 2010). Our study thus provides an important piece to the puzzle on how the delicate morphology of cuticular plates on the apical hair cells may be maintained. Whether CGN also regulates the remodeling of hair cell circumferential belts and cuticular plates during development remains to be addressed.

Our observation that CGN regulates actin polymerization is consistent with previous *in vitro* studies (Cordenonsi *et al*, 1999; D'Atri & Citi, 2001; D'Atri *et al*, 2002; Mangan *et al*, 2016; Yano *et al*, 2018). Two main types of actin molecules, β-actin and γ-actin, are expressed in hair cells (Drummond *et al*, 2012). β-actin is mainly distributed in stereocilia, while γ-actin is the main component of cuticular plates (Drummond *et al*, 2012). *γ-actin-KO* mice show progressive high-frequency hearing loss at 16 weeks of age (Belyantseva *et al*, 2009), while *β-actin* hair cell *cKO* mice show similar high-frequency hearing loss, but subsequent progression is slower (Perrin *et al*, 2010). Interestingly, loss of CGN expression has recently been shown to affect the localization of γ-actin in MDCK cells at cell junctions (Rouaud *et al*, 2023). Based on our findings, we speculate that the restricted expression of CGN at the apical regions of hair cells is responsible for the morphology and tension of the cuticular plates by regulating γ-actin dynamics.

It is worth to note that auditory phenotypes of the *Cgn* mutant mice are milder than those observed in human patients. Such differences are not uncommon in monogenic disorders in which the disease symptoms and severities are modifiable by environmental factors (Rossetti & Harris, 2007; Singer, 2015; Sacco & Milner, 2019). We previously generated a *Pou4f3* knockin mouse model for DFNA15, another human ADNSHL locus (Vahava *et al*, 1998), in which the progressive loss of hearing and OHCs is significantly modified by aging, noise exposure, and genetic background (Zhu *et al*, 2020). The *Cgn*^{delG} mice in this study also display agedepedentn progressive hearing loss and are more susceptible to noise exposures, reminiscent of the DFNA15 mouse model. In addition, hearing loss in DFNB59 (*PVJK*) and *Foxo3*-KO mouse models is also greatly influenced by prior moderate noise exposure (Delmaghani *et al*, 2015; Gilels *et al*, 2017). Lastly, *P2rx2*-null mice, which model human DFNA41 (*P2RX2*), only show mild hearing loss at 17 months of age but are susceptible to noise exposure at 3 months of age (Yan *et al*, 2013). Although potential functional differences of CGN in mice and human cochleae cannot completely be excluded, these studies highlight the importance of gene–environment interactions in the pathogenesis and severity of progressive genetic hearing loss.

In summary, this study reports human *CGN* as a novel deafness gene in ADNSHL and provides both *in vitro* and *in vivo* evidence on the mechanisms of the *CGN* variant causing hearing loss in human and mouse. Furthermore, the findings highlight the important physiological roles of CGN in maintaining the cochlear hair cell cuticular plate morphology and auditory function.

Materials and Methods

Subjects and clinical evaluations

We recruited a three-generation Chinese Han family with postlingual autosomal-dominant non-syndromic hearing loss. Twelve members (seven affected and five unaffected) participated in the present study, informed consent was signed by all participating subjects. The experiments conformed to the principles set out in the WMA Declaration of Helsinki and the Department of Health and Human Services Belmont Report. This study was approved by the ethics committee of Nanjing Drum Tower Hospital, the Affiliated Hospital of Nanjing University Medical School (2019-170 and 2021-122-02).

Hearing levels of all participating members were measured by pure tone audiometry. For affected members, a complete medical history and physical examination were performed to exclude the possibility of environmental causes or syndromic hearing loss. For the proband, additional auditory evaluations were performed including distortion product otoacoustic emission (DPOAE), auditory steady-state response (ASSR), auditory brainstem response (ABR), temporal bone high-resolution computed tomography scanning (HRCT), and internal acoustic meatus magnetic resonance imaging (MRI).

Exome sequencing (ES) and linkage analysis of the Chinese family

All the 12 participating members were included in the ES study. Genomic DNA samples were extracted from whole blood samples of the participating members.

The exomes and flanking intronic regions from whole blood DNA samples were captured by Agilent SureSelect Human All Exon Kit (Agilent Technologies). The captured DNA was sequenced on

© 2023 The Authors

Illumina HiSeq 4000 sequencing platform (Illumina). Bioinformatics were aligned to NCBI build37/hg19 assembly using the BWA (0.7.12) software. Each sample was covered to an average sequencing depth of at least 100x. SNPs and indels were identified using GATK HaplotypeCaller software. Candidate pathogenic variants were defined as nonsense, missense, splice-site, and indel variants with allele frequencies of 0.001 or less in public variant databases dbSNP, 1000 Genomes, ESP6500, nci60, GnomAD and in disease database of COSMIC, Clinvar, OMIM, GWAS. Genotypes distributed in every 0.3 cM of genomic region were chosen for calculation of the logarithm of odds (LOD) scores using the Merlin v. 1.1.2 parametric linkage analysis package.

ES of selected Spanish subjects

Exome sequencing was performed on the 96 Spanish index cases using the Agilent SureSelect V6 exome capture-based method in a NovaSeq6000 platform (Illumina, 150 PE, 18Gb/sample). The sequence data were mapped against the human genome sequence (build GRCh37/hg19), and data analysis was performed using the DNANEXUS' software that enables the single nucleotide variations (SNVs) and the copy number variation (CNV) analysis of the targeted exonic sequences. The analysis of the variant calling files (VCF) was restricted to the cingulin gene (CGN) and variant prioritization was carried out using a custom filtering strategy (Morin *et al*, 2020).

Sanger sequencing

Possible pathogenic variants from the ES analysis were evaluated by computational tools, including Mutation Taster, PolyPhen-2, and SIFT. Candidate pathogenic variants were further genotyped for the 12 family members by Sanger sequencing.

Mouse models and genotyping

Cgn flox mice, named Cgn^{fl}, were generated by CRISPR-Cas9 genome-editing technology on C57BL/6J background, by inserting LoxP fragments at both ends of the exon 6 and exon 21 of mouse *CGN* (ENSMUSG00000068876) (Gempharmatech Inc, China). Genotypes of Cgn^{fl} mice were identified by a primer pair targeting the LoxP insert (forward primer: 5′-CTG GGC TGG GCT CTT CTA TGT AG-3′, reverse primer: 5′-CTA GGG ATT GAA CCC AGA ACT TGA C-3′).

Cgn disease mutation knockin mice, named Cgn^{delG}, were generated by CRISPR-Cas9 genome-editing technology on C57BL/6J background. A repair DNA harboring the G-base deleted in the identified human patients was used as donor template. The mutant allele was amplified using a specific forward primer (5′-ACT CTG CTT AGC TAA CAT TAC GT-3′) paired with the reverse primer (5′-GCA GGG CTG GCT GGG ATC CT-3′). The wildtype- allele was genotyped in a similar manner using the same reverse primer combined with a specific forward primer for the wildtype- allele (5′-ACT CTG CTT AGC TAA CCC TGA GG-3′).

$Pou4f3^{EGFP\text{-}creER}$ knockin mice were generated as described previously (Du *et al*, 2020). Genotypes of $Pou4f3^{EGFP\text{-}creER}$ mice were identified by three primers (forward primer: 5′-GTG GGG GAG AGG GGA GGC AG-3′, the wildtype allele-specific reverse primer: 5′-GAG AGA GCG CGG GGG AGA CA-3′, the mutant allele-specific reverse primer: 5′-CGG TTC ACC AGG GTG TCG CC-3′). *Cgn* hair cell-specific conditional knockout mice ($Cgn^{fl/fl}$;$Pou4f3^{creER/+}$), named *Cgn-cKO*, were generated by mating $Cgn^{fl/fl}$ with $Pou4f3^{EGFP\text{-}creER}$ mice. For Cre recombinase activation, pups at P3 to P6 were intraperitoneally injected daily with 33 mg/kg tamoxifen (T5648, Sigma) dissolved in corn oil.

C57BL/6J were purchased from Gempharmatech Inc, China. Both male and female mice were used in this study. Animals were assigned to control or experimental groups based on their genotypes. For noise exposure experiments, animals of the same genotype were randomly assigned to control or noise exposure groups. Genotypes of the animals were blinded to the experimenters. All animal procedures were approved by the Institutional Animal Care and Use Committee of Model Animal Research Center of Nanjing University, China with protocol approval number #WGQ04.

Plasmid constructions

Human *CGN* (NM_020770.3) and p.Leu1110Leufs*17 variant (C-terminal Flag-tagged or N-terminal EGFP-tagged) was cloned into pcDNA3.1 vector using EcoRV (R3195V, New England Biolabs) site. Mouse *Cgn* (NM_020770.3) (C-terminal Flag-tagged) was also cloned into pcDNA3.1 vector using EcoRV site. All mammalian expression plasmids were amplified in DH5α bacteria. All constructs were verified by sequencing.

Cell culture and transfection

MDCK (ATCC: CCL-34) and COS-7 (ATCC: CRL-1651) cells were cultured in Dulbecco's Modified Eagle Medium (DMEM) (12800017, Gibco) supplemented with 10% fetal bovine serum (FBS) (40130ES76, Yeasen) and penicillin–streptomycin (P/S) (E607011-0100, Sangon Biotech). CACO2 (ATCC: HTB-37) cell was cultured in DMEM supplemented with 20% FBS and P/S. HEK293T (ATCC: CRL-3216) cell was cultured in DMEM supplemented with 10% FBS, GlutaMAX (35050079, Gibco), non-essential amino acid (NEAA) (11140050, Gibco), sodium pyruvate (11360070, Gibco), and P/S. Cell lines were obtained from ATCC and were tested mycoplasma-negative prior to experiments. Cell lines were transfected with indicated plasmids using Hieff Trans™ Liposomal Transfection Reagent (40802ES03, Yeasen) following the manufacturer's instructions.

Reverse transcription quantitative polymerase chain reaction (RT-qPCR) analyses

Cultured cells or mouse tissues were homogenized and the total RNA extracted by RNAiso Plus kit (9109, Takara). The quantity and purity of samples were examined by determining absorbance at 260/280 nm by Nanodrop (Thermo Scientific). Reverse transcription reactions were performed by HiScript III RT SuperMix for qPCR (+gDNA wiper) (R323-01, Vazyme). QPCR was performed with AceQ qPCR SYBR Green Master Mix (Q111-02, Vazyme) on a LightCycler® 96 Instrument (Roche). QPCR reactions were performed with the primers listed in Appendix Table S2. Expression of target genes was normalized to the internal reference genes (*GAPDH* for human and mouse cells/tissues, *Actb/β-actin* for the canine MDCK

© 2023 The Authors

cells). Fold changes of expression were normalized to control treatments or wildtype CGN control samples.

Western blot analysis

Tissues were freshly isolated after sacrificing the mice and grinded into powders in liquid nitrogen. Cultured cells were carefully rinsed three times with phosphate-buffered solution (PBS). Tissue powders or cells were extracted and homogenized with the lysis buffer containing 0.2% SDS (cells, lung, liver, brain, intestine, kidney) or 1% SDS (cochlea), 50 mM Tris–HCl pH 7.4, 150 mM NaCl, 1 mM ethylene diamine tetra-acetic acid (EDTA) pH 8.0, 1% Triton X-100 (A110694-0500, Sangon Biotech) and 1 mM phenylmethylsulfonyl fluoride (PMSF) (ST005, Beyotime) containing the cOmplete Protease Inhibitor Cocktail (11697498001, Roche) at 4°C for 10 min.

Proteins were subjected to SDS–PAGE and transfer to PVDF membrane and analyzed by following antibodies: rabbit anti-Cingulin (1:1,000, 21369-1-AP, Proteintech), mouse anti-Cingulin (1:1,000, sc-365264, Santa Cruz), mouse anti-Flag (1:2,000, 30503ES60, Yeasen), rabbit anti-EGFP (1:2,000, 31002ES60, Yeasen), rabbit anti-ZO-1 (1:1,000, 21773-1-AP, Proteintech), rabbit anti-Occludin (1:1,000, 13409-1-AP, Proteintech), mouse anti-β-actin (1:20,000, 4970, Cell Signaling Technology) antibodies followed by incubation in HRP conjugated anti-Rabbit (1:5,000, BS13278, Bioworld) or anti-mouse (1:5,000, BS12478, Bioworld) secondary antibody for 2 h at room temperature (RT). The signals were visualized using ECL substrate (180-5001, Tanon) on an automatic chemiluminescence/fluorescence image analysis system (Tanon 4600, Tanon).

Cgn shRNA lentiviral vectors and lentivirus packaging

For *Cgn* gene silencing, previously described target sequences for silencing canine CGN (Guillemot & Citi, 2006) were cloned into pLKO1, a lentiviral vector with puromycin resistance screening label, using EcoRI (R3101V, New England Biolabs) and AgeI (R3552S, New England Biolabs) sites. The lentiviral particles were produced by co-transfecting each pLKO1 vector with other two lentiviral packaging helper plasmids (pSPAX2 package and pMD2.G envelope plasmids) in HEK293T cell. The supernatant was collected and concentrated by ultracentrifugation. The *Cgn* shRNA lentivirus were used to infect MDCK cells and puromycin was used to select infected cells to obtain stable shRNA-expressing cell lines. *Cgn* knockdown efficiency in MDCK cell was determined by RT-qPCR, Western blot and immunofluorescence.

SRF-RE dual-luciferase reporter assays

The pGL4.34[luc2P/SRF-RE/Hygro] vector contains a serum response factor (SRF) response element (SRF-RE) that drives transcription of the luciferase reporter gene luc2P in response to the activation of serum response factors through multiple signaling pathways, including RhoA GTPase activation. HEK293T cells in 24-well plates were transfected with plasmids overnight as follows: pGL4.34[luc2P/SRF-RE/Hygro] (Promega), pRL-TK, and CGN (WT or mutant), with or without co-transfecting the constitutively active RhoA. Transfected cells were serum starved for 6 at 18 h after transfection. Starved cells were lysed and the supernatants collected. The dual-luciferase assay kit (RG089M, Beyotime) was used to detect SRF activity, an indirectly representation of actin polymerization levels.

Immunofluorescence

Isolated mouse cochleae were fixed in 4% paraformaldehyde (PFA) in PBS for 2 h at RT. Samples were then decalcified with 5% (w/v) EDTA for 4 days at RT. Decalcified cochleae can be stored in PBS at 4°C. After micro-dissection of the cochleae, samples were permeabilized by freeze-thawing in 30% sucrose and blocked in 5% normal horse serum (NHS) (008-000-121, Jackson ImmunoResearch) with 0.3% Triton X-100 in PBS for 1 h. Then, the samples were incubated with primary antibodies (diluted in 1% NHS with 0.3% Triton X-100 in PBS) overnight at 4°C. Next day, samples were incubated with corresponding secondary antibodies for 2 h at RT and mounted to cover slides.

Transfected cells were fixed on ice in 2% PFA for 15 min and then blocked in 5% NHS with 0.3% Triton X-100 in PBS for 1 h at RT. Then, samples were incubated with primary antibodies (diluted in 1% NHS with 0.3% Triton X-100 in PBS) overnight at 4°C. Next day, samples were incubated with corresponding secondary antibodies for 2 h at RT.

In this study, the primary antibodies used were rabbit anti-Cingulin (1:200, 21369-1-AP, Proteintech; 1:200, A15489, ABclonal), mouse anti-Cingulin (1:200, sc-365264, Santa Cruz), rabbit anti-ZO-1 (1:200, 21773-1-AP, Proteintech), mouse anti-Parvalbumin (1:500, sab4200545, Sigma), rabbit anti-Myosin 7a (1:500, 25-6790, Proteus Biosciences), rabbit anti-Ctbp2 (1:200, 612044, Millipore), mouse anti-LMO7 (1:50, sc-376807, Santa Cruz), mouse anti-Spectrin β II (1:100, sc-136074, Santa Cruz), mouse anti-Pou4f3 (1:200, sc-81980, Santa Cruz), mouse anti-Pan tubulin (1:1,000, 3873, Cell Signaling Technology), mouse anti-Acetylated tubulin (1:1,000, T6793, Sigma), mouse anti-Flag (1:1,000, 30503ES60, Yeasen), and rabbit anti-EGFP (1:400, 31002ES60, Yeasen). The secondary antibodies used were Alexa Fluor® 488 AffiniPure Goat Anti-Rabbit IgG (H+L) (1:500, 111-545-003, Jackson ImmunoResearch), Alexa Fluor® 594 AffiniPure Goat Anti-Rabbit IgG (H + L) (1:500, 111-585-003, Jackson ImmunoResearch), Alexa Fluor® 647 AffiniPure Goat Anti-Rabbit IgG (H+L) (1:500, 111-605-003, Jackson ImmunoResearch), Alexa Fluor® 488 AffiniPure Goat Anti-Mouse IgG (H+L) (1:500, 115-545-003, Jackson ImmunoResearch), Alexa Fluor® 594 AffiniPure Goat Anti-Mouse IgG (H+L) (1:500, 115-585-003, Jackson ImmunoResearch), Alexa Fluor® 647 AffiniPure Goat Anti-Mouse IgG (H+L) (1:500, 115-605-003, Jackson ImmunoResearch), Alexa Fluor™ 488-conjugated goat anti-mouse (IgG2b) (diluted 1:500; A21141, Life Technologies), Alexa Fluor™ 568-conjugated goat anti-mouse (IgG2b) (diluted 1:500; A21144, Life Technologies), and Alexa Fluor™ 568-conjugated goat anti-mouse (IgG1) (1:500; A21124, Life Technologies). F-actin and nucleus were visualized with Rhodamine-conjugated Phalloidin (1:1,000, PHDR1, Cytoskeleton), Actin-Tracker Green (1:500, C1033, Beyotime Biotechnology), and DAPI (1:1,000, 28718-90-3, Roche), respectively.

ImageJ (https://imagej.net/software/fiji) measure line plugin was used to map cochlear length to cochlear best frequencies. Samples were examined using a Leica SP5 confocal microscope (Leica TCS SP5, Leica Microsystems), Zeiss LSM 800 confocal microscope (ZEN 2.1, Carl Zeiss) or inverted fluorescence microscope (XD, SOPTOP). ImageJ was used for image processing.

© 2023 The Authors

Scanning electron microscopy (SEM)

Temporal bones of 2-month-old mice were fixed with 2.5% glutaraldehyde in 0.1 M phosphate buffer at 4°C overnight. The cochleae were dissected out and postfixed with 1% osmium tetroxide in 0.1 M phosphate buffer at 4°C for 2 h. The samples were then dehydrated in ethanol, followed by critically point drying using a Leica EM CPD300 (Leica, Germany). After sputter coating with platinum to a thickness of 15 nm using a Cressington 108 sputter coater (Cressington, United Kingdom), the samples were imaged with a Quanta250 field-emission scanning electron microscope (FEI, The Netherlands). The bundle width is defined as the distance between the two ends of each hair bundle. The height of the bundle arc is defined as the distance from the vertex to the line connecting the two ends of each hair bundle. Width index of each hair bundle is calculated as the bundle width divided by the height of the bundle arc.

DPOAE and ABR measurements

Mice were anesthetized by intraperitoneal injections of 375 mg/kg Avertin (Sigma) before ABR and DPOAE tests.

For ABR measurements, three needle electrodes, A, B, and G, were placed under the skin: A at the dorsal midline between the two ear flaps, B behind the right pinna, and G at the base of the tail. ABR potentials were evoked with 5-ms tone pips (0.5-ms rise-fall, with a cos2 envelope, at 33/s) delivered to the eardrum at log-spaced frequencies at 5.6, 8, 11.3, 16, 22.6, and 32 kHz. The responses were amplified (10,000×) and filtered (0.3–3 kHz) with an analog-to-digital board in a PC-based data-acquisition system. Sound pressure levels (SPLs) were raised at 5 dB-step from 10 to 80 decibels (dB). At each SPL, 1,024 responses were averaged (with alternating polarity) after "artifact rejection". DPOAEs were measured immediately following ABR tests. The DPOAE signal in response to primary and secondary tones with frequencies f1 and f2 respectively was recorded at the third frequency (2 × f1)−f2, with f2/f1 = 1.2, and the f2 level 10 dB lower than the f1 level. SPLs at the ear canal was amplified and digitally sampled at 4-ms intervals. DPOAE thresholds were defined as the f1 level required to evoke a response at −10 dB SPL. Both DPOAE and ABR recordings were carried out using EPL cochlear function test suite software (Mass Eye and Ear, Boston, MA, USA). ABR peak 1 amplitudes were analyzed with ABR peak analysis software (Mass Eye and Ear).

Noise exposure

Both *Cgn* wild-type and mutant mice were subjected to noise exposure at 2 months of age. Individual mouse was housed within small cells in a subdivided cage on a rotation station, which is suspended in a reverberant chamber. Noise exposure experiment was carried out using LabState software (AniLab Software & Instruments, China) to produce 2–20 kHz broadband noise at 100 dB for 2 h.

Rotarod tests

Vestibular function of 2-month-old wildtype and mutant mice was performed using a rotarod system (ZB-200, Chengdu Techman Software, China). Mice were trained each day for 3 consecutive days at 15 round per minute (rpm) for 240 s prior to the experiment. Mice were subjected to three different rotarod test protocols: (i) 12 rpm fixed speed for 240 s; (ii) 20 rpm fixed speed for 240 s; and (iii) 0–44 rpm accelerating speed with 1 rpm/s and maintained at 44 rpm from 44 to 60 s. All three test protocols were performed for three times with 30–60 m resting time in between. The latencies to fall from the rotarod were then recorded and presented as averages of the three tests for each test protocol.

Statistical analysis

Statistical tests were performed using GraphPad Prism 9. Specific statistical tests used in each experiment and replicates were described in figure legends. Distribution of the data was assumed to be normal, but this was not formally tested. No statistical methods were used predetermine sample sizes, but our sample sizes are similar to those reported in our previous publications (Wan *et al*, 2014; Wan & Corfas, 2017; Zhu *et al*, 2020). The genotypes of the control and experimental animals were blinded to the investigators until the completion of data acquisition and analysis. For noise exposure experiments, mice with the same genotype were randomly assigned to control or noise exposure conditions.

The paper explained

Problem
Non-syndromic hearing loss (NSHL) is the most common hereditary sensory impairment. Half of the congenital deaf patients are associated with genetic defects. While more than 100 deafness genes have been identified, the etiologies and pathological mechanisms in a great number of deaf patients are still unknown. Cingulin (CGN) is a cytoskeleton-associated protein and an important component of the tight junction in vertebrate epithelial cells; however, associations of *CGN* mutations with human diseases have not been reported.

Results
We report a novel *CGN* variant (c.3330delG, p.L1110Lfs*17) that co-segregates with the deaf patients in an autosomal dominant NSHL family. CGN is preferentially enriched at the apical cuticular plates and circumferential belts of the hair cells. The CGN mutation abolishes the expression and subcellular localization of CGN protein and fails to promote actin polymerization. A knockin mouse model carrying the disease mutation shows altered morphology of the actin-enriched cuticular plates and hair bundles of sensory hair cells. Importantly, the *Cgn* mutation results in progressive and noise-sensitive hearing impairment and hair cell degeneration in the knockin mouse model.

Impact
Identifying novel deafness genes and the underlying pathogenic mechanisms are essential to personalized treatment for genetic hearing loss. Combining human genetics, cell culture, and animal model studies, we demonstrate that *CGN* is a novel deafness gene and plays an important role in the maintenance of hair cell cuticular plate and auditory function. This work not only provides mechanistic insights into development and maintenance of hair cell cuticular plates but also affords an experimental basis for genetic counseling and personalized therapeutics to deaf patients carrying *CGN* mutations.

© 2023 The Authors

Data availability

Exome sequencing data of the Chinese family are available on Sequence Read Archive (SRA) ID: SRP449242 (https://www.ncbi.nlm.nih.gov/sra?term=SRP449242).

Expanded View for this article is available online.

Acknowledgements
We would like to thank Dr Renjie Chai's laboratory (Southeast University, China) for assistance in the noise exposure experiments and Dr Cheng Deng (Sichuan University, China) for providing the pGL4.34[luc2P/SRF-RE/Hygro] plasmid. We also thank Dr Gabriel Corfas and Dr David Kohrman (University of Michigan, USA) for critical reading and language editing of the manuscript. This work is supported by National Natural Science Foundation of China (82201291 to G-JZ, 82171138 and 81970888 to GW, 82171145 to XQ, 81870721 to JC, 81970884 and 82192862 to XG, 82192861 and 82071051 to ZX), Fundamental Research Funds for the Central Universities (021414380533 to GW), Natural Science Foundation of Jiangsu Province (BK20220189 to G-JZ, BK20200133 to CZ and BK20220188 to QL), Special Foundation for Health Science and Technology Development of Nanjing (YKK21109 to G-JZ, YKK20069 to HZ), Clinical Research Foundation of Drum Tower Hospital affiliated to Nanjing University Medical School (2021-LCYJ-PY-04 to G-JZ). The work has also received funding from the Regional Government of Madrid (B2017/BMD3721 to MAM-P) and from Instituto de Salud Carlos III, cofounded with the European Regional Development Fund "A way to make Europe" within the National Plans for Scientific and Technical Research and Innovation 2017-2020 and 2021-2024 (PI17/1659, PI20/0429 and IMP/00009 to MAM-P).

Author contributions
Guang-Jie Zhu: Conceptualization; resources; data curation; funding acquisition; investigation; writing – original draft; writing – review and editing. **Yuhang Huang:** Data curation; formal analysis; validation; investigation; visualization; methodology; writing – original draft. **Linqing Zhang:** Resources; data curation; formal analysis; investigation; methodology; writing – original draft. **Keji Yan:** Resources; data curation; formal analysis; validation; investigation; visualization; methodology; writing – review and editing. **Cui Qiu:** Validation; investigation. **Yihan He:** Investigation. **Qing Liu:** Funding acquisition; investigation. **Chengwen Zhu:** Funding acquisition; investigation. **Matías Morín:** Data curation; formal analysis; funding acquisition; investigation; writing – original draft. **Miguel Ángel Moreno-Pelayo:** Resources; data curation; formal analysis; funding acquisition; investigation; writing – original draft. **Min-Sheng Zhu:** Supervision; investigation; writing – original draft. **Xin Cao:** Conceptualization; resources; data curation; investigation; writing – original draft. **Han Zhou:** Funding acquisition; investigation. **Xiaoyun Qian:** Resources; funding acquisition; writing – original draft. **Zhigang Xu:** Conceptualization; resources; data curation; formal analysis; supervision; funding acquisition; methodology; writing – review and editing. **Jie Chen:** Conceptualization; resources; data curation; supervision; funding acquisition; investigation; methodology; writing – original draft. **Xia Gao:** Conceptualization; resources; supervision; funding acquisition; writing – original draft; project administration. **Guoqiang Wan:** Conceptualization; formal analysis; supervision; funding acquisition; investigation; visualization; methodology; writing – original draft; project administration; writing – review and editing.

Disclosure and competing interests statement
The authors declare that they have no conflict of interest.

For more information
i https://hereditaryhearingloss.org/
ii https://www.omim.org
ii https://marc.nju.edu.cn/laboratory_views/16/2.html

References

Aijaz S, D'Atri F, Citi S, Balda MS, Matter K (2005) Binding of GEF-H1 to the tight junction-associated adaptor cingulin results in inhibition of Rho signaling and G1/S phase transition. *Dev Cell* 8: 777–786

Belyantseva IA, Perrin BJ, Sonnemann KJ, Zhu M, Stepanyan R, McGee J, Frolenkov GI, Walsh EJ, Friderici KH, Friedman TB *et al* (2009) Gamma-actin is required for cytoskeletal maintenance but not development. *Proc Natl Acad Sci USA* 106: 9703–9708

Bieniussa L, Jain I, Bosch Grau M, Juergens L, Hagen R, Janke C, Rak K (2023) Microtubule and auditory function - an underestimated connection. *Semin Cell Dev Biol* 137: 74–86

Borck G, Ur Rehman A, Lee K, Pogoda HM, Kakar N, von Ameln S, Grillet N, Hildebrand MS, Ahmed ZM, Nurnberg G *et al* (2011) Loss-of-function mutations of ILDR1 cause autosomal-recessive hearing impairment DFNB42. *Am J Hum Genet* 88: 127–137

Cao Y, Li L, Xu M, Feng Z, Sun X, Lu J, Xu Y, Du P, Wang T, Hu R *et al* (2020) The ChinaMAP analytics of deep whole genome sequences in 10,588 individuals. *Cell Res* 30: 717–731

Chadha S, Kamenov K, Cieza A (2021) The world report on hearing, 2021. *Bull World Health Organ* 99: 242–242A

Citi S, D'Atri F, Parry DA (2000) Human and Xenopus cingulin share a modular organization of the coiled-coil rod domain: predictions for intra- and intermolecular assembly. *J Struct Biol* 131: 135–145

Citi S, Paschoud S, Pulimeno P, Timolati F, De Robertis F, Jond L, Guillemot L (2009) The tight junction protein cingulin regulates gene expression and RhoA signaling. *Ann NY Acad Sci* 1165: 88–98

Citi S, Pulimeno P, Paschoud S (2012) Cingulin, paracingulin, and PLEKHA7: signaling and cytoskeletal adaptors at the apical junctional complex. *Ann NY Acad Sci* 1257: 125–132

Cordenonsi M, D'Atri F, Hammar E, Parry DA, Kendrick-Jones J, Shore D, Citi S (1999) Cingulin contains globular and coiled-coil domains and interacts with ZO-1, ZO-2, ZO-3, and myosin. *J Cell Biol* 147: 1569–1582

D'Atri F, Citi S (2001) Cingulin interacts with F-actin *in vitro*. *FEBS Lett* 507: 21–24

D'Atri F, Nadalutti F, Citi S (2002) Evidence for a functional interaction between cingulin and ZO-1 in cultured cells. *J Biol Chem* 277: 27757–27764

Delmaghani S, Defourny J, Aghaie A, Beurg M, Dulon D, Thelen N, Perfettini I, Zelles T, Aller M, Meyer A *et al* (2015) Hypervulnerability to sound exposure through impaired adaptive proliferation of peroxisomes. *Cell* 163: 894–906

DeRosier DJ, Tilney LG (1989) The structure of the cuticular plate, an *in vivo* actin gel. *J Cell Biol* 109: 2853–2867

Drummond MC, Belyantseva IA, Friderici KH, Friedman TB (2012) Actin in hair cells and hearing loss. *Hear Res* 288: 89–99

Du TT, Dewey JB, Wagner EL, Cui R, Heo J, Park JJ, Francis SP, Perez-Reyes E, Guillot SJ, Sherman NE *et al* (2019) LMO7 deficiency reveals the significance of the cuticular plate for hearing function. *Nat Commun* 10: 1117

Du H, Ye C, Wu D, Zang YY, Zhang L, Chen C, He XY, Yang JJ, Hu P, Xu Z *et al* (2020) The cation channel TMEM63B is an osmosensor required for hearing. *Cell Rep* 31: 107596

© 2023 The Authors

Etournay R, Lepelletier L, Boutet de Monvel J, Michel V, Cayet N, Leibovici M, Weil D, Foucher I, Hardelin JP, Petit C (2010) Cochlear outer hair cells undergo an apical circumference remodeling constrained by the hair bundle shape. *Development* 137: 1373 – 1383

Gao X, Chen C, Shi S, Qian F, Liu D, Gong J (2023) Tight junctions in the auditory system: structure, distribution and function. *Curr Protein Pept Sci* 24: 203 – 214

Gilels F, Paquette ST, Beaulac HJ, Bullen A, White PM (2017) Severe hearing loss and outer hair cell death in homozygous Foxo3 knockout mice after moderate noise exposure. *Sci Rep* 7: 1054

Gonzalez-Mariscal L, Dominguez-Calderon A, Raya-Sandino A, Ortega-Olvera JM, Vargas-Sierra O, Martinez-Revollar G (2014) Tight junctions and the regulation of gene expression. *Semin Cell Dev Biol* 36: 213 – 223

Gow A, Davies C, Southwood CM, Frolenkov G, Chrustowski M, Ng L, Yamauchi D, Marcus DC, Kachar B (2004) Deafness in claudin 11-null mice reveals the critical contribution of basal cell tight junctions to stria vascularis function. *J Neurosci* 24: 7051 – 7062

Guillemot L, Citi S (2006) Cingulin regulates claudin-2 expression and cell proliferation through the small GTPase RhoA. *Mol Biol Cell* 17: 3569 – 3577

Hirokawa N, Tilney LG, Fujiwara K, Heuser JE (1982) Organization of actin, myosin, and intermediate filaments in the brush border of intestinal epithelial cells. *J Cell Biol* 94: 425 – 443

Jaeger RG, Fex J, Kachar B (1994) Structural basis for mechanical transduction in the frog vestibular sensory apparatus: II. The role of microtubules in the organization of the cuticular plate. *Hear Res* 77: 207 – 215

Kochhar A, Hildebrand MS, Smith RJ (2007) Clinical aspects of hereditary hearing loss. *Genet Med* 9: 393 – 408

Kohrman D, Wan G, Cassinotti L, Corfas G (2020) Hidden hearing loss: a disorder with multiple etiologies and mechanisms. *Cold Spring Harb Perspect Med* 10: a035493

Lachgar M, Morin M, Villamar M, Del Castillo I, Moreno-Pelayo MA (2021) A novel truncating mutation in HOMER2 causes nonsyndromic progressive DFNA68 hearing loss in a Spanish family. *Genes* 12: 411

Liberman MC (1987) Chronic ultrastructural changes in acoustic trauma: serial-section reconstruction of stereocilia and cuticular plates. *Hear Res* 26: 65 – 88

Liu H, Chen L, Giffen KP, Stringham ST, Li Y, Judge PD, Beisel KW, He DZZ (2018a) Cell-specific transcriptome analysis shows that adult pillar and deiters' cells express genes encoding machinery for specializations of cochlear hair cells. *Front Mol Neurosci* 11: 356

Liu H, Chen L, Giffen KP, Stringham ST, Li Y, Judge PD, Beisel KW, He DZZ (2018b) Gene Expression Omnibus GSE111347 (https://www.ncbi.nlm.nih.gov/geo/query/acc.cgi?acc=GSE111347). [DATASET]

Liu Y, Qi J, Chen X, Tang M, Chu C, Zhu W, Li H, Tian C, Yang G, Zhong C *et al* (2019) Critical role of spectrin in hearing development and deafness. *Sci Adv* 5: eaav7803

Lu Y, Tang D, Zheng Z, Wang X, Zuo N, Yan R, Wu C, Ma J, Wang C, Xu H *et al* (2022) Cingulin b is required for zebrafish lateral line development through regulation of mitogen-activated protein kinase and cellular senescence signaling pathways. *Front Mol Neurosci* 15: 844668

Mangan AJ, Sietsema DV, Li D, Moore JK, Citi S, Prekeris R (2016) Cingulin and actin mediate midbody-dependent apical lumen formation during polarization of epithelial cells. *Nat Commun* 7: 12426

Miralles F, Posern G, Zaromytidou AI, Treisman R (2003) Actin dynamics control SRF activity by regulation of its coactivator MAL. *Cell* 113: 329 – 342

Modamio-Hoybjor S, Mencia A, Goodyear R, del Castillo I, Richardson G, Moreno F, Moreno-Pelayo MA (2007) A mutation in CCDC50, a gene encoding an effector of epidermal growth factor-mediated cell signaling, causes progressive hearing loss. *Am J Hum Genet* 80: 1076 – 1089

Morin M, Borreguero L, Booth KT, Lachgar M, Huygen P, Villamar M, Mayo F, Barrio LC, Santos Serrao de Castro L, Morales C *et al* (2020) Insights into the pathophysiology of DFNA10 hearing loss associated with novel EYA4 variants. *Sci Rep* 10: 6213

Morozko EL, Nishio A, Ingham NJ, Chandra R, Fitzgerald T, Martelletti E, Borck G, Wilson E, Riordan GP, Wangemann P *et al* (2015) ILDR1 null mice, a model of human deafness DFNB42, show structural aberrations of tricellular tight junctions and degeneration of auditory hair cells. *Hum Mol Genet* 24: 609 – 624

Nayak G, Lee SI, Yousaf R, Edelmann SE, Trincot C, Van Itallie CM, Sinha GP, Rafeeq M, Jones SM, Belyantseva IA *et al* (2013) Tricellulin deficiency affects tight junction architecture and cochlear hair cells. *J Clin Invest* 123: 4036 – 4049

Perrin BJ, Sonnemann KJ, Ervasti JM (2010) Beta-actin and gamma-actin are each dispensable for auditory hair cell development but required for Stereocilia maintenance. *PLoS Genet* 6: e1001158

Petit C, Bonnet C, Safieddine S (2023) Deafness: from genetic architecture to gene therapy. *Nat Rev Genet*

Pollock LM, McDermott BM Jr (2015) The cuticular plate: a riddle, wrapped in a mystery, inside a hair cell. *Birth Defects Res C Embryo Today* 105: 126 – 139

Raphael Y, Athey BD, Wang Y, Lee MK, Altschuler RA (1994) F-actin, tubulin and spectrin in the organ of Corti: comparative distribution in different cell types and mammalian species. *Hear Res* 76: 173 – 187

Riazuddin S, Ahmed ZM, Fanning AS, Lagziel A, Kitajiri S, Ramzan K, Khan SN, Chattaraj P, Friedman PL, Anderson JM *et al* (2006) Tricellulin is a tight-junction protein necessary for hearing. *Am J Hum Genet* 79: 1040 – 1051

Rossetti S, Harris PC (2007) Genotype-phenotype correlations in autosomal dominant and autosomal recessive polycystic kidney disease. *J Am Soc Nephrol* 18: 1374 – 1380

Rouaud F, Huang W, Flinois A, Jain K, Vasileva E, Di Mattia T, Mauperin M, Parry DAD, Dugina V, Chaponnier C *et al* (2023) Cingulin and paracingulin tether myosins-2 to junctions to mechanoregulate the plasma membrane. *J Cell Biol* 222: e202208065

Sacco KA, Milner JD (2019) Gene-environment interactions in primary atopic disorders. *Curr Opin Immunol* 60: 148 – 155

Sawada N, Murata M, Kikuchi K, Osanai M, Tobioka H, Kojima T, Chiba H (2003) Tight junctions and human diseases. *Med Electron Microsc* 36: 147 – 156

Sineni CJ, Yildirim-Baylan M, Guo S, Camarena V, Wang G, Tokgoz-Yilmaz S, Duman D, Bademci G, Tekin M (2019) A truncating CLDN9 variant is associated with autosomal recessive nonsyndromic hearing loss. *Hum Genet* 138: 1071 – 1075

Singer FR (2015) Paget's disease of bone-genetic and environmental factors. *Nat Rev Endocrinol* 11: 662 – 671

Steyger PS, Furness DN, Hackney CM, Richardson GP (1989) Tubulin and microtubules in cochlear hair cells: comparative immunocytochemistry and ultrastructure. *Hear Res* 42: 1 – 16

Vahava O, Morell R, Lynch ED, Weiss S, Kagan ME, Ahituv N, Morrow JE, Lee MK, Skvorak AB, Morton CC *et al* (1998) Mutation in transcription factor POU4F3 associated with inherited progressive hearing loss in humans. *Science* 279: 1950 – 1954

Walsh T, Pierce SB, Lenz DR, Brownstein Z, Dagan-Rosenfeld O, Shahin H, Roeb W, McCarthy S, Nord AS, Gordon CR *et al* (2010) Genomic duplication and overexpression of TJP2/ZO-2 leads to altered expression of apoptosis genes in progressive nonsyndromic hearing loss DFNA51. *Am J Hum Genet* 87: 101 – 109

© 2023 The Authors

Wan G, Corfas G (2017) Transient auditory nerve demyelination as a new mechanism for hidden hearing loss. *Nat Commun* 8: 14487

Wan G, Gomez-Casati ME, Gigliello AR, Liberman MC, Corfas G (2014) Neurotrophin-3 regulates ribbon synapse density in the cochlea and induces synapse regeneration after acoustic trauma. *Elife* 3: e03564

Wang J, Puel JL (2018) Toward cochlear therapies. *Physiol Rev* 98: 2477–2522

Wilcox ER, Burton QL, Naz S, Riazuddin S, Smith TN, Ploplis B, Belyantseva I, Ben-Yosef T, Liburd NA, Morell RJ *et al* (2001) Mutations in the gene encoding tight junction claudin-14 cause autosomal recessive deafness DFNB29. *Cell* 104: 165–172

Wrobel C, Zafeiriou MP, Moser T (2021) Understanding and treating paediatric hearing impairment. *EBioMedicine* 63: 103171

Yan D, Zhu Y, Walsh T, Xie D, Yuan H, Sirmaci A, Fujikawa T, Wong AC, Loh TL, Du L *et al* (2013) Mutation of the ATP-gated $P2X_2$ receptor leads to progressive hearing loss and increased susceptibility to noise. *Proc Natl Acad Sci USA* 110: 2228–2233

Yano T, Torisawa T, Oiwa K, Tsukita S (2018) AMPK-dependent phosphorylation of cingulin reversibly regulates its binding to actin filaments and microtubules. *Sci Rep* 8: 15550

Zhu GJ, Gong S, Ma DB, Tao T, He WQ, Zhang L, Wang F, Qian XY, Zhou H, Fan C *et al* (2020) Aldh inhibitor restores auditory function in a mouse model of human deafness. *PLoS Genet* 16: e1009040

License: This is an open access article under the terms of the Creative Commons Attribution License, which permits use, distribution and reproduction in any medium, provided the original work is properly cited.

© 2023 The Authors

Expanded View Figures

Figure EV1. Audiograms of the individuals within the family.

A Audiograms of the individuals affected by hearing loss.
B Audiograms of the individuals without hearing loss. Individual II:2 developed unilateral hearing loss at age 30 due to chronic suppurative otitis media of the right ear.

Source data are available online for this figure.

© 2023 The Authors

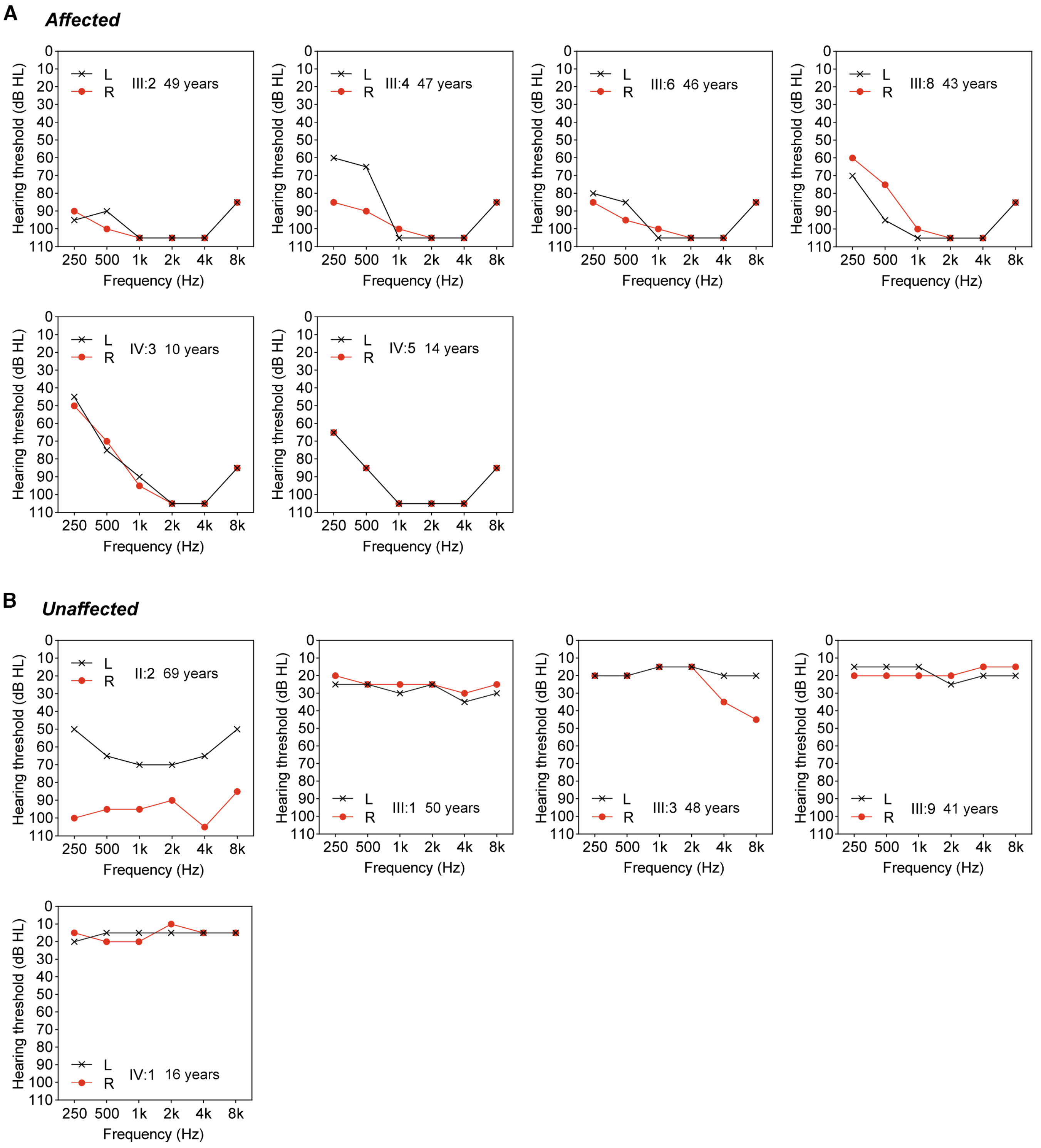

Figure EV1.

© 2023 The Authors

Figure EV2. Abnormal expression pattern of N-terminal EGFP-tagged mutant human CGN.

A A schematic diagram of N-terminal EGFP-tagged WT and mutant (p.L1110Lfs*17) CGN constructs used in this study.
B MDCK cells expressing EGFP-tagged WT or mutant CGN were immunolabeled with CGN or EGFP antibodies.
C Western blot analysis of whole cell lysates from MDCK cells transfected with EGFP-tagged WT or mutant CGN. Exogenous EGFP-CGN fusion protein was immunoblotted with EGFP antibody.
D RT-qPCR of *CGN* expression in transfected MDCK cells ($n = 3$ biological replicates).
E High-magnification immunofluorescent images showing subcellular localizations of EGFP-tagged WT and mutant CGN (white arrows).
F HEK293T cells expressing EGFP-tagged WT or mutant CGN were immunolabeled with EGFP antibodies. Arrow heads indicate subcellular localization of the CGN proteins.
G Western blot analysis of whole cell lysates from HEK293T cells transfected with EGFP-tagged WT or mutant CGN. Exogenous EGFP-CGN fusion protein was immunoblotted with EGFP antibody.

Data information: Data are presented as mean ± SEM; unpaired Student's *t*-test was used in (D). ns, not significant, $P > 0.05$.
Source data are available online for this figure.

© 2023 The Authors

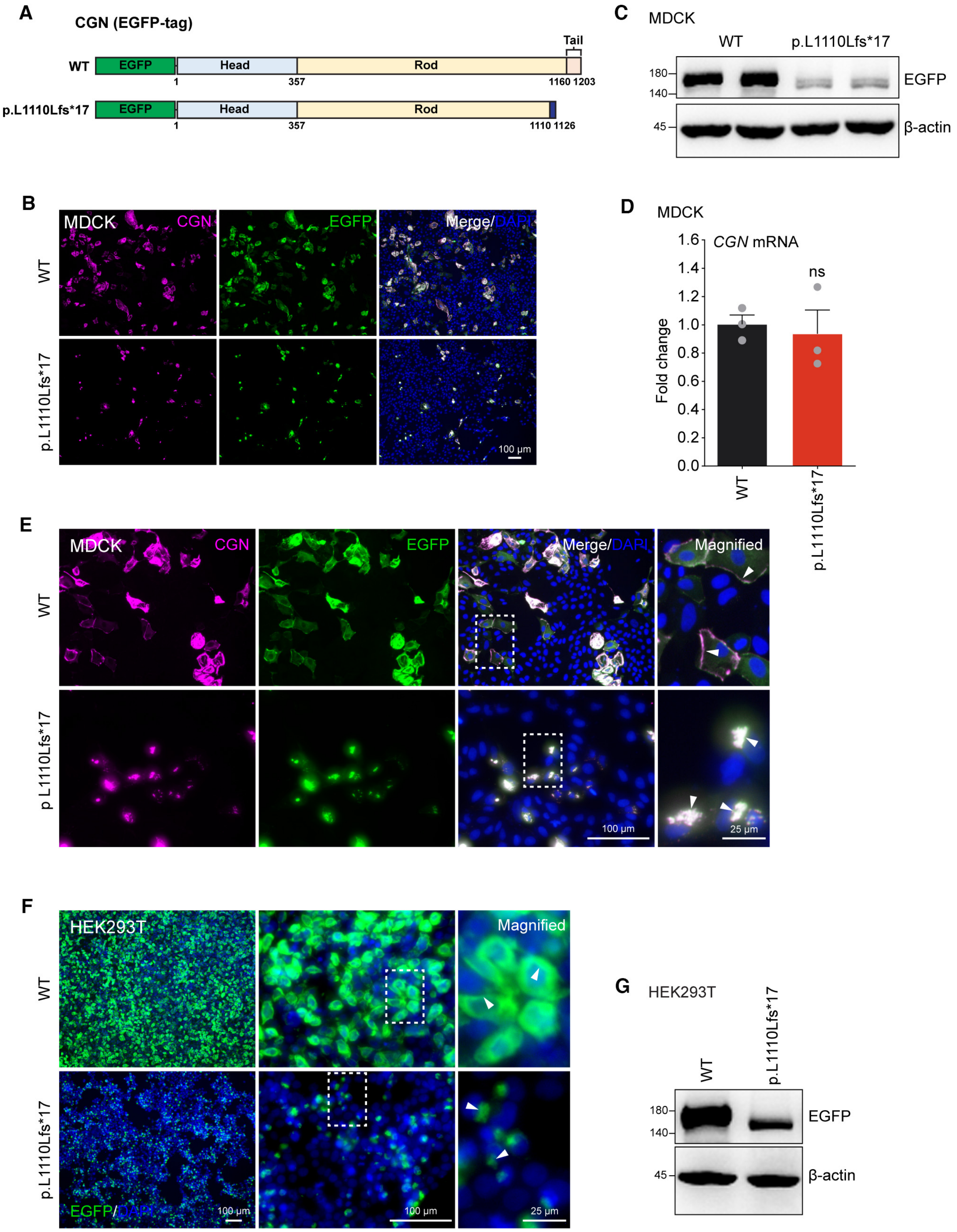

Figure EV2.

 © 2023 The Authors

Figure EV3. Generation and validation of the *Cgn-cKO and Cgn^{delG} knockin mice.*

A Strategy to generate hair cell-specific *Cgn-cKO* mice by crossing *Cgn$^{fl/fl}$* with *Pou4f3$^{EGFP\text{-}creER}$* (*Pou4f3^{creER}*) mice.
B Genotyping of the LoxP knockin allele in *Cgn$^{fl/fl}$* and the creER knockin allele in *Pou4f3^{creER}* mice. A 398-bp size band can be detected in the genomic DNA from *Cgn$^{fl/fl}$* mice. A 504-bp size band can be detected in the genomic DNA from *Pou4f3^{creER}* mice.
C Cochlear whole mount immunofluorescence to validate efficiency of Cre-mediated recombination and *Cgn* knockout by labeling Pou4f3-driven EGFP (hair cells, green) and CGN (magenta) of 2-month-old *Cgn-cKO* mice. Examples of un-recombined hair cells (asterisks) and the neighboring recombined hair cells (arrowheads) were shown.
D Efficiency of Cre-mediated recombination and CGN knockout in IHCs and OHCs from 2-month-old *Cgn-cKO* cochlea (n = 4–22 biological replicates from 2 to 5 cochleae).
E Strategy to generate *Cgn^{delG}* mice by CRISPR-Cas9 technology.
F Sanger sequencing of the genomic DNA from *Cgn$^{+/+}$* and *Cgn$^{delG/delG}$* mice. Arrow indicates the G-base deleted from the *Cgn$^{delG/delG}$* mouse genome.
G Genotyping of *Cgn^{delG}* mice using wild-type and knockin-specific primers.

Data information: Data are presented as mean ± SEM; unpaired Student's t-test was used in (D). ****$P < 0.0001$.
Source data are available online for this figure.

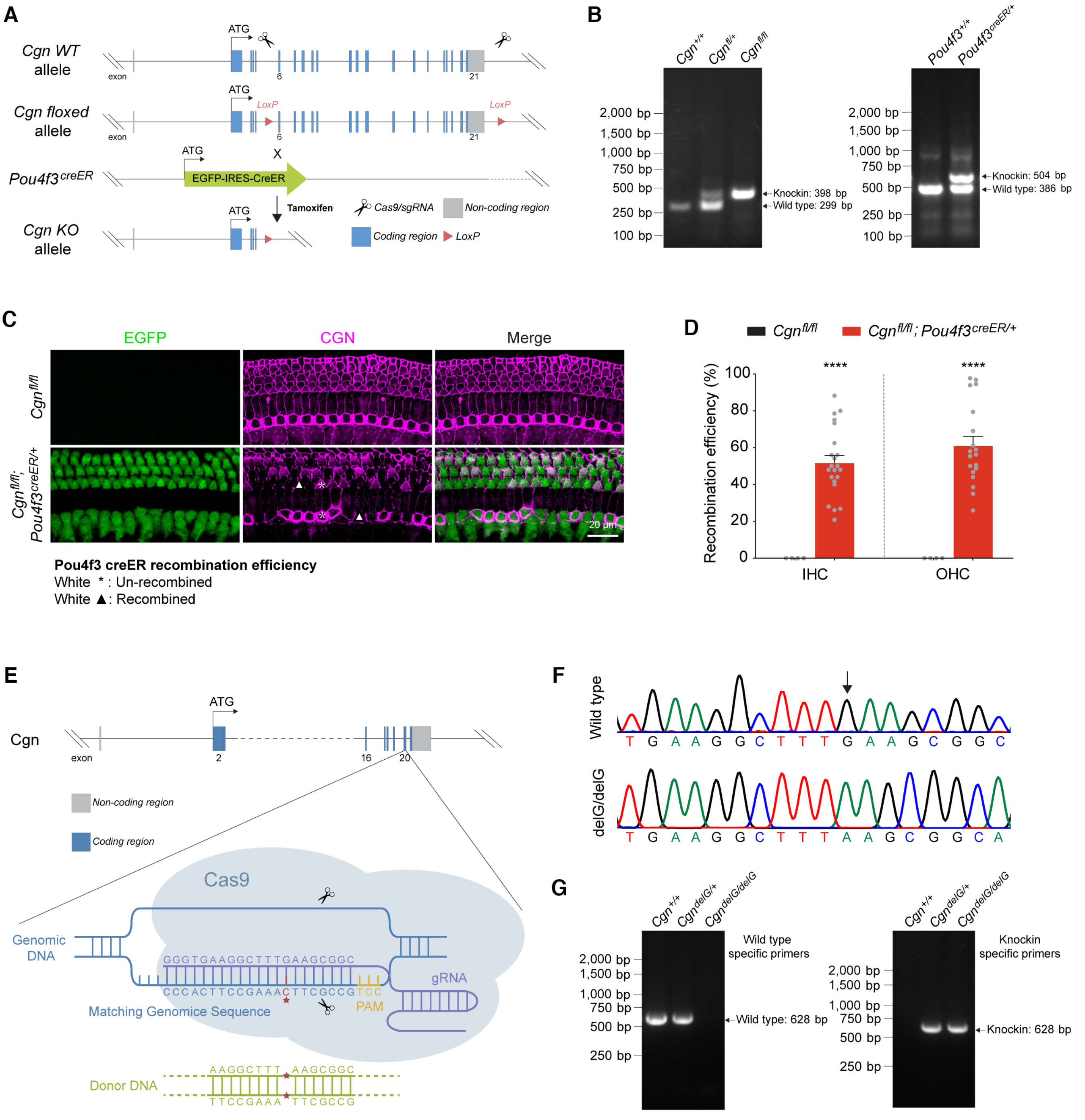

Figure EV3.

 © 2023 The Authors

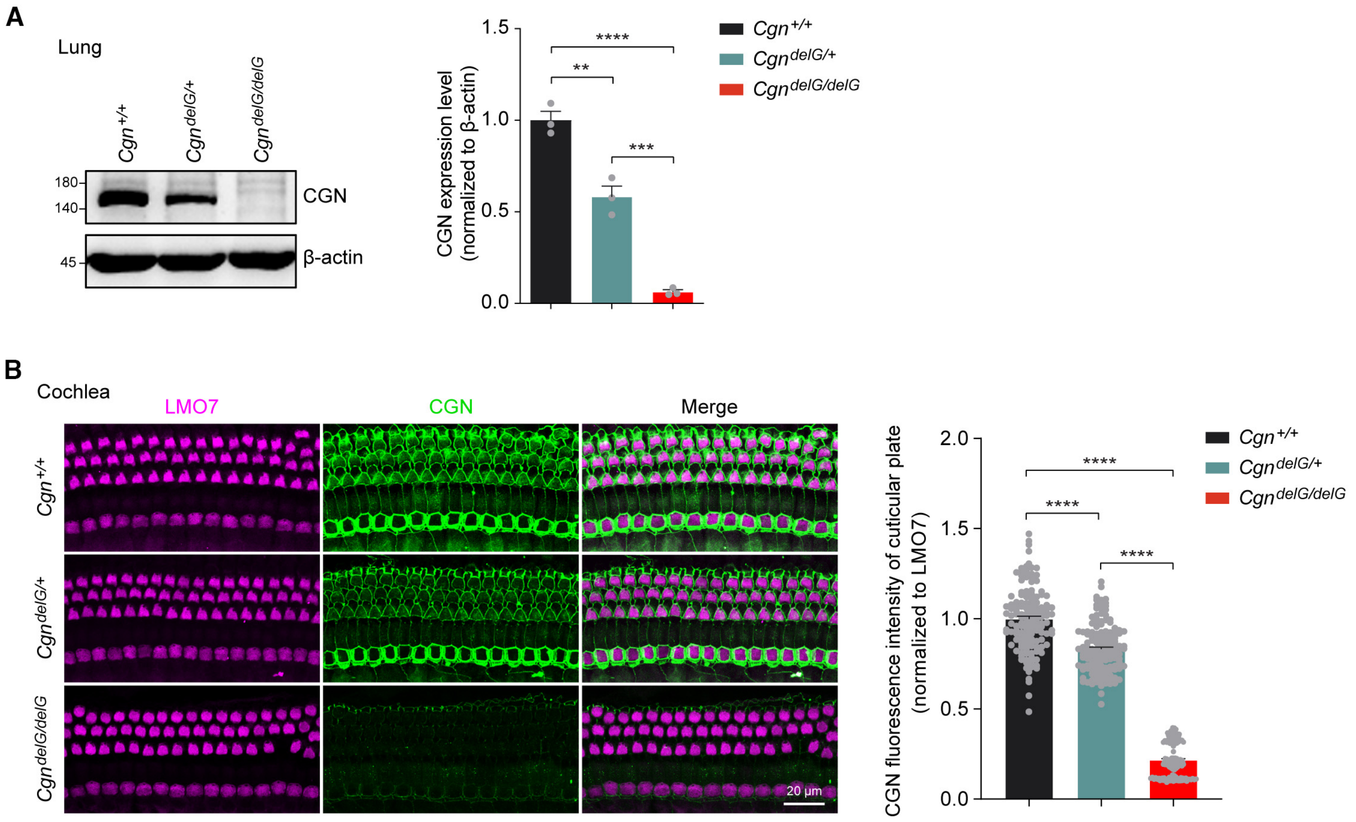

Figure EV4. Abnormal CGN expression in lung and cochlear sensory epithelia of *Cgn*^{delG} mice.

A Western blot and quantification of CGN protein expression in P21 *Cgn*^{delG} lung lysates (n = 3 biological replicates).

B Whole mount immunofluorescence of CGN expression in P21 *Cgn*^{delG} mouse cochlea coimmunostained with LMO7. CGN expression levels at the cuticular plates were normalized to LMO7 immunofluorescent signals (n = 143–148 hair cells from three cochleae).

Data information: Data are presented as mean ± SEM; one-way ANOVA was used in (A and B). **P < 0.01, ***P < 0.001, and ****P < 0.0001.
Source data are available online for this figure.

Figure EV5. Expression and function of CGN in mouse utricles.

A, B Whole mount immunofluorescence of CGN expression in P3, P14, and P21 mouse utricle coimmunostained with Parvalbumin (A) or LMO7 (B).

C Whole mount immunofluorescence of CGN expression in utricles from P21 *Cgn*^{delG} mouse.

D The time to fall from the rotarod of the 2-month-old wildtype and *Cgn*^{delG} mice. No significant difference was observed with all three testing protocols (n = 3–10 mice).

Data information: Data are presented as mean ± SEM; one-way ANOVA was used in (D). not significant, P > 0.05.
Source data are available online for this figure.

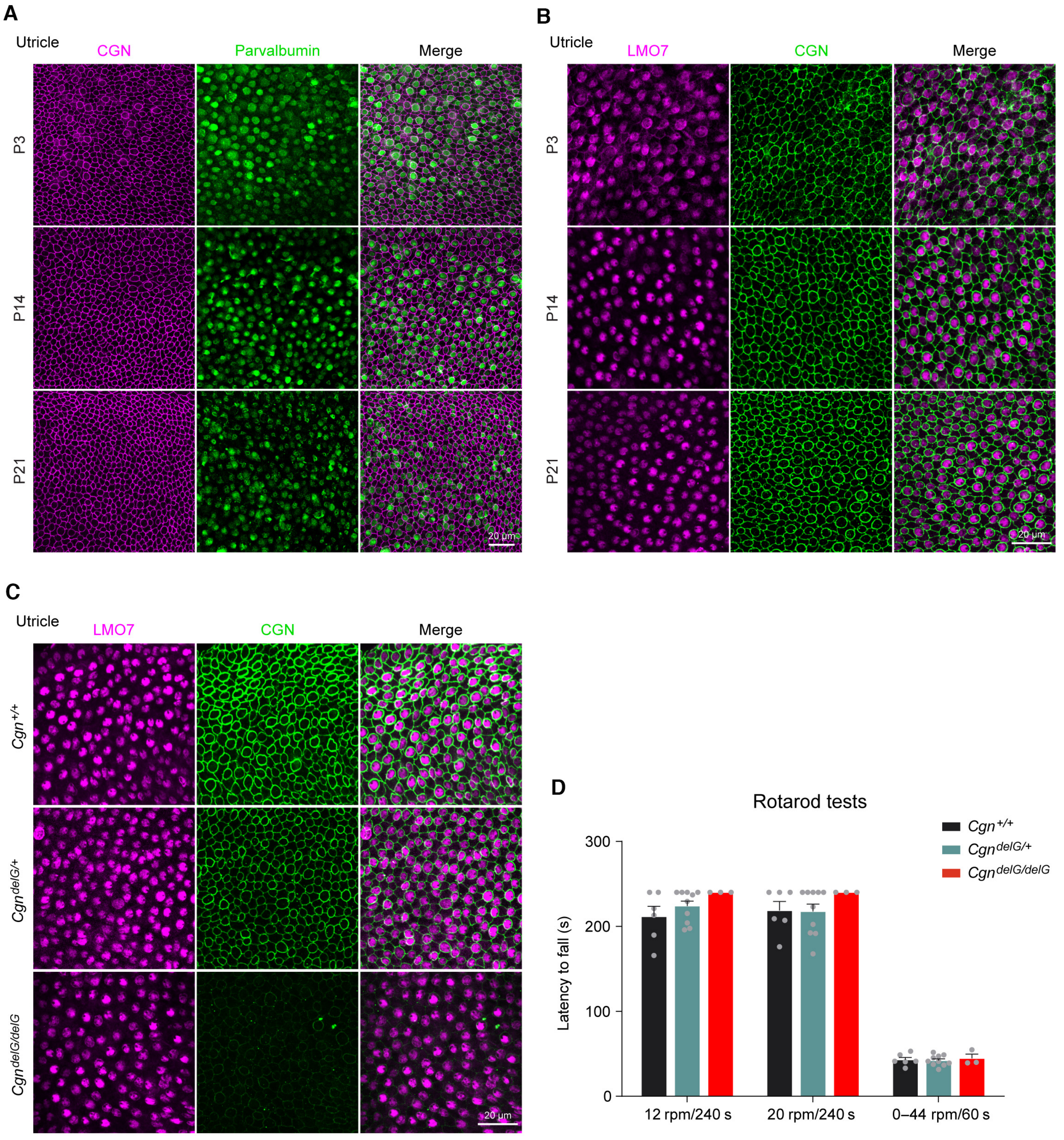

Figure EV5.

 © 2023 The Authors

Appendix

Cingulin regulates hair cell cuticular plate morphology and is required for hearing in human and mouse

Table of Contents……………………………………………………………………………….….1

Appendix Fig S1. Multipoint linkage analysis and the candidate genetic variants………………………………………………………………………………….....2

Appendix Fig S2. Validation of CGN antibodies…………………………………………….…...3

Appendix Fig S3. Abnormal expression pattern of the mutant human CGN in various cell lines..………………………………………………………………………………………5

Appendix Fig S4. Normal cochlear synaptic ribbon densities in the *Cgn*^{delG} mice…………...7

Appendix Fig S5. No effects of abnormal CGN expression on tight junction related markers.8

Appendix Fig S6. Normal microtubule structures in the *Cgn* mutant mice..........................10

Appendix Fig S7. Abnormal hair cell cuticular plate morphology in the *Cgn*^{delG} mice at middle frequency...11

Appendix Table S1. Classification of the different genetic variants identified in *CGN* in Spanish families...12

Appendix Table S2. Primers for RT-qPCR analyses..14

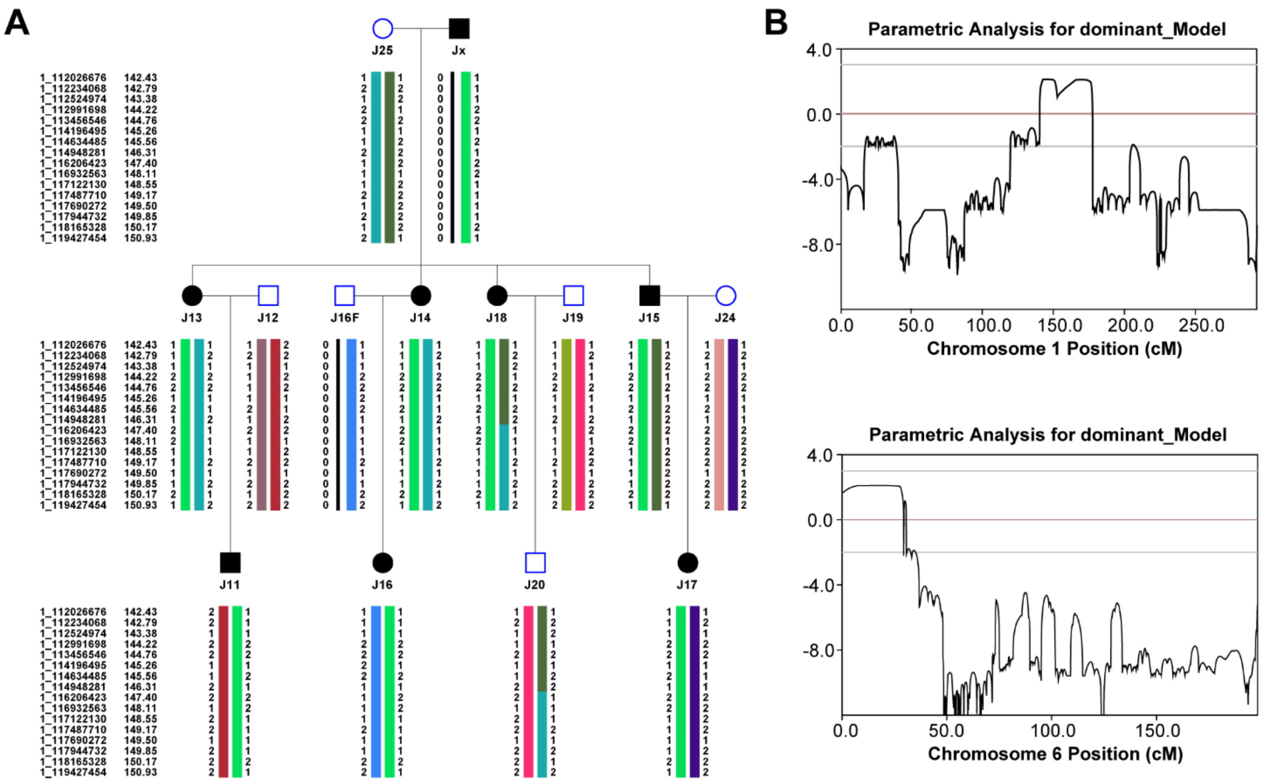

C

CHROM	POS	REF	ALT	Func.refGene	Gene.refGene	ExonicFunc.refGene	cytoBand	avsnp147
chr1	145538716	TCA	T	splicing	*ITGA10*	.	1q21.1	rs587614056
chr1	151509228	TG	T	exonic	*CGN*	frameshift_deletion	1q21.3	.
chr1	154407658	AG	A	intronic	*IL6R*	.	1q21.3	rs761937636
chr1	17084536	TGGAACA	T	exonic	*MST1L*	nonframeshift_deletion	1p36.13	rs142741624
chr6	2971195	A	AG	UTR5	*SERPINB6*	.	6p25.2	rs149382735
chr6	7295300	TA	T	intronic	*SSR1*	.	6p24.3	rs35998405
chr6	3232460	TGGGC	T	intergenic	*TUBB2B;PSMG4*	.	6p25.2	rs10597566

Appendix Fig S1. Multipoint linkage analysis and the candidate genetic variants.

(A) Pedigree and haplotype diagram for linkage region on chromosome 1 of the ADNSHL family. (B) Multipoint linkage analysis results for chromosomes 1 and 6. (C) Candidate genetic variants co-segregated with the ADNSHL phenotype.

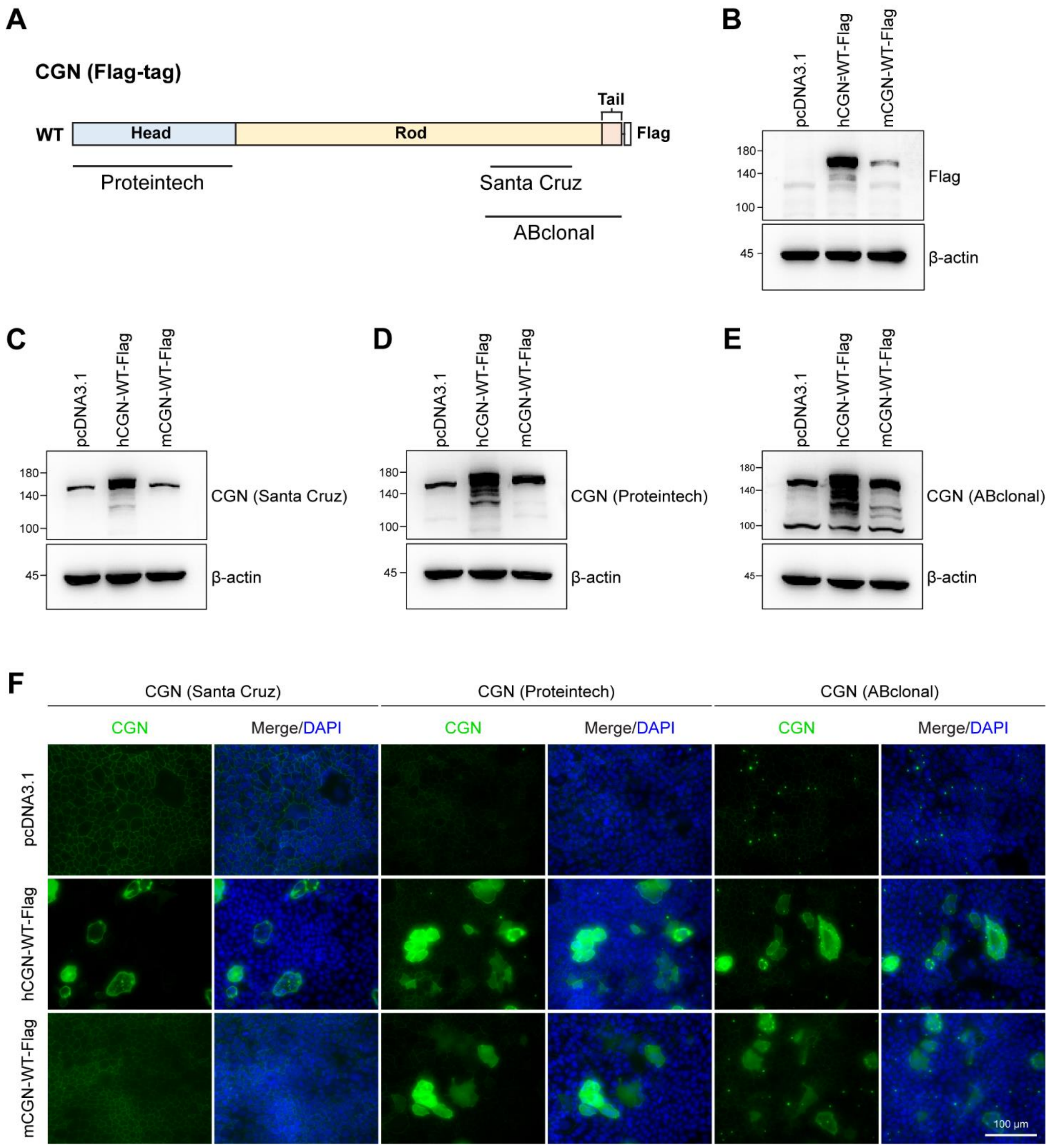

Appendix Fig S2. Validation of CGN antibodies.

(A) A schematic diagram of C-terminal Flag-tagged human or mouse CGN WT constructs (human: hCGN-WT-Flag; mouse: mCGN-WT-Flag) used in this study and antigen recognition sequence of three CGN antibodies. (B) Western blot analysis of whole cell lysates from MDCK cells transfected with WT CGN. Exogenous CGN was immunoblotted with Flag antibody. Both human and mouse exogenous WT CGN were expressed. (C-E) Western blot analysis of whole cell lysates from MDCK cells transfected with WT CGN. CGN was immunoblotted with three different CGN antibodies. (F) MDCK cells expressing WT CGN were immunolabeled with three different CGN antibodies. High magnification immunofluorescent images showing subcellular localizations of WT CGN. According above

results, three different CGN antibodies can recognize the human WT CGN protein sequence, while the monoclonal antibody from Santa Cruz has a lower efficiency for mouse WT CGN protein. Therefore, rabbit anti-Cingulin (Proteintech) and mouse anti-Cingulin (Santa Cruz) were selected for probing CGN protein in cell line samples (Western blot and immunofluorescent). Rabbit anti-Cingulin (Proteintech) has fewer nonspecific bands and was used for Western blot of mouse tissue samples. For mouse cochlear whole mount samples, rabbit anti-Cingulin (ABclonal) showed less background and was used to for immunofluorescence of whole mount samples.

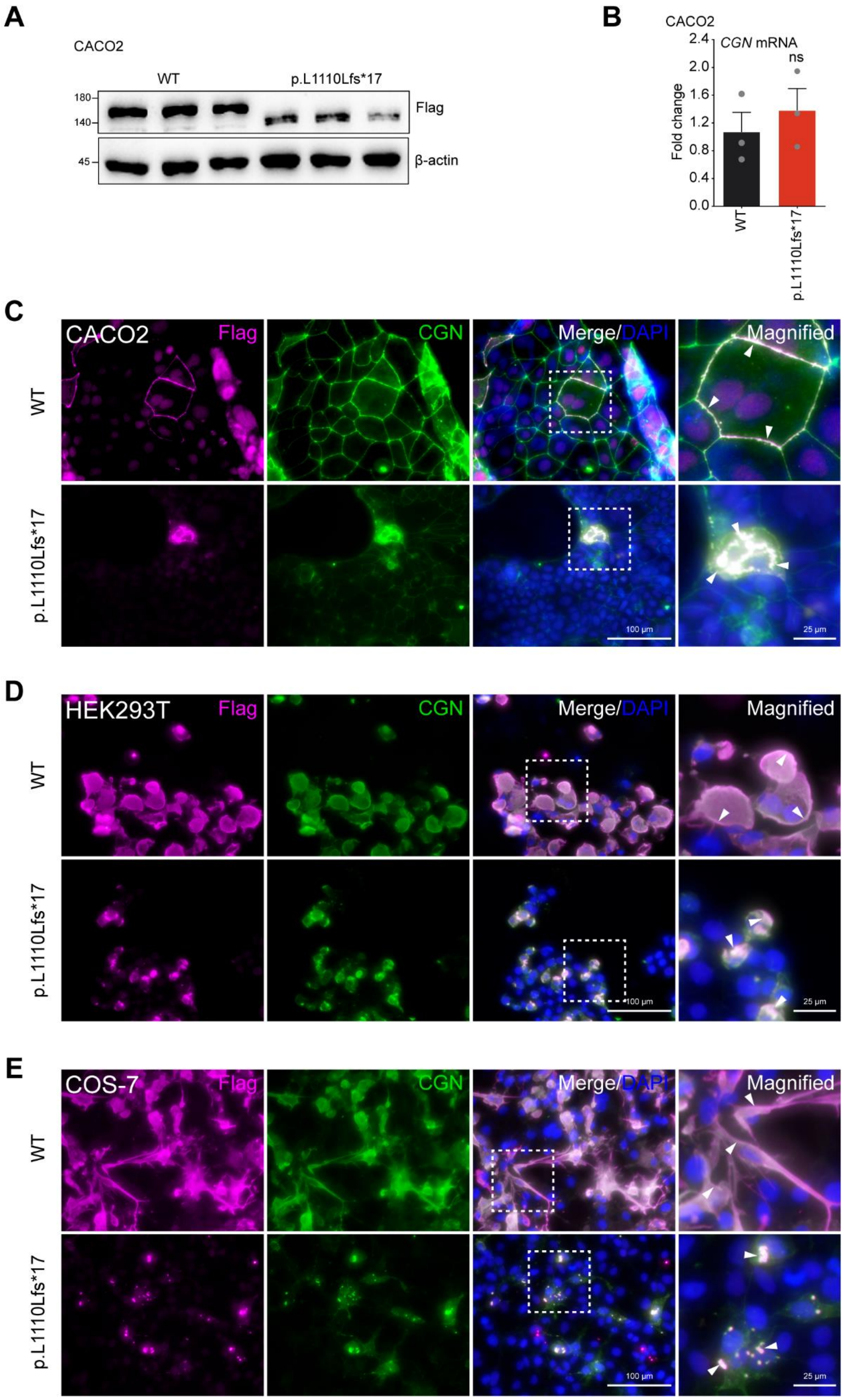
A
CACO2
WT
p.L1110Lfs*17
180
140
Flag
45
β-actin
B
CACO2
CGN mRNA
ns
Fold change
2.4
2.0
1.6
1.2
0.8
0.4
0.0
WT
p.L1110Lfs*17
C
CACO2
Flag
CGN
Merge/DAPI
Magnified
WT
p.L1110Lfs*17
100 µm
25 µm
D
HEK293T
Flag
CGN
Merge/DAPI
Magnified
WT
p.L1110Lfs*17
100 µm
25 µm
E
COS-7
Flag
CGN
Merge/DAPI
Magnified
WT
p.L1110Lfs*17
100 µm
25 µm

Appendix Fig S3. Abnormal expression pattern of the mutant human CGN in various cell lines.

(A) Western blot analysis of whole cell lysates from CACO2 cells transfected with WT or mutant CGN. Exogenous CGN was immunoblotted with Flag antibody. (B) RT-qPCR of *CGN* expression in transfected CACO2 cells. N = 3. Error bars represent ± SEM. ns, P > 0.05 by unpaired student's t-test. (C-E) Immunofluorescence of CGN expression in CACO2 (C), HEK293T (D) or COS-7 (E) cells transfected with WT or mutant CGN. Localizations of WT or mutant CGN (white arrows) were visualized with CGN or Flag antibodies.

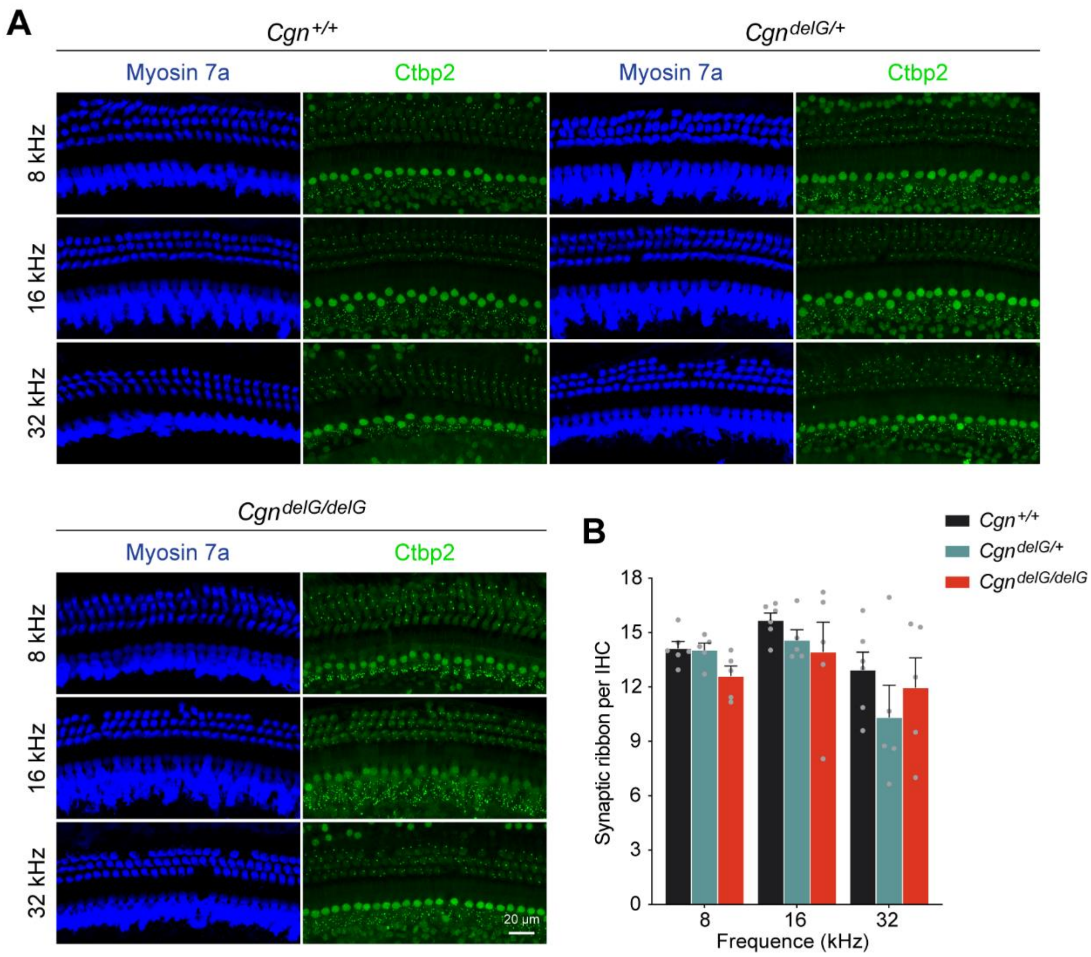

Appendix Fig S4. Normal cochlear synaptic ribbon densities in the *Cgn*^{delG} mice.

(A) Whole mount immunofluorescence of cochlear synaptic ribbons by labeling Ctbp2 (presynaptic ribbons marker, green) and Myosin 7a (blue) of the 2-months old *Cgn*^{delG} mice. (B) Densities of synaptic ribbons in 2-months old *Cgn*^{delG} mice. N = 5-6. Error bars represent ± SEM. P > 0.05 by two-way ANOVA.

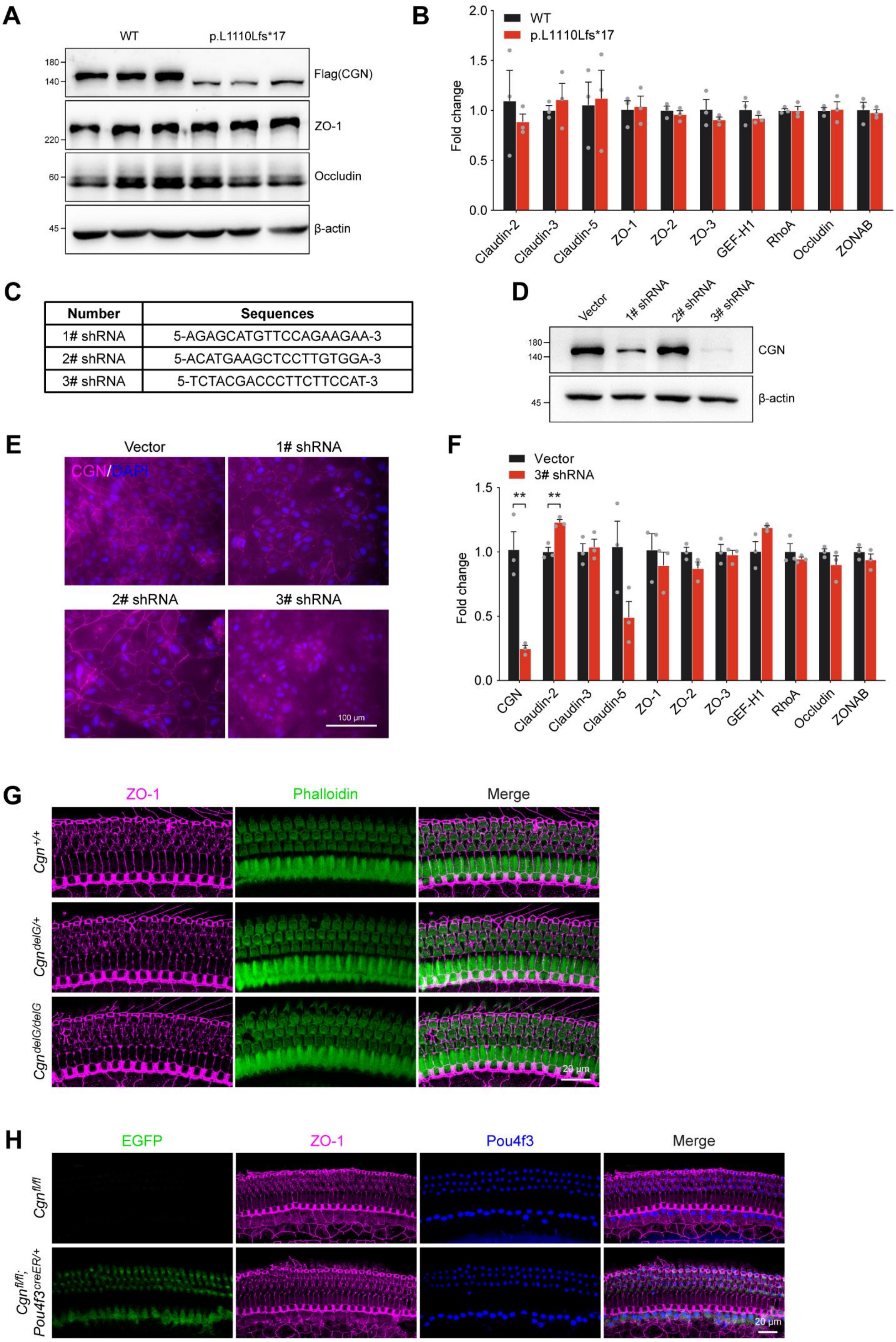

Number	Sequences
1# shRNA	5-AGAGCATGTTCCAGAAGAA-3
2# shRNA	5-ACATGAAGCTCCTTGTGGA-3
3# shRNA	5-TCTACGACCCTTCTTCCAT-3

Appendix Fig S5. No effects of abnormal CGN expression on tight junction related markers.

(A) Western blot analyses of the endogenous ZO-1 and Occludin protein expression in MDCK cells transfected with WT or mutant CGN. (B) RT-qPCR of tight junction related markers in MDCK cells transfected with WT or mutant CGN. Error bars represent ± SEM. P > 0.05 by unpaired student's t-test. (C) shRNA sequences for knocking down *Cgn* in MDCK cells. (D, E) Western blot (D) and immunofluorescence (E) validation of Cgn knockdown from *Cgn-KD* MDCK cells. (F) RT-qPCR of tight junction related markers in *Cgn-KD* MDCK cells by *Cgn* shRNA (3#). N = 3. Error bars represent ± SEM. ** P < 0.01 by unpaired student's *t*-test. (G) Cochlear whole mount immunofluorescence of tight junctions labeled with ZO-1 (magenta) from 2-months old Cgn^{delG} mice. (H) Cochlear whole mount immunofluorescence of the tight junctions labeled with ZO-1 (magenta) from 2-months old *Cgn-cKO* mice. Hair cells were visualized with Pou4f3 (blue) or Pou4f3-driven EGFP (green).

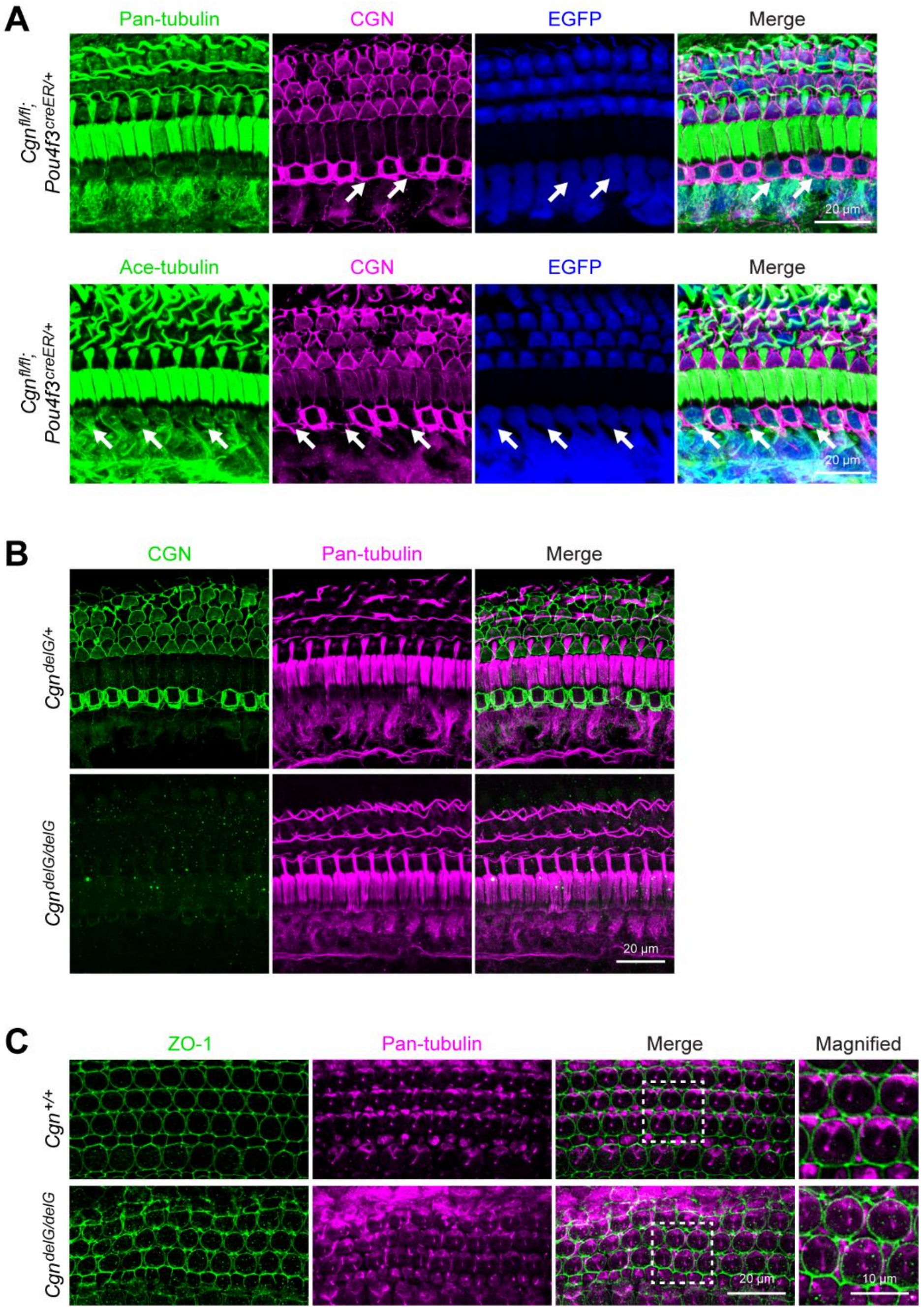

Appendix Fig S6. Normal microtubule structures in the *Cgn* mutant mice.

(A) Cochlear whole mount immunofluorescence of microtubules by labeling pan-tubulin or acetylated-tubulin (green) from 2-months old *Cgn-cKO* mice. White arrows label the recombined IHCs. (B) Cochlear whole mount immunofluorescence of microtubules by labeling pan-tubulin (magenta) from 7-months old *Cgn*^{delG} mice. (C) Cochlear whole mount immunofluorescence of kinocilium by labeling pan-tubulin (magenta) from P0 *Cgn*^{delG} mice.

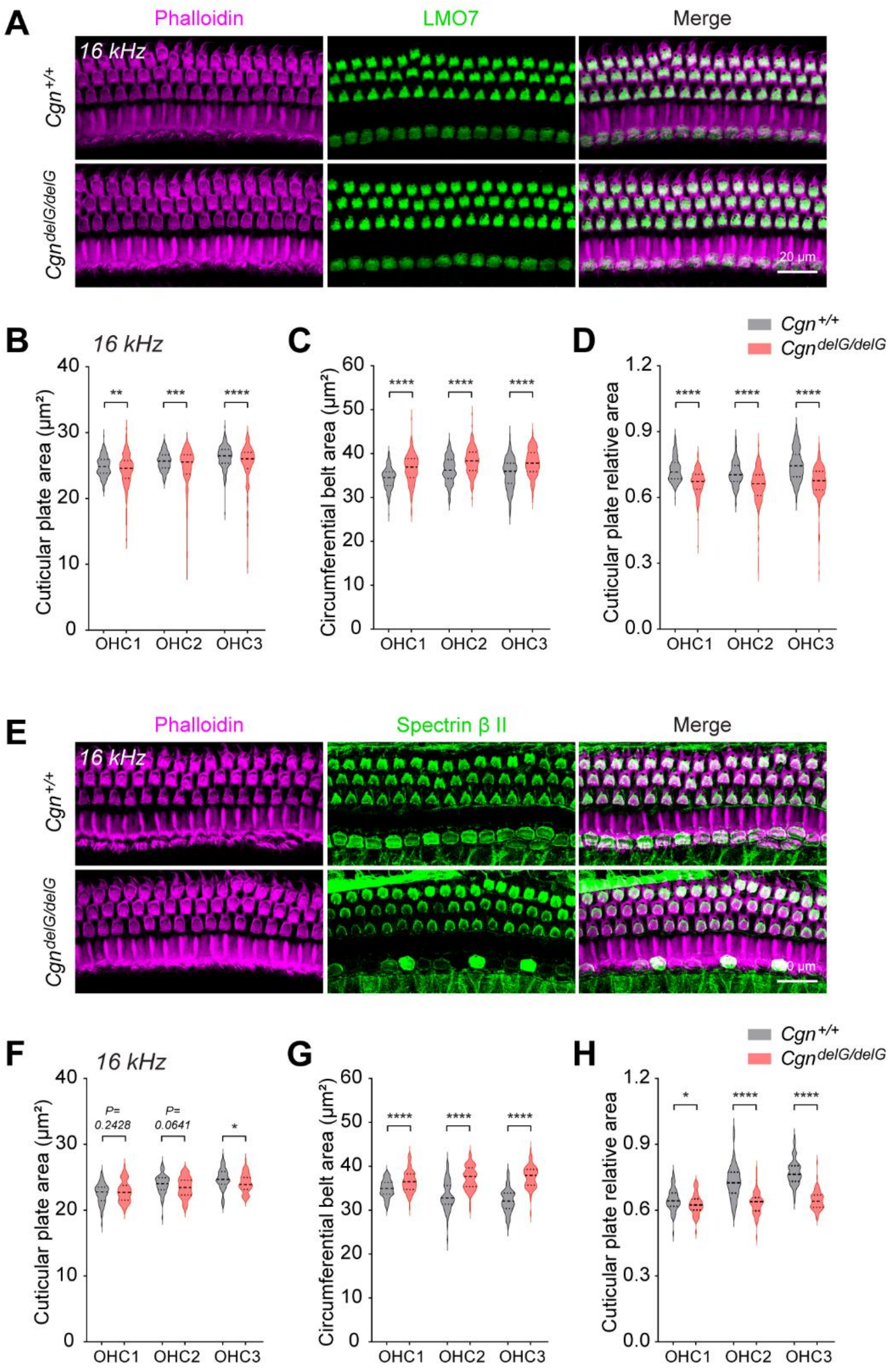

Appendix Fig S7. Abnormal hair cell cuticular plate morphology in the *Cgn^{delG}* mice at middle frequency.

(A, E) Whole mount immunofluorescence of hair cell cuticular plates by labeling (A) LMO7 or (E) Spectrin β II from 2-months old *Cgn^{delG}* mice at 16 kHz cochlear region. (B, F) Areas of the cuticular plates, (C, G) areas of the circumferential belts and (D, H) relative areas of the cuticular plates quantified from immunofluorescent images obtained by (B-D) LMO7 (N = 186-195 hair cells) or (F-H) Spectrin β II (N = 48-64 hair cells). * $P < 0.05$, ** $P < 0.01$, *** $P < 0.001$ and **** $P < 0.0001$ by unpaired student's *t*-test.

Appendix Table S1. Classification of the different genetic variants identified in *CGN* in Spanish families.

DNA change / Protein change	Coding impact	Transcript variant	Exon	Origin	ACMG classification	CSVS Allele Freq	GnomAD Allele Freq	Number of occurrences
c.31C>T p.Arg11Trp	Missense	NM_020770.3	2/21	Spanish	Benign (BS1, BS2, BP4, BP6, BP1)	0.0065	0.00275	1/96
c.586C>T p.Arg196Trp	Missense	NM_020770.3	2/21	Spanish	Likely Benign (PM2, BP4,BP1)	0.001	0.000331	1/96
c.1640G>A p.Arg547Lys	Missense	NM_020770.3	9/21	Spanish	Benign (BS1, BS2, BP4, BP6, BP1)	0.01	0.00654	2/96
c.2311G>A p.Glu771Lys	Missense	NM_020770.3	12/21	Spanish	Benign (BS1, BS2, BP4, BP6, BP1)	0.017	0.007	2/96
c.2420G>A p.Arg807Gln	Missense	NM_020770.3	13/21	Spanish	Benign (BS1, BS2, BP1, BP4)	0.341	0.00389	2/96
c.2500C>T Arg834Trp	Missense	NM_020770.3	13/21	Spanish	Likely Benign (PM2, BP4,BP1)	0.00050	0.000139	1/96
c.2650C>T p.Arg884Trp	Missense	NM_020770.3	14/21	Spanish	Likely Benign (PM2, BP4,BP1)	0.001	0.000162	1/96
c.3113A>G p.Glu1038Gly	Missense	NM_020770.3	18/21	Spanish	Likely Benign (PM2, BP4,BP1	0	0.000573	1/96

All the variants are named according to NM_020770.3 transcript. The nomenclature was checked using Mutalyzer 2.0.34 (Wildeman, van Ophuizen et al., 2008). ACMG criteria (Kopanos, Tsiolkas et al., 2019, Richards, Aziz et al., 2015): **PM2** (Pathogenic, Moderate): absent from controls (or at extremely low frequency if recessive) in Exome Sequencing Project,

1000 Genomes Project, or Exome Aggregation Consortium. **BS1** (Benign, Strong): allele frequency is greater than expected for disorder. **BS2** (Benign, Strong): observed in a healthy adult individual for a recessive (homozygous), dominant (heterozygous), or X-linked (hemizygous) disorder, with full penetrance expected at an early age. **BP1** (Benign, Supporting): missense variant in a gene for which primarily truncating variants are known to cause disease. **BP4** (Benign, Supporting): multiple lines of computational evidence suggest no impact on gene or gene product (conservation, evolutionary, splicing impact, etc.). **BP6** (Benign, Supporting): Reputable source recently reports variant as benign, but the evidence is not available to the laboratory to perform an independent evaluation. The databases GnomAD (Karczewski, Francioli et al., 2020) and CSVS (Peña-Chilet, Roldán et al., 2020) were searched on the 17th of February 2023. N.A: not available.

References

Karczewski KJ, Francioli LC, Tiao G, Cummings BB, Alföldi J, Wang Q, Collins RL, Laricchia KM, Ganna A, Birnbaum DP, Gauthier LD, Brand H, Solomonson M, Watts NA, Rhodes D, Singer-Berk M, England EM, Seaby EG, Kosmicki JA, Walters RK et al. (2020) The mutational constraint spectrum quantified from variation in 141,456 humans. *Nature* 581: 434-443

Kopanos C, Tsiolkas V, Kouris A, Chapple CE, Albarca Aguilera M, Meyer R, Massouras A (2019) VarSome: the human genomic variant search engine. *Bioinformatics* 35: 1978-1980

Peña-Chilet M, Roldán G, Perez-Florido J, Ortuño FM, Carmona R, Aquino V, Lopez-Lopez D, Loucera C, Fernandez-Rueda JL, Gallego A, García-Garcia F, González-Neira A, Pita G, Núñez-Torres R, Santoyo-López J, Ayuso C, Minguez P, Avila-Fernandez A, Corton M, Moreno-Pelayo M et al. (2020) CSVS, a crowdsourcing database of the Spanish population genetic variability. *Nucleic Acids Res*

Richards S, Aziz N, Bale S, Bick D, Das S, Gastier-Foster J, Grody WW, Hegde M, Lyon E, Spector E, Voelkerding K, Rehm HL, Committee ALQA (2015) Standards and guidelines for the interpretation of sequence variants: a joint consensus recommendation of the American College of Medical Genetics and Genomics and the Association for Molecular Pathology. *Genet Med* 17: 405-24

Wildeman M, van Ophuizen E, den Dunnen JT, Taschner PE (2008) Improving sequence variant descriptions in mutation databases and literature using the Mutalyzer sequence variation nomenclature checker. *Hum Mutat* 29: 6-13

Appendix Table S2. Primers for RT-qPCR analyses.

Genes	Primers	Sequences
Mouse *Gapdh*	primer F	5’-ACCACGAGAAATATGACAACTCAC-3’
	primer R	5’-CCAAAGTTGTCATGGATGACC-3’
Human *Gapdh*	primer F	5’-ATGACAACAGCCTCAAGAT-3’
	primer R	5’-GAGTCCTTCCACGATACC-3’
Canis *β-actin*	primer F	5’-GGACCTCTATGCCAACAC-3’
	primer R	5’-TGCGATGATCTTGATCTTCA-3’
EGFP	primer F	5’-TAAACGGCCACAAGTTCAGC-3’
	primer R	5’-GAACTTCAGGGTCAGCTTGC-3’
Human *Cingulin*	primer F	5’-CCAACCACTGGACCTCTA-3’
	primer R	5’-TCTGACGAGAACGGCTAA-3’
Canis *Cingulin*	primer F	5’-GTCCTTCAGTCCACCAAC-3’
	primer R	5’-TCGCTCAATCTCCTCTTCT-3’
Mouse *Cingulin*	primer F	5’-CAACTGCGGATGGAGAAG-3’
	primer R	5’-AACCTGGTGAGTATCTCTTGTA-3’
Canis *Claudin-2*	primer F	5’-CCGACTACTATGACTCCTACC-3’
	primer R	5’-TAAACTCGCTCTTGGCTTTG-3’
Canis *Claudin-3*	primer F	5’-GTGCAAGGTGTACGACTC-3’
	primer R	5’-CAGGATGGACACGACGAT-3’
Canis *Claudin-5*	primer F	5’-CCTTCCTGGACCACAACAT-3’
	primer R	5’-CCGAGTCGTACACCTTGC-3’
Canis *ZO-1*	primer F	5’-CGCAGTCCTATTCTTCAG-3’
	primer R	5’-ACTTCTGGCTTATGTTGAG-3’
Canis *ZO-2*	primer F	5’-GCACAGAGAACAGCAAGGA-3’
	primer R	5’-CACCAGCCAATCGGAGTC-3’
Canis *ZO-3*	primer F	5’-CTCATCCTACAGATCAATGGT-3’
	primer R	5’-CCCTCGGACTTCTCAATC-3’
Canis *GEF-H1*	primer F	5’-TAACAAGAGCATCACAGCCAAG-3’
	primer R	5’-TTCAGCAGAGCAGCCTTC-3’
Canis *RhoA*	primer F	5’-TGGTGATTGTTGGTGATGGA-3’
	primer R	5’-TCAATATCTGCCACATAGTTCTCA-3’
Canis *Occludin*	primer F	5’-CCAGGAGTAATTCGGATTCT-3’
	primer R	5’-CATTAAGCCAGTTCCATAGC-3’
Canis *ZONAB*	primer F	5’-ACTGCCATCAAGAAGAATAAC-3’
	primer R	5’-CGTCTGCCATAGTAACCA-3’
