# Supplementary figures and images for: Cingulin regulates hair cell cuticular plate morphology and is required for hearing in human and mouse (part 2 of 2)
Source: EMBO Mol Med. 2023 Sep 11;15(11):e17611. doi: 10.15252/emmm.202317611 (PMC10630877; doi:10.15252/emmm.202317611)

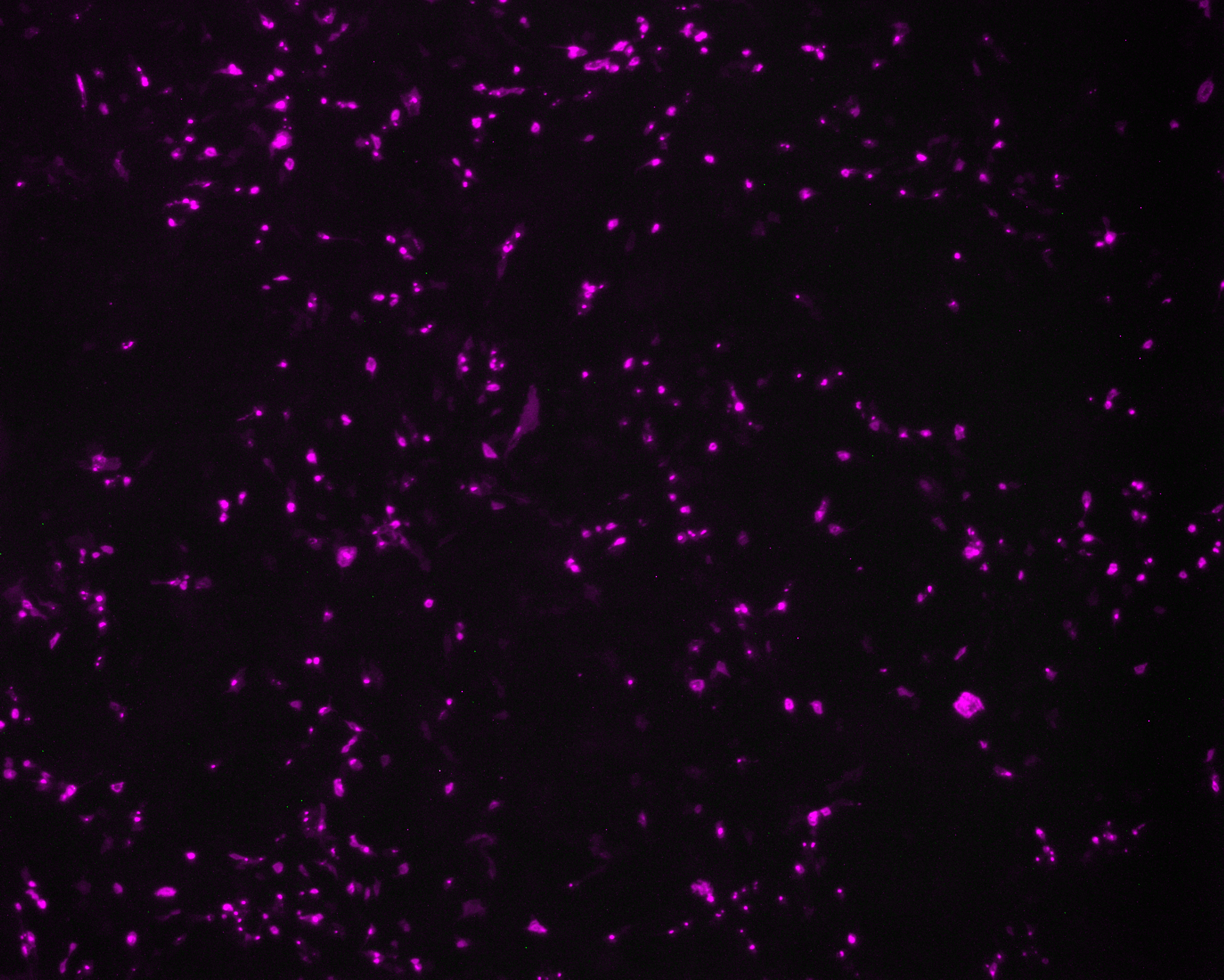

Supplement: Supplementary file 7 — Source Data for Figure 3 [file EMMM-15-e17611-s009.zip › Figure 3/3B/Mut CGN.tif]

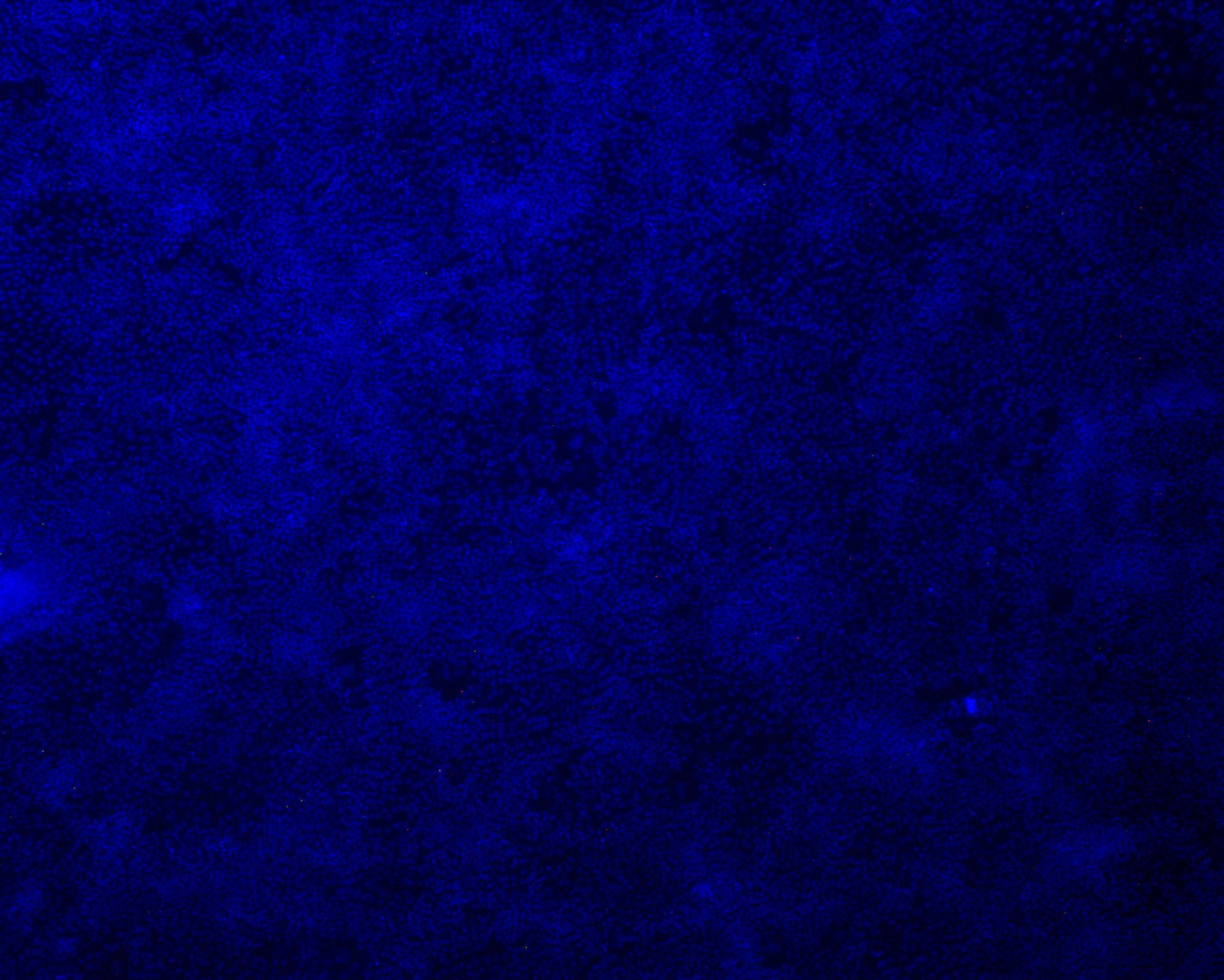

Supplement: Supplementary file 7 — Source Data for Figure 3 [file EMMM-15-e17611-s009.zip › Figure 3/3B/Mut DAPI.tif]

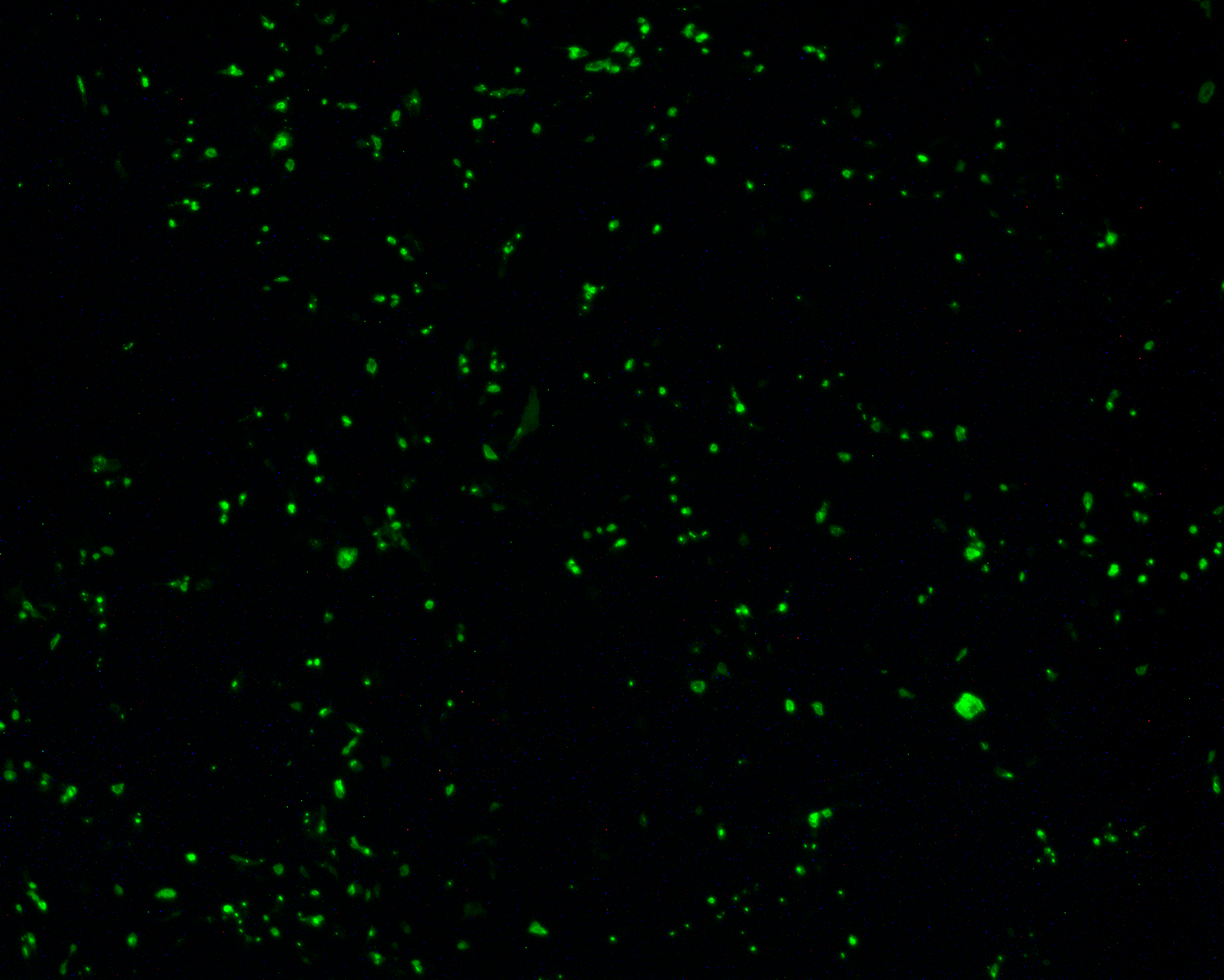

Supplement: Supplementary file 7 — Source Data for Figure 3 [file EMMM-15-e17611-s009.zip › Figure 3/3B/Mut Flag.tif]

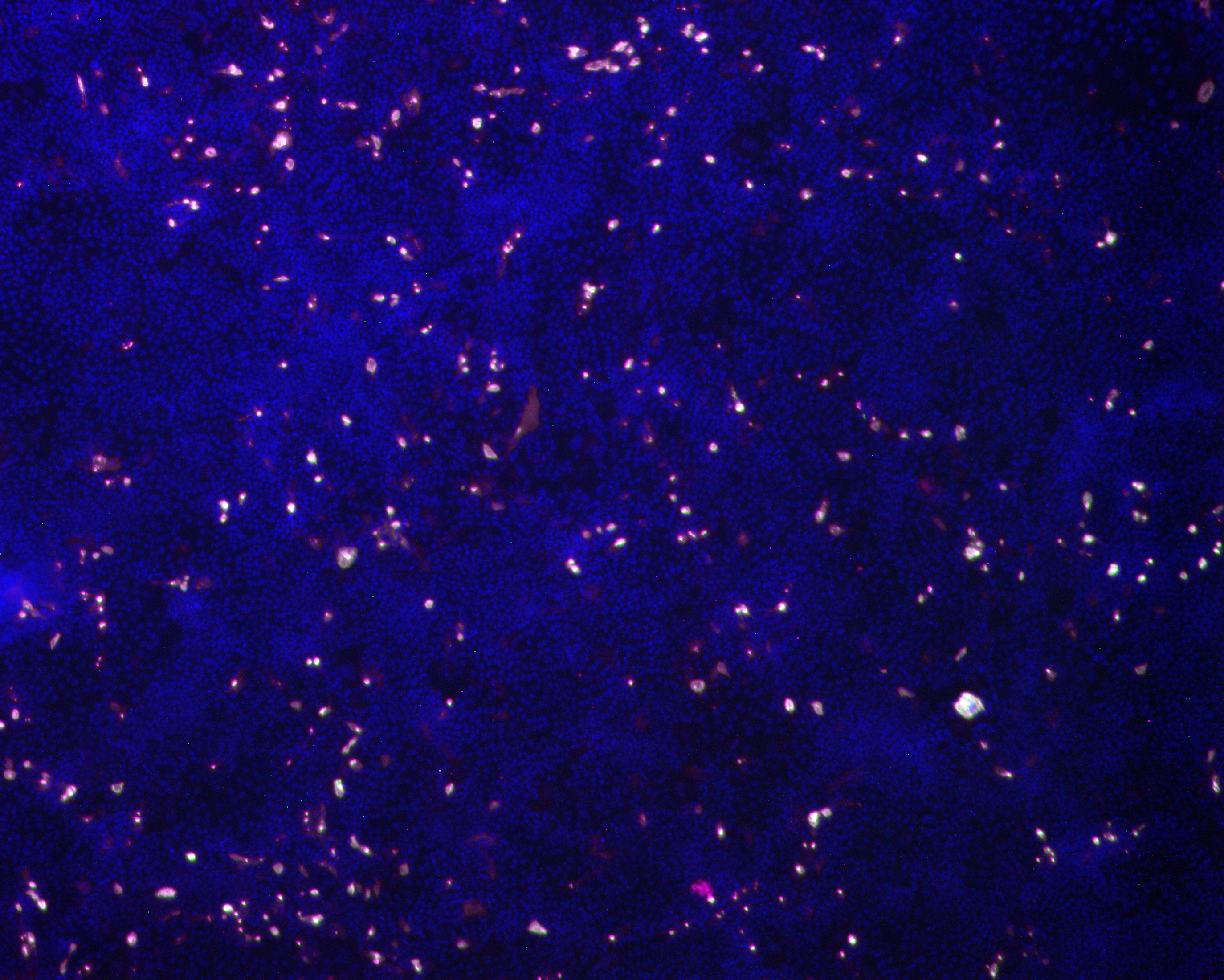

Supplement: Supplementary file 7 — Source Data for Figure 3 [file EMMM-15-e17611-s009.zip › Figure 3/3B/Mut Merge.tif]

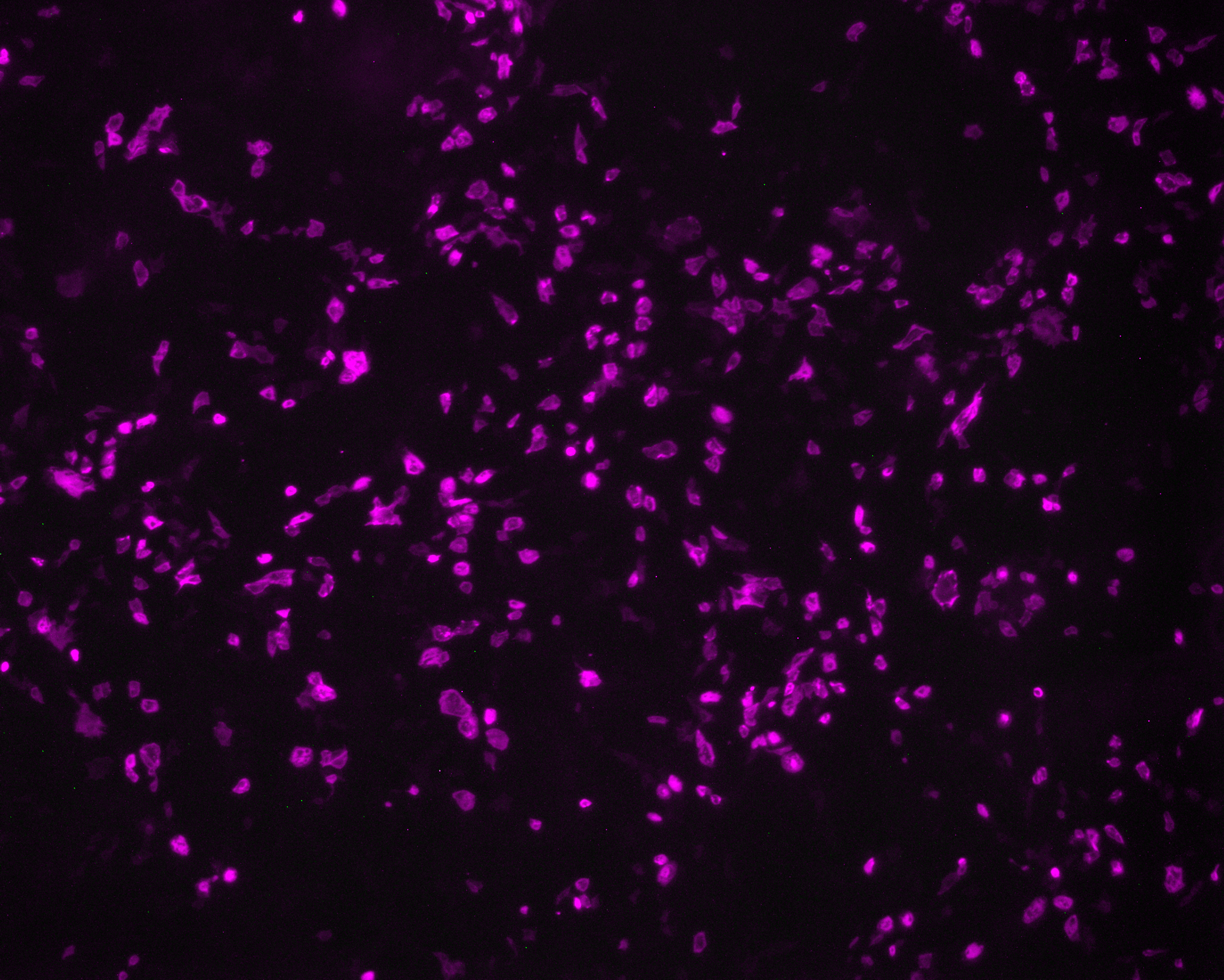

Supplement: Supplementary file 7 — Source Data for Figure 3 [file EMMM-15-e17611-s009.zip › Figure 3/3B/WT CGN.tif]

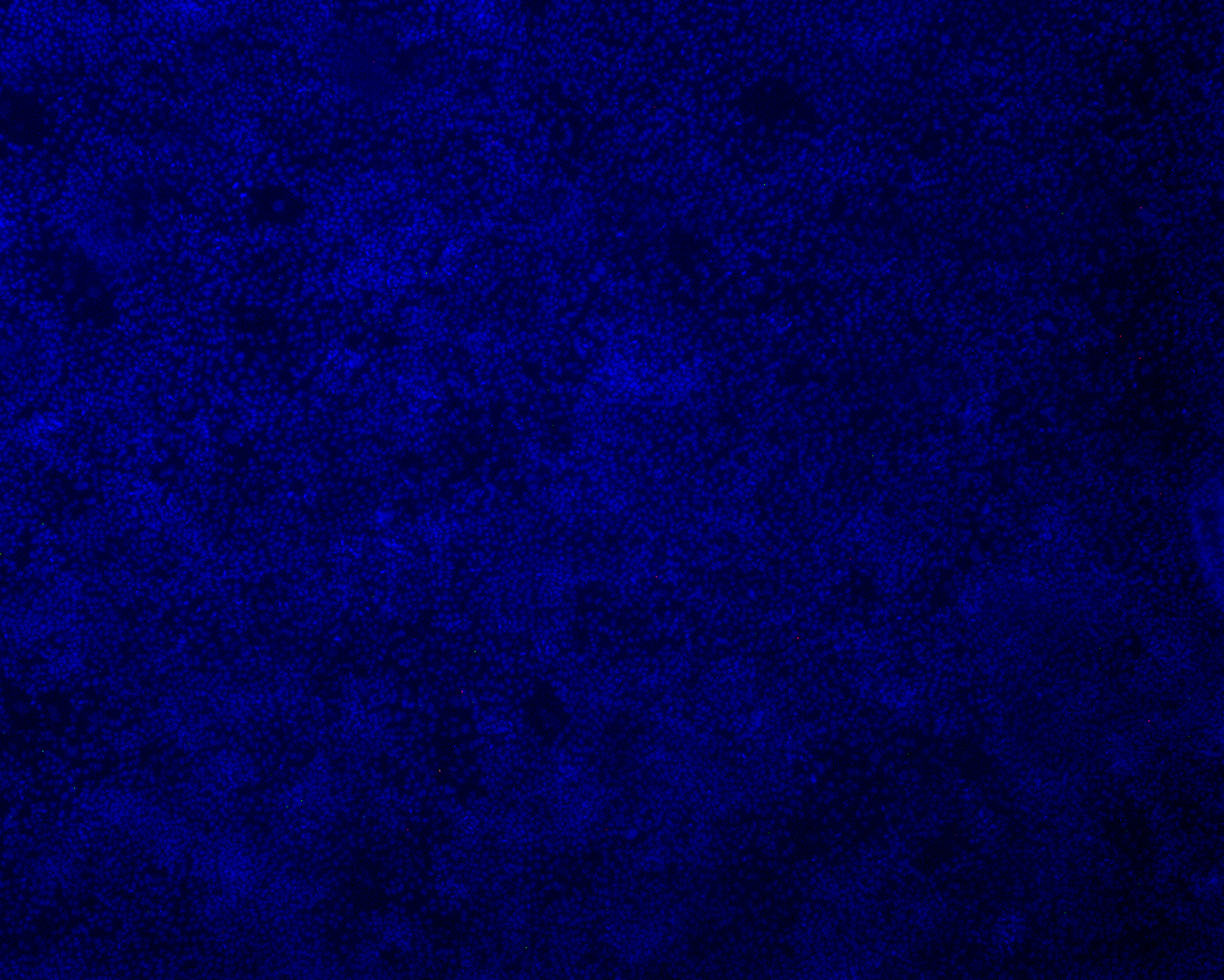

Supplement: Supplementary file 7 — Source Data for Figure 3 [file EMMM-15-e17611-s009.zip › Figure 3/3B/WT DAPI.tif]

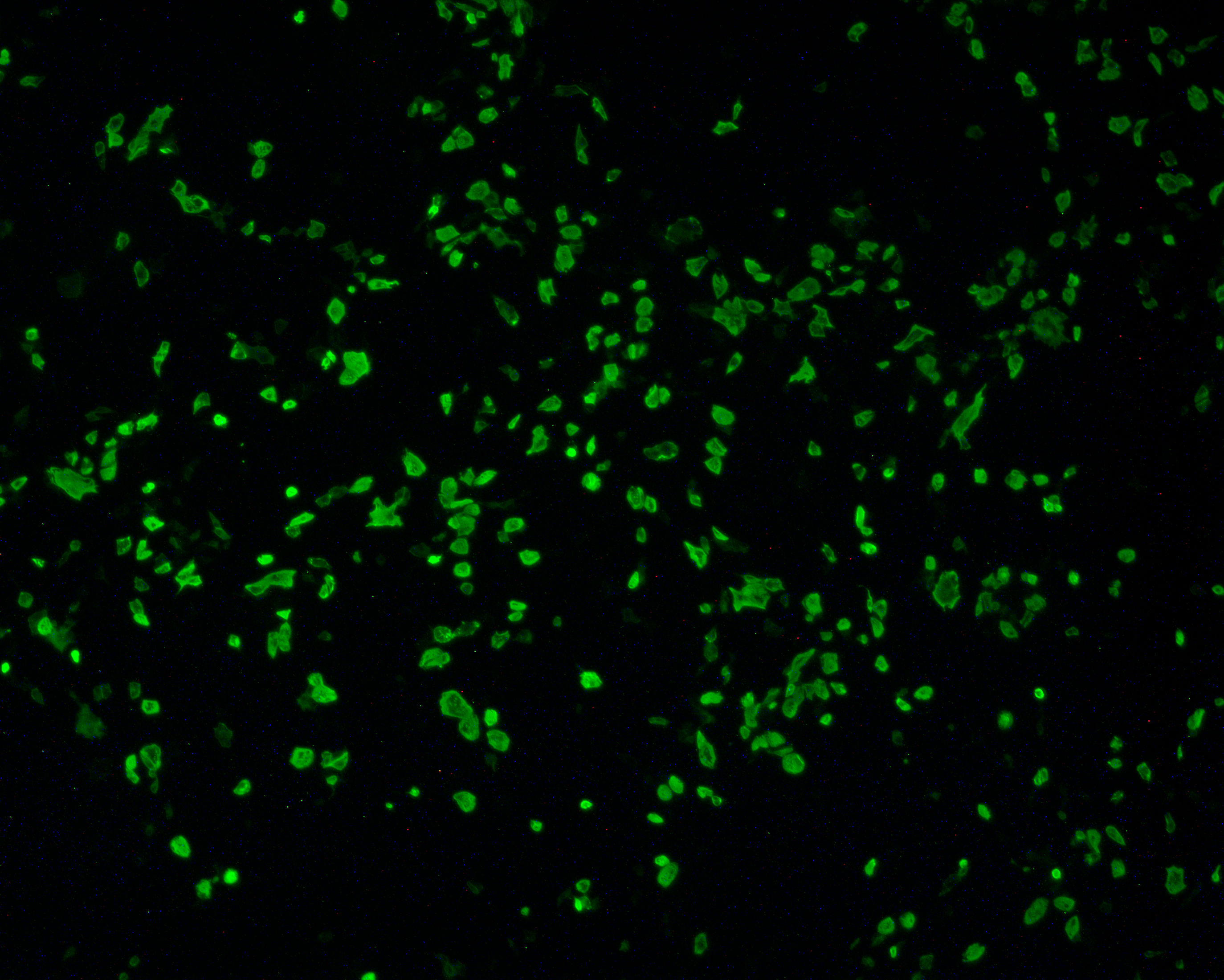

Supplement: Supplementary file 7 — Source Data for Figure 3 [file EMMM-15-e17611-s009.zip › Figure 3/3B/WT Flag.tif]

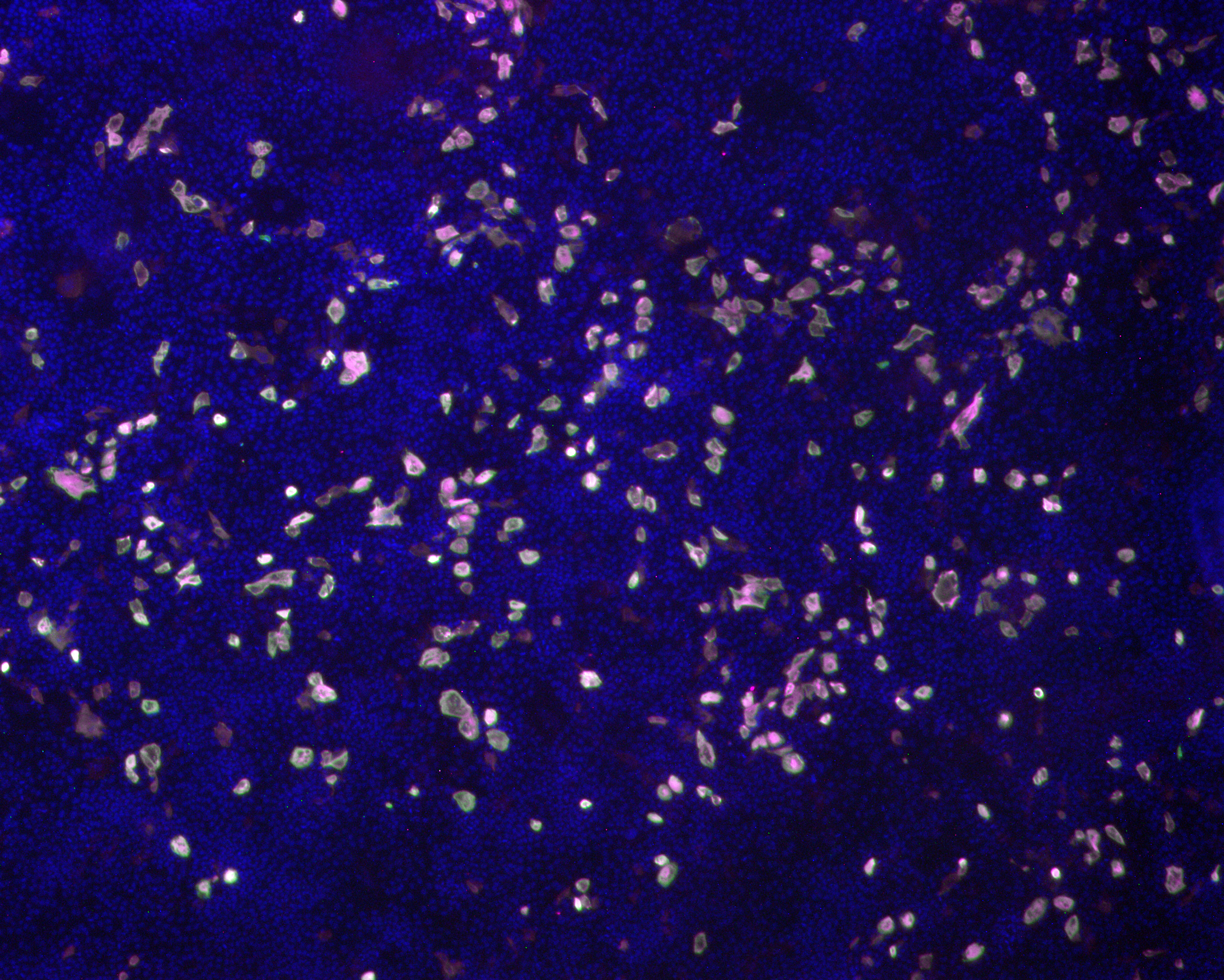

Supplement: Supplementary file 7 — Source Data for Figure 3 [file EMMM-15-e17611-s009.zip › Figure 3/3B/WT Merge.tif]

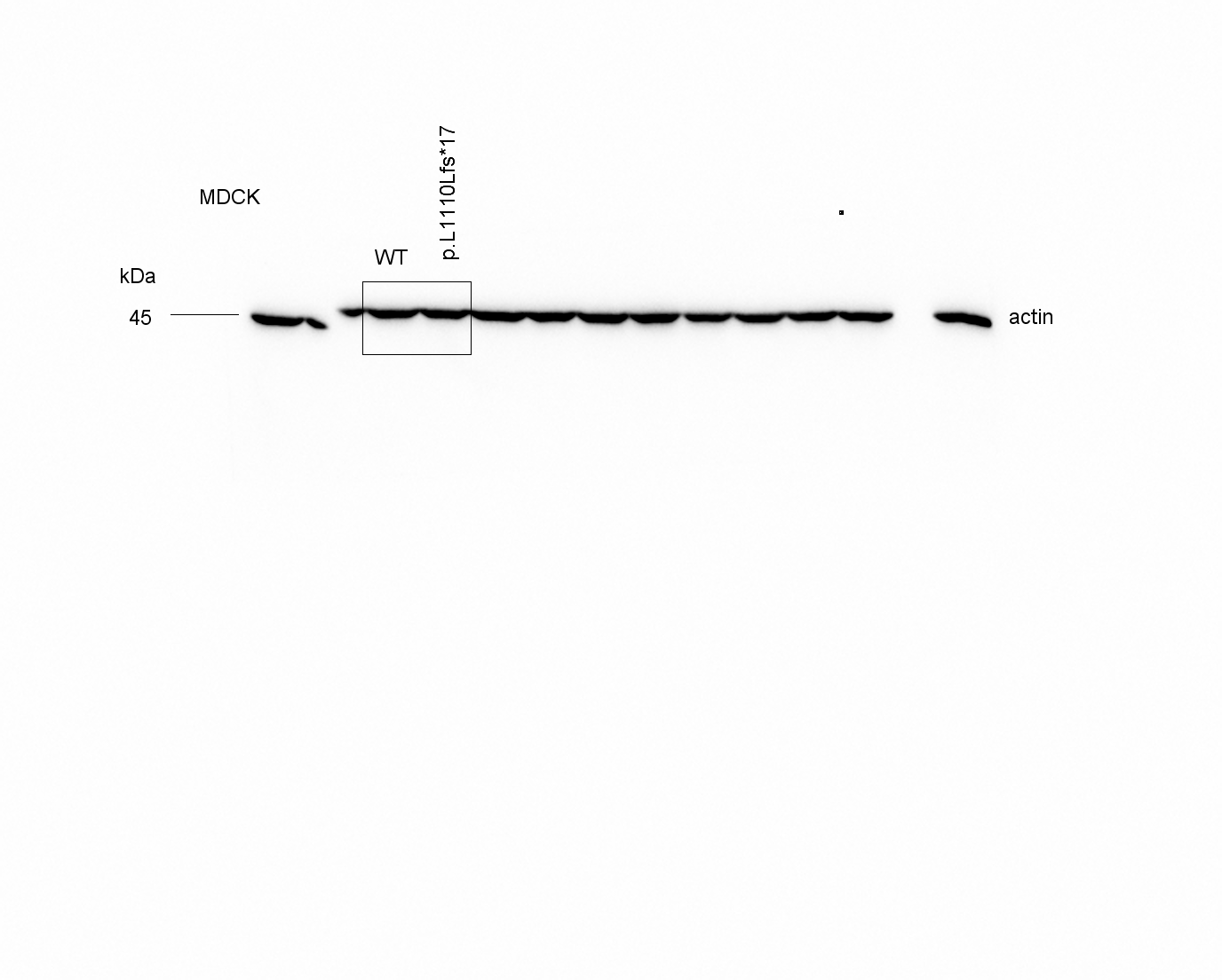

Supplement: Supplementary file 7 — Source Data for Figure 3 [file EMMM-15-e17611-s009.zip › Figure 3/3C/western actin.Tif]

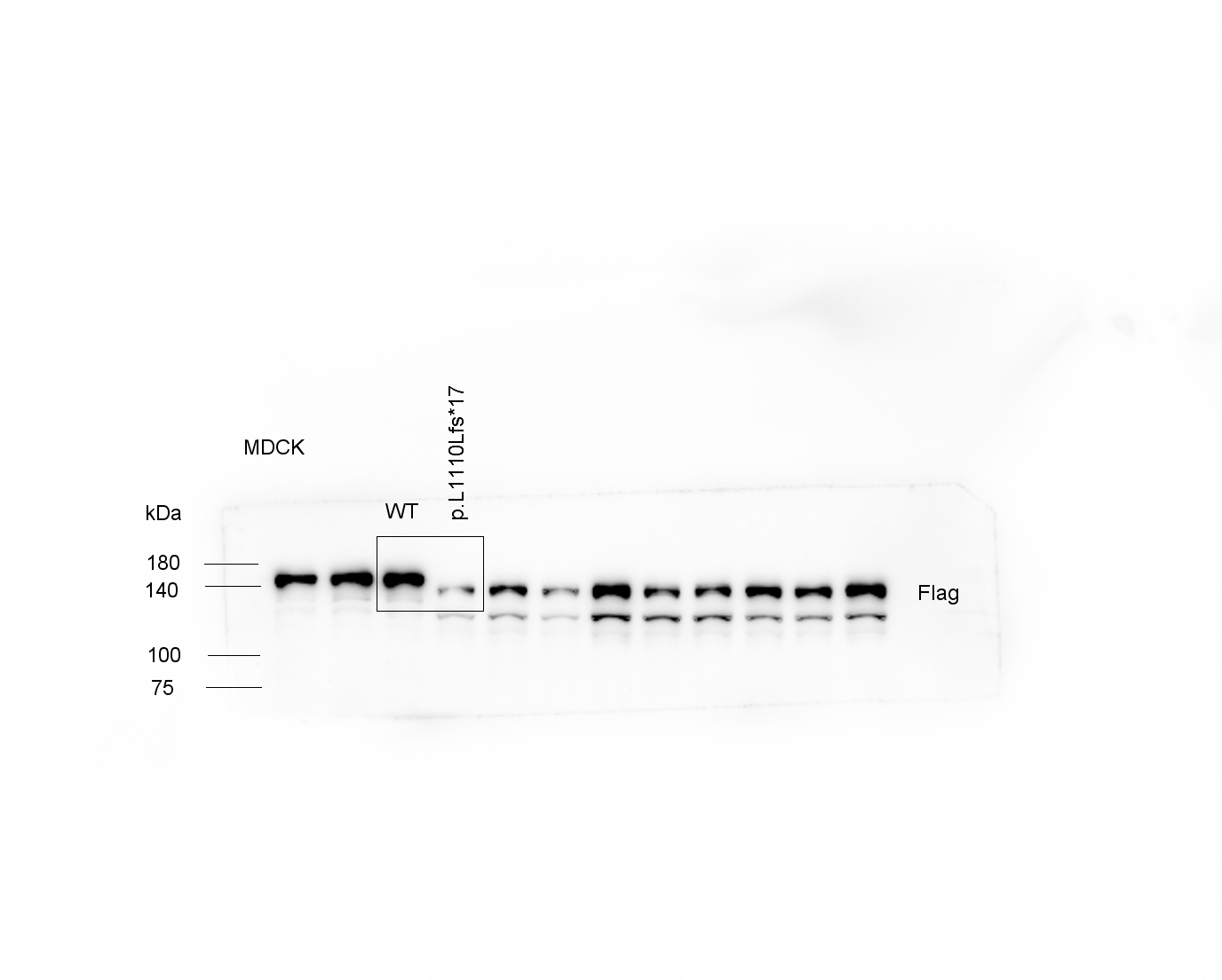

Supplement: Supplementary file 7 — Source Data for Figure 3 [file EMMM-15-e17611-s009.zip › Figure 3/3C/western Flag.Tif]

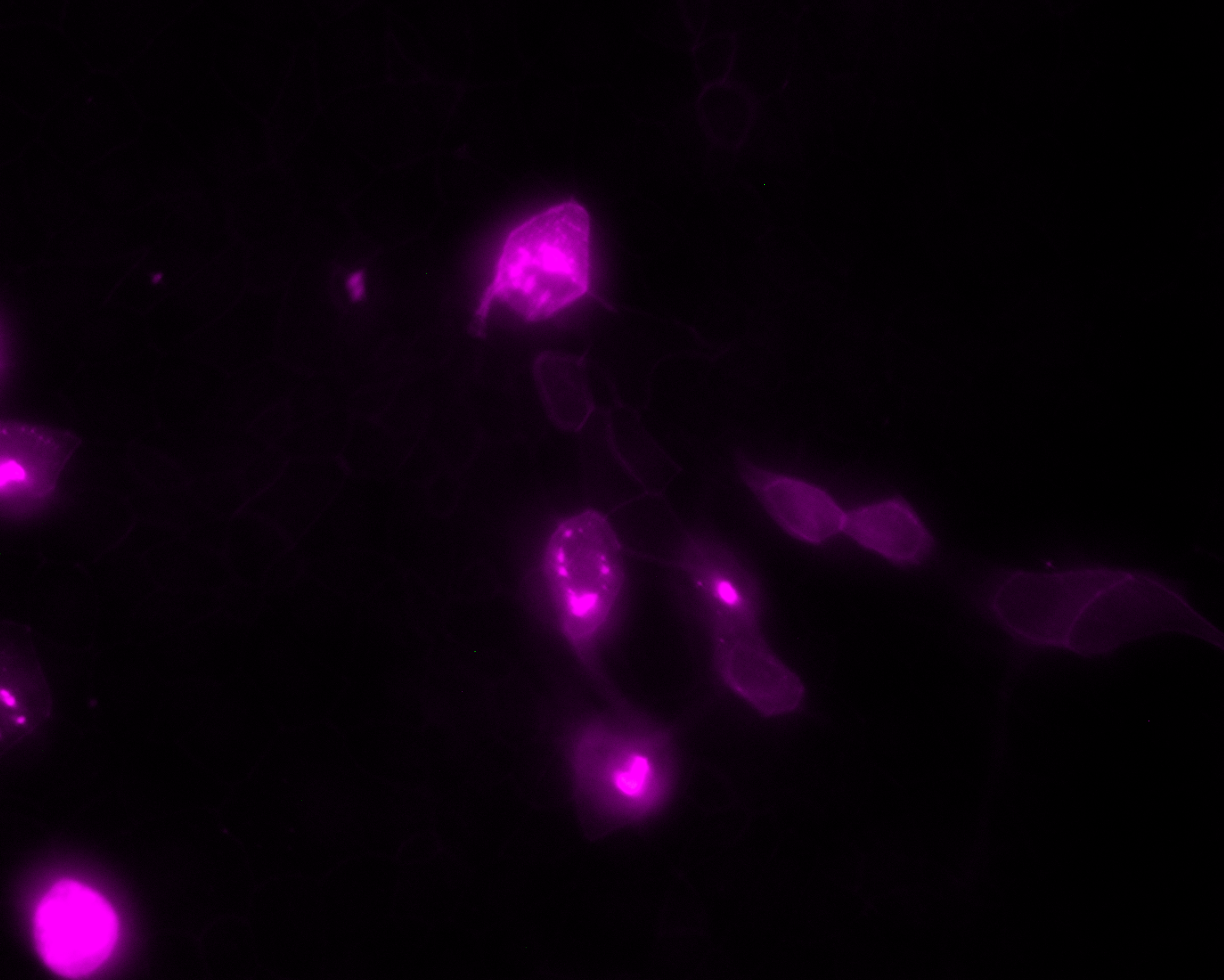

Supplement: Supplementary file 7 — Source Data for Figure 3 [file EMMM-15-e17611-s009.zip › Figure 3/3E/Mut CGN.tif]

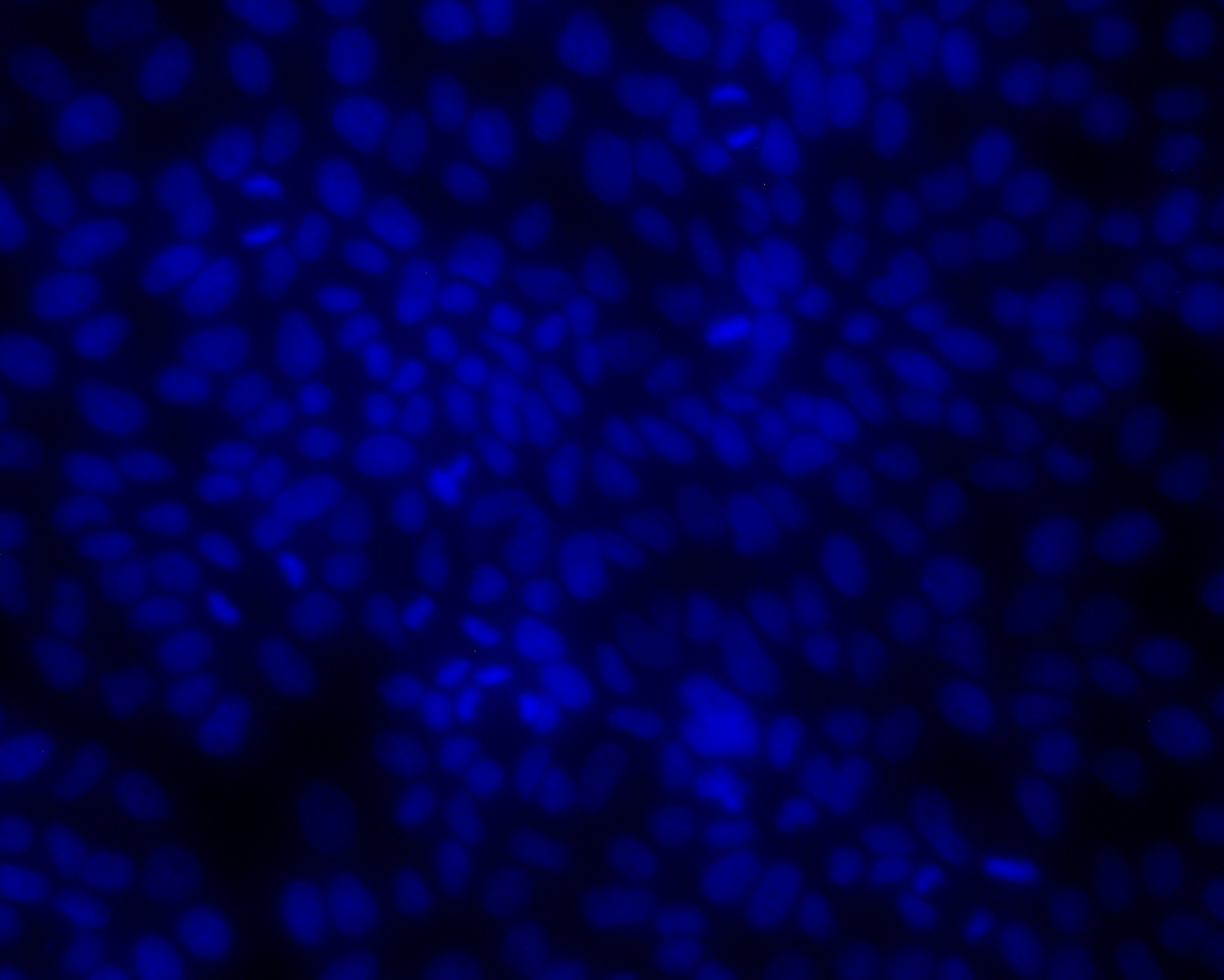

Supplement: Supplementary file 7 — Source Data for Figure 3 [file EMMM-15-e17611-s009.zip › Figure 3/3E/Mut DAPI.tif]

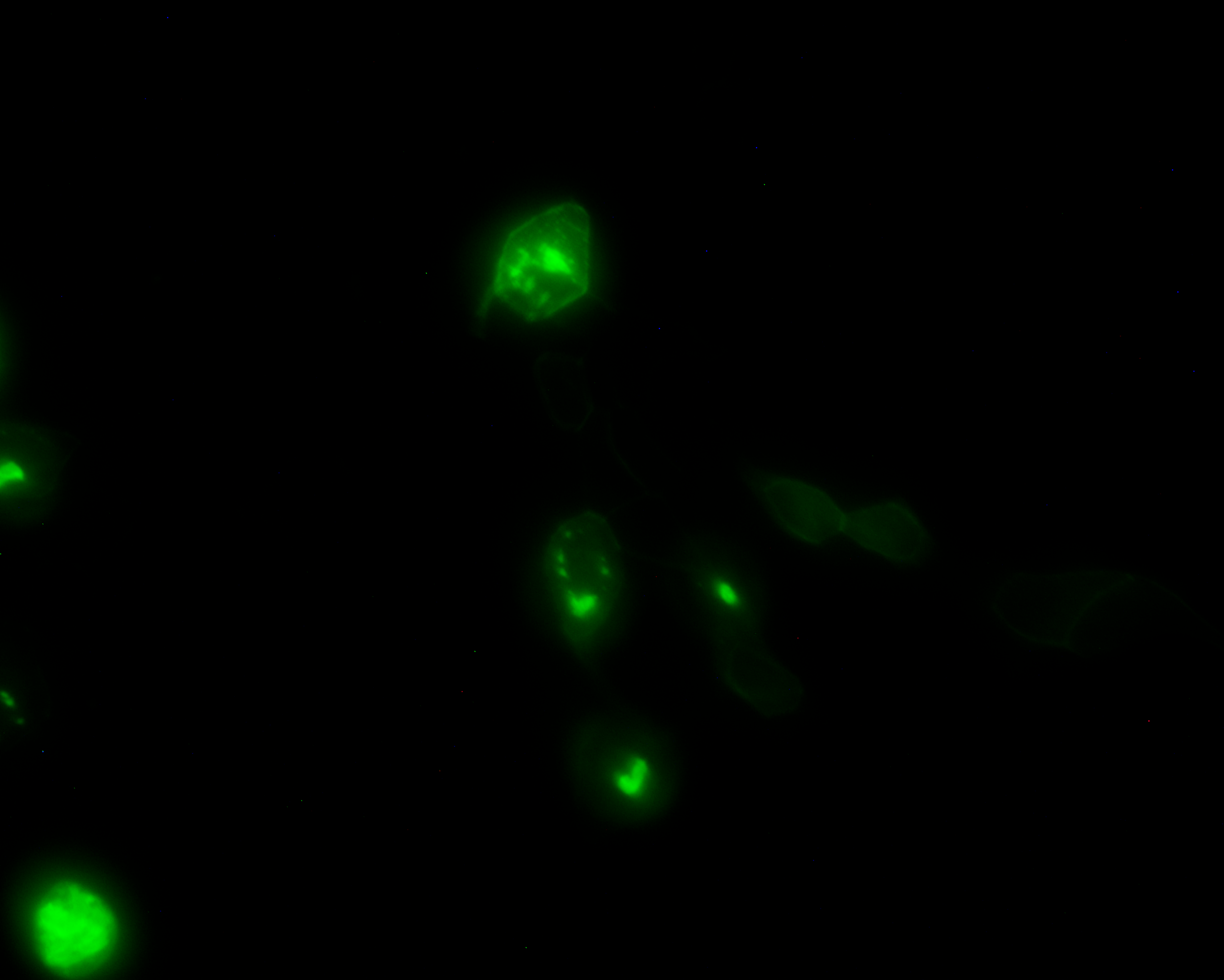

Supplement: Supplementary file 7 — Source Data for Figure 3 [file EMMM-15-e17611-s009.zip › Figure 3/3E/Mut Flag.tif]

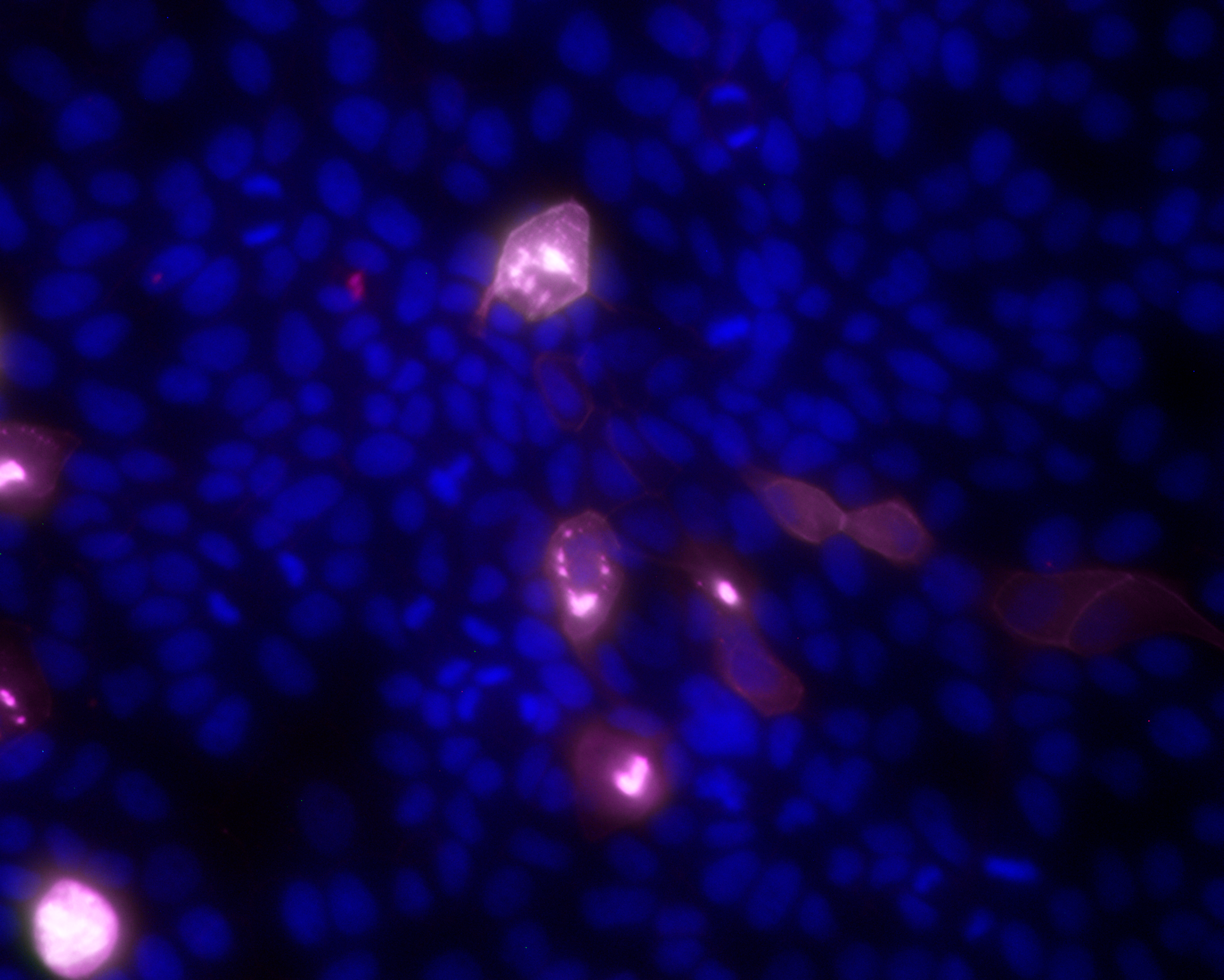

Supplement: Supplementary file 7 — Source Data for Figure 3 [file EMMM-15-e17611-s009.zip › Figure 3/3E/Mut Merge.tif]

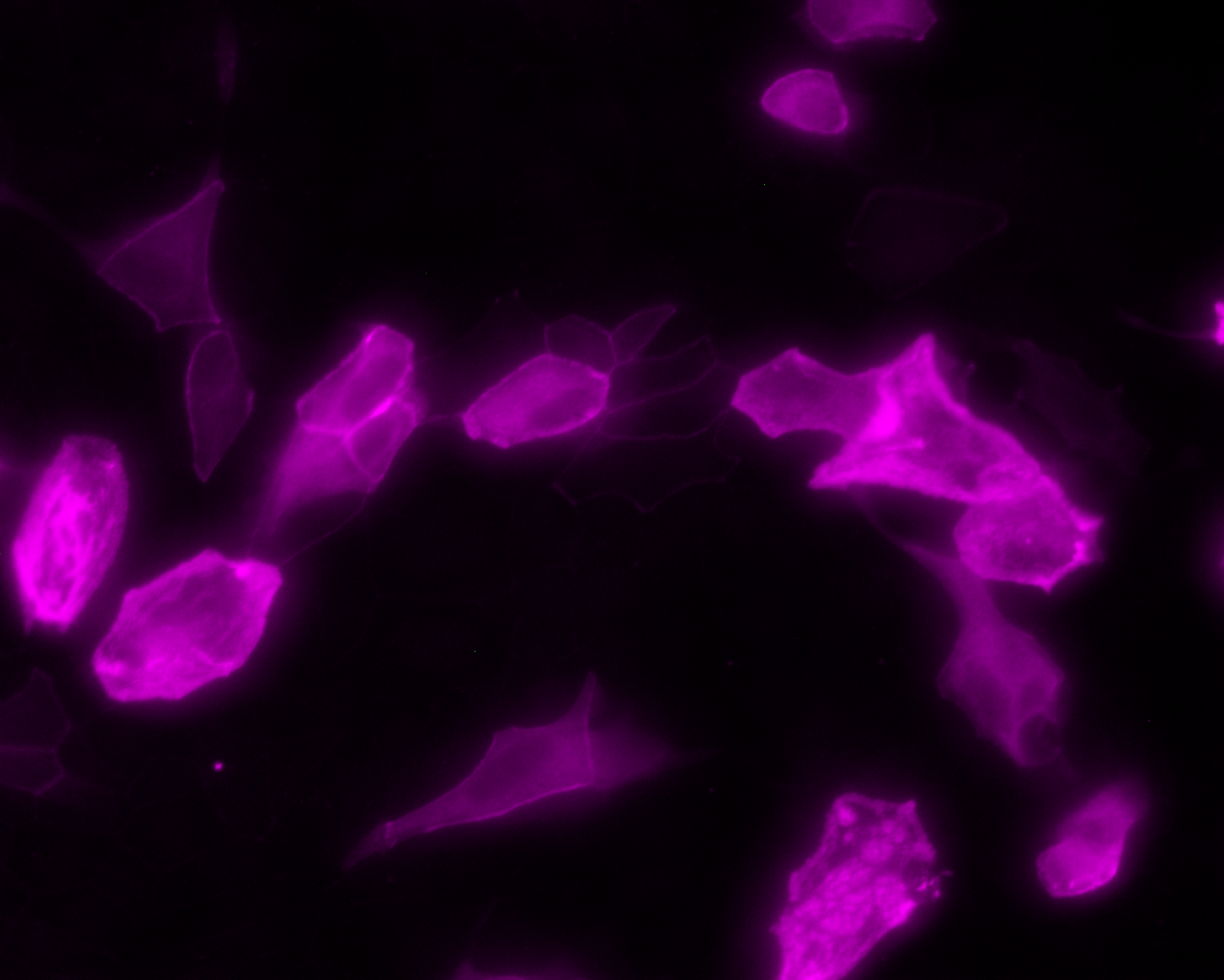

Supplement: Supplementary file 7 — Source Data for Figure 3 [file EMMM-15-e17611-s009.zip › Figure 3/3E/WT CGN.tif]

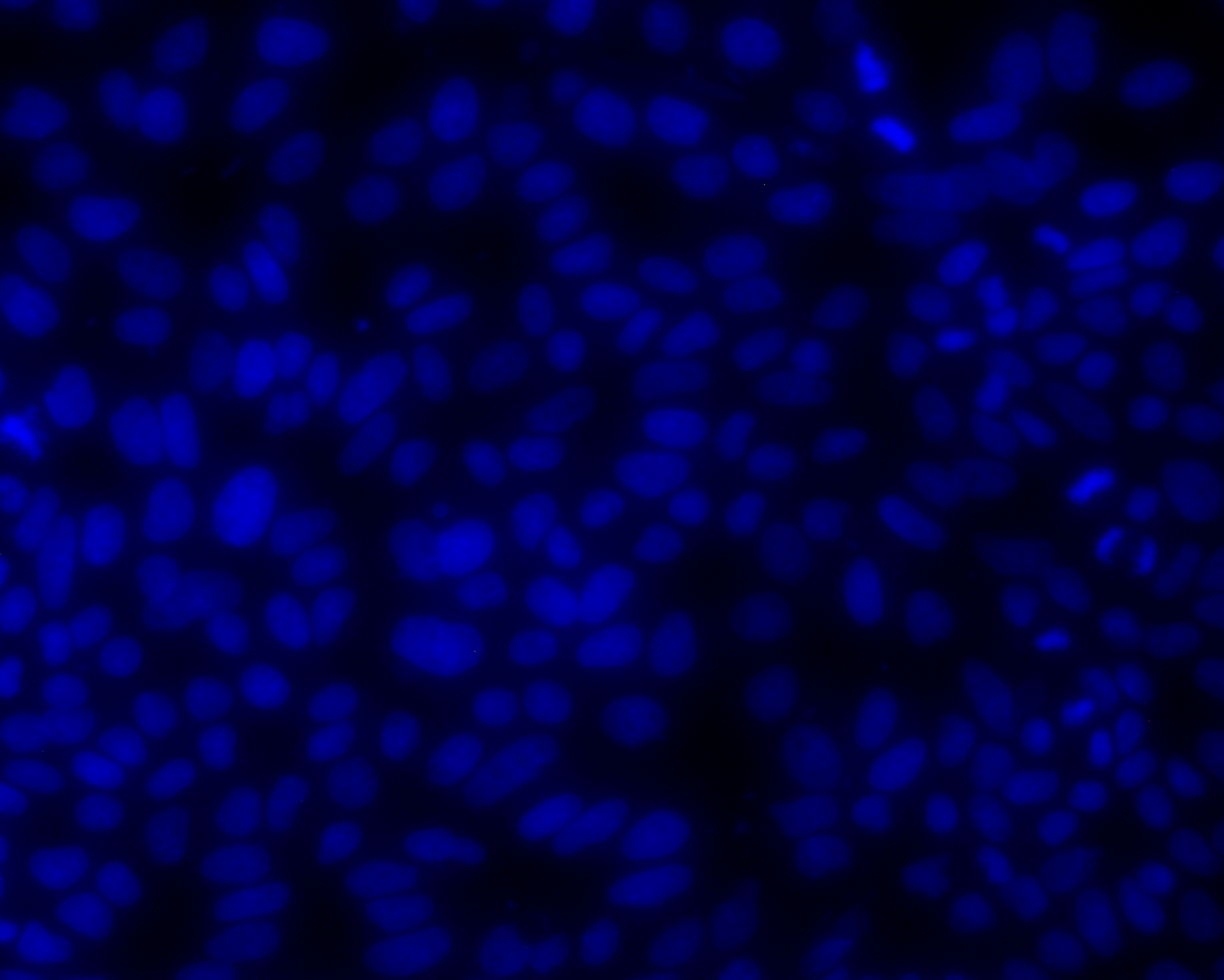

Supplement: Supplementary file 7 — Source Data for Figure 3 [file EMMM-15-e17611-s009.zip › Figure 3/3E/WT DAPI.tif]

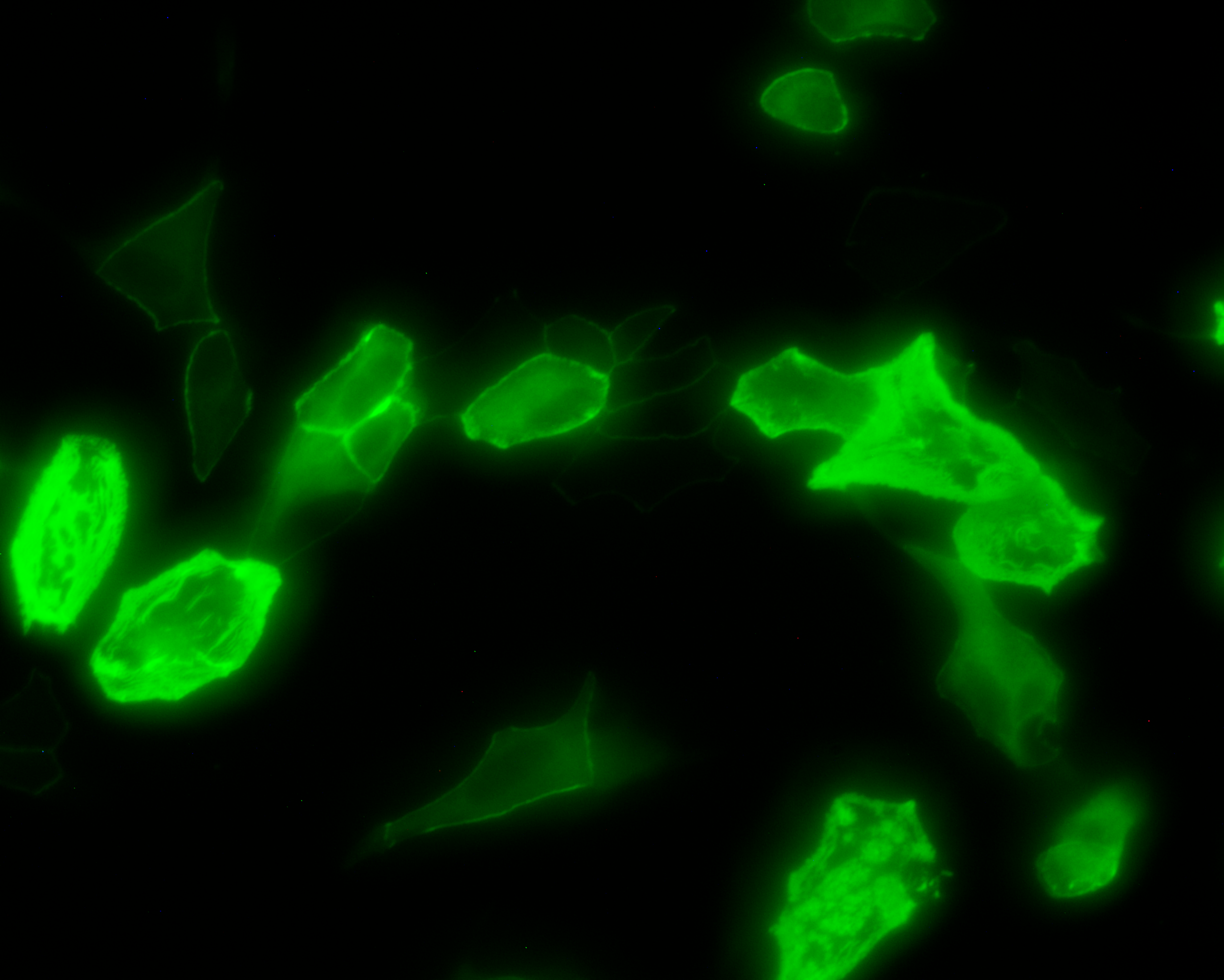

Supplement: Supplementary file 7 — Source Data for Figure 3 [file EMMM-15-e17611-s009.zip › Figure 3/3E/WT Flag.tif]

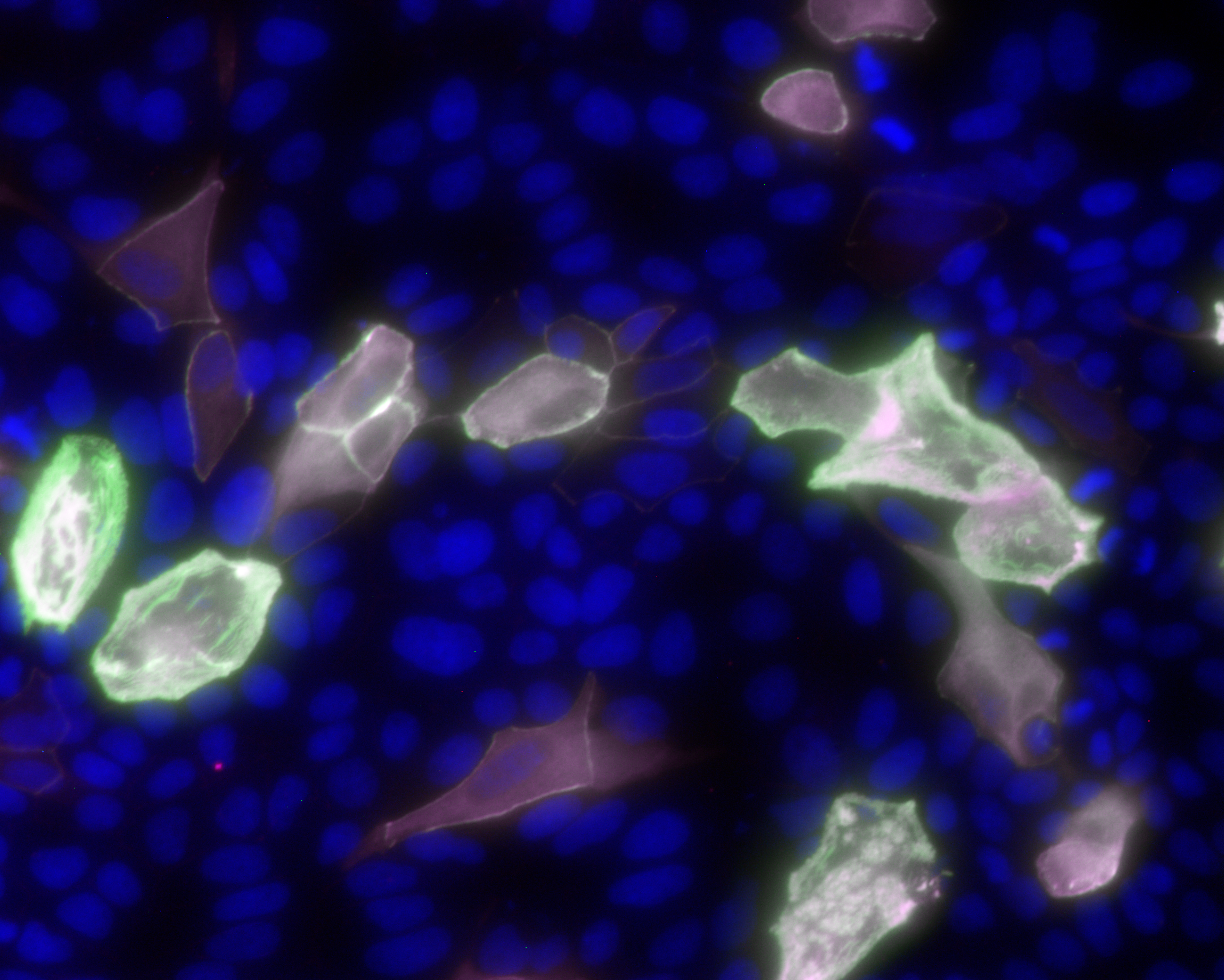

Supplement: Supplementary file 7 — Source Data for Figure 3 [file EMMM-15-e17611-s009.zip › Figure 3/3E/WT Merge.tif]

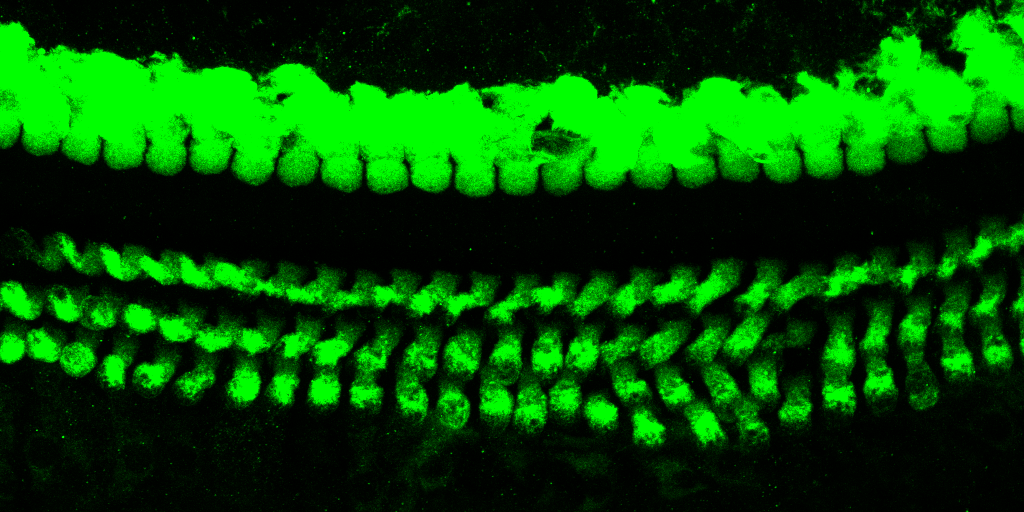

Supplement: Supplementary file 8 — Source Data for Figure 4 [file EMMM-15-e17611-s007.zip › Figure 4/4J/Cgn-fl 16kHz Myosin7a.tif]

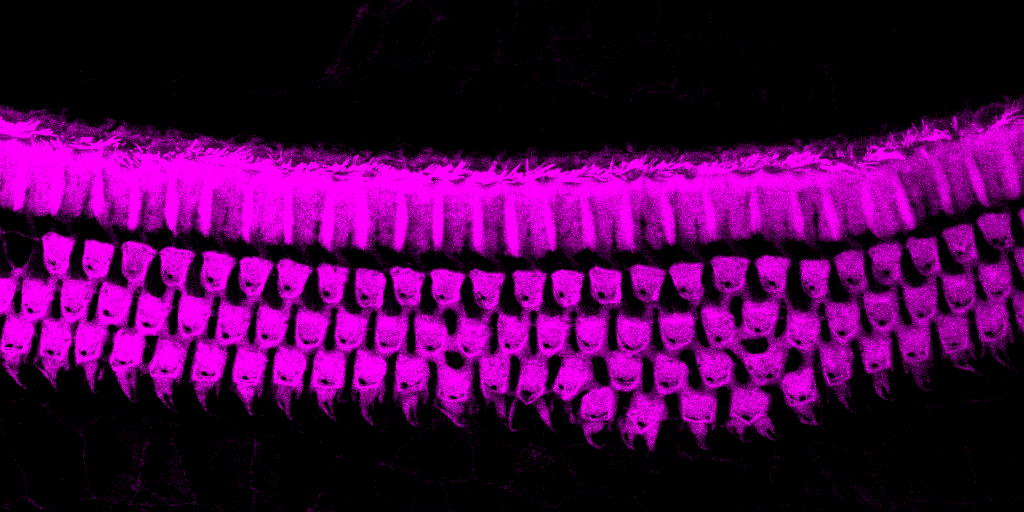

Supplement: Supplementary file 8 — Source Data for Figure 4 [file EMMM-15-e17611-s007.zip › Figure 4/4J/Cgn-fl 16kHz phalloidin.tif]

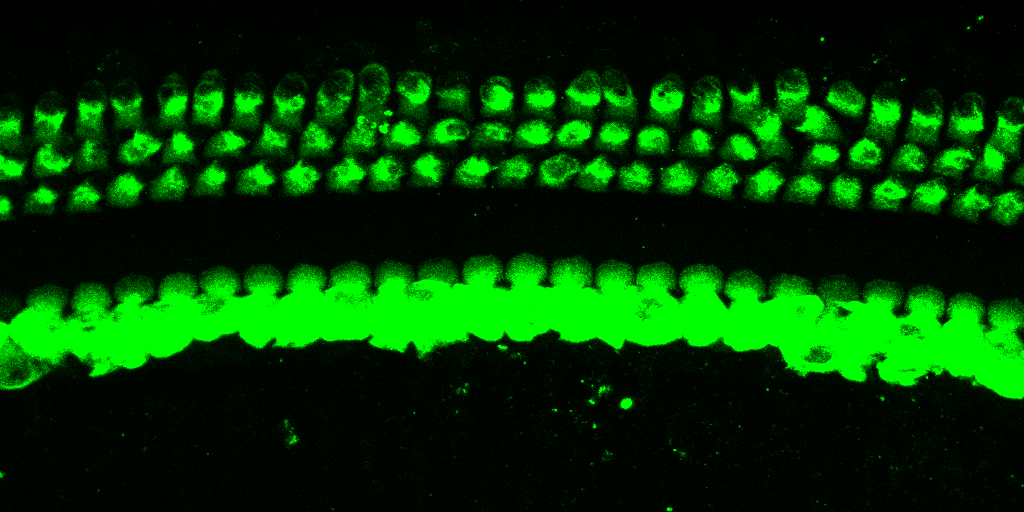

Supplement: Supplementary file 8 — Source Data for Figure 4 [file EMMM-15-e17611-s007.zip › Figure 4/4J/Cgn-fl 32kHz Myosin7a.tif]

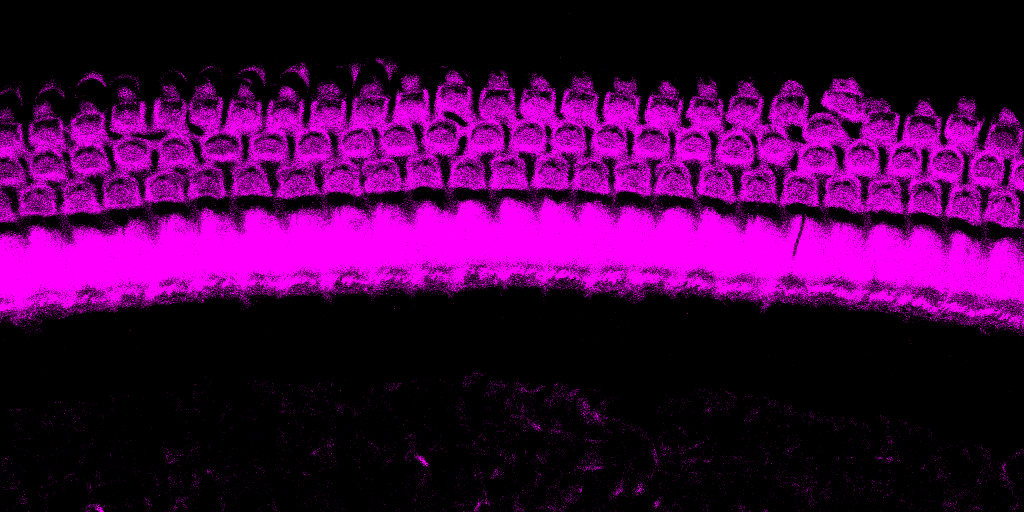

Supplement: Supplementary file 8 — Source Data for Figure 4 [file EMMM-15-e17611-s007.zip › Figure 4/4J/Cgn-fl 32kHz phalloidin.tif]

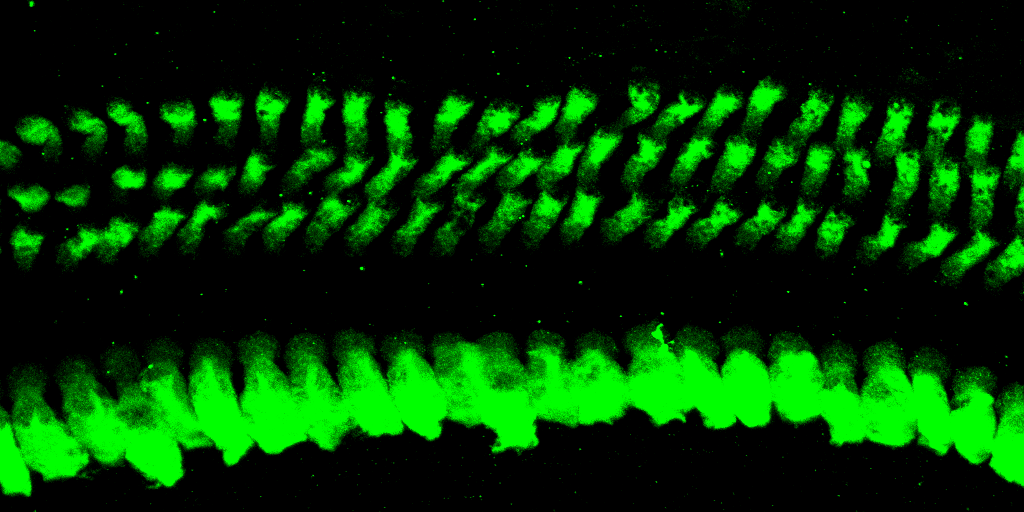

Supplement: Supplementary file 8 — Source Data for Figure 4 [file EMMM-15-e17611-s007.zip › Figure 4/4J/Cgn-fl 8kHz Myosin7a.tif]

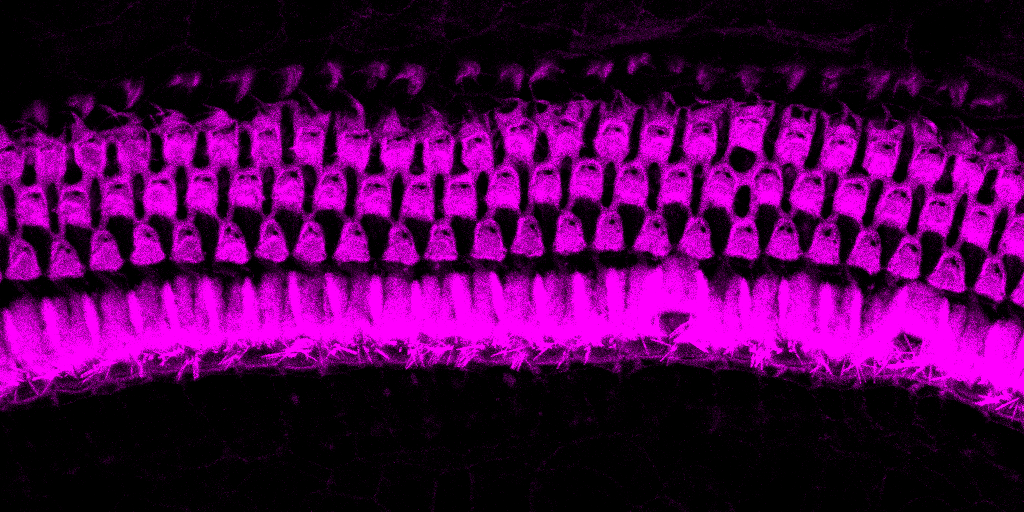

Supplement: Supplementary file 8 — Source Data for Figure 4 [file EMMM-15-e17611-s007.zip › Figure 4/4J/Cgn-fl 8kHz phalloidin.tif]

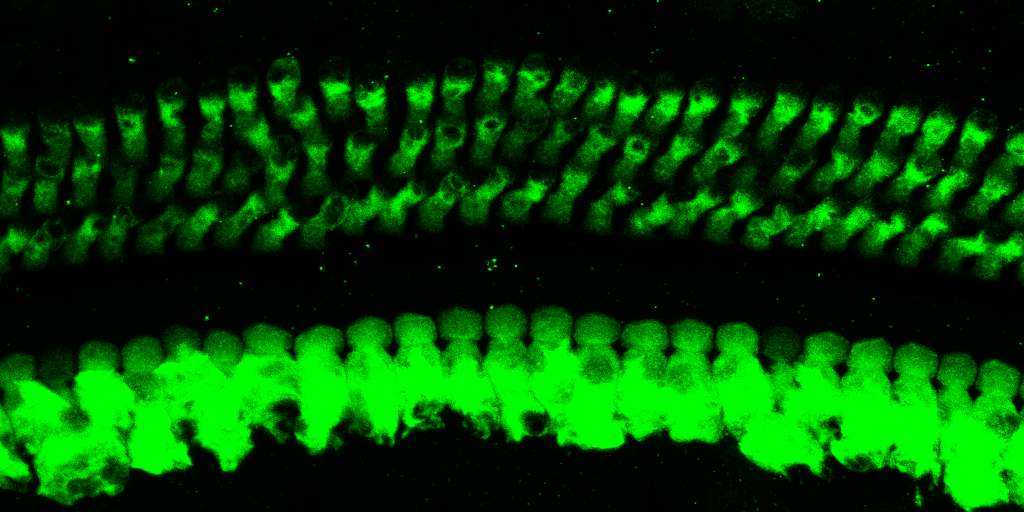

Supplement: Supplementary file 8 — Source Data for Figure 4 [file EMMM-15-e17611-s007.zip › Figure 4/4J/Cgn-fl-Pou4f3-creER 16kHz Myosin7a.tif]

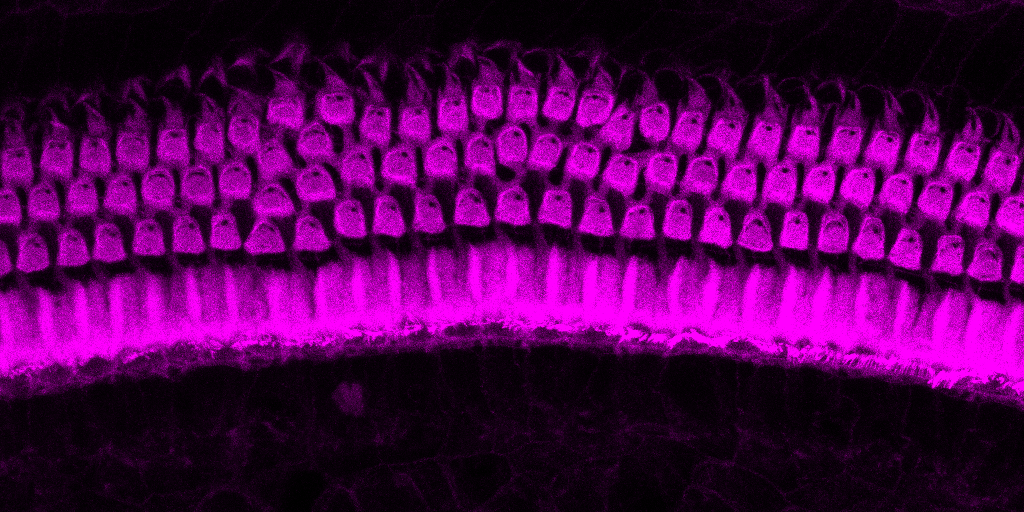

Supplement: Supplementary file 8 — Source Data for Figure 4 [file EMMM-15-e17611-s007.zip › Figure 4/4J/Cgn-fl-Pou4f3-creER 16kHz phalloidin.tif]

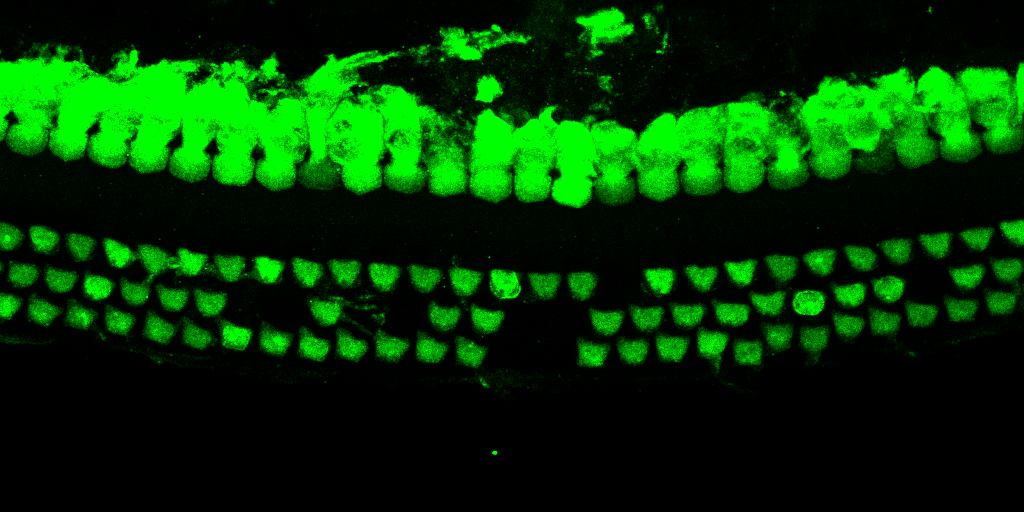

Supplement: Supplementary file 8 — Source Data for Figure 4 [file EMMM-15-e17611-s007.zip › Figure 4/4J/Cgn-fl-Pou4f3-creER 32kHz Myosin7a.tif]

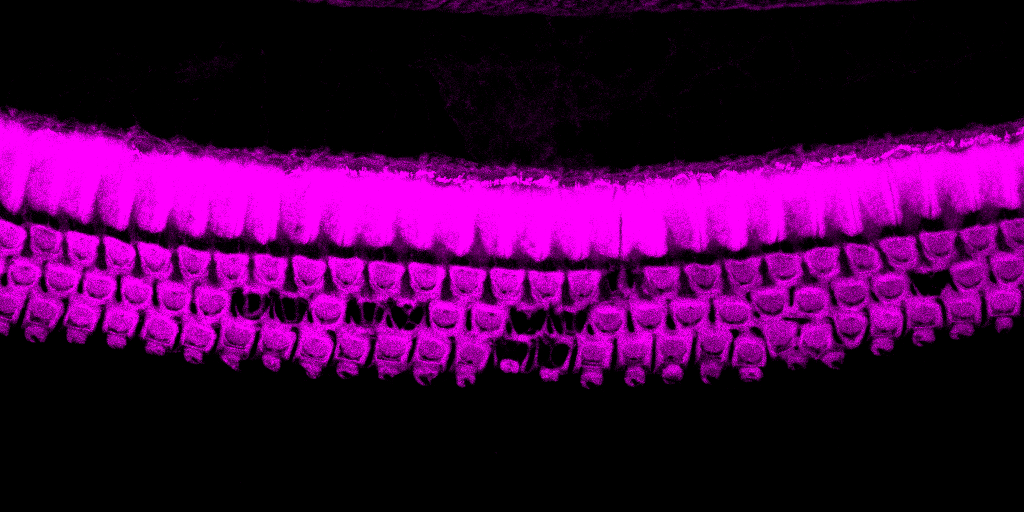

Supplement: Supplementary file 8 — Source Data for Figure 4 [file EMMM-15-e17611-s007.zip › Figure 4/4J/Cgn-fl-Pou4f3-creER 32kHz phalloidin.tif]

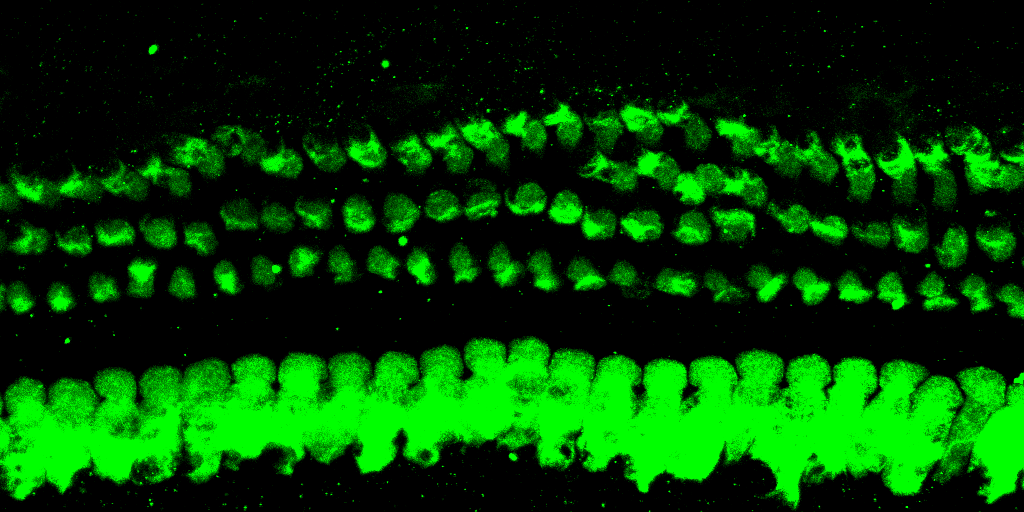

Supplement: Supplementary file 8 — Source Data for Figure 4 [file EMMM-15-e17611-s007.zip › Figure 4/4J/Cgn-fl-Pou4f3-creER 8kHz Myosin7a.tif]

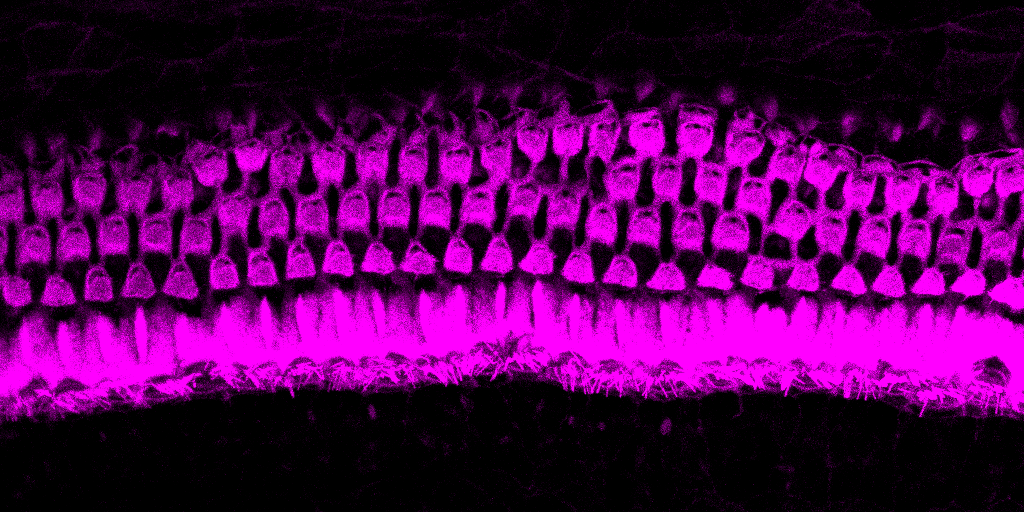

Supplement: Supplementary file 8 — Source Data for Figure 4 [file EMMM-15-e17611-s007.zip › Figure 4/4J/Cgn-fl-Pou4f3-creER 8kHz phalloidin.tif]

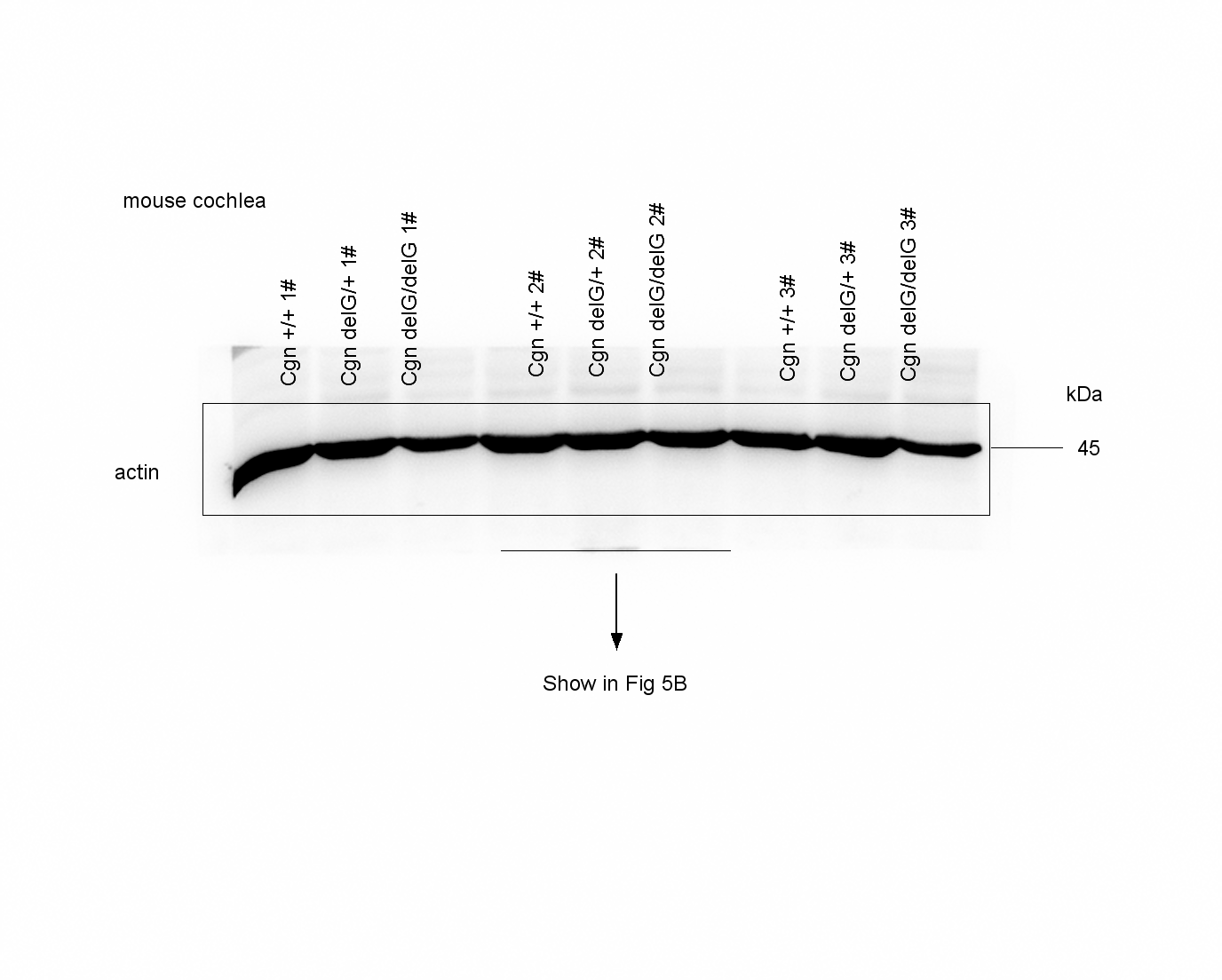

Supplement: Supplementary file 9 — Source Data for Figure 5 [file EMMM-15-e17611-s006.zip › Figure 5/5B/Quantification of Cgn protein expression Western blot/western actin 1.tif]

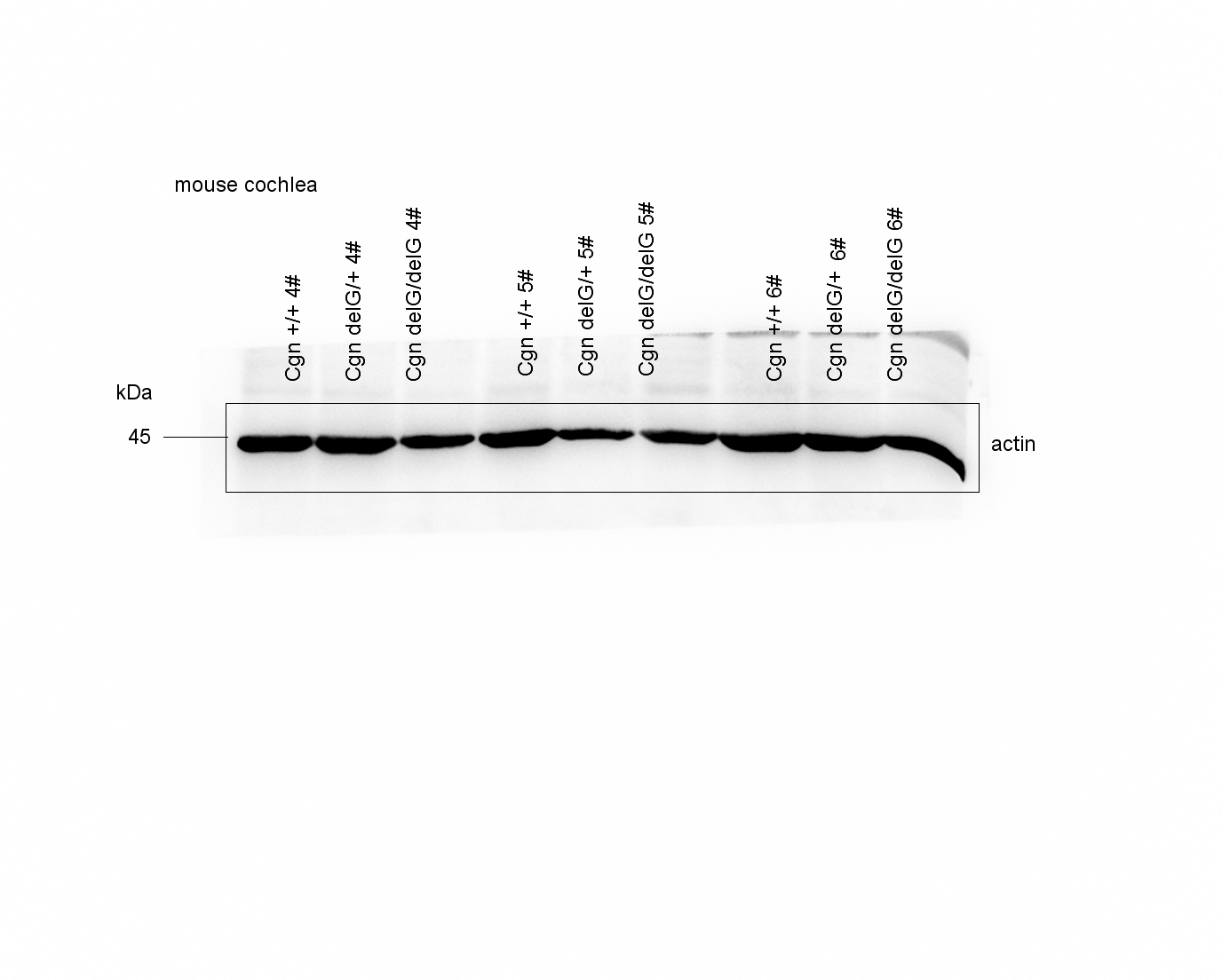

Supplement: Supplementary file 9 — Source Data for Figure 5 [file EMMM-15-e17611-s006.zip › Figure 5/5B/Quantification of Cgn protein expression Western blot/western actin 2.tif]

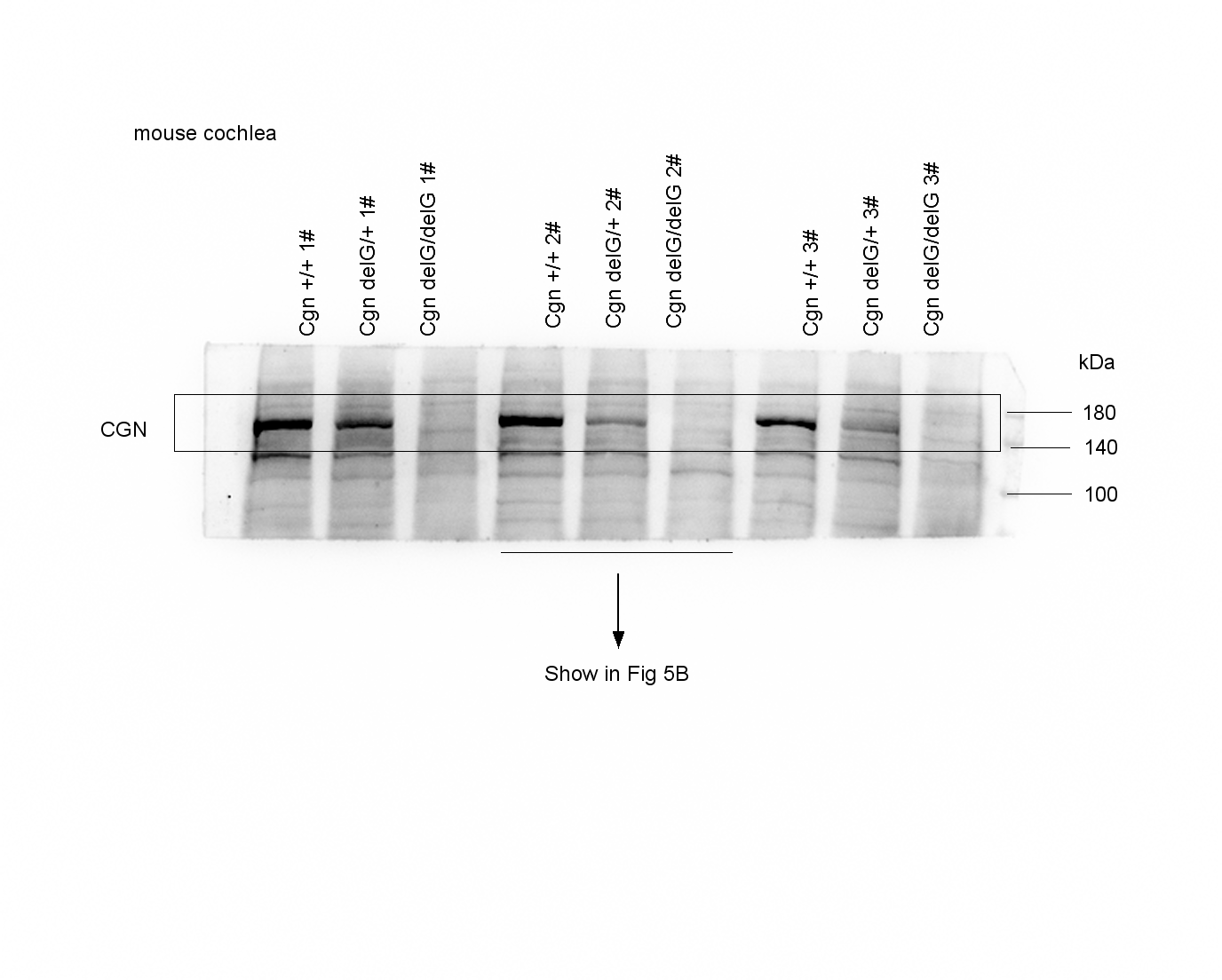

Supplement: Supplementary file 9 — Source Data for Figure 5 [file EMMM-15-e17611-s006.zip › Figure 5/5B/Quantification of Cgn protein expression Western blot/western CGN 1.tif]

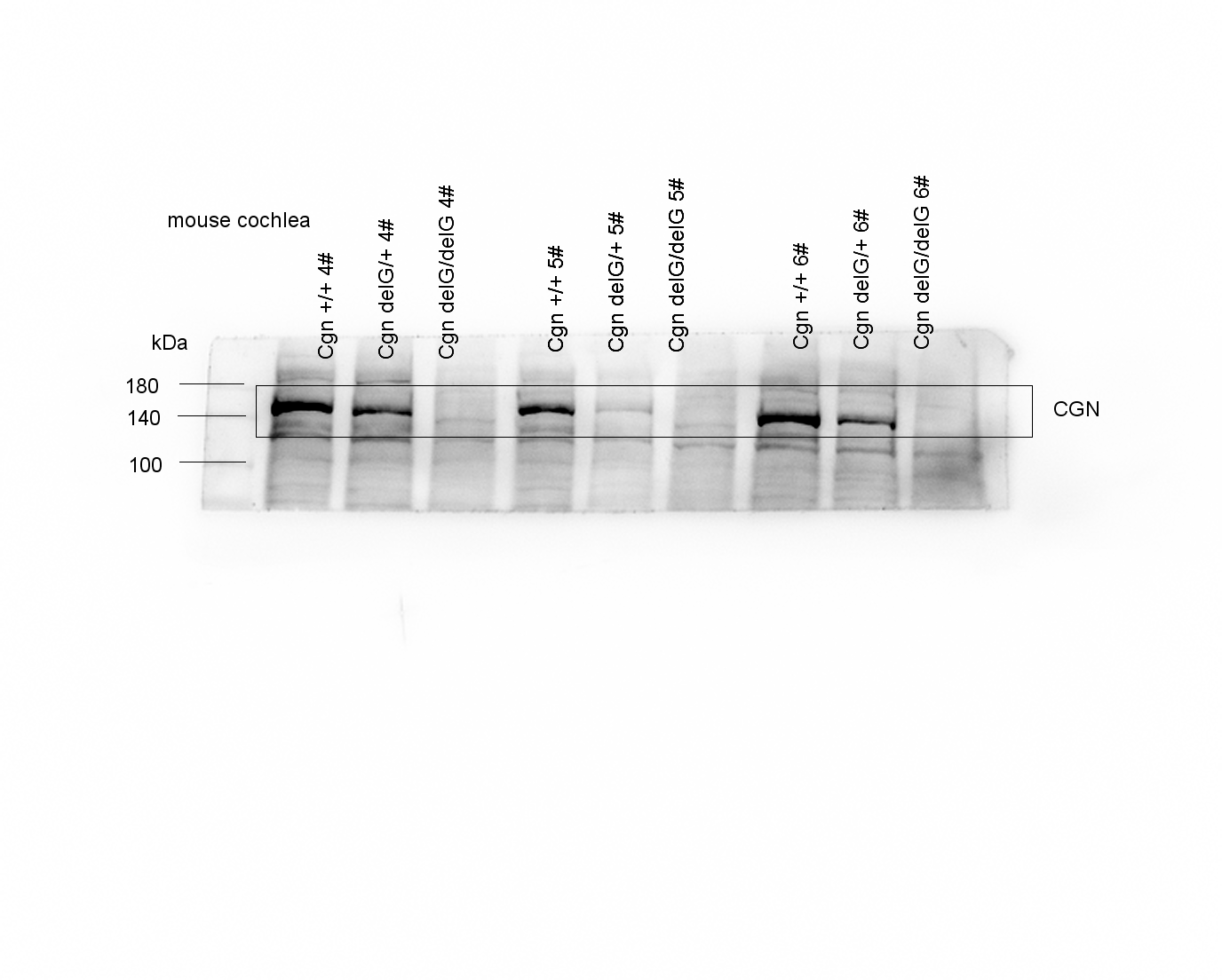

Supplement: Supplementary file 9 — Source Data for Figure 5 [file EMMM-15-e17611-s006.zip › Figure 5/5B/Quantification of Cgn protein expression Western blot/western CGN 2.tif]

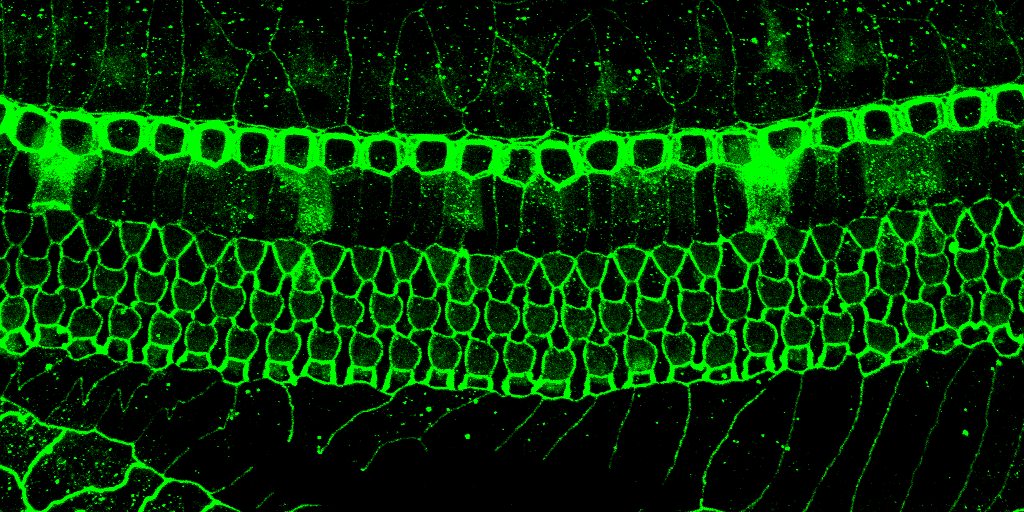

Supplement: Supplementary file 9 — Source Data for Figure 5 [file EMMM-15-e17611-s006.zip › Figure 5/5C/HE CGN.tif]

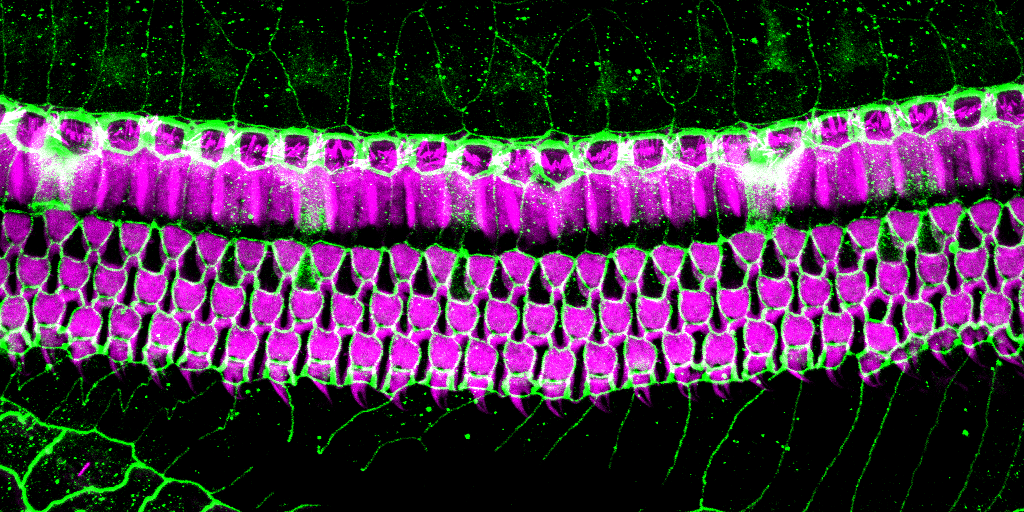

Supplement: Supplementary file 9 — Source Data for Figure 5 [file EMMM-15-e17611-s006.zip › Figure 5/5C/HE Merge.tif]

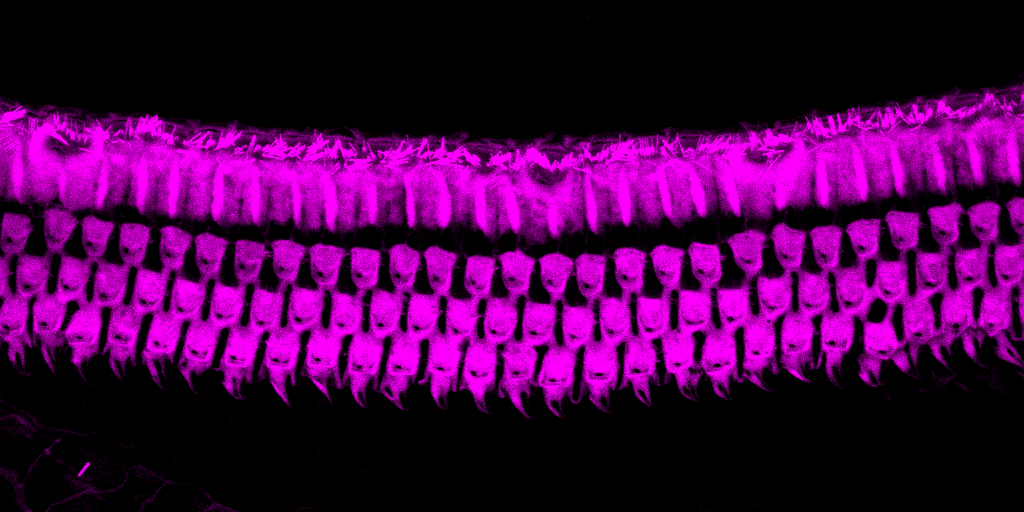

Supplement: Supplementary file 9 — Source Data for Figure 5 [file EMMM-15-e17611-s006.zip › Figure 5/5C/HE Phalloidine.tif]

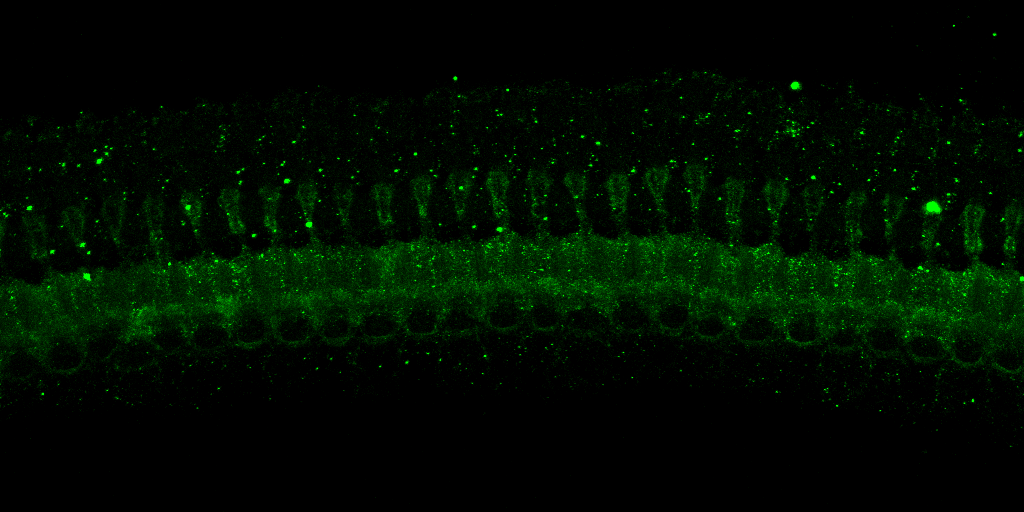

Supplement: Supplementary file 9 — Source Data for Figure 5 [file EMMM-15-e17611-s006.zip › Figure 5/5C/HO CGN.tif]

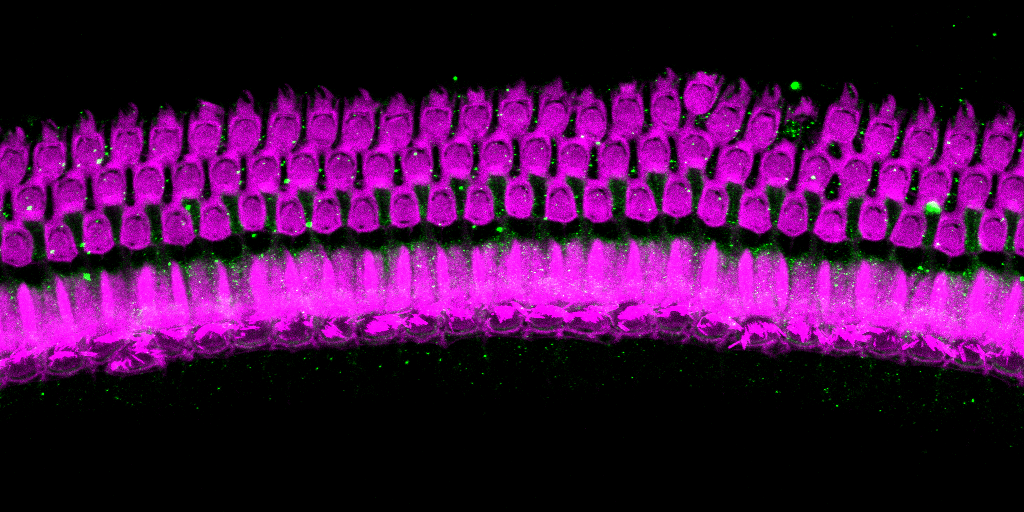

Supplement: Supplementary file 9 — Source Data for Figure 5 [file EMMM-15-e17611-s006.zip › Figure 5/5C/HO Merge.tif]

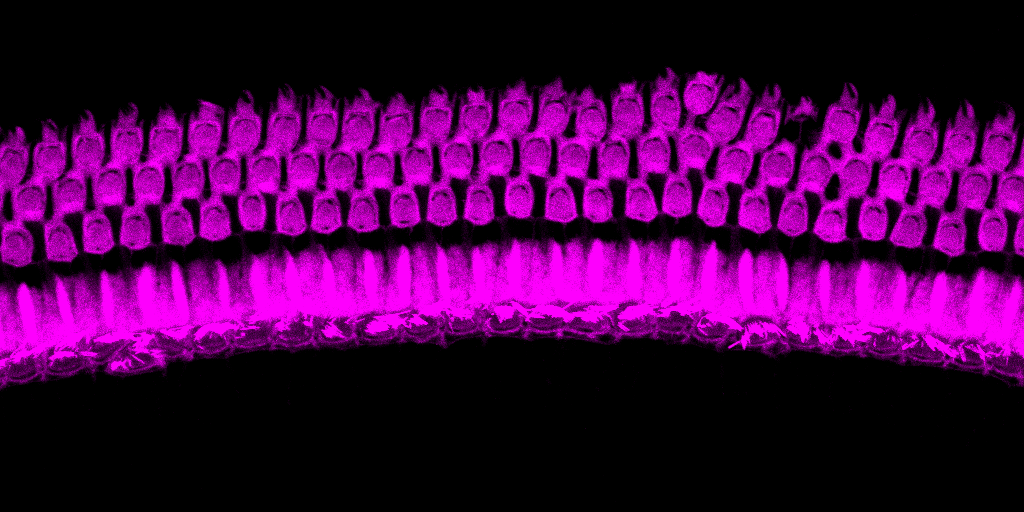

Supplement: Supplementary file 9 — Source Data for Figure 5 [file EMMM-15-e17611-s006.zip › Figure 5/5C/HO Phalloidine.tif]

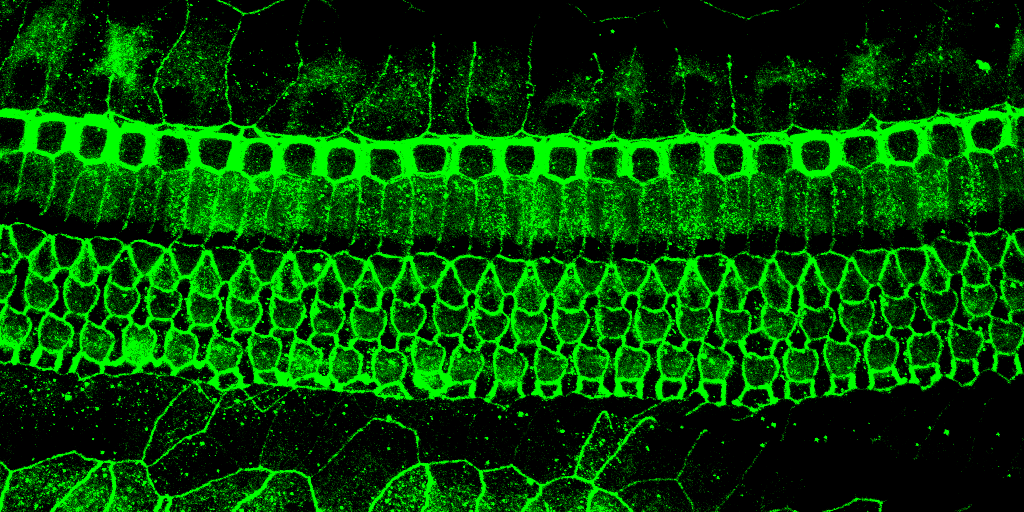

Supplement: Supplementary file 9 — Source Data for Figure 5 [file EMMM-15-e17611-s006.zip › Figure 5/5C/WT CGN.tif]

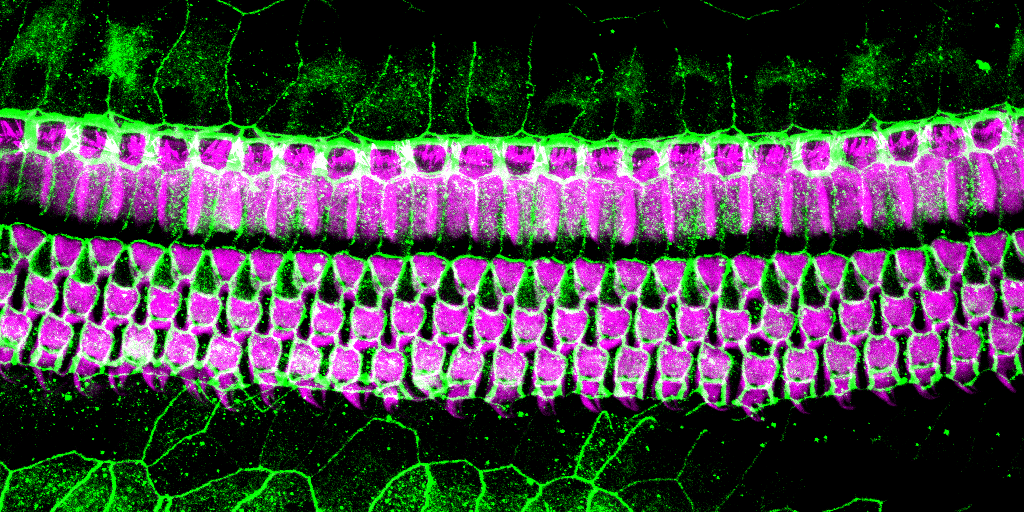

Supplement: Supplementary file 9 — Source Data for Figure 5 [file EMMM-15-e17611-s006.zip › Figure 5/5C/WT Merge.tif]

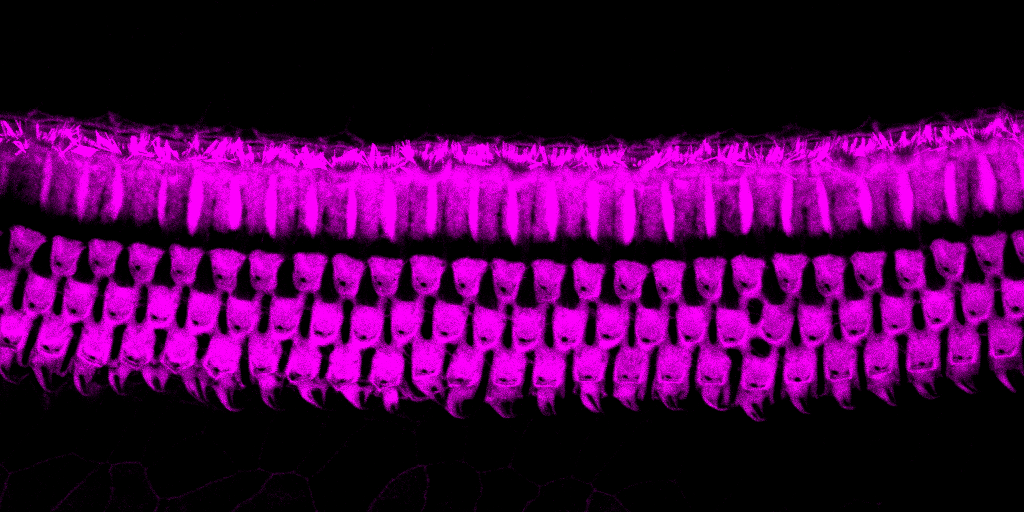

Supplement: Supplementary file 9 — Source Data for Figure 5 [file EMMM-15-e17611-s006.zip › Figure 5/5C/WT phalloidine.tif]

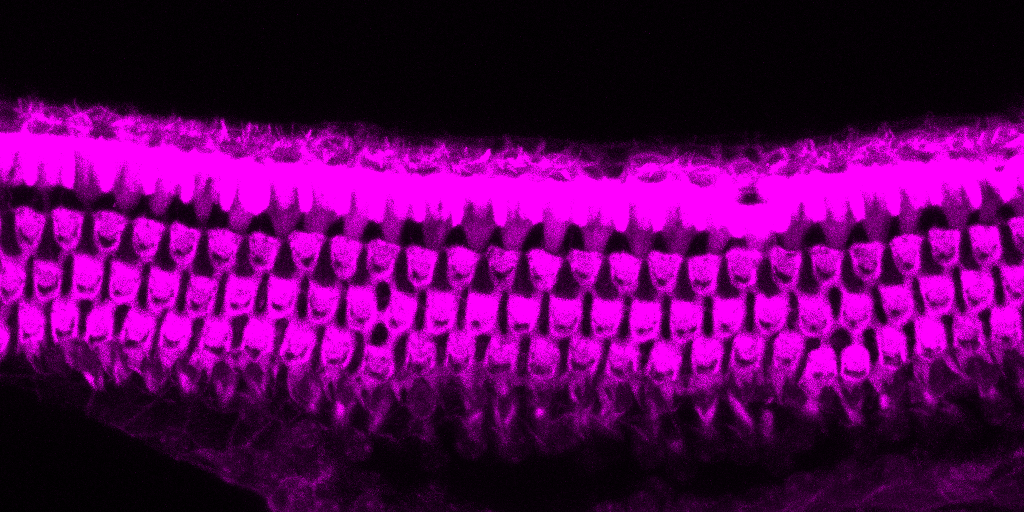

Supplement: Supplementary file 9 — Source Data for Figure 5 [file EMMM-15-e17611-s006.zip › Figure 5/5J/HE phalloidin 16kHz.tif]

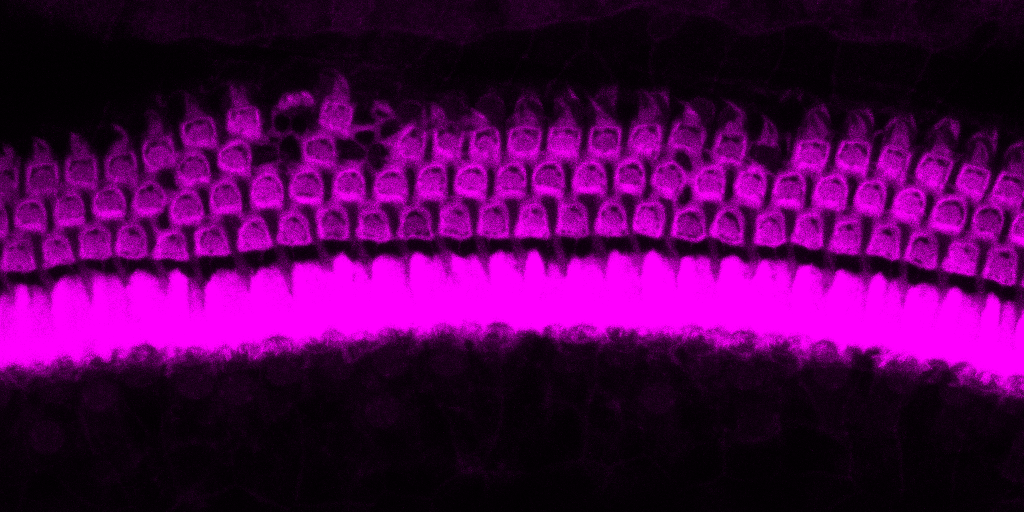

Supplement: Supplementary file 9 — Source Data for Figure 5 [file EMMM-15-e17611-s006.zip › Figure 5/5J/HE phalloidin 32kHz.tif]

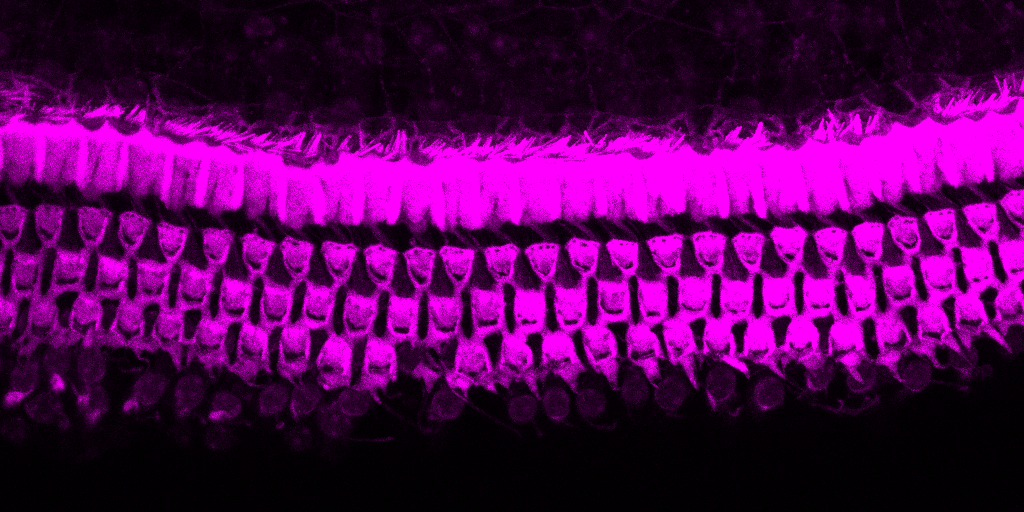

Supplement: Supplementary file 9 — Source Data for Figure 5 [file EMMM-15-e17611-s006.zip › Figure 5/5J/HE phalloidin 8kHz.tif]

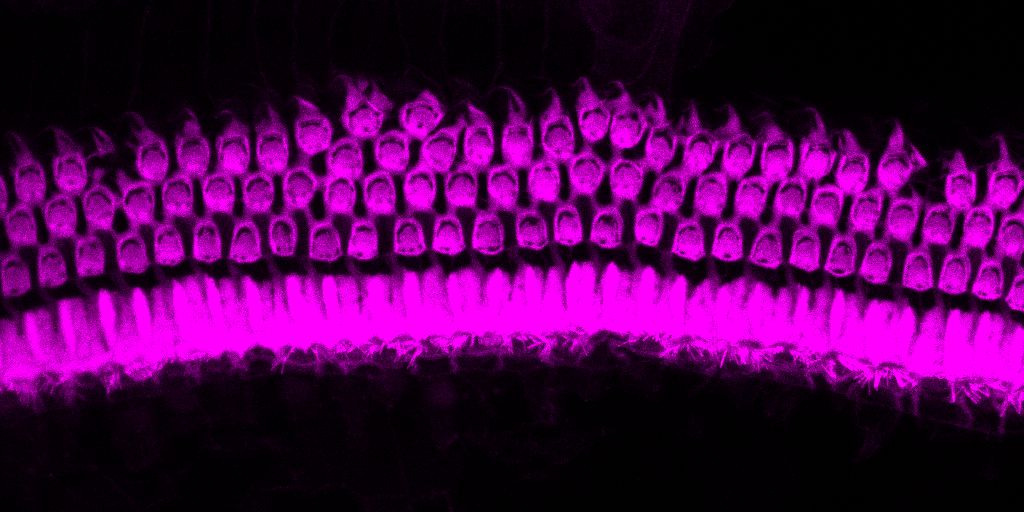

Supplement: Supplementary file 9 — Source Data for Figure 5 [file EMMM-15-e17611-s006.zip › Figure 5/5J/HO phalloidin 16kHz.tif]

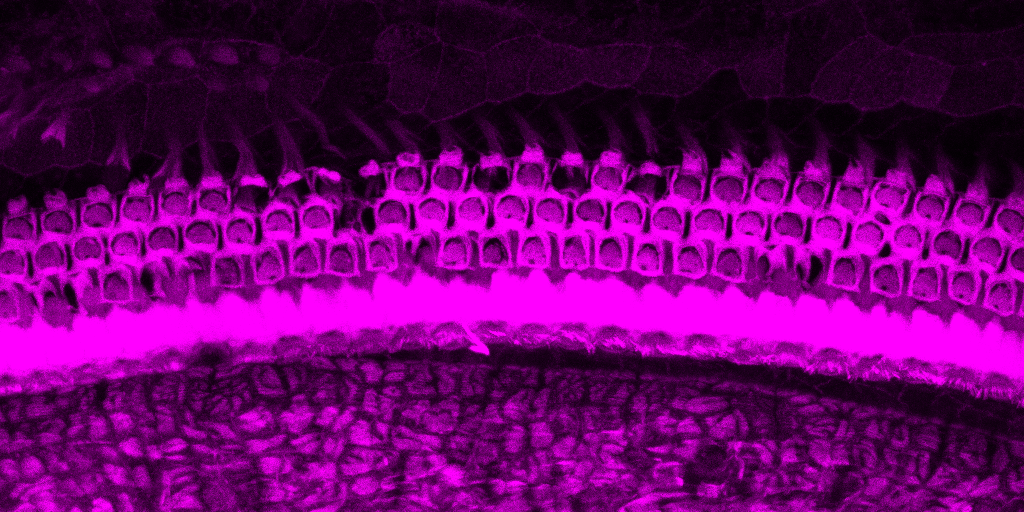

Supplement: Supplementary file 9 — Source Data for Figure 5 [file EMMM-15-e17611-s006.zip › Figure 5/5J/HO phalloidin 32kHz.tif]

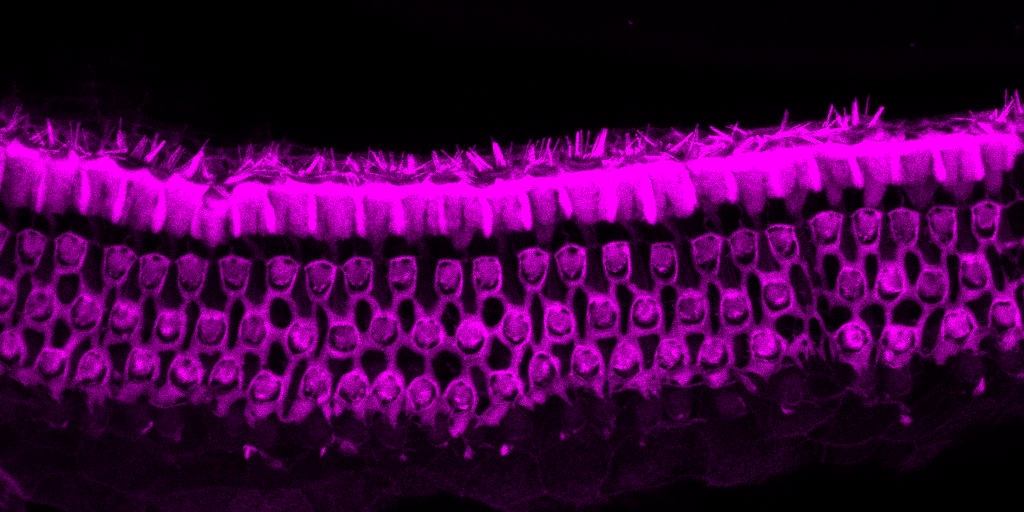

Supplement: Supplementary file 9 — Source Data for Figure 5 [file EMMM-15-e17611-s006.zip › Figure 5/5J/HO phalloidin 8kHz.tif]

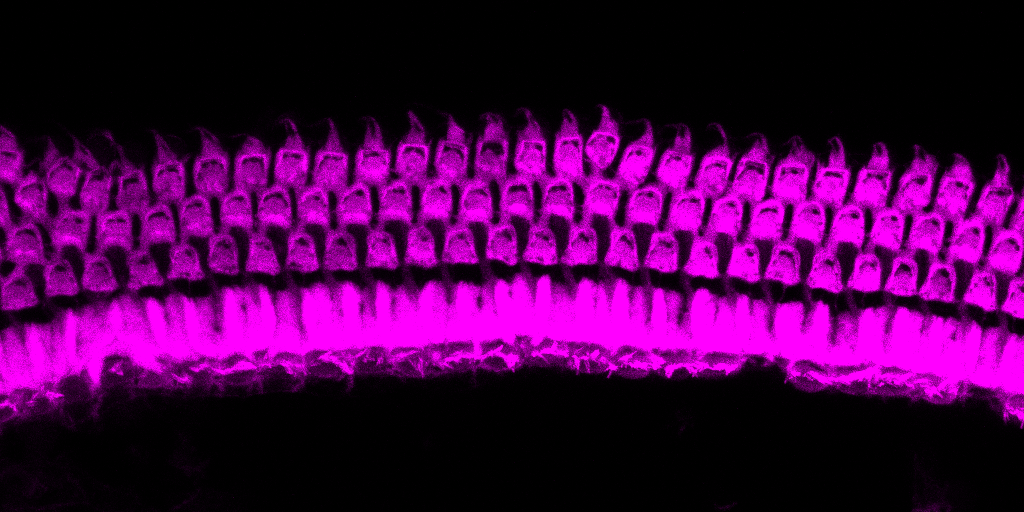

Supplement: Supplementary file 9 — Source Data for Figure 5 [file EMMM-15-e17611-s006.zip › Figure 5/5J/WT phalloidin 16kHz.tif]

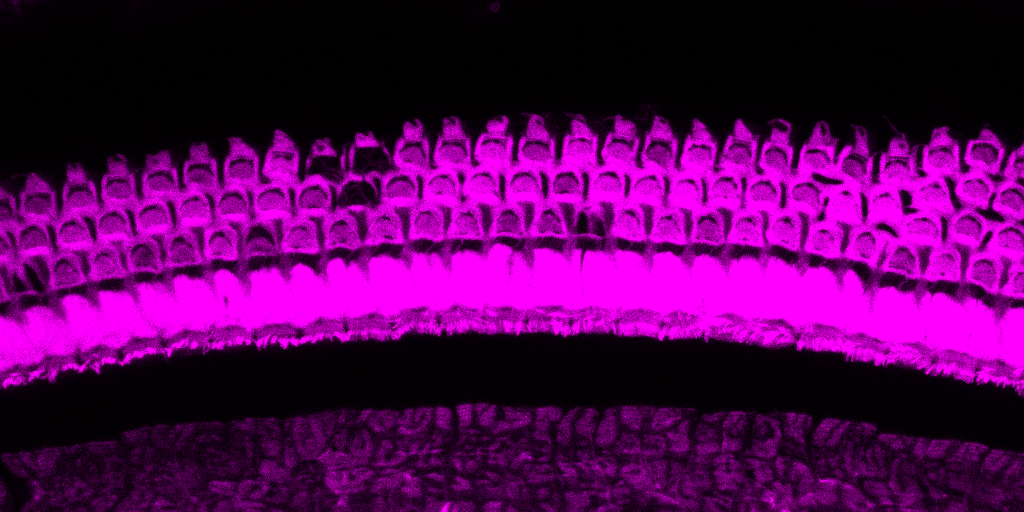

Supplement: Supplementary file 9 — Source Data for Figure 5 [file EMMM-15-e17611-s006.zip › Figure 5/5J/WT phalloidin 32kHz.tif]

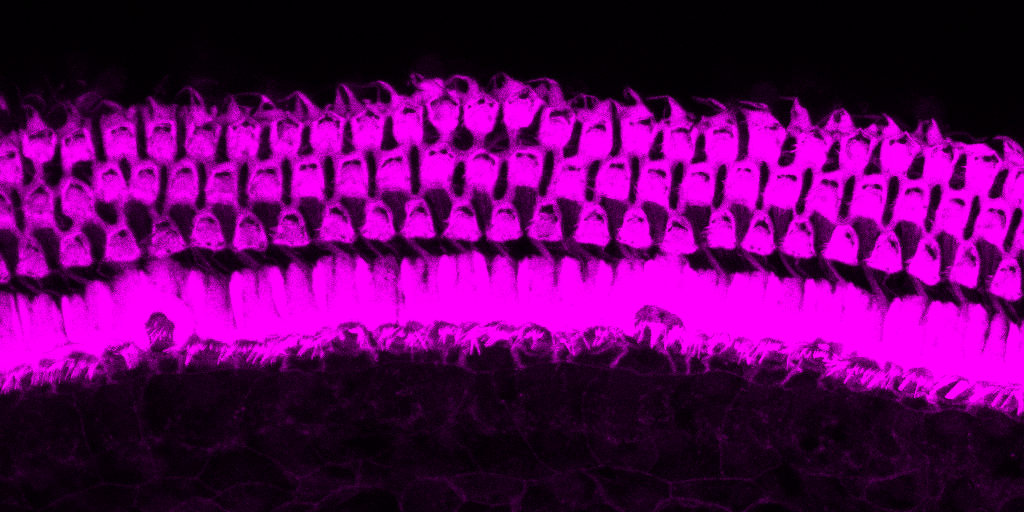

Supplement: Supplementary file 9 — Source Data for Figure 5 [file EMMM-15-e17611-s006.zip › Figure 5/5J/WT phalloidin 8kHz.tif]

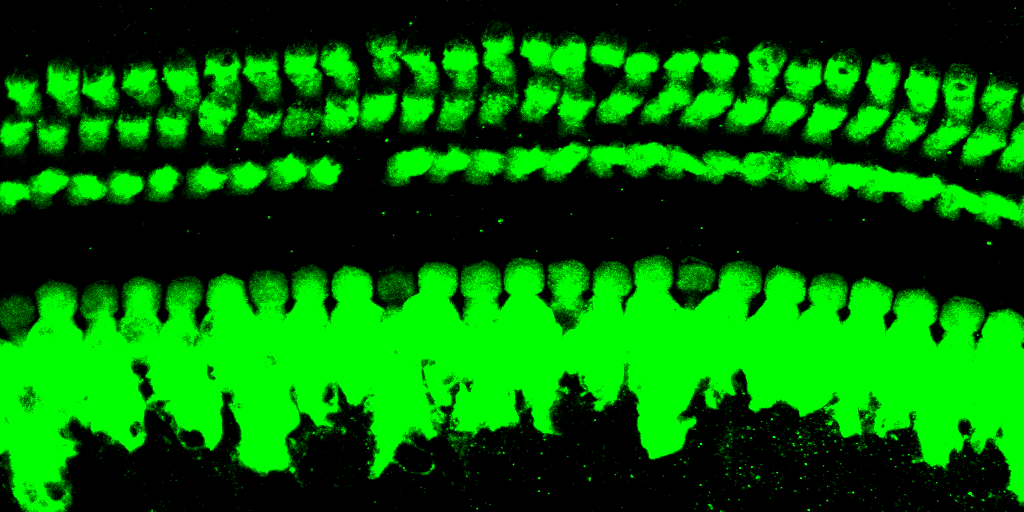

Supplement: Supplementary file 10 — Source Data for Figure 6 [file EMMM-15-e17611-s005.zip › Figure 6/6E/HE myosin7a 16kHz.tif]

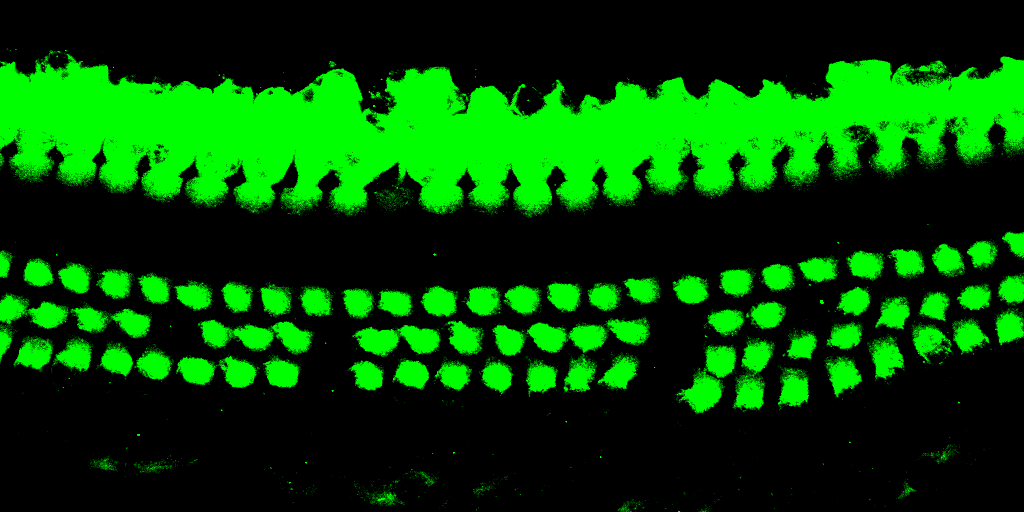

Supplement: Supplementary file 10 — Source Data for Figure 6 [file EMMM-15-e17611-s005.zip › Figure 6/6E/HE myosin7a 32kHz.tif]

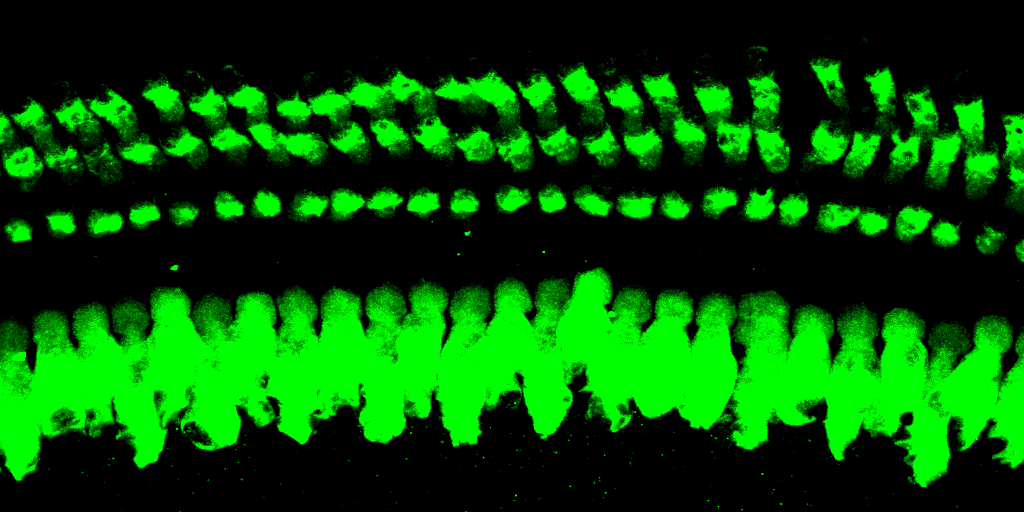

Supplement: Supplementary file 10 — Source Data for Figure 6 [file EMMM-15-e17611-s005.zip › Figure 6/6E/HE myosin7a 8kHz.tif]

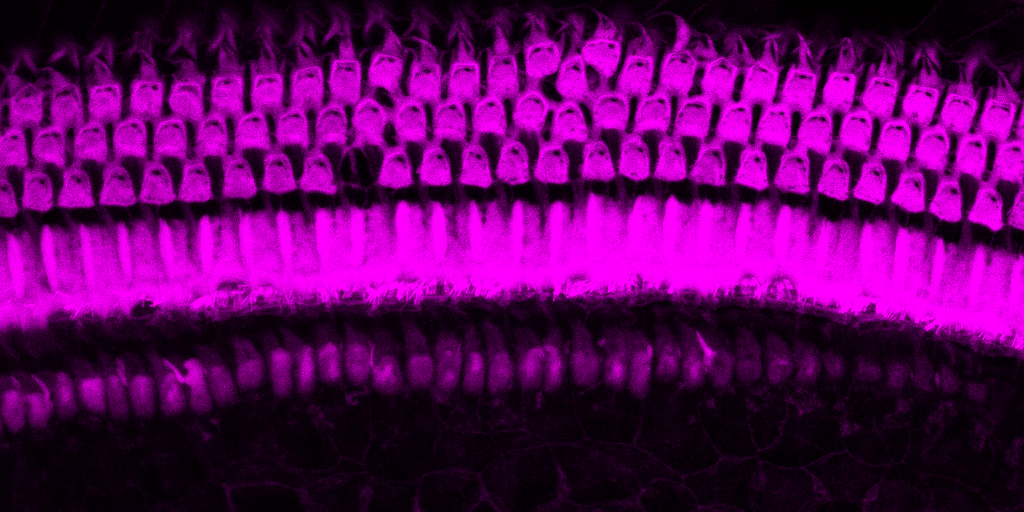

Supplement: Supplementary file 10 — Source Data for Figure 6 [file EMMM-15-e17611-s005.zip › Figure 6/6E/HE phalloidin 16kHz.tif]

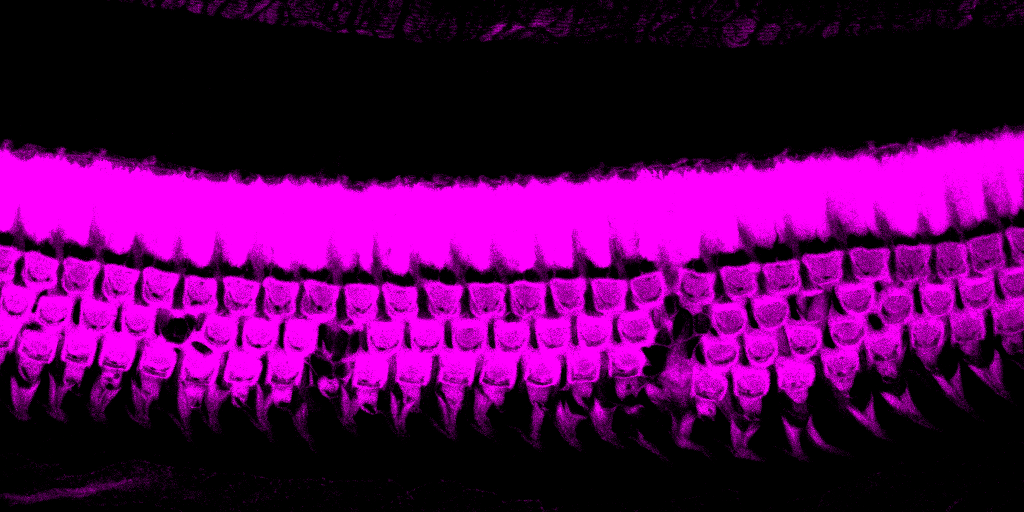

Supplement: Supplementary file 10 — Source Data for Figure 6 [file EMMM-15-e17611-s005.zip › Figure 6/6E/HE phalloidin 32kHz.tif]

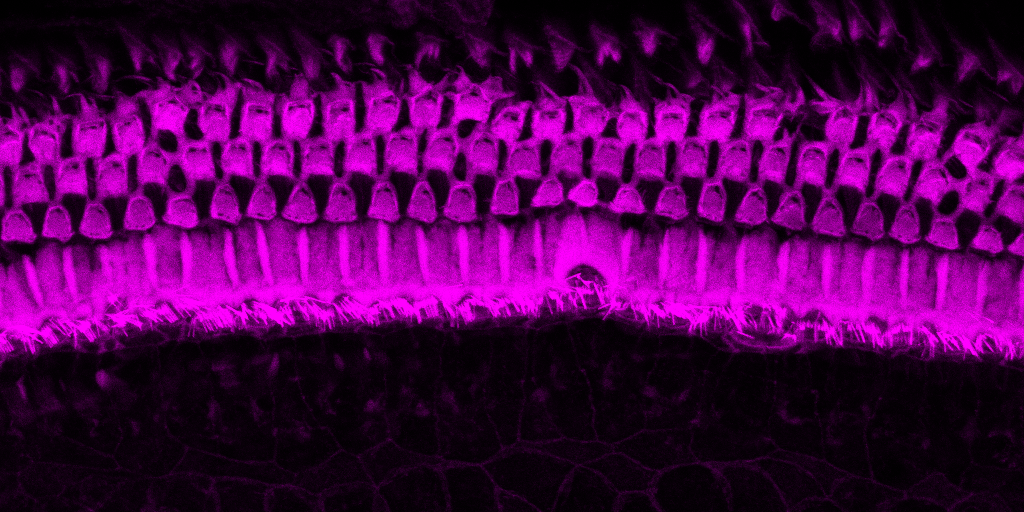

Supplement: Supplementary file 10 — Source Data for Figure 6 [file EMMM-15-e17611-s005.zip › Figure 6/6E/HE phalloidin 8kHz.tif]

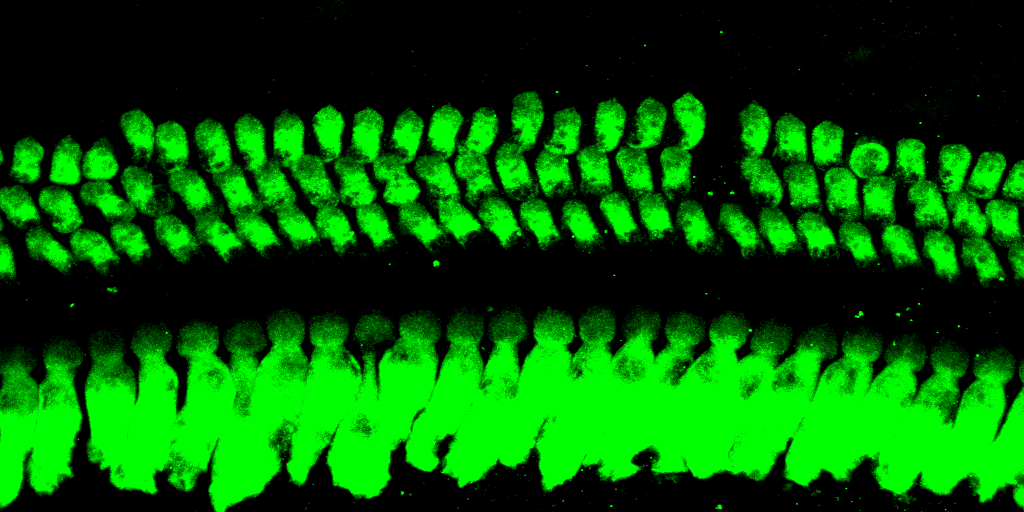

Supplement: Supplementary file 10 — Source Data for Figure 6 [file EMMM-15-e17611-s005.zip › Figure 6/6E/HO myosin7a 16kHz.tif]

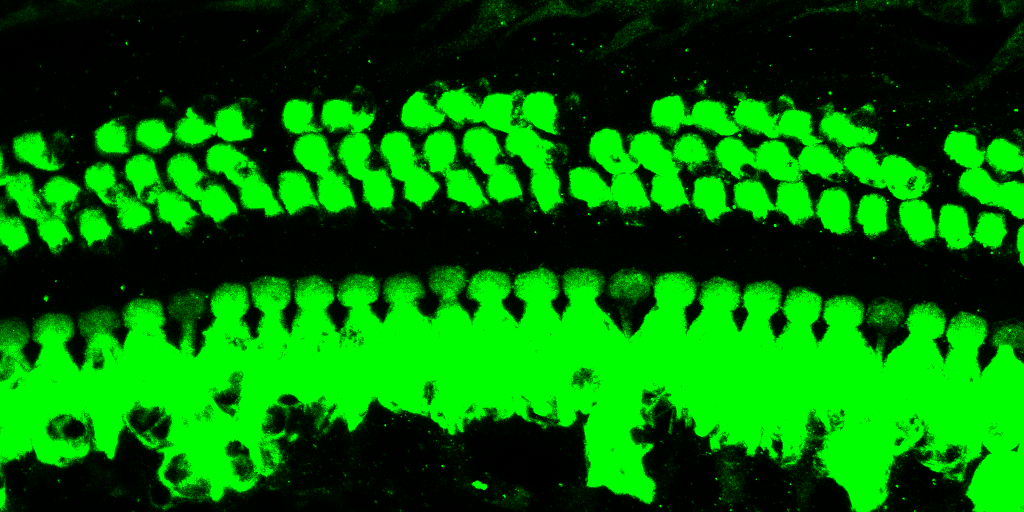

Supplement: Supplementary file 10 — Source Data for Figure 6 [file EMMM-15-e17611-s005.zip › Figure 6/6E/HO myosin7a 32kHz.tif]

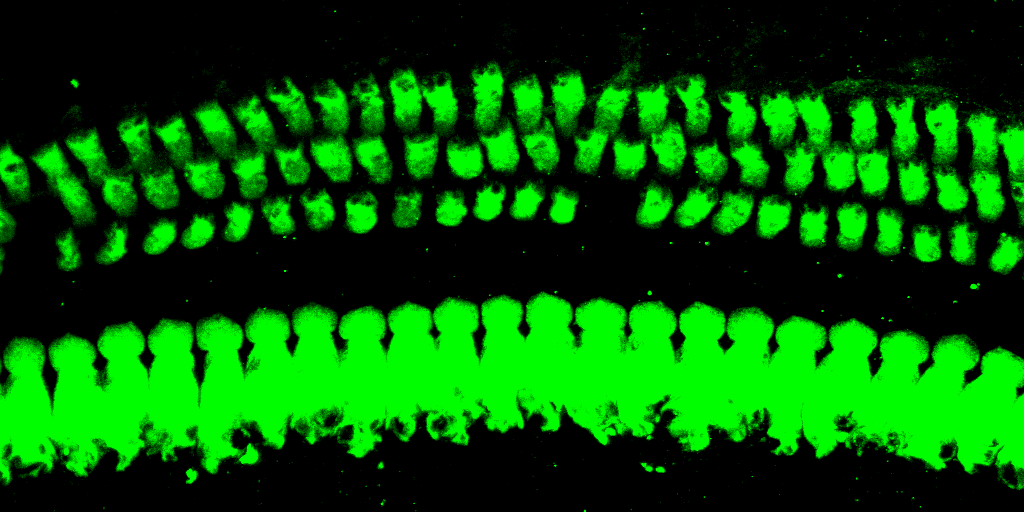

Supplement: Supplementary file 10 — Source Data for Figure 6 [file EMMM-15-e17611-s005.zip › Figure 6/6E/HO myosin7a 8kHz.tif]

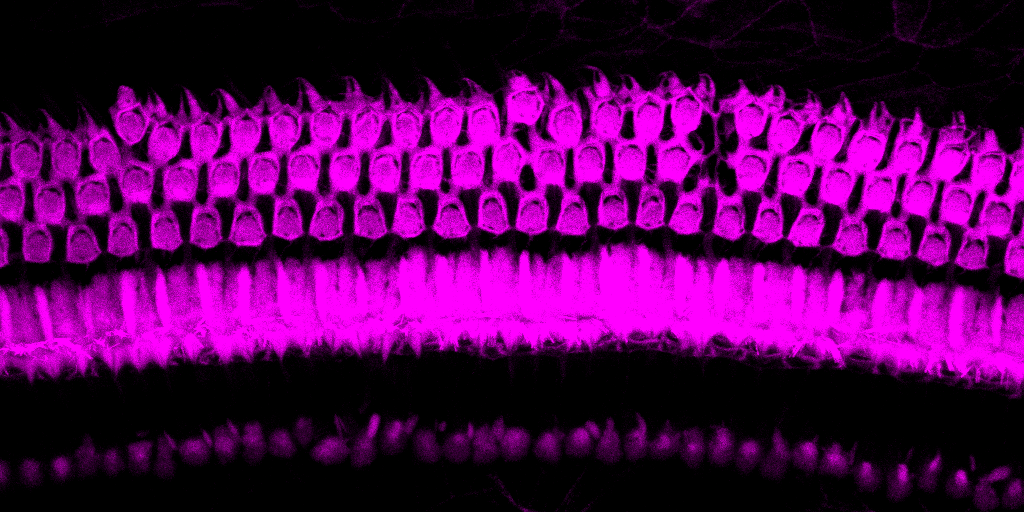

Supplement: Supplementary file 10 — Source Data for Figure 6 [file EMMM-15-e17611-s005.zip › Figure 6/6E/HO phalloidin 16kHz.tif]

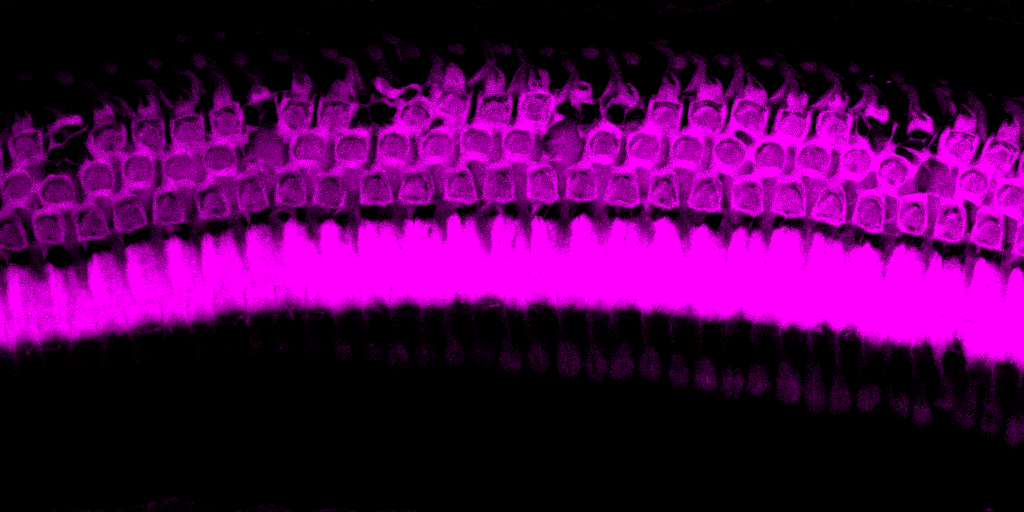

Supplement: Supplementary file 10 — Source Data for Figure 6 [file EMMM-15-e17611-s005.zip › Figure 6/6E/HO phalloidin 32kHz.tif]

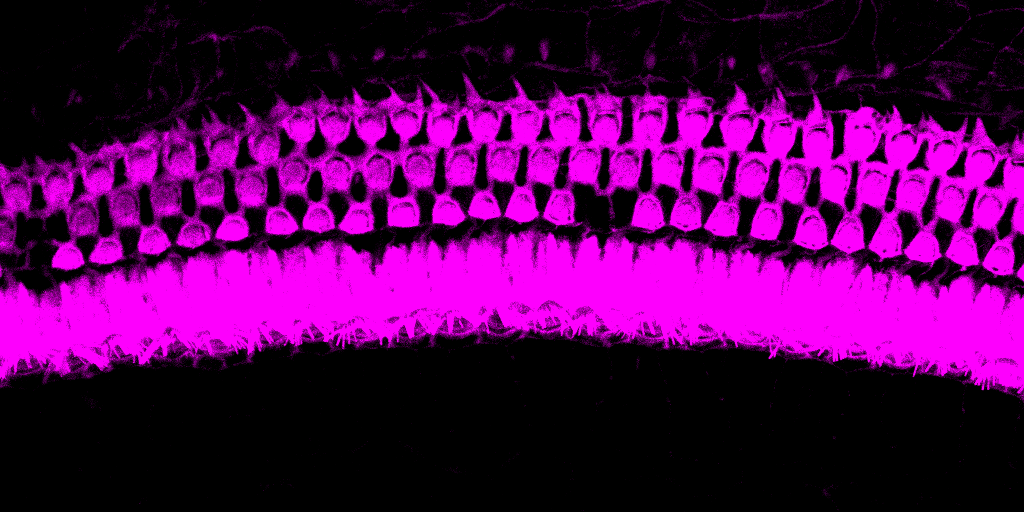

Supplement: Supplementary file 10 — Source Data for Figure 6 [file EMMM-15-e17611-s005.zip › Figure 6/6E/HO phalloidin 8kHz.tif]

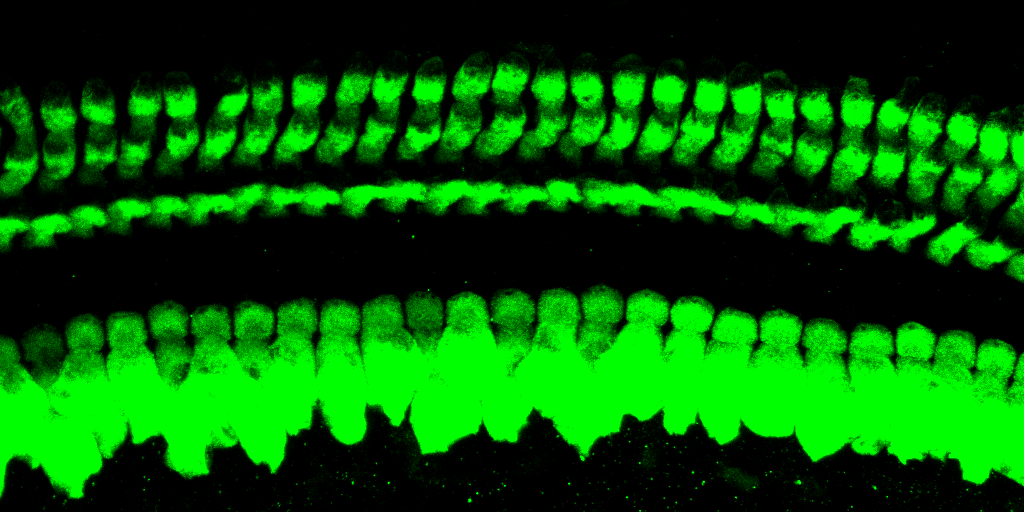

Supplement: Supplementary file 10 — Source Data for Figure 6 [file EMMM-15-e17611-s005.zip › Figure 6/6E/WT myosin7a 16kHz.tif]

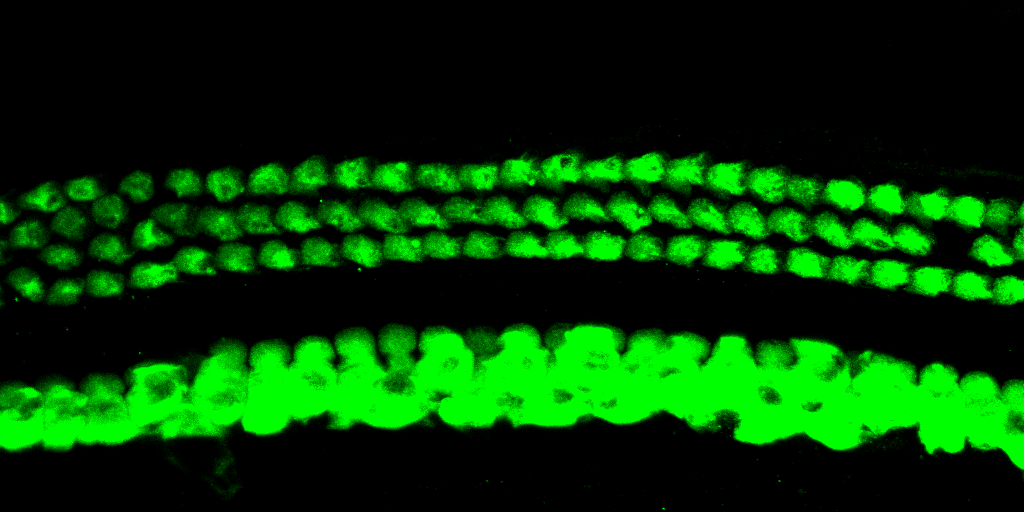

Supplement: Supplementary file 10 — Source Data for Figure 6 [file EMMM-15-e17611-s005.zip › Figure 6/6E/WT myosin7a 32kHz.tif]

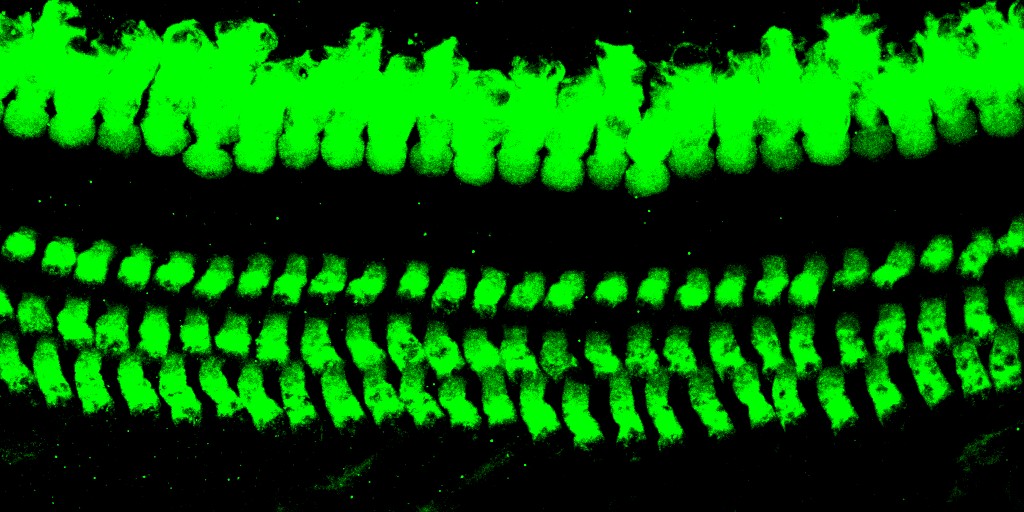

Supplement: Supplementary file 10 — Source Data for Figure 6 [file EMMM-15-e17611-s005.zip › Figure 6/6E/WT myosin7a 8kHz.tif]

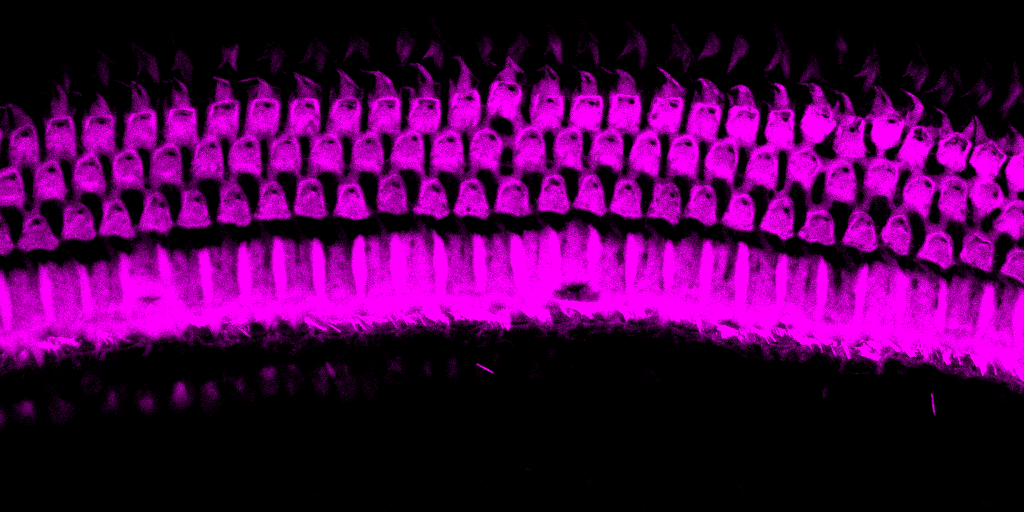

Supplement: Supplementary file 10 — Source Data for Figure 6 [file EMMM-15-e17611-s005.zip › Figure 6/6E/WT phalloidin 16kHz.tif]

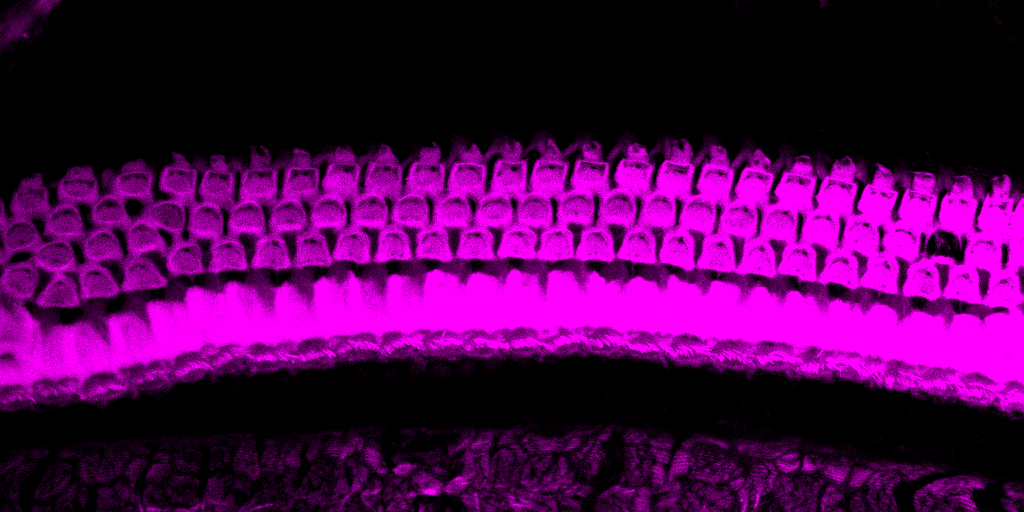

Supplement: Supplementary file 10 — Source Data for Figure 6 [file EMMM-15-e17611-s005.zip › Figure 6/6E/WT phalloidin 32kHz.tif]

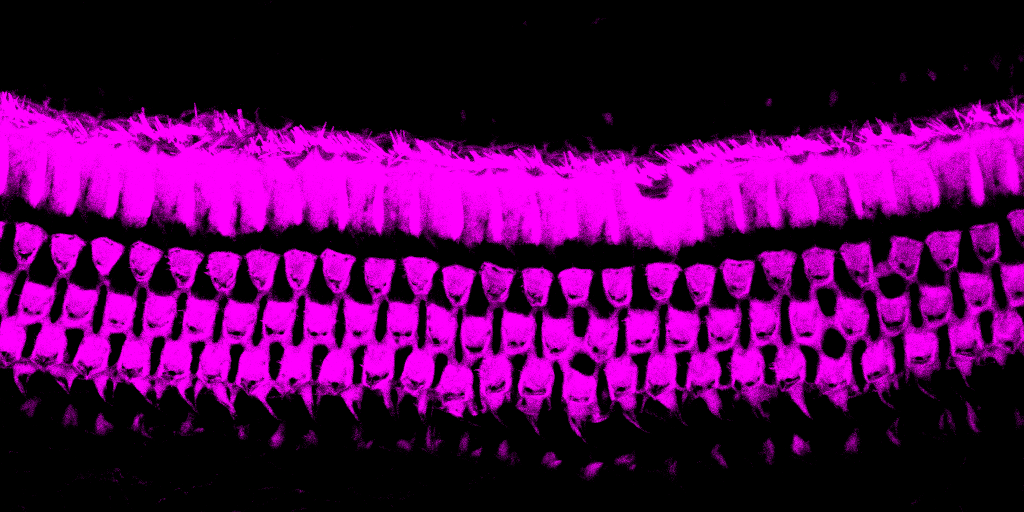

Supplement: Supplementary file 10 — Source Data for Figure 6 [file EMMM-15-e17611-s005.zip › Figure 6/6E/WT phalloidin 8kHz.tif]

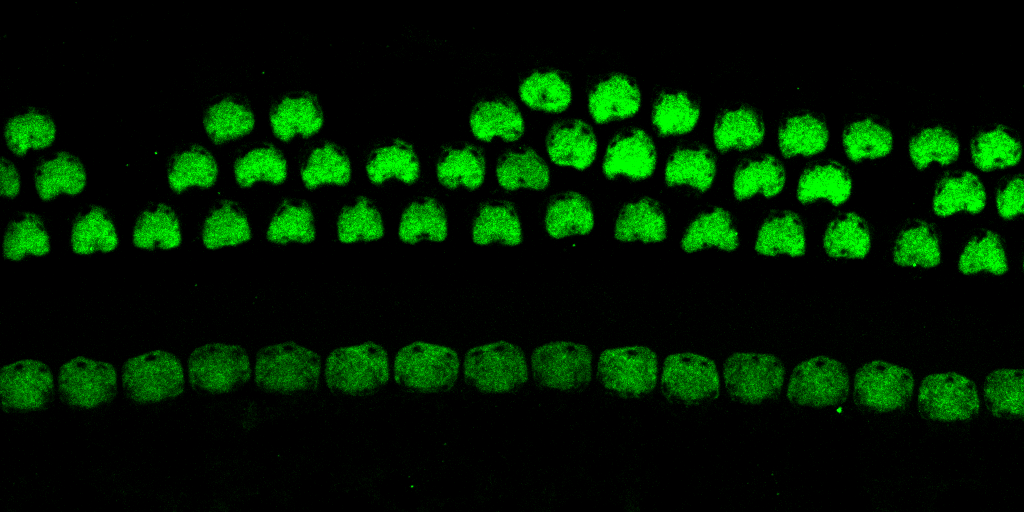

Supplement: Supplementary file 11 — Source Data for Figure 7 [file EMMM-15-e17611-s012.zip › Figure 7/7E/HO LMO7.tif]

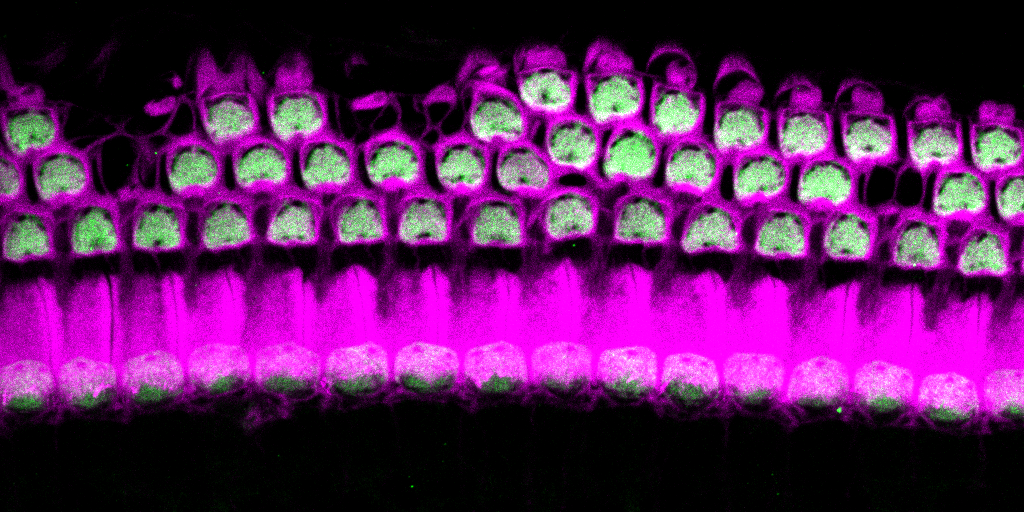

Supplement: Supplementary file 11 — Source Data for Figure 7 [file EMMM-15-e17611-s012.zip › Figure 7/7E/HO Merge.tif]

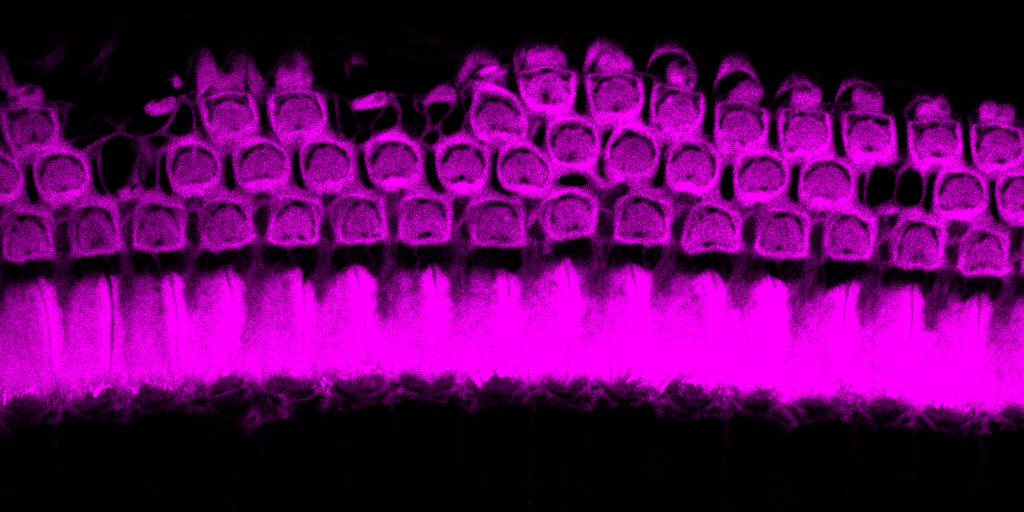

Supplement: Supplementary file 11 — Source Data for Figure 7 [file EMMM-15-e17611-s012.zip › Figure 7/7E/HO Phalloidine.tif]

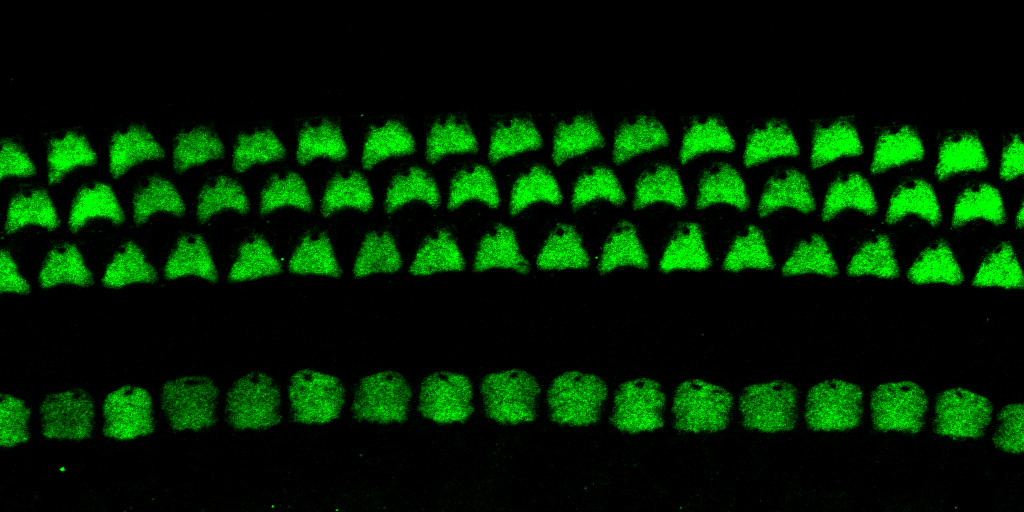

Supplement: Supplementary file 11 — Source Data for Figure 7 [file EMMM-15-e17611-s012.zip › Figure 7/7E/WT LMO7.tif]

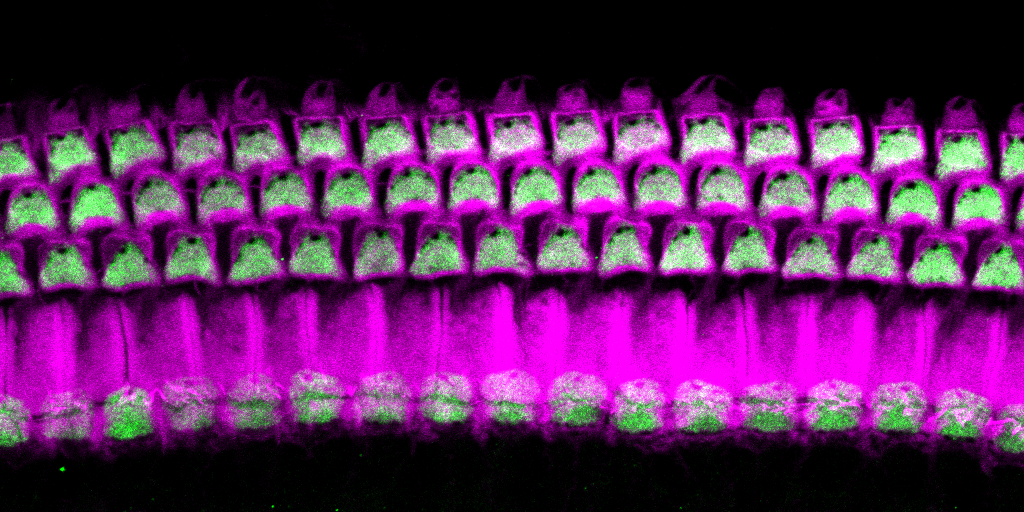

Supplement: Supplementary file 11 — Source Data for Figure 7 [file EMMM-15-e17611-s012.zip › Figure 7/7E/WT Merge.tif]

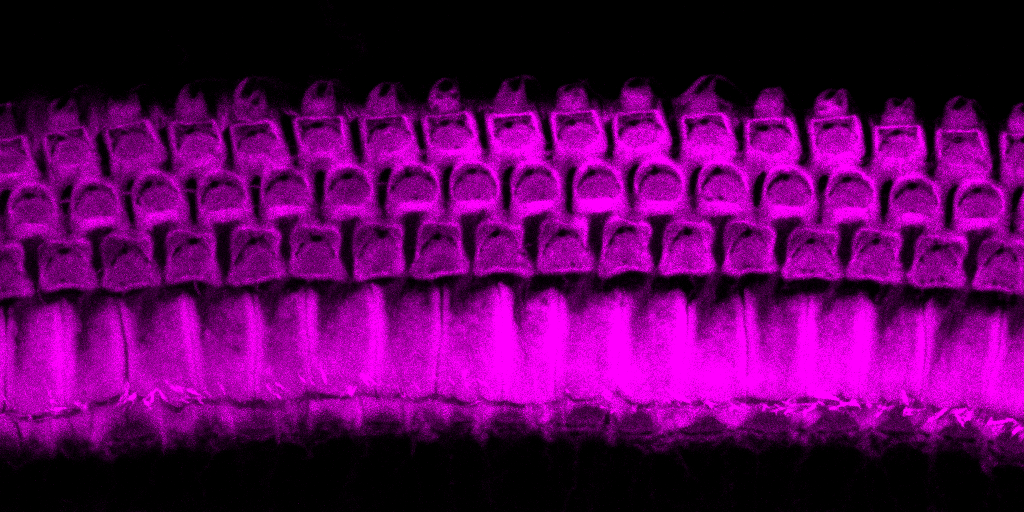

Supplement: Supplementary file 11 — Source Data for Figure 7 [file EMMM-15-e17611-s012.zip › Figure 7/7E/WT Phalloidine.tif]

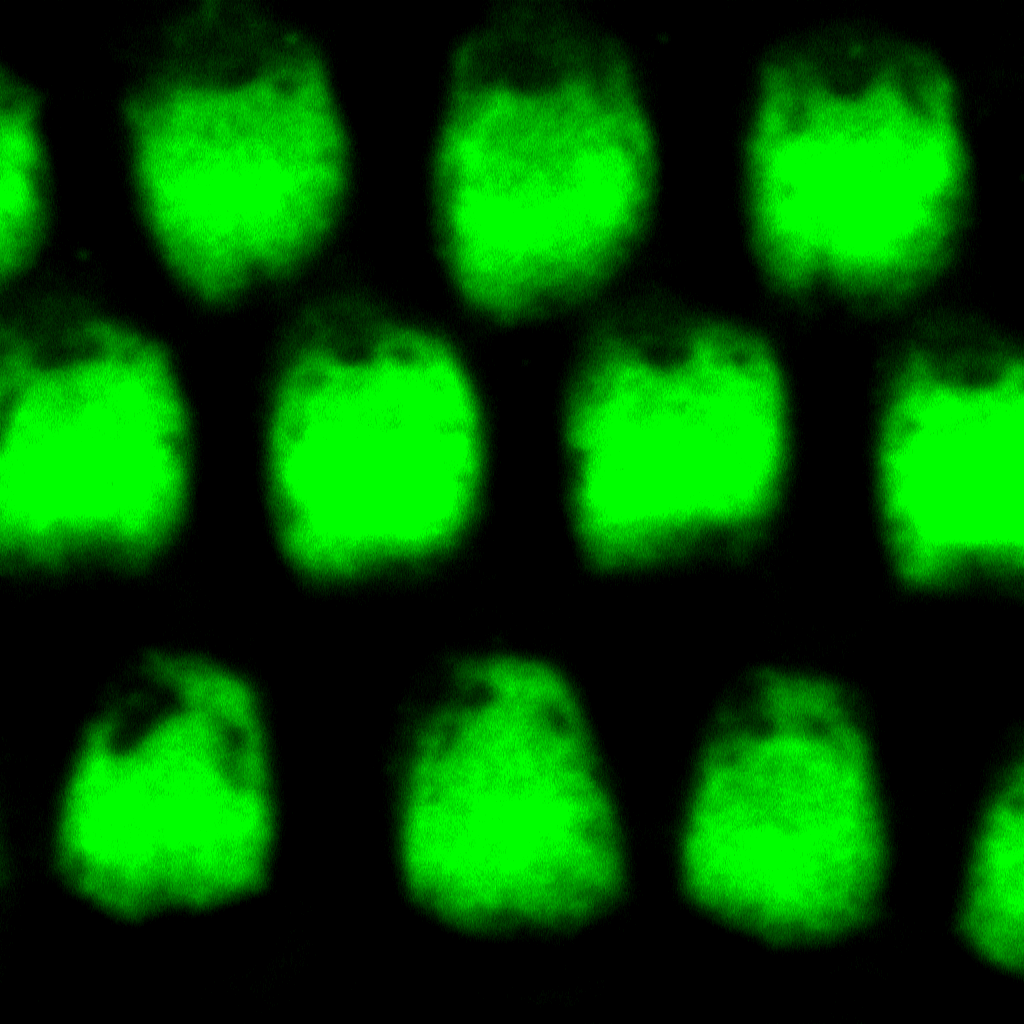

Supplement: Supplementary file 11 — Source Data for Figure 7 [file EMMM-15-e17611-s012.zip › Figure 7/7F/HO LMO7.tif]

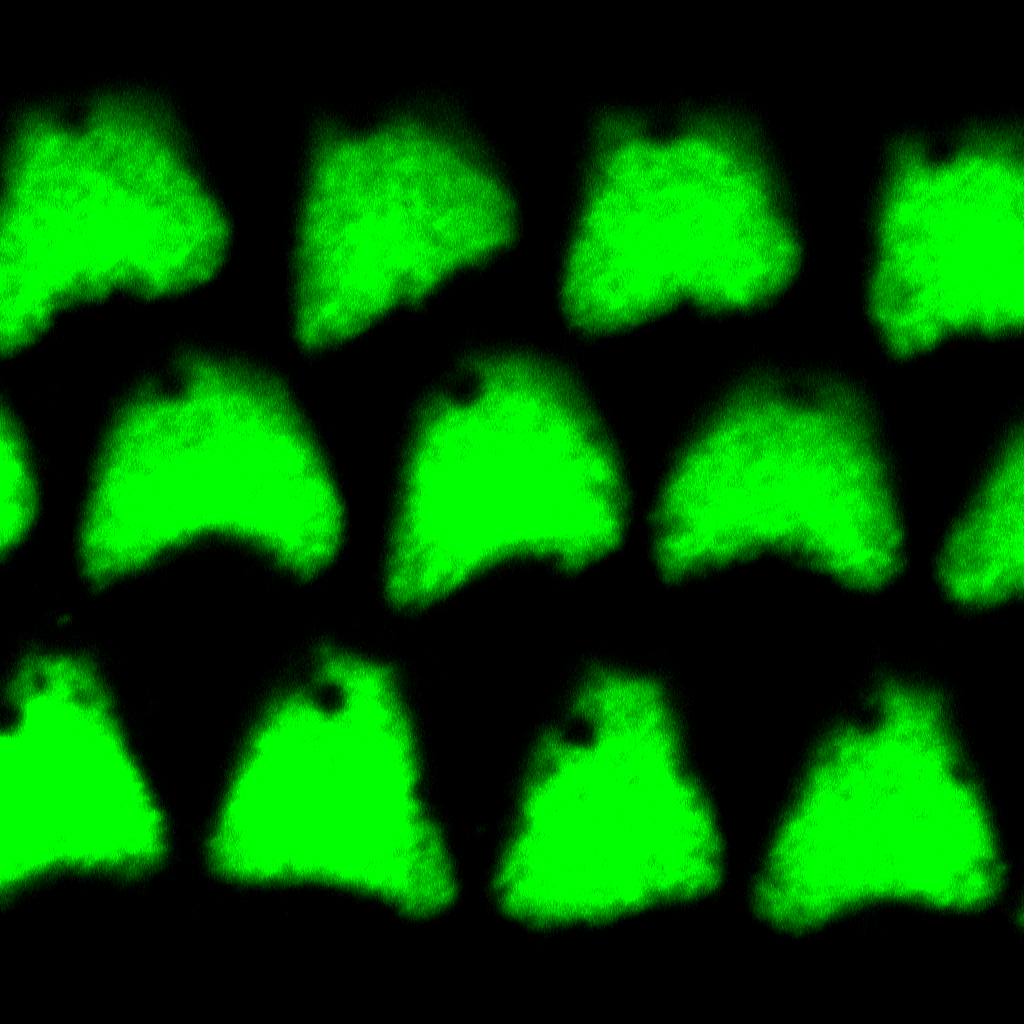

Supplement: Supplementary file 11 — Source Data for Figure 7 [file EMMM-15-e17611-s012.zip › Figure 7/7F/WT LMO7.tif]

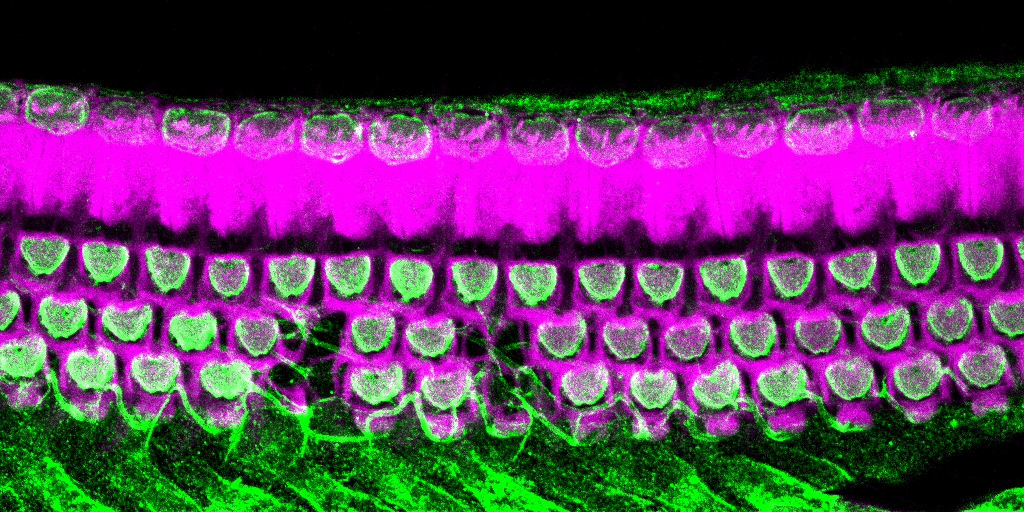

Supplement: Supplementary file 11 — Source Data for Figure 7 [file EMMM-15-e17611-s012.zip › Figure 7/7J/HO Merge.tif]

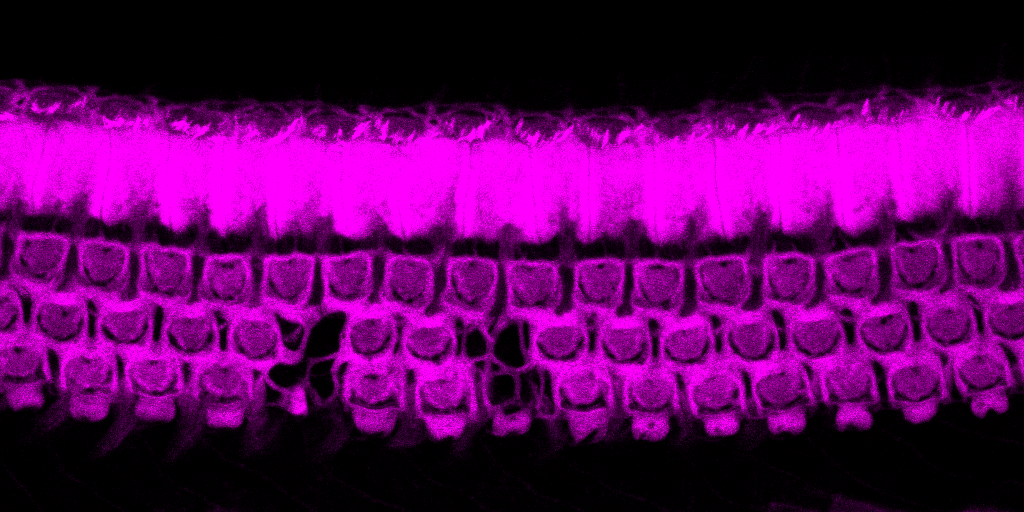

Supplement: Supplementary file 11 — Source Data for Figure 7 [file EMMM-15-e17611-s012.zip › Figure 7/7J/HO Phalloidine.tif]

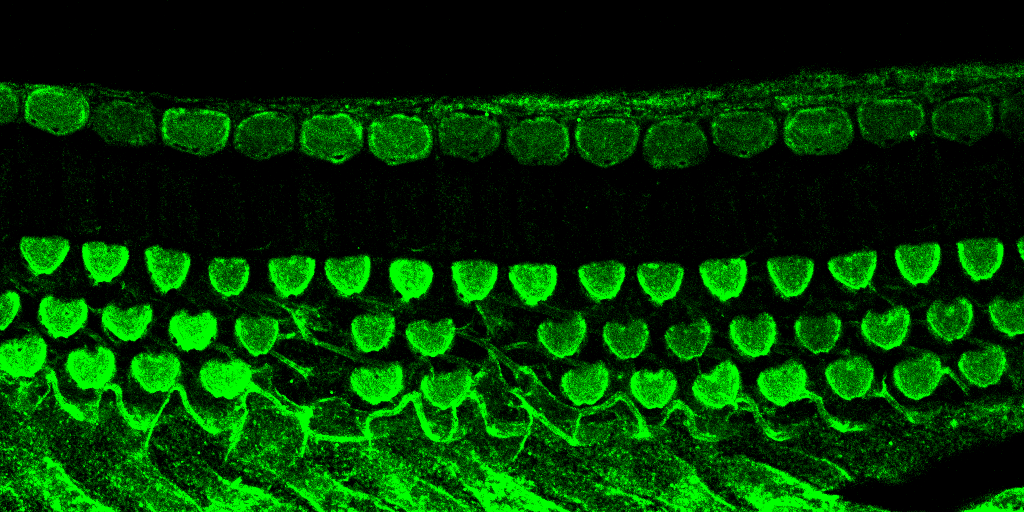

Supplement: Supplementary file 11 — Source Data for Figure 7 [file EMMM-15-e17611-s012.zip › Figure 7/7J/HO Spectrin.tif]

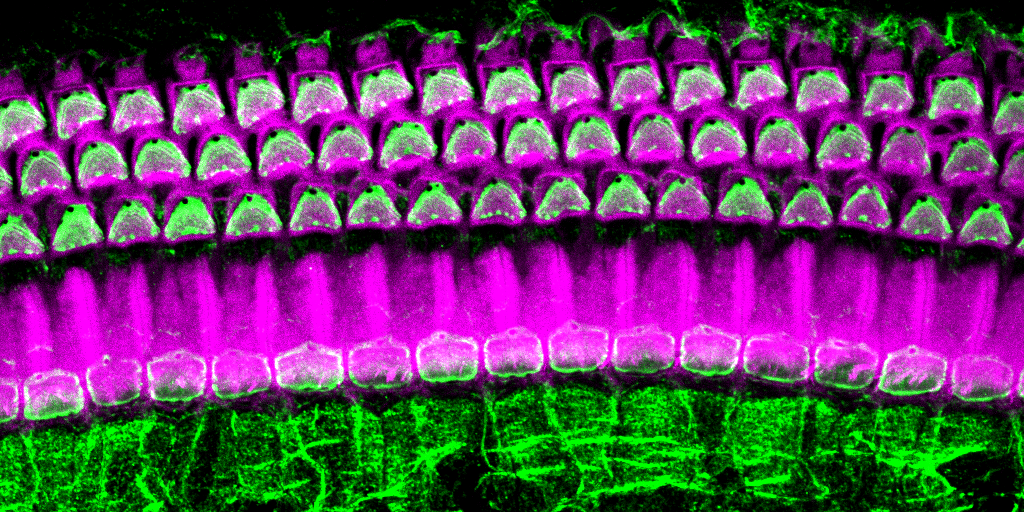

Supplement: Supplementary file 11 — Source Data for Figure 7 [file EMMM-15-e17611-s012.zip › Figure 7/7J/WT Merge.tif]

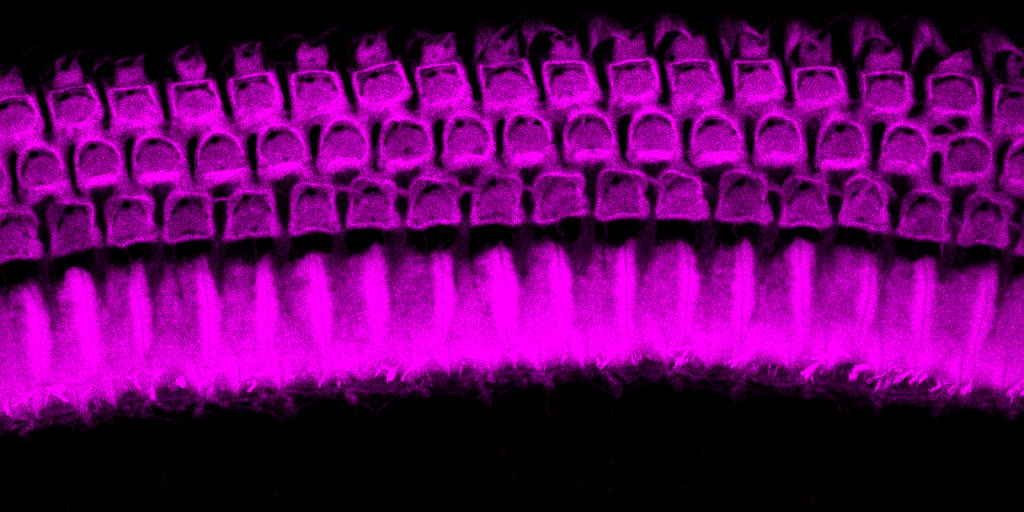

Supplement: Supplementary file 11 — Source Data for Figure 7 [file EMMM-15-e17611-s012.zip › Figure 7/7J/WT Phalloidine.tif]

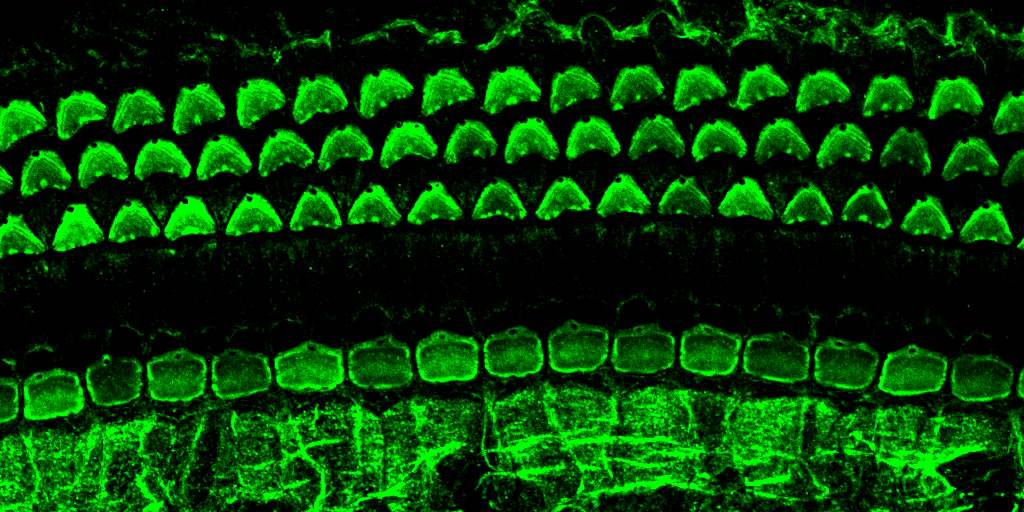

Supplement: Supplementary file 11 — Source Data for Figure 7 [file EMMM-15-e17611-s012.zip › Figure 7/7J/WT Spectrin.tif]

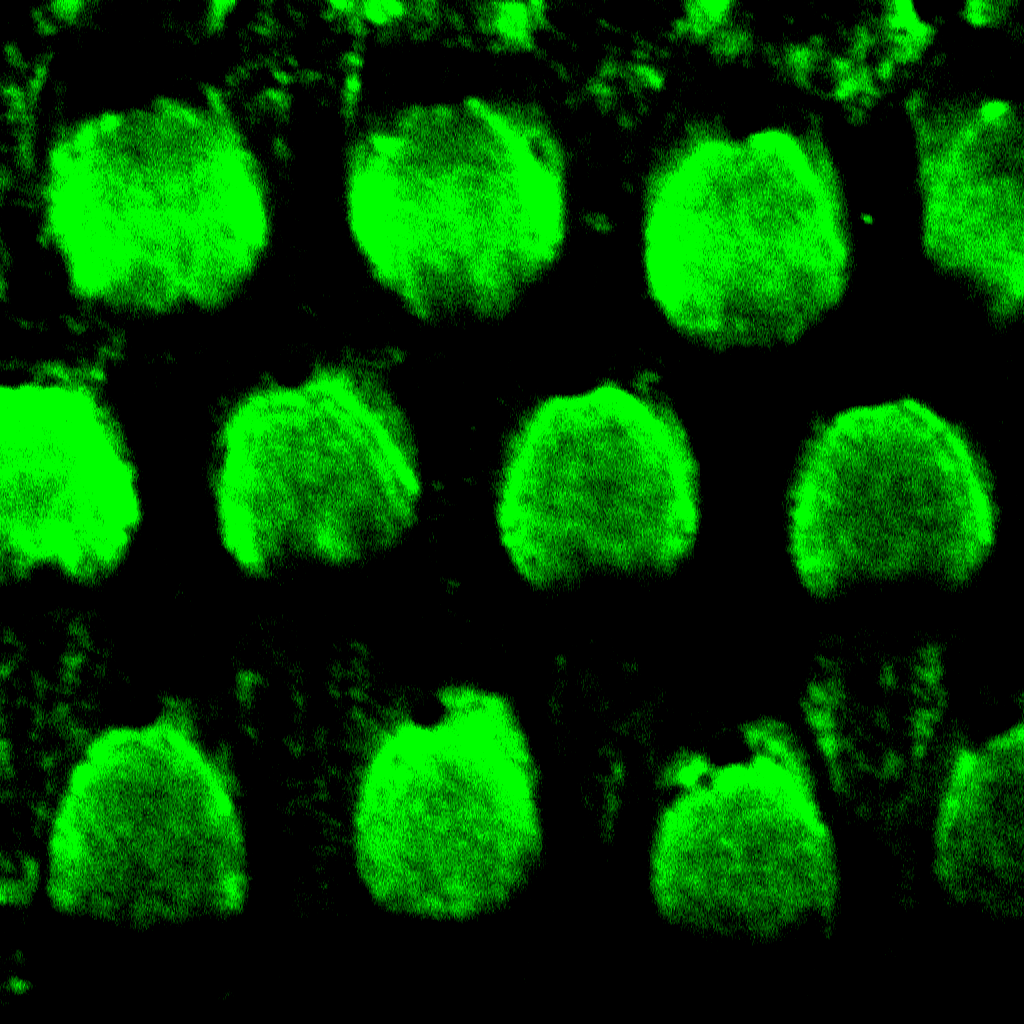

Supplement: Supplementary file 11 — Source Data for Figure 7 [file EMMM-15-e17611-s012.zip › Figure 7/7K/HO Spectrin.tif]

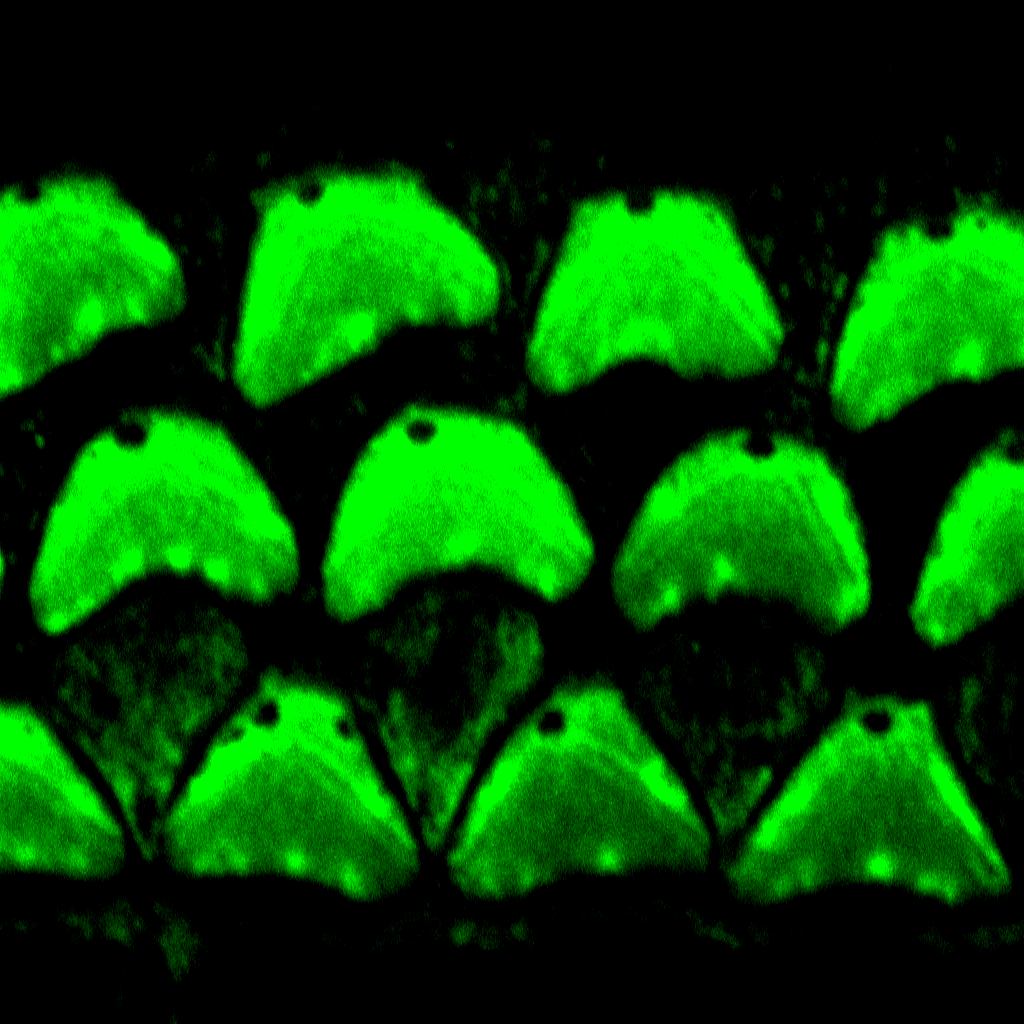

Supplement: Supplementary file 11 — Source Data for Figure 7 [file EMMM-15-e17611-s012.zip › Figure 7/7K/WT Spectrin.tif]

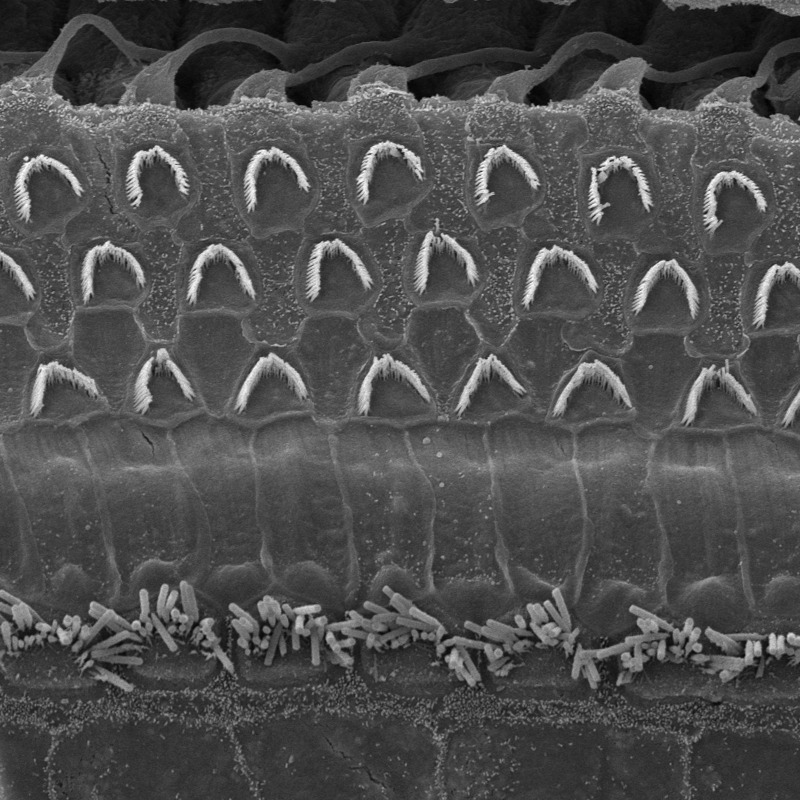

Supplement: Supplementary file 12 — Source Data for Figure 8 [file EMMM-15-e17611-s002.zip › Figure 8/8A/HO 16kHz.tif]

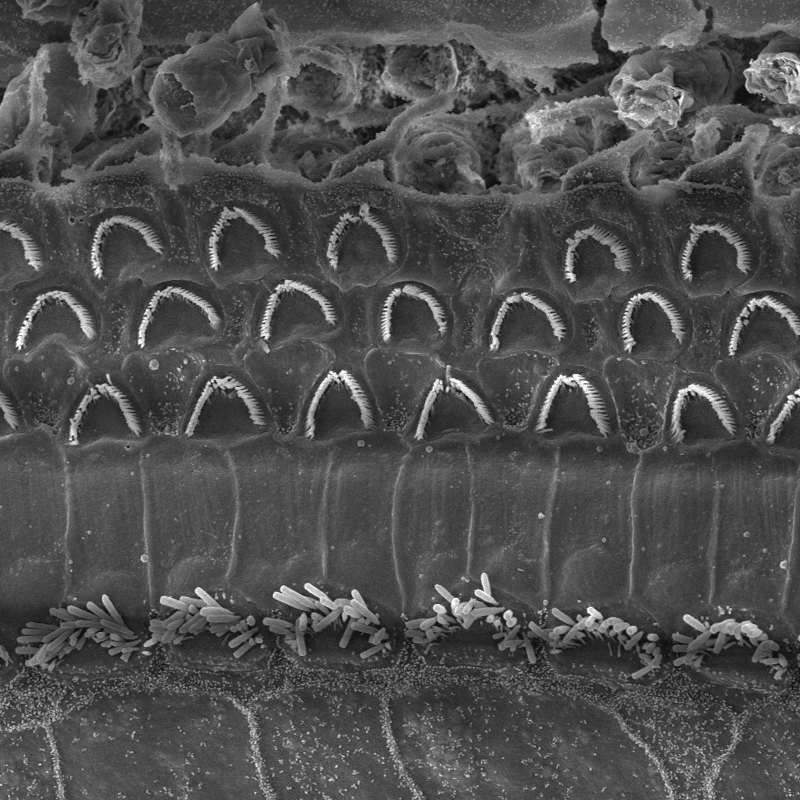

Supplement: Supplementary file 12 — Source Data for Figure 8 [file EMMM-15-e17611-s002.zip › Figure 8/8A/HO 32kHz.tif]

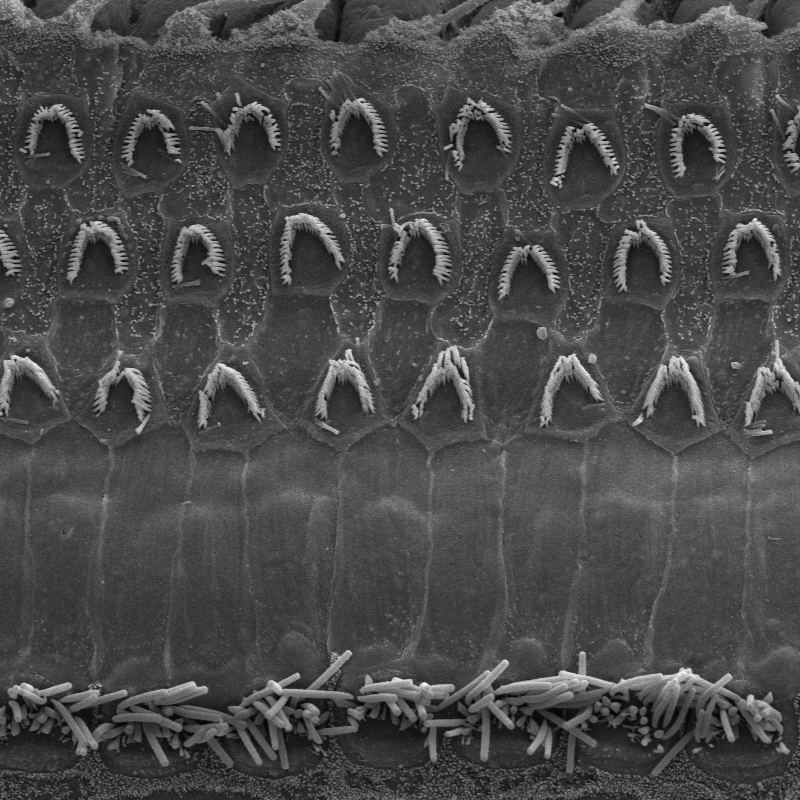

Supplement: Supplementary file 12 — Source Data for Figure 8 [file EMMM-15-e17611-s002.zip › Figure 8/8A/HO 8kHz.tif]

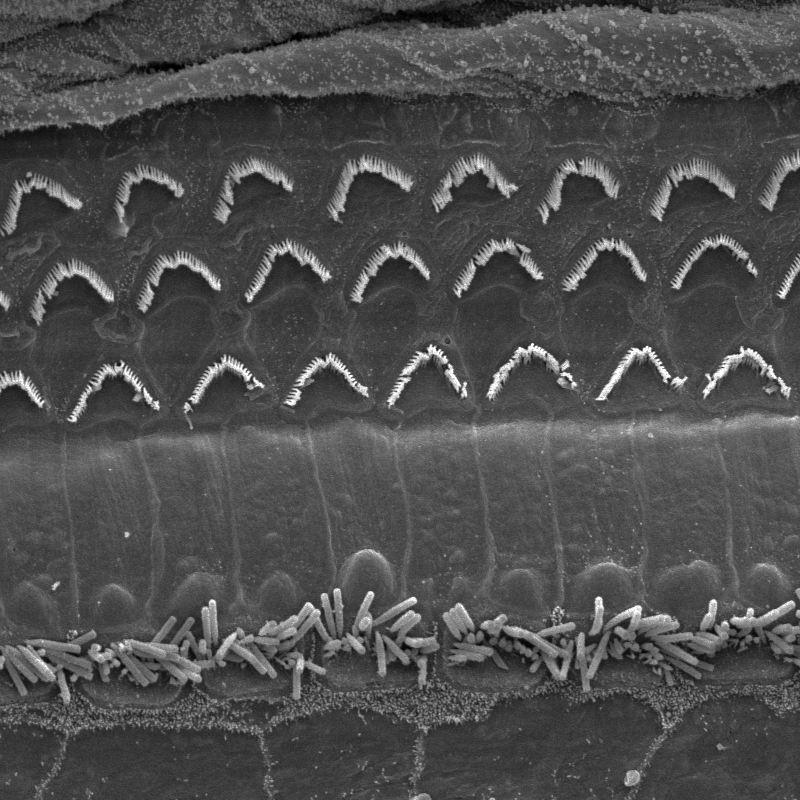

Supplement: Supplementary file 12 — Source Data for Figure 8 [file EMMM-15-e17611-s002.zip › Figure 8/8A/WT 16 kHz.tif]

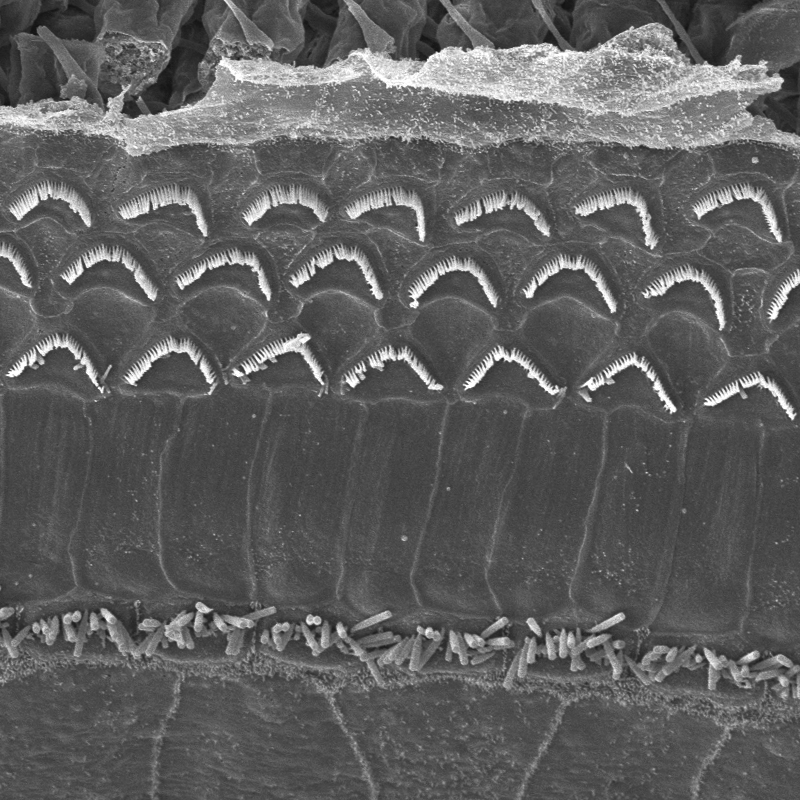

Supplement: Supplementary file 12 — Source Data for Figure 8 [file EMMM-15-e17611-s002.zip › Figure 8/8A/WT 32kHz.tif]

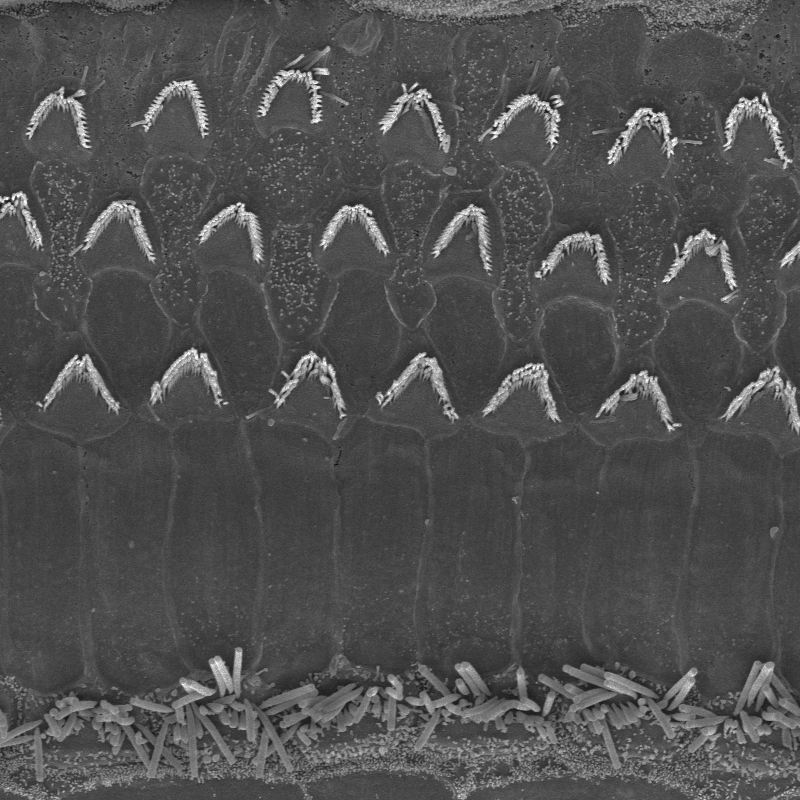

Supplement: Supplementary file 12 — Source Data for Figure 8 [file EMMM-15-e17611-s002.zip › Figure 8/8A/WT 8kHz.tif]

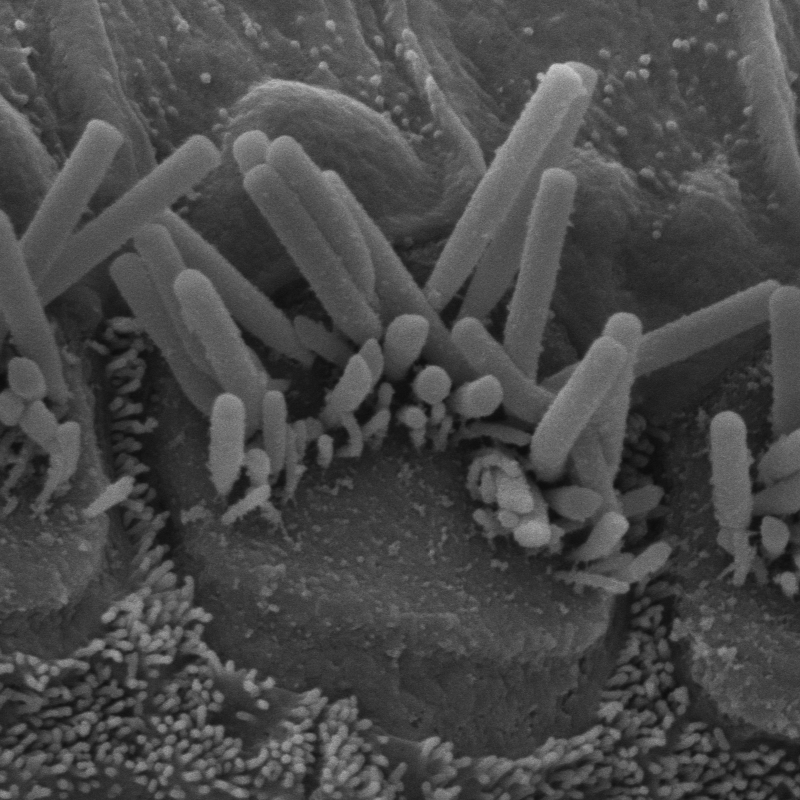

Supplement: Supplementary file 12 — Source Data for Figure 8 [file EMMM-15-e17611-s002.zip › Figure 8/8B/HO.tif]

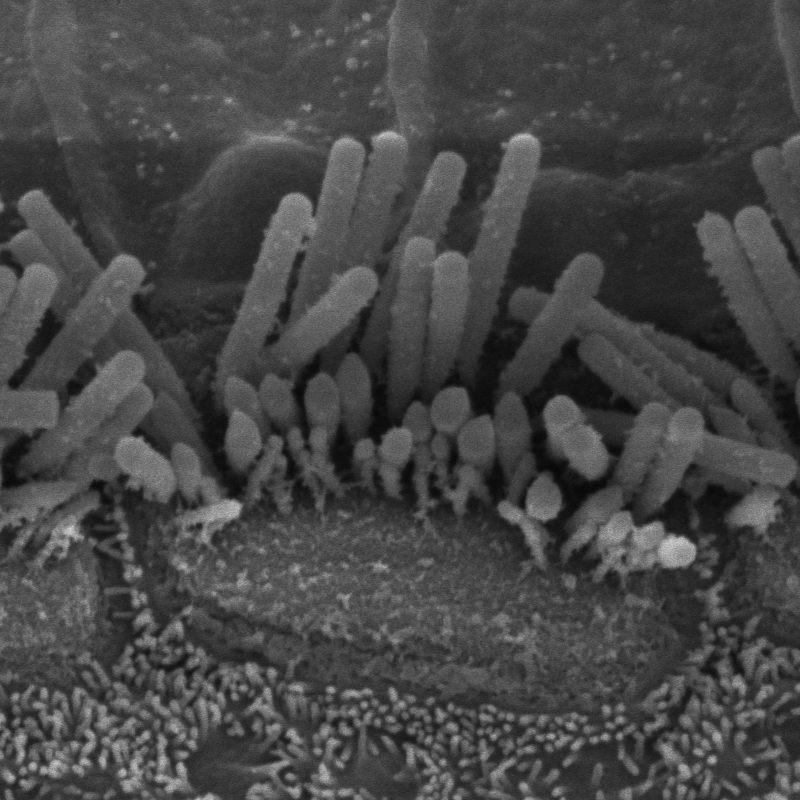

Supplement: Supplementary file 12 — Source Data for Figure 8 [file EMMM-15-e17611-s002.zip › Figure 8/8B/WT.tif]

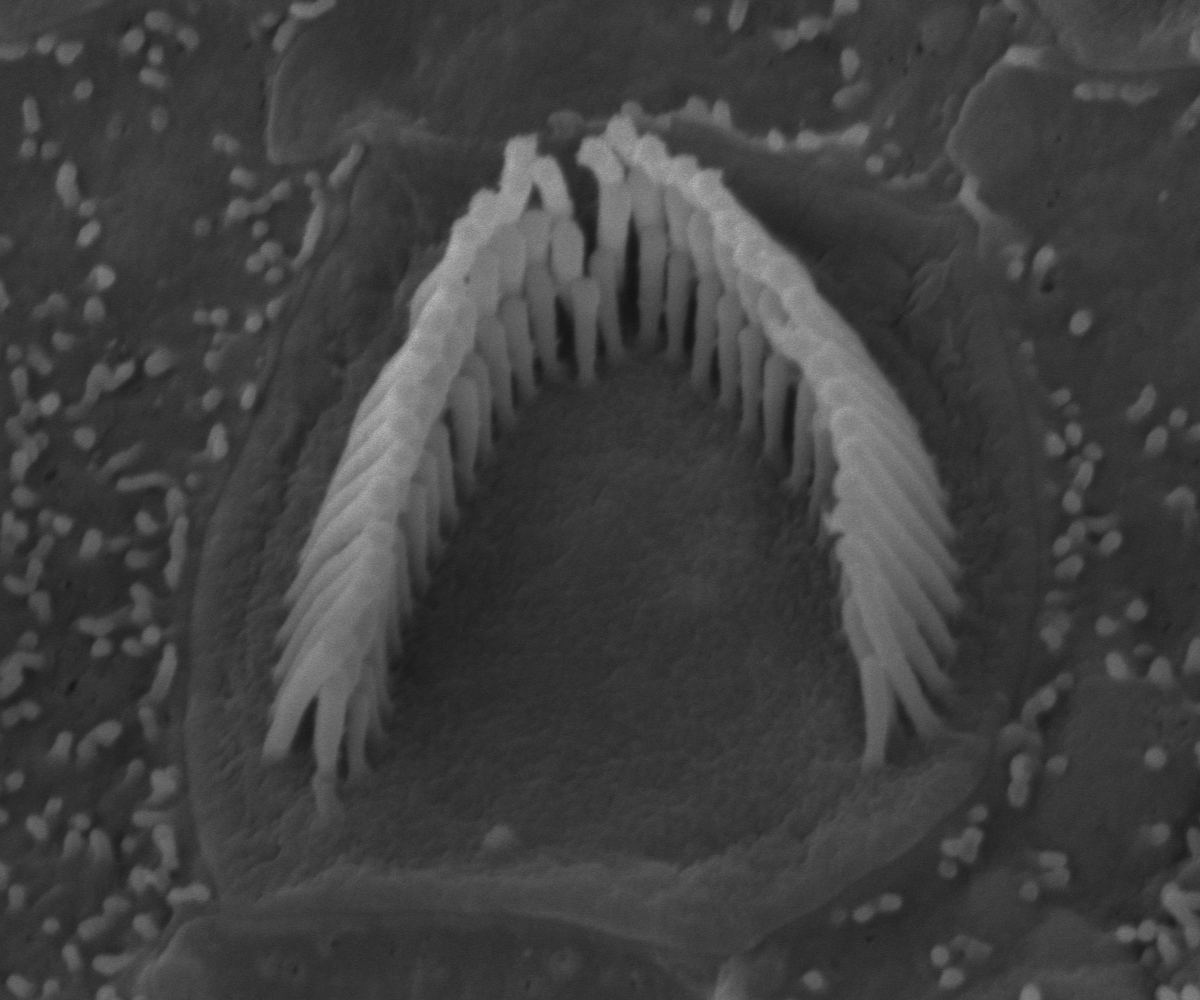

Supplement: Supplementary file 12 — Source Data for Figure 8 [file EMMM-15-e17611-s002.zip › Figure 8/8C/HO 16kHz.tif]

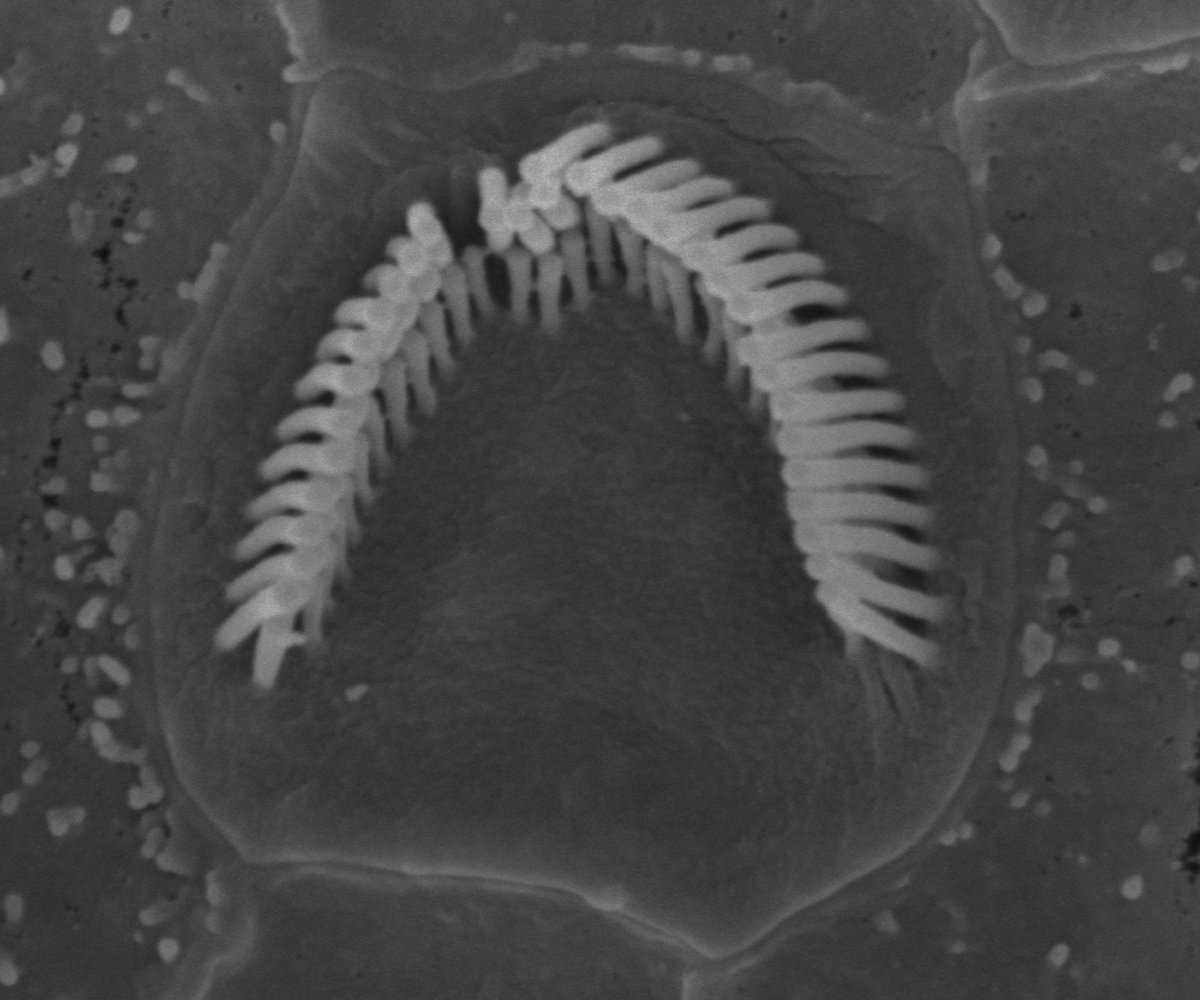

Supplement: Supplementary file 12 — Source Data for Figure 8 [file EMMM-15-e17611-s002.zip › Figure 8/8C/HO 32kHz.tif]

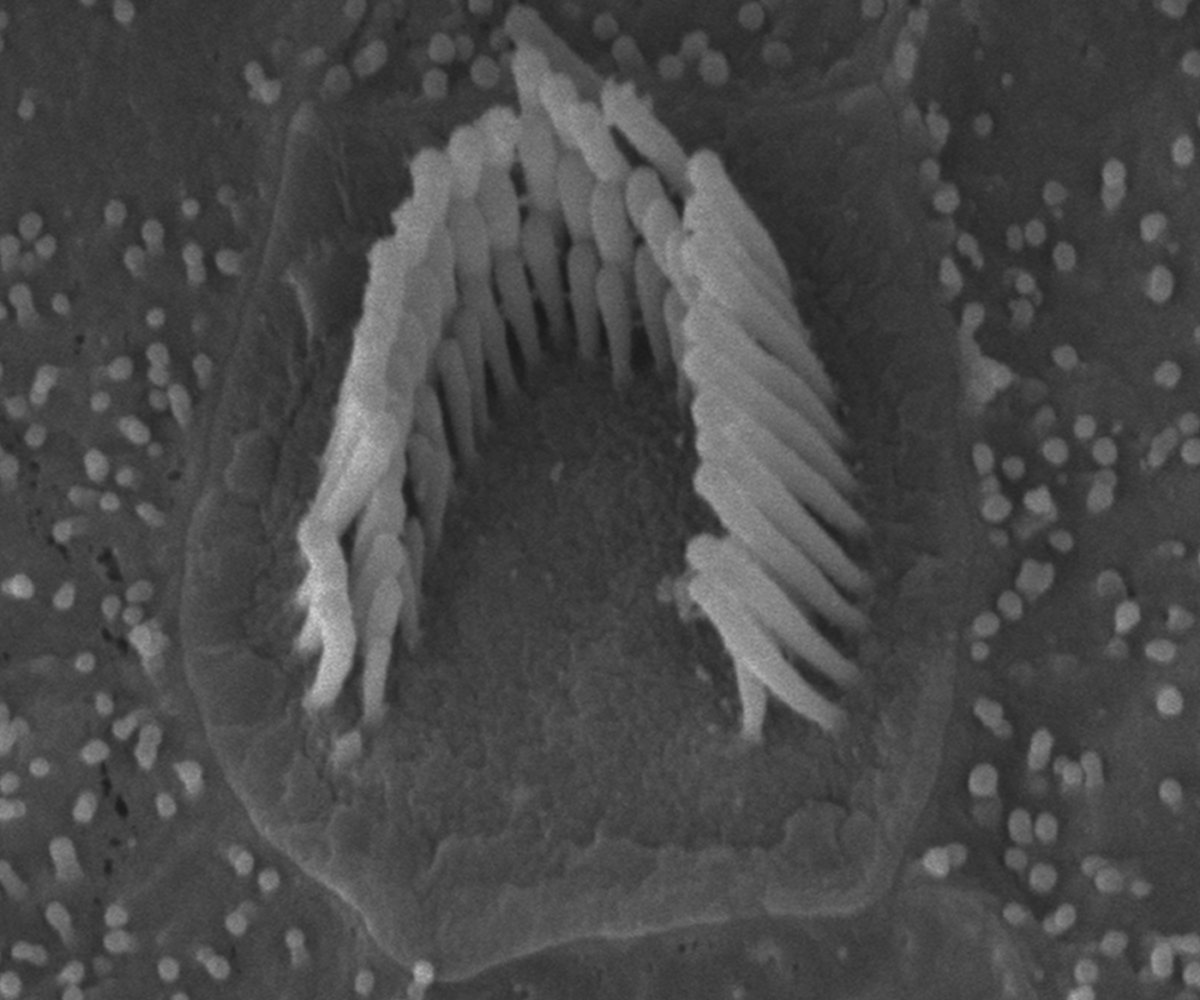

Supplement: Supplementary file 12 — Source Data for Figure 8 [file EMMM-15-e17611-s002.zip › Figure 8/8C/HO 8kHz.tif]

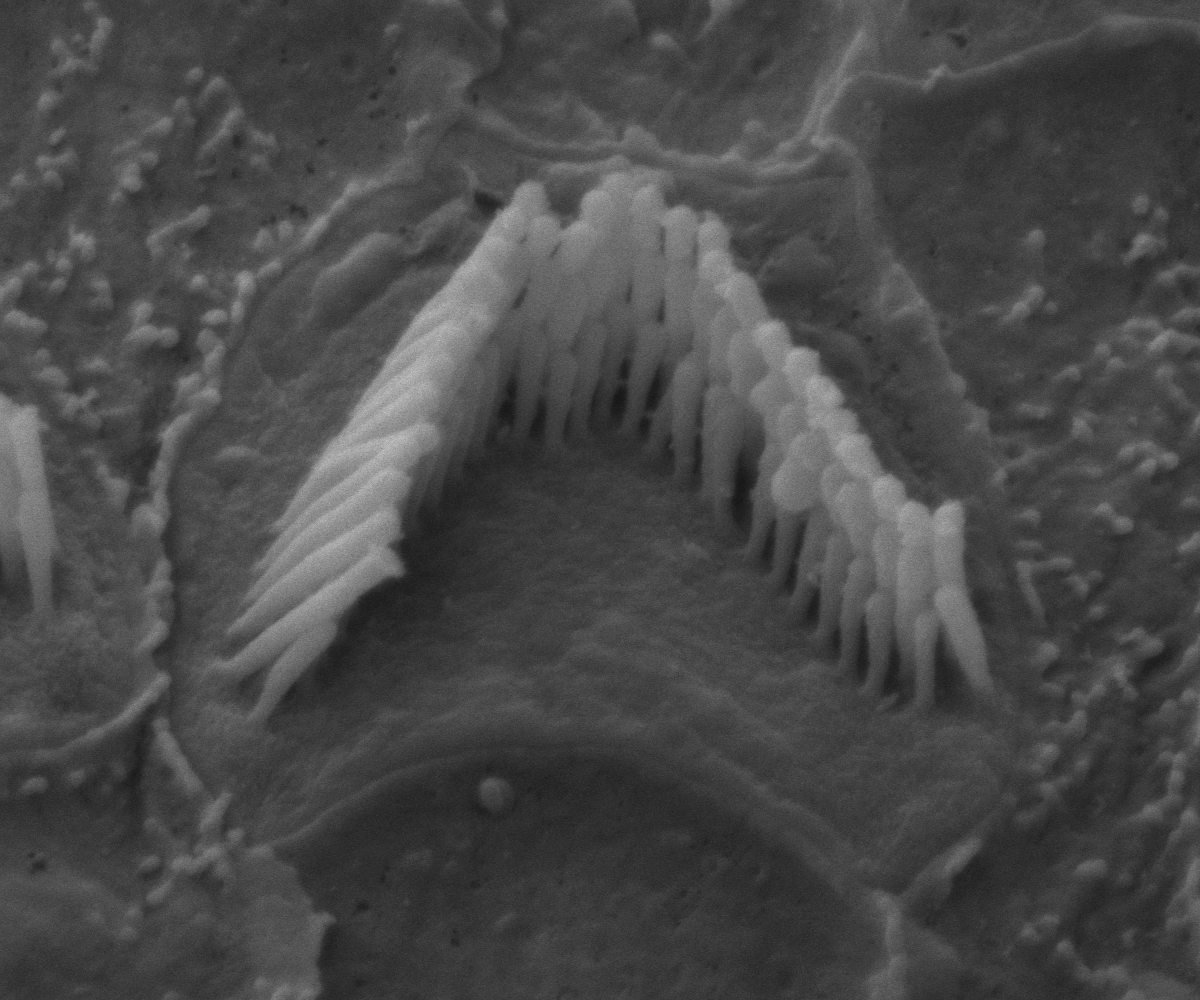

Supplement: Supplementary file 12 — Source Data for Figure 8 [file EMMM-15-e17611-s002.zip › Figure 8/8C/WT 16kHz.tif]

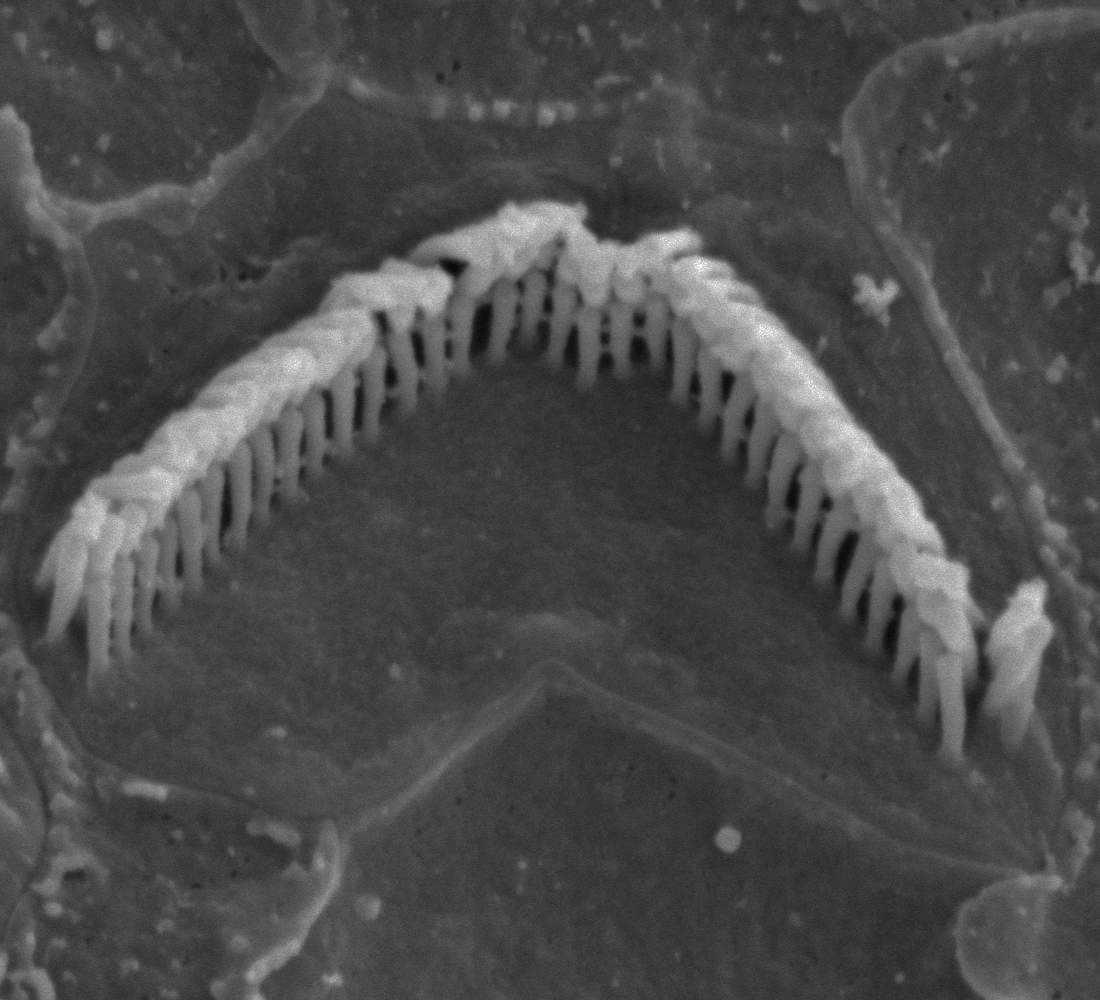

Supplement: Supplementary file 12 — Source Data for Figure 8 [file EMMM-15-e17611-s002.zip › Figure 8/8C/WT 32kHz.tif]

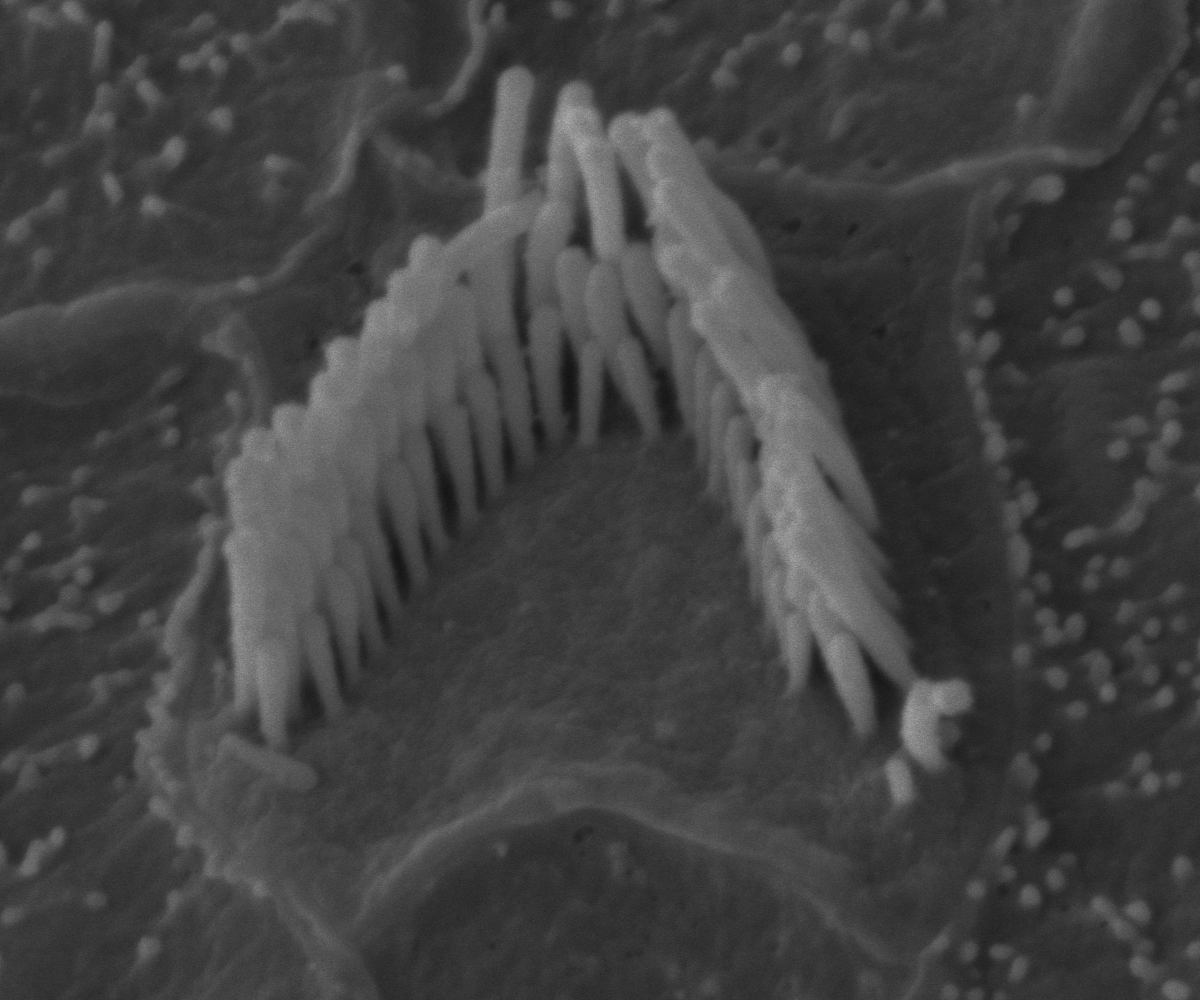

Supplement: Supplementary file 12 — Source Data for Figure 8 [file EMMM-15-e17611-s002.zip › Figure 8/8C/WT 8kHz.tif]
