# Supplementary material for: Cingulin regulates hair cell cuticular plate morphology and is required for hearing in human and mouse
Source: EMBO Mol Med. 2023 Sep 11;15(11):e17611. doi: 10.15252/emmm.202317611 (PMC10630877; doi:10.15252/emmm.202317611)
Supplement: Supplementary file 13 [file EMMM-15-e17611-s008.pdf]

EMBO Molecular Medicine

Cingulin regulates hair cell cuticular plate morphology and is required for hearing in human and mouse

Guang-Jie Zhu, Yuhang Huang, Linqing Zhang, Keji Yan, Cui Qiu, Yihan He, Qing Liu, Chengwen Zhu, Matías Morín, Miguel Ángel Moreno-Pelayo, Min-Sheng Zhu, Xin Cao, Han Zhou, Xiao-Yun Qian, Zhigang Xu, Jie Chen, Xia Gao, and Guoqiang Wan
DOI: 10.15252/emmm.202317611

Corresponding author(s): Guoqiang Wan (guoqiangwan@nju.edu.cn) , Zhigang Xu (xuzg@sdu.edu.cn), Xia Gao (gaoxia@nju.edu.cn), Jie Chen (njjiechen@163.com)

Review Timeline:

Submission Date:	21st Feb 23
Editorial Decision:	16th Mar 23
Revision Received:	21st Jul 23
Editorial Decision:	14th Aug 23
Revision Received:	17th Aug 23
Accepted:	22nd Aug 23

Editor: Zeljko Durdevic

Transaction Report:

(Note: With the exception of the correction of typographical or spelling errors that could be a source of ambiguity, letters and reports are not edited. Depending on transfer agreements, referee reports obtained elsewhere may or may not be included in this compilation. Referee reports are anonymous unless the Referee chooses to sign their reports.)

16th Mar 2023

Dear Dr. Wan,

Thank you for the submission of your manuscript to EMBO Molecular Medicine. We have now received feedback from the three reviewers who agreed to evaluate your manuscript. As you will see from the reports below, while referee #3 supports publication of the manuscript raising important but minor criticism, referees #1 and #2 recognize potential interest of the study but also raise serious and partially overlapping concerns, particularly regarding performing SEM analysis to show abnormal architecture of cuticular plate and hair bundle morphology. Further, referee #2 major point #1 regarding CGN haploinsufficiency should be addressed by quantifying the western blots and CGN immunofluorescence of the disease modeling KI mouse model. If you would like to discuss further the points raised by the referees, I am available to do so via email or video. Let me know if you are interested in this option.

Further consideration of a revision that addresses reviewers' concerns in full will entail a second round of review. EMBO Molecular Medicine encourages a single round of revision only and therefore, acceptance or rejection of the manuscript will depend on the completeness of your responses included in the next, final version of the manuscript. For this reason, and to save you from any frustrations in the end, I would strongly advise against returning an incomplete revision.

We would welcome the submission of a revised version within three months for further consideration. Please let us know if you require longer to complete the revision.

Please use this link to login to the manuscript system and submit your revision: https://embomolmed.msubmit.net/cgi-bin/main.plex

I look forward to receiving your revised manuscript.

Yours sincerely,

Zeljko Durdevic

Zeljko Durdevic
Editor
EMBO Molecular Medicine

*****

When submitting your revised manuscript, please carefully review the instructions that follow below. We perform an initial quality control of all revised manuscripts before re-review; failure to include requested items will delay the evaluation of your revision.

We require:

1) A .docx formatted version of the manuscript text (including legends for main figures, EV figures and tables). Please make sure that the changes are highlighted to be clearly visible.

2) Individual production quality figure files as .eps, .tif, .jpg (one file per figure). For guidance, download the 'Figure Guide PDF': (https://www.embopress.org/page/journal/17574684/authorguide#figureformat).

3) A .docx formatted letter INCLUDING the reviewers' reports and your detailed point-by-point responses to their comments. As part of the EMBO Press transparent editorial process, the point-by-point response is part of the Review Process File (RPF), which will be published alongside your paper.

4) A complete author checklist, which you can download from our author guidelines (https://www.embopress.org/page/journal/17574684/authorguide#submissionofrevisions). Please insert information in the checklist that is also reflected in the manuscript. The completed author checklist will also be part of the RPF.

5) Please note that all corresponding authors are required to supply an ORCID ID for their name upon submission of a revised

manuscript.

6) It is mandatory to include a 'Data Availability' section after the Materials and Methods. Before submitting your revision, primary datasets produced in this study need to be deposited in an appropriate public database, and the accession numbers and database listed under 'Data Availability'. Please remember to provide a reviewer password if the datasets are not yet public (see https://www.embopress.org/page/journal/17574684/authorguide#dataavailability).

In case you have no data that requires deposition in a public database, please state so in this section. Note that the Data Availability Section is restricted to new primary data that are part of this study.

7) For data quantification: please specify the name of the statistical test used to generate error bars and P values, the number (n) of independent experiments (specify technical or biological replicates) underlying each data point and the test used to calculate p-values in each figure legend. The figure legends should contain a basic description of n, P and the test applied. Graphs must include a description of the bars and the error bars (s.d., s.e.m.). See also 'Figure Legend' guidelines: https://www.embopress.org/page/journal/17574684/authorguide#figureformat

8) At EMBO Press we ask authors to provide source data for the main manuscript figures. Our source data coordinator will contact you to discuss which figure panels we would need source data for and will also provide you with helpful tips on how to upload and organize the files.

9) Our journal encourages inclusion of *data citations in the reference list* to directly cite datasets that were re-used and obtained from public databases. Data citations in the article text are distinct from normal bibliographical citations and should directly link to the database records from which the data can be accessed. In the main text, data citations are formatted as follows: "Data ref: Smith et al, 2001" or "Data ref: NCBI Sequence Read Archive PRJNA342805, 2017". In the Reference list, data citations must be labeled with "[DATASET]". A data reference must provide the database name, accession number/identifiers and a resolvable link to the landing page from which the data can be accessed at the end of the reference. Further instructions are available at .

10) We replaced Supplementary Information with Expanded View (EV) Figures and Tables that are collapsible/expandable online. A maximum of 5 EV Figures can be typeset. EV Figures should be cited as 'Figure EV1, Figure EV2" etc... in the text and their respective legends should be included in the main text after the legends of regular figures.

- For the figures that you do NOT wish to display as Expanded View figures, they should be bundled together with their legends in a single PDF file called *Appendix*, which should start with a short Table of Content. Appendix figures should be referred to in the main text as: "Appendix Figure S1, Appendix Figure S2" etc.

- Additional Tables/Datasets should be labeled and referred to as Table EV1, Dataset EV1, etc. Legends have to be provided in a separate tab in case of .xls files. Alternatively, the legend can be supplied as a separate text file (README) and zipped together with the Table/Dataset file.

See detailed instructions here:

.

11) The paper explained: EMBO Molecular Medicine articles are accompanied by a summary of the articles to emphasize the major findings in the paper and their medical implications for the non-specialist reader. Please provide a draft summary of your article highlighting

- the medical issue you are addressing,

- the results obtained and

- their clinical impact.

This may be edited to ensure that readers understand the significance and context of the research. Please refer to any of our published articles for an example.

12) For more information: There is space at the end of each article to list relevant web links for further consultation by our readers. Could you identify some relevant ones and provide such information as well? Some examples are patient associations, relevant databases, OMIM/proteins/genes links, author's websites, etc...

13) Author contributions: You will be asked to provide CRediT (Contributor Role Taxonomy) terms in the submission system. These replace a narrative author contribution section in the manuscript.

14) A Conflict of Interest statement should be provided in the main text.

15) Every published paper now includes a 'Synopsis' to further enhance discoverability. Synopses are displayed on the journal webpage and are freely accessible to all readers. They include a short stand first (maximum of 300 characters, including space) as well as 2-5 one-sentences bullet points that summarizes the paper. Please write the bullet points to summarize the key NEW findings. They should be designed to be complementary to the abstract - i.e. not repeat the same text. We encourage inclusion of key acronyms and quantitative information (maximum of 30 words / bullet point). Please use the passive voice. Please attach these in a separate file or send them by email, we will incorporate them accordingly.

Please also suggest a striking image or visual abstract to illustrate your article as a PNG file 550 px wide x 300-600 px high.

EMBO Molecular Medicine has a "scooping protection" policy, whereby similar findings that are published by others during review or revision are not a criterion for rejection. Should you decide to submit a revised version, I do ask that you get in touch after three months if you have not completed it, to update us on the status.

Please note: When submitting your revision you will be prompted to enter your funding and payment information. This will allow Wiley to send you a quote for the article processing charge (APC) in case of acceptance. This quote takes into account any reduction or fee waivers that you may be eligible for. Authors do not need to pay any fees before their manuscript is accepted and transferred to the publisher.

EMBO Press participates in many Publish and Read agreements that allow authors to publish Open Access with reduced/no publication charges. Check your eligibility: https://authorservices.wiley.com/author-resources/Journal-Authors/open-access/affiliation-policies-payments/index.html

***** Reviewer's comments *****

Referee #1 (Comments on Novelty/Model System for Author):

Please see my comments to the authors, especially #24. There are no ethical issues noticed.

Referee #1 (Remarks for Author):

The Zhu et al manuscript is entitled "Cingulin regulates hair cell cuticular plate morphology and is required for hearing in human and mice". The study describes one family segregating dominantly inherited deafness associated with a variant in the gene encoding cingulin and a mouse model that recapitulates the human deafness. Overall, the science is nicely described in some but not all sections and illustrated but with suggested improvements. The manuscript provides new and important data. However, there are several issues, which need to be addressed by the authors and are listed below.

According to statements in the manuscript and noting that in the Genome Browser, cingulin "Probably plays a role in the formation and regulation of the tight junction (TJ) paracellular permeability barrier, yet there is little mentioned either in the introduction or discussion sections of the Zhu manuscript about what has already been reported for the human and mouse genetics and functions of tight junctions in the auditory system. Place your study in a more complete historical context. Variants of the genes encoding claudin 9, claudin 14, tricellulin and ILDR1 (all necessary for the tight junction network in the inner ear for normal hearing) are associated with human deafness and a mutant for claudin 11 was reported about 20 years ago to be associated with deafness in mouse. There is a rich history of tight junction studies in human and mouse, and this is largely missing from the Zhu et al., manuscript. Much of this history is relevant and can be summarized in a few sentences in the Introduction along with the necessary primary references. The authors do mention published work on claudin 9 and claudin 14 in the Results section. Consider moving these few sentences about claudins and expand this section with mention (include references) about claudin 11 in mouse and ILDR1 in human and mouse to the introduction.

1. Change "mice" to "mouse" in the title to correspond to singular "human".
2. The font for the genotypes in Figure 1, panel A need to be increased in size. There is plenty of room and they are way too tiny.
3. Figure 1, panel B shows hearing thresholds. Do you know if the affected subjects in the family and, in particular the proband, have a vestibular (balance) disorder? Is cingulin necessary for normal vestibular function?
4. Figure 1, panel E, species names should be italicized.
5. Figure 1 should include a drawing of the CGN gene structure (20 exons) and the location in the exon where the variant (c.330delG) is located, not just location relative to the protein structure as nicely drawn in panel D.
6. Introduction, first sentence, I suggest not referring to hearing impairment as a disease.
7. Introduction, line 4, Substitute "associated with" for "cause" and elsewhere in the manuscript. "Cause" is hard to demonstrate.
8. Introduction, 4th paragraph, line 4, There are numerous myosins in this family of motor proteins. Which myosin are you referring to when you say ".. can regulate myosin phosphorylation..."?

9. Introduction, last paragraph, first line, change "...known deafness genes..." to "...reported deafness genes...". You don't know what is "known" but unpublished.000
10. Introduction, last paragraph, line 4, add "to" after "second".
11. Results, change "whole exome sequencing" to "exome sequencing". It was never "whole" and "whole" has been dropped in most papers published about exome sequencing in the past few years. Also delete "Whole" in the 2nd and 3rd sub-headings of the Materials and Methods section. ES not WES
12. Results, 4th paragraph, line 1, change "were" to "was". Sanger sequencing was then ...
13. Provide the allele frequency of the variant of human CGN in the various human databases and in an ethnically matched control population.
14. In the section of the Results "CGN is expressed in the mouse cochlea", indicate briefly how the specificity of the antibody against cingulin was verified when first mentioned in the main text. In the Materials and Methods section, antibodies against cingulin were purchased from Santa Cruz and Proteintech. How was the specificity of these two antibodies (and all the other antibodies used in the study) validated by the authors?
15. Delete "compelling" before "evidence". "Compelling" is not very scientific.
16. Results, "We then accessed the auditory functions of the 2 months CGN-cKO mice using...". Please revise this sentence. Do you mean 2 months old?
17. When CGN is first mentioned, add the OMIM number 609437. In OMIM, it's noted that "Citi et al. (2000) found that both human and Xenopus cingulin interacted with canine Zo1 (TJP1; 601009) and Zo2 (TJP2; 607709) in an in vitro pull-down assay." Consider mentioning these finding.
18. Discussion, 3rd to last paragraph, line 7, the reference is Perrin et al., 2009 not Perrinb et al., 2009. No "b" in the person's last name.
19. Discussion, 2nd to last paragraph, DFNA15 is mentioned. The DFNA15 gene was reported 25 years ago by Vahava et al 1998 and should be referenced here.
20. Discussion, 2nd to last paragraph, the mouse gene is Pou4f3 [italicized] and not POU4F3 [italicized], which is the human gene nomenclature.
21. In the figure legends, the number of mice tested is mentioned. What is missing is the number of male and female mice tested. Indicate the sexes of the mice tested and the number of each?
22. Figures 4 and 5, The white asterisks that label the lost OHCs are too small. Enlarge the asterisks.
23. In most of the figures in this manuscript of images of the inner ear are too small.
24. Figure 8 is an interesting model to explain the hearing loss and enhanced sensitivity to noise damage in the delG variant of Cgn [italics] (use mouse nomenclature) due an abnormal structure of the cuticular plate that fails to adequately support hair cell stereocilia. The authors are urged to provide either SEM, TEM or FIB-SEM data to support the abnormal architecture of the cuticular plate. Acquiring these additional data will take some time and it may be argued that it is an unreasonable request. The data in Figure S10 is an attempt at providing support for the model using light microscopy. But it is not entirely satisfying. In Figure S10 panel A, is the tissue from a wild type or mutant mouse? No indication in the legend.

Referee #2 (Remarks for Author):

Zhu et al describe the potential discovery of a novel rare human deafness-causing mutation. The authors perform a comprehensive clinical and audiological characterization of the affected family, and subsequently determine that a heterozygous (likely deleterious) mutation in the cingulin gene co-segregates with the hearing loss phenotype. After determining that the CGN mutation is likely causal for hearing loss, the authors perform cell culture experiments and analyze a KO and disease-modeling KI mouse model to investigate the function of CGN in hair cells. They arrive at the conclusion that CGN is expressed in hair cells, localizes to the cuticular plate, and that reduced expression of CGN causes progressive deterioration of the cuticular plate, ultimately causing hair cell death and hearing loss.

Strengths of the manuscript:
- The paper is very well written and composed, following a logical progression from clinical discovery to an understanding of the cellular and (less so) molecular mechanism.
- A wide variety of clues were followed to understand how reduced expression of CGN might affect cuticular plate integrity and hair cell function.
- The presented data is of appropriate quality and well presented, and overall support the conclusions drawn by the authors (with some weaknesses that are noted below).

Concerns:

Major:
- As noted by the authors, it is puzzling that the hearing loss phenotype in humans appears to be significantly more severe than in mice. This is not unprecedented, but still concerning, especially considering the fact that this appears to be a dominant

mutation, likely caused by CGN haploinsufficiency. While haploinsufficiency for highly critical genes (e.g. Usher genes, MET components etc) is easily envisioned, it is surprising that a ~50% reduction of a cuticular plate protein might affect hair cell function and survival to this extent. This reviewer does not exclude that possibility, but further supporting data would be desirable.
- The authors seem to suggest that the loss of hair cells is the ultimate cause for hearing loss in the CGN KO and KI mice. However, hair cell loss in these mice even at older ages is rather mild, suggesting that hair cells might become hypofunctional long before they die. This needs to be explored, using SEM analysis of hair cell and hair bundle morphology. Even if CGN is only expressed in the cuticular plate and the junctions, a hair bundle phenotype is likely to be seen well before hair cell death, as was the case in the Lmo7 KO mice (Du et al. 2019, LMO7 ...).

Medium:
- If haploinsufficiency is claimed, quantifications of western blots and IF of the KI mouse model need to be performed (Fig 5B, C).
- It would be good to quantify the postnatal increase in CGN IF in hair cells. The CGN IF in Fig 2E can be quantified by normalizing to LMO7 (B7 mouse antibody from Santa Cruz). Parvalbumin IF cannot be used because it itself changes too much postnatally.
- Figure legends do not mention statistical tests performed, for example, but not limited to 3D.
- Why does the CGN patient mutation in cell lines not cause affect mRNA levels (3D), but does so in the KI mice (5A)?
- How was recombination efficiency calculated? In S5D, recombination efficiency reported in graph and image are not consistent.

Minor:
- Images are not color blind-friendly in any figure. When using two colors, please use green and magenta, for example.
- The phrase in the introduction stating, "Interestingly, no loss-of-function mutations were identified in the list of benign variations, indicating this type of mutations are non-tolerated in the CGN gene as they could be eventually impacting the function of the protein," should be changed or modified. Previous and independent research using different mouse models show that CGN knockout can be viable and relatively healthy (Schossleitner et al., 2016; Guillemot et al., 2012), which contradicts the authors' later argument that homozygous CGN knockout seems to be embryonic lethal.
- The starring of the damaged hair cells in multiple figures obstruct the relevant areas in the image. Use of arrows is preferred.
- Move S10A into figure 7 so that it is intuitive how calculations are done.
- In the results sub-section "CGNdelG mice show normal synaptic density, tight junction and microtubule structures," authors do not consider the possibility that some pathology could be attributed to the mislocalization of tight junction components within hair cells as a result of mutated CGN, despite these mice having similar TJ mRNA levels relative to mice with WT CGN. S8G IF results with ZO1 does seem to indicate abnormal localization in the CGNdelG/delG mice
- The authors should cite CGN expression data as curated in the gEAR database. Especially the RNAseq data from the Chen lab shows a nice postnatal increase of cingulin expression in hair cells, consistent with their data in Fig.2.
- Provide sections or side view reconstructions of CGN IF, to better illustrate CGN localization in the cuticular plate.
- Minor edits to language and grammar are needed, particularly in the discussion section.

Referee #3 (Remarks for Author):

This manuscript reports the identification of a new deafness gene that encodes for a protein called "Cingulin" (CGN) previously reported to be associated with tight junctions in epithelial cells. This discovery arose from identification of a pathogenic non syndromic mutation (CGN delG) in a multi-generation family pedigree. Cingulin is shown to be enriched at the hair cell cuticular plate and circumferential belts. This report demonstrates that the deletion mutation, carried by the family, results in reduced expression and abnormal localization of CGN in transfected cell lines. Furthermore, generation of conditional knockout and knockin mice demonstrates that they are affected by progressive high frequency hearing loss and are sensitive to noise-induced hearing loss. Visualization of F-actin structures demonstrates the role of CGN in actin polymerization with alterations in actin-rich cuticular plates shapes in CGN mutant mice.

This is an important and comprehensive study that combines human, cell and animal work. It is very well written and the conclusions and discussion are appropriate. The figures are very clear and well labeled. Statistical analysis are described and appropriate.

Suggested add-on:
- CGN is also highly expressed in IHCs, however the authors suggest that lack or reduction in CGN mainly affects OHCs. Were there no evidence of cuticular plate malformation in IHCs at 2 months (figure 7)? Could it be that aging the mice past 6 month might reveal alterations in IHCs as well?
- Is CGN expressed in vestibular hair cells and do the mice display behavioral deficits?

- Some minor english mistakes are noted that should be corrected (tenses).

Response to Editor

Dear Dr. Wan,

Thank you for the submission of your manuscript to EMBO Molecular Medicine. We have now received feedback from the three reviewers who agreed to evaluate your manuscript. As you will see from the reports below, while referee #3 supports publication of the manuscript raising important but minor criticism, referees #1 and #2 recognize potential interest of the study but also raise serious and partially overlapping concerns, particularly regarding performing SEM analysis to show abnormal architecture of cuticular plate and hair bundle morphology. Further, referee #2 major point #1 regarding CGN haploinsufficiency should be addressed by quantifying the western blots and CGN immunofluorescence of the disease modeling KI mouse model. If you would like to discuss further the points raised by the referees, I am available to do so via email or video. Let me know if you are interested in this option.

Further consideration of a revision that addresses reviewers' concerns in full will entail a second round of review. EMBO Molecular Medicine encourages a single round of revision only and therefore, acceptance or rejection of the manuscript will depend on the completeness of your responses included in the next, final version of the manuscript. For this reason, and to save you from any frustrations in the end, I would strongly advise against returning an incomplete revision.

We would welcome the submission of a revised version within three months for further consideration. Please let us know if you require longer to complete the revision.

Please use this link to login to the manuscript system and submit your revision:
https://embomolmed.msubmit.net/cgi-bin/main.plex

I look forward to receiving your revised manuscript.

Yours sincerely,

Zeljko Durdevic

Editor

EMBO Molecular Medicine

Response: Thank you for providing the opportunity to revise our manuscript. In the revision, we have performed additional experiments to address the questions you've summarized (Cgn^{delG} mice SEM analysis and haploinsufficiency validation). Specifically, we have:

1) performed SEM analysis of 2-months old WT and $Cgn^{delG/delG}$ mouse cochlea, which not only confirmed the morphological alteration of the cuticular plates but also showed altered hair cell bundles in *Cgn* mutant mice (**revised Fig 8**);

2) performed WB and IF quantification of the WT and Cgn^{delG} KI cochlea to demonstrate Cgn haploinsufficiency (**revised Figs 5B and EV4**);

In addition, we have now showed Cgn expression in mouse utricle and performed vestibular function tests of the knockin mice (**revised Fig EV5**). More detailed revision and responses can be found in "Point-to-point response to reviewers" below. Major changes in texts are also highlighted in blue in the revised manuscript.

Point-to-point response to reviewers

******* Reviewer's comments *******

Referee #1 (Comments on Novelty/Model System for Author):

Please see my comments to the authors, especially #24. There are no ethical issues noticed.

Referee #1 (Remarks for Author):

The Zhu et al manuscript is entitled "Cingulin regulates hair cell cuticular plate morphology and is required for hearing in human and mice". The study describes one family segregating dominantly inherited deafness associated with a variant in the gene encoding cingulin and a mouse model that recapitulates the human deafness. Overall, the science is nicely described in some but not all sections and illustrated but with suggested improvements. The manuscript provides new and important data. However, there are several issues, which need to be addressed by the authors and are listed below.

According to statements in the manuscript and noting that in the Genome Browser, cingulin "Probably plays a role in the formation and regulation of the tight junction (TJ) paracellular permeability barrier, yet there is little mentioned either in the introduction or discussion sections of the Zhu manuscript about what has already been reported for the human and mouse genetics and functions of tight junctions in the auditory system. Place your study in a more complete historical context. Variants of the genes encoding claudin 9, claudin 14, tricellulin and ILDR1 (all necessary for the tight junction network in the inner ear for normal hearing) are associated with human deafness and a mutant for claudin 11 was reported about 20 years ago to be associated with deafness in mouse. There is a rich history of tight junction studies in human and mouse, and this is largely missing from the Zhu et al., manuscript. Much of this history is relevant and can be summarized in a few sentences in the Introduction along with the necessary primary references. The authors do mention published work on claudin 9 and claudin 14 in the Results section. Consider moving these few sentences about claudins and expand this section with mention (include references) about claudin 11 in mouse and ILDR1 in human and mouse to the introduction.

Response: We thank reviewer for this important and critical comment. We have now added a paragraph in the Introduction describing TJs and related proteins in hearing and deafness.

"Tight junctions (TJs) are specialized intercellular junctions that form a continuous, belt-like seal between adjacent cells in epithelial and endothelial tissues (Sawada, Murata et al., 2003). TJs in the inner ear are critical for maintenance of the unique endolymphatic environment and generation of the endocochlear potential (Gao, Chen et al., 2023). Defects in multiple tight junction-related proteins, such as ZO-2 (*TJP2*), claudin-9 (*CLDN9*), claudin-14 (*CLDN14*) and claudin-11 (*CLDN11*) are associate with hearing loss in human and mouse (Gow, Davies et al., 2004, Sineni, Yildirim-Baylan et al., 2019, Walsh, Pierce et al., 2010, Wilcox, Burton et al., 2001). In addition, mutations to the tricellular TJ components, including tricellulin (*TRIC*) and angulin-2 (*ILDR1*) also results in human deafness (Borck, Ur Rehman et al., 2011, Riazuddin, Ahmed et al., 2006), which can be recapitulated in knockout mouse models (Morozko, Nishio et al., 2015, Nayak, Lee et al., 2013). These reports suggest that the integrity of cochlear TJs is pivotal to the auditory function."

1. Change "mice" to "mouse" in the title to correspond to singular "human".

Response: Revised, thanks.

2. The font for the genotypes in Figure 1, panel A need to be increased in size. There is plenty of room and they are way too tiny.

Response: We have increased the font of genotypes.

3. Figure 1, panel B shows hearing thresholds. Do you know if the affected subjects in the family and, in particular the proband, have a vestibular (balance) disorder? Is cingulin necessary for normal vestibular function?

Response: The affected subjects in the family, including the proband, have no vestibular (balance) disorder. The Cgn^{delG} mice did not display nodding or circling behaviors. To address potential role of Cgn in the balance organ, we have now shown Cgn expression in the postnatal mouse utricle, and performed Rotarod tests to examine vestibular function of the mutant mice (**revised Fig EV5**). Although Cgn is also highly expressed in the utricular macula epithelia, it is not required for vestibular function.

4. Figure 1, panel E, species names should be italicized.

Response: Revised, thanks.

5. Figure 1 should include a drawing of the CGN gene structure (20 exons) and the location in the exon where the variant (c.330delG) is located, not just location relative to the protein structure as nicely drawn in panel D.

Response: We have included the CGN gene exon structure with position of the variant (**revised Fig 1D**).

6. Introduction, first sentence, I suggest not referring to hearing impairment as a disease.

Response: Thanks, we have now revised this sentence, it now reads: "Based on recent world report on hearing from the World Health Organization, more than 430 million people worldwide suffer from disabling hearing loss (Chadha, Kamenov et al., 2021)".

7. Introduction, line 4, Substitute "associated with" for "cause" and elsewhere in the manuscript. "Cause" is hard to demonstrate.

Response: Thanks, we have revised accordingly.

8. Introduction, 4th paragraph, line 4, There are numerous myosins in this family of motor proteins. Which myosin are you referring to when you say ".. can regulate myosin phosphorylation..."?

Response: The reference did not mention which myosin it is. They examined total myosin light chain phosphatase (MYP) and phosphorylated myosin light chain phosphatase (p-MYP). However, a very recent paper (published in J Cell Biol. in May 2023) by Citi group showed that myosin-2 is tethered by cingulin. We have now revised with this new reference.

"Studies have shown that CGN can regulate myosin phosphorylation through RhoA and Rac1 (Aijaz, D'Atri et al., 2005), tether the nonmuscle myosin-2 to the junction (Rouaud, Huang et al., 2023), regulate the activity of Rho family GTPase, and thereby regulate the formation and membrane tortuosity of the epithelial tight junctions (Gonzalez-Mariscal, Dominguez-Calderon et al., 2014)."

9. Introduction, last paragraph, first line, change "...known deafness genes..." to "...reported deafness genes...". You don't know what is "known" but unpublished.

Response: Revised, thanks.

10. Introduction, last paragraph, line 4, add "to" after "second".

Response: We have specified the exact exonic location of the variant (i.e. exon 20), instead of its relative position (i.e. second last exon).

11. Results, change "whole exome sequencing" to "exome sequencing". It was never "whole" and "whole" has been dropped in most papers published about exome sequencing in the past few years. Also delete "Whole" in the 2nd and 3rd sub-headings of the Materials and Methods section. ES not WES

Response: Thanks, we have revised accordingly.

12. Results, 4th paragraph, line 1, change "were" to "was". Sanger sequencing was then ...

Response: Revised, thanks.

13. Provide the allele frequency of the variant of human CGN in the various human databases and in an ethnically matched control population.

Response: This variant of human CGN was not present in the currently available databases. We have revised: "This mutation was predicted to be deleterious by the Mutation Taster, PolyPhen-2, and SIFT computational tools and was not present in the dbSNP, 1000 Genomes, ESP6500, nci60, GnomAD databases and 10,588 ethically matched control individuals (Cao, Li et al., 2020)."

14. In the section of the Results "CGN is expressed in the mouse cochlea", indicate briefly how the specificity of the antibody against cingulin was verified when first mentioned in the main text. In the Materials and Methods section, antibodies against cingulin were purchased from Santa Cruz and Proteintech. How was the specificity of these two antibodies (and all the other antibodies used in the study) validated by the authors?

Response: Three CGN antibodies were used in this study (as specified in the materials and methods). We have now provided validation results as the **revised Appendix Fig 2**. The other key antibodies, such as Myo7a, Parvalbumin, LMO7 and Spectrin β II were selected based on previous publications in the research field (Du, Ye et al., 2020, Du, Dewey et al., 2019, Liu, Qi et al., 2019, Zhu, Gong et al., 2020).

15. Delete "compelling" before "evidence". "Compelling" is not very scientific.

Response: Thanks, we've changed to "indicate".

16. Results, "We then accessed the auditory functions of the 2 months CGN-cKO mice using...". Please revise this sentence. Do you mean 2 months old?

Response: Sorry for the confusion, we've revised to "2 months old".

17. When CGN is first mentioned, add the OMIM number 609437. In OMIM, it's noted that "Citi et al. (2000) found that both human and Xenopus cingulin interacted with canine Zo1 (TJP1; 601009) and Zo2 (TJP2; 607709) in an in vitro pull-down assay." Consider mentioning these finding.

Response: We've added OMIM number in the introduction when CGN is first mentioned. We've also added a sentence with the relevant citations: "CGN also interacts with TJ proteins ZO-1 and ZO-2, serving as the linkage between TJs and the actin cytoskeleton (Citi, D'Atri et al., 2000, Citi, Pulimeno et al., 2012)."

18. Discussion, 3rd to last paragraph, line 7, the reference is Perrin et al., 2009 not Perrinb et al., 2009. No "b" in the person's last name.

Response: Revised, thanks.

19. Discussion, 2nd to last paragraph, DFNA15 is mentioned. The DFNA15 gene was reported 25 years ago by Vahava et al 1998 and should be referenced here.

Response: We've added this important reference.

20. Discussion, 2nd to last paragraph, the mouse gene is Pou4f3 [italicized] and not POU4F3 [italicized], which is the human gene nomenclature.

Response: Revised, thanks.

21. In the figure legends, the number of mice tested is mentioned. What is missing is the number of male and female mice tested. Indicate the sexes of the mice tested and the number of each?

Response: We have specified the number of male and female mice used as requested by the reviewer. The details are provided in the revised figure legends.

22. Figures 4 and 5, The white asterisks that label the lost OHCs are too small. Enlarge the asterisks.

Response: We have changed the small asterisks to bigger arrows.

23. In most of the figures in this manuscript of images of the inner ear are too small.

Response: Images of the cochlear sensory epithelia have been enlarged in the revised figures.

24. Figure 8 is an interesting model to explain the hearing loss and enhanced sensitivity to noise damage in the delG variant of Cgn [italics] (use mouse nomenclature) due an abnormal structure of the cuticular plate that fails to adequately support hair cell stereocilia. The authors are urged to provide either SEM, TEM or FIB-SEM data to support the abnormal architecture of the cuticular plate. Acquiring these additional data will take some time and it may be argued that it is an unreasonable request. The data in Figure S10 is an attempt at providing support for the model using light microscopy. But it is not entirely satisfying. In Figure S10 panel A, is the tissue from a wild type or mutant mouse? No indication in the legend.

Response: Thanks to the reviewer for this insightful question. We have performed SEM analysis of 2-months old WT and *Cgn*$^{delG/delG}$ mouse cochlea to examine both cuticular plate and hair bundles. The new result (**revised Fig 8**) confirmed the morphological alteration of the cuticular plates observed with immunofluorescence. In addition, the opening angles of the hair bundles were also altered in the *Cgn* mutant mice, likely caused by altered rigidity of the underlying cuticular plate.

Figure S10 panel A (**revised Fig 7D**) was from a wild type mouse. It is now specified in the legend.

Referee #2 (Remarks for Author):

Zhu et al describe the potential discovery of a novel rare human deafness-causing mutation. The authors perform a comprehensive clinical and audiological characterization of the affected family, and subsequently determine that a heterozygous (likely deleterious) mutation in the cingulin gene co-segregates with the hearing loss phenotype. After determining that the CGN mutation is likely causal for hearing loss, the authors perform cell culture experiments and analyze a KO and disease-modeling KI mouse model to investigate the function of CGN in hair cells. They arrive at the conclusion that CGN is expressed in hair cells, localizes to the cuticular plate, and that reduced expression of CGN causes progressive deterioration of the cuticular plate, ultimately causing hair cell death and hearing loss.

Strengths of the manuscript:

- The paper is very well written and composed, following a logical progression from clinical discovery to an understanding of the cellular and (less so) molecular mechanism.

- A wide variety of clues were followed to understand how reduced expression of CGN might affect cuticular plate integrity and hair cell function.

- The presented data is of appropriate quality and well presented, and overall support the conclusions drawn by the authors (with some weaknesses that are noted below).

Concerns:

Major:

- As noted by the authors, it is puzzling that the hearing loss phenotype in humans appears to be significantly more severe than in mice. This is not unprecedented, but still concerning, especially considering the fact that this appears to be a dominant mutation, likely caused by CGN haploinsufficiency. While haploinsufficiency for highly critical genes (e.g. Usher genes, MET components etc) is easily envisioned, it is surprising that a ~50% reduction of a cuticular plate protein might affect hair cell function and survival to this extent. This reviewer does not exclude that possibility, but further supporting data would be desirable.

Response: Thanks for the insightful comment. As we have shown in the manuscript, the heterozygous knockin mice showed progressive hearing loss that was exacerbated by aging and noise exposure. *These data support the notion that mutation of Cgn in single allele is already causing damage to the cochlear function, which is consistent with dominant inheritance pattern of the deafness.* The discrepancy in disease severity between human patients and mouse model is nonetheless puzzling, for which we are currently exploring other possibilities. For example, we are working on patient-derived iPSCs to recapitulate Cgn-mediated cytoskeletal changes to avoid difference in species, and we are constructing new animal models by overexpressing the human Cgn mutant protein in mouse cochlear cells to counteract the effect of non-sense mediated RNA decay. However, these approaches will take some time to yield meaningful results, and also may not reflect what happens *in vivo* in human.

Works from the recent 10 years have highlighted how environmental factors, such as aging and noise, can modified hearing loss phenotype in genetic deafness mouse models (Petit, Bonnet et al., 2023). For example, we recently show that DFNA15 (*POU4F3*) mouse model is susceptible to noise,

aging and genetic background (Zhu et al., 2020). Hearing loss in DFNB59 (*PVJK*) and *Foxo3*-KO mouse models is also greatly influenced by prior moderate noise exposure (Delmaghani, Defourny et al., 2015, Gilels, Paquette et al., 2017). Probably most remarkably, *P2rx2*-null mouse, which models DFNA41, only show hearing loss at 17 months of age but are susceptible to noise exposure at 3 months of age (Yan, Zhu et al., 2013). Differences in the environmental factors between human and mouse models may be a confounding issue for matched disease severity and progression.

- The authors seem to suggest that the loss of hair cells is the ultimate cause for hearing loss in the CGN KO and KI mice. However, hair cell loss in these mice even at older ages is rather mild, suggesting that hair cells might become hypofunctional long before they die. This needs to be explored, using SEM analysis of hair cell and hair bundle morphology. Even if CGN is only expressed in the cuticular plate and the junctions, a hair bundle phenotype is likely to be seen well before hair cell death, as was the case in the Lmo7 KO mice (Du et al. 2019, LMO7 ...).

Response: Thanks to the reviewer for this insightful question. We have performed SEM analysis of 2-months old WT and $Cgn^{delG/delG}$ mouse cochlea to examine both cuticular plate and hair bundles. The new result (**revised Fig 8**) confirmed the morphological alteration of the cuticular plates observed with immunofluorescence. In addition, the opening angles of the hair bundles were also altered in the *Cgn* mutant mice, likely caused by altered rigidity of the underlying cuticular plate.

Medium:

- If haploinsufficiency is claimed, quantifications of western blots and IF of the KI mouse model need to be performed (Fig 5B, C).

Response: We thank reviewer for this advice. Quantifications of the Western blots and IF results have been carried out and presented in the **revised Figs 5B and EV4**.

- It would be good to quantify the postnatal increase in CGN IF in hair cells. The CGN IF in Fig 2E can be quantified by normalizing to LMO7 (B7 mouse antibody from Santa Cruz). Parvalbumin IF cannot be used because it itself changes too much postnatally.

Response: We performed additional immunostaining of P3, P14 and P21 cochlea with CGN and LMO7 antibodies, the expression levels of CGN at cuticular plates were quantified and normalized to the LMO7 signal (**revised Fig 2G**).

- Figure legends do not mention statistical tests performed, for example, but not limited to 3D.

Response: We've added the statistical tests used in relevant figure legends.

- Why does the CGN patient mutation in cell lines not cause affect mRNA levels (3D), but does so in the KI mice (5A)?

Response: We believe the *Cgn* mutation in the KI mice results in mRNA degradation via nonsense-mediated mRNA decay (NMD), as the mutation produces a frame-shifted mRNA with premature termination codon. In contrast, in the cell lines, we only overexpressed the coding sequence without 3'UTR, thus the mutant transcript will avoid NMD and allow us to examine the effect of patient mutation on protein function more clearly.

- How was recombination efficiency calculated? In S5D, recombination efficiency reported in graph and image are not consistent.

Response: *Cgn-cKO* mice were injected with tamoxifen to induce recombination, one cochlea of each mouse was dissected for evaluation of the recombination efficiency, which was calculated as the ratio of recombined hair cells to the total number of hair cells in each image. In the revised figure, we've now provided an image that is more representative of the average recombination efficiency.

Minor:

- Images are not color blind-friendly in any figure. When using two colors, please use green and magenta, for example.

Response: We have revised, thanks for this suggestion.

- The phrase in the introduction stating, "Interestingly, no loss-of-function mutations were identified in the list of benign variations, indicating this type of mutations are non-tolerated in the CGN gene as they could be eventually impacting the function of the protein," should be changed or modified. Previous and independent research using different mouse models show that CGN knockout can be viable and relatively healthy (Schossleitner et al., 2016; Guillemot et al., 2012), which contradicts the authors' later argument that homozygous CGN knockout seems to be embryonic lethal.

Response: Thanks to the reviewer for this question. We had also paid attention to these two articles mentioned by the reviewer. The Cgn knockout model used in these studies was generated by deleting exon 1 of the mouse *Cgn* gene, which results in a truncated version of Cgn missing the head domain. However, a potential alternative in frame transcription start site is present in exon 4 (Figure 5 from Schossleitner et al., 2016). The truncated Cgn may still be able to interact with MAPKs, Rho-GEF and Rho-GAP proteins via its rod domains, thus preserving much of its physiological function.

Nevertheless, we have removed this sentence which is speculative. We've also removed the citation to the International mouse phenotyping consortium data (https://www.mousephenotype.org/data/genes/MGI:1927237) as it is not a focus of our study.

- The starring of the damaged hair cells in multiple figures obstruct the relevant areas in the image. Use of arrows is preferred.

Response: We have changed the asterisks to arrows.

- Move S10A into figure 7 so that it is intuitive how calculations are done.

Response: Revised, thanks.

- In the results sub-section "CGNdelG mice show normal synaptic density, tight junction and microtubule structures," authors do not consider the possibility that some pathology could be attributed to the mislocalization of tight junction components within hair cells as a result of mutated CGN, despite these mice having similar TJ mRNA levels relative to mice with WT CGN. S8G IF results with ZO1 does seem to indicate abnormal localization in the CGNdelG/delG mice.

Response: We've provided new images to better demonstrate the localization of ZO-1 in WT and *CGN*^{delG} mice (**revised Appendix Fig S5G**). The localization of ZO-1 appeared normal in cochlea of the mutant mice.

- The authors should cite CGN expression data as curated in the gEAR database. Especially the RNAseq data from the Chen lab shows a nice postnatal increase of cingulin expression in hair cells, consistent with their data in Fig.2.

Response: Thanks to the reviewer for this suggestion. We had some issue with some of the gEAR datasets; nevertheless, we obtained *Cgn* expression from cell-type-specific RNA-seq results (Liu, Chen et al., 2018), which was also deposited to the gEAR database. The re-analyzed results demonstrate that *Cgn* was widely expressed in P28-35 cochlear sensory epithelia cells, with enriched expression in both inner and outer hair cells (**revised Fig 2E**).

- Provide sections or side view reconstructions of CGN IF, to better illustrate CGN localization in the cuticular plate.

Response: We performed new co-immunostaining experiments with CGN and LMO7 at P3, P14 and P21. Side view reconstructions of these images were also shown, which demonstrate location of Cgn at both circumferential belts and cuticular plates (**revised Fig 2G**).

- Minor edits to language and grammar are needed, particularly in the discussion section.

Response: We've revised the text with help from native English speakers.

Referee #3 (Remarks for Author):

This manuscript reports the identification of a new deafness gene that encodes for a protein called "Cingulin" (CGN) previously reported to be associated with tight junctions in epithelial cells. This discovery arose from identification of a pathogenic non syndromic mutation (CGN delG) in a multi-generation family pedigree. Cingulin is shown to be enriched at the hair cell cuticular plate and circumferential belts. This report demonstrates that the deletion mutation, carried by the family, results in reduced expression and abnormal localization of CGN in transfected cell lines. Furthermore, generation of conditional knockout and knockin mice demonstrates that they are affected by progressive high frequency hearing loss and are sensitive to noise-induced hearing loss. Visualization of F-actin structures demonstrates the role of CGN in actin polymerization with alterations in actin-rich cuticular plates shapes in CGN mutant mice.

This is an important and comprehensive study that combines human, cell and animal work. It is very well written and the conclusions and discussion are appropriate. The figures are very clear and well labeled. Statistical analysis are described and appropriate.

Suggested add-on:

- CGN is also highly expressed in IHCs, however the authors suggest that lack or reduction in CGN mainly affects OHCs. Were there no evidence of cuticular plate malformation in IHCs at 2 months (figure 7)? Could it be that aging the mice past 6 month might reveal alterations in IHCs as well?

Response: Thanks to the reviewer for this question. We did not observe apparent changes to the IHC cuticular plate morphology based on the immunofluorescent staining (Fig 7E and J). In addition, we performed SEM analyses of 2-months old WT and $Cgn^{delG/delG}$ mouse cochlea to examine both cuticular plate and hair bundles (**revised Fig. 8**), and we fail to observe alterations in cuticular plates or hair bundles of IHCs. While it is possible that IHC microstructures may be affected at older ages, they are less likely to play a major role in the auditory dysfunction of the mutant mice.

- Is CGN expressed in vestibular hair cells and do the mice display behavioral deficits?

Response: Yes, Cgn is also expressed in vestibular hair cells. In the revised manuscript, we've shown Cgn expression in the postnatal mouse utricle, and performed Rotarod tests to examine vestibular function of the mutant mice (**revised Fig EV5**). Although Cgn is highly expressed in the utricular macula epithelia, it is not required for vestibular function.

- Some minor english mistakes are noted that should be corrected (tenses).

Response: Thanks, we've revised the text with help from native English speakers.

References for Reviewers

Aijaz S, D'Atri F, Citi S, Balda MS, Matter K (2005) Binding of GEF-H1 to the tight junction-associated adaptor cingulin results in inhibition of Rho signaling and G1/S phase transition. *Dev Cell* 8: 777-86

Borck G, Ur Rehman A, Lee K, Pogoda HM, Kakar N, von Ameln S, Grillet N, Hildebrand MS, Ahmed ZM, Nurnberg G, Ansar M, Basit S, Javed Q, Morell RJ, Nasreen N, Shearer AE, Ahmad A, Kahrizi K, Shaikh RS, Ali RA et al. (2011) Loss-of-function mutations of ILDR1 cause autosomal-recessive hearing impairment DFNB42. *Am J Hum Genet* 88: 127-37

Cao Y, Li L, Xu M, Feng Z, Sun X, Lu J, Xu Y, Du P, Wang T, Hu R, Ye Z, Shi L, Tang X, Yan L, Gao Z, Chen G, Zhang Y, Chen L, Ning G, Bi Y et al. (2020) The ChinaMAP analytics of deep whole genome sequences in 10,588 individuals. *Cell Res* 30: 717-731

Chadha S, Kamenov K, Cieza A (2021) The world report on hearing, 2021. *Bull World Health Organ* 99: 242-242A

Citi S, D'Atri F, Parry DA (2000) Human and Xenopus cingulin share a modular organization of the coiled-coil rod domain: predictions for intra- and intermolecular assembly. *J Struct Biol* 131: 135-45

Citi S, Pulimeno P, Paschoud S (2012) Cingulin, paracingulin, and PLEKHA7: signaling and cytoskeletal adaptors at the apical junctional complex. *Ann N Y Acad Sci* 1257: 125-32

Delmaghani S, Defourny J, Aghaie A, Beurg M, Dulon D, Thelen N, Perfettini I, Zelles T, Aller M, Meyer A, Emptoz A, Giraudet F, Leibovici M, Dartevelle S, Soubigou G, Thiry M, Vizi ES, Safieddine S, Hardelin JP, Avan P et al. (2015) Hypervulnerability to Sound Exposure through Impaired Adaptive Proliferation of Peroxisomes. *Cell* 163: 894-906

Du H, Ye C, Wu D, Zang YY, Zhang L, Chen C, He XY, Yang JJ, Hu P, Xu Z, Wan G, Shi YS (2020) The Cation Channel TMEM63B Is an Osmosensor Required for Hearing. *Cell Rep* 31: 107596

Du TT, Dewey JB, Wagner EL, Cui R, Heo J, Park JJ, Francis SP, Perez-Reyes E, Guillot SJ, Sherman NE, Xu W, Oghalai JS, Kachar B, Shin JB (2019) LMO7 deficiency reveals the significance of the cuticular plate for hearing function. *Nat Commun* 10: 1117

Gao X, Chen C, Shi S, Qian F, Liu D, Gong J (2023) Tight Junctions in the Auditory System: Structure, Distribution and Function. *Curr Protein Pept Sci* 24: 203-214

Gilels F, Paquette ST, Beaulac HJ, Bullen A, White PM (2017) Severe hearing loss and outer hair cell death in homozygous Foxo3 knockout mice after moderate noise exposure. *Sci Rep* 7: 1054

Gonzalez-Mariscal L, Dominguez-Calderon A, Raya-Sandino A, Ortega-Olvera JM, Vargas-Sierra O, Martinez-Revollar G (2014) Tight junctions and the regulation of gene expression. *Semin Cell Dev Biol* 36: 213-23

Gow A, Davies C, Southwood CM, Frolenkov G, Chrustowski M, Ng L, Yamauchi D, Marcus DC, Kachar B (2004) Deafness in Claudin 11-null mice reveals the critical contribution of basal cell tight junctions to stria vascularis function. *J Neurosci* 24: 7051-62

Liu H, Chen L, Giffen KP, Stringham ST, Li Y, Judge PD, Beisel KW, He DZZ (2018) Cell-Specific Transcriptome Analysis Shows That Adult Pillar and Deiters' Cells Express Genes Encoding Machinery for Specializations of Cochlear Hair Cells. *Front Mol Neurosci* 11: 356

Liu Y, Qi J, Chen X, Tang M, Chu C, Zhu W, Li H, Tian C, Yang G, Zhong C, Zhang Y, Ni G, He S, Chai R, Zhong G (2019) Critical role of spectrin in hearing development and deafness. *Sci Adv* 5: eaav7803

Morozko EL, Nishio A, Ingham NJ, Chandra R, Fitzgerald T, Martelletti E, Borck G, Wilson E, Riordan GP, Wangemann P, Forge A, Steel KP, Liddle RA, Friedman TB, Belyantseva IA (2015) ILDR1 null mice, a model of human deafness DFNB42, show structural aberrations of tricellular tight junctions and

degeneration of auditory hair cells. *Hum Mol Genet* 24: 609-24

Nayak G, Lee SI, Yousaf R, Edelmann SE, Trincot C, Van Itallie CM, Sinha GP, Rafeeq M, Jones SM, Belyantseva IA, Anderson JM, Forge A, Frolenkov GI, Riazuddin S (2013) Tricellulin deficiency affects tight junction architecture and cochlear hair cells. *J Clin Invest* 123: 4036-49

Petit C, Bonnet C, Safieddine S (2023) Deafness: from genetic architecture to gene therapy. *Nat Rev Genet*

Riazuddin S, Ahmed ZM, Fanning AS, Lagziel A, Kitajiri S, Ramzan K, Khan SN, Chattaraj P, Friedman PL, Anderson JM, Belyantseva IA, Forge A, Riazuddin S, Friedman TB (2006) Tricellulin is a tight-junction protein necessary for hearing. *Am J Hum Genet* 79: 1040-51

Rouaud F, Huang W, Flinois A, Jain K, Vasileva E, Di Mattia T, Mauperin M, Parry DAD, Dugina V, Chaponnier C, Mean I, Montessuit S, Mutero-Maeda A, Yan J, Citi S (2023) Cingulin and paracingulin tether myosins-2 to junctions to mechanoregulate the plasma membrane. *J Cell Biol* 222

Sawada N, Murata M, Kikuchi K, Osanai M, Tobioka H, Kojima T, Chiba H (2003) Tight junctions and human diseases. *Med Electron Microsc* 36: 147-56

Sineni CJ, Yildirim-Baylan M, Guo S, Camarena V, Wang G, Tokgoz-Yilmaz S, Duman D, Bademci G, Tekin M (2019) A truncating CLDN9 variant is associated with autosomal recessive nonsyndromic hearing loss. *Hum Genet* 138: 1071-1075

Walsh T, Pierce SB, Lenz DR, Brownstein Z, Dagan-Rosenfeld O, Shahin H, Roeb W, McCarthy S, Nord AS, Gordon CR, Ben-Neriah Z, Sebat J, Kanaan M, Lee MK, Frydman M, King MC, Avraham KB (2010) Genomic duplication and overexpression of TJP2/ZO-2 leads to altered expression of apoptosis genes in progressive nonsyndromic hearing loss DFNA51. *Am J Hum Genet* 87: 101-9

Wilcox ER, Burton QL, Naz S, Riazuddin S, Smith TN, Ploplis B, Belyantseva I, Ben-Yosef T, Liburd NA, Morell RJ, Kachar B, Wu DK, Griffith AJ, Riazuddin S, Friedman TB (2001) Mutations in the gene encoding tight junction claudin-14 cause autosomal recessive deafness DFNB29. *Cell* 104: 165-72

Yan D, Zhu Y, Walsh T, Xie D, Yuan H, Sirmaci A, Fujikawa T, Wong AC, Loh TL, Du L, Grati M, Vlajkovic SM, Blanton S, Ryan AF, Chen ZY, Thorne PR, Kachar B, Tekin M, Zhao HB, Housley GD et al. (2013) Mutation of the ATP-gated P2X(2) receptor leads to progressive hearing loss and increased susceptibility to noise. *Proc Natl Acad Sci U S A* 110: 2228-33

Zhu GJ, Gong S, Ma DB, Tao T, He WQ, Zhang L, Wang F, Qian XY, Zhou H, Fan C, Wang P, Chen X, Zhao W, Sun J, Chen H, Wang Y, Gao X, Zuo J, Zhu MS, Gao X et al. (2020) Aldh inhibitor restores auditory function in a mouse model of human deafness. *PLoS Genet* 16: e1009040

14th Aug 2023

Dear Dr. Wan,

Thank you for the submission of your manuscript to EMBO Molecular Medicine. I am pleased to inform you that we will be able to accept your manuscript pending the following final amendments:

1) Please address all referees' comments. Please pay particular attention to the grammar and syntax and I would recommend running the article by a native English speaker.
2) We note that you currently have together with you, a total of 4 co-corresponding authors. Is that correct? Do you confirm equal contribution of these 4 people, able to take full responsibility for the paper and its content? While there is no limit per se to the number of co-corresponding authors, 3 is rare, 4 even more so, and may not reflect as intended to the community.
3) In the main manuscript file, please do the following:
- Correct/answer the track changes suggested by our data editors by working from the attached document.
- Limit Keywords to max. 5.
- Remove font color.
- All figures should be called in a sequential order. Currently Fig 5G is called out before Fig 5F and Fig 7J before Fig 7G. Please check all the figures and correct.
- In M&M, a statistical paragraph should reflect all information that you have filled in the Authors Checklist, especially regarding randomization, blinding, replication.
- Remove "Availability of materials".
- Add "Disclosure Statement & Competing Interests". We updated our journal's competing interests policy in January 2022 and request authors to consider both actual and perceived competing interests. Please review the policy https://www.embopress.org/competing-interests and update your competing interests if necessary.
- Correct the reference citation in the reference list. Where there are more than 10 authors on a paper, 10 will be listed, followed by "et al.". Please check "Author Guidelines" for more information. https://www.embopress.org/page/journal/17574684/authorguide#referencesformat
- List all main figure legends after the "References" followed by all EV figure legends.
4) Appendix: Please add table of content with page numbers and correct nomenclature for Tables to Appendix Table S1 and Appendix Table S2.
5) The Paper Explained: Please provide "The Paper Explained" and add it to the main manuscript text. Please check "Author Guidelines" for more information. https://www.embopress.org/page/journal/17574684/authorguide#researcharticleguide
6) Synopsis:
- Synopsis text: Please remove the image form the synopsis text file.
- Synopsis image: Please resize it to a 550 pixels wide x 200-400 pixels high image.
- Please check your synopsis text and image, revise them if necessary and submit their final versions with your revised manuscript. Please be aware that in the proof stage minor corrections only are allowed (e.g., typos).
7) For more information: There is space at the end of each article to list relevant web links for further consultation by our readers. Could you identify some relevant ones and provide such information as well? Some examples are patient associations, relevant databases, OMIM/proteins/genes links, author's websites, etc...
8) Source data: Please zipp all source data for EV figures upload as one file. The same applies for Appendix figures and only source data for main figures should remain as one file per figure.
9) As part of the EMBO Publications transparent editorial process initiative (see our Editorial at http://embomolmed.embopress.org/content/2/9/329), EMBO Molecular Medicine will publish online a Review Process File (RPF) to accompany accepted manuscripts. This file will be published in conjunction with your paper and will include the anonymous referee reports, your point-by-point response and all pertinent correspondence relating to the manuscript. Let us know whether you agree with the publication of the RPF and as here, if you want to remove or not any figures from it prior to publication. Please note that the Authors checklist will be published at the end of the RPF.
10) Please provide a point-by-point letter INCLUDING my comments as well as the reviewer's reports and your detailed responses (as Word file).

I look forward to reading a new revised version of your manuscript as soon as possible.

Yours sincerely,

Zeljko Durdevic

Zeljko Durdevic
Editor
EMBO Molecular Medicine

*** Instructions to submit your revised manuscript ***

*** PLEASE NOTE *** As part of the EMBO Publications transparent editorial process initiative (see our Editorial at https://www.embopress.org/doi/pdf/10.1002/emmm.201000094), EMBO Molecular Medicine will publish online a Review Process File to accompany accepted manuscripts.

In the event of acceptance, this file will be published in conjunction with your paper and will include the anonymous referee reports, your point-by-point response and all pertinent correspondence relating to the manuscript. If you do NOT want this file to be published, please inform the editorial office at contact@embomolmed.org.

To submit your manuscript, please follow this link:

https://embomolmed.msubmit.net/cgi-bin/main.plex

When submitting your revised manuscript, please include:

1) a .docx formatted version of the manuscript text (including Figure legends and tables)

2) Separate figure files*

3) supplemental information as Expanded View and/or Appendix. Please carefully check the authors guidelines for formatting Expanded view and Appendix figures and tables at https://www.embopress.org/page/journal/17574684/authorguide#expandedview

4) a letter INCLUDING the reviewer's reports and your detailed responses to their comments (as Word file).

5) The paper explained: EMBO Molecular Medicine articles are accompanied by a summary of the articles to emphasize the major findings in the paper and their medical implications for the non-specialist reader. Please provide a draft summary of your article highlighting
- the medical issue you are addressing,
- the results obtained and
- their clinical impact.
This may be edited to ensure that readers understand the significance and context of the research.
Please refer to any of our published articles for an example.

6) For more information: There is space at the end of each article to list relevant web links for further consultation by our readers. Could you identify some relevant ones and provide such information as well? Some examples are patient associations, relevant databases, OMIM/proteins/genes links, author's websites, etc...

7) Author contributions: the contribution of every author must be detailed in a separate section.

8) EMBO Molecular Medicine now requires a complete author checklist (https://www.embopress.org/page/journal/17574684/authorguide) to be submitted with all revised manuscripts. Please use the checklist as guideline for the sort of information we need WITHIN the manuscript. The checklist should only be filled with page numbers were the information can be found. This is particularly important for animal reporting, antibody dilutions (missing) and exact values and n that should be indicted instead of a range.

9) Every published paper now includes a 'Synopsis' to further enhance discoverability. Synopses are displayed on the journal webpage and are freely accessible to all readers. They include a short stand first (maximum of 300 characters, including space) as well as 2-5 one sentence bullet points that summarise the paper. Please write the bullet points to summarise the key NEW findings. They should be designed to be complementary to the abstract - i.e. not repeat the same text. We encourage inclusion of key acronyms and quantitative information (maximum of 30 words / bullet point). Please use the passive voice. Please attach these in a separate file or send them by email, we will incorporate them accordingly.

You are also welcome to suggest a striking image or visual abstract to illustrate your article. If you do please provide a jpeg file 550 px-wide x 400-px high.

10) A Conflict of Interest statement should be provided in the main text

11) Please note that we now mandate that all corresponding authors list an ORCID digital identifier. This takes <90 seconds to complete. We encourage all authors to supply an ORCID identifier, which will be linked to their name for unambiguous name identification.

Currently, our records indicate that the ORCID for your account is 0000-0003-3841-9301.

Please click the link below to modify this ORCID:
Link Not Available

12) The system will prompt you to fill in your funding and payment information. This will allow Wiley to send you a quote for the article processing charge (APC) in case of acceptance. This quote takes into account any reduction or fee waivers that you may be eligible for. Authors do not need to pay any fees before their manuscript is accepted and transferred to our publisher.

*Additional important information regarding Figures

Each figure should be given in a separate file and should have the following resolution:
Graphs 800-1,200 DPI
Photos 400-800 DPI
Colour (only CMYK) 300-400 DPI"

Figures are not edited by the production team. All lettering should be the same size and style; figure panels should be indicated by capital letters (A, B, C etc). Gridlines are not allowed except for log plots. Figures should be numbered in the order of their appearance in the text with Arabic numerals. Each Figure must have a separate legend and a caption is needed for each panel.

*Additional important information regarding figures and illustrations can be found at https://bit.ly/EMBOPressFigurePreparationGuideline. See also figure legend preparation guidelines: https://www.embopress.org/page/journal/17574684/authorguide#figureformat

The system will prompt you to fill in your funding and payment information. This will allow Wiley to send you a quote for the article processing charge (APC) in case of acceptance. This quote takes into account any reduction or fee waivers that you may be eligible for. Authors do not need to pay any fees before their manuscript is accepted and transferred to our publisher.

***** Reviewer's comments *****

Referee #1 (Remarks for Author):

The revised manuscript from Zhu and co-authors entitled "Cingulin regulates hair cell cuticular plate morphology and is required for hearing in human and mouse" is substantially improved. The responses to reviewers are appropriate and solve concerns raised by reviewers. However, the revised manuscript still has numerous minor issues suggesting that the manuscript was not carefully edited by a person knowledgeable about written English and genetics. As examples, a few issues are listed below.

Figure 1, species names in panel B, E, D and F need a space between the genus and specie. H. sapiens not H.sapiens

Introduction, first sentence, "Based on a recent world report...." Add 'a".

Introduction, end of the 2nd paragraph, "... for personalized treatment of [delete the] hereditary deafness cases."

Results, 2nd paragraph, line 8, "...and were thus excluded." Excluded from exactly what? Finish the thought. Excluded from further analyses?

Results, 2nd page, third paragraph, Gene names need to be italicized. Italicize Cgn.

Results, 3rd page, line 6 and elsewhere in the manuscript, protein names in human and mouse are all caps regardless of what has been published previously. CGN not Cgn.

Referee #2 (Comments on Novelty/Model System for Author):

mouse model is appropriate

Referee #2 (Remarks for Author):

In the revised version, the authors have addressed most of the concerns raised by the reviewers, including the addition of SEM images for hair bundle morphology and testing vestibular function. With these additional data, the article now provides a comprehensive characterization of the phenotypes in CGN deficiency mice. While there are still areas for future exploration, such as delving into the molecular function of CGN in hair cells, the current state of the article meets the standards for publication in EMBO Molecular Medicine, provided a few minor changes are made:

1. The authors need to make grammar and spelling corrections.
1. In Figure 2G, the immunofluorescence labeling indicates that CGN is more pronounced in the cuticular plates of outer hair cells (OHCs) compared to inner hair cells (IHCs). However, the author did not specify which hair cell type was quantified in the right panel of the figure.
2. Please clarify the normalization method used in the qPCR experiments. For instance, in Figure 3D, the y-axis represents fold changes, however, the details of the method are not provided.
3. In Figure 2G, 7G-I, L-N, it is not clear what the replicates represent in these figures. If each data point represents a cell, then the p-value could potentially be affected by oversampling.

Response to the editor (2nd Revision)

Dear Dr. Wan,

Thank you for the submission of your manuscript to EMBO Molecular Medicine. I am pleased to inform you that we will be able to accept your manuscript pending the following final amendments:

1) Please address all referees' comments. Please pay particular attention to the grammar and syntax and I would recommend running the article by a native English speaker.

Response: We have addressed all reviewers' comments listed below. In addition, we have now consulted two colleagues at University of Michigan (Drs. Gabriel Corfas and David Kohrman) who helped us revise the final manuscript, including grammar and syntax.

2) We note that you currently have together with you, a total of 4 co-corresponding authors. Is that correct? Do you confirm equal contribution of these 4 people, able to take full responsibility for the paper and its content? While there is no limit per se to the number of co-corresponding authors, 3 is rare, 4 even more so, and may not reflect as intended to the community.

Response: I confirm the equal contributions of these 4 corresponding authors. This work is a result of collective efforts and shared expertise from various aspects of clinical genetics, animal models, hearing physiology and histology.

3) In the main manuscript file, please do the following:
- Correct/answer the track changes suggested by our data editors by working from the attached document.

Response: We have corrected the track changes made by data editor. The track changes and comments were replicated to the final manuscript file (figure QC file). In the comment window, queries by the data editor are highlighted in red font and our replies below in black font.

- Limit Keywords to max. 5.

Response: Done.

- Remove font color.

Response: Done.

- All figures should be called in a sequential order. Currently Fig 5G is called out before Fig 5F and Fig 7J before Fig 7G. Please check all the figures and correct.

Response: Corrected.

- In M&M, a statistical paragraph should reflect all information that you have filled in the Authors Checklist, especially regarding randomization, blinding, replication.

Response: We have revised the materials and methods to include this important information.

- Remove "Availability of materials".

Response: Done.

- Add "Disclosure Statement & Competing Interests". We updated our journal's competing interests policy in January 2022 and request authors to consider both actual and perceived competing

interests. Please review the policy https://www.embopress.org/competing-interests and update your competing interests if necessary.

Response: Added “The authors declare that they have no conflict of interest.”

- Correct the reference citation in the reference list. Where there are more than 10 authors on a paper, 10 will be listed, followed by "et al.". Please check "Author Guidelines" for more information. https://www.embopress.org/page/journal/17574684/authorguide#referencesformat

Response: Corrected.

- List all main figure legends after the "References" followed by all EV figure legends.

Response: Done.

4) Appendix: Please add table of content with page numbers and correct nomenclature for Tables to Appendix Table S1 and Appendix Table S2.

Response: Done.

5) The Paper Explained: Please provide "The Paper Explained" and add it to the main manuscript text. Please check "Author Guidelines" for more information. https://www.embopress.org/page/journal/17574684/authorguide#researcharticleguide

Response: Done.

6) Synopsis:
- Synopsis text: Please remove the image form the synopsis text file.
- Synopsis image: Please resize it to a 550 pixels wide x 200-400 pixels high image.
- Please check your synopsis text and image, revise them if necessary and submit their final versions with your revised manuscript. Please be aware that in the proof stage minor corrections only are allowed (e.g., typos).

Response: Done.

7) For more information: There is space at the end of each article to list relevant web links for further consultation by our readers. Could you identify some relevant ones and provide such information as well? Some examples are patient associations, relevant databases, OMIM/proteins/genes links, author's websites, etc...

Response: Added.

8) Source data: Please zip all source data for EV figures upload as one file. The same applies for Appendix figures and only source data for main figures should remain as one file per figure.

Response: Done.

9) As part of the EMBO Publications transparent editorial process initiative (see our Editorial at http://embomolmed.embopress.org/content/2/9/329), EMBO Molecular Medicine will publish online a Review Process File (RPF) to accompany accepted manuscripts. This file will be published in conjunction with your paper and will include the anonymous referee reports, your point-by-point response and all pertinent correspondence relating to the manuscript. Let us know whether you agree with the publication of the RPF and as here, if you want to remove or not any figures from it prior to publication. Please note that the Authors checklist will be published at the end of the RPF.

Response: We agree with publication of RPF as the current form.

10) Please provide a point-by-point letter INCLUDING my comments as well as the reviewer's reports and your detailed responses (as Word file).

Response: Done.

Point-to-point response to reviewers (2nd Revision)

We thank the reviewers for positive comments of the work and insightful suggestions that have greatly improved our manuscript.

***** Reviewer's comments *****

Referee #1 (Remarks for Author):

The revised manuscript from Zhu and co-authors entitled "Cingulin regulates hair cell cuticular plate morphology and is required for hearing in human and mouse" is substantially improved. The responses to reviewers are appropriate and solve concerns raised by reviewers. However, the revised manuscript still has numerous minor issues suggesting that the manuscript was not carefully edited by a person knowledgeable about written English and genetics. As examples, a few issues are listed below.

Response: We thank the reviewer for the positive overall comment. We have now consulted two experts/colleagues at University of Michigan who helped us revise the final manuscript, including grammar and syntax.

Figure 1, species names in panel B, E, D and F need a space between the genus and specie. H. sapiens not H.sapiens

Response: Corrected.

Introduction, first sentence, "Based on a recent world report...." Add 'a".

Response: We've revised this sentence.

Introduction, end of the 2nd paragraph, "... for personalized treatment of [delete the] hereditary deafness cases."

Response: Corrected.

Results, 2nd paragraph, line 8, "...and were thus excluded." Excluded from exactly what? Finish the thought. Excluded from further analyses?

Response: We've revised to "… excluded from further analyses."

Results, 2nd page, third paragraph, Gene names need to be italicized. Italicize Cgn.

Response: Corrected.

Results, 3rd page, line 6 and elsewhere in the manuscript, protein names in human and mouse are all caps regardless of what has been published previously. CGN not Cgn.

Response: Corrected, thanks.

Referee #2 (Comments on Novelty/Model System for Author):

mouse model is appropriate

Referee #2 (Remarks for Author):

In the revised version, the authors have addressed most of the concerns raised by the reviewers, including the addition of SEM images for hair bundle morphology and testing vestibular function. With these additional data, the article now provides a comprehensive characterization of the phenotypes in CGN deficiency mice. While there are still areas for future exploration, such as delving into the molecular function of CGN in hair cells, the current state of the article meets the standards for publication in EMBO Molecular Medicine, provided a few minor changes are made:

Response: We thank the reviewer for positive evaluation of our manuscript.

1. The authors need to make grammar and spelling corrections.

Response: We have made numerous corrections to grammar and syntax with the help of native English speakers and auditory neuroscientists.

2. In Figure 2G, the immunofluorescence labeling indicates that CGN is more pronounced in the cuticular plates of outer hair cells (OHCs) compared to inner hair cells (IHCs). However, the author did not specify which hair cell type was quantified in the right panel of the figure.

Response: We quantified the signals from OHCs. This information is added in the results section of revised manuscript.

3. Please clarify the normalization method used in the qPCR experiments. For instance, in Figure 3D, the y-axis represents fold changes, however, the details of the method are not provided.

Response: We have now included the normalization method in the materials and methods under the section of RT-qPCR.

"Expression of target genes was normalized to the internal reference genes (*GAPDH* for human and mouse cells/tissues, *Actb/β-actin* for the canine MDCK cells). Fold changes of expression were normalized to control treatments or wildtype CGN control samples."

4. In Figure 2G, 7G-I, L-N, it is not clear what the replicates represent in these figures. If each data point represents a cell, then the p-value could potentially be affected by oversampling.

Response: The replicate numbers represent numbers of hair cells pulled from 3-4 cochleae of each genotype, as specified in the figure legends. We agree that the concern of oversampling skewing the p-value maybe valid, this method is useful for detecting subtle or highly variable phenotypes where structure of each cell is individually affected (and thus considered as an individual sample point). This method (of pulling individual hair cells from multiple cochleae/mice) is not uncommon for studying hair cell subcellular structures, such as stereocilia morphologies (Gao *et al*, 2022; Krey *et al*, 2022; Zhai *et al*, 2022) and ribbon synapses (Hua *et al*, 2021; Jean *et al*, 2019; Rutherford *et al*, 2023).

References for Reviewers

Gao G, Guo S, Zhang Q, Zhang H, Zhang C, Peng G (2022) Kiaa1024L/Minar2 is essential for hearing by regulating cholesterol distribution in hair bundles. *Elife* 11

Hua Y, Ding X, Wang H, Wang F, Lu Y, Neef J, Gao Y, Moser T, Wu H (2021) Electron Microscopic Reconstruction of Neural Circuitry in the Cochlea. *Cell Rep* 34: 108551

Jean P, Ozcete OD, Tarchini B, Moser T (2019) Intrinsic planar polarity mechanisms influence the position-dependent regulation of synapse properties in inner hair cells. *Proc Natl Acad Sci U S A* 116: 9084-9093

Krey JF, Liu C, Belyantseva IA, Bateschell M, Dumont RA, Goldsmith J, Chatterjee P, Morrill RS, Fedorov LM, Foster S *et al* (2022) ANKRD24 organizes TRIOBP to reinforce stereocilia insertion points. *J Cell Biol* 221

Rutherford MA, Bhattacharyya A, Xiao M, Cai HM, Pal I, Rubio ME (2023) GluA3 subunits are required for appropriate assembly of AMPAR GluA2 and GluA4 subunits on cochlear afferent synapses and for presynaptic ribbon modiolar-pillar morphology. *Elife* 12
Zhai X, Du H, Shen Y, Zhang X, Chen Z, Wang Y, Xu Z (2022) FCHSD2 is required for stereocilia maintenance in mouse cochlear hair cells. *J Cell Sci* 135

22nd Aug 2023

Dear Dr. Wan,

We are pleased to inform you that your manuscript is accepted for publication and is now being sent to our publisher to be included in the next available issue of EMBO Molecular Medicine.

Please read below for additional IMPORTANT information regarding your article, its publication and the production process.

Congratulations on your interesting work,

Zeljko Durdevic

Zeljko Durdevic
Editor
EMBO Molecular Medicine

Follow us on Twitter @EmboMolMed
Sign up for eTOCs at embopress.org/alertsfeeds

*** *** *** IMPORTANT INFORMATION *** *** ***

SPEED OF PUBLICATION
The journal aims for rapid publication of papers, using using the advance online publication "Early View" to expedite the process: A properly copy-edited and formatted version will be published as "Early View" after the proofs have been corrected. Please help the Editors and publisher avoid delays by providing e-mail address(es), telephone and fax numbers at which author(s) can be contacted.

Should you be planning a Press Release on your article, please get in contact with embomolmed@wiley.com as early as possible, in order to coordinate publication and release dates.

LICENSE AND PAYMENT:

All articles published in EMBO Molecular Medicine are fully open access: immediately and freely available to read, download and share.

EMBO Molecular Medicine charges an article processing charge (APC) to cover the publication costs. You, as the corresponding author for this manuscript, should have already received a quote with the article processing fee separately. Please let us know in case this quote has not been received.

Once your article is at Wiley for editorial production you will receive an email from Wiley's Author Services system, which will ask you to log in and will present you with the publication license form for completion. Within the same system the publication fee can be paid by credit card, an invoice, pro forma invoice or purchase order can be requested.

Payment of the publication charge and the signed Open Access Agreement form must be received before the article can be published online.

PROOFS

You will receive the proofs by e-mail approximately 2 weeks after all relevant files have been sent o our Production Office. Please return them within 48 hours and if there should be any problems, please contact the production office at embopressproduction@wiley.com.

Please inform us if there is likely to be any difficulty in reaching you at the above address at that time. Failure to meet our

deadlines may result in a delay of publication.

All further communications concerning your paper proofs should quote reference number EMM-2023-17611-V3 and be directed to the production office at embopressproduction@wiley.com.

Thank you,

Zeljko Durdevic
Editor
EMBO Molecular Medicine

EMBO Press Author Checklist

Corresponding Author Name: Guoqiang Wan	
Journal Submitted to: EMBO Molecular Medicine	
Manuscript Number: EMM-2023-17611	

USEFUL LINKS FOR COMPLETING THIS FORM
The EMBO Journal - Author Guidelines
EMBO Reports - Author Guidelines
Molecular Systems Biology - Author Guidelines
EMBO Molecular Medicine - Author Guidelines

Reporting Checklist for Life Science Articles (updated January 2022)

This checklist is adapted from Materials Design Analysis Reporting (MDAR) Checklist for Authors. MDAR establishes a minimum set of requirements in transparent reporting in the life sciences (see Statement of Task: 10.31222/osf.io/9sm4x). Please follow the journal's guidelines in preparing your manuscript.

Please note that a copy of this checklist will be published alongside your article.

Abridged guidelines for figures

1. Data

The data shown in figures should satisfy the following conditions:

- the data were obtained and processed according to the field's best practice and are presented to reflect the results of the experiments in an accurate and unbiased manner.
- ideally, figure panels should include only measurements that are directly comparable to each other and obtained with the same assay.
- plots include clearly labeled error bars for independent experiments and sample sizes. Unless justified, error bars should not be shown for technical replicates.
- if n<5, the individual data points from each experiment should be plotted. Any statistical test employed should be justified.
- Source Data should be included to report the data underlying figures according to the guidelines set out in the authorship guidelines on Data Presentation.

2. Captions

Each figure caption should contain the following information, for each panel where they are relevant:

- a specification of the experimental system investigated (eg cell line, species name).
- the assay(s) and method(s) used to carry out the reported observations and measurements.
- an explicit mention of the biological and chemical entity(ies) that are being measured.
- an explicit mention of the biological and chemical entity(ies) that are altered/varied/perturbed in a controlled manner.
- the exact sample size (n) for each experimental group/condition, given as a number, not a range;
- a description of the sample collection allowing the reader to understand whether the samples represent technical or biological replicates (including how many animals, litters, cultures, etc.).
- a statement of how many times the experiment shown was independently replicated in the laboratory.
- definitions of statistical methods and measures:
  - common tests, such as t-test (please specify whether paired vs. unpaired), simple χ2 tests, Wilcoxon and Mann-Whitney tests, can be unambiguously identified by name only, but more complex techniques should be described in the methods section;
  - are tests one-sided or two-sided?
  - are there adjustments for multiple comparisons?
  - exact statistical test results, e.g., P values = x but not P values < x;
  - definition of 'center values' as median or average;
  - definition of error bars as s.d. or s.e.m.

Please complete ALL of the questions below.
Select "Not Applicable" only when the requested information is not relevant for your study.

Materials

Newly Created Materials	Information included in the manuscript?	In which section is the information available? (Reagents and Tools Table, Materials and Methods, Figures, Data Availability Section)
New materials and reagents need to be available; do any restrictions apply?	Yes	Materials and Methods, Data Availability Section

Antibodies	Information included in the manuscript?	In which section is the information available? (Reagents and Tools Table, Materials and Methods, Figures, Data Availability Section)
For **antibodies** provide the following information:<br>- Commercial antibodies: RRID (if possible) or supplier name, catalogue number and or/clone number<br>- Non-commercial: RRID or citation	Yes	Materials and Methods

DNA and RNA sequences	Information included in the manuscript?	In which section is the information available? (Reagents and Tools Table, Materials and Methods, Figures, Data Availability Section)
**Short novel DNA or RNA including primers, probes**: provide the sequences.	Yes	Materials and Methods, Appendix Table S2

Cell materials	Information included in the manuscript?	In which section is the information available? (Reagents and Tools Table, Materials and Methods, Figures, Data Availability Section)
**Cell lines:** Provide species information, strain. Provide accession number in repository **OR** supplier name, catalog number, clone number, and/**OR** RRID.	Yes	Materials and Methods
**Primary cultures:** Provide species, strain, sex of origin, genetic modification status.	Not Applicable	
Report if the cell lines were recently **authenticated** (e.g., by STR profiling) and tested for mycoplasma contamination.	Yes	Materials and Methods

Experimental animals	Information included in the manuscript?	In which section is the information available? (Reagents and Tools Table, Materials and Methods, Figures, Data Availability Section)
**Laboratory animals or Model organisms:** Provide species, strain, sex, age, genetic modification status. Provide accession number in repository **OR** supplier name, catalog number, clone number, **OR** RRID.	Yes	Materials and Methods, Results, Figure legends
**Animal observed in or captured from the field:** Provide species, sex, and age where possible.	Not Applicable	
Please detail **housing and husbandry conditions**.	Yes	Materials and Methods

Plants and microbes	Information included in the manuscript?	In which section is the information available? (Reagents and Tools Table, Materials and Methods, Figures, Data Availability Section)
**Plants:** provide species and strain, ecotype and cultivar where relevant, unique accession number if available, and source (including location for collected wild specimens).	Not Applicable	
**Microbes:** provide species and strain, unique accession number if available, and source.	Not Applicable	

Human research participants	Information included in the manuscript?	In which section is the information available? (Reagents and Tools Table, Materials and Methods, Figures, Data Availability Section)
If collected and within the bounds of privacy constraints report on age, sex and gender or ethnicity for all study participants.	Yes	Materials and Methods

Core facilities	Information included in the manuscript?	In which section is the information available? (Reagents and Tools Table, Materials and Methods, Figures, Data Availability Section)
If your work benefited from core facilities, was their service mentioned in the acknowledgments section?	Not Applicable	

Design

Study protocol	Information included in the manuscript?	In which section is the information available? (Reagents and Tools Table, Materials and Methods, Figures, Data Availability Section)
If study protocol has been **pre-registered, provide DOI in the manuscript**. For clinical trials, provide the trial registration number **OR** cite DOI.	Not Applicable	
Report the **clinical trial registration number** (at ClinicalTrials.gov or equivalent), where applicable.	Not Applicable	

Laboratory protocol	Information included in the manuscript?	In which section is the information available? (Reagents and Tools Table, Materials and Methods, Figures, Data Availability Section)
Provide DOI OR other citation details if **external detailed step-by-step protocols** are available.	Not Applicable	

Experimental study design and statistics	Information included in the manuscript?	In which section is the information available? (Reagents and Tools Table, Materials and Methods, Figures, Data Availability Section)
Include a statement about **sample size** estimate even if no statistical methods were used.	Not Applicable	
Were any steps taken to minimize the effects of subjective bias when allocating animals/samples to treatment (e.g. **randomization procedure**)? If yes, have they been described?	Yes	Materials and Methods
Include a statement about **blinding** even if no blinding was done.	Yes	Materials and Methods
Describe **inclusion/exclusion criteria** if samples or animals were excluded from the analysis. Were the criteria pre-established?<br><br>If sample or data points were omitted from analysis, report if this was due to attrition or intentional exclusion and provide justification.	Not Applicable	
For every figure, are **statistical tests** justified as appropriate? Do the data meet the assumptions of the tests (e.g., normal distribution)? Describe any methods used to assess it. Is there an estimate of variation within each group of data? Is the variance similar between the groups that are being statistically compared?	Yes	Figure legends

Sample definition and in-laboratory replication	Information included in the manuscript?	In which section is the information available? (Reagents and Tools Table, Materials and Methods, Figures, Data Availability Section)
In the figure legends: state number of times the experiment was **replicated** in laboratory.	Yes	Figure legends
In the figure legends: define whether data describe **technical or biological replicates**.	Yes	Figure legends

Ethics

Ethics	Information included in the manuscript?	In which section is the information available? (Reagents and Tools Table, Materials and Methods, Figures, Data Availability Section)
Studies involving **human participants**: State details of **authority granting ethics approval** (IRB or equivalent committee(s), provide reference number for approval.	Yes	Materials and Methods
Studies involving **human participants**: Include a statement confirming that **informed consent** was obtained from all subjects and that the experiments conformed to the principles set out in the WMA Declaration of Helsinki and the Department of Health and Human Services Belmont Report.	Yes	Materials and Methods
Studies involving **human participants:** For publication of **patient photos**, include a statement confirming that consent to publish was obtained.	Not Applicable	
Studies involving experimental **animals**: State details of **authority granting ethics approval** (IRB or equivalent committee(s), provide reference number for approval. Include a statement of compliance with ethical regulations.	Yes	Materials and Methods
Studies involving **specimen and field samples:** State if relevant **permits** obtained, provide details of authority approving study; if none were required, explain why.	Not Applicable	

Dual Use Research of Concern (DURC)	Information included in the manuscript?	In which section is the information available? (Reagents and Tools Table, Materials and Methods, Figures, Data Availability Section)
Could your study fall under dual use research restrictions? Please check biosecurity documents and list of **select agents and toxins** (CDC): https://www.selectagents.gov/sat/list.htm	Not Applicable	
If you used a select agent, is the security level of the lab appropriate and reported in the manuscript?	Not Applicable	
If a study is subject to dual use research of concern regulations, is the name of the **authority granting approval and reference number** for the regulatory approval provided in the manuscript?	Not Applicable	

Reporting

The MDAR framework recommends adoption of discipline-specific guidelines, established and endorsed through community initiatives. Journals have their own policy about requiring specific guidelines and recommendations to complement MDAR.

Adherence to community standards	Information included in the manuscript?	In which section is the information available? (Reagents and Tools Table, Materials and Methods, Figures, Data Availability Section)
State if relevant guidelines or checklists (e.g., **ICMJE, MIBBI, ARRIVE, PRISMA**) have been followed or provided.	Not Applicable	
For **tumor marker prognostic studies,** we recommend that you follow the **REMARK** reporting guidelines (see link list at top right). See author guidelines, under 'Reporting Guidelines'. Please confirm you have followed these guidelines.	Not Applicable	
For **phase II and III randomized controlled trials**, please refer to the **CONSORT** flow diagram (see link list at top right) and submit the CONSORT checklist (see link list at top right) with your submission. See author guidelines, under 'Reporting Guidelines'. Please confirm you have submitted this list.	Not Applicable	

Data Availability

Data availability	Information included in the manuscript?	In which section is the information available? (Reagents and Tools Table, Materials and Methods, Figures, Data Availability Section)
Have **primary datasets** been deposited according to the journal's guidelines (see 'Data Deposition' section) and the respective accession numbers provided in the Data Availability Section?	Yes	Data Availability Section
Were **human clinical and genomic datasets** deposited in a public access-controlled repository in accordance to ethical obligations to the patients and to the applicable consent agreement?	Yes	Materials and Methods
Are **computational models** that are central and integral to a study available without restrictions in a machine-readable form? Were the relevant accession numbers or links provided?	Not Applicable	
If publicly available data were reused, provide the respective **data citations in the reference list**.	Yes	References
